# Modular synthesis of bis-α-chiral amines using Ellman sulfinamide for consecutive *S*-to-*C* chirality induction/transfer

**DOI:** 10.1126/sciadv.adv2010

**Published:** 2025-04-04

**Authors:** Guangwu Sun, Herui Liu, Baobiao Dong, Yuchao Zhang, Zilong Zhao, Bing Gao

**Affiliations:** ^1^ State Key Laboratory of Chemo and Biosensing, College of Chemistry and Chemical Engineering, Hunan University, Changsha, Hunan 410082, China.; ^2^ Institute of Drug Discovery and Design, College of Pharmaceutical Sciences, Zhejiang University, Hangzhou, Zhejiang 310058, China.; ^3^ Scientific Experiment Center, Hangzhou Institute of Medicine, Chinese Academy of Sciences, Hangzhou, Zhejiang 310022, China.

## Abstract

Amines are ubiquitous components in pharmaceuticals. Increasing saturated substitutions (*sp^3^*-hybridized carbon) at the amino center and the number of chiral centers can enrich the molecular diversity and chemical space, ultimately enhancing the success of drug development. However, the synthesis of such advanced amines is challenging due to a higher level of structural complexity and stereo-control. Here, we report a modular protocol for short de novo synthesis of bis-α-chiral amines. This protocol uses commercially available Ellman sulfinamide, *tert*-butanesulfinamide (*^t^*BS), as the exclusive chiral source to selectively produce all possible stereoisomers. Sequential formation of contiguous α-amino chiral carbons is achieved by chirality induction and transfer mechanisms that are both enabled by *^t^*BS, the stereoselective imine functionalization and alkyne-participated rearrangement reaction. The second step we developed is crucial for high diastereoselectivity, which is problematic in previous methods. The other coupling partners used in this protocol are abundant feedstocks, providing desirable chemical diversity in the products.

## Teaser

Highly branched secondary amines are synthesized through *tert*-butanesulfinamide–mediated stepwise stereochemical control.

## INTRODUCTION

**Fig. 1.**
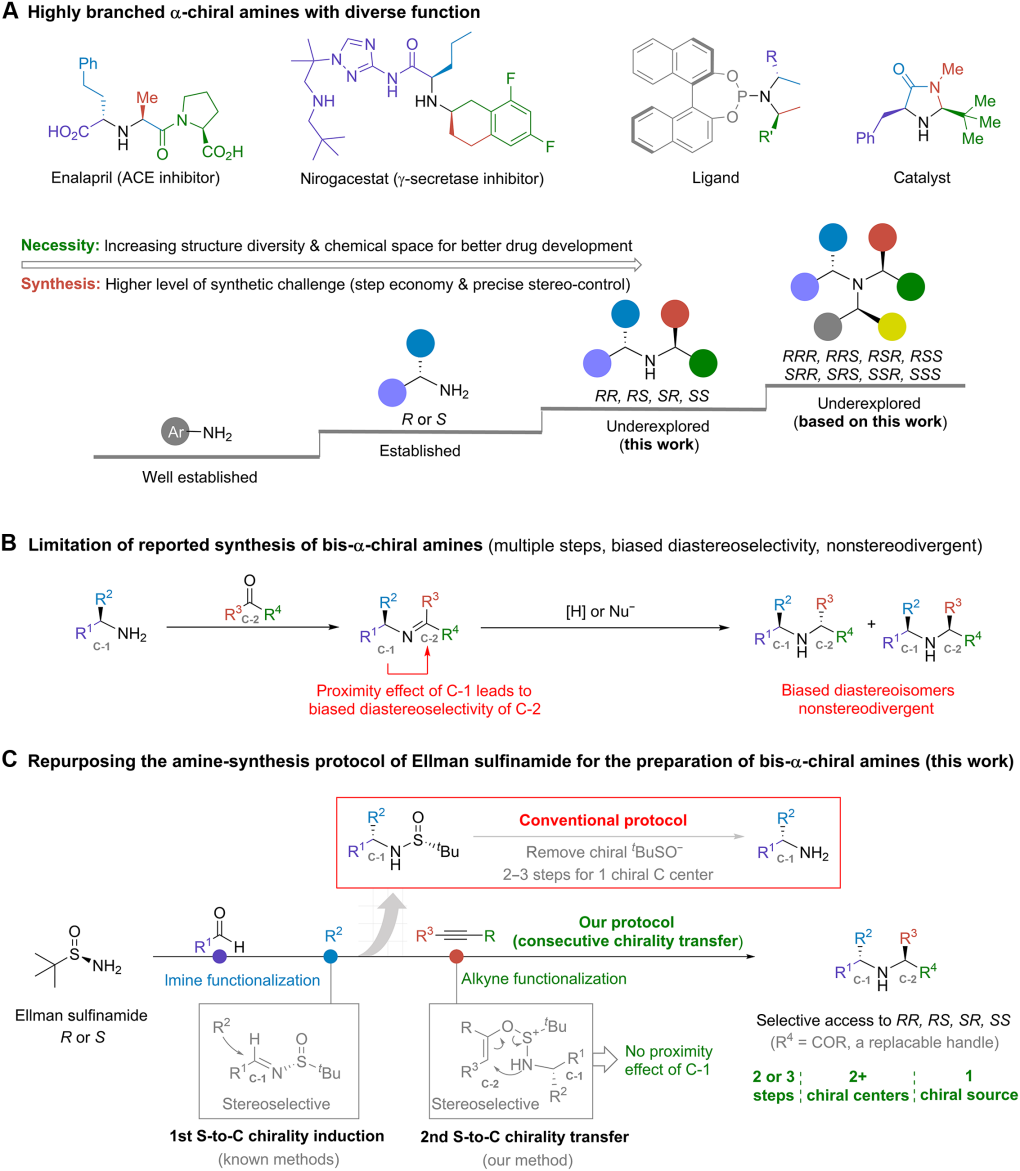
Unsymmetric secondary amines bearing two α-chiral carbons and our synthetic strategy. **(A)** functional molecules of chiral amines. **(B)** limitations of previous synthetic approaches. **(C)** our proposed protocol.

Chiral amines are key units of bioactive molecules in supporting and regulating biological events. The excellent bioavailability of amino fragments has also spurred the development of corresponding unnatural molecules for disease treatment (*1*), accounting for approximately 40% of the top 200 small molecular drugs by sales in 2023 (*2*). The growing demand from the pharmaceutical industry, as well as other relevant fields, has stimulated continuous efforts toward their synthesis (*3*). Many methods have been established for the asymmetric preparation of amines with one α-chiral carbon, including reductive amination (*4*–*9*), imine functionalization (*10*, *11*), alkene amination (*12*, *13*), carbene insertion (*14*, *15*), and *N*-alkylation (*16*–*18*). Research has shown that further incorporation of saturated substituents to the amino center, specifically the *sp*^3^-hybridized carbons to form highly branched secondary or tertiary alkyl amines, can expand the chemical space and molecular complexity (*19*–*22*), thereby enhancing the success of drug development and relevant function discovery (*23*–*25*). However, given the intricate structure and precise stereocontrol involved, obtaining these advanced molecules poses a long-standing challenge to synthetic chemists (Fig. 1A).

To date, the preparation of pyrrolidine derivatives through 1,3-dipolar cycloaddition reactions is a main method that allows synergistic formation of two α-chiral carbons (*26*, *27*). For the acyclic and unsymmetric secondary amines featuring two α-chiral carbons, multiple synthetic steps are required, and no general method is available to selectively access all stereoisomers (*28*). In a few reports, the optically pure α-chiral primary amines, derived from organic synthesis or natural sources, were converted to bis-α-chiral secondary amines via imine functionalization. However, the existing chiral center (C-1) in substrates often interferes with the stereoselectivity of the second chiral carbon (C-2; Fig. 1B), leading to biased formation of specific diastereoisomer (*29*–*31*). The proximity effect is unavoidable, making the corresponding reactions unsuitable for divergent synthesis. Recently, the Buchwald group (*13*) developed a copper catalysis for alkene hydroamination. The stereochemistry of the C─N formation was ligand-controlled that exhibited negligible influence from the existing α-chiral carbon in substrates, implying its potential application for stereodivergent synthesis of highly branched amines. One limitation was the requirement of hydroxylamine esters to gain umpolung reactivity at the amino center. The direct use of nucleophilic amines for asymmetric *N*-alkylation is an emerging field (*16*–*18*). Despite these advances, a general and practical method for modular synthesis of advanced bis-α-chiral amines, with precise control over the formation of any desired stereoisomers, remains unavailable.

Here, we disclose a general strategy for the stereodivergent synthesis of advanced amines carrying two or more chiral carbons (Fig. 1C). It provides selective access to all stereoisomers via consecutive chirality induction and transfer events, using *tert*-butanesulfinamide (*^t^*BS) reagent as an exclusive chiral source. *^t^*BS was introduced by Ellman in 1997 as a chiral auxiliary and amino source. It has shown excellent stereocontrol across a wide range of reactions and is especially effective for the preparation of primary chiral amines via imine intermediates (*32*–*34*). According to our recent survey, *^t^*BS has been used in thousands of reports with more than half of them patented for important applications (see the Supplementary Materials and fig. S1) (*35*). Unfortunately, the optically pure *tert-*butanesulfinyl group was removed from the final products as a waste at the final step of syntheses (Fig. 1C and fig. S2A). We and the Maulide group (*36*–*38*) recently reported that *^t^*BS derivatives could facilitate a [2,3]-rearrangement reaction with activated alkynes or amides, affording enantioenriched α-amino carbonyl skeletons. In this context, we hypothesized that integrating two distinct *^t^*BS-enabled chirality induction/transfer events, the traditional imine functionalization and our [2,3]-rearrangement reaction, might establish a general platform for stereoselective synthesis of secondary chiral amines. It would be attractive for medicinal discovery due to its simplicity, which does not require precious transition metal catalysts or chiral ligands and affords the desired scaffolds and stereochemistry in a modular and predictable manner.

## RESULTS

**Fig. 2.**
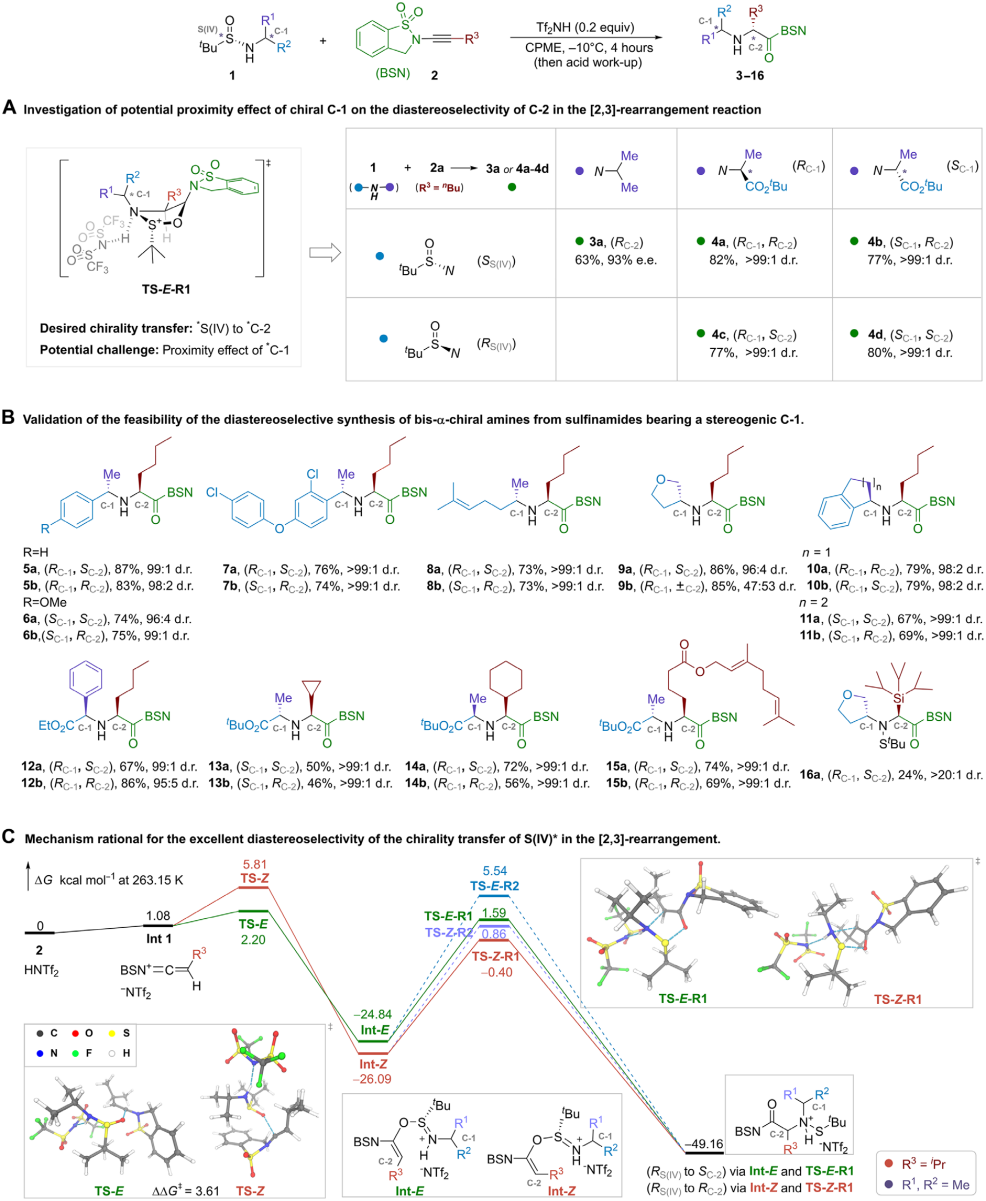
The stereochemistry and scope of the Ellman sulfinamide enabled [2,3]-rearrangement reaction. **(A)** Study of the diastereoselectivity in the rearrangement reaction. **(B)** Scope of the asymmetric rearrangement reaction. **(C)** Density functional theory calculations.

Given the notorious proximity effect that an existing α-amino chiral carbon can affect the stereochemistry of the newly formed chiral center in previous methods (Fig. 1B), the second-step chirality transfer using our *^t^*BS-enabled [2,3]-rearrangement is crucial for the success of the proposed protocol (Fig. 1C). Therefore, we reinvestigated this reaction and its diastereoselectivity (Fig. 2). The model reaction of (*S*_S(IV)_)-*N*-*iso*-propyl *^t^*BS substrate **1a** and benzosulfonamide (BSN) ynamide **2a** under acidic conditions afforded secondary amine (*R*_c-2_)-**3a** in 63% yield with 93% e.e. (enantiomeric excess; Fig. 2A; more information for studying the ynamide auxiliaries is in the Supplementary Materials, table S1, and fig. S8). The replacement of one methyl group in substrate **1a** with a *tert*-butyl carboxylate group introduced an α-chiral carbon (C-1 in substrates and products). Starting from optically pure (*S*_S(IV)_, *R*_C-1_)-substrate, the corresponding rearrangement product (*R*_C-1_, *R*_C-2_)-**4a** was obtained in 82% yield with a diastereomeric ratio (d.r.) exceeding 99:1. The stereoisomeric (*S*_S(IV)_, *S*_C-1_)-substrate afforded (*S*_C-1_, *R*_C-2_)-**4b** in 77% yield and a d.r. of more than 99:1. These results suggested that the absolute configuration of the new stereogenic carbon (C-2 in products) was exclusively determined by the chiral sulfinyl group, irrespective of the stereochemistry of the existing α-chiral carbon (C-1) in substrate **1**. It was supported by further experiments using the other two stereoisomers as substrates, where C-2 in products **4c** and **4d** adopted an *S* configuration due to the use of (*R*_S(IV)_)-*^t^*BS reagent. Moreover, the diastereoselectivity of C-2 was higher in all four cases (**4a** to **4d**) compared to **3a**.

To further validate the substituent effect of carbon C-1 and alkynes, a variety of substrates were studied under the standard rearrangement reaction conditions (Fig. 2B). Across all substrates investigated, no negative proximity effect of C-1 was observed. Excellent diastereoselectivity was achieved in the reactions involving aryl-substituted substrates (**5** to **7**). The absolute configuration of C-2 was tuned by changing the configuration of the Ellman sulfinamide precursor. Substrates with flexible alkyl chain and olefin group were well tolerated, giving secondary amines with more than 99:1 d.r. and good yields (**8a** and **8b**). Cyclic primary amine-derived sulfinamides also afforded the desired products with excellent stereoselectivity. The d.r. increases with the size of rings in the substrates (**9a**-**11b**). Further modification of substituents based on the core structure of **1a** did not result in decrease of stereoselectivity (**12a** and **12b**). The d.r. remained over 99:1 in most reactions and showed good compatibility with various ynamides (**13a**-**15b**). The silyl alkyne was also successfully converted to desired product with excellent diastereoselectivity (**16**). However, the removal of *tert*-butanesulfide group by acid workup resulted in the hydrolysis of the silyl motif.

Density functional theory calculations suggested that the (*E*)-enolonium intermediate from sulfinamide and ynamide was kinetically favored over the (*Z*)-isomer (ΔΔ*G*^‡^ = 3.61 kcal/mol, **TS-*E*** versus **TS-*Z***; Fig. 2C and fig. S3). The [2,3]-rearrangement of the (*E*)-enolonium intermediate resulted in the *N*-S*^t^*Bu adduct. In the corresponding five-membered ring transition state (**TS-*E*-R1**; Fig. 2, A and C), the *N*-alkyl motif was far away from the C-2 center, which explained the absence of proximity effect from C-1 in this reaction. The absolute configuration of stereogenic S(IV) in sulfinamide dictated the stereochemistry of the newly formed chiral carbon center at C-2.

**Fig. 3.**
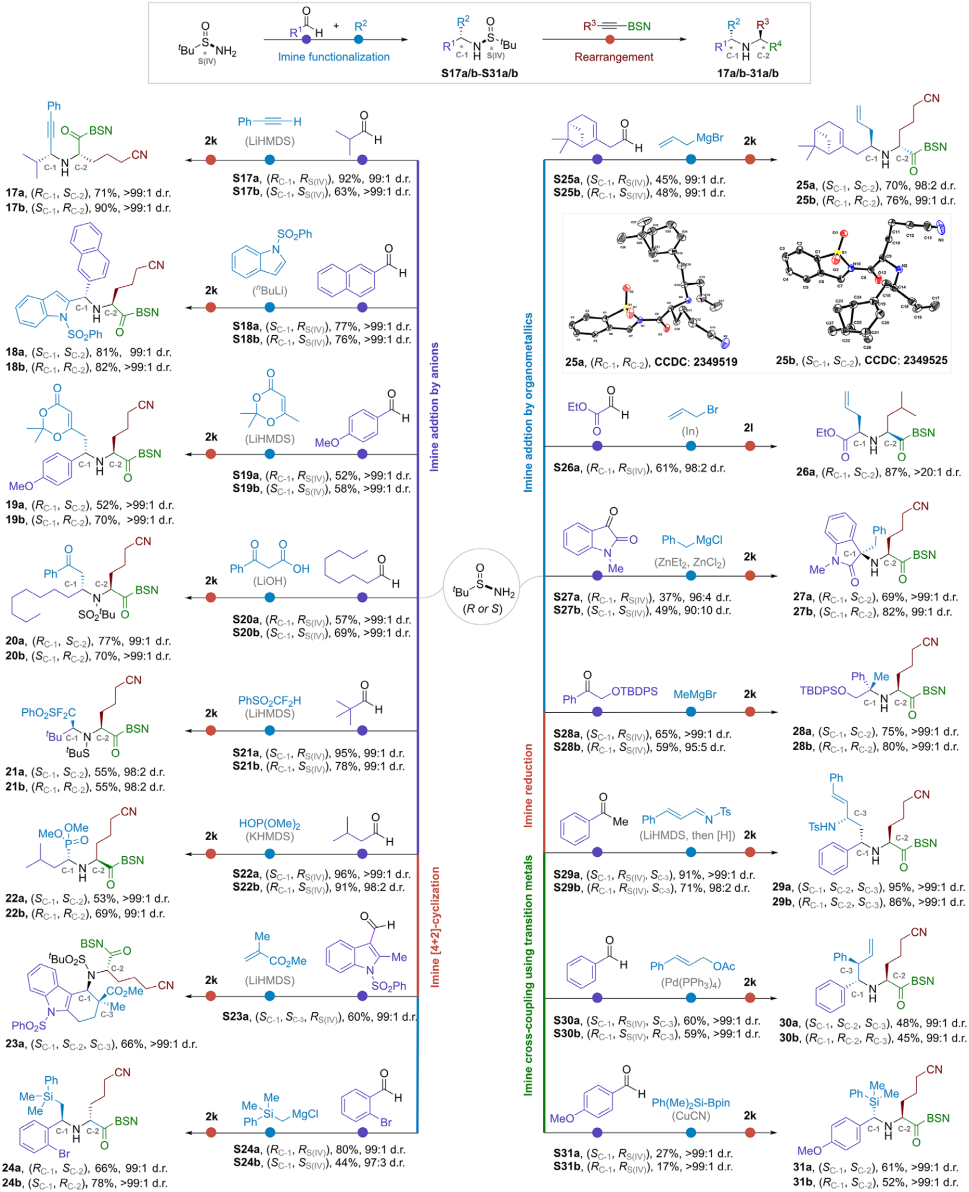
Stereodivergent synthesis of bis-α-chiral amines through consecutive *S*-to-*C* chirality induction and transfer strategy (grouped by the first chirality induction reactions involving imine intermediates). 2k: R^3^ = [CH_2_]_3_CN, 2l: R^3^ = CH_2_CH[CH_3_]_2_.

Among the conventional *^t^*BS-enabled asymmetric amine synthesis, imine functionalization was most extensively used. The process involved three typical steps: (i) imine formation through the condensation of *^t^*BS with aldehydes or ketones, (ii) diastereoselective imine functionalization, and (iii) removal of the sulfinyl group from the resulting products via N-S cleavage (*34*). The last step was less economic due to the wastage of one equivalent of optically pure sulfinyl moiety (*39*, *40*). In Fig. 3, we demonstrated that this conventional protocol could be repurposed for the preparation of highly branched bis-α-chiral secondary amines. Instead of removing the chiral sulfinyl group, enantiopure *^t^*BS-amine adducts obtained from imine functionalization in step 2 was subjected to another chirality transfer event using our [2,3]-rearrangement reaction. This three-step protocol included (i) imine preparation, (ii) diastereoselective imine functionalization, and (iii) diastereoselective [2,3]-rearrangement. Stereodivergent formation of two or more adjacent chiral carbons could be achieved using *^t^*BS reagent as the exclusive chiral sources, with *tert*-butanesulfide as the only by-product.

Nucleophilic addition of carbon or heteroatom fragments to aldimines was a straightforward route to branched amines. This reaction proceeded through an intramolecular transfer of organic residues from intermediate species, with metal cations serving as a coordination site associated with the sulfinyl oxygen or nitrogen. It was known as the closed transition state (CTS). Alternatively, there were a few examples of the Cram-type open transition state (OTS) mechanism, a nonchelation model that offered the opposite diastereoselectivity (*33*). The imine adduct of isobutyl aldehyde and (*R*)-*^t^*BS underwent a smooth reaction with lithium acetylene at −78°C, affording the addition product in 92% yield and 99:1 d.r. [*R*_C-1_, *R*_S(IV)_; Fig. 3] (*41*). This product was then subjected to a [2,3]-rearrangement reaction with alkyne **2k** that gave an unsymmetric propargyl amine **17a** in 71% yield and more than 99:1 d.r. (*R*_C-1_, *S*_C-2_). The other enantiomer **17b** was obtained through the same protocol but using (*S*)-*^t^*BS as the starting material.

Indole was an important unit in natural products and pharmaceuticals. The metalation of *N-*phenylsulfonyl indole and its subsequent addition to *^t^*BS-aldimine could be achieved with excellent yield and diastereoselectivity. It proceeded through an OTS mechanism that gave an (*S*)-C-1 from (*R*)-*^t^*BS (*42*). An (*S*)-C-2 was later derived from the corresponding adduct via rearrangement reaction (**18a**). The opposite isomer **18b** was also synthesized in nearly optically pure form. Moving forward to the *sp^3^*-hybridized carbon nucleophile for imine functionalization, dioxinone was a useful module for versatile posttransformations. Zhang *et al.* (*43*) reported that BF_3_•Et_2_O could promote high stereoselective reaction between *^t^*BS-imines and dioxinone lithium dienolate. The yields were moderate, but the d.r. remained excellent. Their secondary amine derivatives **19a** and **19b** were readily accessible through our protocol. The base-promoted decarboxylative reaction of β-keto acids with *^t^*BS-imines was mild and efficient (*44*), affording β-amino ketone via a CTS mechanism. The corresponding amines **20a** and **20b** were then derived in good yield and excellent diastereoselectivity.

The α-difluoromethyl amines were bioisosteres of α-aminocarbinols. Stereoselective synthesis of these compounds was achieved by Hu and Li (*45*) using a difluoromethyl phenyl sulfone reagent. The reaction with *^t^*BS-imines was efficient, and the high diastereoselectivity could be explained by a Cram-type OTS model. The adducts were converted to amines **21a**/**21b** in excellent selectivity. We found that the *N*─S*^t^*Bu bond was difficult to cleave under acidic conditions (6 M HCl). The strong electron-withdrawing effect of the PhSO_2_CF_2_ group might be responsible for this issue and the low yields. Nucleophilic addition of dialkyl phosphites to *^t^*BS-imines provided optically pure α-aminophosphonic acids (*46*). Through further integration with rearrangement reaction, we were able to obtain either enantiomer of the corresponding secondary amines **22a** and **22b**. Apart from being used as an electrophile, *^t^*BS-ketimines were also known to act as nucleophiles for various transformations, where the configuration of the newly formed stereogenic center was induced by the chiral sulfinyl group. The Michael addition reaction of *^t^*BS-metalloenamine was reported by Ellman *et al.* (*47*, *48*). Further development of this chemistry led to Michael/Mannich domino cyclization (*49*). The two contiguous stereogenic centers could be formed with excellent stereocontrol (C-1 and C-3 in **23a**) using methyl acrylate as a receptor. The third α-amino chiral carbon (C-2) was subsequently established with our iterative assembly method.

The reaction of *^t^*BS-aldimines and ketimines with organometallic reagents provided a general entry to α-chiral amines, including the formation of tertiary carbon centers (**24** to **28**) (*50*, *51*). All the corresponding nucleophilic addition adducts were compatible with the subsequent rearrangement reaction, yielding secondary amines with high diastereoselectivity and excellent structural diversity. The absolute configurations of **25a** and **25b** were identified by x-ray diffraction analysis, which were consistent with mechanism proposal. It is noteworthy that indium powder could reduce the allylic bromide in situ (*52*). The resulting species underwent nucleophilic addition with imines to afford homoallylic *^t^*BS-amine with high diastereoselectivity during the preparation of **26a**. Sequential creation of multiple chiral carbon centers could even be achieved in the synthesis of 1,3-diamines. The first step involved a Mannich reaction of (*R*)-*^t^*BS-metalloenamine with *N*-cinnamylidene-*p*-toluenesulfonamide that afforded the 1,2-addtion adduct with an (*S*)-C-3, following Chen *et al*.’s protocol (*53*). Subsequent reduction of *^t^*BS-imine with catecholborane gave *syn-*1,3-diamines [*R*_C-1_, *R*_S(IV)_, *S*_C-3_]. Alternatively, the anti-isomer was obtained using LiBHEt_3_ as the reductant (*S*_C-1_, *R*_S(IV)_, *S*_C-3_). They were then converted to **29a** and **29b** using our method, with a new (*S*)-carbon (C-2) derived from the (*R*)-*^t^*BS group. Recent development of transition metal catalysis provided another robust tool for imine functionalization. For instance, palladium-catalyzed cinnamylation of *^t^*BS-aldimines allowed the installation of two contiguous chiral carbons via a CTS mechanism (*54*). The third chiral carbon was subsequently established with our protocol (**30a** and **30b**). Copper catalysis allowed for diastereoselective introduction of heteroatoms to imino center that was challenging to achieve via conventional ionic protocols. Using this strategy, α-silylamines **31a** and **31b** were obtained (*55*). The yields were moderate, but the diastereselectivity remained excellent.

**Fig. 4.**
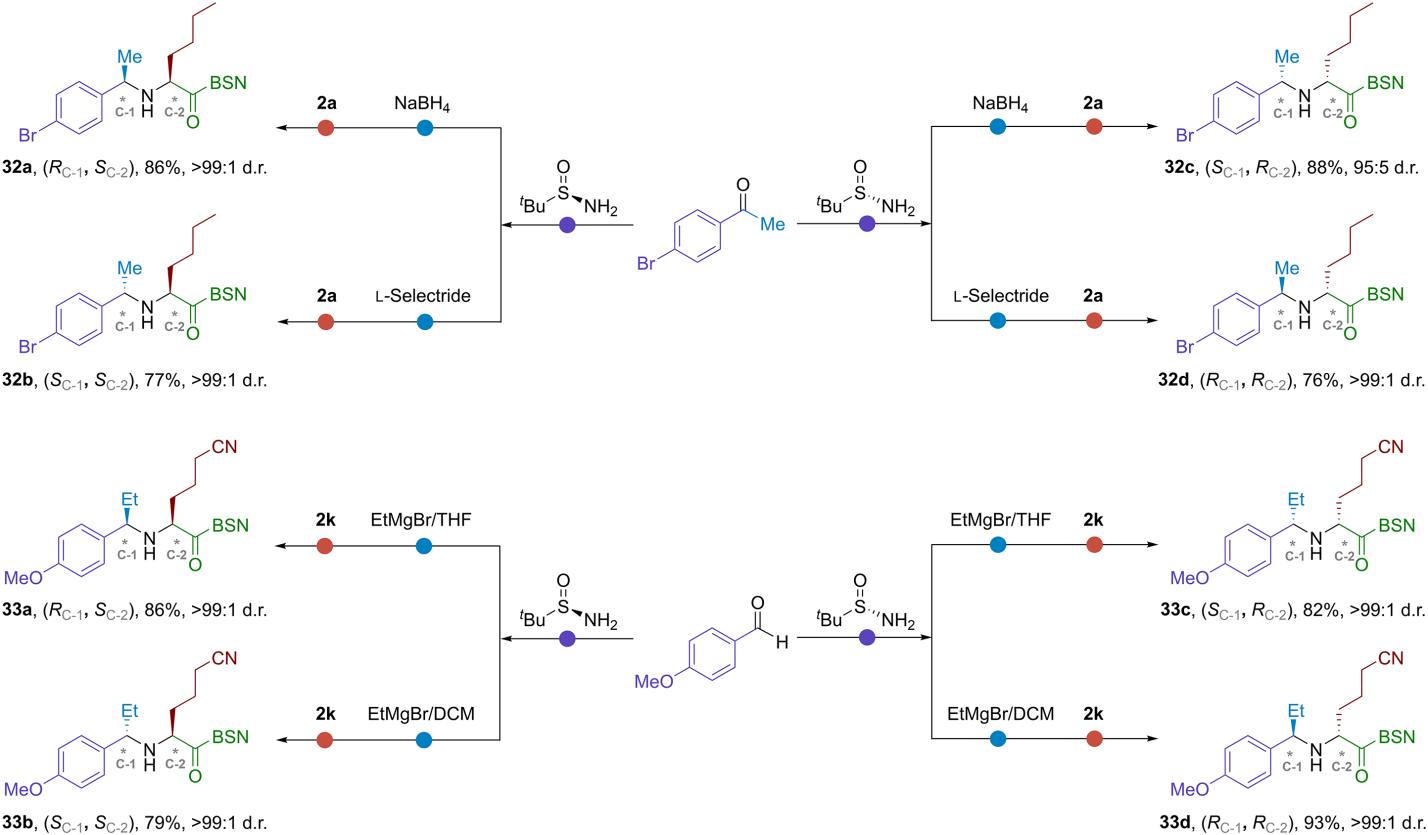
Modular synthesis of all four stereoisomers of unsymmetric bis-α-chiral secondary amines (2a: R^3^ = [CH_2_]_3_CH_3_; 2k: R^3^ = [CH_2_]_3_CN).

The stereoselectivity of *^t^*BS-imine functionalization was tunable by reagents, additives, solvents, and related intermediates. This tunability could be rationalized by either CTS or OTS mechanism and served as a useful strategy in the organic synthesis. By incorporating our [2,3]-rearrangement method, these approaches allowed for the facile preparation of all four stereoisomers of bis-α-chiral amines in a modular protocol. As a proof of concept, two representative examples were demonstrated in Fig. 4. The reduction of *^t^*BS-ketimine by NaBH_4_ or l-selectride (lithium tri-*sec*-butylborohydride) could afford two opposite enantiomers of carbon C-1 with high yield and stereoselectivity (e.g., **32a** and **32b**) (*56*). Notably, the ketimine preparation and diastereoselective reduction could be performed in one pot (*57*). Thereafter, the stereochemistry of carbon C-2 was induced by *tert*-butanesulfinamide through [2,3]-rearrangement reaction [*R*_S(IV)_ to *S*_C-2_]. Repeating these protocols with (*S*)-*tert*-butanesulfinamide gave the other two stereoisomers **32c** and **32d**. *^t^*BS-aldimines derived from the aldehydes and *tert*-butanesulfinamide could also be stereodivergently converted through reactions with various nucleophiles (*58*). An example was given for the synthesis of **33a**-**33d**, where the stereochemistry of C-1 was tuned by solvents.

**Fig. 5.**
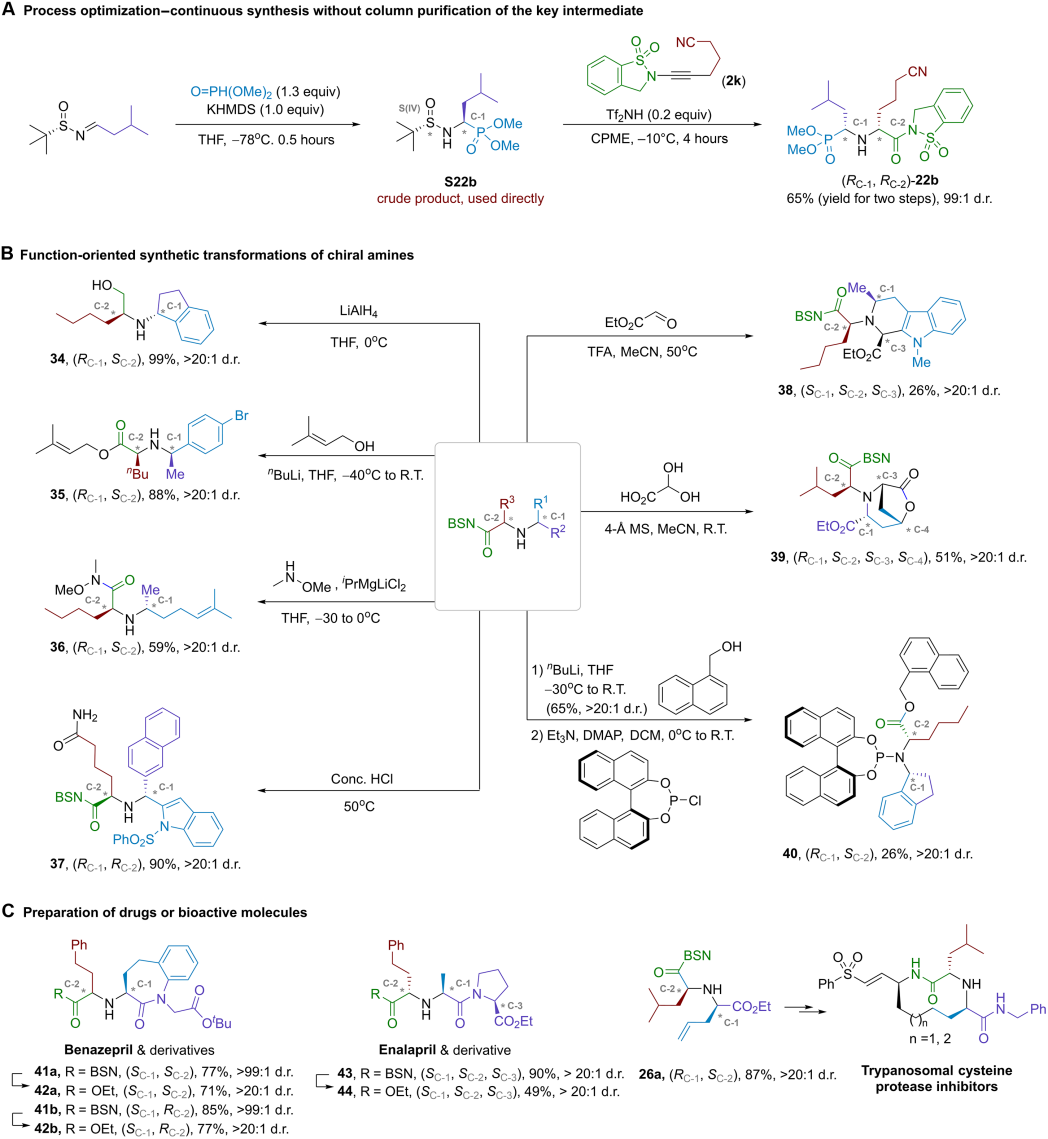
Demonstration of the synthetic utility of the consecutive *S*-to-*C* chirality induction/transfer protocol. **(A)** Green synthesis using optimized process. **(B)** Synthetic transformations. **(C)** Preparation of bioactive molecules. RT, room temperature; MS, molecular sieves; TFA, trifluoroacetic acid; THF, tetrahydrofuran; DCM, dichloromethane; DMAP, 4-dimethylaminopyridine.

Considering potential concerns about the practicality of this method, we demonstrated an example that this multistep process could be simplified to operate (Fig. 5A). The crude product from the first step of *^t^*BS-imine functionalization can be used directly without purification by column chromatography. This is because the reaction with ynamide in the second step did not require complex additives as promoting reagents, except for a substoichiometric amount of acid. Compound **22b** was obtained in 65% overall yield using the streamlined process, and the diastereoselectivity remained excellent. Integrating this process with continuous-flow synthesis is expected to further enhance synthetic efficiency and scalability. Further functional group-based skeleton modifications of the amino products obtained above provided desired functionalities (Fig. 5B). The BSN-amide group served as a versatile handle for chemical diversification and ligation. For example, it acted as an alcohol precursor that could be reduced by LiAlH_4_ (from **9b** to **34**). The optically pure amino alcohols were valuable modules in both organic synthesis and medicinal chemistry. Substitution of the BSN motif with other nucleophiles facilitated the connection of new functional fragments into the core structure, such as alcohol and amines (**35** from **32a** and **36** from **8a**). The hydrolysis of cyanide hydrolysis to amide (**37** from **18b**) exemplified that many other functional groups in the secondary amines also offered opportunity for late-stage diversification. Further incorporation of aliphatic substituent to the secondary amino center would allow the preparation of tris-α-chiral tertiary amines, which had the highest level of saturation and complexity in their neutral form. We demonstrated the preparation of polycyclicamines through the aza-Prins cyclization as a proof of concept. The condensation of ethyl glyoxylate with secondary amine yielded an iminium ion, which was intramolecularly captured by indole to form a piperidine ring (**38**) (*59*). The absolution configuration of C-3 was assigned on the basis of nuclear magnetic resonance spectroscopy. Alternatively, the iminium ion derived from amine and glyoxylic acid underwent intramolecular reaction with a terminal alkene. The resulting carbocation was trapped by the carboxyl group, yielding a lactone (**39** from **26a**). The two new stereogenic carbons were generated in optically pure form. Chiral secondary amines are key fragments in phosphoramidite ligands, which have been widely used in asymmetric synthesis (*60*). Condensation of chlorophosphite with chiral secondary amines enabled modular synthesis of these ligands, allowing for quick screening in the development of asymmetric reactions (**40**).

This short de novo protocol could be used for rational design and synthesis of chiral amine-containing complex scaffolds, as demonstrated in the preparation and modification of drugs and bioactive molecules (Fig. 5C). Benazepril was one of the most potent angiotensin-converting enzyme (ACE) inhibitors on the market. It was previously prepared through nucleophilic substitution of the benzolactam substrate (C-1 fragment in Fig. 2B, **42a**) by l-homophenylalanine ethyl ester (LHPE; C-2 fragment) (*61*). LHPE was an unnatural amino acid derivative that required additional steps for asymmetric synthesis. Our method bypassed the reported protocol and provided both diastereoisomers in good yields and excellent selectivity (**42a** and **42b**). The corresponding intermediates (**41a** and **41b**) were useful for drug conjugation or activity-based structural modification. A similar strategy could be applied to the preparation of another ACE inhibitor, enalapril (**43** and **44**). Compound (*R, S*)-**26a** was a key intermediate for the total synthesis of macrocyclic trypanosomal cysteine protease inhibitor (*62*). It was obtainable through our iterative synthetic method.

## DISCUSSION

The strategic balance of steps, materials, and labor is crucial in the synthesis of complex molecules. This de novo synthetic protocol offers a practical solution for the preparation of bis-α-chiral amines. It takes only two to three steps without requiring precious catalysts, exquisite reaction conditions, or advanced expertise. Contiguous stereogenic carbon centers are afforded in enantiopure form using a single commercially available chiral source. All possible stereoisomers can be selectively produced by minor adjustment of the starting materials or reagents. Function-oriented structural diversity of desired scaffolds can be achieved using readily accessible coupling partners. In contrast to conventional methods that discard the optically pure sulfinyl auxiliary after one chirality induction event, the repurposed protocol makes full use of Ellman sulfinamide with minimal production of waste.

## MATERIALS AND METHODS

### Imine functionalization (synthesis of S17a as an example)

The LiHMDS [4.0 ml, 1.0 M in tetrahydrofuran (THF), 2.0 equiv] was added dropwise to the solution of ethynylbenzene (408 mg, 4.0 mmol, 2.0 equiv) in hexane (24 ml) at −78°C under N_2_ atmosphere. The resulting mixture was warmed to 0°C, stirred for 10 min, and then cooled back to −78°C. Sulfinimine (350 mg, 2.0 mmol, 1.0 equiv) dissolved in THF (20 ml) was subsequently added dropwise at −78°C. The mixture was stirred at the same temperature overnight and then quenched by saturated aqueous NH_4_Cl. After extraction with ethyl acetate, the combined organic layer was washed with brine and concentrated under vacuum. The crude product was purified by flash chromatography (eluent: petroleum ether/ethyl acetate = 10:1 to 6:1) to afford **S17a** as colorless viscous oil.

### [2,3]-Rearrangement

Under N_2_ atmosphere, the solution of *tert*-butanesulfinamide **1** (0.30 mmol, 1.5 equiv) and ynamide **2** (0.20 mmol, 1.0 equiv) in cyclopentyl methyl ether (CPME; 1.5 ml, 0.1 M) was cooled to −10°C, followed by the addition of HNTf_2_ (0.4 ml, 0.1 M stock solution in CPME, 0.20 equiv). The resulting mixture was stirred at the same temperature for 4 hours and then treated with 6 M HCl (0.20 ml, 6.0 equiv) at room temperature for 2 to 3 hours, unless otherwise noted. The resulting reaction mixture was quenched by saturated aqueous NaHCO_3_ and extracted with ethyl acetate (2 × 20.0 ml). The combined organic layer was washed with brine and concentrated under reduced pressure. The crude mixture was purified by flash chromatography on silica gel to afford the desired product.

## Supplementary Materials

### This PDF file includes:

Supplementary Text Figs. S1 to S12 Table S1 References
