# Supplementary material for: Modular synthesis of bis-α-chiral amines using Ellman sulfinamide for consecutive S-to-C chirality induction/transfer
Source: Sci Adv. 2025 Apr 4;11(14):eadv2010. doi: 10.1126/sciadv.adv2010 (PMC11970453; doi:10.1126/sciadv.adv2010)
Supplement: Supplementary file 1 — Supplementary Text Figs. S1 to S12 Table S1 References [file sciadv.adv2010_sm.pdf]

Science Advances
AAAS

Supplementary Materials for

Modular synthesis of bis-α-chiral amines using Ellman sulfinamide for consecutive *S*-to-*C* chirality induction/transfer

Guangwu Sun *et al.*

Corresponding author: Bing Gao, gaobing@hnu.edu.cn

Sci. Adv. **11**, eadv2010 (2025)
DOI: 10.1126/sciadv.adv2010

This PDF file includes:

Supplementary Text
Figs. S1 to S12
Table S1
References

1. General Information

Unless otherwise noted, all reagents, catalysts, and solvents were obtained from commercial suppliers and used without further purification. And all *tert*-butyl sulfinimines were synthesized according to the literature procedure unless otherwise noticed (*63*). Reactions were monitored by thin-layer chromatography (TLC). Chromatograms were visualized by fluorescence quenching with UV light at 254 nm or by staining using phosphomolybdic acid, $KMnO_4$ or iodine. Products were purified by flash column chromatography on 200-300 mesh silica gel or separated by preparative thin layer chromatography (PTLC).

NMR spectra were recorded on Bruker AVANCE Ⅲ HD or AVANCE Neo 400 MHz spectrometer instruments for ^{1}H, ^{13}C, ^{19}F and ^{31}P acquisitions. All NMR spectra were recorded at 25°C unless otherwise stated. Chemical shifts (δ) are reported in parts per million (ppm) and referenced $CDCl_3$ (^{1}H: 7.26 ppm; ^{13}C: 77.16 ppm). Coupling constants, *J*, are reported in Hertz (Hz), and multiplicities are indicated as follows: s = singlet, d = doublet, t = triplet, m = multiplet, q = quartet, dd = doublet of doublets, dt = doublet of triplets, td = triplet of doublets, qt = quartet of triplets, tq = triplet of quartets, br = broad. Integration is provided and assignments are indicated. Structural assignments were made with additional information from COSY, NOESY, and HMBC experiments.

High resolution mass spectra (HRMS) were measured on a Waters Xevo G2-XS qtof equipped with ESI ion source.

High-Performance Liquid Chromatography (HPLC) analysis of chiral compounds was recorded on a SHIMADZU LC-20AT equipped with a variable wavelength UV-Vis detector SPD-20A and Daicel Chiralpak chiral column.

Optical rotations were measured in corresponding solvent on Anton Paar MCP-100 apparatus with a LED of wavelength 589 nm, and reported as follows: $[\alpha]_D^T$ (c = g/100 mL, solvent).

X-ray crystallography was performed on a Bruker D8 Quest instrument.

2. Summary of the Synthetic Applications of Ellman Sulfinamide.

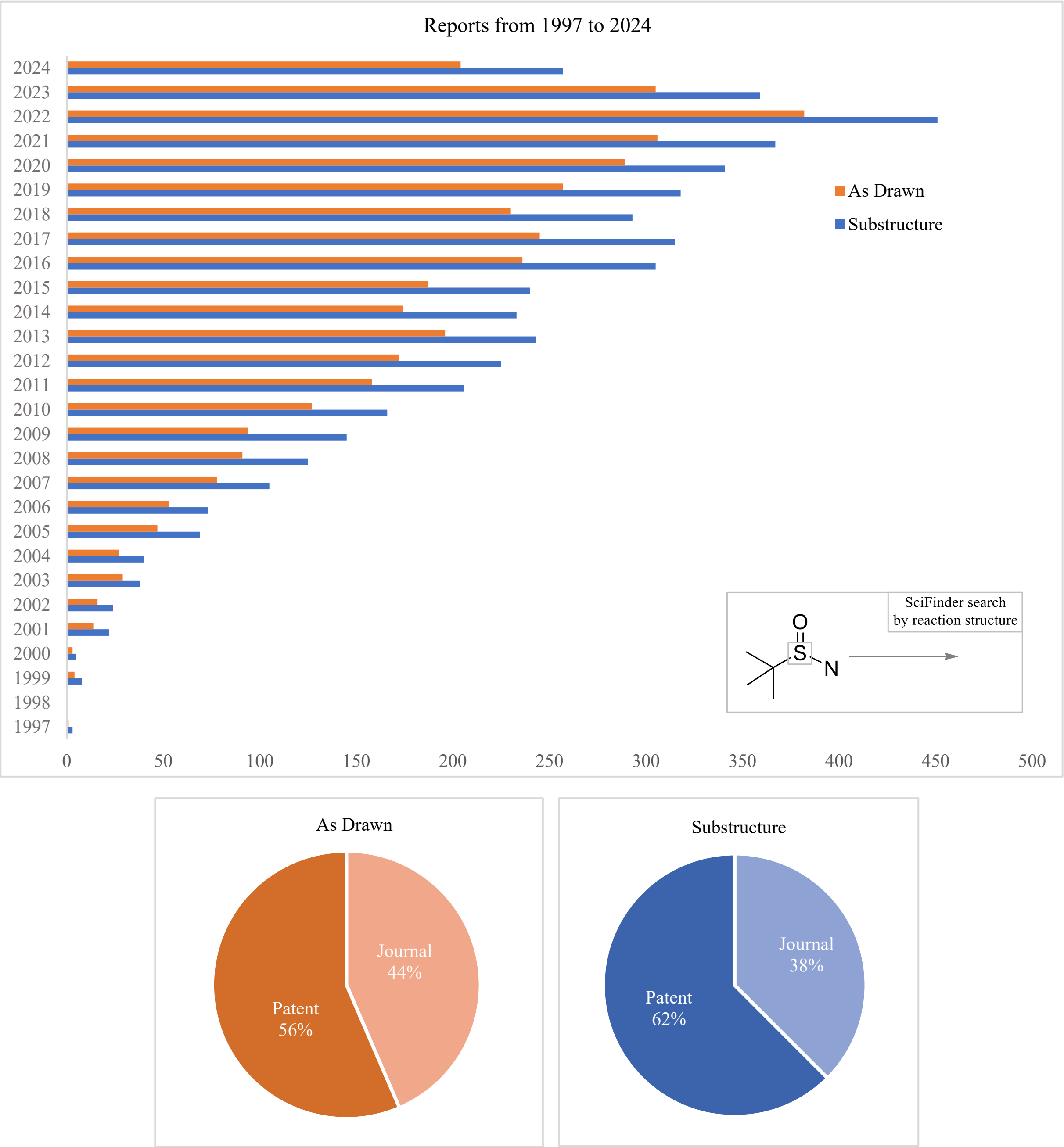

Figure S1. Reports using *tert*-butanesulfinamide and its derivatives as a reactant (based on a SciFinder search of reaction structure from 1997 to 2024).

Search settings: (reaction structure: *tert*-butanesulfinamide/ **lock atom**: S/ **variable parameter**: *N*-substituents)
Search results: (Structure match of "**As Drawn**": 5.0k refs / Structure match of "**Substructure**": 5.7k refs)
Search notes: 1, the lock of S atom in the search settings could exclude sulfonamides (^{t}BuSO$_2$N) as reactants; 2, no refinement on the N atom allows for potential sulfinamide derivatives as reactants, such as the ^{t}BuS imines; 3, the search results have not been individually validated and are for general reference only. There may be few reports that are less relevant, such as intermediates that are generated in situ or reactions that do not involve *tert*-butanesulfinamide auxiliary for asymmetric synthesis, particularly in the search results under the "**Substructure**" group.

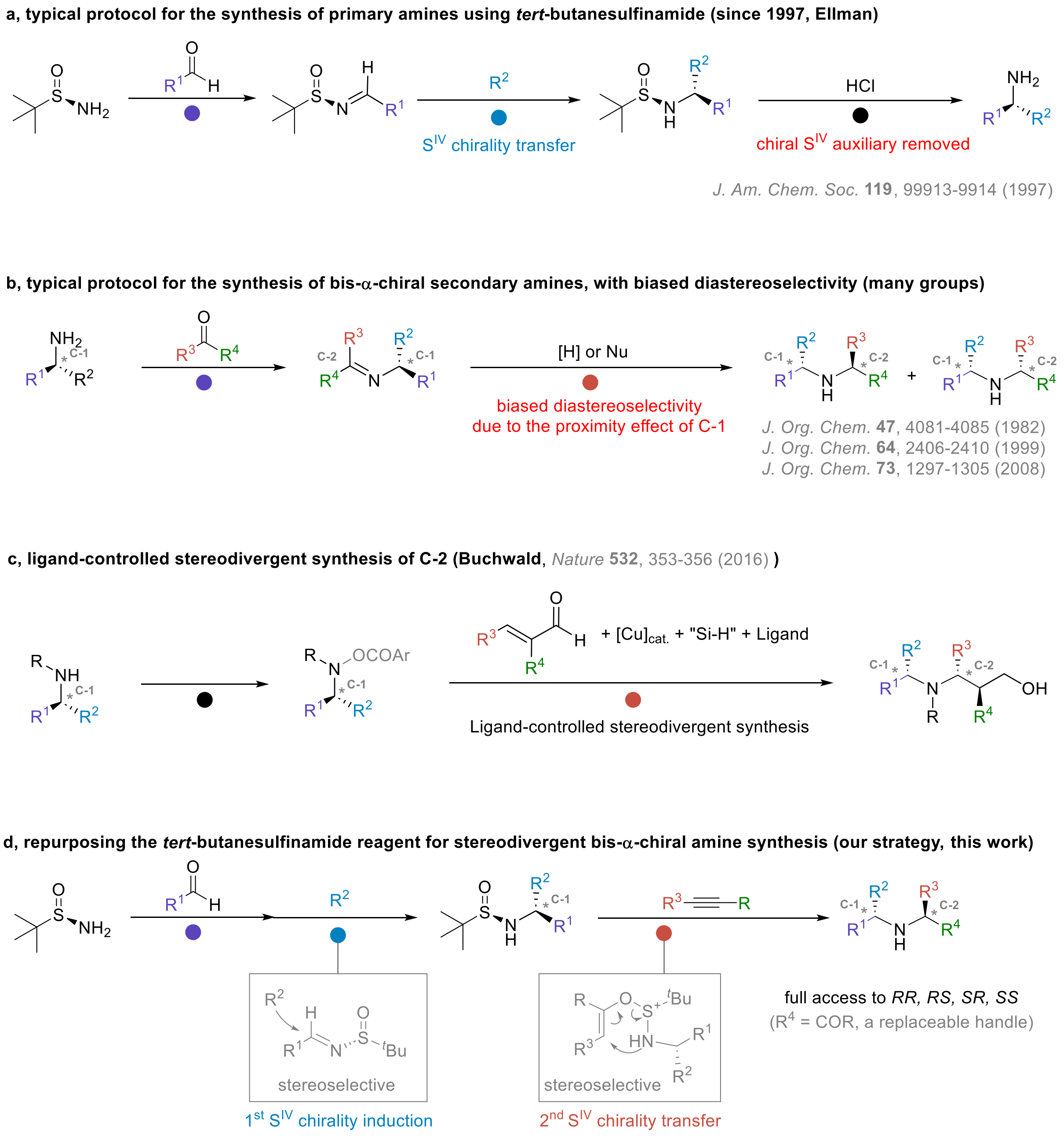

Figure S2. Representative protocol for amine synthesis and our strategy.

3. DFT Calculations

Computational Methods

DFT calculations were carried out with ORCA 5.0.4 program (*WIREs Comput. Mol. Sci.* **8**, e1327 (2018)), in the solution phase with the CPCM implicit solvation model (*J. Phys. Chem. A.* **103**, 11060-11079 (1999)) (static dielectric constant and refractive index were manually set as 4.76 and 1.42 respectively). The M06-2X functional (*Theor. Chem. Acc.* **120**, 215-241 (2008)) augmented with Grimme's D3 dispersion correction (*J. Chem. Phys.* **132**, 154104 (2010)) and the The 6-31G(d,p) basis set (*J. Chem. Phys.* **54**, 724-728 (1971); *J. Chem. Phys.* **56**, 2257-2261 (1972); *Theor. Chim. Acta* **28**, 213-222 (1973); *J. Am. Chem. Soc.* **104**, 2797-2803 (1982); *J. Chem. Phys.* **77**, 3654-3665 (1982)) set was used for geometry optimizations. All optimized geometries were confirmed by the frequency analysis while transition states were further confirmed by the intrinsic reaction coordinate (IRC) calculation. Thermal corrections at 263.15 K were calculated in the Shermo 2.5 program (*Comput. Theor. Chem*. **1200**, 113249 (2021)) with Grimme's quasi- rigid-rotor harmonic oscillator model from harmonic vibrational frequency. The wB97M-V functional (*J. Chem. Phys.* **142**, 074111 (2015)) with the aug-cc-pVTZ basis set (*J. Chem. Phys.* **90**, 1007-1023 (1989); *J. Chem. Phys.* **96**, 6796-6806 (1992); *J. Chem. Phys.* **98**, 1358-1371 (1993)) was used to calculate the single-point energies and give more accurate energy information. The solvent effects were considered by single-point calculations as same as in geometry optimizations. Solvation free energies were corrected to concentration of 1 $mol{\cdot}L^{-1}$ by adding +1.89 $kcal{\cdot}mol^{-1}$ to all species. Interaction region indicator (IRI) analysis (*Chem.: Methods* **1**, 231-239 (2021)) of wavefunction from single point energy calculation was performed using the Multiwfn 3.8 (dev) program (*J. Comput. Chem.* **33**, 580-592 (2012)). Structures were visualized in VMD program (*J. Mol. Graph.* **14**, 33-38 (1996)).

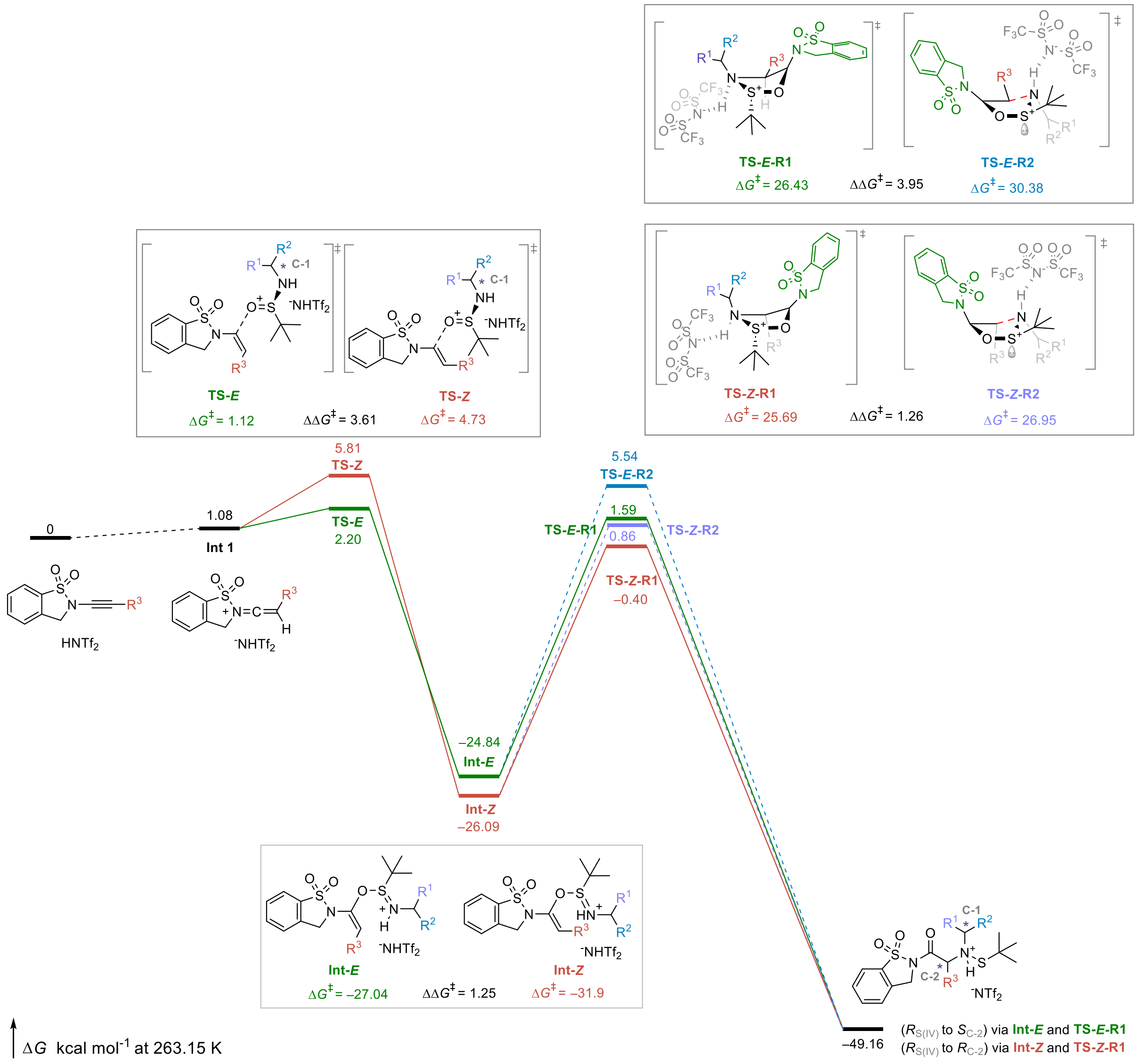

Figure S3. DFT-computed relative energy profile of the [2,3]-rearrangement reaction.

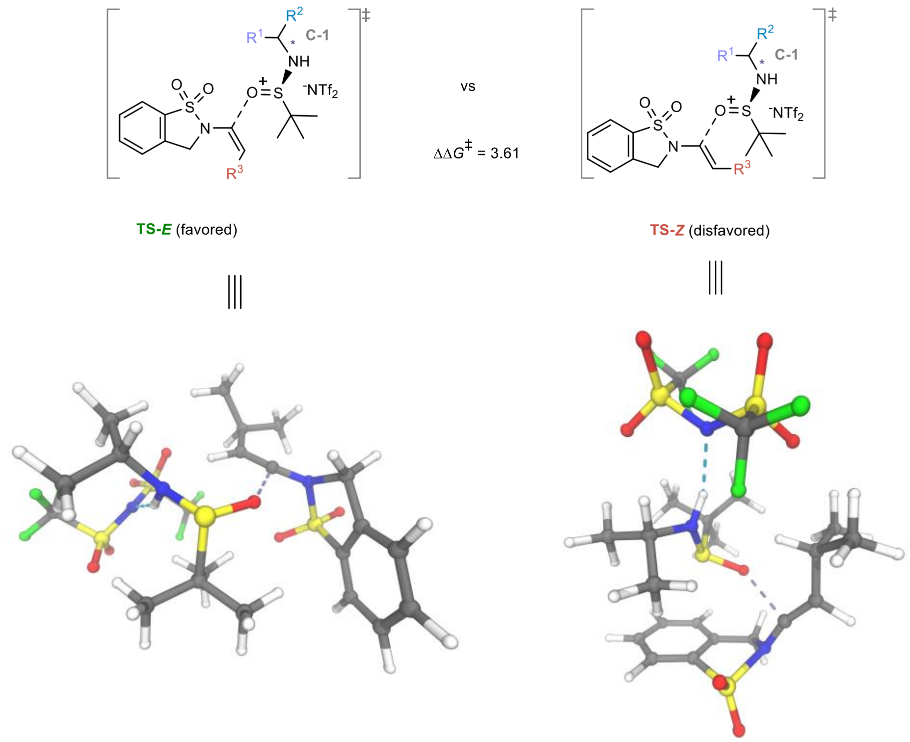

Figure S4. *Z*/*E* selectivity of the enolonium intermediates.

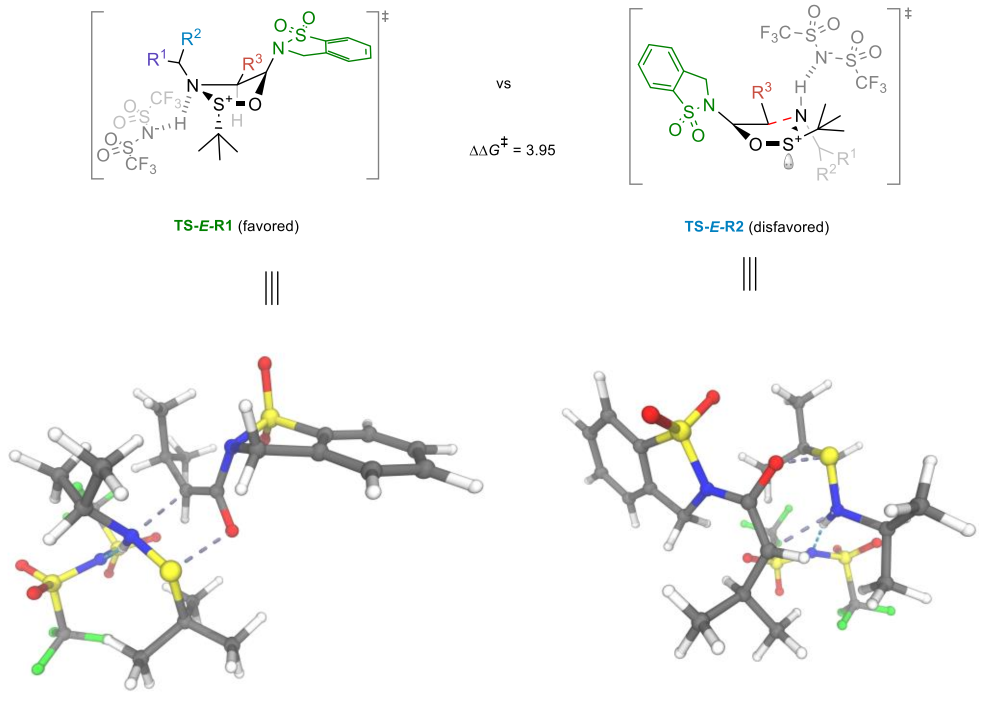

Figure S5. [2,3]-rearrangement from the *E* intermediate.

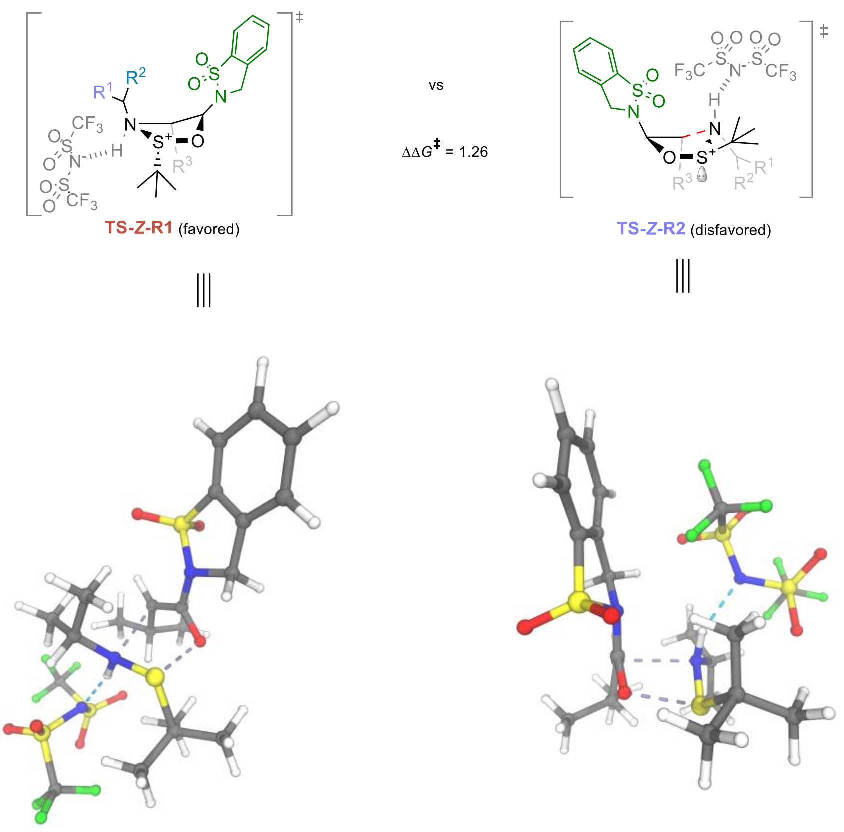

Figure S6. [2,3]-rearrangement from the *Z* intermediate.

4. Preparation and Characterization of Ynamides

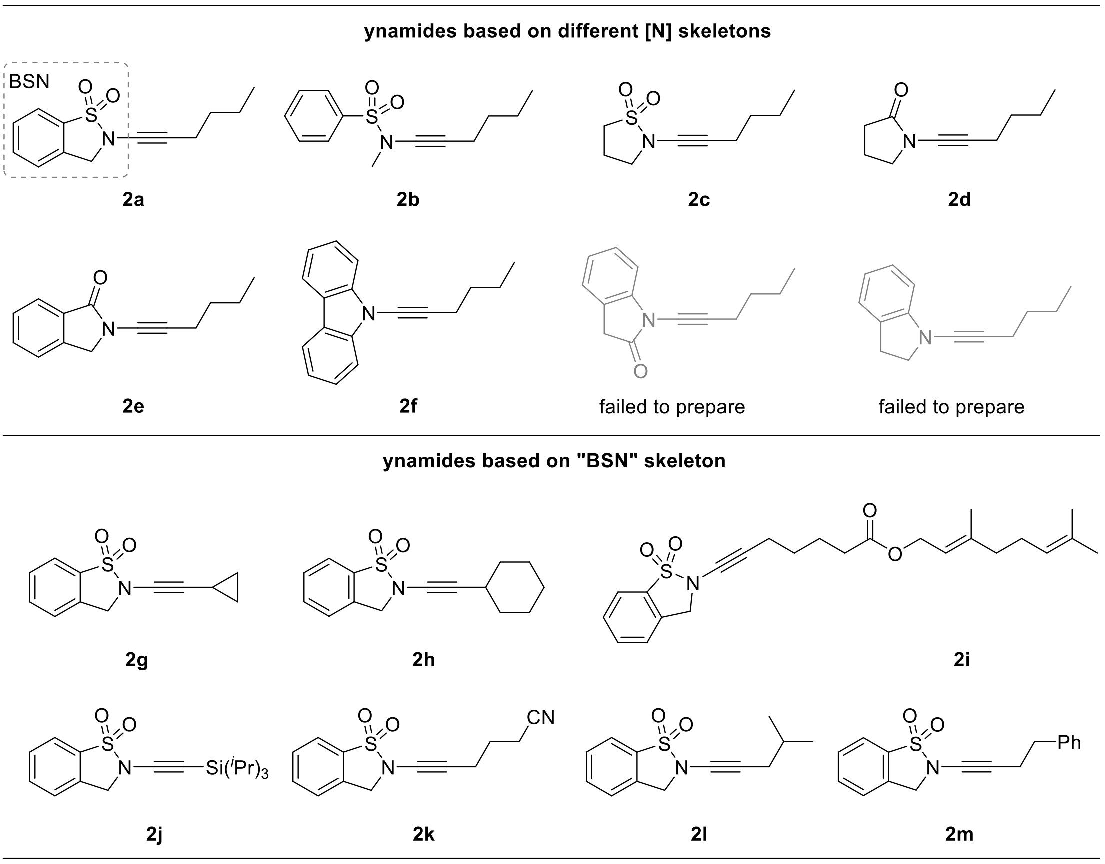

Figure S7. Ynamides involved in the manuscript (prepared via the reported procedure (*36*, *64 and 65*).

Typical procedure A for the preparation of ynamide

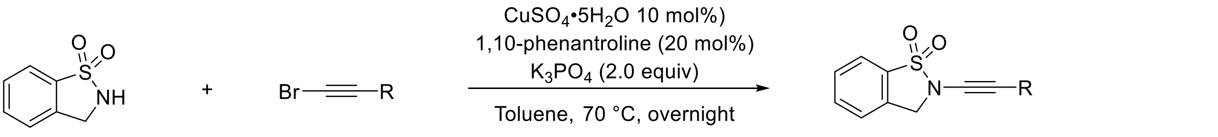

(1.0 equiv) (2.0 equiv) **2**

Take the synthesis of **2m** as an example: Under N_2 atmosphere, to a 50 mL glass tube were added 2,3-dihydro-1,2-benzisothiazole 1,1-dioxide (567 mg, 3.5 mmol, 1.0 equiv), K_3PO_4 (1.45 g, 7.0 mmol, 2.0 equiv), $CuSO_4 \cdot 5H_2O$ (87.5 mg, 0.35 mmol, 10 mol%), 1,10-phenanthroline (126 mg, 0.70 mmol, 20 mol%), (4-bromobut-3-yn-1-yl)benzene (1.08 g, ~5.2 mmol, 1.5 equiv), and degassed toluene (14 mL). The reaction mixture was stirred at 70 °C overnight and then cooled to room temperature. The resulting mixture was filtered over a plug of celite (washed with ethyl acetate), concentrated under reduced pressure and then purified by flash column chromatography (eluent: petroleum ether/ethyl acetate = 8:1 to 6:1) to afforded **2m** as yellow solid (822 mg, 79% yield).

2-(Hex-1-yn-1-yl)-2,3-dihydrobenzoisothiazole 1,1-dioxide (2a)

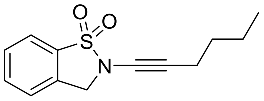

The title compound was prepared according to procedure A and all data are consistent with the literature reports (*36*).

^{1}H NMR (400 MHz, $CDCl_3$) δ 7.82 (d, *J* = 7.8 Hz, 1H), 7.65 (t, *J* = 7.4 Hz, 1H), 7.56 (t, *J* = 7.6 Hz, 1H), 7.39 (d, *J* = 7.7 Hz, 1H), 4.72 (s, 2H), 2.36 (t, *J* = 7.0 Hz, 2H), 1.59 – 1.50 (m, 2H), 1.49 – 1.38 (m, 2H), 0.92 (t, *J* = 7.3 Hz, 3H).

***N*-(hex-1-yn-1-yl)-*N*-methylbenzenesulfonamide (2b)**

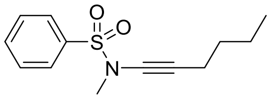

The title compound was prepared according to procedure A and all data are consistent with the literature reports (*36*).

^{1}H NMR (400 MHz, $CDCl_3$) δ 7.90 (d, *J* = 7.8 Hz, 2H), 7.66 (t, *J* = 7.4 Hz, 1H), 7.56 (t, *J* = 7.6 Hz, 2H), 3.03 (s, 3H), 2.24 (t, *J* = 6.9 Hz, 2H), 1.49 – 1.41 (m, 2H), 4.02 – 1.33 (m, 2H), 0.89 (t, *J* = 7.2 Hz, 3H).

2-(Hex-1-yn-1-yl)isothiazolidine 1,1-dioxide (2c)

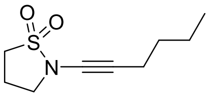

The title compound was prepared according to procedure A and all data are consistent with the literature reports (*36*).

^{1}H NMR (400 MHz, $CDCl_3$) δ 3.57 (t, *J* = 6.8 Hz, 2H), 3.15 (t, *J* = 7.5 Hz, 2H), 2.41 – 2.31 (m, 2H), 2.22 (t, *J* = 7.0 Hz, 2H), 1.46 – 1.38 (m, 2H), 1.37 – 1.30 (m, 2H), 0.83 (t, *J* = 7.2 Hz, 3H).

1-(Hex-1-yn-1-yl)pyrrolidin-2-one (2d)

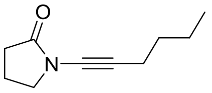

The title compound was prepared according to procedure A and all data are consistent with the literature reports (*36*).

^{1}H NMR (400 MHz, $CDCl_3$) δ 3.61 – 3.54 (m, 2H), 2.37 – 2.30 (m, 2H), 2.29 – 2.22 (m, 2H), 2.08 – 1.99 (m, 2H), 1.42 (t, *J* = 11.0 Hz, 2H), 1.37 – 1.29 (m, 2H), 0.86 – 0.80 (m, 3H).

2-(Hex-1-yn-1-yl)isoindolin-1-one (2e)

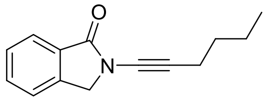

The title compound was prepared according to procedure A. Red brown solid (78 mg, 7%).

^{1}H NMR (400 MHz, $CDCl_3$) δ 7.83 (d, *J* = 7.6 Hz, 1H), 7.55 (t, *J* = 7.5 Hz, 1H), 7.44 (t, *J* = 7.5 Hz, 1H), 7.39 (d, *J* = 7.7 Hz, 1H), 4.59 (s, 2H), 2.37 (t, *J* = 7.1 Hz, 2H), 1.54 (p, *J* = 7.0 Hz, 2H), 1.43 (p, *J* = 7.2 Hz, 2H), 0.90 (t, *J* = 7.3 Hz, 3H).

^{13}C NMR (101 MHz, $CDCl_3$) δ 169.01, 140.86, 132.71, 130.15, 128.53, 124.35, 122.87, 73.70, 71.44, 52.74, 31.04, 22.03, 18.47, 13.68.

HRMS (ESI, m/z): $[M+Na]^+$ Calcd. For $C_{14}H_{15}NONa$: 236.1051; Found: 236.1054.

9-(Hex-1-yn-1-yl)-9*H*-carbazole (2f)

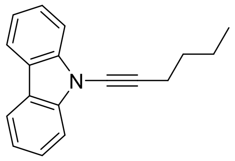

The title compound was prepared according to procedure A and all data are consistent with the literature reports (*64*).

^{1}H NMR (400 MHz, $CDCl_3$) δ 8.05 (d, *J* = 7.7 Hz, 2H), 7.67 (d, *J* = 8.1 Hz, 2H), 7.54 (t, *J* = 7.6 Hz, 2H), 7.34 (t, *J* = 7.5 Hz, 2H), 2.63 (t, *J* = 7.0 Hz, 2H), 1.74 (dd, *J* = 9.0, 6.1 Hz, 2H), 1.68 – 1.56 (m, 2H), 1.05 (t, *J* = 7.3 Hz, 3H).

2-(Cyclopropylethynyl)-2,3-dihydrobenzoisothiazole 1,1-dioxide (2g)

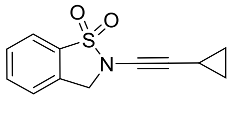

The title compound was prepared according to procedure A and all data are consistent with the literature reports (*36*).

^{1}H NMR (400 MHz, $CDCl_3$) δ 7.80 (d, *J* = 7.8 Hz, 1H), 7.65 (t, *J* = 7.5 Hz, 1H), 7.56 (t, *J* = 7.6 Hz, 1H), 7.39 (d, *J* = 7.7 Hz, 1H), 4.69 (s, 2H), 1.42 – 1.36 (m, 1H), 0.86 – 0.80 (m, 2H), 0.77 – 0.76 (m, 2H).

2-(Cyclohexylethynyl)-2,3-dihydrobenzoisothiazole 1,1-dioxide (2h)

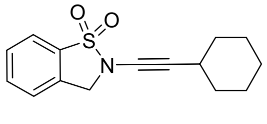

The title compound was prepared according to procedure A and all data are consistent with the literature reports (*36*).

^{1}H NMR (400 MHz, $CDCl_3$) δ 7.82 (d, *J* = 7.8 Hz, 1H), 7.65 (t, *J* = 7.4 Hz, 1H), 7.56 (t, *J* = 7.6 Hz, 1H), 7.39 (d, *J* = 7.7 Hz, 1H), 4.72 (s, 2H), 2.59 – 2.50 (m, 1H), 1.87 – 1.81 (m, 2H), 1.73 – 1.71 (m, 2H), 1.54 – 1.44 (m, 3H), 1.38 – 1.28 (m, 3H).

(*E*)-3,7-dimethylocta-2,6-dien-1-yl-7-(1,1-dioxidobenzoisothiazol-2(3*H*)-yl)hept-6-ynoate(2i)

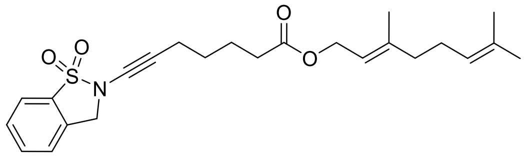

The title compound was prepared according to procedure A and all data are consistent with the literature reports (*36*).

^{1}H NMR (400 MHz, $CDCl_3$) δ 7.81 (d, *J* = 7.8 Hz, 1H), 7.64 (t, *J* = 7.5 Hz, 1H), 7.55 (t, *J* = 7.6 Hz, 1H), 7.39 (d, *J* = 7.7 Hz, 1H), 5.33 (t, *J* = 7.0 Hz, 1H), 5.07 (t, *J* = 6.4 Hz, 1H), 4.71 (s, 2H), 4.59 (d, *J* = 7.1 Hz, 2H), 2.38 (t, *J* = 7.1 Hz, 2H), 2.33 (t, *J* = 7.5 Hz, 2H), 2.09 (dd, *J* = 13.9, 7.0 Hz, 2H), 2.05 – 2.00 (m, 2H), 1.80 – 1.72 (m, 2H), 1.69 (s, 3H), 1.67 (s, 3H), 1.64 – 1.56 (m, 5H).

2-((Triisopropylsilyl)ethynyl)-2,3-dihydrobenzoisothiazole 1,1-dioxide (2j)

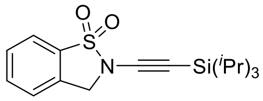

The title compound was prepared according to procedure A (*65*). White solid (3.08 g, 88%).

^{1}H NMR (400 MHz, $CDCl_3$) δ 7.78 (d, *J* = 7.8 Hz, 1H), 7.66 (t, *J* = 7.5 Hz, 1H), 7.56 (t, *J* = 7.6 Hz, 1H), 7.40 (d, *J* = 7.8 Hz, 1H), 4.77 (s, 2H), 1.09 (s, 21H).

^{13}C NMR (101 MHz, $CDCl_3$) δ 133.66, 133.01, 131.52, 129.71, 124.66, 121.77, 91.78, 72.11, 52.76, 18.66, 11.32.

HRMS (ESI, m/z): $[M+Na]^+$ Calcd. For $C_{17}H_{17}NO_2NaSSi$: 350.0647; Found: 350.0650.

6-(1,1-Dioxidobenzoisothiazol-2(3*H*)-yl)hex-5-ynenitrile (2k)

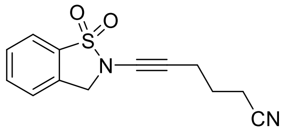

The title compound was prepared according to procedure A and all data are consistent with the literature reports (*36*).

^{1}H NMR (400 MHz, $CDCl_3$) δ 7.84 (d, *J* = 7.8 Hz, 1H), 7.68 (t, *J* = 7.6 Hz, 1H), 7.59 (t, *J* = 7.6 Hz, 1H), 7.41 (d, *J* = 7.7 Hz, 1H), 4.75 (s, 2H), 2.59 – 2.52 (m, 4H), 1.96 – 1.89 (m, 2H).

2-(4-Methylpent-1-yn-1-yl)-2,3-dihydrobenzoisothiazole 1,1-dioxide (2l)

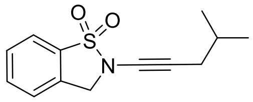

The title compound was prepared according to procedure A and all data are consistent with the literature reports (*36*). **^{1}H NMR** (400 MHz, $CDCl_3$) δ 7.69 (d, *J* = 7.8 Hz, 1H), 7.57 (t, *J* = 7.6 Hz, 1H), 7.48 (t, *J* = 7.6 Hz, 1H), 7.36 (d, *J* = 7.8 Hz, 1H), 4.64 (s, 2H), 2.16 (d, *J* = 6.6 Hz, 2H), 1.83 – 1.69 (m, 1H), 0.93 (d, *J* = 6.8 Hz, 6H).

2-(4-phenylbut-1-yn-1-yl)-2,3-dihydrobenzoisothiazole 1,1-dioxide (2m)

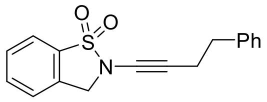

The title compound was prepared according to procedure A.

^{1}H NMR (400 MHz, $CDCl_3$) δ 7.82 (d, *J* = 7.7 Hz, 1H), 7.65 (t, *J* = 7.0 Hz, 1H), 7.56 (t, *J* = 7.2 Hz, 1H), 7.39 (d, *J* = 7.8 Hz, 1H), 7.35 – 7.28 (m, 2H), 7.28 – 7.19 (m, 3H), 4.67 (s, 2H), 2.89 (t, *J* = 7.6 Hz, 2H), 2.65 (t, *J* = 7.6 Hz, 2H).

^{13}C NMR (101 MHz, $CDCl_3$) δ 140.6, 133.5, 133.1, 131.1, 129.7, 128.6, 128.5, 126.4, 124.6, 121.9, 72.2, 69.3, 52.7, 35.3, 21.1.

HRMS (ESI, m/z): $[M+Na]^+$ Calcd. For $C_{17}H_{15}NO_2NaS$: 320.0721; Found: 320.0725.

5. The Auxiliary Effect of Different Ynamides.

Table S1. Auxiliary effect of ynamide substrates on the rearrangement reaction.

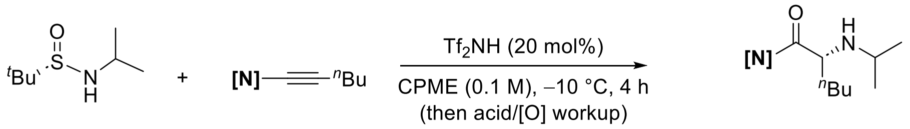

1, 0.30 mmol　**2**, 0.20 mmol　**3**

entry[a]	[N] =		yield (%)[d]	e.e. (%)[e]
1[a]	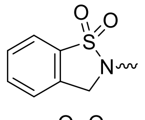	(**2a**)	63[f]	93
2[a]	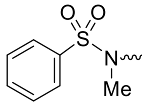	(**2b**)	80	88
3[b]	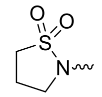	(**2c**)	65	87
4[b]	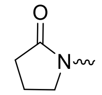	(**2d**)	75	77
5[c]	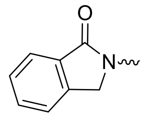	(**2e**)	71[f]	84
6[c]	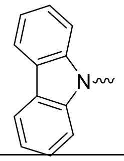	(**2f**)	99[f]	74

[a] Under N_2 atmosphere, the solution of ynamide **2** (0.20 mmol) and sulfonamide **1** (0.30 mmol) in CPME (2.0 mL, 0.1 M) was cooled to –10 °C, and then Tf_2NH (0.20 equiv, 0.4 mL, 0.1 M stock solution in CPME) was added dropwise. The resulting mixture was stirred at the same temperature for 4 hours, followed by workup with 6M HCl (0.2 mL, 6.0 equiv) for 2~3 hours.

[b] Using *m*-CPBA (173 mg, 1.0 mmol, 5.0 equiv) for workup (room temperature, overnight) instead of 6M HCl. The corresponding product has a *N*-$SO_2{}^t$Bu group.

[c] No acid work-up to afford product with protecting group (PG) = S^{t}Bu.

[d] Yields were determined by ^{1}H NMR analysis of the crude products using 0.10 mmol of benzyl benzoate as an internal standard.

[e] Enantiomeric excess (e.e.) was determined using chiral high-performance liquid chromatography (HPLC).

[f] Isolated yield.

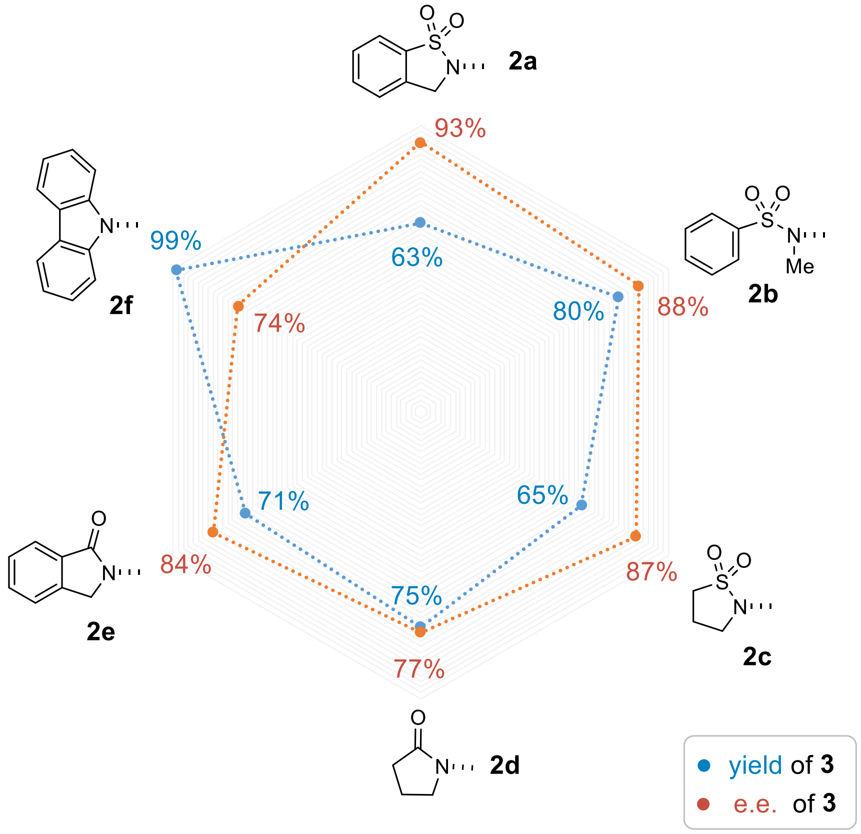

Figure S8. The auxiliary effect of different ynamides.

6. Synthesis and Characterization of bis-α-Chiral Amines.

Synthesis of secondary amines via [2,3]-rearrangement (GENERAL PROCEDURE C)

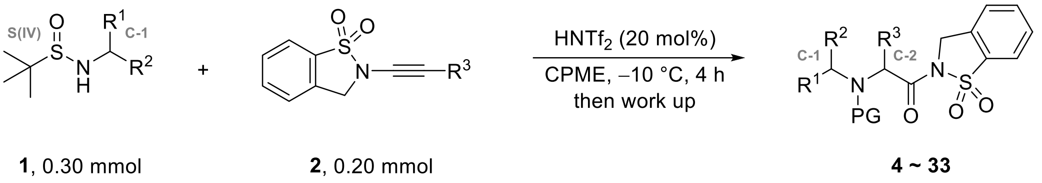

Under N_2 atmosphere, the solution of *tert*-butanesulfinamide **1** (0.30 mmol, 1.5 equiv) and ynamide **2** (0.20 mmol, 1.0 equiv) in cyclopentyl methyl ether (CPME, 1.5 mL, 0.1 M) was cooled to −10 °C, followed by the addition of $HNTf_2$ (0.4 mL, 0.1 M stock solution in CPME, 0.20 equiv). The resulted mixture was stirred at the same temperature for 4 h and then workup with 6M HCl (0.20 mL, 6.0 equiv) at room temperature for 2~3 hours, unless otherwise noticed. Thereafter, the reaction mixture was quenched by saturated aqueous $NaHCO_3$ and extracted with ethyl acetate (EA, 2 × 20.0 mL). The combined organic layer was washed with brine and concentrated under reduced pressure. The crude mixture was purified by flash chromatography on silica gel to afford products **4~33**.

Synthesis of ($R_{C\text{-}1}$, $R_{C\text{-}2}$)-4a

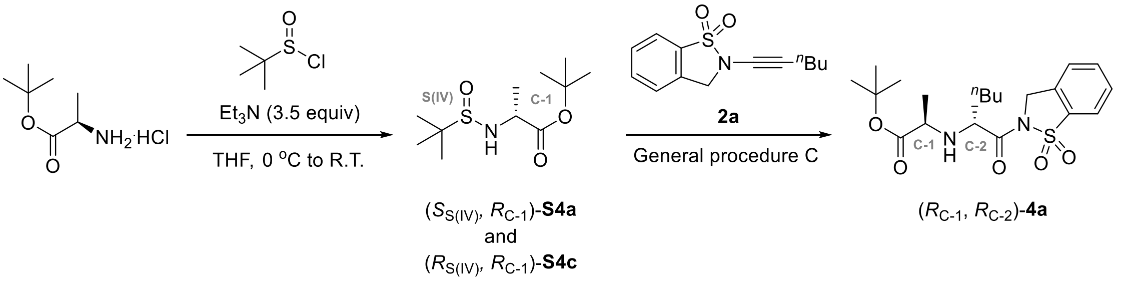

Synthesis of ($S_{S(IV)}$, $R_{C\text{-}1}$)**-S4a**

Under N_2 atmosphere, the mixture of *tert*-butyl-*D*-alaninate hydrochloride (1.81g, 10 mmol, 1.0 equiv) and Et_3N (3.54 g, 35 mmol, 3.5 equiv) in THF was cooled to 0 °C and then 2-methylpropane-2-sulfinic chloride (2.0 equiv) dissolved in THF was added slowly. The resulted mixture was stirred at room temperature overnight and then quenched with saturated aqueous NH_4Cl, extracted with ethyl acetate. The combined organic layer was washed with brine and concentrated under vacuum. The crude products were purified through flash chromatography (petroleum ether/ethyl acetate/dichloromethane = 6:1:1) to afford ($S_{S(IV)}$, $R_{C\text{-}1}$)-**S4a** as white solid (306 mg, 12% yield, >99:1 d.r.) and ($R_{S(IV)}$, $R_{C\text{-}1}$)-**S4c** as colorless oil (1.61 g, 64% yield, >99:1 d.r.). These two compounds were used for the preparation of **4a** and **4c** respectively.

^{1}H NMR (400 MHz, $CDCl_3$) δ 3.90 – 3.81 (m, 1H), 3.63 (d, J = 8.1 Hz, 1H), 1.43 – 1.35 (m, 12H), 1.15 (s, 9H).

^{13}C NMR (101 MHz, $CDCl_3$) δ 172.3, 81.7, 56.3, 54.2, 28.0, 22.5, 20.7.

HRMS (ESI, m/z): $[M+Na]^+$ Calcd. For $C_{11}H_{23}NO_3NaS$: 272.1296; Found: 272.1294.

HPLC (Chiralpak OD-H Column), *i*-PrOH/hexane = 3/97, flow rate = 1.0 mL/min, λ = 210 nm; t_R = 5.7 min (minor), t_R = 6.9 min (major).

[α]$_D^{20}$ = +50.4 (c = 1.0, $CHCl_3$).

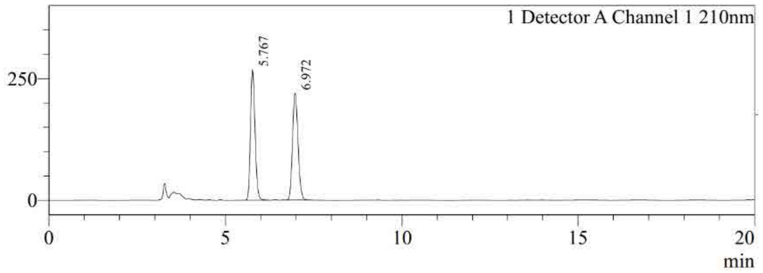

Detector A Channel 1 210nm

Peak#	Ret. Time	USP Width	Area	Height	Area%
1	5.767	0.246	2406130	267697	50.476
2	6.972	0.292	2360746	218700	49.524
Total			4766876	486397	100.000

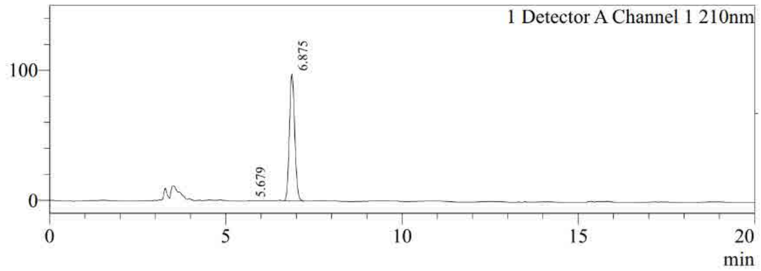

Detector A Channel 1 210nm

Peak#	Ret. Time	USP Width	Area	Height	Area%
1	5.679	0.033	43	-16	0.004
2	6.875	0.285	1024428	97368	99.996
Total			1024471	97352	100.000

***tert*-Butyl-((*R*)-1-(1,1-dioxidobenzoisothiazol-2(3*H*)-yl)-1-oxohexan-2-yl)-*D*-alaninate (4a)**

Prepared according to the **GENERAL PROCEDURE C** with ($S_{S(IV)}$, R_{C-1})-**S4a** (74.8 mg, 0.30 mmol, >99:1 d.r.) and **2a** (50.0 mg, 0.20 mmol) as substrates. Column chromatography: silica gel, petroleum ether/ethyl acetate/dichloromethane = 10:1:1. Colorless viscous liquid (67.7 mg, 82% yield, >99:1 d.r.).

^{1}H NMR (400 MHz, $CDCl_3$) δ 7.78 (d, *J* = 7.9 Hz, 1H), 7.68 (t, *J* = 7.6 Hz, 1H), 7.56 (t, *J* = 7.7 Hz, 1H), 7.45 (d, *J* = 7.8 Hz, 1H), 4.99 (d, *J* = 15.8 Hz, 1H), 4.86 (d, *J* = 15.9 Hz, 1H), 4.22 – 4.04 (m, 1H), 3.27 (q, *J* = 7.2 Hz, 1H), 2.18 (s, 1H), 1.90 – 1.78 (m, 1H), 1.68 – 1.47 (m, 3H), 1.44 (s, 9H), 1.38 – 1.28 (m, 2H), 1.26 (d, *J* = 6.4 Hz, 3H), 0.87 (t, *J* = 7.2 Hz, 3H).

^{13}C NMR (101 MHz, $CDCl_3$) δ 174.5, 174.2, 134.5, 134.1, 130.8, 129.7, 125.0, 121.9, 81.0, 60.3, 55.1, 47.4, 33.4, 28.1, 28.1, 22.6, 19.8, 13.9.

HRMS (ESI, m/z): $[M+H]^+$ Calcd. For $C_{20}H_{31}N_2O_5S$: 411.1954; Found: 411.1956.

HPLC (Chiralpak OD-H Column), *i*-PrOH/hexane = 2/98, flow rate = 1.0 mL/min, λ = 254 nm; t_R = 15.8 min (minor), t_R = 18.4 min (major).

[α]$_D^{25}$ = +17.6 (c = 0.5, $CHCl_3$).

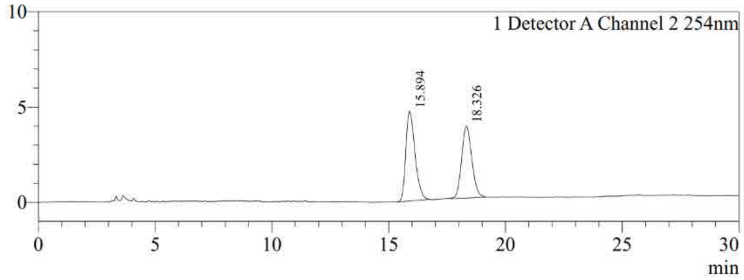

Detector A Channel 2 254nm

Peak#	Ret. Time	USP Width	Area	Height	Area%
1	15.894	0.720	129176	4703	52.870
2	18.326	0.800	115152	3778	47.130
Total			244327	8482	100.000

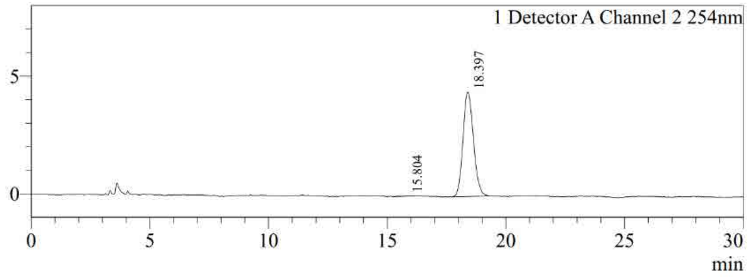

Detector A Channel 2 254nm

Peak#	Ret. Time	USP Width	Area	Height	Area%
1	15.804	0.257	315	12	0.231
2	18.397	0.814	136308	4437	99.769
Total			136623	4449	100.000

Synthesis of ($S_{C\text{-}1}$, $R_{C\text{-}2}$)-4b

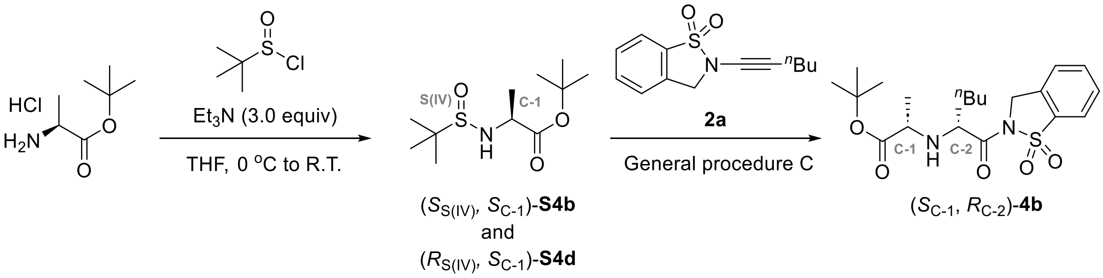

Synthesis of ($S_{S(IV)}$, $S_{C\text{-}1}$)-**S4b**

Under N_2 atmosphere, the mixture of *tert*-butyl-*L*-alaninate hydrochloride (545 mg, 3.0 mmol, 1.0 equiv) and Et_3N (910 mg, 9.0 mmol, 3.0 equiv) in THF was cooled at 0 °C and then 2-methylpropane-2-sulfinic chloride (2.0 equiv) dissolved in THF was added slowly. The resulted mixture was stirred at room temperature overnight and then quenched with saturated aqueous NH_4Cl, extracted with ethyl acetate. The combined organic layer was washed with brine and concentrated under vacuum. The crude products were purified through flash chromatography (eluent: petroleum ether/ethyl acetate/dichloromethane = 6:1:1) to afford ($S_{S(IV)}$, $S_{C\text{-}1}$)-**S4b** as colorless viscous oil (471 mg, 63% yield, >99:1 d.r.) and ($R_{S(IV)}$, $S_{C\text{-}1}$)-**S4d** as white solid (158 mg, 21% yield, >99:1 d.r.). These two compounds were used for the preparation of **4b** and **4d** respectively.

^{1}H NMR (400 MHz, $CDCl_3$) δ 4.08 (d, J = 5.1 Hz, 1H), 3.85 – 3.78 (m, 1H), 1.38 (s, 9H), 1.28 (d, J = 7.0 Hz, 3H), 1.14 (s, 9H).

^{13}C NMR (101 MHz, $CDCl_3$) δ 172.4, 82.0, 55.4, 52.8, 27.8, 22.4, 19.6.

HRMS (ESI, m/z): $[M+Na]^+$ Calcd. For $C_{11}H_{23}NO_3NaS$: 272.1296; Found: 272.1300.

HPLC (Chiralpak OD-H Column), *i*-PrOH/hexane = 5/95, flow rate = 1.0 mL/min, λ = 210 nm; t_R = 7.2 min (major), t_R = 8.9 min (minor).

$[\alpha]_D^{20}$ = +89.2 (c = 1.0, $CHCl_3$).

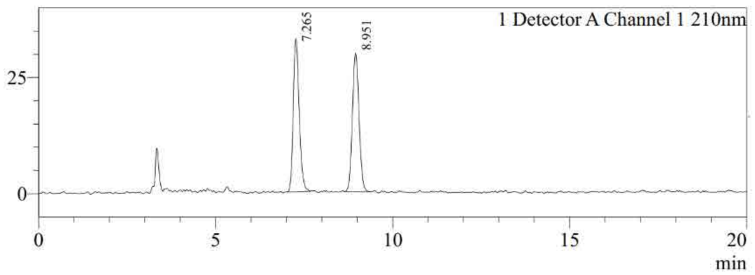

Detector A Channel 1 210nm

Peak#	Ret. Time	USP Width	Area	Height	Area%
1	7.265	0.310	382931	32895	49.650
2	8.951	0.350	388333	29709	50.350
Total			771264	62604	100.000

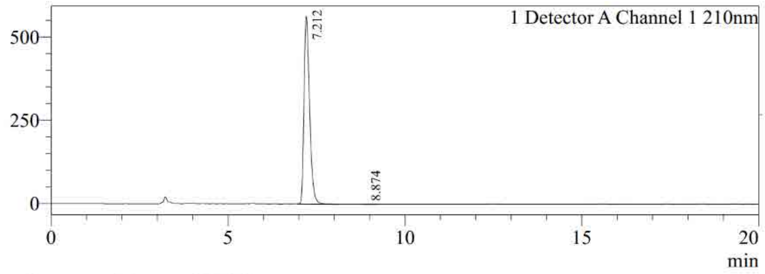

Detector A Channel 1 210nm

Peak#	Ret. Time	USP Width	Area	Height	Area%
1	7.212	0.292	6196955	562708	99.958
2	8.874	0.313	2614	177	0.042
Total			6199569	562886	100.000

***tert*-Butyl-((*R*)-1-(1,1-dioxidobenzoisothiazol-2(3*H*)-yl)-1-oxohexan-2-yl)-*L*-alaninate (4b)**

Prepared according to the **GENERAL PROCEDURE C** with ($S_{S(IV)}$, $S_{C\text{-}1}$)-**S4b** (74.8 mg, 0.30 mmol, >99:1 d.r.) and **2a** (50.0 mg, 0.20 mmol) as substrates. Column chromatography: silica gel, petroleum ether/ethyl acetate/dichloromethane = 10:1:1. Colorless viscous oil (63.1 mg, 77% yield, >99:1 d.r.).

^{1}H NMR (400 MHz, $CDCl_3$) δ 7.79 (d, *J* = 7.9 Hz, 1H), 7.69 (t, *J* = 7.7 Hz, 1H), 7.57 (t, *J* = 7.8 Hz, 1H), 7.47 (d, *J* = 7.8 Hz, 1H), 5.00 (d, *J* = 15.9 Hz, 1H), 4.88 (d, *J* = 15.9 Hz, 1H), 4.10 – 3.95 (m, 1H), 3.27 (q, *J* = 7.0 Hz, 1H), 2.44 (s, 1H), 1.82 (tt, *J* = 11.5, 5.4 Hz, 1H), 1.64 – 1.48 (m, 3H), 1.43 – 1.33 (s, 11H), 1.29 (d, *J* = 7.0 Hz, 3H), 0.87 (t, *J* = 7.3 Hz, 3H).

^{13}C NMR (101 MHz, $CDCl_3$) δ 174.9, 174.1, 134.4, 134.2, 130.9, 129.8, 125.1, 121.8, 81.0, 59.8, 55.6, 47.4, 34.5, 28.0, 22.6, 18.8, 14.0.

HRMS (ESI, m/z): $[M+H]^+$ Calcd. For $C_{20}H_{31}N_2O_5S$: 411.1954; Found: 411.1955.

HPLC (Chiralpak OD-H Column), *i*-PrOH/hexane = 1/99, flow rate = 1.0 mL/min, λ = 254 nm; t_R = 27.2 min (major), t_R = 29.7 min (minor).

$[\alpha]_D^{25}$ = –4.00 (c = 0.5, $CHCl_3$).

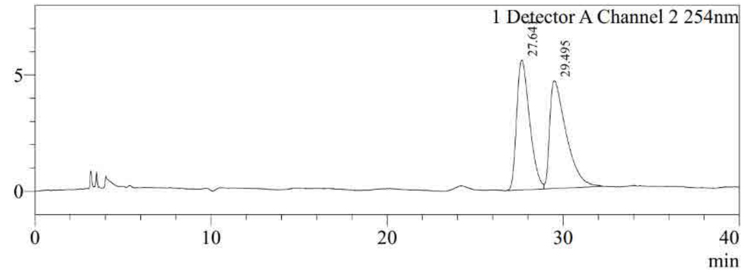

Detector A Channel 2 254nm

Peak#	Ret. Time	USP Width	Area	Height	Area%
1	27.641	1.361	285422	5589	48.483
2	29.495	1.718	303288	4636	51.517
Total			588710	10225	100.000

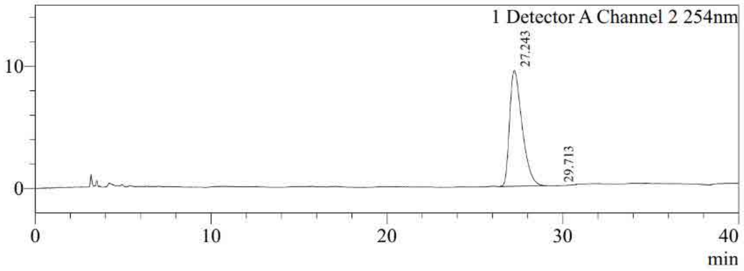

Detector A Channel 2 254nm

Peak#	Ret. Time	USP Width	Area	Height	Area%
1	27.243	1.296	468800	9465	99.998
2	29.713	0.064	9	-0	0.002
Total			468809	9465	100.000

Synthesis of ($R_{C\text{-}1}$, $S_{C\text{-}2}$)-4c

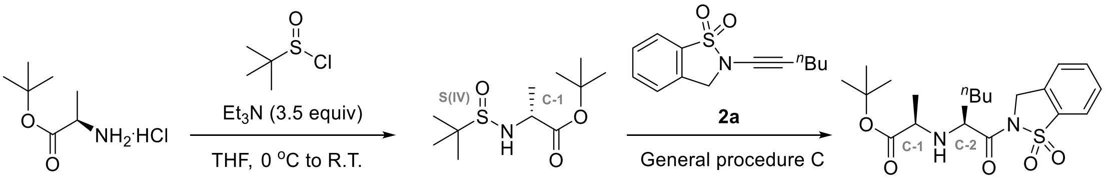

($R_{S(IV)}$, $R_{C\text{-}1}$)-**S4c** and ($S_{S(IV)}$, $R_{C\text{-}1}$)-**S4a**

($R_{C\text{-}1}$, $S_{C\text{-}2}$)-**4c**

Synthesis of ($R_{S(IV)}$, $R_{C\text{-}1}$)-**S4c**

The title compound was obtained as an isomer during the the preparation of **S4a**. Colorless oil (1.61 g, 64% yield, >99:1 d.r.)

^{1}H NMR (400 MHz, $CDCl_3$) δ 4.11 (d, *J* = 5.2 Hz, 1H), 3.90 – 3.76 (m, 1H), 1.40 (s, 9H), 1.30 (d, *J* = 7.0 Hz, 3H), 1.16 (s, 9H).

^{13}C NMR (101 MHz, $CDCl_3$) δ 172.6, 82.2, 55.6, 53.0, 27.9, 22.5, 19.8.

HRMS (ESI, m/z): $[M+Na]^+$ Calcd. For $C_{11}H_{23}NO_3NaS$: 272.1296; Found: 272.1299.

HPLC (Chiralpak OD-H Column), *i*-PrOH/hexane = 3/97, flow rate = 1.0 mL/min, λ = 210 nm; t_R = 5.7 min (major), t_R = 6.8 min (minor).

$[\alpha]_D^{20}$ = –86.4 (c = 1.0, $CHCl_3$).

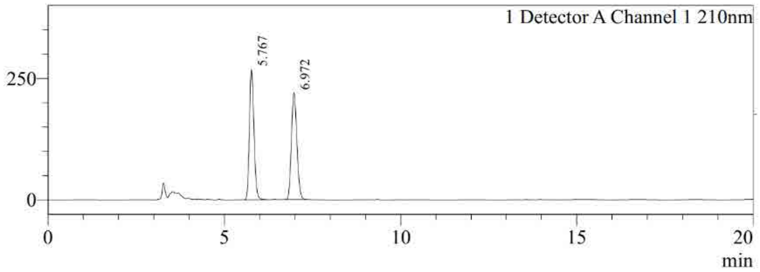

Detector A Channel 1 210nm

Peak#	Ret. Time	USP Width	Area	Height	Area%
1	5.767	0.246	2406130	267697	50.476
2	6.972	0.292	2360746	218700	49.524
Total			4766876	486397	100.000

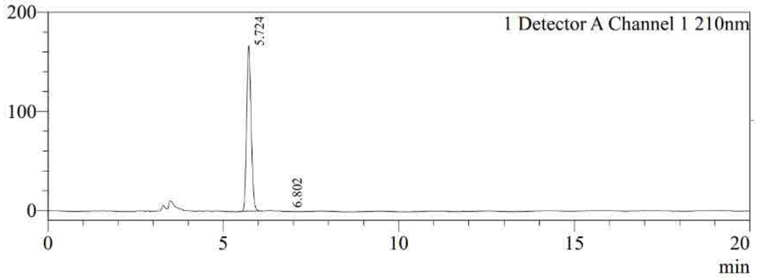

Detector A Channel 1 210nm

Peak#	Ret. Time	USP Width	Area	Height	Area%
1	5.724	0.246	1492552	166594	99.984
2	6.802	0.098	238	89	0.016
Total			1492790	166683	100.000

***tert*-Butyl-((*S*)-1-(1,1-dioxidobenzoisothiazol-2(3*H*)-yl)-1-oxohexan-2-yl)-*D*-alaninate (4c)**

Prepared according to the **GENERAL PROCEDURE C** with ($R_{S(IV)}$, $R_{C\text{-}1}$)-**S4c** (74.8 mg, 0.30 mmol, >99:1 d.r.) and **2a** (50.0 mg, 0.20 mmol) as substrates. Column chromatography: silica gel, petroleum ether/ethyl acetate/dichloromethane = 10:1:1. Colorless viscous oil (63.2 mg, 77% yield, >99:1 d.r.).

^{1}H NMR (400 MHz, $CDCl_3$) δ 7.79 (d, *J* = 7.9 Hz, 1H), 7.69 (t, *J* = 7.6 Hz, 1H), 7.58 (t, *J* = 7.7 Hz, 1H), 7.47 (d, *J* = 7.8 Hz, 1H), 5.01 (d, *J* = 15.9 Hz, 1H), 4.89 (d, *J* = 15.9 Hz, 1H), 4.04 (t, *J* = 6.0 Hz, 1H), 3.27 (q, *J* = 6.9 Hz, 1H), 2.57 (s, 1H), 1.91 – 1.75 (m, 1H), 1.65 – 1.53 (m, 2H), 1.37 (s, 9H), 1.29 (d, *J* = 7.0 Hz, 3H), 0.88 (td, *J* = 7.2, 2.0 Hz, 3H).

^{13}C NMR (101 MHz, $CDCl_3$) δ 174.9, 174.1, 134.5, 134.2, 130.9, 129.8, 125.1, 121.8, 81.0, 59.8, 55.7, 47.4, 34.5, 28.0, 28.0, 22.6, 18.8, 14.0.

HRMS (ESI, m/z): $[M+H]^+$ Calcd. For $C_{20}H_{31}N_2O_5S$: 411.1954; Found: 411.1956.

HPLC (Chiralpak OD-H Column), *i*-PrOH/hexane = 2/98, flow rate = 1.0 mL/min, λ = 254 nm; t_R = 16.2 min (major), t_R = 18.6 min (minor).

[α]D[25] = +3.60 (c = 0.5, $CHCl_3$).

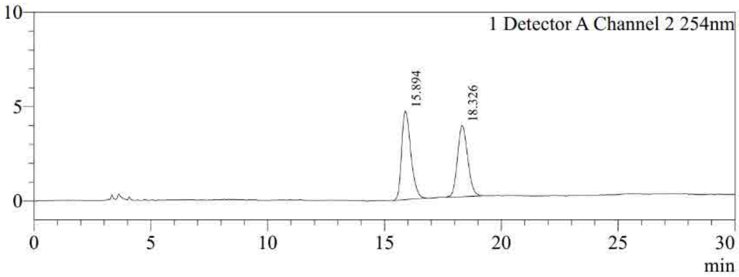

Detector A Channel 2 254nm

Peak#	Ret. Time	USP Width	Area	Height	Area%
1	15.894	0.720	129176	4703	52.870
2	18.326	0.800	115152	3778	47.130
Total			244327	8482	100.000

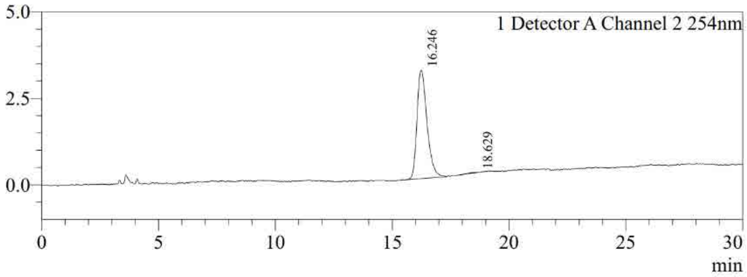

Detector A Channel 2 254nm

Peak#	Ret. Time	USP Width	Area	Height	Area%
1	16.246	0.779	93568	3137	99.712
2	18.629	0.588	270	-2	0.288
Total			93839	3135	100.000

Synthesis of (S_{C-1}, S_{C-2})-4d

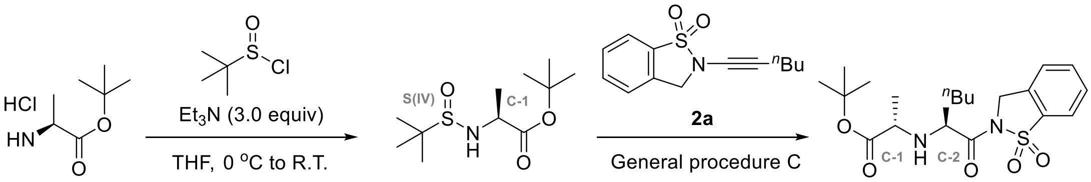

($R_{S(IV)}$, S_{C-1})-**S4d**
and
($S_{S(IV)}$, S_{C-1})-**S4b**

(S_{C-1}, S_{C-2})-**4d**

Synthesis of ($R_{S(IV)}$, S_{C-1})-**S4d**

The title compound was obtained as an isomer during the the preparation of **S4b**. White solid (158 mg, 21% yield, >99:1 d.r.)

^{1}H NMR (400 MHz, $CDCl_3$) δ 3.92 – 3.85 (m, 1H), 3.63 (d, J = 8.1 Hz, 1H), 1.44 – 1.38 (m, 12H), 1.18 (s, 9H).

^{13}C NMR (101 MHz, $CDCl_3$) δ 172.4, 81.8, 56.4, 54.3, 28.0, 22.6, 20.8.

HRMS (ESI, m/z): $[M+Na]^+$ Calcd. For $C_{11}H_{23}NO_3NaS$: 272.1296; Found: 272.1295.

HPLC (Chiralpak OD-H Column), *i*-PrOH/hexane = 5/95, flow rate = 1.0 mL/min, λ = 210 nm; t_R = 7.2 min

(minor), t_R = 8.8 min (major).

[α]$_D^{20}$ = −50.8 (c = 1.0, $CHCl_3$).

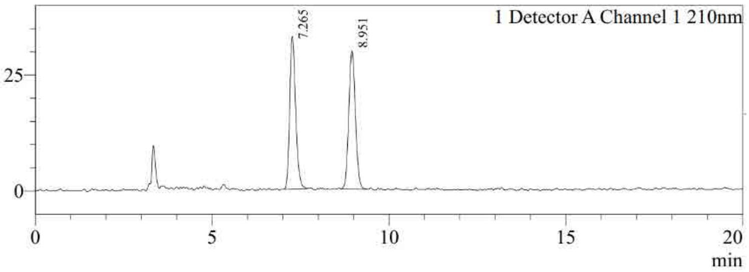

Detector A Channel 1 210nm

Peak#	Ret. Time	USP Width	Area	Height	Area%
1	7.265	0.310	382931	32895	49.650
2	8.951	0.350	388333	29709	50.350
Total			771264	62604	100.000

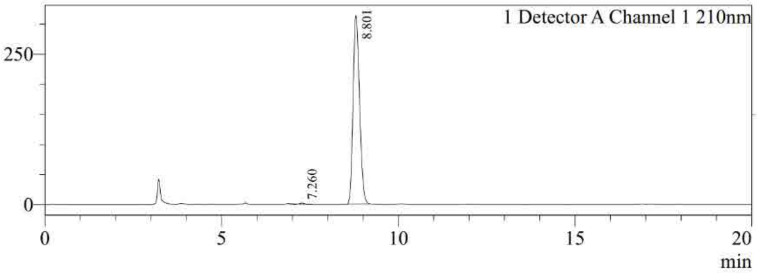

Detector A Channel 1 210nm

Peak#	Ret. Time	USP Width	Area	Height	Area%
1	7.260	0.258	8044	1709	0.201
2	8.801	0.345	3985826	312425	99.799
Total			3993870	314134	100.000

***tert*-Butyl-((*S*)-1-(1,1-dioxidobenzoisothiazol-2(3*H*)-yl)-1-oxohexan-2-yl)-*L*-alaninate (4d)**

Prepared according to the **GENERAL PROCEDURE C** with ($R_{S(IV)}$, S_{C-1})-**S4d** (74.8 mg, 0.30 mmol, >99:1 d.r.) and **2a** (50.0 mg, 0.20 mmol) as substrates. Column chromatography: silica gel, petroleum ether/ethyl acetate/dichloromethane = 10:1:1. Colorless viscous oil (65.5 mg, 80% yield, >99:1 d.r.).

^{1}H NMR (400 MHz, $CDCl_3$) δ 7.78 (d, *J* = 7.9 Hz, 1H), 7.68 (t, *J* = 7.6 Hz, 1H), 7.56 (t, *J* = 7.7 Hz, 1H), 7.45 (d, *J* = 7.8 Hz, 1H), 4.98 (d, *J* = 15.8 Hz, 1H), 4.86 (d, *J* = 15.8 Hz, 1H), 4.14 (t, *J* = 6.3 Hz, 1H), 3.26 (q, *J* = 7.0 Hz, 1H), 2.16 (s, 1H), 1.92 – 1.77 (m, 1H), 1.66 – 1.48 (m, 3H), 1.44 (s, 9H), 1.36 – 1.29 (m, 2H), 1.26 (d, *J* = 7.0 Hz, 3H), 0.87 (t, *J* = 7.2 Hz, 3H).

^{13}C NMR (101 MHz, $CDCl_3$) δ 174.5, 174.2, 134.5, 134.1, 130.8, 129.7, 125.0, 121.9, 81.0, 60.3, 55.1, 47.5, 33.4, 28.1, 22.6, 19.8, 14.0.

HRMS (ESI, m/z): $[M+H]^+$ Calcd. For $C_{20}H_{31}N_2O_5S$: 411.1954; Found: 411.1955.

HPLC (Chiralpak OD-H Column), *i*-PrOH/hexane = 1/99, flow rate = 1.0 mL/min, λ = 254 nm; t_R = 27.4 min (minor), t_R = 29.3 min (major).

[α]$_D^{25}$ = −19.2 (c = 0.5, $CHCl_3$).

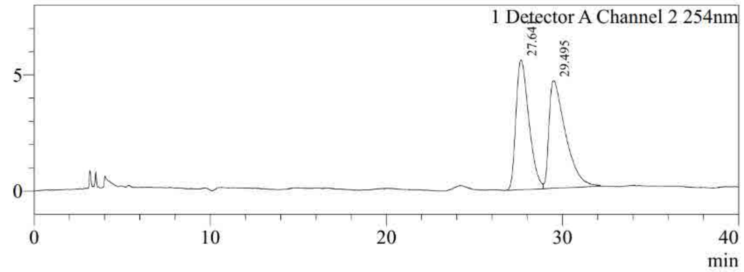

Detector A Channel 2 254nm

Peak#	Ret. Time	USP Width	Area	Height	Area%
1	27.641	1.361	285422	5589	48.483
2	29.495	1.718	303288	4636	51.517
Total			588710	10225	100.000

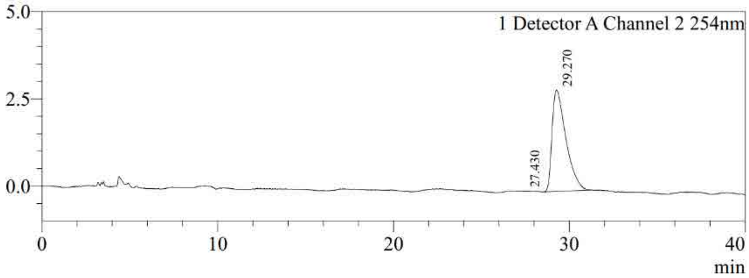

Detector A Channel 2 254nm

Peak#	Ret. Time	USP Width	Area	Height	Area%
1	27.430	0.112	101	13	0.063
2	29.270	1.458	159800	2906	99.937
Total			159901	2918	100.000

*The absolute configuration of S(IV) centers in **S4a** ~ **S4d** prepared above were further confirmed by comparison with the same products derived from another procedure (66) using optically pure tert-butanesulfinamide as the starting material.*

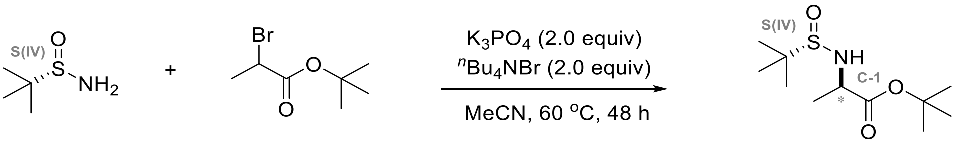

(0.50 mmol) (1.0 mmol) ($S_{S(IV)}$, R_{C-1})-**S4a** and ($S_{S(IV)}$, S_{C-1})-**S4b**

The mixture of (*S*)-*tert*-butanesulfinamide (60.5 mg, 0.50 mmol, 1.0 equiv), *tert*-butyl 2-bromopropanoate (209 mg, 1.0 mmol, 1.0 equiv), K_3PO_4 (212 mg, 1.0 mmol, 2.0 equiv), and $^nBu_4NBr$ (323 mg, 1.0 mmol, 2.0 equiv) in MeCN (2.0 mL) was stirred at 60 °C for 48 hours. After regular workup, the crude products were purified through flash chromatography (eluent: petroleum ether/ethyl acetate = 5:1 to 3:1) to afford two diastereoisomers ($S_{S(IV)}$, R_{C-1})-**S4a** (19.5 mg) and primary product ($S_{S(IV)}$, S_{C-1})-**S4b** (48.0 mg).

Synthesis of ($R_{C\text{-}1}$, $S_{C\text{-}2}$)-5a

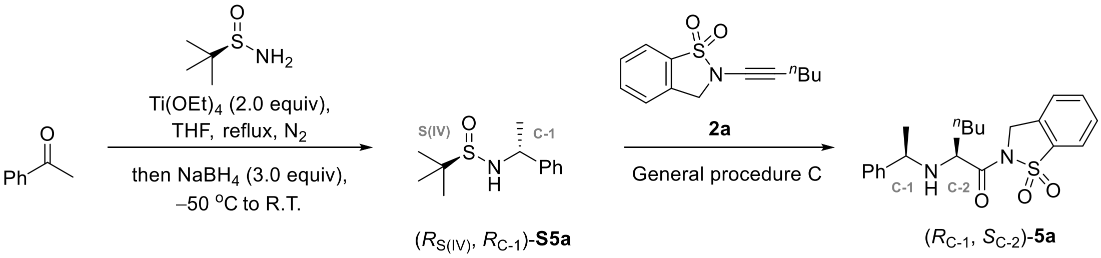

Synthesis of ($R_{S(IV)}$, $R_{C\text{-}1}$)-**S5a** (*67*)

Based on the literature procedure (*57*): Under N_2 atmosphere, the mixture of (*R*)-*tert*-butanesulfinamide (1.44 g, 12 mmol, 1.0 equiv), ketone (1.73 g, 14.4 mmol, 1.2 equiv) and $Ti(OEt)_4$ (5.47 g, 24 mmol, 2.0 equiv) in THF was refluxed overnight. After completion, $NaBH_4$ (1.36 g, 36 mmol, 3.0 equiv) was added in batches at −50 °C, and the reaction mixture was warmed to room temperature over a period of 3 hours (monitored by TLC), then quenched with MeOH and saturated brine at 0 °C. The resulting suspension was filtered and washed with ethyl acetate. The filtrate was collected and extracted with ethyl acetate. The combined organic layer was washed with saturated brine and concentrated under vacuum. The crude product was purified through flash chromatography (eluent: petroleum ether/ethyl acetate = 10:1 to 5:1) to afford ($R_{S(IV)}$, $R_{C\text{-}1}$)-**S5a** as colorless viscous oil (1.65 g, 61% yield, >99:1 d.r.).

^{1}H NMR (400 MHz, $CDCl_3$) δ 7.54 – 7.08 (m, 5H), 4.51 (qd, *J* = 6.5, 2.8 Hz, 1H), 3.59 – 3.37 (m, 1H), 1.47 (d, *J* = 6.6 Hz, 3H), 1.19 (s, 9H).

HPLC (Chiralpak OD-H Column), *i*-PrOH/hexane = 3/97, flow rate = 1.0 mL/min, λ = 210 nm; t_R = 8.4 min (major), t_R = 10.6 min (minor).

$[\alpha]_D^{20}$ = –43.4 (c = 1.0, EA).

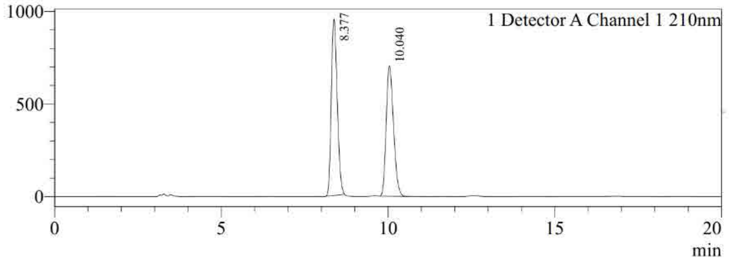

Detector A Channel 1 210nm

Peak#	Ret. Time	USP Width	Area	Height	Area%
1	8.377	0.334	11757470	953589	52.175
2	10.040	0.411	10777274	704528	47.825
Total			22534745	1658117	100.000

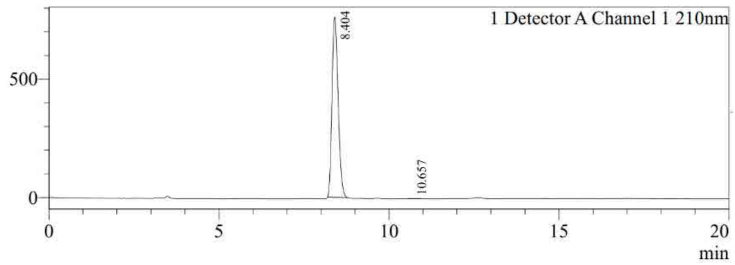

Detector A Channel 1 210nm

Peak#	Ret. Time	USP Width	Area	Height	Area%
1	8.404	0.338	9507855	760489	99.988
2	10.657	0.109	1127	218	0.012
Total			9508982	760706	100.000

(*S*)-1-(1,1-Dioxidobenzoisothiazol-2(3*H*)-yl)-2-(((*R*)-1-phenylethyl)amino)hexan-1-one (5a)

Prepared according to the **GENERAL PROCEDURE C** with ($R_{S(IV)}$, R_{C-1})-**S5a** (67.5 mg, 0.30 mmol, >99:1 d.r.) and **2a** (50.0 mg, 0.20 mmol) as substrates. Column chromatography: silica gel, petroleum ether/ethyl acetate = 15:1. Colorless viscous oil (67.3 mg, 87% yield, 99:1 d.r.).

^{1}H NMR (400 MHz, $CDCl_3$) δ 7.79 (d, *J* = 7.9 Hz, 1H), 7.67 (t, *J* = 7.6 Hz, 1H), 7.56 (t, *J* = 7.7 Hz, 1H), 7.42 (d, *J* = 7.9 Hz, 1H), 7.33 (d, *J* = 7.6 Hz, 2H), 7.23 (d, *J* = 6.0 Hz, 2H), 7.20 – 7.12 (m, 1H), 4.80 (d, *J* = 16.0 Hz, 1H), 4.74 (d, *J* = 15.6 Hz, 1H), 4.17 (s, 1H), 3.76 (q, *J* = 6.5 Hz, 1H), 2.08 – 1.96 (m, 1H), 1.83 (td, *J* = 10.0, 8.4, 4.3 Hz, 1H), 1.64 – 1.41 (m, 3H), 1.38 (d, *J* = 6.5 Hz, 3H), 1.36 – 1.29 (m, 2H), 0.88 (t, *J* = 7.6 Hz, 3H).

^{13}C NMR (101 MHz, $CDCl_3$) δ 175.2, 145.8, 134.5, 134.1, 130.9, 129.8, 128.3, 127.1, 127.0, 125.1, 121.9, 59.8, 56.4, 47.3, 34.1, 3.1, 22.6, 22.6, 14.1.

HRMS (ESI, m/z): $[M+H]^+$ Calcd. For $C_{21}H_{27}N_2O_3S$: 387.1742; Found: 387.1745.

HPLC (Chiralpak AD-H Column), *i*-PrOH/hexane = 3/97, flow rate = 1.0 mL/min, λ = 210 nm; t_R = 29.2 min (minor), t_R = 31.5 min (major).

$[\alpha]_D^{25}$ = +15.8 (c = 0.38, $CHCl_3$).

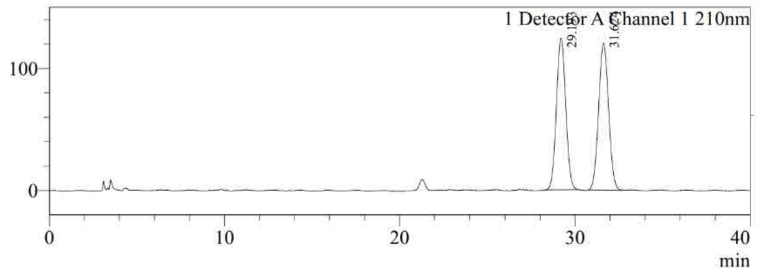

Detector A Channel 1 210nm

Peak#	Ret. Time	USP Width	Area	Height	Area%
1	29.193	0.987	4609895	124157	49.074
2	31.624	1.059	4783831	120296	50.926
Total			9393726	244454	100.000

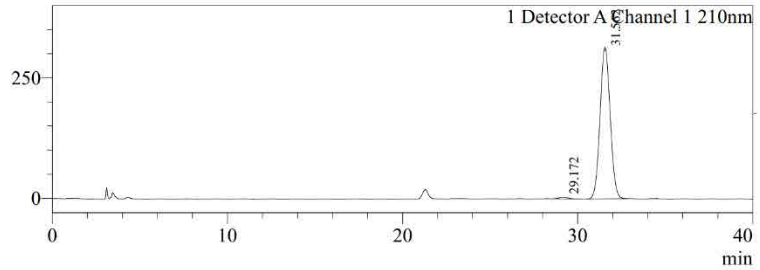

Detector A Channel 1 210nm

Peak#	Ret. Time	USP Width	Area	Height	Area%
1	29.172	0.936	122002	3310	0.959
2	31.562	1.065	12596811	314148	99.041
Total			12718812	317458	100.000

Synthesis of ($R_{C\text{-}1}$, $R_{C\text{-}2}$)-5b

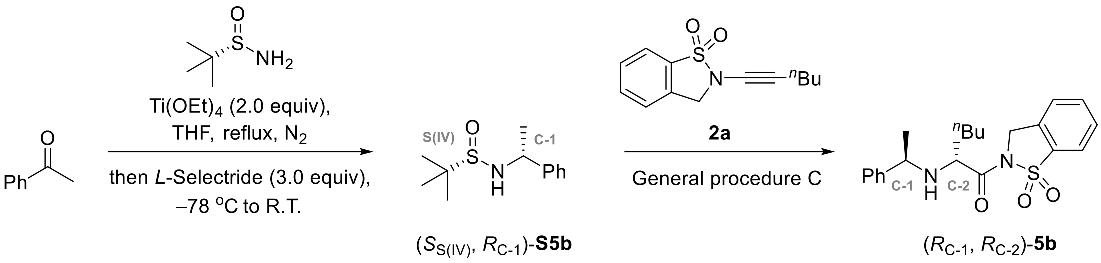

Synthesis of ($S_{S(IV)}$, $R_{C\text{-}1}$)**-S5b**

Based on the literature procedure (*57*): Under N_2 atmosphere, the mixture of (*S*)-*tert*-butanesulfinamide (1.21 g, 10 mmol, 1.0 equiv), ketone (1.44 g, 12 mmol, 1.2 equiv) and $Ti(OEt)_4$ (4.56 g, 20 mmol, 2.0 equiv) in THF was refluxed overnight. After completion, *L*-Selectride (30 mL, 3.0 equiv, 1.0 M in THF) was added dropwise at −78 °C, and the reaction mixture was stirred at the same temperature for 3 hours then warmed to room temperature and stirred for another 3 hours (monitored by TLC), followed by quenching with MeOH and saturated brine at 0 °C. The resulting suspension was filtered and washed with ethyl acetate. The filtrate was collected and extracted with ethyl acetate. The combined organic layer was washed with brine and concentrated under vacuum. The crude product was purified through flash chromatography (eluent: petroleum ether/ethyl acetate = 10:1 to 5:1) to afford ($S_{S(IV)}$, $R_{C\text{-}1}$)-**S5b** as white solid (0.741 g, 33% yield, >99:1 d.r.).

^{1}H NMR (400 MHz, $CDCl_3$) δ 7.52 – 7.12 (m, 5H), 4.56 (qd, *J* = 6.7, 3.4 Hz, 1H), 3.37 (d, *J* = 3.8 Hz, 1H), 1.53 (d, *J* = 6.7 Hz, 3H), 1.19 (s, 9H).

^{13}C NMR (101 MHz, $CDCl_3$) δδ 143.5, 128.6, 127.6, 127.0, 55.6, 54.7, 25.3, 22.6.

HRMS (ESI, m/z): $[M+Na]^+$ Calcd. For $C_{12}H_{19}NONaS$: 248.1085; Found: 248.1086.

HPLC (Chiralpak OD-H Column), *i*-PrOH/hexane = 3/97, flow rate = 1.0 mL/min, λ = 210 nm; t_R = 8.6 min (minor), t_R = 10.0 min (major).

$[\alpha]_D^{20}$ = +86.8 (c = 1.0, EA).

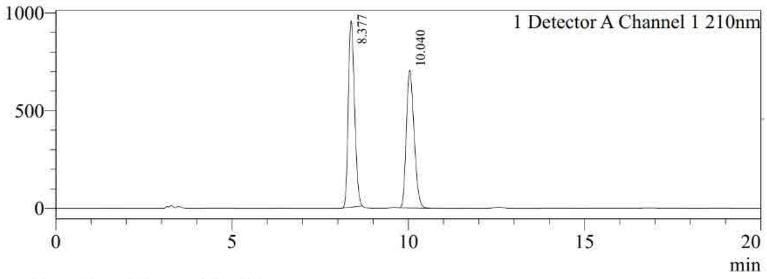

Detector A Channel 1 210nm

Peak#	Ret. Time	USP Width	Area	Height	Area%
1	8.377	0.334	11757470	953589	52.175
2	10.040	0.411	10777274	704528	47.825
Total			22534745	1658117	100.000

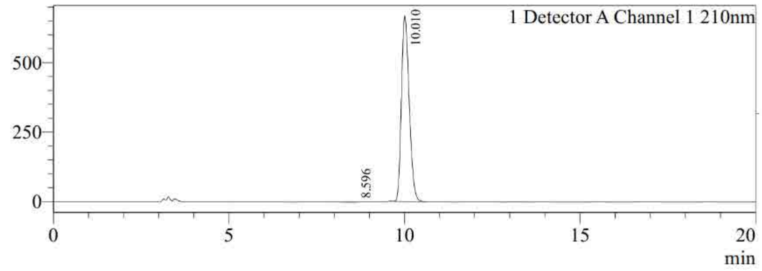

Detector A Channel 1 210nm

Peak#	Ret. Time	USP Width	Area	Height	Area%
1	8.596	0.064	363	89	0.004
2	10.010	0.412	10252867	667118	99.996
Total			10253231	667207	100.000

(*R*)-1-(1,1-Dioxidobenzoisothiazol-2(3*H*)-yl)-2-(((*R*)-1-phenylethyl)amino)hexan-1-one (5b)

Prepared according to the **GENERAL PROCEDURE C** with ($S_{S(IV)}$, $R_{C\text{-}1}$)-**S5b** (67.5 mg, 0.30 mmol, >99:1 d.r.) and **2a** (50.0 mg, 0.20 mmol) as substrates. Column chromatography: silica gel, petroleum ether/ethyl acetate = 15:1. Colorless viscous oil (64.3 mg, 83% yield, 98:2 d.r.).

^{1}H NMR (400 MHz, $CDCl_3$) δ 7.74 (d, *J* = 7.9 Hz, 1H), 7.68 (t, *J* = 7.6 Hz, 1H), 7.55 (t, *J* = 7.7 Hz, 1H), 7.44 (d, *J* = 8.0 Hz, 1H), 7.40 (d, *J* = 7.6 Hz, 2H), 7.31 (t, *J* = 7.4 Hz, 2H), 7.23 (t, *J* = 7.3 Hz, 1H), 5.09 – 4.92 (m, 1H), 4.91 – 4.69 (m, 1H), 4.00 – 3.78 (m, 1H), 3.74 (q, *J* = 6.6 Hz, 1H), 2.26 (s, 1H), 1.88 – 1.72 (m, 1H), 1.64 – 1.45 (m, 2H), 1.43 – 1.38 (m, 1H), 1.36 (d, *J* = 6.5 Hz, 3H), 1.30 – 1.20 (m, 2H), 0.87 (t, *J* = 7.3 Hz, 3H).

^{13}C NMR (101 MHz, $CDCl_3$) δ 175.9, 145.0, 134.5, 134.1, 130.7, 129.7, 128.3, 127.2, 127.1, 125.0, 121.8, 59.8, 56.9, 47.4, 34.2, 28.1, 25.6, 22.4, 14.0.

HRMS (ESI, m/z): $[M+H]^+$ Calcd. For $C_{21}H_{27}N_2O_3S$: 387.1742; Found: 387.1745.

HPLC (Chiralpak AD-H Column), *i*-PrOH/hexane = 3/97, flow rate = 1.0 mL/min, λ = 210 nm; t_R = 29.2 min (major), t_R = 31.5 min (minor).

$[\alpha]_D^{25}$ = +53.7 (c = 0.38, $CHCl_3$).

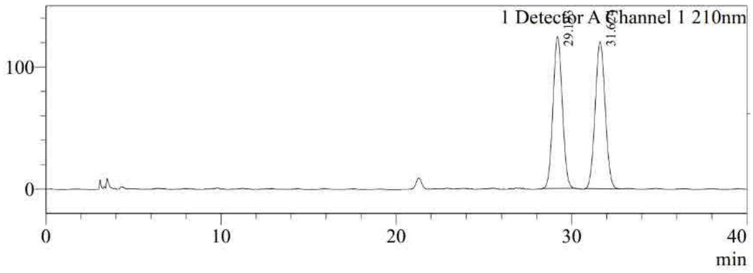

Detector A Channel 1 210nm

Peak#	Ret. Time	USP Width	Area	Height	Area%
1	29.193	0.987	4609895	124157	49.074
2	31.624	1.059	4783831	120296	50.926
Total			9393726	244454	100.000

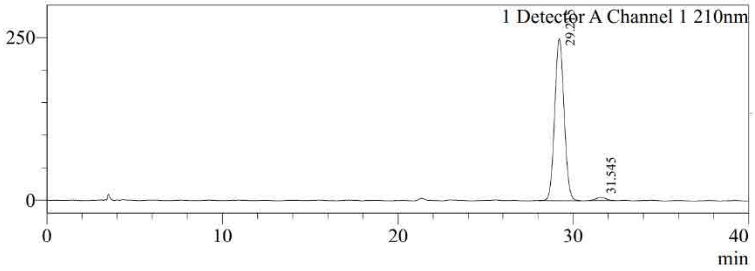

Detector A Channel 1 210nm

Peak#	Ret. Time	USP Width	Area	Height	Area%
1	29.215	0.988	9266135	248880	98.004
2	31.545	0.995	188719	4804	1.996
Total			9454854	253684	100.000

Synthesis of ($S_{C\text{-}1}$, $S_{C\text{-}2}$)-6a

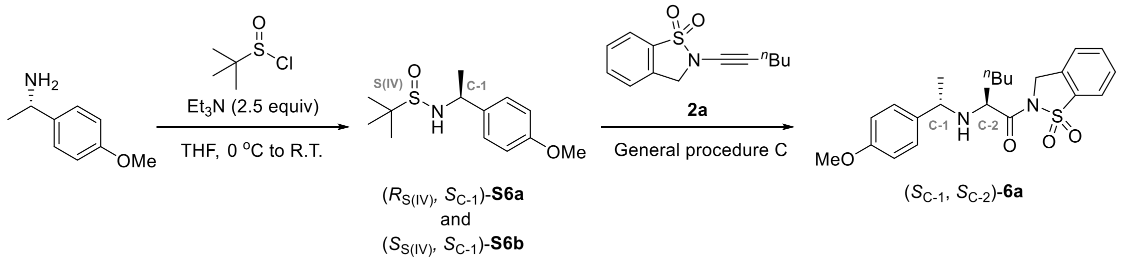

Synthesis of ($R_{S(IV)}$, $S_{C\text{-}1}$)-**S6a** (*68*)

Under N_2 atmosphere, the mixture of (*S*)-1-(4-methoxyphenyl)ethan-1-amine (1.51 g, 10 mmol, 1.0 equiv), Et_3N (2.53 g, 25 mmol, 2.5 equiv) in THF was cooled at 0 °C and then 2-methylpropane-2-sulfinic chloride (*69*). (20mmol, 2.0 equiv) dissolved in THF was added slowly. The resulted mixture was stirred at room temperature overnight and then quenched with saturated aqueous NH_4Cl, extracted with ethyl acetate. The combined organic layer was washed with brine and concentrated under vacuum. The crude product was purified through flash chromatography (eluent: petroleum ether/ethyl acetate = 4:1 to 3:1) to afford ($R_{S(IV)}$, $S_{C\text{-}1}$)-**S6a** as white solid (332 mg, 13% yield, >99:1 d.r.) and ($S_{S(IV)}$, $S_{C\text{-}1}$)-**S6b** as colorless viscous oil (1.37 g, 54% yield, >99:1 d.r.). These two compounds were used for the preparation of **6a** and **6b** respectively.

^{1}**H NMR** (400 MHz, $CDCl_3$) δ 7.24 (d, J = 8.4 Hz, 2H), 6.86 (d, J = 8.6 Hz, 2H), 4.55 – 4.49 (m, 1H), 3.79 (s, 3H), 3.31 (d, J = 3.7 Hz, 1H), 1.51 (d, J = 6.7 Hz, 3H), 1.18 (s, 9H).

HPLC (Chiralpak OD-H Column), *i*-PrOH/hexane = 5/95, flow rate = 1.0 mL/min, λ = 210 nm; t_R = 8.1 min (minor), t_R = 14.9 min (major).

$[\alpha]_D^{20}$ = –86.8 (c = 1.0, $CHCl_3$).

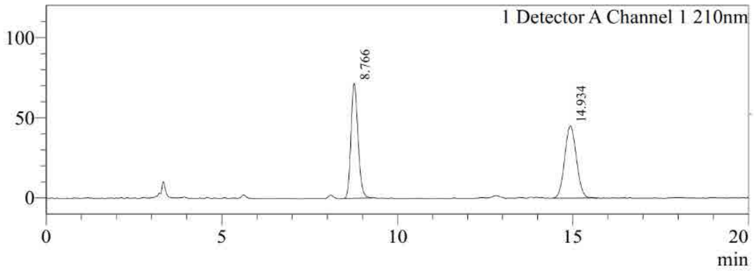

Detector A Channel 1 210nm

Peak#	Ret. Time	USP Width	Area	Height	Area%
1	8.766	0.372	997115	71619	48.877
2	14.934	0.617	1042922	44902	51.123
Total			2040037	116522	100.000

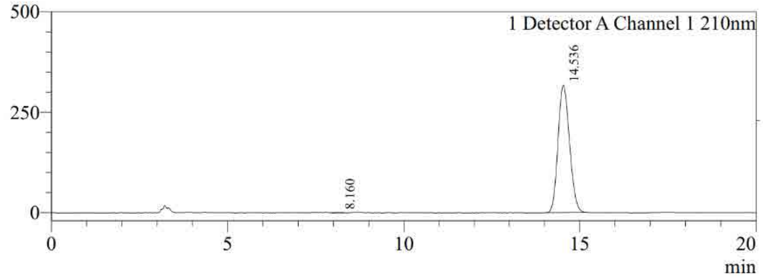

Detector A Channel 1 210nm

Peak#	Ret. Time	USP Width	Area	Height	Area%
1	8.160	0.160	938	447	0.013
2	14.536	0.608	7191760	316426	99.987
Total			7192699	316874	100.000

(*S*)-1-(1,1-Dioxidobenzoisothiazol-2(3*H*)-yl)-2-(((*S*)-1-(4-methoxyphenyl)ethyl)amino)hexan-1-one (6a)

Prepared according to the **GENERAL PROCEDURE C** with ($R_{S(IV)}$, $S_{C\text{-}1}$)-**S6a** (76.6 mg, 0.30 mmol, >99:1 d.r.) and **2a** (50.0 mg, 0.20 mmol) as substrates. Column chromatography: silica gel, petroleum ether/ethyl acetate = 10:1. Colorless viscous oil (61.8 mg, 74% yield, 96:4 d.r.).

^{1}**H NMR** (400 MHz, $CDCl_3$) δ 7.75 (d, J = 7.9 Hz, 1H), 7.68 (t, J = 7.6 Hz, 1H), 7.56 (t, J = 7.6 Hz, 1H), 7.45 (d, J = 7.9 Hz, 1H), 7.31 (d, J = 8.2 Hz, 2H), 6.84 (d, J = 8.2 Hz, 2H), 5.10 – 4.70 (m, 2H), 3.86 – 3.73 (m, 4H), 3.69 (q, J = 6.6 Hz, 1H), 2.36 – 2.13 (m, 1H), 1.84 – 1.72 (m, 1H), 1.60 – 1.45 (m, 2H), 1.43 – 1.25 (m, 6H), 0.86 (t, J = 7.3 Hz, 3H).

^{13}**C NMR** (101 MHz, $CDCl_3$) δ 172.4, 158.8, 137.0, 134.7, 134.1, 130.8, 129.8, 128.3, 125.0, 121.9, 113.7, 59.8, 56.4, 55.3, 47.4, 34.2, 28.1, 25.5, 22.4, 14.0.

HRMS (ESI, m/z): $[M+H]^+$ Calcd. For $C_{22}H_{29}N_2O_4S$: 417.1848; Found: 417.1849.

HPLC (Chiralpak OD-H Column), *i*-PrOH/hexane = 3/97, flow rate = 1.0 mL/min, λ = 210 nm; t_R = 17.1 min (major), t_R = 20.7 min (minor).

[α]D[25] = –60.0 (c = 0.45, $CHCl_3$).

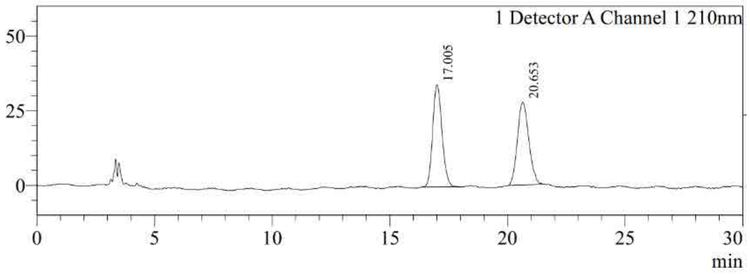

Detector A Channel 1 210nm

Peak#	Ret. Time	USP Width	Area	Height	Area%
1	17.005	0.733	947153	34202	50.321
2	20.653	0.882	935085	27772	49.679
Total			1882238	61974	100.000

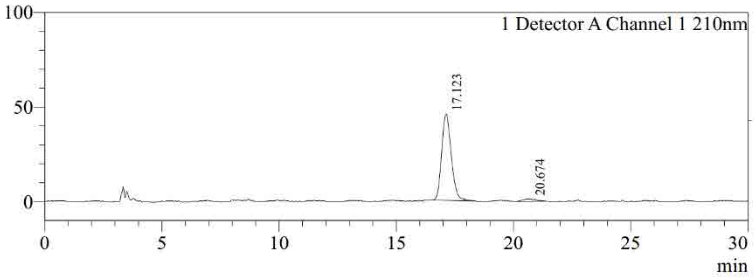

Detector A Channel 1 210nm

Peak#	Ret. Time	USP Width	Area	Height	Area%
1	17.123	0.742	1302541	45684	96.425
2	20.674	1.037	48288	1275	3.575
Total			1350829	46959	100.000

Synthesis of (S_{C-1}, R_{C-2},)-6b

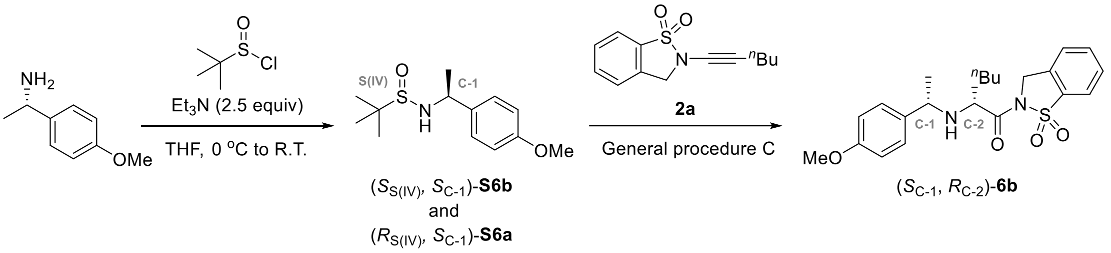

Synthesis of ($S_{S(IV)}$, S_{C-1})-**S6b** (*68*)

The title compound was obtained as an isomer during the the preparation of **S6a**. Colorless viscous oil (1.37 g, 54% yield, >99:1 d.r.).

^{1}H NMR (400 MHz, $CDCl_3$) δ 7.24 (d, *J* = 8.7 Hz, 1H), 6.84 (d, *J* = 8.8 Hz, 1H), 4.50 – 4.44 (m, 1H), 3.76 (s, 3H), 3.39 (s, 1H), 1.45 (d, *J* = 6.6 Hz, 3H), 1.19 (s, 9H).

HPLC (Chiralpak OD-H Column), *i*-PrOH/hexane = 5/95, flow rate = 1.0 mL/min, λ = 210 nm; t_R = 8.6 min (major), t_R = 14.9 min (minor).

[α]D[20] = +36.2 (c = 1.0, $CHCl_3$).

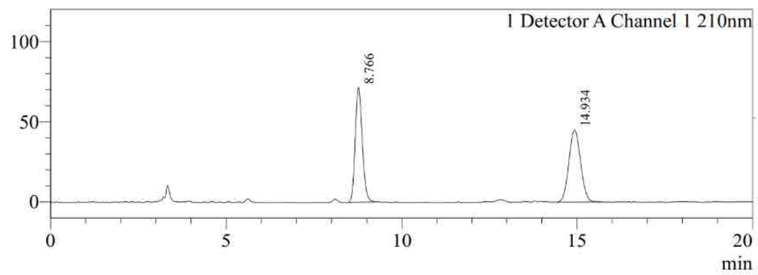

Detector A Channel 1 210nm

Peak#	Ret. Time	USP Width	Area	Height	Area%
1	8.766	0.372	997115	71619	48.877
2	14.934	0.617	1042922	44902	51.123
Total			2040037	116522	100.000

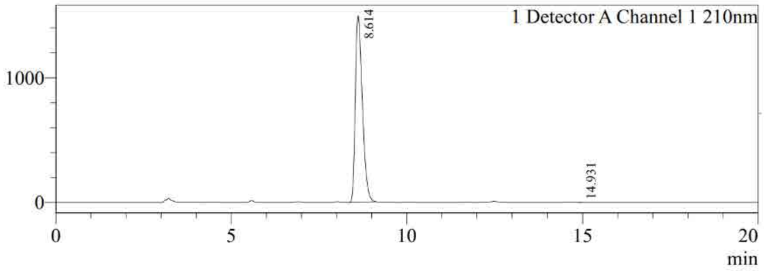

Detector A Channel 1 210nm

Peak#	Ret. Time	USP Width	Area	Height	Area%
1	8.614	0.373	20852501	1493933	99.999
2	14.931	0.058	107	64	0.001
Total			20852608	1493997	100.000

(*R*)-1-(1,1-Dioxidobenzoisothiazol-2(3*H*)-yl)-2-(((*S*)-1-(4-methoxyphenyl)ethyl)amino)hexan-1-one (6b)

Prepared according to the **GENERAL PROCEDURE C** with ($S_{S(IV)}$, $S_{C\text{-}1}$)-**S6b** (76.6 mg, 0.30 mmol, >99:1 d.r.) and **2a** (50.0 mg, 0.20 mmol) as substrates. Column chromatography: silica gel, petroleum ether/ethyl acetate = 10:1. Colorless viscous oil (62.7 mg, 75% yield, 99:1 d.r.).

^{1}H NMR (400 MHz, $CDCl_3$) δ 7.81 (d, J = 7.9 Hz, 1H), 7.70 (t, J = 7.6 Hz, 1H), 7.59 (t, J = 7.6 Hz, 1H), 7.45 (d, J = 7.8 Hz, 1H), 7.27 (d, J = 8.4 Hz, 2H), 6.80 (d, J = 8.3 Hz, 2H), 4.86 – 4.73 (m, 2H), 4.18 (s, 1H), 3.79 – 3.68 (m, 4H), 2.11 – 1.98 (m, 1H), 1.91 – 1.79 (m, 1H), 1.65 – 1.30 (m, 8H), 0.90 (t, J = 7.3 Hz, 3H).

^{13}C NMR (101 MHz, $CDCl_3$) δ 172.3, 158.6, 137.9, 134.5, 134.2, 130.9, 129.8, 128.1, 125.1, 121.8, 113.6, 59.7, 55.8, 55.3, 47.4, 34.1, 28.1, 22.6, 22.4, 14.1.

HRMS (ESI, m/z): $[M+H]^+$ Calcd. For $C_{22}H_{29}N_2O_4S$: 417.1848; Found: 417.1851.

HPLC (Chiralpak OD-H Column), *i*-PrOH/hexane = 3/97, flow rate = 1.0 mL/min, λ = 210 nm; t_R = 17.1 min (minor), t_R = 20.4 min (major).

$[\alpha]_D^{25}$ = –32.9 (c = 0.45, $CHCl_3$).

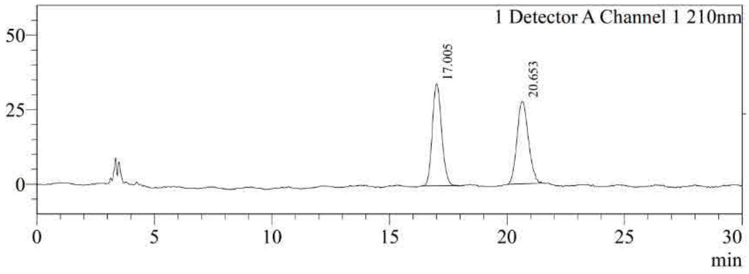

Detector A Channel 1 210nm

Peak#	Ret. Time	USP Width	Area	Height	Area%
1	17.005	0.733	947153	34202	50.321
2	20.653	0.882	935085	27772	49.679
Total			1882238	61974	100.000

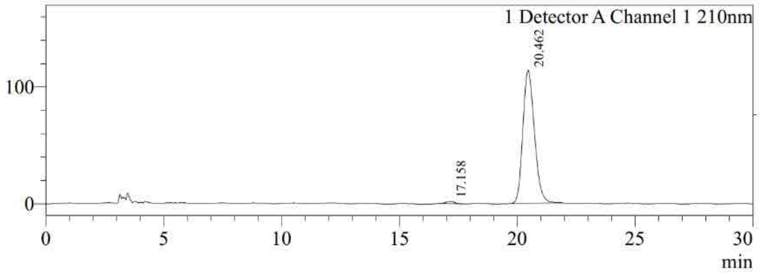

Detector A Channel 1 210nm

Peak#	Ret. Time	USP Width	Area	Height	Area%
1	17.158	0.665	53855	2120	1.369
2	20.462	0.894	3880615	113866	98.631
Total			3934470	115986	100.000

Synthesis of ($R_{C\text{-}1}$, $S_{C\text{-}2}$)-7a

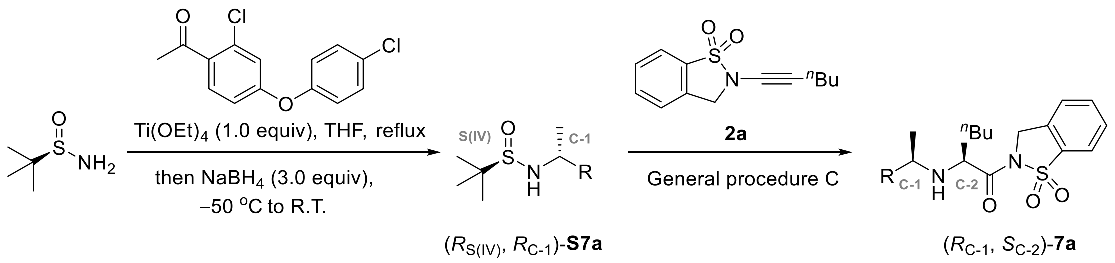

Synthesis of ($R_{S(IV)}$, $R_{C\text{-}1}$)-S7a

Based on the literature procedure (*57*): Under N_2 atmosphere, the mixture of (*R*)-*tert*-butanesulfinamide (3.64 g, 30 mmol, 1.0 equiv), 4-Acetyl-3,4′-dichlordiphenylether (8.43 g, 30 mmol, 1.0 equiv) and $Ti(OEt)_4$ (8.64 g, 30 mmol, 1.0 equiv) in THF was refluxed overnight. After completion, $NaBH_4$ (3.40 g, 90 mmol, 3.0 equiv) was added in batches at −50 °C, and the reaction mixture was warmed to room temperature over 3 hours period (monitored by TLC), followed by quenching with MeOH and saturated brine at 0 °C. The resulting suspension was filtered and washed with ethyl acetate. The filtrate was collected and extracted with ethyl acetate. The combined organic layer was washed with brine and concentrated under vacuum. The crude product was purified through flash chromatography (eluent: petroleum ether/ethyl acetate = 4:1 to 2:1) to afford ($R_{S(IV)}$, $R_{C\text{-}1}$)-**S7a** as white solid (4.70 g, 40% yield, >99:1 d.r.).

¹H NMR (400 MHz, $CDCl_3$) δ 7.37 (d, *J* = 8.7 Hz, 1H), 7.26 – 7.21 (m, 2H), 6.93 – 6.87 (m, 3H), 6.85 (dd, *J* = 8.6, 2.6 Hz, 1H), 4.88 (dt, *J* = 11.2, 5.5 Hz, 1H), 3.63 (d, *J* = 4.6 Hz, 1H), 1.46 (d, *J* = 6.6 Hz, 3H), 1.18 (s, 9H).

¹³C NMR (101 MHz, $CDCl_3$) δ 156.7, 154.8, 136.3, 133.3, 129.9, 129.1, 128.5, 120.6, 119.4, 117.2, 55.7, 50.6, 22.5, 22.0.

HRMS (ESI, m/z): $[M+Na]^+$ Calcd. For $C_{18}H_{21}Cl_2NO_2NaS$: 408.0568; Found: 408.0570.

HPLC (Chiralpak AD-H Column), *i*-PrOH/hexane = 5/95, flow rate = 1.0 mL/min, λ = 210 nm; t_R = 9.3 min (major), t_R = 11.4 min (minor).

$[\alpha]_D^{20}$ = –34.8 (c = 1.0, $CHCl_3$).

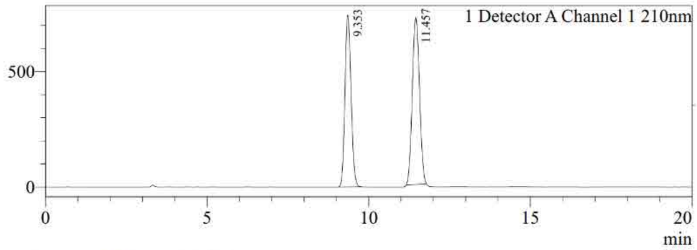

Detector A Channel 1 210nm

Peak#	Ret. Time	USP Width	Area	Height	Area%
1	9.353	0.356	9804570	743375	46.635
2	11.457	0.421	11219289	723382	53.365
Total			21023859	1466757	100.000

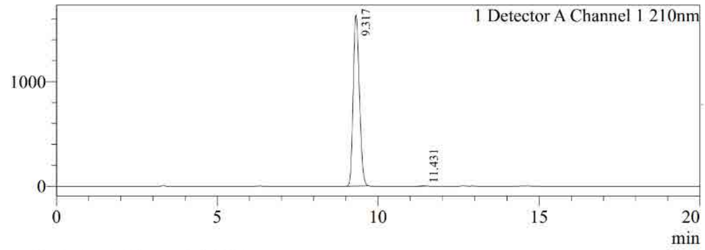

Detector A Channel 1 210nm

Peak#	Ret. Time	USP Width	Area	Height	Area%
1	9.317	0.362	21954491	1635906	99.908
2	11.431	0.252	20157	1875	0.092
Total			21974647	1637781	100.000

(*S*)-2-(((*R*)-1-(2-Chloro-4-(4-chlorophenoxy)phenyl)ethyl)amino)-1-(1,1-dioxidobenzoisothiazol-2(3*H*)-yl)hexan-1-one (7a)

Prepared according to the **GENERAL PROCEDURE C** with ($R_{S(IV)}$, R_{C-1})-**S7a** (115.9 mg, 0.30 mmol, >99:1 d.r.) and **2a** (50.0 mg, 0.20 mmol) as substrates. Column chromatography: silica gel, petroleum ether/ethyl acetate = 200:13. Colorless viscous oil (83.8 mg, 76% yield, 99:1 d.r.).

¹H NMR (400 MHz, $CDCl_3$) δ 7.81 (d, *J* = 7.8 Hz, 1H), 7.71 (t, *J* = 7.6 Hz, 1H), 7.63 – 7.56 (m, 2H), 7.47 (d, *J* = 7.8 Hz, 1H), 7.23 (d, *J* = 8.6 Hz, 2H), 6.93 (d, *J* = 2.4 Hz, 1H), 6.89 (dd, *J* = 9.7, 3.2 Hz, 3H), 4.94 (d, *J* = 15.9 Hz, 1H), 4.87 (d, *J* = 15.9 Hz, 1H), 4.31 (q, *J* = 6.4 Hz, 1H), 4.15 (s, 1H), 2.21 (s, 1H), 1.89 (ddt, *J* = 14.7, 10.4, 4.1 Hz, 1H), 1.59 (ddt, *J* = 12.9, 7.6, 4.4 Hz, 2H), 1.51 – 1.29 (m, 6H), 0.91 (t, *J* = 7.3 Hz, 3H).

¹³C NMR (101 MHz, $CDCl_3$) δ 174.7, 156.1, 155.5, 138.0, 134.5, 134.2, 133.6, 130.9, 129.87, 129.85, 129.2, 128.7, 125.1, 121.9, 120.3, 119.6, 117.6, 59.2, 51.6, 47.5, 33.9, 28.0, 22.6, 21.8, 14.1.

HRMS (ESI, m/z): $[M+H]^+$ Calcd. For $C_{27}H_{29}Cl_2N_2O_4S$: 547.1225; Found: 547.1226.

HPLC (Chiralpak AD-H Column), *i*-PrOH/hexane = 15/85, flow rate = 1.0 mL/min, λ = 210 nm; t_R = 11.4 min (major), t_R = 15.4 min (minor).

[α]$_D^{25}$ = +15.2 (c = 0.5, $CHCl_3$).

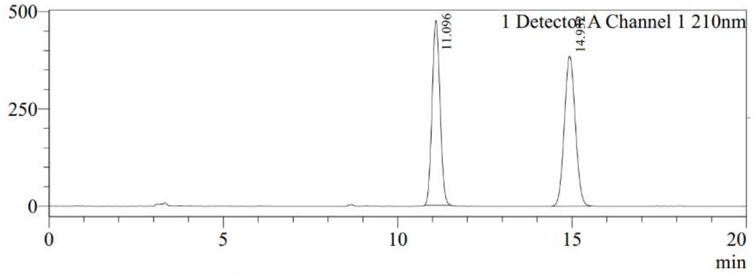

Detector A Channel 1 210nm

Peak#	Ret. Time	USP Width	Area	Height	Area%
1	11.096	0.425	7522100	475399	47.060
2	14.932	0.587	8461926	386301	52.940
Total			15984026	861700	100.000

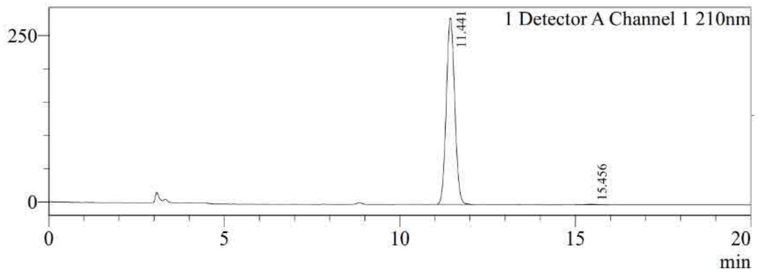

Detector A Channel 1 210nm

Peak#	Ret. Time	USP Width	Area	Height	Area%
1	11.441	0.447	4694502	280556	99.443
2	15.456	0.488	26288	1237	0.557
Total			4720791	281793	100.000

Synthesis of ($S_{C\text{-}1}$, $R_{C\text{-}2}$)-7b

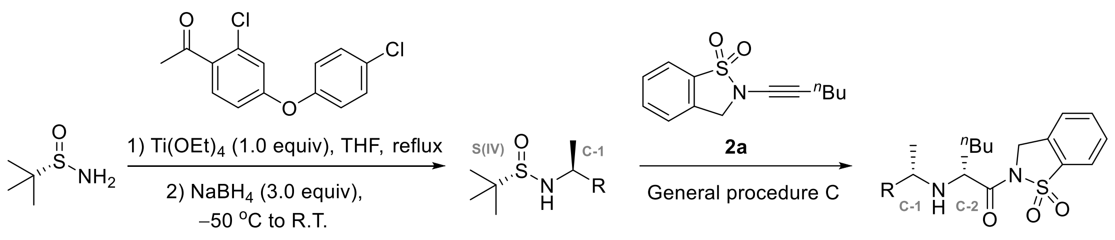

($S_{S(IV)}$, $S_{C\text{-}1}$)-**S7b** ($S_{C\text{-}1}$, $R_{C\text{-}2}$)-**7b**

Synthesis of ($S_{S(IV)}$, $S_{C\text{-}1}$)**-S7b**

Step 1: Under N_2 atmosphere, the mixture of (*S*)-*tert*-butanesulfinamide (3.64 g, 30 mmol, 1.0 equiv), 4-acetyl-3,4′-dichlordiphenylether (8.43 g, 30 mmol, 1.0 equiv) and $Ti(OEt)_4$ (8.64 g, 30 mmol, 1.0 equiv) in THF was refluxed overnight. After completion, the mixture was quenched with ice-water and the resulting suspension was filtered and washed with ethyl acetate. The filtrate was collected and extracted with ethyl acetate. The combined

organic layer was washed with brine and concentrated under vacuum. The crude product was purified through flash chromatography (eluent: petroleum ether/ethyl acetate/ dichloromethane = 20:1:1) to afford imine as white solid (8.73 g, 76% yield), which was used for the next step.

Step 2: Under N_2 atmosphere, the solution of imine (8.73 g, 22.7 mmol, 1.0 equiv) dissolved in THF was cooled at −50 °C and $NaBH_4$ (2.58 g, 68.1 mmol, 3.0 equiv) was added in batches subsequently. The reaction mixture was warmed to room temperature over 3 hours period (monitored by TLC). Once the reaction was completed, MeOH and saturated brine was added in carefully and the mixture was extracted with ethyl acetate. The combined organic layer was washed with brine and concentrated under vacuum. The crude product was purified through flash chromatography (eluent: petroleum ether/ethyl acetate = 4:1 to 2:1) to afford ($S_{S(IV)}$, S_{C-1})-**S7b** as white solid (3.33 g, 38% yield, >99:1 d.r.).

^{1}H NMR (400 MHz, $CDCl_3$) δ 7.40 (d, J = 8.7 Hz, 1H), 7.34 – 7.26 (m, 2H), 6.95 (dd, J = 9.2, 2.5 Hz, 3H), 6.89 (dd, J = 8.6, 2.7 Hz, 1H), 4.93 (qd, J = 6.7, 4.2 Hz, 1H), 3.56 (d, J = 4.5 Hz, 1H), 1.50 (d, J = 6.6 Hz, 3H), 1.22 (s, 8H).

^{13}C NMR (101 MHz, $CDCl_3$) δ 156.9, 154.9, 136.3, 133.4, 130.0, 129.2, 128.6, 120.8, 119.5, 117.3, 55.8, 50.7, 22.6, 22.0.

HRMS (ESI, m/z): $[M+Na]^+$ Calcd. For $C_{18}H_{21}Cl_2NO_2NaS$: 408.0568; Found: 408.0570.

HPLC (Chiralpak AD-H Column), *i*-PrOH/hexane = 5/95, flow rate = 1.0 mL/min, λ = 210 nm; t_R = 9.3 min (minor), t_R = 11.4 min (major).

$[\alpha]_D^{20}$ = +29.2 (c = 1.0, $CHCl_3$).

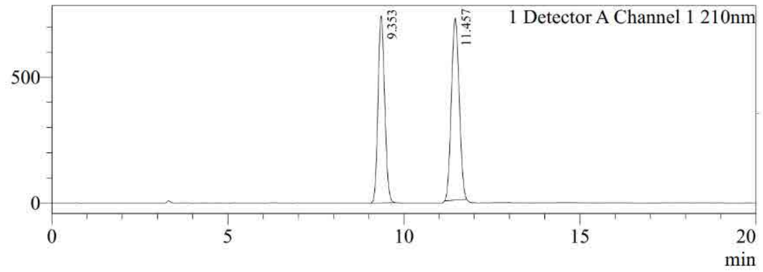

Detector A Channel 1 210nm

Peak#	Ret. Time	USP Width	Area	Height	Area%
1	9.353	0.356	9804570	743375	46.635
2	11.457	0.421	11219289	723382	53.365
Total			21023859	1466757	100.000

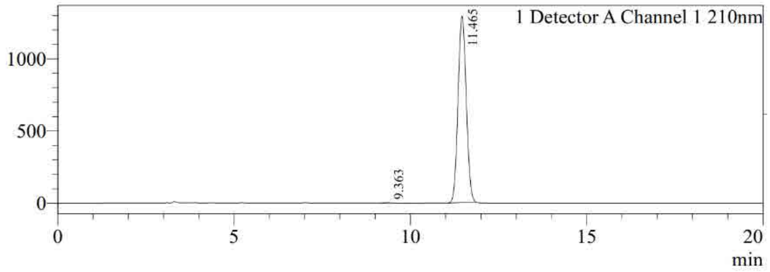

Detector A Channel 1 210nm

Peak#	Ret. Time	USP Width	Area	Height	Area%
1	9.363	0.196	5626	706	0.027
2	11.465	0.436	20978886	1293078	99.973
Total			20984512	1293784	100.000

(*R*)-2-(((*S*)-1-(2-Chloro-4-(4-chlorophenoxy)phenyl)ethyl)amino)-1-(1,1-dioxidobenzoisothiazol-2(3*H*)-

yl)hexan-1-one (7b)

Prepared according to the **GENERAL PROCEDURE C** with ($S_{S(IV)}$, S_{C-1})-**S7b** (115.9 mg, 0.30 mmol, >99:1 d.r.) and **2a** (50.0 mg, 0.20 mmol) as substrates. Column chromatography: silica gel, petroleum ether/ethyl acetate = 200:13. Colorless viscous oil (81.2 mg, 74% yield, >99:1 d.r.).

^{1}H NMR (400 MHz, $CDCl_3$) δ 7.81 (d, J = 7.9 Hz, 1H), 7.70 (t, J = 7.6 Hz, 1H), 7.63 – 7.55 (m, 2H), 7.46 (d, J = 7.8 Hz, 1H), 7.24 – 7.19 (m, 2H), 6.93 (d, J = 2.5 Hz, 1H), 6.88 (dd, J = 9.6, 2.6 Hz, 3H), 4.93 (d, J = 15.9 Hz, 1H), 4.87 (d, J = 15.9 Hz, 1H), 4.30 (q, J = 6.4 Hz, 1H), 4.15 (s, 1H), 2.10 (s, 1H), 1.88 (ddt, J = 17.6, 9.7, 3.6 Hz, 1H), 1.64 – 1.52 (m, 2H), 1.52 – 1.43 (m, 1H), 1.40 – 1.31 (m, 5H), 0.91 (t, J = 7.3 Hz, 3H).

^{13}C NMR (101 MHz, $CDCl_3$) δ 174.9, 155.9, 155.5, 138.2, 134.4, 134.2, 133.5, 130.8, 129.8, 129.1, 128.6, 125.1, 121.8, 120.2, 119.6, 117.6, 59.1, 51.5, 47.4, 33.9, 28.0, 22.5, 21.8, 14.1.

HRMS (ESI, m/z): $[M+H]^+$ Calcd. For $C_{27}H_{29}Cl_2N_2O_4S$: 547.1225; Found: 547.1228.

HPLC (Chiralpak AD-H Column), *i*-PrOH/hexane = 15/85, flow rate = 1.0 mL/min, λ = 210 nm; t_R = 11.4 min (minor), t_R = 15.4 min (major).

$[\alpha]_D^{25}$ = –15.6 (c = 0.5, $CHCl_3$).

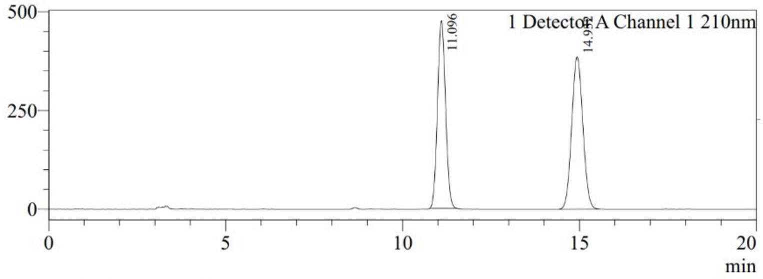

Detector A Channel 1 210nm

Peak#	Ret. Time	USP Width	Area	Height	Area%
1	11.096	0.425	7522100	475399	47.060
2	14.932	0.587	8461926	386301	52.940
Total			15984026	861700	100.000

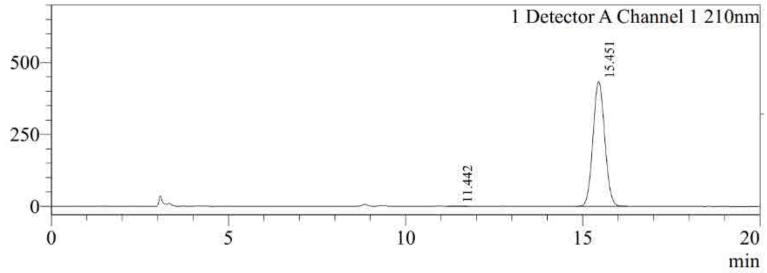

Detector A Channel 1 210nm

Peak#	Ret. Time	USP Width	Area	Height	Area%
1	11.442	0.374	15414	1010	0.153
2	15.451	0.617	10060146	434311	99.847
Total			10075560	435320	100.000

Synthesis of ($R_{C\text{-}1}$, $S_{C\text{-}2}$)-8a

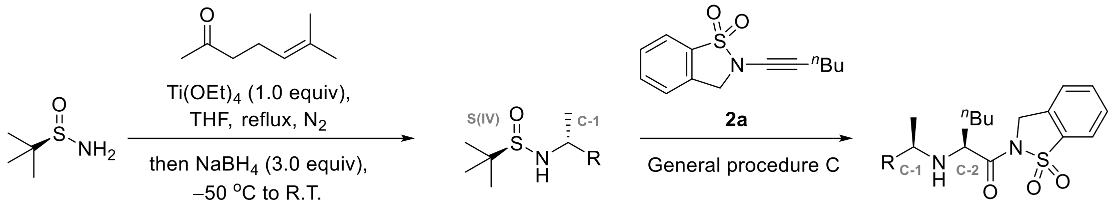

($R_{S(IV)}$, $R_{C\text{-}1}$)-**S8a** ($R_{C\text{-}1}$, $S_{C\text{-}2}$)-**8a**

Synthesis of ($R_{S(IV)}$, $R_{C\text{-}1}$)-**S8a**

Based on the literature procedure (*57*): Under N_2 atmosphere, the mixture of (*R*)-*tert*-butanesulfinamide (3.64 g, 30 mmol, 1.0 equiv), 6-methylhept-5-en-2-one (3.78 g, 30 mmol, 1.0 equiv) and $Ti(OEt)_4$ (8.64 g, 30 mmol, 1.0 equiv) in THF was refluxed overnight. After completion, $NaBH_4$ (3.40 g, 90 mmol, 3.0 equiv) was added in batches at −50 °C, and the reaction mixture was warmed to room temperature over 3 h period (monitored by TLC), followed by quenching with MeOH and saturated brine at 0 °C. The resulting suspension was filtered and washed with ethyl acetate. The filtrate was collected and extracted with ethyl acetate. The combined organic layer was washed with brine and concentrated under vacuum. The crude product was purified through flash chromatography (eluent: petroleum ether/ethyl acetate/dichloromethane = 5:1:1) to afford ($R_{S(IV)}$, $R_{C\text{-}1}$)-**S8a** as colorless oil (4.90 g, 71% yield, >99:1 d.r.).

^{1}H NMR (400 MHz, $CDCl_3$) δ 4.91 (t, *J* = 7.3 Hz, 1H), 3.23 – 3.12 (m, 1H), 3.08 (d, *J* = 4.6 Hz, 1H), 1.86 (q, *J* = 7.5 Hz, 2H), 1.47 (s, 3H), 1.41 (s, 3H), 1.44 – 1.26 (m, 2H), 0.98 (s, 9H), 0.96 (s, 3H).

^{13}C NMR (101 MHz, $CDCl_3$) δ 131.7, 123.5, 54.7, 50.7, 37.9, 25.4, 24.2, 22.2, 21.2, 17.4.

HRMS (ESI, m/z): $[M+Na]^+$ Calcd. For $C_{12}H_{25}NONaS$: 254.1555; Found: 254.1558.

HPLC (Chiralpak OD-H Column), *i*-PrOH/hexane = 5/95, flow rate = 1.0 mL/min, λ = 210 nm; t_R = 4.6 min (minor), t_R = 4.9 min (major).

$[\alpha]_D^{20}$ = −85.6 (c = 1.0, $CHCl_3$).

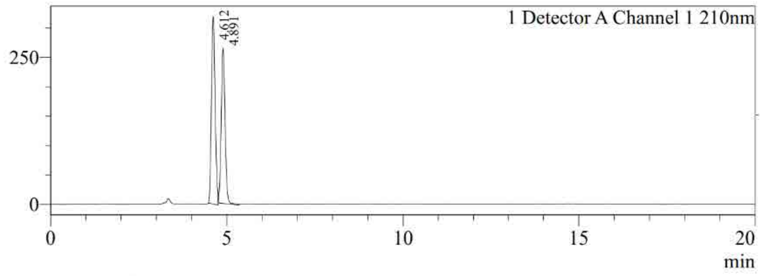

Detector A Channel 1 210nm

Peak#	Ret. Time	USP Width	Area	Height	Area%
1	4.612	0.204	2322214	318708	53.807
2	4.891	0.210	1993629	261860	46.193
Total			4315842	580568	100.000

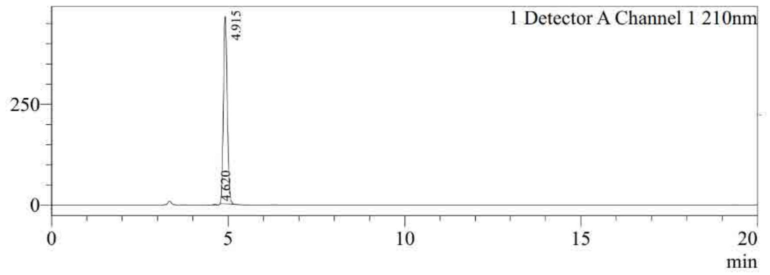

Detector A Channel 1 210nm

Peak#	Ret. Time	USP Width	Area	Height	Area%
1	4.620	0.167	5293	933	0.151
2	4.915	0.208	3501156	463588	99.849
Total			3506449	464520	100.000

(*S*)-1-(1,1-Dioxidobenzoisothiazol-2(3*H*)-yl)-2-(((*R*)-6-methylhept-5-en-2-yl)amino)hexan-1-one (8a)

Prepared according to the **GENERAL PROCEDURE C** with ($R_{S(IV)}$, $R_{C\text{-}1}$)-**S8a** (69.4 mg, 0.30 mmol, >99:1 d.r.) and **2a** (50.0 mg, 0.20 mmol) as substrates. Column chromatography: silica gel, petroleum ether/ethyl acetate = 200:13. Colorless viscous oil (57.5 mg, 73% yield, >99:1 d.r.).

^{1}H NMR (400 MHz, $CDCl_3$) δ 7.82 (d, J = 7.9 Hz, 1H), 7.71 (t, J = 7.6 Hz, 1H), 7.59 (t, J = 7.6 Hz, 1H), 7.48 (d, J = 7.8 Hz, 1H), 5.08 (t, J = 7.4 Hz, 1H), 5.02 (d, J = 15.8 Hz, 1H), 4.90 (d, J = 15.9 Hz, 1H), 4.20 – 4.10 (m, 1H), 2.56 (h, J = 6.4 Hz, 1H), 2.04 – 1.91 (m, 3H), 1.82 (tt, J = 10.1, 5.9 Hz, 1H), 1.65 (s, 3H), 1.59 (s, 3H), 1.57 – 1.49 (m, 2H), 1.48 – 1.27 (m, 5H), 1.07 (d, J = 6.1 Hz, 3H), 0.90 (t, J = 7.3 Hz, 3H).

^{13}C NMR (101 MHz, $CDCl_3$) δ 176.0, 134.6, 134.2, 131.6, 131.0, 129.8, 125.1, 124.4, 121.9, 59.2, 51.5, 47.5, 38.1, 34.5, 28.1, 25.8, 24.8, 22.7, 20.2, 17.8, 14.1.

HRMS (ESI, m/z): $[M+H]^+$ Calcd. For $C_{21}H_{33}N_2O_3S$: 393.2212; Found: 393.2214.

HPLC (Chiralpak OD-H Column), *i*-PrOH/hexane = 1/99, flow rate = 1.0 mL/min, λ = 254 nm; t_R = 14.0 min (major), t_R = 15.5 min (minor).

$[\alpha]_D^{25}$ = –6.00 (c = 0.5, $CHCl_3$).

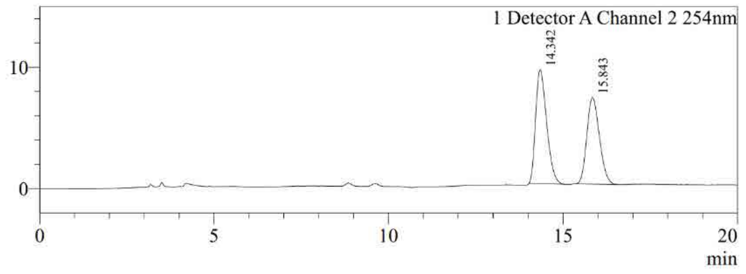

Detector A Channel 2 254nm

Peak#	Ret. Time	USP Width	Area	Height	Area%
1	14.342	0.587	206008	9360	53.448
2	15.843	0.674	179428	7125	46.552
Total			385436	16485	100.000

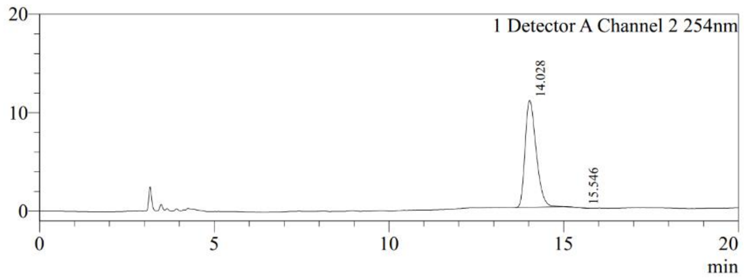

Detector A Channel 2 254nm

Peak#	Ret. Time	USP Width	Area	Height	Area%
1	14.028	0.581	239392	10863	99.709
2	15.546	0.801	698	11	0.291
Total			240090	10874	100.000

Synthesis of ($S_{C\text{-}1}$, $R_{C\text{-}2}$)-8b

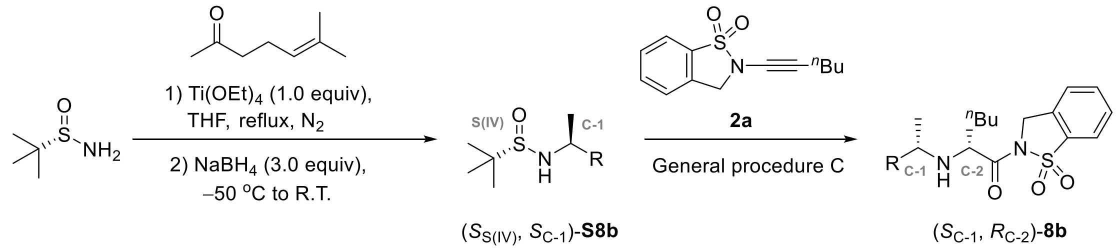

Synthesis of ($S_{S(IV)}$, $S_{C\text{-}1}$)-**S8b**

Step 1: Under N_2 atmosphere, the mixture of (*S*)-*tert*-butanesulfinamide (3.64 g, 30 mmol, 1.0 equiv), 6-methylhept-5-en-2-one (3.78 g, 30 mmol, 1.0 equiv) and $Ti(OEt)_4$ (8.64 g, 30 mmol, 1.0 equiv) in THF was refluxed overnight. After completion, the mixture was quenched with ice-water and the resulting suspension was filtered and washed with ethyl acetate. The filtrate was collected and extracted with ethyl acetate. The combined organic layer was washed with brine and concentrated under vacuum. The crude product was purified through flash chromatography (eluent: petroleum ether/ethyl acetate/ dichloromethane = 15:1:1) to afford imine as colorless oil (4.17 g, 60% yield), which was used for the next step.

Step 2: Under N_2 atmosphere, the solution of imine (4.17 g, 18.2 mmol, 1.0 equiv) dissolved in THF was cooled at −50 °C and $NaBH_4$ (2.06 g, 54.6 mmol, 3.0 equiv) was added in batches subsequently. The reaction mixture was warmed to room temperature over 3 h period (monitored by TLC). Once the reaction is completed, MeOH and saturated brine was added in carefully and the mixture was extracted with ethyl acetate. The combined organic layer was washed with brine and concentrated under vacuum. The crude product was purified through flash chromatography (eluent: petroleum ether/ethyl acetate/ dichloromethane = 5:1:1) to afford ($S_{S(IV)}$, $S_{C\text{-}1}$)-**S8b** as colorless oil (1.44 g, 34% yield, >99:1 d.r.).

^{1}H NMR (400 MHz, $CDCl_3$) δ 4.98 (t, *J* = 7.1 Hz, 1H), 3.25 (pd, *J* = 6.5, 4.8 Hz, 1H), 3.10 (d, *J* = 4.5 Hz, 1H), 1.94 (q, *J* = 7.5 Hz, 2H), 1.55 (s, 3H), 1.49 (s, 3H), 1.49 – 1.36 (m, 2H), 1.09 – 1.02 (m, 12H).

^{13}C NMR (101 MHz, $CDCl_3$) δ 132.0, 123.6, 54.9, 50.8, 38.1, 25.6, 24.4, 22.4, 21.3, 17.6.

HRMS (ESI, m/z): $[M+Na]^+$ Calcd. For $C_{12}H_{25}NONaS$: 254.1555; Found: 254.1558.

HPLC (Chiralpak OD-H Column), *i*-PrOH/hexane = 5/95, flow rate = 1.0 mL/min, λ = 210 nm; t_R = 4.6 min (major), t_R = 4.8 min (minor).

[α]$_D^{20}$ = +89.2 (c = 1.0, $CHCl_3$).

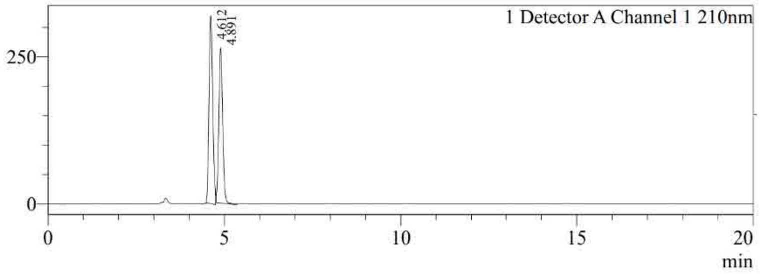

Detector A Channel 1 210nm

Peak#	Ret. Time	USP Width	Area	Height	Area%
1	4.612	0.204	2322214	318708	53.807
2	4.891	0.210	1993629	261860	46.193
Total			4315842	580568	100.000

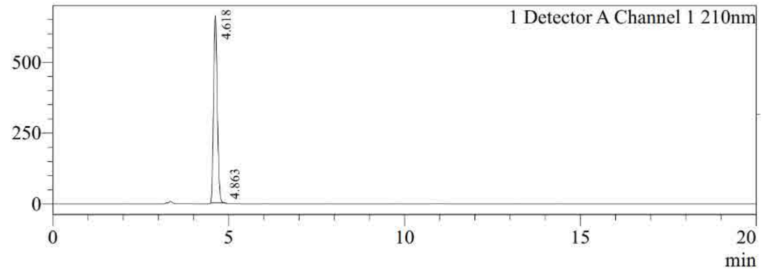

Detector A Channel 1 210nm

Peak#	Ret. Time	USP Width	Area	Height	Area%
1	4.618	0.206	4847478	657473	99.998
2	4.863	0.105	90	188	0.002
Total			4847567	657661	100.000

(*R*)-1-(1,1-Dioxidobenzoisothiazol-2(3*H*)-yl)-2-(((*S*)-6-methylhept-5-en-2-yl)amino)hexan-1-one (8b)

Prepared according to the **GENERAL PROCEDURE C** with ($S_{S(IV)}$, $S_{C\text{-}1}$)-**S8b** (69.4 mg, 0.30 mmol, >99:1 d.r.) and **2a** (50.0 mg, 0.20 mmol) as substrates. Column chromatography: silica gel, petroleum ether/ethyl acetate = 200:13. Colorless viscous oil (57.1 mg, 73% yield, >99:1 d.r.).

^{1}H NMR (400 MHz, $CDCl_3$) δ 7.82 (d, *J* = 7.9 Hz, 1H), 7.71 (t, *J* = 7.6 Hz, 1H), 7.60 (t, *J* = 7.7 Hz, 1H), 7.48 (d, *J* = 7.8 Hz, 1H), 5.08 (t, *J* = 7.2 Hz, 1H), 5.03 (d, *J* = 15.9 Hz, 1H), 4.90 (d, *J* = 15.9 Hz, 1H), 4.15 (t, *J* = 6.4 Hz, 1H), 2.56 (h, *J* = 6.3 Hz, 1H), 2.00 (q, *J* = 8.1 Hz, 2H), 1.93 – 1.78 (m, 2H), 1.65 (s, 3H), 1.59 (s, 3H), 1.58 – 1.49 (m, 2H), 1.48 – 1.30 (m, 5H), 1.07 (d, *J* = 6.1 Hz, 3H), 0.90 (t, *J* = 7.2 Hz, 3H).

^{13}C NMR (101 MHz, $CDCl_3$) δ 176.1, 134.6, 134.2, 131.6, 131.0, 129.8, 125.1, 124.4, 121.9, 59.2, 51.5, 47.5, 38.1, 34.5, 28.1, 25.8, 24.8, 22.7, 20.2, 17.8, 14.1.

HRMS (ESI, m/z): $[M+H]^+$ Calcd. For $C_{21}H_{33}N_2O_3S$: 393.2212; Found: 393.2215.

HPLC (Chiralpak OD-H Column), *i*-PrOH/hexane = 1/99, flow rate = 1.0 mL/min, λ = 254 nm; t_R = 13.9 min (minor), t_R = 15.6 min (major).

[α]$_D^{25}$ = +4.40 (c = 0.5, $CHCl_3$).

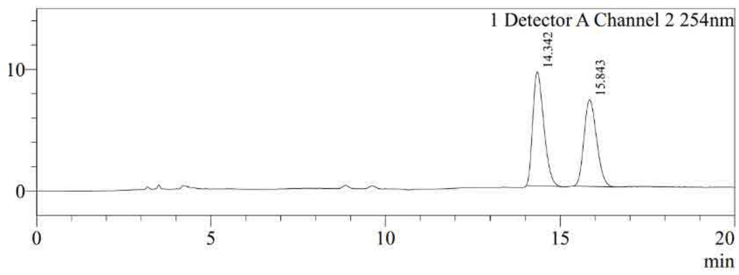

Detector A Channel 2 254nm

Peak#	Ret. Time	USP Width	Area	Height	Area%
1	14.342	0.587	206008	9360	53.448
2	15.843	0.674	179428	7125	46.552
Total			385436	16485	100.000

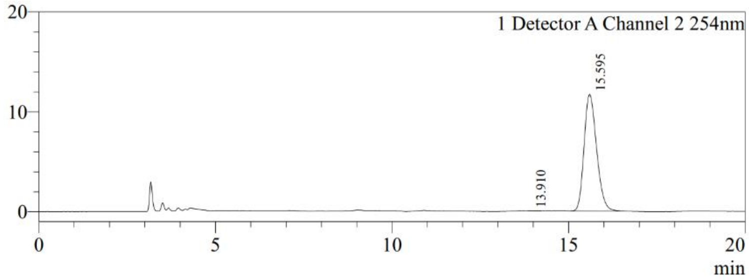

Detector A Channel 2 254nm

Peak#	Ret. Time	USP Width	Area	Height	Area%
1	13.910	1.295	248	3	0.086
2	15.595	0.650	287518	11677	99.914
Total			287765	11679	100.000

Synthesis of ($R_{C\text{-}1}$, $S_{C\text{-}2}$)-9a

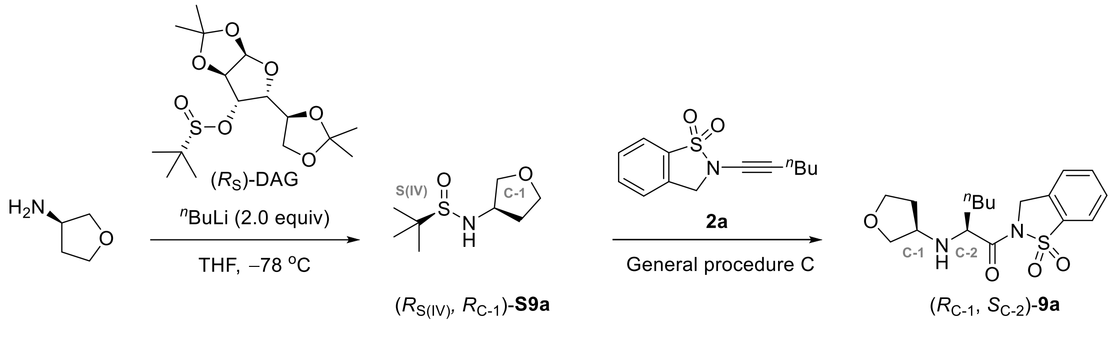

Synthesis of ($R_{S(IV)}$, $R_{C\text{-}1}$)-S9a

Under N_2 atmosphere, the solution of (*R*)-tetrahydrofuran-3-amine (1.00 g, 11.5 mmol, 2.2 equiv) in THF was cooled at −78 °C, followed by addition of ^{n}BuLi (4.2 mL, 2.5 M, 2.0 equiv). The resulted mixture was stirred for about 45 minutes and then the solution of (R_S)-diacetone-*D*-glucose *tert*-butanesulfinate ((R_S)-DAG) (*70*) (1.90 g, 5.22 mmol, 1.0 equiv) in THF was added dropwise. The reaction solution was stirred about 20 minutes and then quenched with saturated aqueous NH_4Cl, extracted with ethyl acetate. The combined organic layer was washed with brine and concentrated under vacuum. The crude product was purified through flash chromatography (eluent: petroleum ether/ethyl acetate = 3:1) to afford ($R_{S(IV)}$, $R_{C\text{-}1}$)-**S9a** as white solid (318 mg, 32% yield, 99:1 d.r.).

¹H NMR (400 MHz, $CDCl_3$) δ 4.03 – 3.97 (m, 1H), 3.91 – 3.82 (m, 2H), 3.79 – 3.74 (m, 1H), 3.67 (dd, J = 9.6, 3.2 Hz, 1H), 3.31 (d, J = 5.5 Hz, 1H), 2.14 (dq, J = 14.8, 7.6 Hz, 1H), 1.91 (ddt, J = 12.9, 7.9, 4.2 Hz, 1H), 1.17 (s, 9H).

¹³C NMR (101 MHz, $CDCl_3$) δ 74.4, 66.8, 55.8, 55.6, 33.7, 22.6.

HRMS (ESI, m/z): $[M+Na]^+$ Calcd. For $C_8H_{17}NO_2NaS$: 214.0878; Found: 214.0879.

HPLC (Chiralpak OD-H Column), *i*-PrOH/hexane = 5/95, flow rate = 1.0 mL/min, λ = 210 nm; t_R = 12.6 min (minor), t_R = 18.9 min (major).

$[\alpha]_D^{20}$ = –84.4 (c = 1.0, $CHCl_3$).

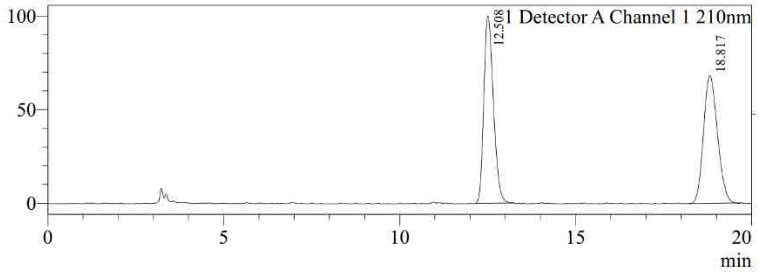

Detector A Channel 1 210nm

Peak#	Ret. Time	USP Width	Area	Height	Area%
1	12.508	0.507	1898553	100050	50.209
2	18.817	0.740	1882734	68085	49.791
Total			3781286	168135	100.000

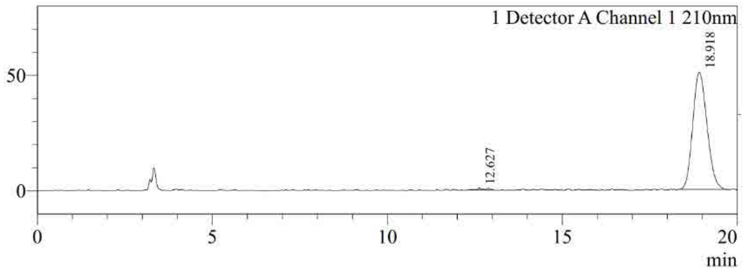

Detector A Channel 1 210nm

Peak#	Ret. Time	USP Width	Area	Height	Area%
1	12.627	0.171	14405	813	0.993
2	18.918	0.755	1435946	50786	99.007
Total			1450351	51599	100.000

(*S*)-1-(1,1-Dioxidobenzoisothiazol-2(3*H*)-yl)-2-(((*R*)-tetrahydrofuran-3-yl)amino)hexan-1-one (9a)

Prepared according to the **GENERAL PROCEDURE C** with ($R_{S(IV)}$, $R_{C\text{-}1}$)-**S9a** (57.4 mg, 0.30 mmol, 99:1 d.r.) and **2a** (50.0 mg, 0.20 mmol) as substrates. Column chromatography: silica gel, petroleum ether/ethyl acetate/dichloromethane = 4:1:1. White solid (61.1 mg, 86% yield, 96:4 d.r.).

¹H NMR (400 MHz, $CDCl_3$) δ 7.80 (d, J = 7.9 Hz, 1H), 7.70 (t, J = 7.6 Hz, 1H), 7.59 (t, J = 7.6 Hz, 1H), 7.48 (d, J = 7.8 Hz, 1H), 5.03 (d, J = 15.9 Hz, 1H), 4.89 (d, J = 15.9 Hz, 1H), 4.09 (dd, J = 8.3, 4.3 Hz, 1H), 3.90 (q, J = 7.5 Hz, 1H), 3.81 (dd, J = 8.9, 5.8 Hz, 1H), 3.75 (td, J = 8.0, 5.4 Hz, 1H), 3.47 (dd, J = 8.9, 3.9 Hz, 1H), 3.34 (ddd, J = 7.9, 6.3, 4.0 Hz, 1H), 2.08 – 1.97 (m, 2H), 1.87 – 1.75 (m, 2H), 1.62 – 1.47 (m, 2H), 1.46 – 1.28 (m, 3H), 0.88 (t, J = 7.2 Hz, 3H).

^{13}C NMR (101 MHz, $CDCl_3$) δ 175.7, 134.34, 134.28, 130.8, 129.9, 125.1, 121.8, 74.1, 67.4, 60.4, 56.9, 47.4, 34.1, 33.1, 28.1, 22.5, 14.0.

HRMS (ESI, m/z): $[M+H]^+$ Calcd. For $C_{17}H_{25}N_2O_4S$: 353.1535; Found: 353.1535.

HPLC (Chiralpak AD-H Column), *i*-PrOH/hexane = 30/70, flow rate = 1.0 mL/min, λ = 210 nm; t_R = 8.1 min (major), t_R = 8.8 min (minor).

$[\alpha]_D^{25}$ = –20.0 (c = 0.9, $CHCl_3$).

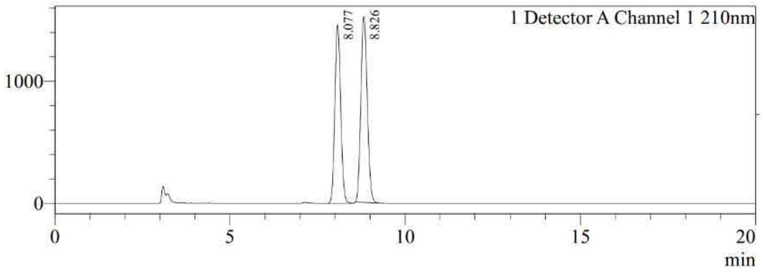

Detector A Channel 1 210nm

Peak#	Ret. Time	USP Width	Area	Height	Area%
1	8.077	0.313	16922247	1460318	46.584
2	8.826	0.345	19404402	1519937	53.416
Total			36326649	2980255	100.000

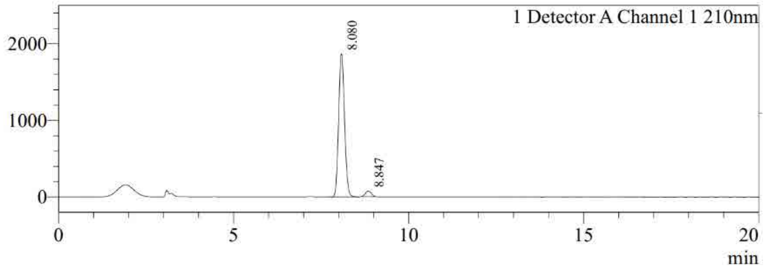

Detector A Channel 1 210nm

Peak#	Ret. Time	USP Width	Area	Height	Area%
1	8.080	0.315	21954018	1867945	96.121
2	8.847	0.328	886001	74403	3.879
Total			22840019	1942348	100.000

Synthesis of (*R*C-1, ±)-9b

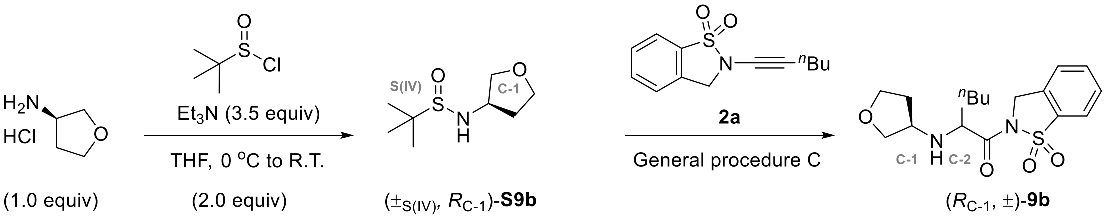

Synthesis of (±S(IV), *R*C-1)-**S9b**

Under N_2 atmosphere, the mixture of (*R*)-tetrahydrofuran-3-amine hydrogen chloride (1.23 g, 10 mmol, 1.0 equiv), Et_3N (3.54 g, 35 mmol, 3.5 equiv) in THF was cooled at 0 °C and then 2-methylpropane-2-sulfinic chloride (2.0 equiv) dissolved in THF was added slowly. The resulted mixture was stirred at room temperature overnight and

then quenched with saturated aqueous NH_4Cl, extracted with ethyl acetate. The combined organic layer was washed with brine and concentrated under vacuum. The crude product was purified through flash chromatography (eluent: petroleum ether/ethyl acetate = 3:1) to afford (±, R_{C-1})-**S9b** as colorless oil (452 mg, 23%, 50:50 d.r.).

^{1}H NMR (400 MHz, $CDCl_3$) δ 3.93 – 3.85 (m, 1H), 3.85 – 3.60 (m, 3H), 3.57 (ddd, *J* = 9.4, 6.2, 3.4 Hz, 1H), 3.45 (dd, *J* = 18.9, 6.0 Hz, 1H), 2.08 (ddq, *J* = 35.3, 13.2, 7.4 Hz, 1H), 1.82 (dtd, *J* = 11.7, 8.2, 7.5, 4.4 Hz, 1H), 1.07 (d, *J* = 2.9 Hz, 9H).

^{13}C NMR (101 MHz, $CDCl_3$) δ 74.1, 73.3, 66.8, 66.6, 55.8, 55.60, 55.58, 55.4, 34.4, 33.4, 22.4, 22.3.

(*R*)-1-(1,1-Dioxidobenzoisothiazol-2(3*H*)-yl)-2-((tetrahydrofuran-3-yl)amino)-2l3-hexan-1-one (9b)

Prepared according to the **GENERAL PROCEDURE C** with (±$_{S(IV)}$, R_{C-1})-**S9b** (57.4 mg, 0.30 mmol, 50:50 d.r.) and **2a** (50.0 mg, 0.20 mmol) as substrates. Column chromatography: silica gel, petroleum ether/ethyl acetate/dichloromethane = 4:1:1. White solid (59.7 mg, 85% yield, 47:53 d.r.).

^{1}H NMR (400 MHz, $CDCl_3$) δ 7.81 (d, *J* = 7.9 Hz, 1H), 7.71 (t, *J* = 7.6 Hz, 1H), 7.59 (t, *J* = 7.6 Hz, 1H), 7.48 (d, *J* = 7.8 Hz, 1H), 5.03 (d, *J* = 15.9 Hz, 1H), 4.89 (d, *J* = 15.9 Hz, 1H), 4.09 (dd, *J* = 8.1, 4.3 Hz, 0.5H), 4.00 (dd, *J* = 8.0, 4.7 Hz, 0.5H), 3.90 (q, *J* = 7.5 Hz, 1H), 3.83 (dt, *J* = 8.9, 5.9 Hz, 1H), 3.73 (dtd, *J* = 19.2, 8.0, 5.7 Hz, 1H), 3.61 (dd, *J* = 8.9, 4.1 Hz, 0.5H), 3.47 (dd, *J* = 8.9, 4.0 Hz, 0.5H), 3.35 (ddd, *J* = 9.6, 7.3, 4.1 Hz, 1H), 2.06 (dt, *J* = 11.9, 6.7 Hz, 1H), 2.00 (s, 1H), 1.80 (tddd, *J* = 12.7, 10.5, 6.9, 3.6 Hz, 1.5H), 1.69 – 1.60 (m, 0.5H), 1.59 – 1.48 (m, 2H), 1.41 (dddd, *J* = 14.5, 11.0, 7.8, 4.1 Hz, 1H), 1.33 (dtd, *J* = 13.2, 6.2, 5.8, 2.8 Hz, 2H), 0.88 (td, *J* = 7.2, 2.3 Hz, 3H).

Synthesis of (R_{C-1}, R_{C-2})-10a

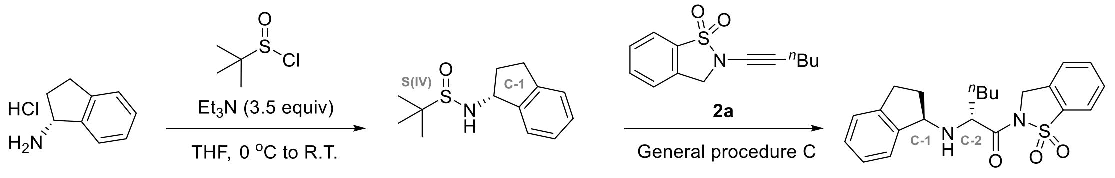

($S_{S(IV)}$, R_{C-1})-**S10a** and ($R_{S(IV)}$, R_{C-1})-**S10b** (R_{C-1}, R_{C-2})-**10a**

Synthesis of ($S_{S(IV)}$, R_{C-1})-S10a

Under N_2 atmosphere, the mixture of (*R*)-2,3-dihydro-1*H*-inden-1-amine hydrochloride (1.69 g, 10 mmol, 1.0 equiv) and Et_3N (3.54 g, 35 mmol, 3.5 equiv) in THF was cooled at 0 °C and then 2-methylpropane-2-sulfinic chloride (2.0 equiv) dissolved in THF was added slowly. The resulted mixture was stirred at room temperature overnight and then quenched with saturated aqueous NH_4Cl, extracted with ethyl acetate. The combined organic layer was washed with brine and concentrated under vacuum. The crude product was purified through flash chromatography (eluent: petroleum ether/ethyl acetate = 8:1) to afford ($S_{S(IV)}$, R_{C-1})-**S10a** as white solid (409 mg, 17% yield, >99:1 d.r.) and ($R_{S(IV)}$, R_{C-1})-**S10b** as white solid (609 mg, 25% yield, >99:1 d.r.). These two compounds were used for the preparation of **10a** and **10b** respectively.

^{1}H NMR (400 MHz, $CDCl_3$) δ 7.37 – 7.31 (m, 1H), 7.31 – 7.18 (m, 3H), 4.82 (dt, *J* = 10.0, 7.5 Hz, 1H), 3.36 (d, *J* = 9.8 Hz, 1H), 2.99 (ddd, *J* = 15.9, 8.7, 3.4 Hz, 1H), 2.84 (dt, *J* = 15.9, 8.1 Hz, 1H), 2.71 (dtd, *J* = 13.1, 7.6, 3.4 Hz, 1H), 1.98 (dq, *J* = 13.1, 8.3 Hz, 1H), 1.26 (s, 9H).

^{13}C NMR (101 MHz, $CDCl_3$) δ 143.9, 143.1, 128.2, 126.7, 125.0, 124.5, 62.5, 56.1, 36.7, 30.4, 22.9.

HRMS (ESI, m/z): $[M+Na]^+$ Calcd. For $C_{13}H_{19}NONaS$: 260.1085; Found: 260.1086.

HPLC (Chiralpak OD-H Column), *i*-PrOH/hexane = 5/95, flow rate = 1.0 mL/min, λ = 210 nm; t_R = 7.1 min (major), t_R = 9.0 min (minor).

$[\alpha]_D^{20}$ = –25.8 (c = 1.0, $CHCl_3$).

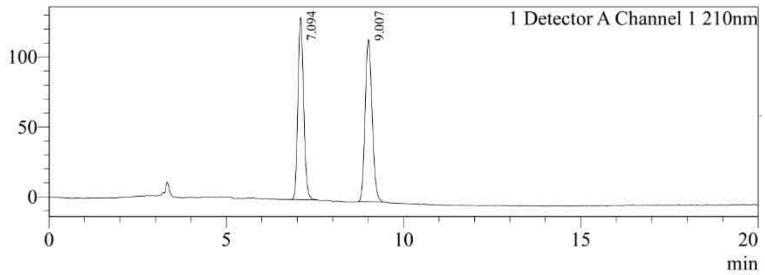

Detector A Channel 1 210nm

Peak#	Ret. Time	USP Width	Area	Height	Area%
1	7.094	0.302	1465607	130320	47.972
2	9.007	0.367	1589547	116018	52.028
Total			3055155	246338	100.000

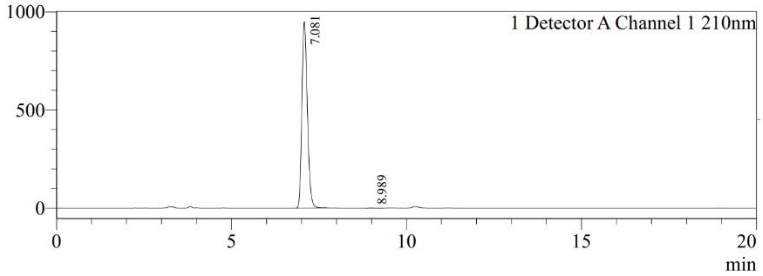

Detector A Channel 1 210nm

Peak#	Ret. Time	USP Width	Area	Height	Area%
1	7.081	0.291	10360725	949197	99.918
2	8.989	0.284	8519	787	0.082
Total			10369245	949984	100.000

(*R*)-2-(((*R*)-2,3-Dihydro-1*H*-inden-1-yl)amino)-1-(1,1-dioxidobenzoisothiazol-2(3*H*)-yl)hexan-1-one (10a)

Prepared according to the **GENERAL PROCEDURE C** with ($S_{S(IV)}$, $R_{C\text{-}1}$)-**S10a** (71.2 mg, 0.30 mmol, >99:1 d.r.) and **2a** (50.0 mg, 0.20 mmol) as substrates. Column chromatography: silica gel, petroleum ether/ethyl acetate/dichloromethane = 10:1:1. Colorless viscous oil (63.4 mg, 79% yield, 98:2 d.r.).

^{1}H NMR (400 MHz, $CDCl_3$) δ 7.84 (d, *J* = 7.8 Hz, 1H), 7.71 (t, *J* = 7.6 Hz, 1H), 7.60 (t, *J* = 7.6 Hz, 1H), 7.49 (d, *J* = 7.6 Hz, 2H), 7.24 – 7.14 (m, 3H), 5.01 (d, *J* = 15.8 Hz, 1H), 4.93 (d, *J* = 15.9 Hz, 1H), 4.31 (t, *J* = 7.2 Hz, 1H), 4.28 – 4.22 (m, 1H), 2.93 (ddd, *J* = 15.7, 8.6, 3.4 Hz, 1H), 2.74 (dt, *J* = 16.0, 8.2 Hz, 1H), 2.39 (dtd, *J* = 11.2, 7.4, 3.4 Hz, 1H), 2.05 (s, 1H), 1.89 (ddt, *J* = 13.7, 9.1, 4.2 Hz, 1H), 1.78 – 1.58 (m, 3H), 1.58 – 1.47 (m, 1H), 1.40 (tdd, *J* = 14.1, 8.4, 5.6 Hz, 2H), 0.94 (t, *J* = 7.3 Hz, 3H).

^{13}C NMR (101 MHz, $CDCl_3$) δ 176.4, 145.8, 143.3, 134.6, 134.2, 130.9, 129.8, 127.3, 126.3, 125.1, 124.5, 124.5, 121.9, 63.4, 61.2, 47.6, 35.4, 34.6, 30.5, 28.2, 22.6, 14.1.

HRMS (ESI, m/z): $[M+H]^+$ Calcd. For $C_{22}H_{27}N_2O_3S$: 399.1742; Found: 399.1743.

HPLC (Chiralpak OD-H Column), *i*-PrOH/hexane = 2/98, flow rate = 1.0 mL/min, λ = 210 nm; t_R = 18.7 min (minor), t_R = 21.8 min (major).

$[\alpha]_D^{25}$ = +5.60 (c = 0.5, $CHCl_3$).

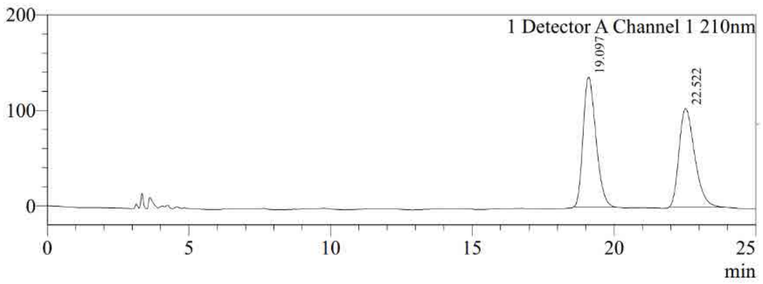

Detector A Channel 1 210nm

Peak#	Ret. Time	USP Width	Area	Height	Area%
1	19.097	0.835	4298098	136242	51.832
2	22.522	1.011	3994304	103561	48.168
Total			8292402	239803	100.000

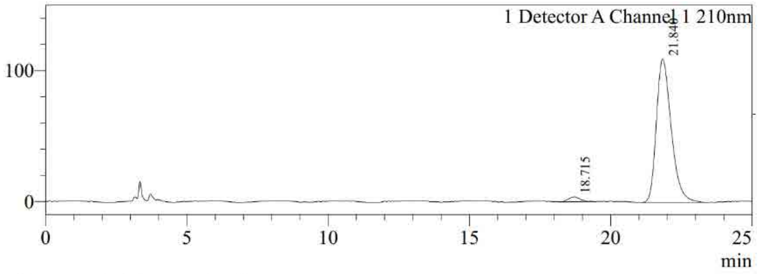

Detector A Channel 1 210nm

Peak#	Ret. Time	USP Width	Area	Height	Area%
1	18.715	0.684	101008	3682	2.459
2	21.840	0.952	4006279	109480	97.541
Total			4107287	113162	100.000

Synthesis of (R_{C-1}, S_{C-2})-10b

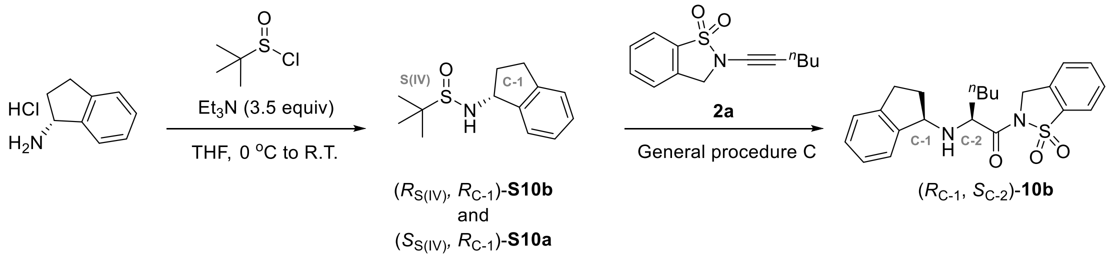

Synthesis of ($R_{S(IV)}$, R_{C-1})-**S10b** (*67*)

The title compound was obtained as an isomer during the the preparation of **S10a**. White solid (609 mg, 25% yield, >99:1 d.r.).

^{1}H NMR (400 MHz, $CDCl_3$) δ 7.56 – 7.53 (m, 1H), 7.23 – 7.21 (m, 3H), 4.90 (q, *J* = 6.7 Hz, 1H), 3.46 (d, *J* = 6.3 Hz, 1H), 3.00 (ddd, *J* = 15.9, 8.6, 4.5 Hz, 1H), 2.82 (dt, *J* = 15.7, 7.7 Hz, 1H), 2.47 (dtd, *J* = 12.5, 7.6, 4.5 Hz, 1H), 2.06 – 1.92 (m, 1H), 1.22 (s, 9H).

HPLC (Chiralpak OD-H Column), *i*-PrOH/hexane = 5/95, flow rate = 1.0 mL/min, λ = 210 nm; t_R = 7.1 min (minor), t_R = 9.0 min (major).

$[\alpha]_D^{20}$ = –49.6 (c = 1.0, $CHCl_3$).

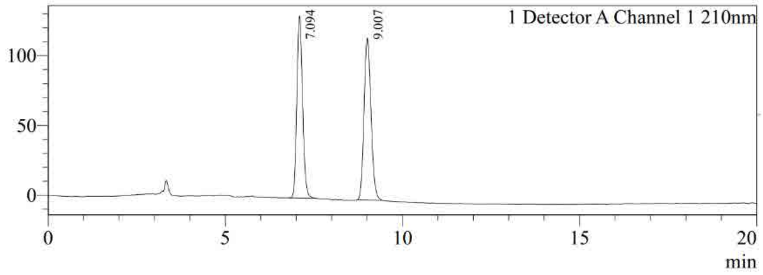

Detector A Channel 1 210nm

Peak#	Ret. Time	USP Width	Area	Height	Area%
1	7.094	0.302	1465607	130320	47.972
2	9.007	0.367	1589547	116018	52.028
Total			3055155	246338	100.000

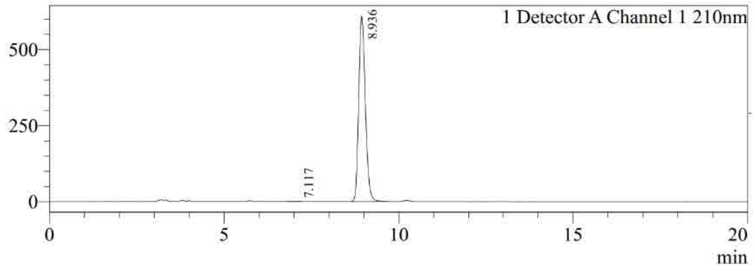

Detector A Channel 1 210nm

Peak#	Ret. Time	USP Width	Area	Height	Area%
1	7.117	0.220	2444	356	0.030
2	8.936	0.352	8053899	609270	99.970
Total			8056343	609627	100.000

(*S*)-2-(((*R*)-2,3-dihydro-1*H*-inden-1-yl)amino)-1-(1,1-dioxidobenzoisothiazol-2(3*H*)-yl)hexan-1-one (10b)

Prepared according to the **GENERAL PROCEDURE C** with ($R_{S(IV)}$, $R_{C\text{-}1}$)-**S10b** (71.2 mg, 0.30 mmol, >99:1 d.r.) and **2a** (50.0 mg, 0.20 mmol) as substrates. Column chromatography: silica gel, petroleum ether/ethyl acetate/dichloromethane = 10:1:1. Colorless viscous oil (62.8 mg, 79% yield, 98:2 d.r.).

^{1}H NMR (400 MHz, $CDCl_3$) δ 7.84 (d, *J* = 7.9 Hz, 1H), 7.72 (t, *J* = 7.6 Hz, 1H), 7.61 (t, *J* = 7.6 Hz, 1H), 7.50 (d, *J* = 7.8 Hz, 1H), 7.38 – 7.33 (m, 1H), 7.25 – 7.17 (m, 3H), 5.07 (d, *J* = 15.9 Hz, 1H), 4.94 (d, *J* = 15.6 Hz, 1H), 4.30 – 4.21 (m, 1H), 4.16 (t, *J* = 6.0 Hz, 1H), 3.07 (ddd, *J* = 15.7, 8.5, 5.3 Hz, 1H), 2.89 – 2.74 (m, 1H), 2.48 – 2.32 (m, 1H), 2.18 (s, 1H), 2.01 – 1.85 (m, 2H), 1.61 (tdt, *J* = 13.0, 8.2, 3.8 Hz, 2H), 1.53 – 1.43 (m, 1H), 1.36 (dq, *J* = 14.8, 7.4 Hz, 2H), 0.91 (t, *J* = 7.3 Hz, 3H).

^{13}C NMR (101 MHz, $CDCl_3$) δ 176.0, 144.9, 144.0, 134.4, 134.2, 130.9, 129.8, 127.6, 126.4, 125.1, 124.8, 123.7, 121.9, 61.6, 60.3, 47.5, 34.2, 33.3, 30.6, 28.3, 22.6, 14.1.

HRMS (ESI, m/z): $[M+H]^+$ Calcd. For $C_{22}H_{27}N_2O_3S$: 399.1742; Found: 399.1743.

HPLC (Chiralpak OD-H Column), *i*-PrOH/hexane = 2/98, flow rate = 1.0 mL/min, λ = 210 nm; t_R = 18.7 min (major), t_R = 22.3 min (minor).

[α]$_D$^{25} = +3.30 (c = 0.5, $CHCl_3$).

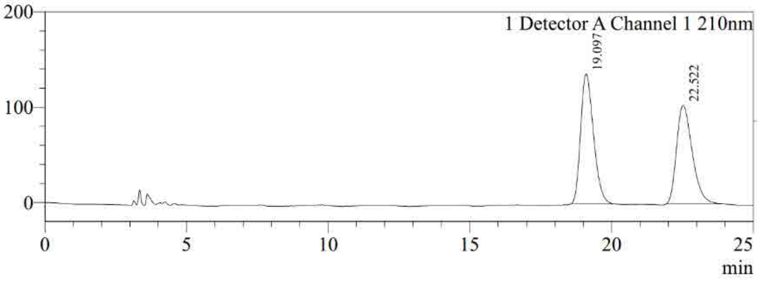

Detector A Channel 1 210nm

Peak#	Ret. Time	USP Width	Area	Height	Area%
1	19.097	0.835	4298098	136242	51.832
2	22.522	1.011	3994304	103561	48.168
Total			8292402	239803	100.000

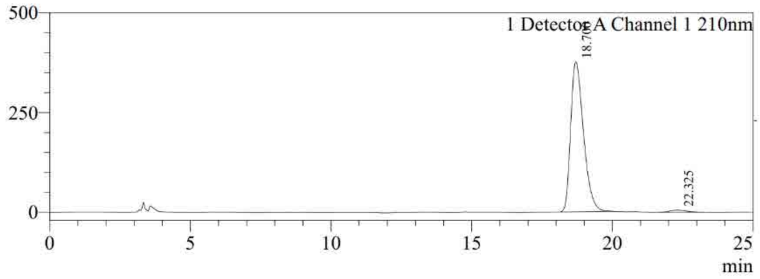

Detector A Channel 1 210nm

Peak#	Ret. Time	USP Width	Area	Height	Area%
1	18.700	0.831	11932356	376301	98.181
2	22.325	1.030	221129	5479	1.819
Total			12153485	381780	100.000

Synthesis of (S_{C-1}, S_{C-2})-11a

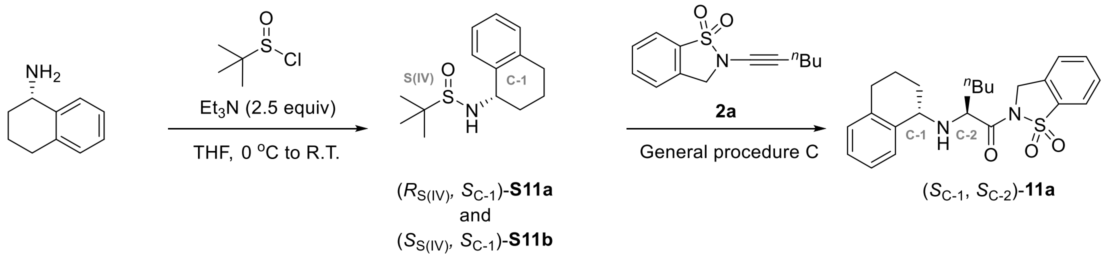

Synthesis of ($R_{S(IV)}$, S_{C-1})-**S11a** (*67*)

Under N_2 atmosphere, the mixture of (*S*)-1,2,3,4-tetrahydronaphthalen-1-amine (1.47 g, 10 mmol, 1.0 equiv) and Et_3N (2.53 g, 25 mmol, 2.5 equiv) in THF was cooled at 0 °C and then 2-methylpropane-2-sulfinic chloride (2.0 equiv) dissolved in THF was added slowly. The resulted mixture was stirred at room temperature overnight and then quenched with saturated aqueous NH_4Cl, extracted with ethyl acetate. The combined organic layer was washed with brine and concentrated under vacuum. The crude product was purified through flash chromatography (eluent: petroleum ether/ethyl acetate = 5:1 to 3:1) to afford ($R_{S(IV)}$, S_{C-1})-**S11a** as white solid (493 mg, 20% yield,

99:1 d.r.) and ($S_{S(IV)}$, $S_{C\text{-}1}$)-**S11b** as white solid (1.21 g, 48% yield, 97:3 d.r.). These two compounds were used for the preparation of **11a** and **11b** respectively.

^{1}H NMR (400 MHz, $CDCl_3$) δ 7.43 – 7.37 (m, 1H), 7.19 – 7.13 (m, 2H), 7.11 – 7.04 (m, 1H), 4.48 – 4.43 (m, 1H), 3.44 (dd, *J* = 10.1, 3.0 Hz, 1H), 2.85 – 2.69 (m, 2H), 2.35 – 2.27 (m, 1H), 2.02 – 1.86 (m, 2H), 1.86 – 1.74 (m, 1H), 1.26 (s, 9H).

HPLC (Chiralpak OD-H Column), *i*-PrOH/hexane = 5/95, flow rate = 1.0 mL/min, λ = 210 nm; t_R = 7.7 min (major), t_R = 9.0 min (minor).

$[\alpha]_D^{20}$ = +33.4 (c = 1.0, $CHCl_3$).

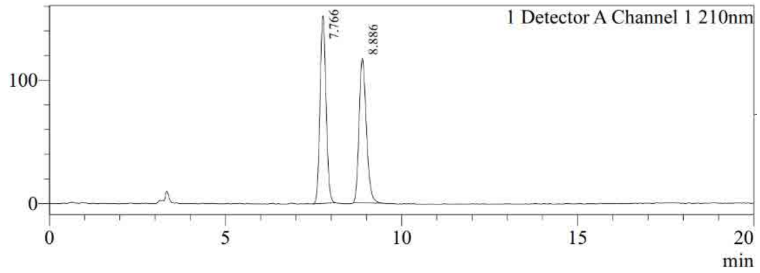

Detector A Channel 1 210nm

Peak#	Ret. Time	USP Width	Area	Height	Area%
1	7.766	0.321	1804903	151967	51.717
2	8.886	0.381	1685079	117218	48.283
Total			3489982	269185	100.000

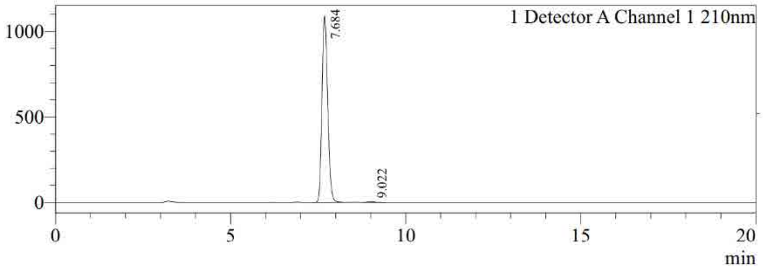

Detector A Channel 1 210nm

Peak#	Ret. Time	USP Width	Area	Height	Area%
1	7.684	0.305	12372545	1090211	99.458
2	9.022	0.350	67427	4709	0.542
Total			12439971	1094919	100.000

(*S*)-1-(1,1-Dioxidobenzoisothiazol-2(3*H*)-yl)-2-(((*S*)-1,2,3,4-tetrahydronaphthalen-1-yl)amino)-hexan-1-one (11a)

Prepared according to the **GENERAL PROCEDURE C** with ($R_{S(IV)}$, $S_{C\text{-}1}$)-**S11a** (75.4 mg, 0.30 mmol, 99:1 d.r.) and **2a** (50.0 mg, 0.20 mmol) as substrates. Column chromatography: silica gel, petroleum ether/ethyl acetate/dichloromethane = 10:1:1. Colorless viscous oil (55.4 mg, 67% yield, >99:1 d.r.).

^{1}H NMR (400 MHz, $CDCl_3$) δ 7.84 (d, *J* = 7.9 Hz, 1H), 7.72 (t, *J* = 7.6 Hz, 1H), 7.65 – 7.56 (m, 2H), 7.49 (d, *J* = 7.8 Hz, 1H), 7.18 (t, *J* = 7.4 Hz, 1H), 7.14 (t, *J* = 7.4 Hz, 1H), 7.05 (d, *J* = 7.4 Hz, 1H), 5.04 (d, *J* = 15.8 Hz, 1H), 4.92 (d, *J* = 15.8 Hz, 1H), 4.18 (s, 1H), 3.92 – 3.79 (m, 1H), 2.86 – 2.74 (m, 1H), 2.74 – 2.61 (m, 1H), 2.00 – 1.84 (m, 4H), 1.75 – 1.49 (m, 5H), 1.45 – 1.32 (m, 2H), 0.94 (t, *J* = 7.3 Hz, 3H).

^{13}C NMR (101 MHz, $CDCl_3$) δ 176.2, 139.7, 137.5, 134.6, 134.2, 131.0, 129.8, 129.1, 128.8, 126.7, 125.8, 125.1, 121.9, 61.1, 56.1, 47.6, 34.9, 30.4, 29.4, 28.4, 22.6, 19.6, 14.2.

HRMS (ESI, m/z): $[M+H]^+$ Calcd. For $C_{23}H_{29}N_2O_3S$: 413.1899; Found: 413.1901.

HPLC (Chiralpak AD-H Column), *i*-PrOH/hexane = 10/90, flow rate = 1.0 mL/min, λ = 254 nm; t_R = 12.1 min (major), t_R = 16.1 min (minor).

$[\alpha]_D^{25}$ = −1.20 (c = 0.4, $CHCl_3$).

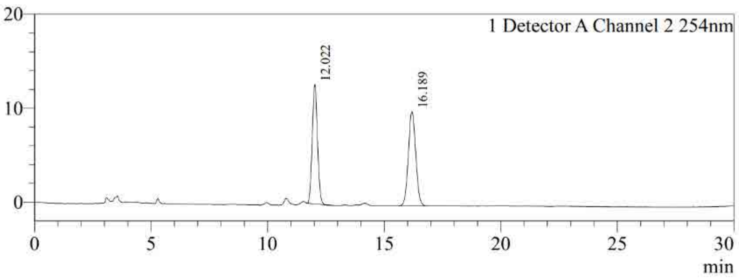

Detector A Channel 2 254nm

Peak#	Ret. Time	USP Width	Area	Height	Area%
1	12.022	0.421	199047	12693	47.296
2	16.189	0.588	221811	10036	52.704
Total			420858	22729	100.000

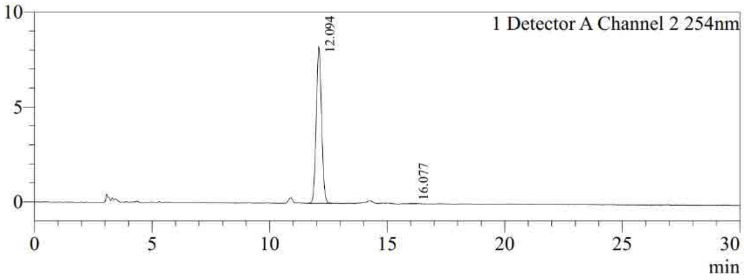

Detector A Channel 2 254nm

Peak#	Ret. Time	USP Width	Area	Height	Area%
1	12.094	0.426	131179	8246	99.988
2	16.077	0.101	15	8	0.012
Total			131195	8253	100.000

Synthesis of ($S_{C\text{-}1}$, $R_{C\text{-}2}$)-11b

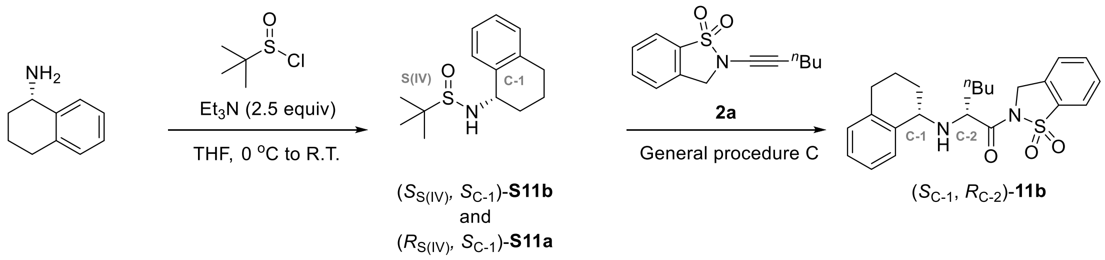

Synthesis of ($S_{S(IV)}$, $S_{C\text{-}1}$)**-S11b**

The title compound was obtained as an isomer during the the preparation of **S11a**. White solid (1.21 g, 48% yield, 97:3 d.r.).

^{1}H NMR (400 MHz, $CDCl_3$) δ 7.47 – 7.41 (m, 1H), 7.22 – 7.14 (m, 2H), 7.11 – 7.06 (m, 1H), 4.56 (q, *J* = 4.2 Hz,

1H), 3.24 (d, $J = 3.8$ Hz, 1H), 2.85 – 2.64 (m, 2H), 2.04 – 1.83 (m, 3H), 1.79 – 1.71 (m, 1H), 1.21 (s, 9H).

^{13}C NMR (101 MHz, $CDCl_3$) δ 137.8, 137.0, 129.7, 129.3, 127.6, 126.6, 55.5, 52.8, 30.7, 29.2, 22.7, 18.3.

HRMS (ESI, m/z): $[M+Na]^+$ Calcd. For $C_{14}H_{21}NONaS$: 274.1242; Found: 274.1244.

HPLC (Chiralpak OD-H Column), *i*-PrOH/hexane = 5/95, flow rate = 1.0 mL/min, λ = 210 nm; $t_R = 7.7$ min (minor), $t_R = 8.9$ min (major).

$[\alpha]_D^{20}$ = +40.0 (c = 1.0, $CHCl_3$).

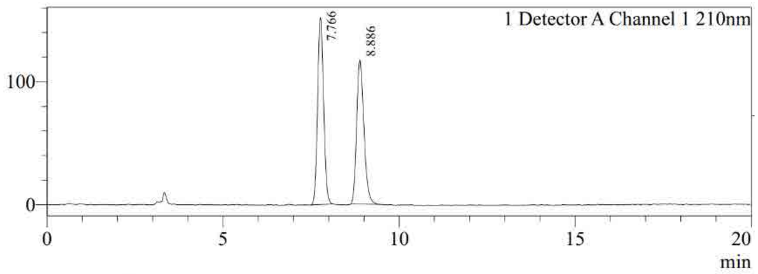

Detector A Channel 1 210nm

Peak#	Ret. Time	USP Width	Area	Height	Area%
1	7.766	0.321	1804903	151967	51.717
2	8.886	0.381	1685079	117218	48.283
Total			3489982	269185	100.000

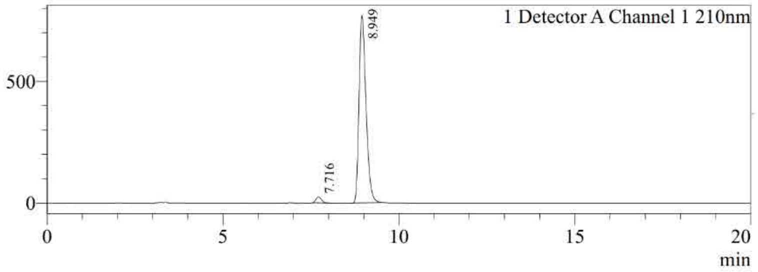

Detector A Channel 1 210nm

Peak#	Ret. Time	USP Width	Area	Height	Area%
1	7.716	0.295	277143	25571	2.538
2	8.949	0.367	10642392	768958	97.462
Total			10919535	794529	100.000

(*R*)-1-(1,1-Dioxidobenzoisothiazol-2(3*H*)-yl)-2-(((*S*)-1,2,3,4-tetrahydronaphthalen-1-yl)amino)-hexan-1-one (11b)

Prepared according to the **GENERAL PROCEDURE C** with ($S_{S(IV)}$, $S_{C\text{-}1}$)-**S11b** (75.4 mg, 0.30 mmol, 97:3 d.r.) and **2a** (50.0 mg, 0.20 mmol) as substrates. Column chromatography: silica gel, petroleum ether/ethyl acetate/dichloromethane = 10:1:1. Colorless viscous oil (57.1 mg, 69% yield, >99:1 d.r.).

^{1}H NMR (400 MHz, $CDCl_3$) δ 7.84 (d, $J = 7.9$ Hz, 1H), 7.72 (t, $J = 7.6$ Hz, 1H), 7.61 (t, $J = 7.6$ Hz, 1H), 7.51 (d, $J = 7.9$ Hz, 1H), 7.36 (d, $J = 6.5$ Hz, 1H), 7.19 – 7.12 (m, 2H), 7.08 (d, $J = 6.0$ Hz, 1H), 5.12 (d, $J = 15.8$ Hz, 1H), 4.95 (d, $J = 15.8$ Hz, 1H), 4.34 – 4.25 (m, 1H), 3.70 (t, $J = 4.0$ Hz, 1H), 2.83 (dt, $J = 17.0$, 4.4 Hz, 1H), 2.70 (ddd, $J = 16.7$, 10.8, 5.6 Hz, 1H), 2.18 – 2.03 (m, 2H), 2.02 – 1.83 (m, 2H), 1.77 – 1.67 (m, 2H), 1.66 – 1.57 (m, 1H), 1.57 – 1.45 (m, 2H), 1.42 – 1.31 (m, 2H), 0.91 (t, $J = 7.3$ Hz, 3H).

^{13}C NMR (101 MHz, $CDCl_3$) δ 176.4, 137.9, 134.6, 134.2, 131.0, 129.8, 129.3, 129.2, 127.0, 126.1, 125.2, 121.9, 59.8, 53.6, 47.5, 34.2, 29.6, 28.4, 27.5, 22.5, 18.0, 14.1.

HRMS (ESI, m/z): $[M+H]^+$ Calcd. For $C_{23}H_{29}N_2O_3S$: 413.1899; Found: 413.1902.

HPLC (Chiralpak AD-H Column), *i*-PrOH/hexane = 10/90, flow rate = 1.0 mL/min, λ = 254 nm; t_R = 12.2 min (minor), t_R = 16.1 min (major).

$[\alpha]_D^{25}$ = –5.00 (c = 0.4, $CHCl_3$).

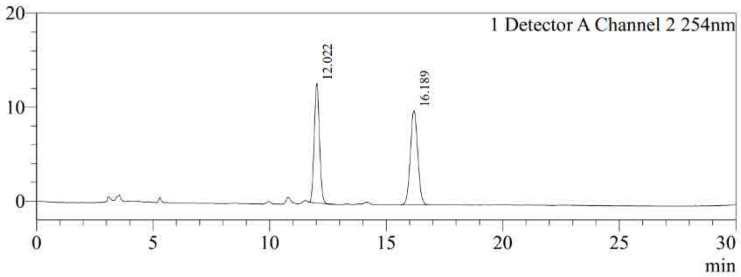

Detector A Channel 2 254nm

Peak#	Ret. Time	USP Width	Area	Height	Area%
1	12.022	0.421	199047	12693	47.296
2	16.189	0.588	221811	10036	52.704
Total			420858	22729	100.000

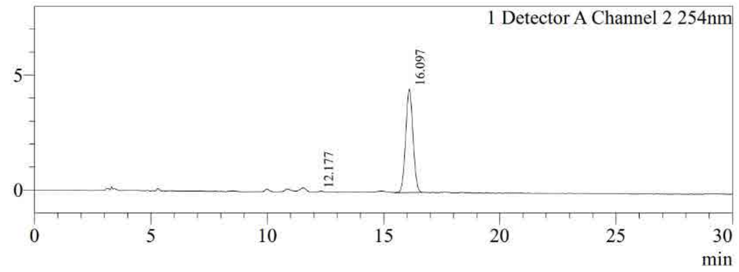

Detector A Channel 2 254nm

Peak#	Ret. Time	USP Width	Area	Height	Area%
1	12.177	0.074	14	3	0.014
2	16.097	0.581	98635	4508	99.986
Total			98650	4511	100.000

Synthesis of ($R_{C\text{-}1}$, $S_{C\text{-}2}$)-12a

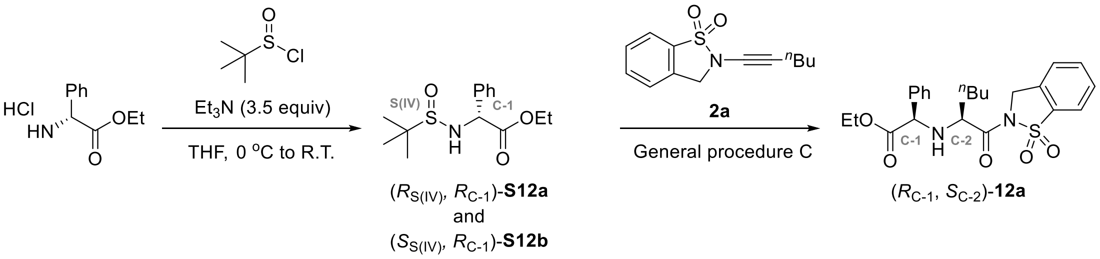

Synthesis of ($R_{S(IV)}$, $R_{C\text{-}1}$)-**S12a** (*71*)

Under N_2 atmosphere, the mixture of *D*-phenylglycine ethyl ester hydrochloride (2.16 g, 10 mmol, 1.0 equiv) and Et_3N (3.54 g, 35 mmol, 3.5 equiv) in THF was cooled at 0 °C and then 2-methylpropane-2-sulfinic chloride (2.0 equiv) dissolved in THF was added slowly. The resulted mixture was stirred at room temperature overnight and then quenched with saturated aqueous NH_4Cl, extracted with ethyl acetate. The combined organic layer was

washed with brine and concentrated under vacuum. The crude product was purified through flash chromatography (eluent: petroleum ether/ethyl acetate/dichloromethane = 8:1:1) to afford ($R_{S(IV)}$, $R_{C\text{-}1}$)-**S12a** as colorless oil (768 mg, 27% yield, 96:4 d.r.) and ($S_{S(IV)}$, $R_{C\text{-}1}$)-**S12b** as white solid (713 mg, 25% yield, 96:4 d.r.). These two compounds were used for the preparation of **12a** and **12b** respectively.

^{1}H NMR (400 MHz, $CDCl_3$) δ 7.39 – 7.29 (m, 5H), 5.05 (d, *J* = 4.4 Hz, 1H), 4.59 (d, *J* = 4.5 Hz, 1H), 4.30 – 4.04 (m, 2H), 1.23 (s, 9H), 1.19 (t, *J* = 7.2 Hz, 3H).

HPLC (Chiralpak AD-H Column), *i*-PrOH/hexane = 10/90, flow rate = 1.0 mL/min, λ = 210 nm; t_R = 7.9 min (major), t_R = 11.7 min (minor).

[α]$_D^{20}$ = –116 (c = 0.1, MeOH).

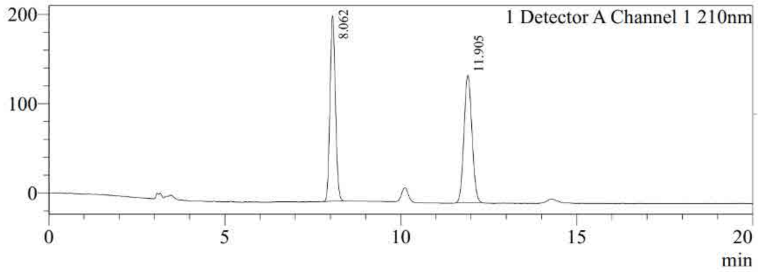

Detector A Channel 1 210nm

Peak#	Ret. Time	USP Width	Area	Height	Area%
1	8.062	0.290	2215762	207399	49.555
2	11.905	0.425	2255545	142676	50.445
Total			4471307	350075	100.000

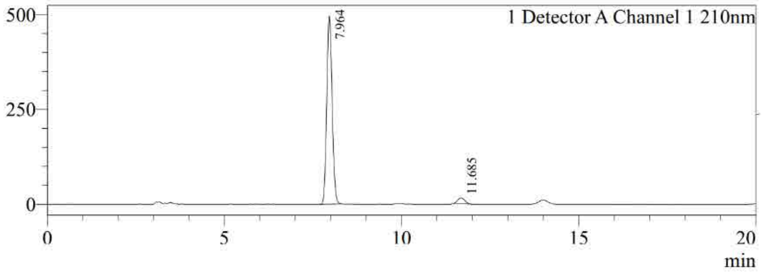

Detector A Channel 1 210nm

Peak#	Ret. Time	USP Width	Area	Height	Area%
1	7.964	0.288	5274262	496009	95.978
2	11.685	0.388	221024	15710	4.022
Total			5495286	511719	100.000

Ethyl-(*R*)-2-(((*S*)-1-(1,1-dioxidobenzoisothiazol-2(3*H*)-yl)-1-oxohexan-2-yl)amino)-2-phenyl-acetate (12a)

Prepared according to the **GENERAL PROCEDURE C** with ($R_{S(IV)}$, $R_{C\text{-}1}$)-**S12a** (80.8 mg, 0.30 mmol, 96:4 d.r.) and **2a** (50.0 mg, 0.20 mmol) as substrates. Column chromatography: silica gel, petroleum ether/ethyl acetate/dichloromethane = 8:1:1. Colorless viscous oil (60.1 mg, 67% yield, 99:1 d.r.).

^{1}H NMR (400 MHz, $CDCl_3$) δ 7.76 (d, *J* = 7.9 Hz, 1H), 7.70 (t, *J* = 7.6 Hz, 1H), 7.58 (t, *J* = 7.7 Hz, 1H), 7.50 – 7.42 (m, 3H), 7.32 (td, *J* = 10.4, 9.2, 4.3 Hz, 3H), 5.02 (d, *J* = 15.8 Hz, 1H), 4.86 (d, *J* = 16.2 Hz, 1H), 4.37 (s, 1H), 4.19 – 3.99 (m, 2H), 3.90 (s, 1H), 2.96 (s, 1H), 1.92 – 1.76 (m, 1H), 1.66 – 1.51 (m, 2H), 1.45 – 1.32 (m, 1H), 1.30 – 1.22 (m, 2H), 1.13 (t, *J* = 7.2 Hz, 3H), 0.86 (t, *J* = 7.3 Hz, 3H).

^{13}C NMR (101 MHz, $CDCl_3$) δ 175.1, 172.4, 138.2, 134.5, 134.2, 130.9, 129.8, 128.5, 128.3, 128.1, 125.1, 121.9, 64.1, 61.4, 59.6, 47.5, 34.3, 28.1, 22.4, 14.1, 14.0.

HRMS (ESI, m/z): $[M+H]^+$ Calcd. For $C_{23}H_{29}N_2O_5S$: 445.1797; Found: 445.1796.

HPLC (Chiralpak AD-H Column), *i*-PrOH/hexane = 20/80, flow rate = 1.0 mL/min, λ = 254 nm; t_R = 19.3 min (major), t_R = 22.9 min (minor).

$[\alpha]_D^{25}$ = –67.0 (c = 0.5, $CHCl_3$).

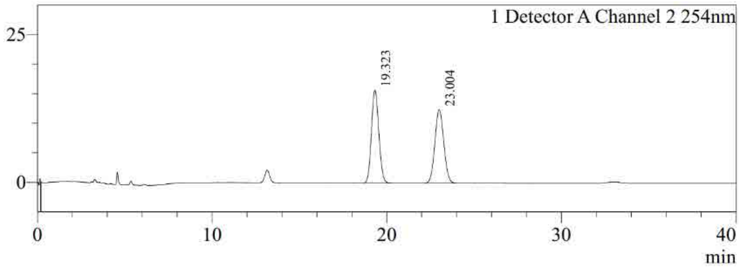

Detector A Channel 2 254nm

Peak#	Ret. Time	USP Width	Area	Height	Area%
1	19.323	0.776	460739	15720	51.105
2	23.004	0.942	440818	12431	48.895
Total			901557	28151	100.000

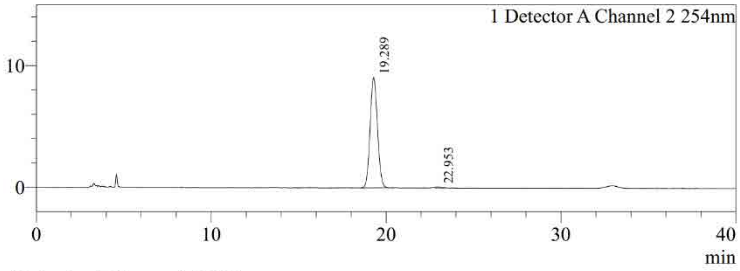

Detector A Channel 2 254nm

Peak#	Ret. Time	USP Width	Area	Height	Area%
1	19.289	0.777	265638	9074	99.269
2	22.953	0.673	1957	79	0.731
Total			267595	9153	100.000

Synthesis of ($R_{C\text{-}1}$, $R_{C\text{-}2}$)-12b

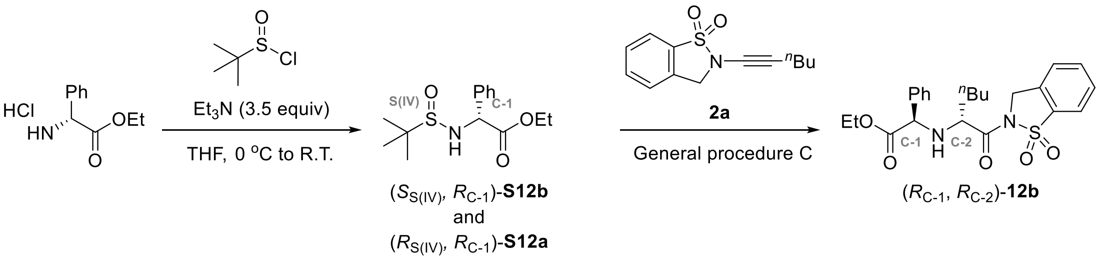

Synthesis of ($S_{S(IV)}$, $R_{C\text{-}1}$)**-S12b** (*72*)

The title compound was obtained as an isomer during the the preparation of **S12a**. White solid (713 mg, 25% yield, 96:4 d.r.).

¹H NMR (400 MHz, $CDCl_3$) δ 7.44 – 7.28 (m, 5H), 5.10 (d, *J* = 6.1 Hz, 1H), 4.27 (d, *J* = 6.2 Hz, 1H), 4.23 – 4.05 (m, 2H), 1.20 – 1.16 (m, 12H).

HPLC (Chiralpak AD-H Column), *i*-PrOH/hexane = 10/90, flow rate = 1.0 mL/min, λ = 210 nm; t_R = 8.0 min (minor), t_R = 11.7 min (major).

$[\alpha]_D^{20}$ = –34.0 (c = 0.1, MeOH).

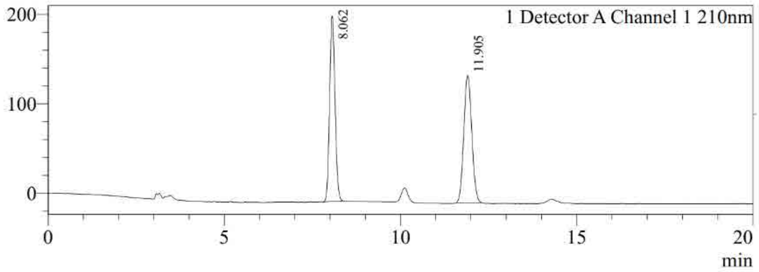

Detector A Channel 1 210nm

Peak#	Ret. Time	USP Width	Area	Height	Area%
1	8.062	0.290	2215762	207399	49.555
2	11.905	0.425	2255545	142676	50.445
Total			4471307	350075	100.000

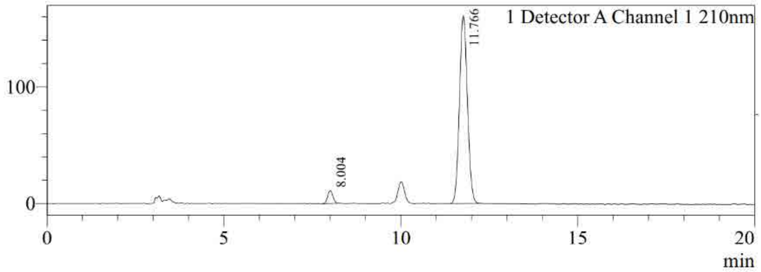

Detector A Channel 1 210nm

Peak#	Ret. Time	USP Width	Area	Height	Area%
1	8.004	0.287	115160	11016	4.372
2	11.766	0.420	2519033	160734	95.628
Total			2634193	171750	100.000

Ethyl-(*R*)-2-(((*R*)-1-(1,1-dioxidobenzoisothiazol-2(3*H*)-yl)-1-oxohexan-2-yl)amino)-2-phenyl-acetate (12b)

Prepared according to the **GENERAL PROCEDURE C** with ($S_{S(IV)}$, $R_{C\text{-}1}$)-**S12b** (80.8 mg, 0.30 mmol, 96:4 d.r.) and **2a** (50.0 mg, 0.20 mmol) as substrates. Column chromatography: silica gel, petroleum ether/ethyl acetate/dichloromethane = 8:1:1. White solid (76.7 mg, 86% yield, 95:5 d.r.).

¹H NMR (400 MHz, $CDCl_3$) δ 7.81 (d, *J* = 7.9 Hz, 1H), 7.70 (t, *J* = 7.6 Hz, 1H), 7.59 (t, *J* = 7.6 Hz, 1H), 7.44 (d, *J* = 7.8 Hz, 1H), 7.40 (d, *J* = 8.1 Hz, 2H), 7.30 – 7.21 (m, 3H), 4.81 (d, *J* = 15.9 Hz, 1H), 4.76 (d, *J* = 16.0 Hz, 1H), 4.46 (s, 1H), 4.26 – 4.09 (m, 3H), 2.60 (s, 1H), 1.88 (ddt, *J* = 16.3, 11.5, 5.5 Hz, 1H), 1.71 – 1.54 (m, 2H), 1.52 – 1.30 (m, 3H), 1.20 (t, *J* = 7.2 Hz, 3H), 0.90 (t, *J* = 7.3 Hz, 3H).

¹³C NMR (101 MHz, $CDCl_3$) δ 174.2, 172.4, 138.4, 134.4, 134.2, 130.9, 129.8, 128.6, 128.5, 128.3, 128.2, 128.0, 125.1, 121.9, 63.9, 61.3, 60.1, 47.3, 33.6, 28.0, 22.6, 14.2, 14.0.

HRMS (ESI, m/z): $[M+H]^+$ Calcd. For $C_{23}H_{29}N_2O_5S$: 445.1797; Found: 445.1797.

HPLC (Chiralpak AD-H Column), *i*-PrOH/hexane = 20/80, flow rate = 1.0 mL/min, λ = 254 nm; t_R = 19.3 min (minor), t_R = 23.0 min (major).

[α]D[25] = –43.2 (c = 0.5, $CHCl_3$).

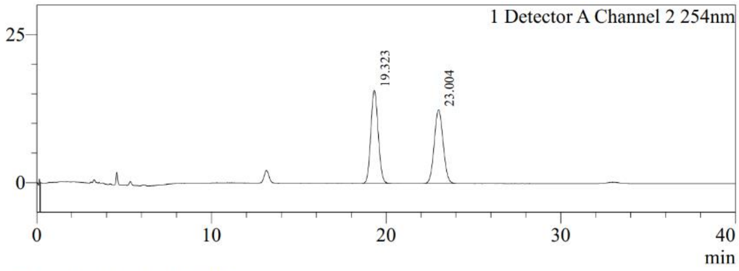

Detector A Channel 2 254nm

Peak#	Ret. Time	USP Width	Area	Height	Area%
1	19.323	0.776	460739	15720	51.105
2	23.004	0.942	440818	12431	48.895
Total			901557	28151	100.000

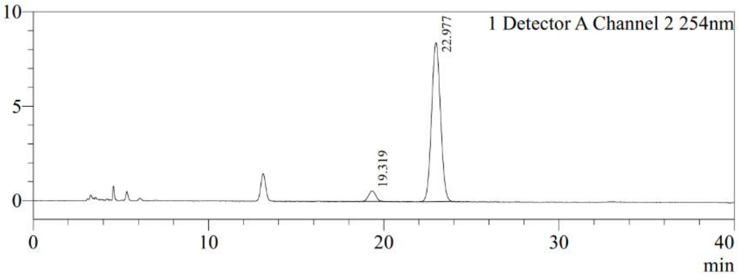

Detector A Channel 2 254nm

Peak#	Ret. Time	USP Width	Area	Height	Area%
1	19.319	0.792	15922	560	5.054
2	22.977	0.945	299078	8434	94.946
Total			315000	8994	100.000

Synthesis of (S_{C-1}, S_{C-2})-13a

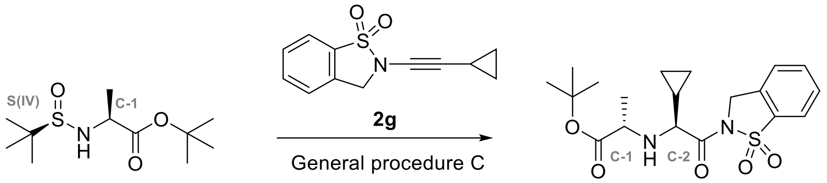

($R_{S(IV)}$, S_{C-1})-**S4d** (S_{C-1}, S_{C-2})-**13a**

Prepared according to the **GENERAL PROCEDURE C** with ($R_{S(IV)}$, S_{C-1})-**S4d** (74.8 mg, 0.30 mmol, >99:1 d.r.) and **2g** (46.6 mg, 0.20 mmol) as substrates. Column chromatography: silica gel, petroleum ether/ethyl acetate/dichloromethane = 5:1:1. Colorless viscous oil (39.6 mg, 50% yield, >99:1 d.r.).

^{1}H NMR (400 MHz, $CDCl_3$) δ 7.79 (d, *J* = 7.9 Hz, 1H), 7.69 (t, *J* = 7.6 Hz, 1H), 7.57 (t, *J* = 7.8 Hz, 1H), 7.47 (d, *J* = 7.9 Hz, 1H), 5.08 (d, *J* = 15.9 Hz, 1H), 4.89 (d, *J* = 15.9 Hz, 1H), 3.53 (d, *J* = 8.5 Hz, 1H), 3.30 (q, *J* = 7.1 Hz, 1H), 2.25 (s, 1H), 1.46 (s, 9H), 1.30 – 1.21 (m, 4H), 0.76 (dq, *J* = 10.0, 4.7 Hz, 1H), 0.64 (tq, *J* = 8.2, 5.2 Hz, 1H), 0.51 (ddq, *J* = 17.8, 9.0, 4.7, 4.0 Hz, 2H).

^{13}C NMR (101 MHz, $CDCl_3$) δ 174.4, 173.7, 134.5, 134.2, 130.8, 129.7, 125.0, 121.9, 81.1, 64.3, 55.2, 47.6, 28.1, 20.0, 14.9, 4.2, 2.4.

HRMS (ESI, m/z): $[M+H]^+$ Calcd. For $C_{19}H_{27}N_2O_5S$: 395.1641; Found: 395.1642.

HPLC (Chiralpak OD-H Column), *i*-PrOH/hexane = 3/97, flow rate = 1.0 mL/min, λ = 210 nm; t_R = 21.3 min (major), t_R = 25.3 min (minor).

$[\alpha]_D^{25}$ = –27.2 (c = 0.5, $CHCl_3$).

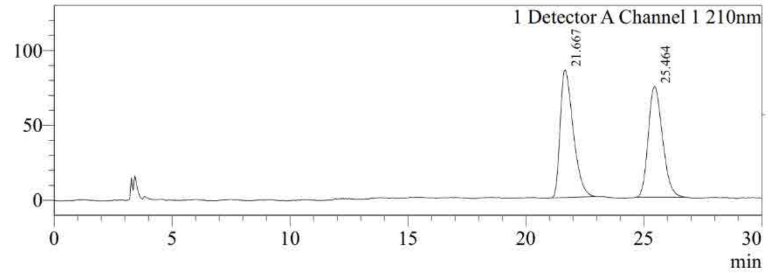

Detector A Channel 1 210nm

Peak#	Ret. Time	USP Width	Area	Height	Area%
1	21.667	1.014	3267706	85082	51.342
2	25.464	1.112	3096848	73677	48.658
Total			6364554	158759	100.000

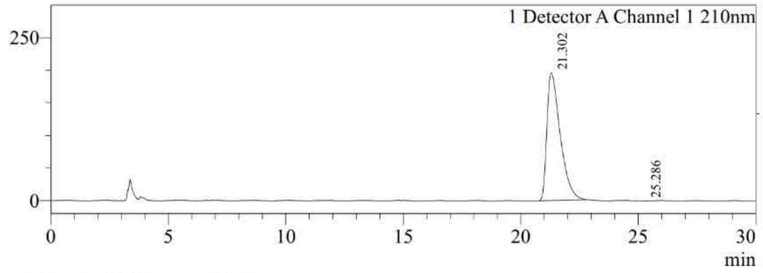

Detector A Channel 1 210nm

Peak#	Ret. Time	USP Width	Area	Height	Area%
1	21.302	1.021	7729581	195677	99.994
2	25.286	0.076	470	149	0.006
Total			7730050	195826	100.000

Synthesis of ($S_{C\text{-}1}$, $R_{C\text{-}2}$)-13b

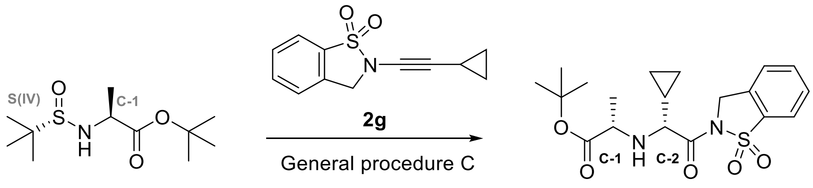

($S_{S(IV)}$, $S_{C\text{-}1}$)-**S4b** ($S_{C\text{-}1}$, $R_{C\text{-}2}$)-**13b**

Prepared according to the **GENERAL PROCEDURE C** with ($S_{S(IV)}$, $S_{C\text{-}1}$)-**S4b** (74.8 mg, 0.30 mmol, >99:1 d.r.) and **2g** (46.6 mg, 0.20 mmol) as substrates. Column chromatography: silica gel, petroleum ether/ethyl acetate/dichloromethane = 5:1:1. Colorless viscous oil (36.5 mg, 46% yield, >99:1 d.r.).

^{1}H NMR (400 MHz, $CDCl_3$) δ 7.80 (d, *J* = 7.9 Hz, 1H), 7.70 (t, *J* = 7.6 Hz, 1H), 7.58 (t, *J* = 7.6 Hz, 1H), 7.48 (d, *J* = 7.8 Hz, 1H), 5.06 (d, *J* = 15.9 Hz, 1H), 4.92 (d, *J* = 16.0 Hz, 1H), 3.70 (d, *J* = 7.2 Hz, 1H), 3.37 (q, *J* = 6.9 Hz,

1H), 2.52 (s, 1H), 1.38 (s, 9H), 1.27 (dd, J = 19.3, 9.8 Hz, 4H), 0.71 (q, J = 6.9, 6.3 Hz, 1H), 0.59 (q, J = 8.4, 6.5 Hz, 1H), 0.45 (t, J = 6.3 Hz, 2H).

^{13}C NMR (101 MHz, $CDCl_3$) δ 174.1, 173.9, 134.5, 134.2, 130.9, 129.8, 125.1, 121.9, 81.1, 62.1, 55.5, 47.5, 28.0, 19.1, 14.7, 3.6, 1.8.

HRMS (ESI, m/z): $[M+H]^+$ Calcd. For $C_{19}H_{27}N_2O_5S$: 395.1641; Found: 395.1643.

HPLC (Chiralpak OD-H Column), *i*-PrOH/hexane = 3/97, flow rate = 1.0 mL/min, λ = 210 nm; t_R = 21.5 min (minor), t_R = 25.3 min (major).

$[\alpha]_D^{25}$ = +1.20 (c = 0.5, $CHCl_3$).

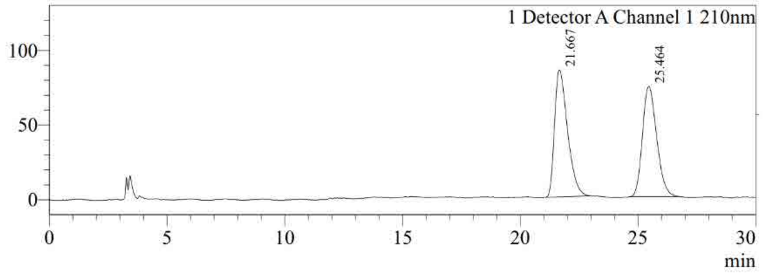

Detector A Channel 1 210nm

Peak#	Ret. Time	USP Width	Area	Height	Area%
1	21.667	1.014	3267706	85082	51.342
2	25.464	1.112	3096848	73677	48.658
Total			6364554	158759	100.000

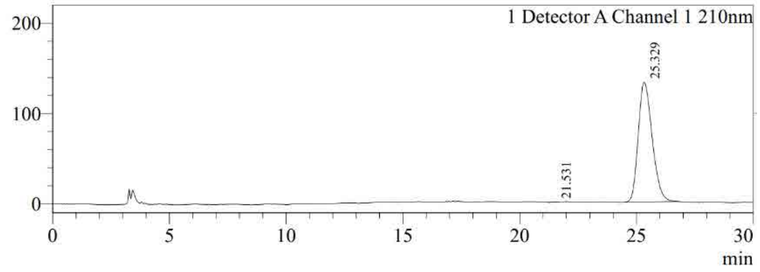

Detector A Channel 1 210nm

Peak#	Ret. Time	USP Width	Area	Height	Area%
1	21.531	0.469	6464	259	0.117
2	25.329	1.095	5497865	132475	99.883
Total			5504329	132734	100.000

Synthesis of ($R_{C\text{-}1}$, $S_{C\text{-}2}$)-14a

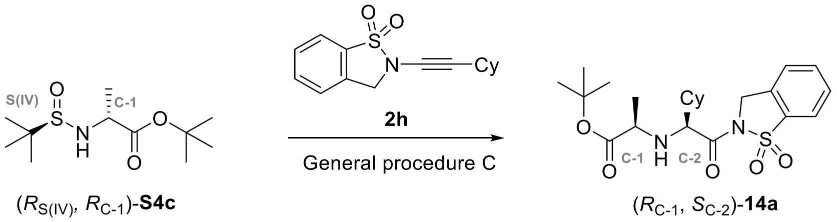

($R_{S(IV)}$, $R_{C\text{-}1}$)-**S4c** ($R_{C\text{-}1}$, $S_{C\text{-}2}$)-**14a**

Prepared according to the **GENERAL PROCEDURE C** with ($R_{S(IV)}$, $R_{C\text{-}1}$)-**S4c** (74.8 mg, 0.30 mmol, >99:1 d.r.) and **2h** (55.0 mg, 0.20 mmol) as substrates. Column chromatography: silica gel, petroleum ether/ethyl acetate/dichloromethane = 20:1:1. Colorless viscous oil (62.8 mg, 72% yield, >99:1 d.r.).

^{1}H NMR (400 MHz, $CDCl_3$) δ 7.79 (d, J = 7.9 Hz, 1H), 7.69 (t, J = 7.6 Hz, 1H), 7.58 (t, J = 7.7 Hz, 1H), 7.47 (d, J = 7.8 Hz, 1H), 5.04 (d, J = 15.9 Hz, 1H), 4.87 (d, J = 15.9 Hz, 1H), 3.78 (d, J = 5.9 Hz, 1H), 3.27 (q, J = 6.9 Hz, 1H), 2.50 (s, 1H), 1.98 (d, J = 11.9 Hz, 1H), 1.79 – 1.55 (m, 5H), 1.48 – 1.38 (m, 1H), 1.35 (s, 9H), 1.28 (d, J = 6.9 Hz, 3H), 1.22 – 1.02 (m, 4H).

^{13}C NMR (101 MHz, $CDCl_3$) δ 174.6, 174.5, 134.6, 134.1, 131.0, 129.7, 125.1, 121.8, 80.9, 65.1, 56.1, 47.4, 42.4, 30.0, 28.2, 28.0, 26.5, 26.3, 26.3, 19.1.

HRMS (ESI, m/z): $[M+H]^+$ Calcd. For $C_{22}H_{33}N_2O_5S$: 437.2110; Found: 437.2112.

HPLC (Chiralpak OD-H Column), *i*-PrOH/hexane = 5/95, flow rate = 1.0 mL/min, λ = 210 nm; t_R = 8.5 min (major), t_R = 10.6 min (minor).

$[\alpha]_D^{25}$ = +70.4 (c = 0.5, $CHCl_3$).

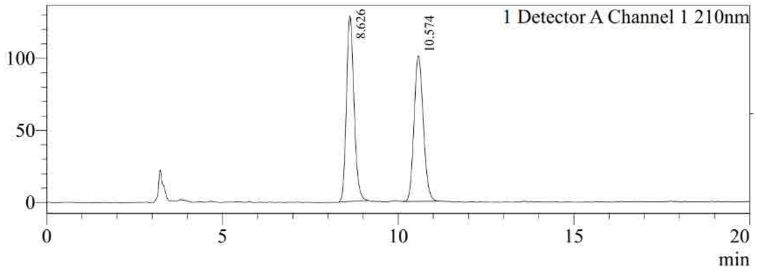

Detector A Channel 1 210nm

Peak#	Ret. Time	USP Width	Area	Height	Area%
1	8.626	0.399	1930476	128620	51.026
2	10.574	0.487	1852813	100903	48.974
Total			3783289	229523	100.000

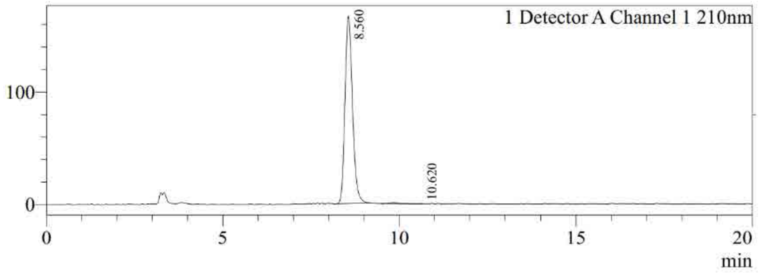

Detector A Channel 1 210nm

Peak#	Ret. Time	USP Width	Area	Height	Area%
1	8.560	0.390	2462925	166593	99.371
2	10.620	0.075	15601	182	0.629
Total			2478525	166775	100.000

Synthesis of ($\boldsymbol{R}_{\text{C-1}}$, $\boldsymbol{R}_{\text{C-2}}$)-14b

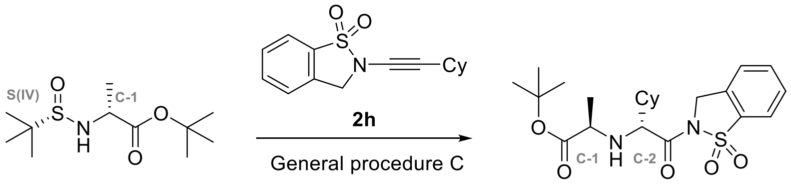

($S_{S(IV)}$, R_{C-1})-**S4a**　　　　　　　　　　　　　　　　(R_{C-1}, R_{C-2})-**14b**

Prepared according to the **GENERAL PROCEDURE C** with ($S_{S(IV)}$, R_{C-1})-**S4a** (74.8 mg, 0.30 mmol, >99:1 d.r.) and **2h** (55.0 mg, 0.20 mmol) as substrates. Column chromatography: silica gel, petroleum ether/ethyl acetate/dichloromethane = 20:1:1. Colorless viscous oil (49.5 mg, 56% yield, >99:1 d.r.).

^{1}H NMR (400 MHz, $CDCl_3$) δ 7.80 (d, J = 7.9 Hz, 1H), 7.69 (t, J = 7.6 Hz, 1H), 7.58 (t, J = 7.7 Hz, 1H), 7.46 (d, J = 7.8 Hz, 1H), 5.01 (d, J = 15.8 Hz, 1H), 4.86 (d, J = 15.8 Hz, 1H), 4.02 – 3.91 (m, 1H), 3.28 (q, J = 7.1 Hz, 1H), 2.20 – 2.03 (m, 1H), 1.96 (d, J = 11.4 Hz, 1H), 1.85 – 1.67 (m, 3H), 1.63 (d, J = 10.6 Hz, 2H), 1.46 (s, 9H), 1.42 – 1.31 (m, 2H), 1.26 (d, J = 7.1 Hz, 3H), 1.20 – 1.07 (m, 3H).

^{13}C NMR (101 MHz, $CDCl_3$) δ 174.4, 174.1, 134.6, 134.1, 130.9, 129.7, 125.1, 122.0, 80.9, 65.2, 54.7, 47.4, 40.7, 30.2, 28.5, 28.2, 26.4, 26.3, 26.2, 20.0.

HRMS (ESI, m/z): $[M+H]^+$ Calcd. For $C_{22}H_{33}N_2O_5S$: 437.2110; Found: 437.2112.

HPLC (Chiralpak OD-H Column), *i*-PrOH/hexane = 5/95, flow rate = 1.0 mL/min, λ = 210 nm; t_R = 8.6 min (minor), t_R = 10.4 min (major).

$[\alpha]_D^{25}$ = –6.80 (c = 0.5, $CHCl_3$).

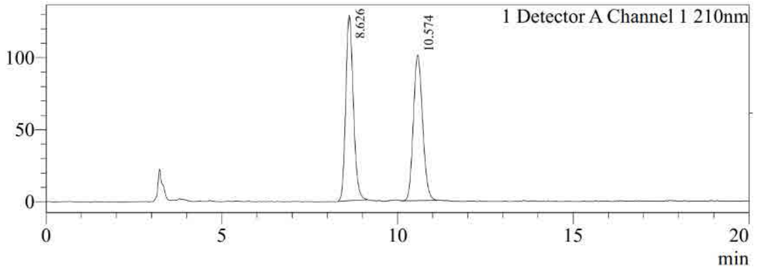

Detector A Channel 1 210nm

Peak#	Ret. Time	USP Width	Area	Height	Area%
1	8.626	0.399	1930476	128620	51.026
2	10.574	0.487	1852813	100903	48.974
Total			3783289	229523	100.000

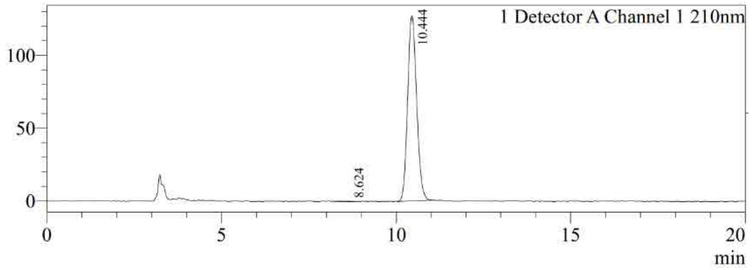

Detector A Channel 1 210nm

Peak#	Ret. Time	USP Width	Area	Height	Area%
1	8.624	0.105	1005	232	0.044
2	10.444	0.476	2277681	127255	99.956
Total			2278687	127487	100.000

Synthesis of ($R_{C\text{-}1}$, $S_{C\text{-}2}$)-15a

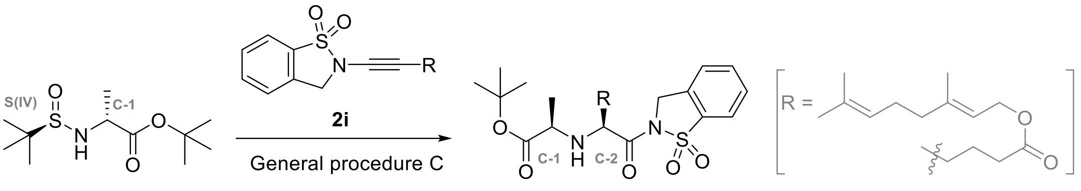

Prepared according to the **GENERAL PROCEDURE C** with ($R_{S(IV)}$, $R_{C\text{-}1}$)-**S4c** (74.8 mg, 0.30 mmol, >99:1 d.r.) and **2i** (86.0 mg, 0.20 mmol) as substrates. Column chromatography: silica gel, petroleum ether/ethyl acetate/dichloromethane = 8:1:1. Colorless viscous oil (87.6 mg, 74% yield, >99:1 d.r.).

^{1}H NMR (400 MHz, $CDCl_3$) δ 7.78 (d, J = 7.9 Hz, 1H), 7.69 (t, J = 7.6 Hz, 1H), 7.57 (t, J = 7.8 Hz, 1H), 7.47 (d, J = 7.8 Hz, 1H), 5.34 – 5.26 (m, 1H), 5.08 – 5.02 (m, 1H), 4.99 (d, J = 15.9 Hz, 1H), 4.88 (d, J = 16.0 Hz, 1H), 4.55 (d, J = 7.1 Hz, 2H), 4.07 – 3.96 (m, 1H), 3.26 (q, J = 6.9 Hz, 1H), 2.51 (s, 1H), 2.29 (t, J = 7.1 Hz, 2H), 2.04 (dq, J = 24.1, 7.9 Hz, 4H), 1.84 (tt, J = 13.0, 6.4 Hz, 1H), 1.66 (d, J = 5.6 Hz, 6H), 1.61 – 1.55 (m, 4H), 1.51 – 1.42 (m, 1H), 1.37 (s, 9H), 1.32 – 1.24 (d, J = 7.0 Hz, 4H).

^{13}C NMR (101 MHz, $CDCl_3$) δ 174.7, 174.1, 173.7, 142.0, 134.4, 134.2, 131.8, 130.9, 129.8, 125.1, 123.9, 121.8, 118.5, 81.0, 61.2, 59.7, 55.7, 47.4, 39.6, 34.3, 34.2, 28.0, 26.3, 25.7, 25.4, 24.8, 18.8, 17.8, 16.5.

HRMS (ESI, m/z): $[M+H]^+$ Calcd. For $C_{30}H_{45}N_2O_7S$: 577.2947; Found: 577.2932.

HPLC (Chiralpak OD-H Column), *i*-PrOH/hexane = 5/95, flow rate = 1.0 mL/min, λ = 210 nm; t_R = 16.9 min (major), t_R = 19.3 min (minor).

$[\alpha]_D^{25}$ = +1.20 (c = 0.5, $CHCl_3$).

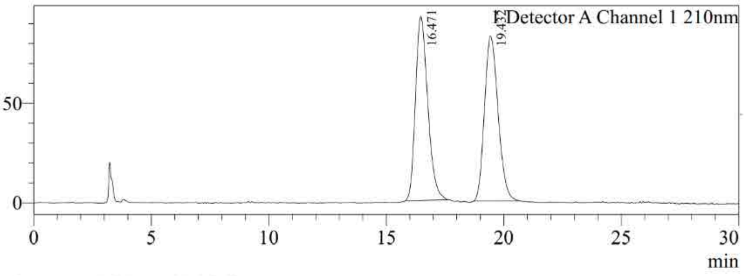

Detector A Channel 1 210nm

Peak#	Ret. Time	USP Width	Area	Height	Area%
1	16.471	0.950	3358000	92537	50.209
2	19.432	1.067	3330088	82797	49.791
Total			6688088	175335	100.000

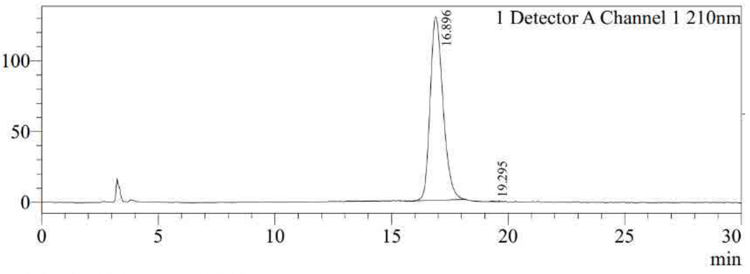

Detector A Channel 1 210nm

Peak#	Ret. Time	USP Width	Area	Height	Area%
1	16.896	0.995	4978938	129765	99.961
2	19.295	0.106	1944	365	0.039
Total			4980882	130130	100.000

Synthesis of (R_{C-1}, R_{C-2})-15b

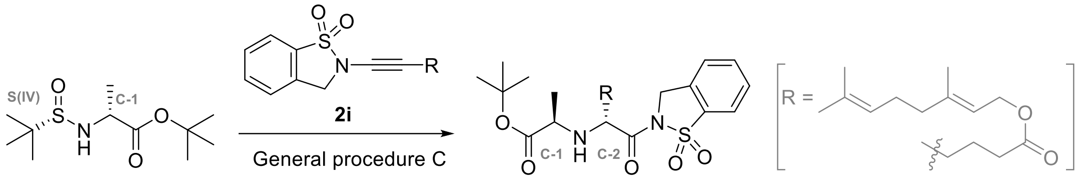

($S_{S(IV)}$, R_{C-1})-**S4a** (R_{C-1}, R_{C-2})-**15b**

Prepared according to the **GENERAL PROCEDURE C** with ($S_{S(IV)}$, R_{C-1})-**S4a** (74.8 mg, 0.30 mmol, >99:1 d.r.) and **2i** (86.0 mg, 0.20 mmol) as substrates. Column chromatography: silica gel, petroleum ether/ethyl acetate/dichloromethane = 8:1:1. Colorless viscous oil (81.3 mg, 69% yield, >99:1 d.r.).

^{1}H NMR (400 MHz, $CDCl_3$) δ 7.78 (d, J = 7.9 Hz, 1H), 7.68 (t, J = 7.6 Hz, 1H), 7.56 (t, J = 7.6 Hz, 1H), 7.45 (d, J = 7.8 Hz, 1H), 5.29 (t, J = 7.2 Hz, 1H), 5.08 – 5.01 (m, 1H), 4.97 (d, J = 15.9 Hz, 1H), 4.86 (d, J = 15.9 Hz, 1H), 4.54 (d, J = 7.1 Hz, 2H), 4.13 (t, J = 6.2 Hz, 1H), 3.26 (q, J = 7.0 Hz, 1H), 2.29 (t, J = 7.1 Hz, 2H), 2.04 (ddd, J = 24.4, 19.2, 14.4 Hz, 5H), 1.91 – 1.79 (m, 1H), 1.65 (d, J = 4.4 Hz, 9H), 1.56 (s, 3H), 1.44 (s, 9H), 1.25 (d, J = 6.8 Hz, 3H).

^{13}C NMR (101 MHz, $CDCl_3$) δ 174.3, 174.1, 173.7, 142.0, 134.4, 134.1, 131.8, 130.8, 129.7, 125.1, 123.8, 121.9, 118.5, 81.0, 61.2, 60.1, 55.0, 47.4, 39.6, 34.2, 33.3, 28.1, 26.3, 25.7, 25.5, 24.9, 19.7, 17.7, 16.5.

HRMS (ESI, m/z): $[M+H]^+$ Calcd. For $C_{30}H_{45}N_2O_7S$: 577.2947; Found: 577.2941.

HPLC (Chiralpak OD-H Column), *i*-PrOH/hexane = 5/95, flow rate = 1.0 mL/min, λ = 210 nm; t_R = 17.0 min (minor), t_R = 19.8 min (major).

$[\alpha]_D^{25}$ = +10.4 (c = 0.5, $CHCl_3$).

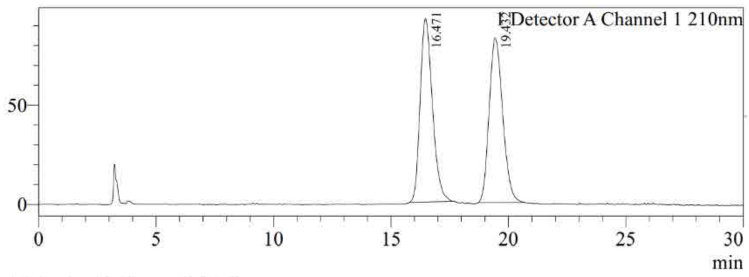

Detector A Channel 1 210nm

Peak#	Ret. Time	USP Width	Area	Height	Area%
1	16.471	0.950	3358000	92537	50.209
2	19.432	1.067	3330088	82797	49.791
Total			6688088	175335	100.000

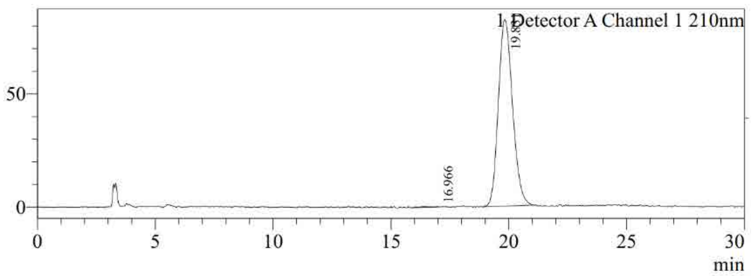

Detector A Channel 1 210nm

Peak#	Ret. Time	USP Width	Area	Height	Area%
1	16.966	0.117	9623	233	0.278
2	19.831	1.110	3454385	82564	99.722
Total			3464008	82797	100.000

Synthesis of ($R_{C\text{-}1}$, $S_{C\text{-}2}$)-16a

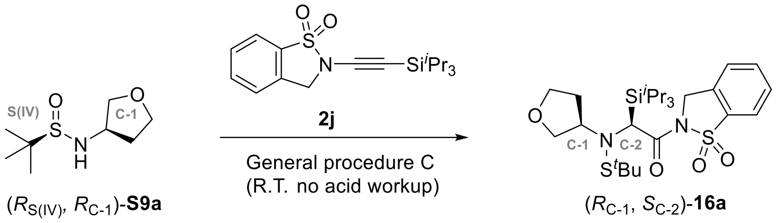

($R_{S(IV)}$, $R_{C\text{-}1}$)-**S9a** ($R_{C\text{-}1}$, $S_{C\text{-}2}$)-**16a**

16a was prepared from ($R_{S(IV)}$, $R_{C\text{-}1}$)-**S9a** (57.4 mg, 0.30 mmol, 99:1 d.r.) and **2j** (69.9 mg, 0.20 mmol) according to the **GENERAL PROCEDURE C** but at room temperature and without acid workup. Column chromatography: silica gel, petroleum ether/ethyl acetate/dichloromethane = 20:1:1. White solid (26.3 mg, 24% yield, >20:1 d.r. signal of diastereomers cannot be observed in ^{1}H NMR).

^{1}H NMR (400 MHz, $CDCl_3$) δ 7.80 (d, J = 8.1 Hz, 1H), 7.67 (t, J = 7.0 Hz, 1H), 7.57 (t, J = 7.6 Hz, 1H), 7.45 (d, J = 7.8 Hz, 1H), 5.28 – 4.62 (m, 2H), 4.01 (qd, J = 8.1, 5.9 Hz, 1H), 3.93 (t, J = 8.0 Hz, 1H), 3.83 (td, J = 6.7, 6.1, 1.8 Hz, 2H), 3.55 (t, J = 8.4 Hz, 1H), 2.53 – 2.35 (m, 1H), 2.63 – 2.08 (m, 1H), 1.66 (s, 1H), 1.41 – 1.31 (m, 12H), 1.24 – 1.12 (m, 18H).

^{13}C NMR (101 MHz, $CDCl_3$) δ 171.3, 133.8, 129.7, 124.9, 121.7, 70.9, 68.6, 67.4, 60.5, 47.6, 31.2, 29.6, 21.2, 19.0, 18.9, 14.3, 12.5.

HRMS (ESI, m/z): $[M+Na]^+$ Calcd. For $C_{26}H_{44}N_2O_4NaS_2Si$: 563.2409; Found: 563.2413.

$[\alpha]_D^{25}$ = –16.0 (c = 0.5, $CHCl_3$).

Synthesis of ($R_{C\text{-}1}$, $S_{C\text{-}2}$)-17a

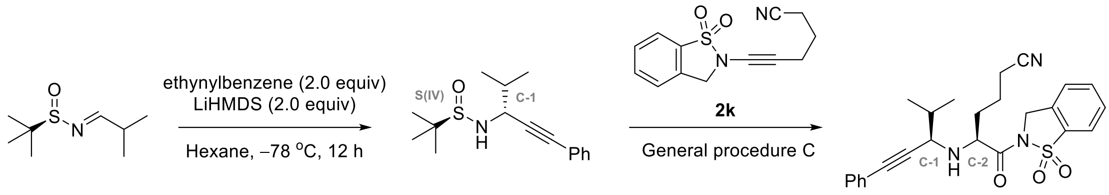

($R_{S(IV)}$, $R_{C\text{-}1}$)-**S17a** ($R_{C\text{-}1}$, $S_{C\text{-}2}$)-**17a**

Synthesis of ($R_{S(IV)}$, $R_{C\text{-}1}$)-**S17a** (*41*)

Accrding to the known literature procedure (*73*): Under N_2 atmosphere, LiHMDS (4.0 mL, 1.0 M in THF, 2.0 equiv) was added dropwise to the solution of ethynylbenzene (408 mg, 4.0 mmol, 2.0 equiv) in hexane (24 mL) at –78 °C. The resulting mixture was then transferred to 0 ℃ and stirred for 10 minutes before the addition of (*R*)-sulfinimine (350 mg, 2.0 mmol, 1.0 equiv) dissolved in THF (20 mL) dropwise at –78 °C. The mixture was stirred at –78 °C overnight, and then quenched by saturated aqueous NH_4Cl. After extraction with ethyl acetate, the combined organic layer was washed with brine and concentrated under vacuum. The crude product was purified through flash chromatography (eluent: petroleum ether/ethyl acetate = 10:1 to 6:1) to afford ($R_{S(IV)}$, $R_{C\text{-}1}$)-**S17a** as colorless viscous oil (510 mg, 92% yield, 99:1 d.r.).

^{1}H NMR (400 MHz, $CDCl_3$) δ 7.44 – 7.41 (m, 2H), 7.28 – 7.25 (m, 3H), 4.11 (t, *J* = 5.8 Hz, 1H), 3.42 (d, *J* = 6.4 Hz, 1H), 2.05 – 1.97 (m, 1H), 1.23 (s, 9H), 1.05 (d, *J* = 6.8 Hz, 6H).

HPLC (Chiralpak OD-H Column), *i*-PrOH/hexane = 5/95, flow rate = 1.0 mL/min, λ = 210 nm; t_R = 5.5 min (major), t_R = 7.6 min (minor).

$[\alpha]_D^{25}$ = –26.0 (c = 0.5, $CHCl_3$).

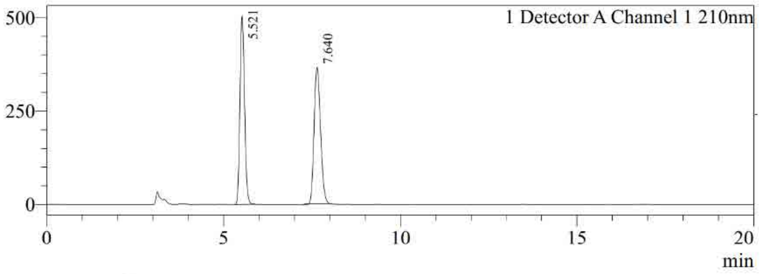

Detector A Channel 1 210nm

Peak#	Ret. Time	USP Width	Area	Height	Area%
1	5.521	0.248	4571205	503840	49.612
2	7.640	0.344	4642786	364856	50.388
Total			9213991	868696	100.000

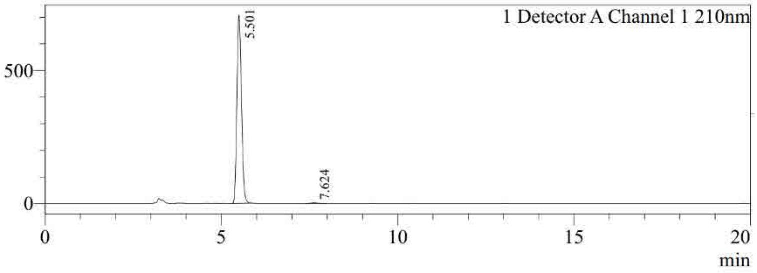

Detector A Channel 1 210nm

Peak#	Ret. Time	USP Width	Area	Height	Area%
1	5.501	0.248	6409345	706031	99.478
2	7.624	0.322	33635	2560	0.522
Total			6442981	708591	100.000

(*S*)-6-(1,1-Dioxidobenzoisothiazol-2(3*H*)-yl)-5-(((*R*)-4-methyl-1-phenylpent-1-yn-3-yl)amino)-6-oxohexanenitrile (17a)

Prepared according to the **GENERAL PROCEDURE C** with ($R_{S(IV)}$, $R_{C\text{-}1}$)-**S17a** (83.2 mg, 0.30 mmol, 99:1 d.r.) and **2k** (52.0 mg, 0.20 mmol) as substrates. Column chromatography: silica gel, petroleum ether/ethyl acetate/dichloromethane = 5:1:1. Colorless viscous oil (63.7 mg, 71% yield, >99:1 d.r.).

^{1}H NMR (400 MHz, $CDCl_3$) δ 7.80 (d, *J* = 7.8 Hz, 1H), 7.63 (t, *J* = 7.6 Hz, 1H), 7.57 (t, *J* = 7.7 Hz, 1H), 7.26 – 7.16 (m, 3H), 7.12 (t, *J* = 7.4 Hz, 1H), 7.02 (t, *J* = 7.6 Hz, 2H), 4.73 (d, *J* = 15.8 Hz, 1H), 4.41 (d, *J* = 15.8 Hz, 1H), 4.06 (d, *J* = 9.6 Hz, 1H), 3.49 (d, *J* = 5.5 Hz, 1H), 2.42 (hept, *J* = 7.9, 6.8 Hz, 2H), 2.25 (s, 1H), 1.96 (dddt, *J* = 32.7, 20.0, 13.4, 7.4 Hz, 4H), 1.66 (dt, *J* = 14.7, 8.5 Hz, 1H), 1.06 (d, *J* = 7.0 Hz, 6H).

^{13}C NMR (101 MHz, $CDCl_3$) δ 174.4, 134.1, 134.0, 131.5, 130.8, 129.7, 128.0, 127.9, 124.9, 123.2, 121.7, 119.7, 89.9, 85.9, 60.3, 57.0, 47.3, 33.4, 33.0, 22.4, 19.6, 18.2, 16.9.

HRMS (ESI, m/z): $[M+H]^+$ Calcd. For $C_{25}H_{28}N_3O_3S$: 450.1851; Found: 450.1853.

HPLC (Chiralpak OD-H Column), *i*-PrOH/hexane = 15/85, flow rate = 1.0 mL/min, λ = 210 nm; t_R = 13.9 min (major), t_R = 22.2 min (minor).

$[\alpha]_D^{25}$ = +32.9 (c = 0.5, $CHCl_3$).

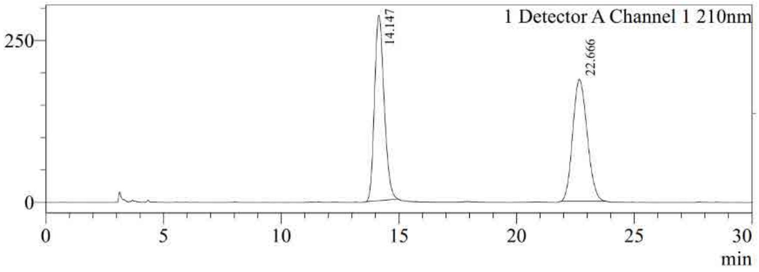

Detector A Channel 1 210nm

Peak#	Ret. Time	USP Width	Area	Height	Area%
1	14.147	0.752	8144948	286087	50.293
2	22.666	1.137	8050079	188472	49.707
Total			16195027	474558	100.000

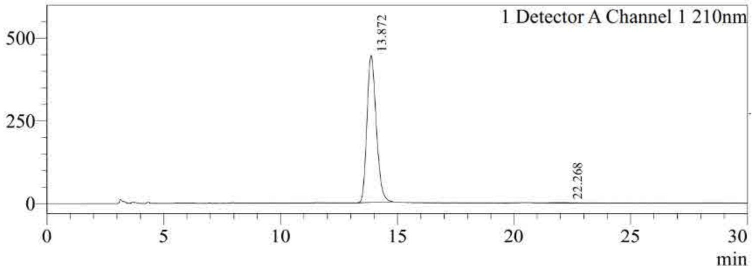

Detector A Channel 1 210nm

Peak#	Ret. Time	USP Width	Area	Height	Area%
1	13.872	0.724	12283236	444555	99.787
2	22.268	0.587	26168	838	0.213
Total			12309404	445394	100.000

Synthesis of ($S_{C\text{-}1}$, $R_{C\text{-}2}$)-17b

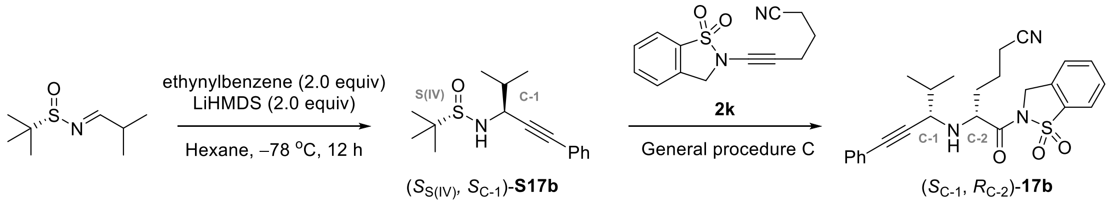

Synthesis of ($S_{S(IV)}$, $S_{C\text{-}1}$)-**S17b**

Under N_2 atmosphere, LiHMDS (4.0 mL, 1.0 M in THF, 2.0 equiv) was added dropwise to the solution of ethynylbenzene (408 mg, 4.0 mmol, 2.0 equiv) in hexane (24 mL) at –78 °C. The resulting mixture was then transferred to 0 °C and stirred for 10 minutes before the addition of (*S*)-sulfinimine (350 mg, 2.0 mmol, 1.0 equiv) dissolved in THF (20 mL) dropwise at –78 °C. The mixture was stirred at –78 °C overnight, and then quenched by saturated aqueous NH_4Cl. After extraction with ethyl acetate, the combined organic layer was washed with brine and concentrated under vacuo. The crude product was purified through flash chromatography (eluent: petroleum ether/ethyl acetate = 10:1 to 6:1) to afford ($S_{S(IV)}$, $S_{C\text{-}1}$)-**S17b** as colorless viscous oil (350 mg, 63% yield, >99:1 d.r.).

^{1}H NMR (400 MHz, $CDCl_3$) δ 7.44 – 7.42 (m, 2H), 7.29 – 7.26 (m, 3H), 4.13 (t, *J* = 5.7 Hz, 1H), 3.40 (d, *J* = 6.6 Hz, 1H), 2.06 – 1.98 (m, 1H), 1.24 (s, 9H), 1.06 (d, *J* = 6.8 Hz, 6H).

^{13}C NMR (101 MHz, $CDCl_3$) δ 131.9, 128.4, 128.3, 122.8, 87.6, 85.7, 56.3, 54.2, 33.8, 22.7, 19.2, 17.5.

HRMS (ESI, m/z): $[M+Na]^+$ Calcd. For $C_{16}H_{23}NONaS$: 300.1398; Found: 300.1401.

HPLC (Chiralpak OD-H Column), *i*-PrOH/hexane = 5/95, flow rate = 1.0 mL/min, λ = 210 nm; t_R = 5.5 min (minor), t_R = 7.6 min (major).

$[\alpha]_D^{25}$ = +26.4 (c = 0.5, $CHCl_3$).

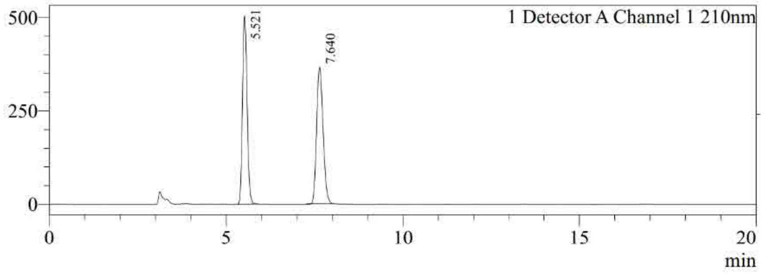

Detector A Channel 1 210nm

Peak#	Ret. Time	USP Width	Area	Height	Area%
1	5.521	0.248	4571205	503840	49.612
2	7.640	0.344	4642786	364856	50.388
Total			9213991	868696	100.000

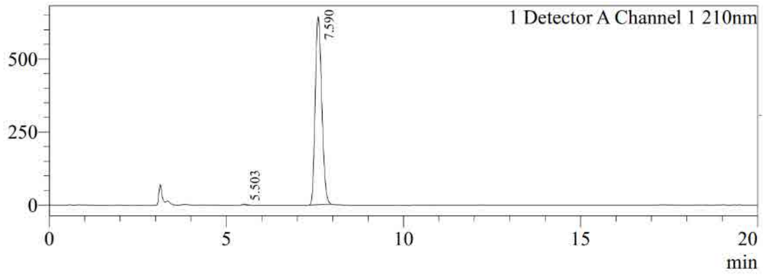

Detector A Channel 1 210nm

Peak#	Ret. Time	USP Width	Area	Height	Area%
1	5.503	0.157	9321	1729	0.112
2	7.590	0.348	8302456	644874	99.888
Total			8311777	646603	100.000

(*R*)-6-(1,1-Dioxidobenzoisothiazol-2(3*H*)-yl)-5-(((*S*)-4-methyl-1-phenylpent-1-yn-3-yl)amino)-6-oxohexanenitrilen (17b)

Prepared according to the **GENERAL PROCEDURE C** with ($S_{S(IV)}$, $S_{C\text{-}1}$)-**S17b** (83.2 mg, 0.30 mmol, >99:1 d.r.) and **2k** (52.0 mg, 0.20 mmol) as substrates. Column chromatography: silica gel, petroleum ether/ethyl acetate/dichloromethane = 5:1:1. Colorless viscous oil (81.1 mg, 90% yield, >99:1 d.r.).

^{1}H NMR (400 MHz, $CDCl_3$) δ 7.80 (d, *J* = 7.7 Hz, 1H), 7.63 (t, *J* = 7.5 Hz, 1H), 7.57 (t, *J* = 7.6 Hz, 1H), 7.27 – 7.18 (m, 3H), 7.12 (t, *J* = 7.5 Hz, 1H), 7.02 (t, *J* = 7.6 Hz, 2H), 4.73 (d, *J* = 15.9 Hz, 1H), 4.42 (d, *J* = 15.8 Hz, 1H), 4.07 (dd, *J* = 9.4, 3.2 Hz, 1H), 3.50 (d, *J* = 5.4 Hz, 1H), 2.43 (td, *J* = 7.1, 4.0 Hz, 2H), 2.22 (s, 1H), 2.12 – 1.84 (m, 4H), 1.74 – 1.61 (m, 1H), 1.06 (dd, *J* = 6.7, 3.2 Hz, 6H).

^{13}C NMR (101 MHz, $CDCl_3$) δ 174.4, 134.2, 134.1, 131.6, 130.9, 129.7, 128.0, 127.9, 125.0, 123.3, 121.8, 119.7, 89.9, 86.0, 60.4, 57.1, 47.4, 33.5, 33.1, 22.5, 19.6, 18.3, 17.0.

HRMS (ESI, m/z): $[M+H]^+$ Calcd. For $C_{25}H_{28}N_3O_3S$: 450.1851; Found: 450.1853.

HPLC (Chiralpak OD-H Column), *i*-PrOH/hexane = 15/85, flow rate = 1.0 mL/min, λ = 210 nm; t_R = 14.0 min (minor), t_R = 22.1 min (major).

$[\alpha]_D^{25}$ = –34.4 (c = 0.5, $CHCl_3$).

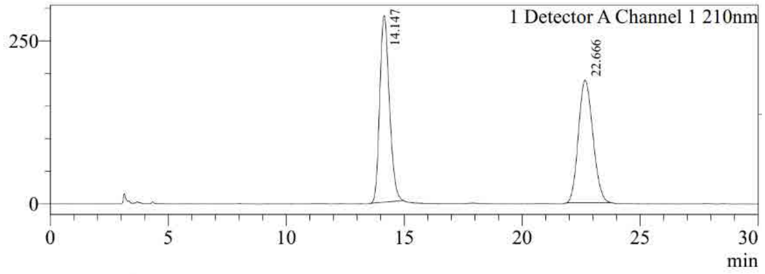

Detector A Channel 1 210nm

Peak#	Ret. Time	USP Width	Area	Height	Area%
1	14.147	0.752	8144948	286087	50.293
2	22.666	1.137	8050079	188472	49.707
Total			16195027	474558	100.000

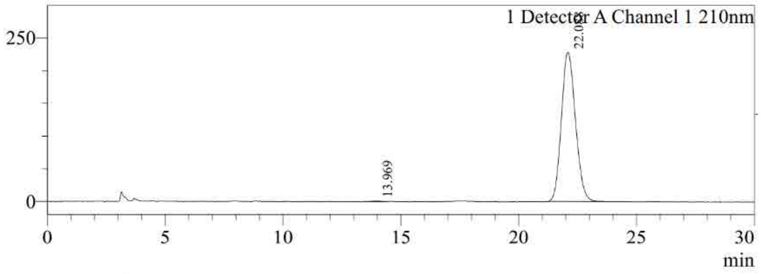

Detector A Channel 1 210nm

Peak#	Ret. Time	USP Width	Area	Height	Area%
1	13.969	0.701	30062	1294	0.316
2	22.088	1.101	9475500	228320	99.684
Total			9505562	229614	100.000

Synthesis of ($S_{C\text{-}1}$, $S_{C\text{-}2}$)-18a

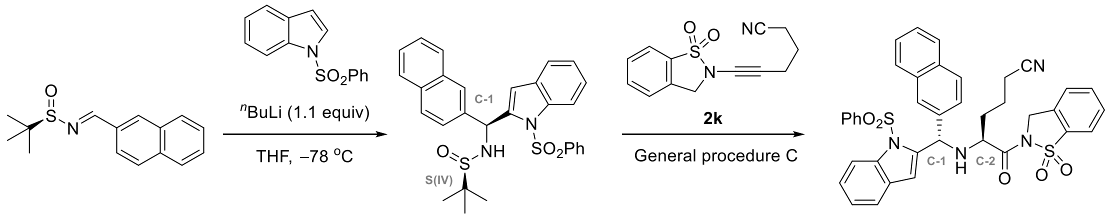

Synthesis of ($R_{S(IV)}$, $S_{C\text{-}1}$)**-S18a** (*42*)

Under N_2 atmosphere, ^{n}BuLi (1.4 mL, 1.6 M in THF, 1.1 equiv) was added dropwise to the solution of *N*-phenylsulfonyl indole (529 mg, 2.0 mmol, 1.0 equiv) in THF (0.20 M) at –78 °C. The resulting mixture was stirred at this temperature for 1 h and then tansferred to the stirred solution of (*R*)-sulfinimine (467 mg, 1.8 mmol, 0.9 equiv) in THF (0.20 M) at –78 °C. The mixture was stirred continuely at –78 °C for 2 h, and then quenched by

saturated aqueous NH_4Cl. After extraction with ethyl acetate, the combined organic layer was washed with brine and concentrated under vacuo. The crude product was purified through flash chromatography (eluent: petroleum ether/ethyl acetate = 4:1 to 2:1) to afford ($R_{S(IV)}$, S_{C-1})-**S18a** as white solid (715 mg, 77% yield, >99:1 d.r.).

^{1}H NMR (400 MHz, $CDCl_3$) δ 8.18 (d, J = 8.2 Hz, 1H), 7.81 – 7.78 (m, 2H), 7.66 – 7.64 (m, 2H), 7.59 – 7.54 (m, 2H), 7.49 – 7.40 (m, 4H), 7.36 – 7.26 (m, 3H), 7.07 – 7.03 (m, 3H), 6.72 (s, 1H), 3.76 (s, 1H), 1.27 (s, 9H).

HPLC (Chiralpak OD-H Column), *i*-PrOH/hexane = 10/90, flow rate = 1.0 mL/min, λ = 210 nm; t_R = 12.7 min (major), t_R = 15.1 min (minor).

$[\alpha]_D^{20}$ = +9.00 (c = 1.2, $CHCl_3$).

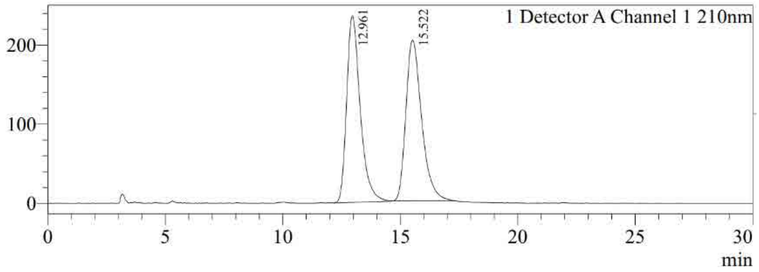

Detector A Channel 1 210nm

Peak#	Ret. Time	USP Width	Area	Height	Area%
1	12.961	1.022	9461200	235894	49.901
2	15.522	1.198	9498689	203287	50.099
Total			18959888	439181	100.000

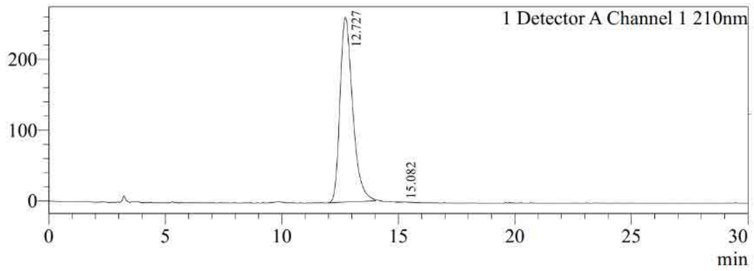

Detector A Channel 1 210nm

Peak#	Ret. Time	USP Width	Area	Height	Area%
1	12.727	0.971	9855083	261036	100.000
2	15.082	0.105	46	111	0.000
Total			9855130	261148	100.000

(*S*)-6-(1,1-Dioxidobenzoisothiazol-2(3*H*)-yl)-5-(((*S*)-naphthalen-2-yl(1-(phenylsulfonyl)-1*H*-indol-2-yl)methyl)amino)-6-oxohexanenitrile (18a)

Prepared according to the **GENERAL PROCEDURE C** with ($R_{S(IV)}$, S_{C-1})-**S18a** (155 mg, 0.30 mmol, >99:1 d.r.) and **2k** (52.0 mg, 0.20 mmol) as substrates (*Note: Workup with 6M HCl (1.0 mL) and 50 mol% HNTf$_2$ overnight*). Column chromatography: silica gel, petroleum ether/ethyl acetate/dichloromethane = 3:1:1. White solid (112.5 mg, 81% yield, 99:1 d.r.).

^{1}H NMR (400 MHz, $CDCl_3$) δ 8.11 (d, J = 7.9 Hz, 1H), 7.80 – 7.70 (m, 3H), 7.66 (dd, J = 13.4, 5.7 Hz, 2H), 7.62 – 7.52 (m, 3H), 7.43 (t, J = 9.1 Hz, 2H), 7.34 (t, J = 8.0 Hz, 2H), 7.29 (d, J = 8.2 Hz, 3H), 7.15 – 7.07 (m, 2H), 6.89 (t, J = 7.8 Hz, 2H), 5.91 (s, 1H), 4.75 (d, J = 15.7 Hz, 1H), 4.54 (d, J = 15.7 Hz, 1H), 4.48 – 4.32 (m, 1H), 2.43 (q, J = 7.2 Hz, 2H), 2.09 (dqt, J = 25.2, 16.3, 7.3 Hz, 3H), 1.75 (h, J = 7.8 Hz, 1H).

^{13}C NMR (101 MHz, $CDCl_3$) δ 173.7, 142.5, 138.8, 138.0, 137.8, 134.2, 134.0, 133.1, 133.0, 130.8, 129.7, 129.2, 128.6, 128.3, 128.1, 127.9, 127.5, 126.4, 126.3, 126.2, 126.0, 125.1, 124.7, 123.7, 121.8, 121.1, 119.6, 114.9, 110.9, 60.0, 59.5, 47.3, 33.0, 22.3, 17.0.

HRMS (ESI, m/z): $[M+H]^+$ Calcd. For $C_{38}H_{33}N_4O_5S_2$: 689.1892; Found: 689.1890.

HPLC (Chiralpak OD-H Column), *i*-PrOH/hexane = 20/80, flow rate = 1.0 mL/min, λ = 254 nm; t_R = 45.3 min (major), t_R = 49.8 min (minor).

$[\alpha]_D^{25}$ = –61.0 (c = 0.4, $CHCl_3$).

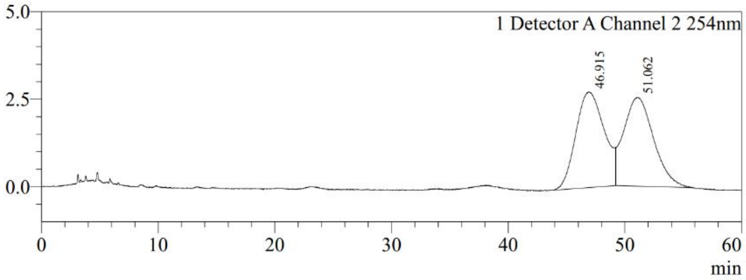

Detector A Channel 2 254nm

Peak#	Ret. Time	USP Width	Area	Height	Area%
1	46.915	4.786	466105	2738	50.666
2	51.062	4.808	453846	2539	49.334
Total			919950	5277	100.000

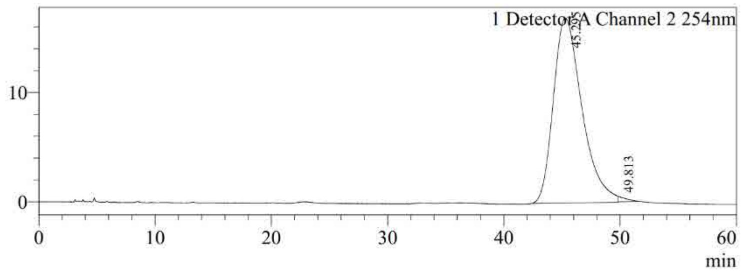

Detector A Channel 2 254nm

Peak#	Ret. Time	USP Width	Area	Height	Area%
1	45.295	4.377	2891490	16930	99.144
2	49.813	--	24959	520	0.856
Total			2916448	17451	100.000

Synthesis of ($R_{C\text{-}1}$, $R_{C\text{-}2}$)-18b

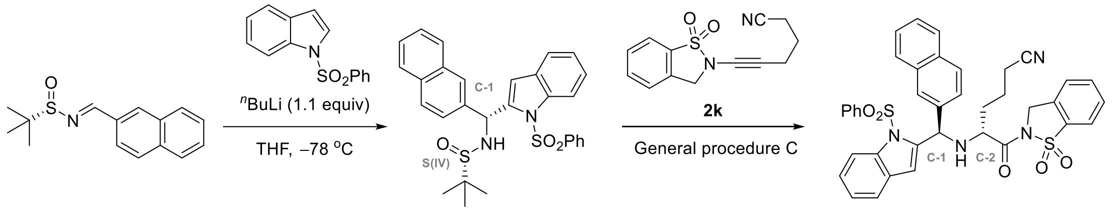

($S_{S(IV)}$, $R_{C\text{-}1}$)-**S18b** ($R_{C\text{-}1}$, $R_{C\text{-}2}$)-**18b**

Synthesis of ($S_{S(IV)}$, $R_{C\text{-}1}$)**-S18b**

Under N_2 atmosphere, ^{n}BuLi (1.4 mL, 1.6 M in THF, 1.1 equiv) was added dropwise to the solution of *N*-phenylsulfonyl indole (529 mg, 2.0 mmol, 1.0 equiv) in THF (0.20 M) at −78 °C The resulting mixture was stirred

at this temperature for 1 h and then tansferred to the stirred solution of (*S*)-sulfinimine (467 mg, 1.8 mmol, 0.9 equiv) in THF (0.20 M) at −78 °C. The mixture was stirred continuely at −78 °C for 2 h, and then quenched by saturated aqueous NH_4Cl. After extraction with ethyl acetate, the combined organic layer was washed with brine and concentrated under vacuo. The crude product was purified through flash chromatography (eluent: petroleum ether/ethyl acetate = 4:1 to 2:1) to afford ($S_{S(IV)}$, $R_{C\text{-}1}$)-**S18b** as white solid (710 mg, 76% yield, >99:1 d.r.).

^{1}H NMR (400 MHz, $CDCl_3$) δ 8.18 (d, *J* = 8.3 Hz, 1H), 7.81 – 7.78 (m, 2H), 7.65 (d, *J* = 8.4 Hz, 2H), 7.58 – 7.54 (m, 2H), 7.49 – 7.40 (m, 4H), 7.36 – 7.24 (m, 3H), 7.08 – 7.03 (m, 3H), 6.72 (s, 1H), 3.75 (s, 1H), 1.27 (s, 9H).

^{13}C NMR (101 MHz, $CDCl_3$) δ 141.7, 138.4, 137.8, 137.6, 133.5, 133.3, 133.2, 129.2, 128.9, 128.9, 128.4, 127.7, 126.6, 126.4, 126.3, 125.0, 124.0, 121.2, 115.2, 112.2, 56.4, 22.8.

HRMS (ESI, m/z): $[M+Na]^+$ Calcd. For $C_{29}H_{28}N_2O_3NaS_2$: 539.1439; Found: 539.1442.

HPLC (Chiralpak OD-H Column), *i*-PrOH/hexane = 10/90, flow rate = 1.0 mL/min, λ = 210 nm; t_R = 12.9 min (minor), t_R = 15.2 min (major).

$[\alpha]_D^{20}$ = −15.0 (c = 1.2, $CHCl_3$).

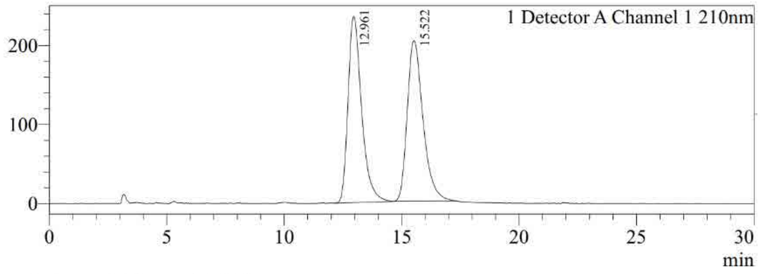

Detector A Channel 1 210nm

Peak#	Ret. Time	USP Width	Area	Height	Area%
1	12.961	1.022	9461200	235894	49.901
2	15.522	1.198	9498689	203287	50.099
Total			18959888	439181	100.000

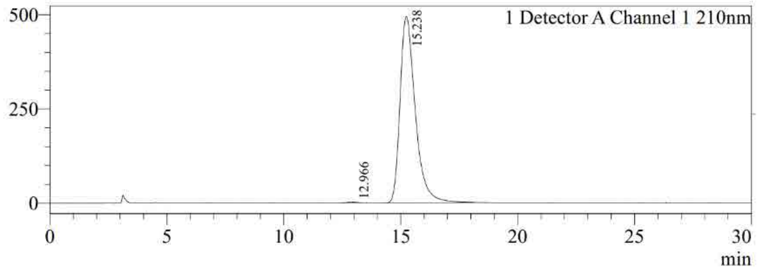

Detector A Channel 1 210nm

Peak#	Ret. Time	USP Width	Area	Height	Area%
1	12.966	0.684	52664	1780	0.235
2	15.238	1.137	22351920	493954	99.765
Total			22404585	495734	100.000

(*R*)-6-(1,1-Dioxidobenzoisothiazol-2(3*H*)-yl)-5-(((*R*)-naphthalen-2-yl(1-(phenylsulfonyl)-1*H*-indol-2-yl)methyl)amino)-6-oxohexanenitrile (18b)

Prepared according to the **GENERAL PROCEDURE C** with ($S_{S(IV)}$, $R_{C\text{-}1}$)-**S18b** (155 mg, 0.30 mmol, >99:1 d.r.) and **2k** (52.0 mg, 0.20 mmol) as substrates (*Note: Workup with 6M HCl (1.0 mL) and 50 mol% HNTf$_2$ overnight*). Column chromatography: silica gel, petroleum ether/ethyl acetate/dichloromethane = 3:1:1. White solid (113.7 mg, 82% yield, >99:1 d.r.).

^{1}H NMR (400 MHz, $CDCl_3$) δ 8.11 (d, J = 7.9 Hz, 1H), 7.74 (dt, J = 13.8, 8.0 Hz, 3H), 7.69 – 7.64 (m, 2H), 7.61 – 7.52 (m, 3H), 7.43 (t, J = 7.4 Hz, 2H), 7.37 – 7.27 (m, 6H), 7.16 – 7.05 (m, 2H), 6.89 (t, J = 7.7 Hz, 2H), 5.89 (s, 1H), 4.76 (d, J = 15.7 Hz, 1H), 4.54 (d, J = 15.7 Hz, 1H), 4.41 (s, 1H), 2.51 – 2.34 (m, 2H), 2.22 – 1.95 (m, 3H), 1.82 – 1.66 (m, 1H).

^{13}C NMR (101 MHz, $CDCl_3$) δ 173.8, 142.6, 138.9, 138.0, 137.9, 134.3, 134.1, 133.2, 133.1, 133.0, 130.8, 129.8, 129.2, 128.7, 128.3, 128.1, 127.9, 127.6, 126.4, 126.3, 126.2, 126.0, 125.1, 124.7, 123.7, 121.9, 121.1, 119.6, 114.9, 110.9, 60.0, 59.5, 47.3, 33.1, 22.4, 17.0.

HRMS (ESI, m/z): $[M+H]^+$ Calcd. For $C_{38}H_{33}N_4O_5S_2$: 689.1892; Found: 689.1895.

HPLC (Chiralpak OD-H Column), *i*-PrOH/hexane = 20/80, flow rate = 1.0 mL/min, λ = 254 nm; t_R = 45.5 min (minor), t_R = 49.5 min (major).

$[\alpha]_D^{25}$ = +60.0 (c = 0.4, $CHCl_3$).

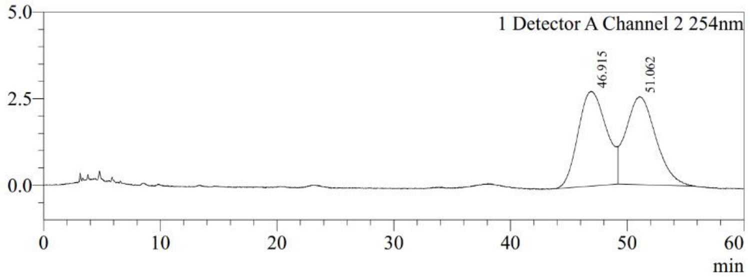

Detector A Channel 2 254nm

Peak#	Ret. Time	USP Width	Area	Height	Area%
1	46.915	4.786	466105	2738	50.666
2	51.062	4.808	453846	2539	49.334
Total			919950	5277	100.000

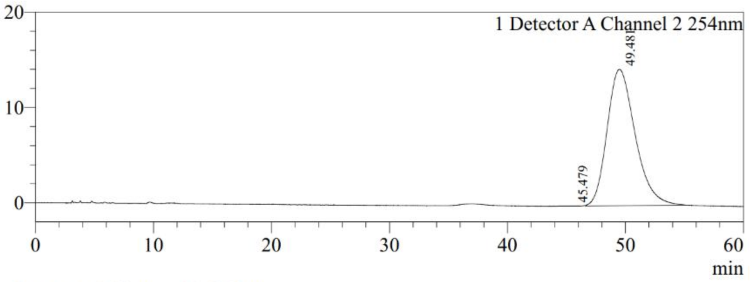

Detector A Channel 2 254nm

Peak#	Ret. Time	USP Width	Area	Height	Area%
1	45.479	0.145	603	9	0.025
2	49.481	4.300	2367480	14304	99.975
Total			2368083	14313	100.000

Synthesis of ($R_{C\text{-}1}$, $S_{C\text{-}2}$)-19a

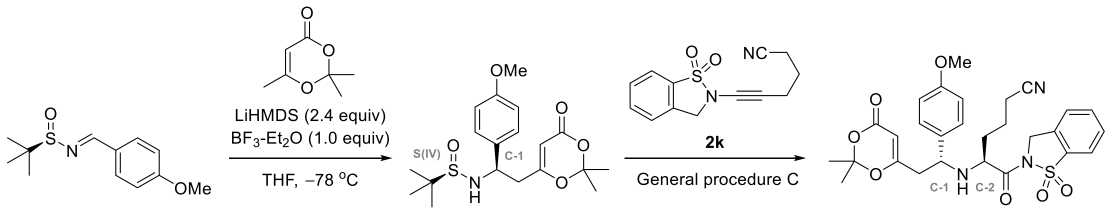

($R_{S(IV)}$, $R_{C\text{-}1}$)-**S19a**   ($R_{C\text{-}1}$, $S_{C\text{-}2}$)-**19a**

Synthesis of ($R_{S(IV)}$, $R_{C\text{-}1}$)-S19a

Under N_2 atmosphere, LiHMDS (2.4 mL, 1.0 M in THF, 2.4 equiv) was added dropwise to the solution of Diketton acetone adduct (284 mg, 2.0 mmol, 2.0 equiv) in THF (0.20 M) at −78 °C The resulting mixture was stirred at this temperature for 1 h and then tansferred to the stirred solution of (*R*)-sulfinimine (239 mg, 1.0 mmol, 1.0 equiv) and BF_3-Et_2O (0.13 mL, 46.5% wt, 1.0 equiv) in THF (0.10 M) at −78 °C. The mixture was stirred continuely at −78 °C for 3 h, and then quenched by saturated aqueous NH_4Cl. After extraction with ethyl acetate, the combined organic layer was washed with brine and concentrated under vacuo. The crude product was purified through flash chromatography (eluent: petroleum ether/ethyl acetate = 2:1 to 1:1) to afford ($R_{S(IV)}$, $R_{C\text{-}1}$)-**S19a** as yellow viscous oil (200 mg, 52% yield, >99:1 d.r.).

^{1}H NMR (400 MHz, $CDCl_3$) δ 7.22 (d, *J* = 8.7 Hz, 2H), 6.85 (d, *J* = 8.7 Hz, 2H), 5.08 (s, 1H), 4.63 (ddd, *J* = 8.7, 6.4, 3.9 Hz, 1H), 3.77 (s, 3H), 3.46 (d, *J* = 3.8 Hz, 1H), 2.95 (dd, *J* = 14.3, 6.3 Hz, 1H), 2.62 (dd, *J* = 14.3, 8.6 Hz, 1H), 1.58 (s, 3H), 1.47 (s, 3H), 1.18 (s, 9H).

^{13}C NMR (101 MHz, $CDCl_3$) δ 167.7, 160.8, 159.6, 132.2, 128.3, 114.2, 106.6, 95.5, 56.0, 55.7, 55.2, 55.2, 41.0, 25.5, 24.5, 22.5, 22.1.

HRMS (ESI, m/z): $[M+Na]^+$ Calcd. For $C_{19}H_{27}NO_5NaS$: 404.1508; Found: 404.1511.

HPLC (Chiralpak OD-H Column), *i*-PrOH/hexane = 10/90, flow rate = 1.0 mL/min, λ = 254 nm; t_R = 16.2 min (major), t_R = 24.0 min (minor).

$[\alpha]_D^{20}$ = −56.3 (c = 1.1, $CHCl_3$).

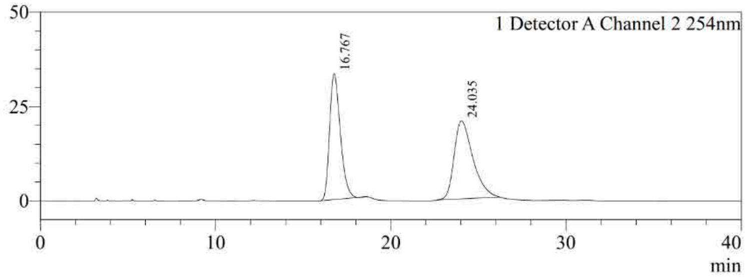

Detector A Channel 2 254nm

Peak#	Ret. Time	USP Width	Area	Height	Area%
1	16.767	1.119	1420734	33349	47.957
2	24.035	1.931	1541800	20642	52.043
Total			2962534	53992	100.000

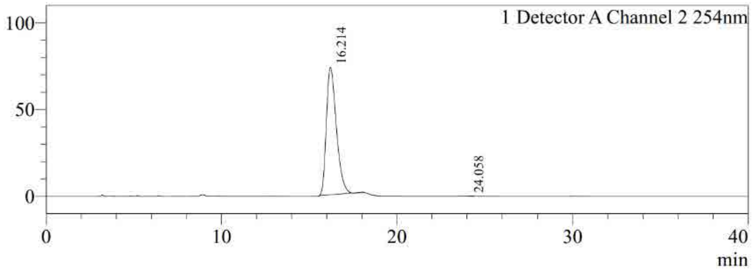

Detector A Channel 2 254nm

Peak#	Ret. Time	USP Width	Area	Height	Area%
1	16.214	1.044	2913483	73547	99.990
2	24.058	0.410	294	10	0.010
Total			2913776	73556	100.000

(*S*)-5-(((*R*)-2-(2,2-Dimethyl-4-oxo-4*H*-1,3-dioxin-6-yl)-1-(4-methoxyphenyl)ethyl)amino)-6-(1,1-dioxidobenzoisothiazol-2(3*H*)-yl)-6-oxohexanenitrile (19a)

Prepared according to the **GENERAL PROCEDURE C** with ($R_{S(IV)}$, $R_{C\text{-}1}$)-**S19a** (114 mg, 0.30 mmol, >99:1 d.r.) and **2k** (52.0 mg, 0.20 mmol) as substrates. Column chromatography: silica gel, petroleum ether/ethyl acetate/dichloromethane = 2:1:1. Light-yellow solid (57.5 mg, 52% yield, >99:1 d.r.).

^{1}H NMR (400 MHz, $CDCl_3$) δ 7.81 (d, *J* = 7.9 Hz, 1H), 7.72 (t, *J* = 7.7 Hz, 1H), 7.61 (t, *J* = 7.7 Hz, 1H), 7.44 (d, *J* = 7.8 Hz, 1H), 7.17 (d, *J* = 8.2 Hz, 2H), 6.75 (d, *J* = 8.1 Hz, 2H), 5.13 (s, 1H), 4.75 (d, *J* = 15.9 Hz, 1H), 4.55 (d, *J* = 15.9 Hz, 1H), 4.13 – 4.00 (m, 1H), 3.92 – 3.83 (m, 1H), 3.70 (s, 3H), 2.85 (dd, *J* = 14.4, 6.2 Hz, 1H), 2.52 (dd, *J* = 14.4, 8.8 Hz, 1H), 2.38 (t, *J* = 7.0 Hz, 2H), 2.19 (s, 1H), 2.04 – 1.76 (m, 3H), 1.64 – 1.54 (m, 1H), 1.50 (s, 3H), 1.44 (s, 3H).

^{13}C NMR (101 MHz, $CDCl_3$) δ 173.6, 168.9, 161.3, 159.3, 134.4, 133.9, 133.1, 130.7, 129.9, 128.8, 125.1, 121.9, 119.6, 113.7, 106.5, 95.0, 59.3, 59.2, 55.3, 47.2, 40.7, 33.1, 25.1, 24.8, 22.2, 17.0.

HRMS (ESI, m/z): $[M+Na]^+$ Calcd. For $C_{28}H_{31}N_3O_7NaS$: 576.1780; Found: 576.1778.

HPLC (Chiralpak OD-H Column), *i*-PrOH/hexane = 30/70, flow rate = 1.0 mL/min, λ = 254 nm; t_R = 26.0 min (major), t_R = 33.2 min (minor).

$[\alpha]_D^{25}$ = –8.40 (c = 0.5, $CHCl_3$).

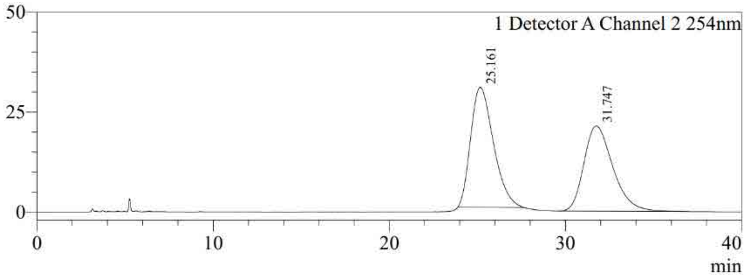

Detector A Channel 2 254nm

Peak#	Ret. Time	USP Width	Area	Height	Area%
1	25.161	2.409	2714178	29975	52.697
2	31.747	2.978	2436342	21287	47.303
Total			5150520	51262	100.000

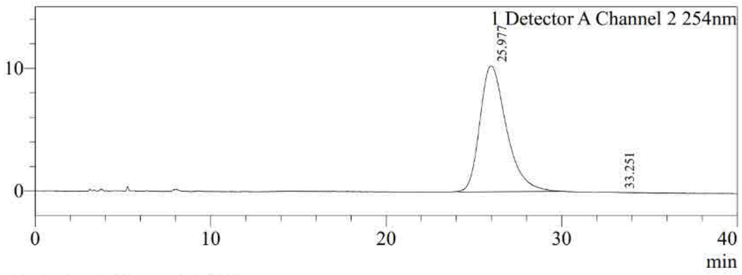

Detector A Channel 2 254nm

Peak#	Ret. Time	USP Width	Area	Height	Area%
1	25.977	2.716	1081660	10276	99.957
2	33.251	0.154	468	25	0.043
Total			1082128	10302	100.000

Synthesis of ($S_{C\text{-}1}$, $R_{C\text{-}2}$)-19b

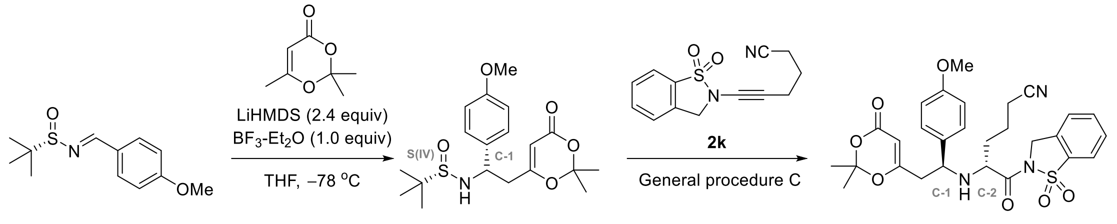

($S_{S(IV)}$, $S_{C\text{-}1}$)-**S19b** ($S_{C\text{-}1}$, $R_{C\text{-}2}$)-**19b**

Synthesis of ($S_{S(IV)}$, $S_{C\text{-}1}$)-**S19b** (*43*)

Under N_2 atmosphere, LiHMDS (7.2 mL, 1.0 M in THF, 2.4 equiv) was added dropwise to the solution of Diketton acetone adduct (853 mg, 6.0 mmol, 2.0 equiv) in THF (0.20 M) at −78 °C The resulting mixture was stirred at this temperature for 1 h and then tansferred to the stirred solution of (*S*)-sulfinimine (718 mg, 3.0 mmol, 1.0 equiv) and BF_3-Et_2O (0.39 mL, 46.5% wt, 1.0 equiv) in THF (0.10 M) at −78 °C. The mixture was stirred continuely at −78 °C for 3 h, and then quenched by saturated aqueous NH_4Cl. After extraction with ethyl acetate, the combined organic layer was washed with brine and concentrated under vacuo. The crude product was purified through flash chromatography (eluent: petroleum ether/ethyl acetate = 2:1 to 1:1) to afford ($S_{S(IV)}$, $S_{C\text{-}1}$)-**S19b** as yellow viscous oil (666 mg, 58% yield, >99:1 d.r.).

^{1}H NMR (400 MHz, $CDCl_3$) δ 7.23 (d, *J* = 8.3 Hz, 2H), 6.85 (d, *J* = 8.2 Hz, 2H), 5.08 (s, 1H), 4.64 (ddd, *J* = 9.6, 6.2, 3.6 Hz, 1H), 3.77 (s, 3H), 3.47 (d, *J* = 3.9 Hz, 1H), 2.96 (dd, *J* = 14.3, 6.3 Hz, 1H), 2.62 (dd, *J* = 14.3, 8.6 Hz, 1H), 1.59 (s, 3H), 1.48 (s, 3H), 1.19 (s, 9H).

HPLC (Chiralpak OD-H Column), *i*-PrOH/hexane = 10/90, flow rate = 1.0 mL/min, λ = 254 nm; t_R = 16.4 min (minor), t_R = 23.0 min (major).

[α]$_D^{20}$ = +67.0 (c = 1.1, $CHCl_3$).

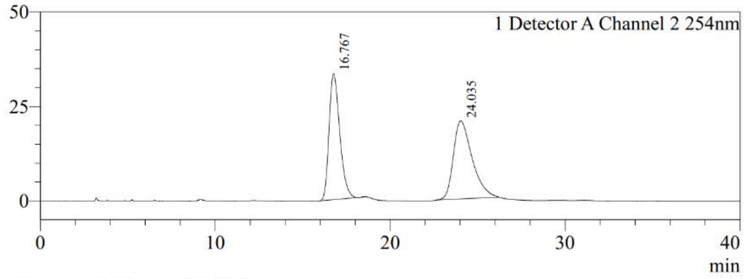

Detector A Channel 2 254nm

Peak#	Ret. Time	USP Width	Area	Height	Area%
1	16.767	1.119	1420734	33349	47.957
2	24.035	1.931	1541800	20642	52.043
Total			2962534	53992	100.000

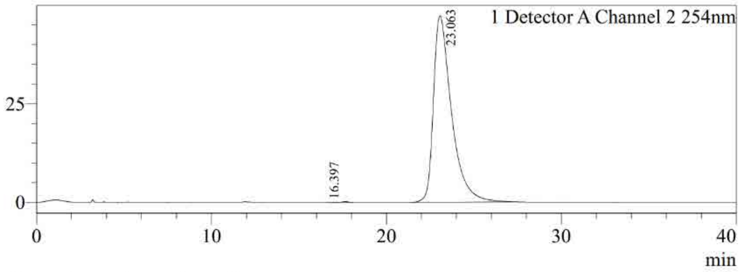

Detector A Channel 2 254nm

Peak#	Ret. Time	USP Width	Area	Height	Area%
1	16.397	0.125	3310	2	0.097
2	23.063	1.778	3403273	47276	99.903
Total			3406584	47278	100.000

(*R*)-5-(((*S*)-2-(2,2-Dimethyl-4-oxo-4*H*-1,3-dioxin-6-yl)-1-(4-methoxyphenyl)ethyl)amino)-6-(1,1-dioxidobenzoisothiazol-2(3*H*)-yl)-6-oxohexanenitrile (19b)

Prepared according to the **GENERAL PROCEDURE C** with ($S_{S(IV)}$, $S_{C\text{-}1}$)-**S19b** (114 mg, 0.30 mmol, >99:1 d.r.) and **2k** (52.0 mg, 0.20 mmol) as substrates. Column chromatography: silica gel, petroleum ether/ethyl acetate/dichloromethane = 2:1:1. Light-yellow solid (77.2 mg, 70% yield, >99:1 d.r.).

^{1}H NMR (400 MHz, $CDCl_3$) δ 7.81 (d, *J* = 7.9 Hz, 1H), 7.72 (t, *J* = 7.6 Hz, 1H), 7.61 (t, *J* = 7.7 Hz, 1H), 7.44 (d, *J* = 7.8 Hz, 1H), 7.17 (d, *J* = 8.1 Hz, 2H), 6.75 (d, *J* = 8.1 Hz, 2H), 5.13 (s, 1H), 4.75 (d, *J* = 15.9 Hz, 1H), 4.54 (d, *J* = 15.9 Hz, 1H), 4.08 (d, *J* = 9.0 Hz, 1H), 3.87 (t, *J* = 7.4 Hz, 1H), 3.70 (s, 3H), 2.84 (dd, *J* = 14.4, 6.2 Hz, 1H), 2.52 (dd, *J* = 14.3, 8.7 Hz, 1H), 2.38 (t, *J* = 7.0 Hz, 2H), 2.16 (s, 2H), 1.90 (tdd, *J* = 24.6, 12.0, 7.0 Hz, 3H), 1.66 – 1.54 (m, 1H), 1.50 (s, 3H), 1.45 (s, 3H).

^{13}C NMR (101 MHz, $CDCl_3$)δ 173.6, 169.0, 161.3, 159.3, 134.5, 133.9, 133.1, 130.7, 129.9, 128.8, 125.2, 121.9, 119.6, 113.7, 106.5, 95.0, 59.4, 59.2, 55.3, 47.2, 40.7, 33.1, 25.1, 24.8, 22.2, 17.0.

HRMS (ESI, m/z): $[M+Na]^+$ Calcd. For $C_{28}H_{31}N_3O_7NaS$: 576.1780; Found: 576.1781.

HPLC (Chiralpak OD-H Column), *i*-PrOH/hexane = 30/70, flow rate = 1.0 mL/min, λ = 254 nm; t_R = 26.2 min (minor), t_R = 33.0 min (major).

$[\alpha]_D^{25}$ = +5.20 (c = 0.5, $CHCl_3$).

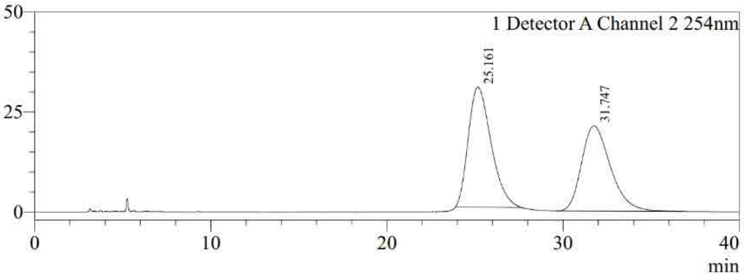

Detector A Channel 2 254nm

Peak#	Ret. Time	USP Width	Area	Height	Area%
1	25.161	2.409	2714178	29975	52.697
2	31.747	2.978	2436342	21287	47.303
Total			5150520	51262	100.000

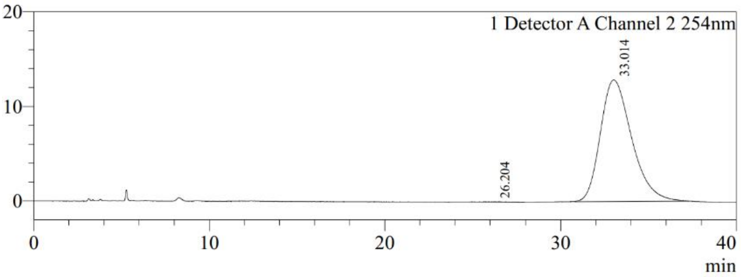

Detector A Channel 2 254nm

Peak#	Ret. Time	USP Width	Area	Height	Area%
1	26.204	2.051	3019	30	0.188
2	33.014	3.249	1600605	12875	99.812
Total			1603624	12905	100.000

Synthesis of (*R*$_{\text{C-1}}$, *S*$_{\text{C-2}}$)-20a

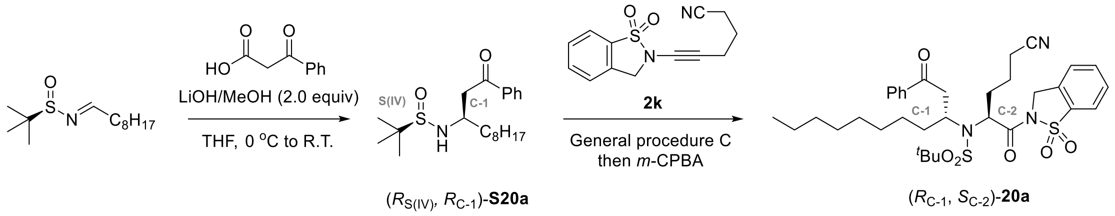

Synthesis of (*R*$_{\text{S(IV)}}$, *R*$_{\text{C-1}}$)-**S20a** (*44*)

Under N_2 atmosphere, LiOH in MeOH (2.0 mL, 2.0 M in MeOH, 2.0 equiv) was added dropwise to the solution of *β*-keto acid (492 mg, 3.0 mmol, 1.5 equiv) in THF (20 mL) at 0 °C. The resulting mixture was warmed to room temperature, followed by addition of (*R*)-sulfinimine (490 mg, 2.0 mmol, 1.0 equiv) in THF (2.0 mL). The mixture was stirred continuely for 1 h and then quenched with water. After extraction with ethyl acetate, the combined organic layer was washed with brine and concentrated under vacuo. The crude product was purified through flash chromatography (eluent: petroleum ether/ethyl acetate = 5:1) to afford (*R*$_{\text{S(IV)}}$, *R*$_{\text{C-1}}$)-**S20a** as yellow oil (422 mg, 57% yield, >99:1 d.r.). Known compound.

^{1}H NMR (400 MHz, $CDCl_3$) δ 7.92 (d, *J* = 7.7 Hz, 2H), 7.53 (t, *J* = 7.4 Hz, 1H), 7.43 (t, *J* = 7.6 Hz, 2H), 4.10 (d, *J* = 8.7 Hz, 1H), 3.73 – 3.69 (m, 1H), 3.41 – 3.29 (m, 2H), 1.68 – 1.52 (m, 2H), 1.45 – 1.23 (m, 12H), 1.19 (s, 9H), 0.84 (t, *J* = 6.6 Hz, 3H).

HPLC (Chiralpak OD-H Column), *i*-PrOH/hexane = 3/97, flow rate = 1.0 mL/min, λ = 210 nm; t_R = 9.6 min (minor), t_R = 10.6 min (major).

[α]$_D^{20}$ = –42.0 (c = 1.1, $CHCl_3$).

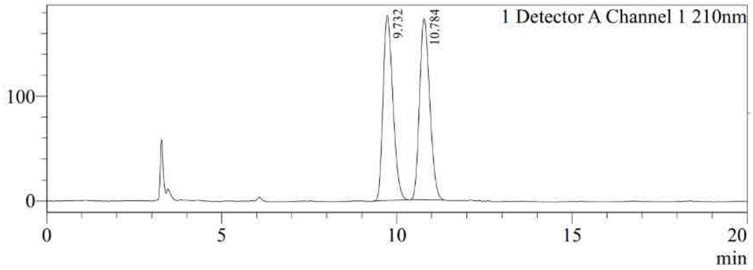

Detector A Channel 1 210nm

Peak#	Ret. Time	USP Width	Area	Height	Area%
1	9.732	0.520	3442276	176748	49.503
2	10.784	0.544	3511444	172801	50.497
Total			6953720	349549	100.000

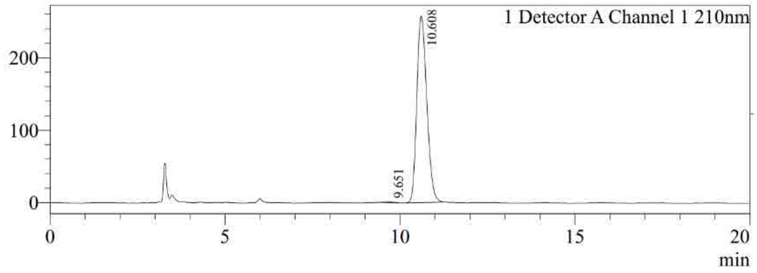

Detector A Channel 1 210nm

Peak#	Ret. Time	USP Width	Area	Height	Area%
1	9.651	0.403	14755	874	0.288
2	10.608	0.530	5104437	257594	99.712
Total			5119192	258468	100.000

***N*-((*S*)-5-Cyano-1-(1,1-dioxidobenzoisothiazol-2(*3H*)-yl)-1-oxopentan-2-yl)-2-methyl-*N*-((*R*)-1-oxo-1-phenylundecan-3-yl)propane-2-sulfonamide (20a)**

Prepared according to the **GENERAL PROCEDURE C** with ($R_{S(IV)}$, $R_{C\text{-}1}$)-^{t}BS-**13a** (110 mg, 0.30 mmol, >99:1 d.r.) and **2k** (52.0 mg, 0.20 mmol) as substrates *(Note: Workup with m-CPBA (345 mg, 2.0 mmol, 10 equiv) instead of 6M HCl after the rearrangement reaction)*. Column chromatography: silica gel, petroleum ether/ethyl acetate = 8:1 to 4:1. White solid (101.2 mg, 77% yield, 99:1 d.r.).

^{1}H NMR (400 MHz, $CDCl_3$) δ 8.00 (d, *J* = 7.8 Hz, 2H), 7.81 (d, *J* = 7.9 Hz, 1H), 7.74 (t, *J* = 7.6 Hz, 1H), 7.61 (t, *J* = 7.4 Hz, 1H), 7.57 (d, *J* = 7.2 Hz, 1H), 7.48 (q, *J* = 8.3, 7.7 Hz, 3H), 5.22 – 5.01 (m, 1H), 5.00 – 4.75 (m, 1H), 4.70 – 4.51 (m, 1H), 3.94 – 3.64 (m, 1H), 3.50 – 3.19 (m, 1H), 2.50 – 2.14 (m, 5H), 2.08 – 1.77 (m, 3H), 1.40 (s, 9H), 1.30 – 1.11 (m, 11H), 1.08 – 0.94 (m, 1H), 0.84 (t, *J* = 6.9 Hz, 3H).

^{13}C NMR (101 MHz, $CDCl_3$) δ 198.0, 170.5, 136.5, 134.5, 134.2, 133.6, 130.9, 130.0, 128.9, 128.3, 125.2, 121.9, 119.4, 63.4, 62.8, 55.2, 47.8, 38.0, 31.9, 29.7, 29.6, 29.3, 27.4, 25.7, 23.2, 22.7, 16.6, 14.2.

HRMS (ESI, m/z): $[M+Na]^+$ Calcd. For $C_{34}H_{47}N_3O_6NaS_2$: 680.2804; Found: 680.2806.

HPLC (Chiralpak OD-H Column), *i*-PrOH/hexane = 30/70, flow rate = 1.0 mL/min, λ = 210 nm; t_R = 8.9 min (minor), t_R = 11.9 min (major).

$[\alpha]_D^{25}$ = –6.80 (c = 0.5, $CHCl_3$).

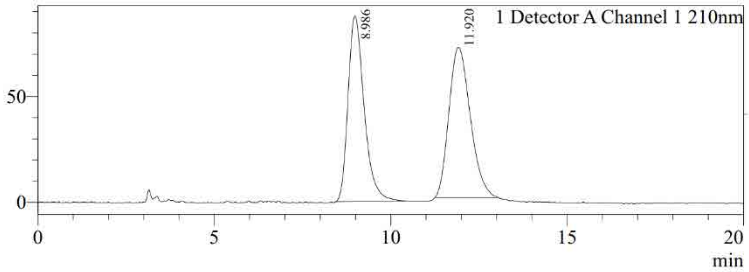

Detector A Channel 1 210nm

Peak#	Ret. Time	USP Width	Area	Height	Area%
1	8.986	0.808	2785473	87602	48.271
2	11.920	1.113	2984987	70908	51.729
Total			5770460	158510	100.000

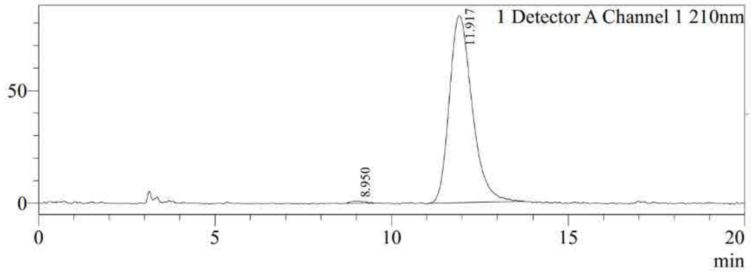

Detector A Channel 1 210nm

Peak#	Ret. Time	USP Width	Area	Height	Area%
1	8.950	0.614	28200	1097	0.748
2	11.917	1.151	3741072	83006	99.252
Total			3769272	84103	100.000

Synthesis of (*S*C-1, *R*C-2)-20b

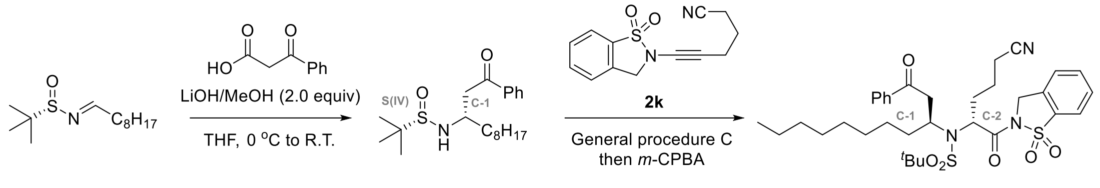

($S_{S(IV)}$, S_{C-1})-**S20b**

(S_{C-1}, R_{C-2})-**20b**

Synthesis of ($S_{S(IV)}$, S_{C-1})-**S20b**

Under N_2 atmosphere, LiOH in MeOH (2.0 mL, 2.0 M in MeOH, 2.0 equiv) was added dropwise to the solution of *β*-keto acid (492 mg, 3.0 mmol, 1.5 equiv) in THF (20 mL) at 0 °C. The resulting mixture was warmed to room temperature, followed by addition of (*S*)-sulfinimine (490 mg, 2.0 mmol, 1.0 equiv) in THF (2.0 mL). The mixture was stirred continuely for 1 h and then quenched with water. After extraction with ethyl acetate, the combined organic layer was washed with brine and concentrated under vacuo. The crude product was purified through flash chromatography (eluent: petroleum ether/ethyl acetate = 5:1) to afford ($S_{S(IV)}$, S_{C-1})-**S20b** as yellow oil (510 mg, 69% yield, >99:1 d.r.).

^{1}H NMR (400 MHz, $CDCl_3$) δ 7.92 (d, *J* = 7.7 Hz, 2H), 7.54 (t, *J* = 7.4 Hz, 1H), 7.43 (t, *J* = 7.7 Hz, 2H), 4.10 (d, *J* = 8.7 Hz, 1H), 3.73 – 3.70 (m, 1H), 3.42 – 3.29 (m, 2H), 1.69 – 1.52 (m, 2H), 1.45 – 1.23 (m, 12H), 1.19 (s, 9H), 0.85 (t, *J* = 6.8 Hz, 3H).

^{13}C NMR (101 MHz, $CDCl_3$) δ 199.5, 137.1, 133.4, 128.7, 128.2, 56.0, 54.2, 44.4, 35.7, 31.9, 29.6, 29.3, 29.3, 26.3, 22.8, 22.7, 14.2.

HRMS (ESI, m/z): $[M+Na]^+$ Calcd. For $C_{21}H_{35}NO_2NaS$: 388.2286; Found: 388.2289.

HPLC (Chiralpak OD-H Column), *i*-PrOH/hexane = 3/97, flow rate = 1.0 mL/min, λ = 210 nm; t_R = 9.6 min (major), t_R = 10.7 min (minor).

[α]$_D^{20}$ = +43.1 (c = 1.1, $CHCl_3$).

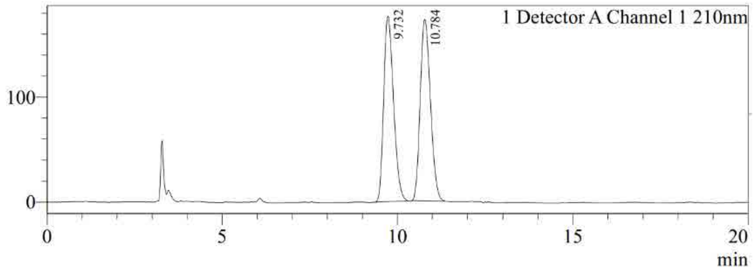

Detector A Channel 1 210nm

Peak#	Ret. Time	USP Width	Area	Height	Area%
1	9.732	0.520	3442276	176748	49.503
2	10.784	0.544	3511444	172801	50.497
Total			6953720	349549	100.000

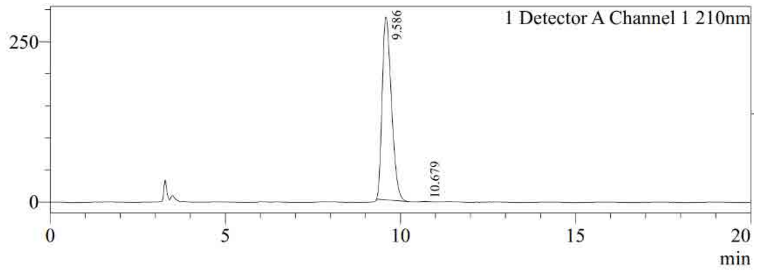

Detector A Channel 1 210nm

Peak#	Ret. Time	USP Width	Area	Height	Area%
1	9.586	0.493	5250061	285260	99.847
2	10.679	0.342	8050	613	0.153
Total			5258111	285873	100.000

_N_-((_R_)-5-Cyano-1-(1,1-dioxidobenzoisothiazol-2(_3H_)-yl)-1-oxopentan-2-yl)-2-methyl-_N_-((_S_)-1-oxo-1-phenylundecan-3-yl)propane-2-sulfonamide (20b)

Prepared according to the **GENERAL PROCEDURE C** with ($S_{S(IV)}$, $S_{C\text{-}1}$)-**S20b** (110 mg, 0.30 mmol, >99:1 d.r.) and **2k** (52.0 mg, 0.20 mmol) as substrates *(Note: Workup with m-CPBA (345 mg, 2.0 mmol, 10 equiv) instead of 6M HCl after the rearrangement reaction)*. Column chromatography: silica gel, petroleum ether/ethyl acetate = 8:1 to 4:1. White solid (92.2 mg, 70% yield, >99:1 d.r.).

^{1}H NMR (400 MHz, $CDCl_3$) δ 7.99 (d, *J* = 7.8 Hz, 2H), 7.80 (d, *J* = 7.9 Hz, 1H), 7.73 (t, *J* = 7.6 Hz, 1H), 7.61 (t, *J* = 7.3 Hz, 1H), 7.57 (d, *J* = 7.3 Hz, 1H), 7.53 – 7.42 (m, 3H), 5.22 – 5.01 (m, 2H), 4.98 – 4.78 (m, 1H), 4.69 – 4.54 (m, 1H), 3.93 – 3.65 (m, 1H), 3.45 (m, 1H), 2.46 – 2.11 (m, 5H), 2.07 – 1.78 (m, 3H), 1.39 (s, 9H), 1.29 – 1.13 (m, 11H), 1.06 – 0.93 (m, 1H), 0.83 (t, *J* = 6.8 Hz, 3H).

^{13}C NMR (101 MHz, $CDCl_3$) δ 197.9, 170.4, 136.4, 134.5, 134.1, 133.6, 130.9, 130.0, 128.8, 128.2, 125.2, 121.9, 119.4, 63.2, 62.7, 55.2, 47.8, 38.2, 31.9, 29.7, 29.6, 29.5, 29.3, 29.2, 27.3, 25.7, 23.2, 22.7, 16.6, 14.2.

HRMS (ESI, m/z): $[M+Na]^+$ Calcd. For $C_{34}H_{47}N_3O_6NaS_2$: 680.2804; Found: 680.2806.

HPLC (Chiralpak OD-H Column), *i*-PrOH/hexane = 30/70, flow rate = 1.0 mL/min, λ = 210 nm; t_R = 9.0 min (major), t_R = 12.2 min (minor).

$[\alpha]_D^{25}$ = +5.20 (c = 0.5, $CHCl_3$).

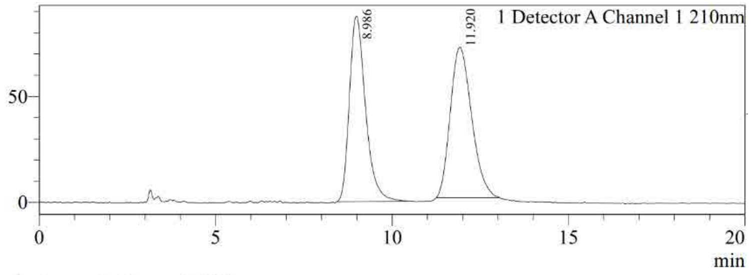

Detector A Channel 1 210nm

Peak#	Ret. Time	USP Width	Area	Height	Area%
1	8.986	0.808	2785473	87602	48.271
2	11.920	1.113	2984987	70908	51.729
Total			5770460	158510	100.000

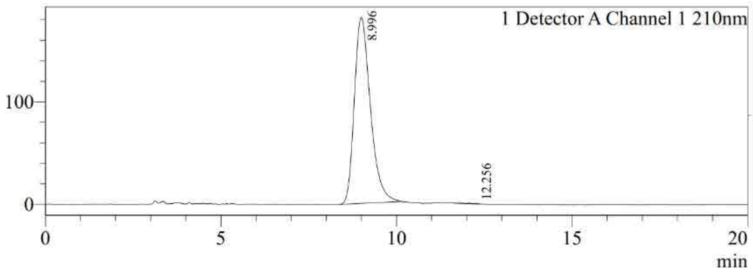

Detector A Channel 1 210nm

Peak#	Ret. Time	USP Width	Area	Height	Area%
1	8.996	0.824	5788053	181470	99.902
2	12.256	0.096	5684	420	0.098
Total			5793738	181890	100.000

Synthesis of ($S_{C\text{-}1}$, $S_{C\text{-}2}$)-21a

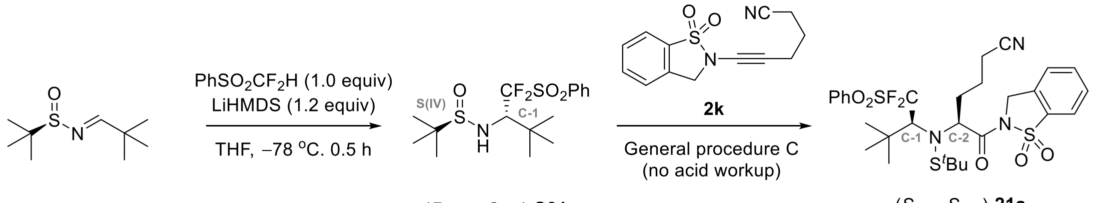

Synthesis of ($R_{S(IV)}$, $S_{C\text{-}1}$)-**S21a** (*45*)

Under N_2 atmosphere, to the solution of ((difluoromethyl)sulfonyl)benzene (768 mg, 4.0 mmol, 1.0 equiv) and (*R*)-sulfinimine (832 mg, 4.4 mmol, 1.1 equiv) in THF (20 mL) was added LiHMDS (4.8 mL, 1.0 M in THF, 1.2 equiv) dropwise at −78 °C. The reaction was stirred at the same temperature for 30 minutes, and then quenched by saturated brine. After extraction with ethyl acetate, the combined organic layer was washed with brine and concentrated under vacuo. The crude product was purified through flash chromatography (eluent: petroleum ether/ethyl acetate = 6:1) to afford ($R_{S(IV)}$, $S_{C\text{-}1}$)-**S21a** as white solid (1.45 g, 95% yield, >99:1 d.r.).

^{1}H NMR (400 MHz, $CDCl_3$) δ 7.91 (d, *J* = 7.8 Hz, 2H), 7.72 (t, *J* = 7.5 Hz, 1H), 7.59 (t, *J* = 7.7 Hz, 2H), 3.99 – 3.90 (m, 1H), 3.79 (d, *J* = 9.8 Hz, 1H), 1.31 (s, 9H), 1.17 (s, 9H).

HPLC (Chiralpak OD-H Column), *i*-PrOH/hexane = 5/95, flow rate = 1.0 mL/min, λ = 210 nm; t_R = 8.7 min (major), t_R = 10.2 min (minor).

$[\alpha]_D^{25}$ = −5.60 (c = 1.0, $CHCl_3$).

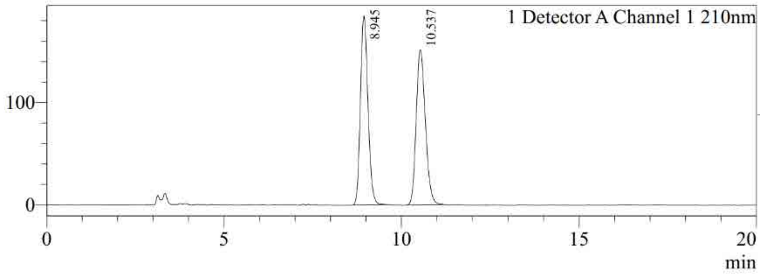

Detector A Channel 1 210nm

Peak#	Ret. Time	USP Width	Area	Height	Area%
1	8.945	0.393	2720626	184432	49.999
2	10.537	0.478	2720738	151336	50.001
Total			5441363	335768	100.000

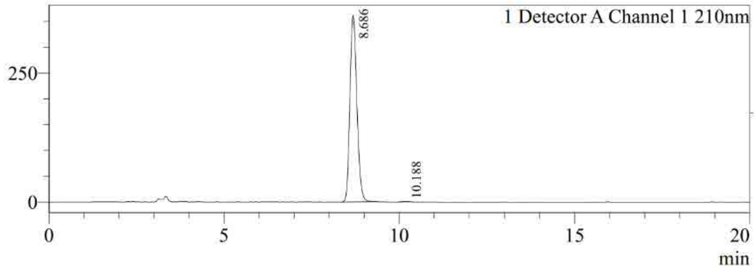

Detector A Channel 1 210nm

Peak#	Ret. Time	USP Width	Area	Height	Area%
1	8.686	0.371	5005206	360811	99.908
2	10.188	0.297	4617	461	0.092
Total			5009823	361272	100.000

(*S*)-5-((*tert*-Butylthio)((*S*)-1,1-difluoro-3,3-dimethyl-1-(phenylsulfonyl)butan-2-yl)amino)-6-(1,1-dioxidobenzoisothiazol-2(3*H*)-yl)-6-oxohexanenitrile (21a)

Prepared according to the **GENERAL PROCEDURE C** with ($R_{S(IV)}$, $S_{C\text{-}1}$)-^{t}BS-**14a** (114 mg, 0.30 mmol, >99:1 d.r.) and **2k** (52.0 mg, 0.20 mmol) as substrates (*Note, without acid workup after the rearrangement reaction*). Column chromatography: silica gel, petroleum ether/ethyl acetate/dichloromethane = 5:1:1. White solid (70.1 mg, 55% yield, 98:2 d.r.).

^{1}H NMR (400 MHz, $CDCl_3$) δ 8.03 (d, J = 7.8 Hz, 2H), 7.85 (d, J = 7.9 Hz, 1H), 7.74 (td, J = 7.6, 3.7 Hz, 2H), 7.61 (t, J = 7.8 Hz, 3H), 7.52 (d, J = 7.9 Hz, 1H), 5.05 (d, J = 16.3 Hz, 1H), 4.86 (d, J = 16.3 Hz, 1H), 4.80 (dd, J = 10.2, 4.3 Hz, 1H), 4.65 (dd, J = 27.0, 2.3 Hz, 1H), 2.59 – 2.21 (m, 4H), 1.96 – 1.80 (m, 1H), 1.67 – 1.54 (m, 1H), 1.39 (s, 9H), 1.20 (s, 9H).

^{13}C NMR (101 MHz, $CDCl_3$) δ 171.3, 135.0, 134.4, 133.6, 130.8, 130.5, 130.0, 129.2, 129.1, 125.2, 122.0, 119.8, 71.9, 71.7, 71.5 (dd, J = 35.6, 18.0 Hz), 69.6, 51.8, 47.6, 38.7, 33.9, 30.6, 30.6, 30.5, 29.4, 21.4, 17.4.

^{19}F NMR (376 MHz, $CDCl_3$) δ -93.00 (d, J = 233.0 Hz, 1F), -104.31 (dd, J = 233.0, 26.6 Hz, 1F).

HRMS (ESI, m/z): $[M+Na]^+$ Calcd. For $C_{29}H_{37}F_2N_3O_5NaS_3$: 664.1761; Found: 664.1761.

HPLC (Chiralpak AD-H Column), *i*-PrOH/hexane = 30/70, flow rate = 1.0 mL/min, λ = 210 nm; t_R = 8.7 min (minor), t_R = 10.1 min (major).

$[\alpha]_D^{25}$ = +67.6 (c = 0.5, $CHCl_3$).

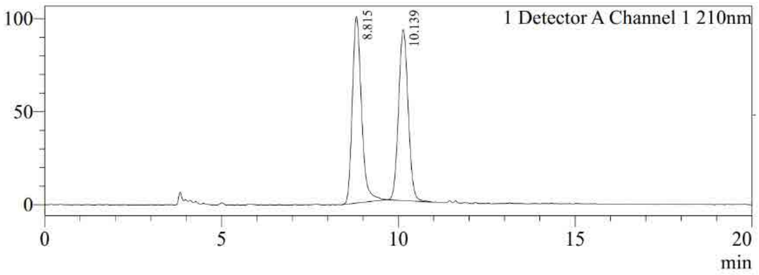

Detector A Channel 1 210nm

Peak#	Ret. Time	USP Width	Area	Height	Area%
1	8.815	0.468	1815926	100263	51.152
2	10.139	0.500	1734160	91946	48.848
Total			3550086	192209	100.000

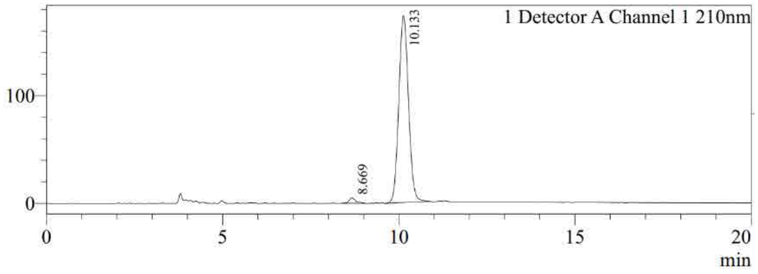

Detector A Channel 1 210nm

Peak#	Ret. Time	USP Width	Area	Height	Area%
1	8.669	0.322	64427	4982	1.901
2	10.133	0.507	3324019	173496	98.099
Total			3388447	178478	100.000

Synthesis of (R_{C-1}, R_{C-2})-21b

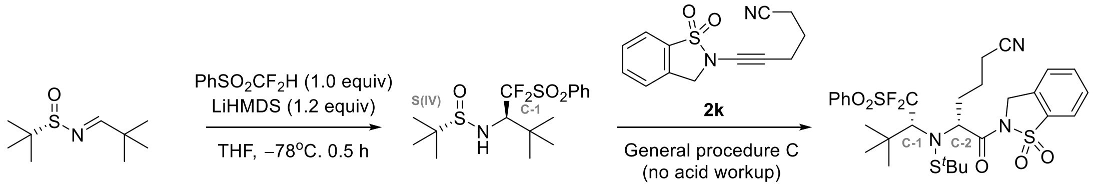

($S_{S(IV)}$, R_{C-1})-**S21b** (R_{C-1}, R_{C-2})-**21b**

Synthesis of ($S_{S(IV)}$, R_{C-1})-**S21b**

Under N_2 atmosphere, to the solution of ((difluoromethyl)sulfonyl)benzene (768 mg, 4.0 mmol, 1.0 equiv) and (*S*)-sulfinimine (832 mg, 4.4 mmol, 1.1 equiv) in THF (20 mL) was added LiHMDS (4.8 mL, 1.0 M in THF, 1.2 equiv) dropwise at −78 °C. The reaction was stirred at the same temperature for 30 minutes, and then quenched by saturated brine. After extraction with ethyl acetate, the combined organic layer was washed with brine and concentrated under vacuo. The crude product was purified through flash chromatography (eluent: petroleum ether/ethyl acetate = 6:1) to afford ($S_{S(IV)}$, R_{C-1})-**S21b** as white solid (1.20 g, 78% yield, >99:1 d.r.).

^{1}H NMR (400 MHz, $CDCl_3$) δ 7.91 (d, *J* = 7.7 Hz, 2H), 7.73 (t, *J* = 7.6 Hz, 1H), 7.59 (t, *J* = 7.7 Hz, 2H), 4.00 – 3.91 (m, 1H), 3.80 (d, *J* = 9.8 Hz, 1H), 1.32 (s, 9H), 1.18 (s, 9H).

^{13}C NMR (101 MHz, $CDCl_3$) δ 135.3, 133.9, 130.6, 129.3, 125.9, 123.00 (dd, *J* = 296.7, 291.1 Hz), 66.13 (t, *J* = 20.9 Hz), 57.8, 35.5, 28.07 (t, *J* = 2.6 Hz), 23.0.

^{19}F NMR (376 MHz, $CDCl_3$) δ -90.50 (dd, *J* = 237.1, 7.5 Hz, 1F), -103.81 (dd, *J* = 237.1, 19.1 Hz, 1F).

HRMS (ESI, m/z): $[M+Na]^+$ Calcd. For $C_{16}H_{25}F_2NO_3NaS_2$: 404.1142; Found: 404.1144.

HPLC (Chiralpak OD-H Column), *i*-PrOH/hexane = 5/95, flow rate = 1.0 mL/min, λ = 210 nm; t_R = 8.7 min (minor), t_R = 10.1 min (major).

[α]$_D^{25}$ = +5.20 (c = 1.0, $CHCl_3$).

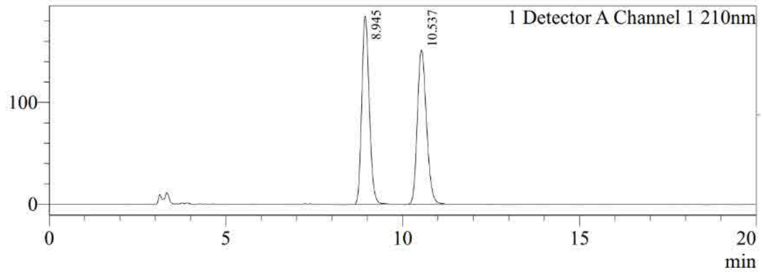

Detector A Channel 1 210nm

Peak#	Ret. Time	USP Width	Area	Height	Area%
1	8.945	0.393	2720626	184432	49.999
2	10.537	0.478	2720738	151336	50.001
Total			5441363	335768	100.000

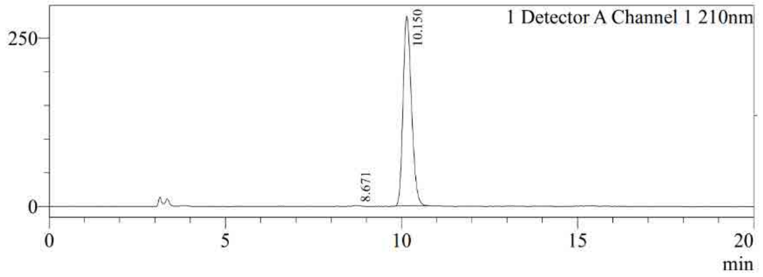

Detector A Channel 1 210nm

Peak#	Ret. Time	USP Width	Area	Height	Area%
1	8.671	0.058	185	-19	0.004
2	10.150	0.444	4705034	281965	99.996
Total			4705219	281946	100.000

(*R*)-5-((*tert*-Butylthio)((*R*)-1,1-difluoro-3,3-dimethyl-1-(phenylsulfonyl)butan-2-yl)amino)-6-(1,1-dioxidobenzoisothiazol-2(3*H*)-yl)-6-oxohexanenitrile (21b)

Prepared according to the **GENERAL PROCEDURE C** with ($S_{S(IV)}$, R_{C-1})-**S21b** (114 mg, 0.30 mmol, >99:1 d.r.) and **2k** (52.0 mg, 0.20 mmol) as substrates (*Note, without acid workup after the rearrangement reaction*). Column chromatography: silica gel, petroleum ether/ethyl acetate/dichloromethane = 5:1:1. White solid (70.6 mg, 55% yield, 98:2 d.r.).

^{1}H NMR (400 MHz, $CDCl_3$) δ 8.03 (d, *J* = 7.8 Hz, 2H), 7.85 (d, *J* = 7.9 Hz, 1H), 7.74 (td, *J* = 7.8, 3.6 Hz, 2H), 7.62 (t, *J* = 7.8 Hz, 3H), 7.52 (d, *J* = 7.8 Hz, 1H), 5.05 (d, *J* = 16.3 Hz, 1H), 4.86 (d, *J* = 16.1 Hz, 1H), 4.80 (dd, *J* = 10.1, 4.2 Hz, 1H), 4.66 (dd, *J* = 27.0, 2.5 Hz, 1H), 2.60 – 2.21 (m, 4H), 1.94 – 1.80 (m, 1H), 1.65 – 1.54 (m, 1H), 1.39 (s, 9H), 1.20 (s, 9H).

^{13}C NMR (101 MHz, $CDCl_3$) δ 171.3, 135.1, 134.4, 134.4, 133.6, 130.9, 130.6, 130.0, 129.2, 129.2, 125.2, 125.1, 122.1, 119.8, 71.67 (dd, *J* = 36.9, 18.4 Hz), 69.6, 51.8, 47.6, 38.7, 33.9, 30.7, 30.6, 30.5, 29.4, 21.4, 17.4.

^{19}F NMR (376 MHz, $CDCl_3$) δ -93.00 (d, *J* = 232.3 Hz, 1F), -104.32 (dd, *J* = 232.6, 26.9 Hz, 1F).

HRMS (ESI, m/z): $[M+Na]^+$ Calcd. For $C_{29}H_{37}F_2N_3O_5NaS_3$: 664.1761; Found: 664.1763.

HPLC (Chiralpak AD-H Column), *i*-PrOH/hexane = 30/70, flow rate = 1.0 mL/min, λ = 210 nm; t_R = 8.8 min (major), t_R = 10.3 min (minor).

$[\alpha]_D^{25}$ = –71.2 (c = 0.5, $CHCl_3$).

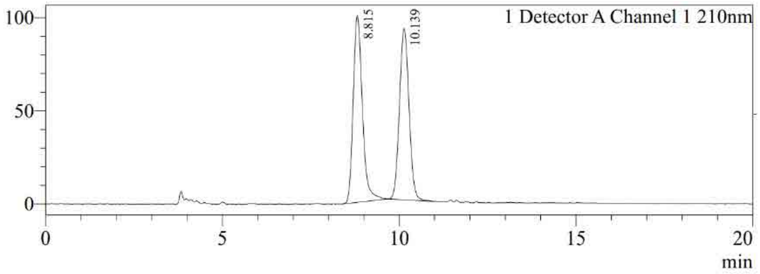

Detector A Channel 1 210nm

Peak#	Ret. Time	USP Width	Area	Height	Area%
1	8.815	0.468	1815926	100263	51.152
2	10.139	0.500	1734160	91946	48.848
Total			3550086	192209	100.000

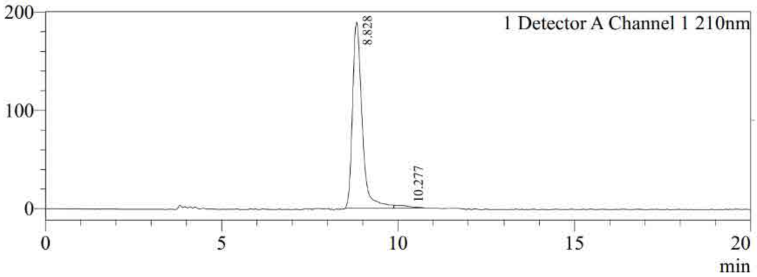

Detector A Channel 1 210nm

Peak#	Ret. Time	USP Width	Area	Height	Area%
1	8.828	0.473	3587572	188959	97.868
2	10.277	4.657	78153	1913	2.132
Total			3665725	190872	100.000

Synthesis of ($S_{C\text{-}1}$, $S_{C\text{-}2}$)-22a

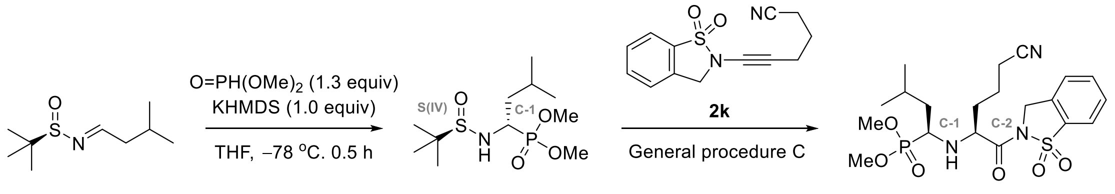

($R_{S(IV)}$, $S_{C\text{-}1}$)-**S22a** ($S_{C\text{-}1}$, $S_{C\text{-}2}$)-**22a**

Synthesis of ($R_{S(IV)}$, $S_{C\text{-}1}$)-**S22a:**

Under N_2 atmosphere, to the solution of dimethyl phosphite (768 mg, 4.0 mmol, 1.3 equiv) in THF (30 mL) was added KHMDS (7.5 mL, 0.4 M in toluene, 1.0 equiv) dropwise at –78 °C. The mixture was stirred at the same temperature for 30 minutes, and then transferred to another tube charged with (*R*)-sulfinimine (567 mg, 3.0 mmol, 1.0 equiv) in THF (30 mL) dropwise at –78 °C. The reaction was stirred at the same temperature for 20 minutes and then quenched by saturated aqueous NH_4Cl. After extraction with ethyl acetate, the combined organic layer was washed with brine and concentrated under vacuo. The crude product was purified through flash

chromatography (eluent: dichloromethane/MeOH = 40:1) to afford ($R_{S(IV)}$, S_{C-1})-**S22a** as colorless viscous oil (870 mg, 96% yield, >99:1 d.r.).

^{1}H NMR (400 MHz, $CDCl_3$) δ 3.81 (d, J = 10.6 Hz, 3H), 3.75 (d, J = 10.6 Hz, 3H), 3.67 (t, J = 8.4 Hz, 1H), 3.52 (tdd, J = 15.2, 8.4, 3.9 Hz, 1H), 1.88 – 1.75 (m, 1H), 1.69 – 1.45 (m, 2H), 1.19 (s, 9H), 0.90 (d, J = 6.7 Hz, 3H), 0.85 (d, J = 6.6 Hz, 3H).

^{13}C NMR (101 MHz, $CDCl_3$) δ 57.0, 54.00 (d, J = 7.4 Hz), 53.03 (d, J = 7.1 Hz), 50.54 (d, J = 150.7 Hz), 40.04 (d, J = 2.1 Hz), 24.02 (d, J = 11.8 Hz), 23.3, 22.8, 20.8.

^{31}P NMR (162 MHz, $CDCl_3$) δ 28.53 (s, 1P).

HRMS (ESI, m/z): $[M+Na]^+$ Calcd. For $C_{11}H_{26}NO_4NaPS$: 322.1218; Found: 322.1220.

HPLC (Chiralpak OD-H Column), *i*-PrOH/hexane = 3/97, flow rate = 1.0 mL/min, λ = 210 nm; t_R = 13.0 min (major), t_R = 14.3 min (minor).

$[\alpha]_D^{20}$ = –30.4 (c = 1.0, $CHCl_3$).

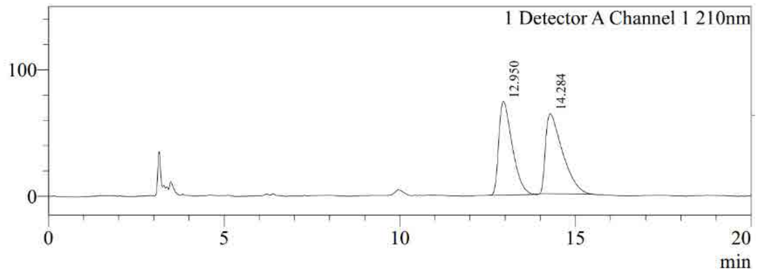

Detector A Channel 1 210nm

Peak#	Ret. Time	USP Width	Area	Height	Area%
1	12.950	0.709	1976655	73999	48.112
2	14.284	0.906	2131830	63375	51.888
Total			4108486	137374	100.000

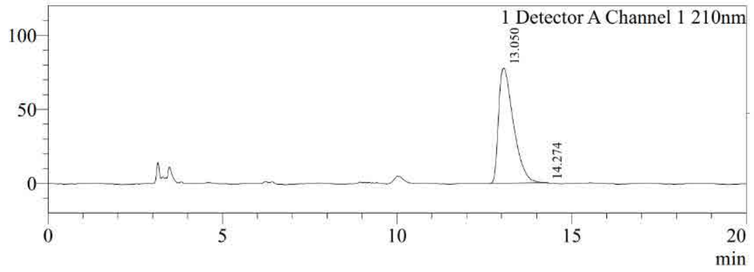

Detector A Channel 1 210nm

Peak#	Ret. Time	USP Width	Area	Height	Area%
1	13.050	0.749	2229230	77835	99.983
2	14.274	0.109	386	66	0.017
Total			2229617	77901	100.000

Dimethyl-((*S*)-1-(((*S*)-5-cyano-1-(1,1-dioxidobenzoisothiazol-2(3*H*)-yl)-1-oxopentan-2-yl)-amino)-3-methylbutyl)phosphonate (22a)

Prepared according to the **GENERAL PROCEDURE C** with ($R_{S(IV)}$, S_{C-1})-**S22a** (89.8 mg, 0.30 mmol, >99:1 d.r.) and **2k** (52.0 mg, 0.20 mmol) as substrates. Column chromatography: silica gel, dichloromethane /ethyl acetate = 2:1. White solid (49.8 mg, 53% yield, >99:1 d.r.).

^{1}H NMR (400 MHz, $CDCl_3$) δ 7.80 (d, *J* = 7.9 Hz, 1H), 7.70 (t, *J* = 7.6 Hz, 1H), 7.59 (t, *J* = 7.7 Hz, 1H), 7.48 (d, *J* = 7.8 Hz, 1H), 5.08 – 4.80 (m, 2H), 4.07 (d, *J* = 9.1 Hz, 1H), 3.71 (t, *J* = 10.7 Hz, 6H), 2.99 (dt, *J* = 13.8, 7.3 Hz, 1H), 2.49 – 2.26 (m, 4H), 1.93 (ddt, *J* = 33.3, 17.3, 10.1 Hz, 4H), 1.63 (q, *J* = 9.5 Hz, 1H), 1.55 – 1.45 (m, 2H), 0.90 (t, *J* = 5.5 Hz, 6H).

^{13}C NMR (101 MHz, $CDCl_3$) δ 173.3, 134.3, 134.2, 130.9, 129.9, 125.2, 121.9, 119.6, 77.5, 77.2, 76.8, 60.2, 53.2, 52.71 (d, *J* = 7.2 Hz), 51.7, 47.5, 39.7, 34.1, 24.36 (d, *J* = 10.0 Hz), 23.2, 22.2, 21.7, 17.0.

^{31}P NMR (162 MHz, $CDCl_3$) δ 30.05 (s, 1P).

HRMS (ESI, m/z): $[M+Na]^+$ Calcd. For $C_{20}H_{30}N_3O_6NaPS$: 494.1491; Found: 494.1495.

HPLC (Chiralpak OD-H Column), *i*-PrOH/hexane = 20/80, flow rate = 1.0 mL/min, λ = 210 nm; t_R = 17.3 min (major), t_R = 21.2 min (minor).

[α]$_D^{25}$ = −6.40 (c = 0.5, $CHCl_3$).

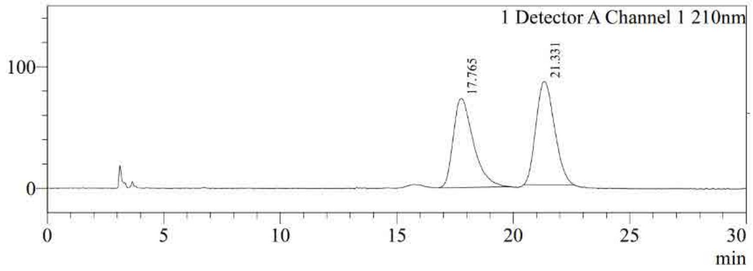

Detector A Channel 1 210nm

Peak#	Ret. Time	USP Width	Area	Height	Area%
1	17.765	1.543	4390038	73132	48.178
2	21.331	1.479	4722070	85038	51.822
Total			9112108	158170	100.000

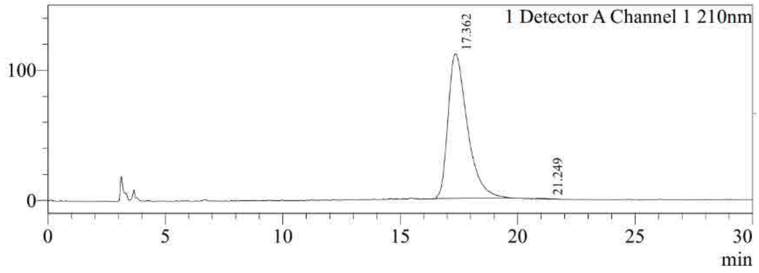

Detector A Channel 1 210nm

Peak#	Ret. Time	USP Width	Area	Height	Area%
1	17.362	1.461	6366504	111055	99.896
2	21.249	0.149	6647	470	0.104
Total			6373151	111525	100.000

Synthesis of (R_{C-1}, R_{C-2})-22b

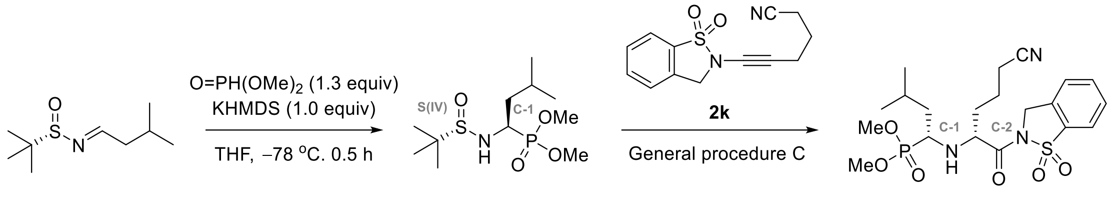

($S_{S(IV)}$, R_{C-1})-**S22b** (R_{C-1}, R_{C-2})-**22b**

Synthesis of ($S_{S(IV)}$, R_{C-1})**-S22b** (*74*)

Under N_2 atmosphere, to the solution of dimethyl phosphite (220 mg, 2.0 mmol, 1.3 equiv) in THF (20 mL) was added KHMDS (3.75 mL, 0.4 M in toluene, 1.0 equiv) dropwise at −78 °C. The mixture was stirred at the same temperature for 30 minutes, and then transferred to another tube charged with (*S*)-sulfinimine (283 mg, 1.5 mmol, 1.0 equiv) in THF (20 mL) dropwise at −78 °C. The reaction was stirred at the same temperature for 20 minutes and then quenched by saturated aqueous NH_4Cl. After extraction with ethyl acetate, the combined organic layer was washed with brine and concentrated under vacuo. The crude product was purified through flash chromatography (eluent: dichloromethane/MeOH = 40:1) to afford ($S_{S(IV)}$, R_{C-1})-**S22b** as colorless viscous oil (410 mg, 91% yield, 98:2 d.r.).

^{1}H NMR (400 MHz, $CDCl_3$) δ 3.80 (d, *J* = 10.6 Hz, 3H), 3.74 (d, *J* = 10.6 Hz, 3H), 3.66 (t, *J* = 8.3 Hz, 1H), 3.51 (dddd, *J* = 16.1, 10.6, 8.3, 4.1 Hz, 1H), 1.87 – 1.76 (m, 1H), 1.68 – 1.45 (m, 2H), 1.18 (s, 9H), 0.90 (d, *J* = 6.7 Hz, 3H), 0.84 (d, *J* = 6.6 Hz, 3H).

HPLC (Chiralpak OD-H Column), *i*-PrOH/hexane = 3/97, flow rate = 1.0 mL/min, λ = 210 nm; t_R = 13.4 min (minor), t_R = 14.5 min (major).

$[\alpha]_D^{20}$ = +26.6 (c = 1.0, $CHCl_3$).

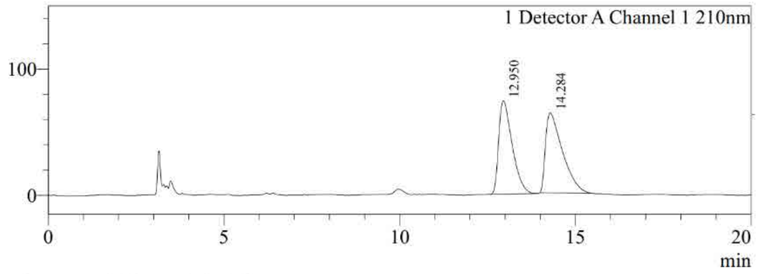

Detector A Channel 1 210nm

Peak#	Ret. Time	USP Width	Area	Height	Area%
1	12.950	0.709	1976655	73999	48.112
2	14.284	0.906	2131830	63375	51.888
Total			4108486	137374	100.000

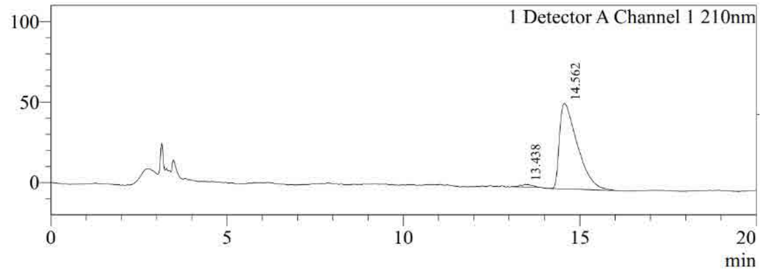

Detector A Channel 1 210nm

Peak#	Ret. Time	USP Width	Area	Height	Area%
1	13.438	0.541	34671	1581	1.810
2	14.562	0.939	1880540	52979	98.190
Total			1915211	54559	100.000

Dimethyl-((*R*)-1-(((*R*)-5-cyano-1-(1,1-dioxidobenzoisothiazol-2(3*H*)-yl)-1-oxopentan-2-yl)-amino)-3-methylbutyl)phosphonate (22b)

Prepared according to the **GENERAL PROCEDURE C** with ($S_{S(IV)}$, $R_{C\text{-}1}$)-**S22b** (89.8 mg, 0.30 mmol, 98:2 d.r.) and **2k** (52.0 mg, 0.20 mmol) as substrates. Column chromatography: silica gel, dichloromethane /ethyl acetate = 2:1. White solid (65.1 mg, 69% yield, 99:1 d.r.).

^{1}H NMR (400 MHz, $CDCl_3$) δ 7.79 (d, J = 7.9 Hz, 1H), 7.69 (t, J = 7.6 Hz, 1H), 7.57 (t, J = 7.6 Hz, 1H), 7.47 (d, J = 7.8 Hz, 1H), 5.03 – 4.78 (m, 1H), 4.04 (d, J = 9.2 Hz, 1H), 3.70 (t, J = 10.4 Hz, 6H), 2.98 (dt, J = 13.8, 7.7 Hz, 1H), 2.38 (td, J = 6.9, 2.8 Hz, 2H), 2.34 – 2.24 (m, 1H), 1.91 (ddt, J = 34.9, 16.8, 9.2 Hz, 4H), 1.61 (q, J = 9.2 Hz, 1H), 1.49 (dt, J = 9.5, 6.4 Hz, 2H), 0.91 – 0.86 (m, 6H).

^{13}C NMR (101 MHz, $CDCl_3$) δ 173.4, 134.3, 130.9, 129.8, 125.2, 121.8, 119.5, 60.2, 53.09 (d, J = 8.6 Hz), 52.65 (d, J = 7.3 Hz), 51.7, 47.4, 39.8, 34.1, 24.36 (d, J = 10.1 Hz), 23.1, 22.1, 21.7, 16.9.

^{31}P NMR (162 MHz, $CDCl_3$) δ 29.98 (s, 1P).

HRMS (ESI, m/z): $[M+Na]^+$ Calcd. For $C_{20}H_{30}N_3O_6NaPS$: 494.1491; Found: 494.1494.

HPLC (Chiralpak OD-H Column), *i*-PrOH/hexane = 20/80, flow rate = 1.0 mL/min, λ = 210 nm; t_R = 17.6 min (minor), t_R = 21.0 min (major).

[α]$_D^{25}$ = +5.60 (c = 0.5, $CHCl_3$).

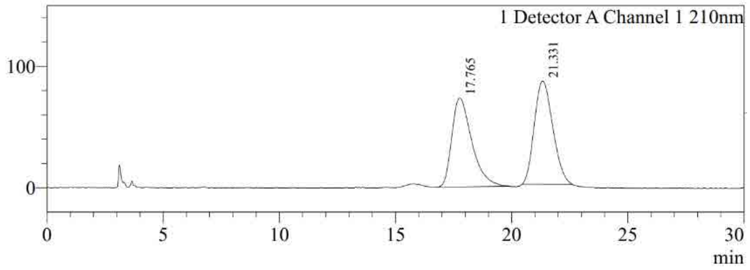

Detector A Channel 1 210nm

Peak#	Ret. Time	USP Width	Area	Height	Area%
1	17.765	1.543	4390038	73132	48.178
2	21.331	1.479	4722070	85038	51.822
Total			9112108	158170	100.000

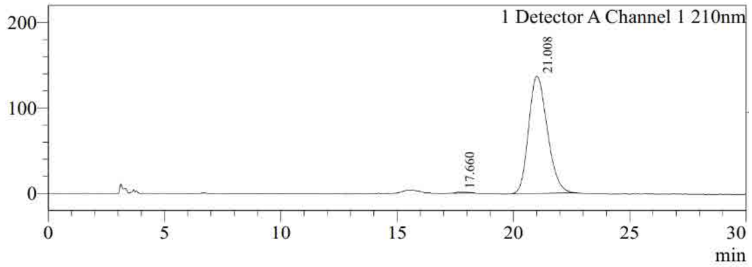

Detector A Channel 1 210nm

Peak#	Ret. Time	USP Width	Area	Height	Area%
1	17.660	0.587	40900	1609	0.528
2	21.008	1.480	7699705	137031	99.472
Total			7740605	138640	100.000

Process optimization for the synthesis of (*R,R*)-22b

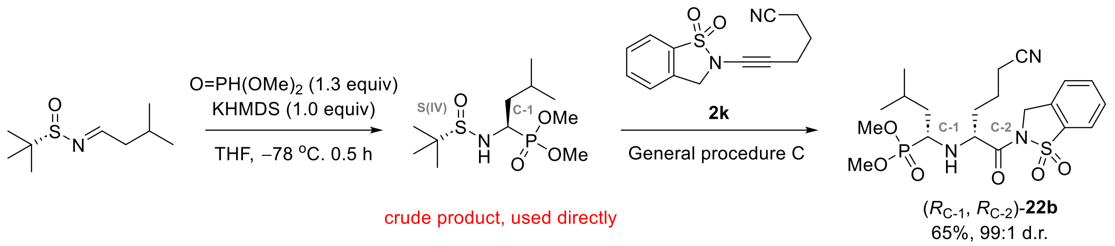

To the solution of dimethyl phosphite (57.2 mg, 0.52 mmol, 1.3 equiv) in THF (5.0 mL) was added KHMDS (0.4 mL, 1.0 M in toluene, 1.0 equiv) dropwise at −78 °C under N_2 atmosphere. The mixture was stirred at the same temperature for 30 minutes, and then transferred to another tube charged with (*S*)-sulfinimine (75.7 mg, 0.40 mmol, 1.0 equiv) in THF (5.0 mL) dropwise at −78 °C. The reaction was stirred at the same temperature for 30 minutes and then quenched by saturated aqueous NH_4Cl. After extraction with ethyl acetate, the combined organic layer was washed with brine, dried with Na_2SO_4 and concentrated under vacuo. The crude product was used directly for the next step.

Under N_2 atmosphere, the solution of *tert*-butanesulfinamide prepared above and ynamide **2k** (0.20 mmol, 52.0 mg) in cyclopentyl methyl ether (CPME, 1.5 mL, 0.1 M) was cooled to −10 °C, followed by the addition of $HNTf_2$ (0.4 mL, 0.1 M stock solution in CPME, 0.20 equiv). The resulted mixture was stirred at the same temperature for 4 h and then workup with 6M HCl (0.20 mL, 6.0 equiv) at room temperature for 3 hours. Thereafter, the reaction mixture was quenched by saturated aqueous $NaHCO_3$ and extracted with ethyl acetate (EA, 2 × 20.0 mL). The combined organic layer was washed with brine and concentrated under reduced pressure. The crude mixture was purified by flash chromatography on silica gel to afford product as colorless syrup (61.8 mg, 65% yield, 99:1 d.r.).

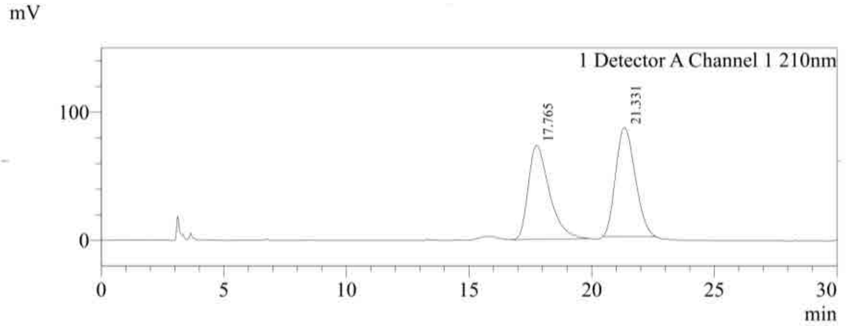

Detector A Channel 1 210nm

Peak#	Ret. Time	USP Width	Area	Height	Area%
1	17.765	1.543	4390038	73132	48.178
2	21.331	1.479	4722070	85038	51.822
Total			9112108	158170	100.000

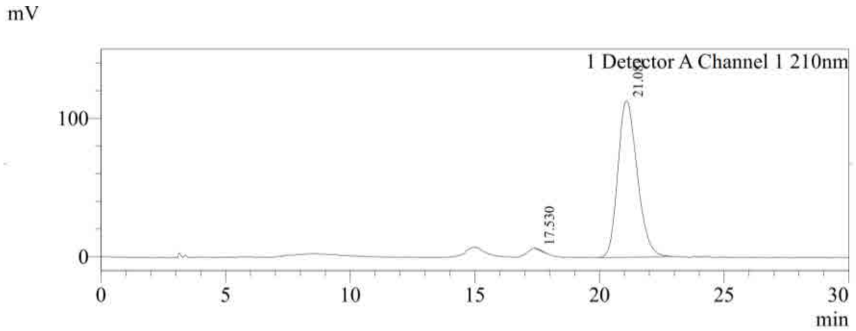

Detector A Channel 1 210nm

Peak#	Ret. Time	USP Width	Area	Height	Area%
1	17.530	0.371	9336	473	0.152
2	21.082	1.420	6142997	113104	99.848
Total			6152332	113577	100.000

Synthesis of ($\boldsymbol{R}_{\mathbf{C\text{-}1}}$, $\boldsymbol{S}_{\mathbf{C\text{-}2}}$, $\boldsymbol{S}_{\mathbf{C\text{-}3}}$)-23a

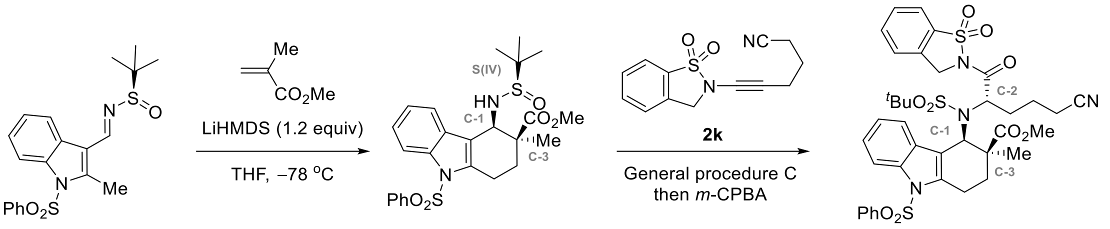

($R_{C\text{-}1}$, $R_{S(IV)}$, $S_{C\text{-}3}$)-**S23a** ($R_{C\text{-}1}$, $S_{C\text{-}2}$, $S_{C\text{-}3}$)-**23a**

Synthesis of ($R_{C\text{-}1}$, $R_{S(IV)}$, $S_{C\text{-}3}$)-**S23** (*49*)

Under N_2 atmosphere, LiHMDS (2.4 mL, 1.0 M in THF, 1.2 equiv) was added dropwise to the solution of (*R*)-sulfinimine (805 mg, 2.0 mmol, 1.0 equiv) in THF (40 mL) at −78 °C. The resulting mixture was stirred at this temperature for 1 h, followed by addition of methyl methacrylate in THF (10 mL). The mixture was stirred continuely at −78 °C for 2 h, and then quenched by saturated aqueous NH_4Cl. After extraction with ethyl acetate, the combined organic layer was washed with brine and concentrated under vacuo. The crude product was purified through flash chromatography (eluent: petroleum ether/ethyl acetate = 3:1 to 1:1) to afford ($R_{C\text{-}1}$, $R_{S(IV)}$, $S_{C\text{-}3}$)-**S23** as colorless viscous oil (610 mg, 60% yield, >99:1 d.r.).

^{1}H NMR (400 MHz, $CDCl_3$) δ 8.18 – 8.13 (m, 1H), 7.73 – 7.68 (m, 2H), 7.66 – 7.62 (m, 1H), 7.56 – 7.47 (m, 1H), 7.42 – 7.37 (m, 2H), 7.34 – 7.27 (m, 2H), 4.71 (d, *J* = 6.1 Hz, 1H), 4.08 (d, *J* = 6.1 Hz, 1H), 3.78 (s, 3H), 3.25 (ddd, *J* = 19.0, 6.4, 2.0 Hz, 1H), 2.86 (ddd, *J* = 18.2, 11.2, 6.8 Hz, 1H), 2.23 (ddd, *J* = 14.2, 11.2, 6.4 Hz, 1H), 2.06 (ddt, *J* = 14.1, 6.6, 1.8 Hz, 1H), 1.07 (s, 3H), 1.02 (s, 8H).

HPLC (Chiralpak OD-H Column), *i*-PrOH/hexane = 5/95, flow rate = 1.0 mL/min, λ = 210 nm; t_R = 19.1 min (major), t_R = 27.8 min (minor).

$[\alpha]_D^{20}$ = −8.40 (c = 1.5, $CHCl_3$).

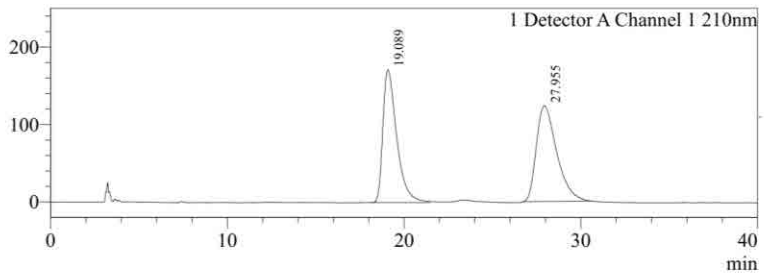

Detector A Channel 1 210nm

Peak#	Ret. Time	USP Width	Area	Height	Area%
1	19.089	1.380	9309193	171083	48.420
2	27.955	2.097	9916906	123359	51.580
Total			19226099	294441	100.000

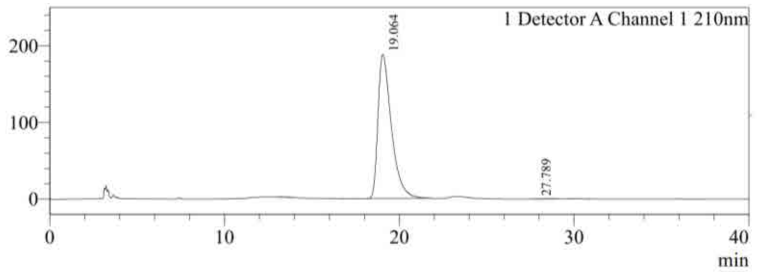

Detector A Channel 1 210nm

Peak#	Ret. Time	USP Width	Area	Height	Area%
1	19.064	1.360	10022923	187747	99.821
2	27.789	0.091	18006	213	0.179
Total			10040929	187960	100.000

Methyl (3*S*,4*R*)-4-((*N*-((*S*)-5-cyano-1-(1,1-dioxidobenzoisothiazol-2(3*H*)-yl)-1-oxopentan-2-yl)-2-methylpropan-2-yl)sulfonamido)-3-methyl-9-(phenylsulfonyl)-2,3,4,9-tetrahydro-1*H*-carbazole-3-carboxylate (23)

Prepared according to the **GENERAL PROCEDURE C** with ($R_{C\text{-}1}$, $R_{S(IV)}$, $S_{C\text{-}3}$)-**S23a** (151 mg, 0.30 mmol, >99:1 d.r.) and **2k** (52.0 mg, 0.20 mmol) as substrates (*Note, workup with m-CPBA (345 mg, 2.0 mmol, 10 equiv) instead of 6M HCl after the rearrangement reaction*). Column chromatography: silica gel, petroleum ether/ethyl acetate = 8:1 to 4:1. White solid (104.4 mg, 66% yield, >99:1 d.r.).

^{1}H NMR (400 MHz, $CDCl_3$) δ 7.85 (d, *J* = 7.9 Hz, 1H), 7.73 (t, *J* = 7.6 Hz, 1H), 7.70 – 7.61 (m, 2H), 7.61 – 7.41 (m, 4H), 7.33 (t, *J* = 7.8 Hz, 2H), 7.28 – 7.18 (m, 3H), 5.31 – 5.20 (m, 2H), 4.14 – 4.00 (m, 1H), 3.83 – 3.70 (m, 4H), 2.90 – 2.62 (m, 3H), 2.54 – 2.34 (m, 4H), 2.18 – 2.05 (m, 2H), 1.78 (dd, *J* = 15.1, 7.9 Hz, 1H), 1.69 (s, 9H), 0.82 (s, 3H).

^{13}C NMR (101 MHz, $CDCl_3$) δ175.3, 169.9, 141.1, 138.9, 135.2, 134.1, 131.4, 129.7, 129.7, 129.5, 126.2, 124.8, 124.2, 123.3, 122.0, 120.1, 119.7, 113.3, 113.1, 112.4, 67.8, 62.3, 62.2, 52.5, 48.3, 46.5, 29.6, 26.6, 25.9, 23.9, 21.5, 20.8, 17.3.

HRMS (ESI, m/z): $[M+Na]^+$ Calcd. For $C_{38}H_{42}N_4O_9NaS_3$: 817.2012; Found: 817.2015.

HPLC (Chiralpak AD-H Column), *i*-PrOH/hexane = 30/70, flow rate = 1.0 mL/min, λ = 254 nm; t_R = 16.3 min (major), t_R = 28.1 min (minor).

$[\alpha]_D^{25}$ = +110.8 (c = 0.5, $CHCl_3$).

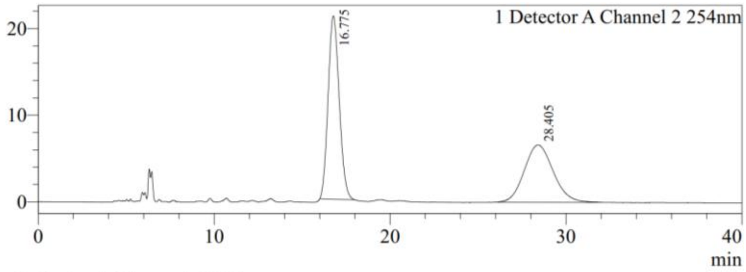

Detector A Channel 2 254nm

Peak#	Ret. Time	USP Width	Area	Height	Area%
1	16.775	1.171	931598	21165	55.120
2	28.405	3.008	758516	6620	44.880
Total			1690114	27785	100.000

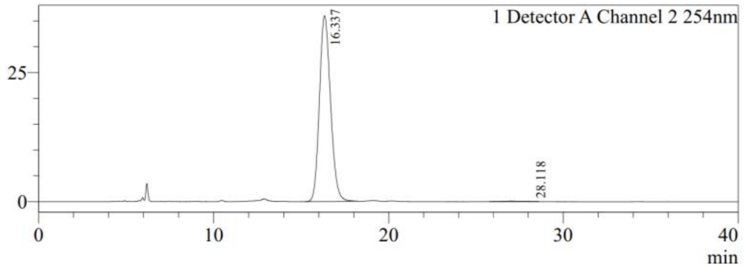

Detector A Channel 2 254nm

Peak#	Ret. Time	USP Width	Area	Height	Area%
1	16.337	1.160	1590948	36015	99.457
2	28.118	2.272	8688	49	0.543
Total			1599636	36065	100.000

Synthesis of ($R_{C\text{-}1}$, $S_{C\text{-}2}$)-24a

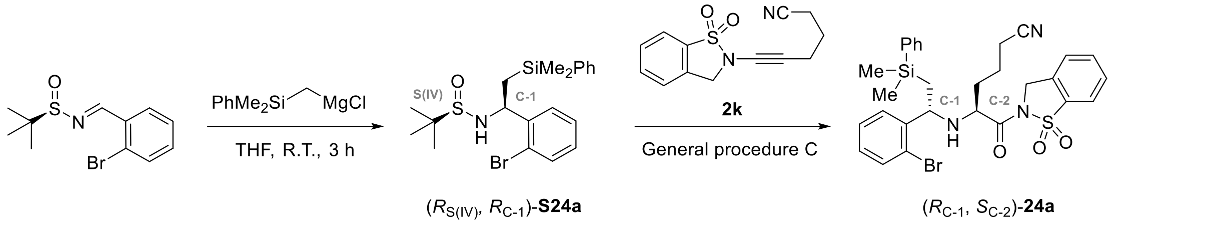

Synthesis of ($R_{S(IV)}$, $R_{C\text{-}1}$)**-S24a** (*75*)

Step 1: Procedure for the synthesis of Grignard reagent was according to the known literature (*76*): Under N_2 atmosphere, the mxiture of magnesium shavings (233 mg, 9.6 mmol), a grain of iodine in distilled THF (4.0 mL) was heated to weak reflux (60–68 °C), follwed by addition of 1,2-dibromoethane (10 uL) and chlorosilane (1.48 g, 8.0 mmol), dropwise. Meantime, the reaction solution changed from reddish brown to colorless. Once the addition was completed, the mixture was refluxed for 2 h. And the reasulting crude Grignard reagent was used directly for the next step.

Step 2: Under N_2 atmosphere, to the solution of (*R*)-sulfinimine (288 mg, 1.0 mmol) in THF (5.0 mL) was added the [(dimethylphenylsilyl)methyl]magnesium chloride prepared above dropwise at room temperature. The mixture was stirred for 3 h, and then quenched with saturated aqueous NH_4Cl, extracted with ethyl acetate. The combined organic layer was washed with brine and concentrated under vacuo. The crude product was purified through flash chromatography (eluent: petroleum ether/ethyl acetate/ dichloromethane = 100:1:1) to afford ($R_{S(IV)}$, $R_{C\text{-}1}$)-**S24a** as colorless oil (350 mg, 80% yield, >99:1 d.r.).

^{1}H NMR (400 MHz, $CDCl_3$) δ 7.52 – 7.43 (m, 3H), 7.38 – 7.29 (m, 4H), 7.21 (t, *J* = 7.6 Hz, 1H), 7.05 (t, *J* = 7.5 Hz, 1H), 5.08 (t, *J* = 8.0 Hz, 1H), 3.42 (s, 1H), 1.55 (dd, *J* = 15.0, 6.8 Hz, 1H), 1.45 (dd, *J* = 14.5, 7.5 Hz, 1H), 1.00 (s, 9H), 0.37 (s, 3H), 0.13 (s, 3H).

HPLC (Chiralpak OD-H Column), *i*-PrOH/hexane = 3/97, flow rate = 1.0 mL/min, λ = 210 nm; t_R = 8.2 min (minor), t_R = 8.9 min (major).

$[\alpha]_D^{20}$ = −77.7 (c = 0.5, $CHCl_3$).

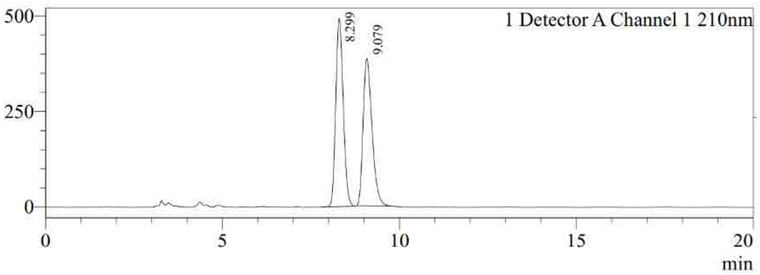

Detector A Channel 1 210nm

Peak#	Ret. Time	USP Width	Area	Height	Area%
1	8.299	0.394	7234924	491931	51.611
2	9.079	0.463	6783360	385553	48.389
Total			14018283	877483	100.000

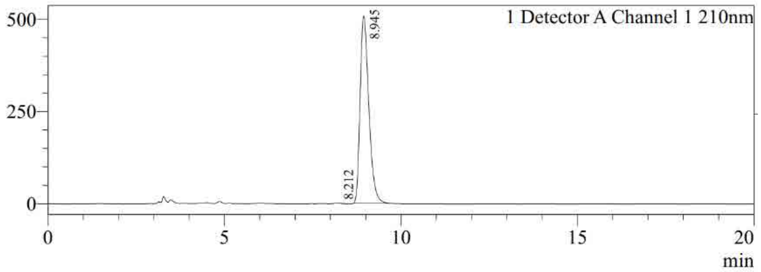

Detector A Channel 1 210nm

Peak#	Ret. Time	USP Width	Area	Height	Area%
1	8.212	0.191	2479	233	0.028
2	8.945	0.451	8700339	507400	99.972
Total			8702818	507633	100.000

(*S*)-5-(((*R*)-1-(2-Bromophenyl)-2-(dimethyl(phenyl)silyl)ethyl)amino)-6-(1,1-dioxidobenzo-isothiazol-2(3*H*)-yl)-6-oxohexanenitrile (24a)

Prepared according to the **GENERAL PROCEDURE C** with ($R_{S(IV)}$, $R_{C\text{-}1}$)-**S24a** (131 mg, 0.30 mmol, >99:1 d.r.) and **2k** (52.0 mg, 0.20 mmol) as substrates. Column chromatography: silica gel, petroleum ether/ethyl acetate/dichloromethane = 8:1:1. White solid (80.3 mg, 66% yield, 99:1 d.r.).

^{1}H NMR (400 MHz, $CDCl_3$) δ 7.68 (t, J = 8.0 Hz, 2H), 7.63 – 7.51 (m, 4H), 7.45 (dd, J = 7.9, 3.3 Hz, 2H), 7.39 – 7.33 (m, 3H), 7.30 (t, J = 7.5 Hz, 1H), 7.08 (t, J = 7.7 Hz, 1H), 4.93 (d, J = 15.6 Hz, 1H), 4.88 (d, J = 16.0 Hz, 1H), 4.35 (t, J = 6.9 Hz, 1H), 3.65 (s, 1H), 2.21 (t, J = 7.2 Hz, 2H), 1.92 – 1.77 (m, 2H), 1.72 (h, J = 6.5, 4.9 Hz, 1H), 1.41 (dd, J = 15.3, 6.4 Hz, 1H), 1.34 – 1.20 (m, 2H), 0.45 (s, 3H), 0.24 (s, 3H).

^{13}C NMR (101 MHz, $CDCl_3$) δ 174.0, 143.7, 139.0, 134.2, 134.1, 133.8, 132.8, 130.7, 129.7, 129.1, 128.5, 127.82, 127.77, 125.1, 124.2, 121.9, 119.6, 58.1, 56.1, 47.3, 32.7, 22.0, 16.6, -2.3, -3.1.

HRMS (ESI, m/z): $[M+H]^+$ Calcd. For $C_{29}H_{33}BrN_3O_3SSi$: 610.1195; Found: 610.1198.

HPLC (Chiralpak AD-H Column), *i*-PrOH/hexane = 5/95, flow rate = 1.0 mL/min, λ = 210 nm; t_R = 37.7 min (major), t_R = 39.2 min (minor).

$[\alpha]_D^{25}$ = –46.8 (c = 0.5, $CHCl_3$).

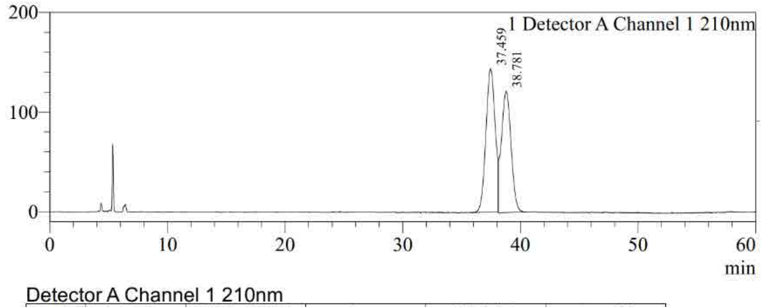

Detector A Channel 1 210nm

Peak#	Ret. Time	USP Width	Area	Height	Area%
1	37.459	1.521	7859314	143969	52.748
2	38.781	1.623	7040462	121850	47.252
Total			14899776	265819	100.000

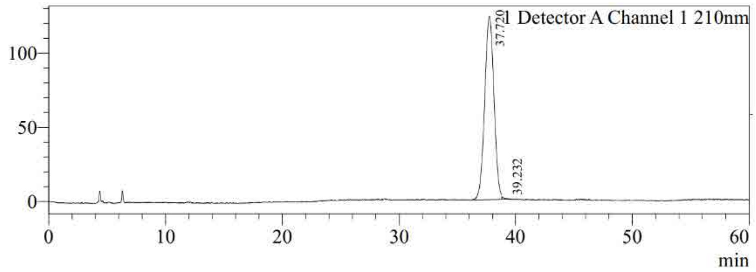

Detector A Channel 1 210nm

Peak#	Ret. Time	USP Width	Area	Height	Area%
1	37.720	1.463	6840324	123537	99.566
2	39.232	12.499	29787	592	0.434
Total			6870111	124129	100.000

Synthesis of (*S*$_{C-1}$, *R*$_{C-2}$)-24b

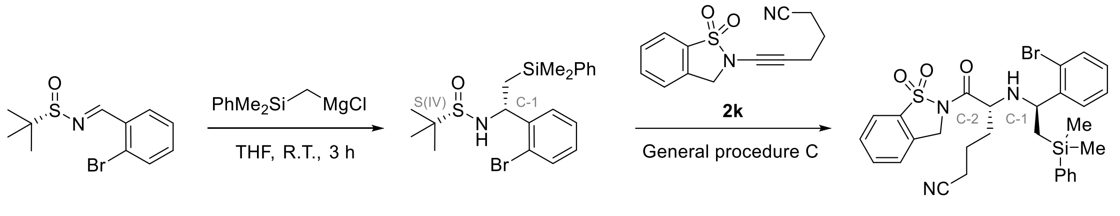

(*S*$_{S(IV)}$, *S*$_{C-1}$)-**S24b** (*R*$_{C-2}$, *S*$_{C-1}$)-**24b**

Synthesis of (*S*$_{S(IV)}$, *S*$_{C-1}$)-**S24b**

Step 1: Procedure for the synthesis of Grignard reagent is the same as above (**S24a)**.

Step 2: Under N_2 atmosphere, to the solution of (*S*)-sulfinimine (288 mg, 1.0 mmol) in THF (5.0 mL) was added the [(dimethylphenylsilyl)methyl]magnesium chloride prepared above dropwise at room temperature. The mixture was stirred for 3 h, and then quenched with saturated aqueous NH_4Cl, extracted with ethyl acetate. The combined organic layer was washed with brine and concentrated under vacuo. The crude product was purified through flash chromatography (eluent: petroleum ether/ethyl acetate/ dichloromethane = 100:1:1) to afford (*S*$_{S(IV)}$, *S*$_{C-1}$)-**S24b** as colorless oil (194 mg, 44% yield, 97:3 d.r.).

^{1}H NMR (400 MHz, $CDCl_3$) δ 7.53 – 7.46 (m, 3H), 7.39 – 7.28 (m, 4H), 7.22 (t, *J* = 7.5 Hz, 1H), 7.06 (td, *J* = 7.6, 1.8 Hz, 1H), 5.07 (t, *J* = 7.3 Hz, 1H), 3.39 (s, 1H), 1.56 (dd, *J* = 14.7, 6.9 Hz, 1H), 1.45 (dd, *J* = 14.7, 7.7 Hz, 1H), 1.01 (s, 9H), 0.37 (s, 3H), 0.13 (s, 3H).

^{13}C NMR (101 MHz, $CDCl_3$) δ 143.0, 138.2, 133.9, 133.1, 129.4, 128.8, 128.2, 127.5, 123.3, 55.5, 26.5, 22.5, -2.2, -3.3.

HRMS (ESI, m/z): $[M+Na]^+$ Calcd. For $C_{20}H_{28}BrNONaSSi$: 460.0742; Found: 460.0745.

HPLC (Chiralpak OD-H Column), *i*-PrOH/hexane = 3/97, flow rate = 1.0 mL/min, λ = 210 nm; t_R = 8.2 min (major), t_R = 9.0 min (minor).

$[\alpha]_D^{20}$ = +78.0 (c = 0.5, $CHCl_3$).

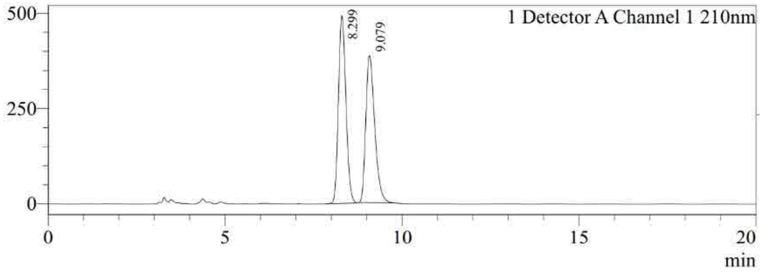

Detector A Channel 1 210nm

Peak#	Ret. Time	USP Width	Area	Height	Area%
1	8.299	0.394	7234924	491931	51.611
2	9.079	0.463	6783360	385553	48.389
Total			14018283	877483	100.000

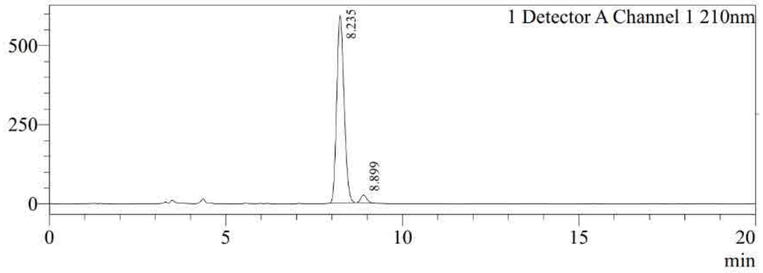

Detector A Channel 1 210nm

Peak#	Ret. Time	USP Width	Area	Height	Area%
1	8.235	0.390	8586591	592352	96.724
2	8.899	0.321	290800	24701	3.276
Total			8877391	617052	100.000

(*R*)-5-(((*S*)-1-(2-Bromophenyl)-2-(dimethyl(phenyl)silyl)ethyl)amino)-6-(1,1-dioxidobenzo-isothiazol-2(3*H*)-yl)-6-oxohexanenitrile (24b)

Prepared according to the **GENERAL PROCEDURE C** with ($S_{S(IV)}$, $S_{C\text{-}1}$)-**S24b** (131 mg, 0.30 mmol, 97:3 d.r.) and **2k** (52.0 mg, 0.20 mmol) as substrates. Column chromatography: silica gel, petroleum ether/ethyl acetate/dichloromethane = 8:1:1. White solid (95.2 mg, 78% yield, >99:1 d.r.).

^{1}H NMR (400 MHz, $CDCl_3$) δ 7.69 (t, *J* = 7.9 Hz, 2H), 7.63 – 7.51 (m, 4H), 7.45 (t, *J* = 6.7 Hz, 2H), 7.38 – 7.33 (m, 3H), 7.30 (t, *J* = 7.5 Hz, 1H), 7.07 (t, *J* = 7.7 Hz, 1H), 4.93 (d, *J* = 16.0 Hz, 1H), 4.88 (d, *J* = 15.6 Hz, 1H), 4.34 (t, *J* = 7.0 Hz, 1H), 3.63 (d, *J* = 9.1 Hz, 1H), 2.21 (t, *J* = 7.1 Hz, 2H), 2.15 – 1.94 (m, 1H), 1.84 (dq, *J* = 15.4, 8.9, 6.7 Hz, 2H), 1.71 (dt, *J* = 13.9, 8.6 Hz, 1H), 1.38 (q, *J* = 8.3, 7.3 Hz, 1H), 1.26 (d, *J* = 8.2 Hz, 2H), 0.45 (s, 3H), 0.23 (s, 3H).

^{13}C NMR (101 MHz, $CDCl_3$) δ 174.1, 139.1, 134.2, 133.9, 132.9, 130.7, 129.8, 129.1, 128.6, 127.9, 127.8, 125.1, 124.2, 121.9, 119.7, 58.1, 55.8, 47.3, 32.8, 25.8, 22.1, 16.7, -2.2, -3.0.

HRMS (ESI, m/z): $[M+H]^+$ Calcd. For $C_{29}H_{33}BrN_3O_3SSi$: 610.1195; Found: 610.1197.

HPLC (Chiralpak AD-H Column), *i*-PrOH/hexane = 5/95, flow rate = 1.0 mL/min, λ = 210 nm; t_R = 37.4 min (minor), t_R = 39.0 min (major).

$[\alpha]_D^{25}$ = +44.0 (c = 0.5, $CHCl_3$).

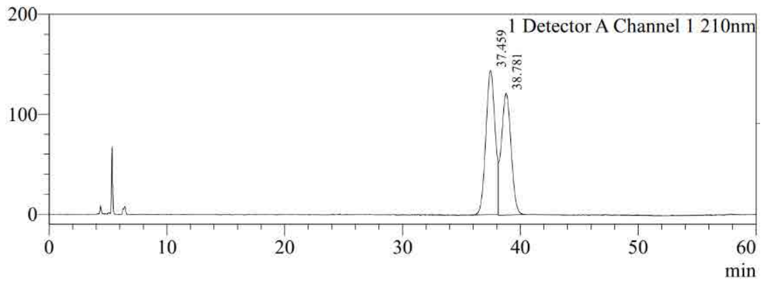

Detector A Channel 1 210nm

Peak#	Ret. Time	USP Width	Area	Height	Area%
1	37.459	1.521	7859314	143969	52.748
2	38.781	1.623	7040462	121850	47.252
Total			14899776	265819	100.000

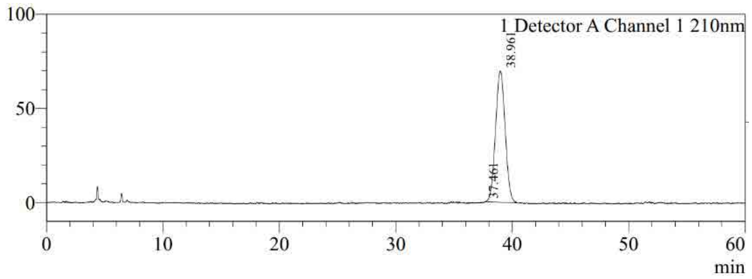

Detector A Channel 1 210nm

Peak#	Ret. Time	USP Width	Area	Height	Area%
1	37.461	0.102	1736	254	0.043
2	38.961	1.526	4013747	69667	99.957
Total			4015483	69921	100.000

Synthesis of (S_{C-1}, S_{C-2})-25a

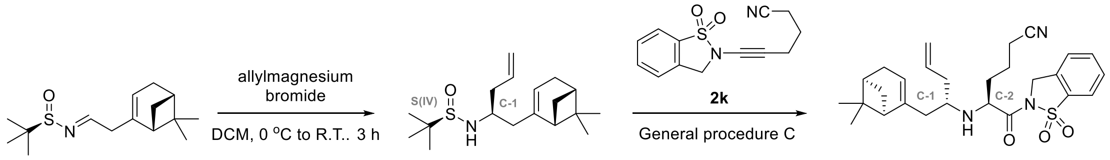

($R_{S(IV)}$, S_{C-1})-**S25a** (S_{C-2}, S_{C-1})-**25a**

Synthesis of ($R_{S(IV)}$, S_{C-1})-**S25a**

Under N_2 atmosphere, to the solution of (*R*)-sulfinimine (2.67 g, 10.0 mmol, 1.0 equiv) in dichoromethane (0.13 M) was added allylmagnesium bromide (15 mL, 1.0 M in Et_2O, 1.5 equiv) dropwise at 0 °C. The reaction was warmed to room temperature and stirred for 1 h, and then quenched by saturated aqueous NH_4Cl. After extraction with dichloromethane, the combined organic layer was washed with brine and concentrated under vacuo. The crude product was purified through flash chromatography (eluent: petroleum ether/ethyl acetate = 4:1 to 2:1) to afford ($R_{S(IV)}$, S_{C-1})-**S25a** as colorless oil (1.41 g, 45% yield, 99:1 d.r.).

^{1}H NMR (400 MHz, $CDCl_3$) δ 5.81 – 5.71 (m, 1H), 5.25 (s, 1H), 5.14 – 5.10 (m, 2H), 3.35 – 3.25 (m, 2H), 2.49 – 2.43 (m, 1H), 2.37 – 2.32 (m, 1H), 2.28 – 2.19 (m, 4H), 2.06 – 2.04 (m, 3H), 1.25 (s, 3H), 1.17 (s, 9H), 1.12 (d, *J* = 8.7 Hz, 1H), 0.81 (s, 3H).

^{13}C NMR (101 MHz, $CDCl_3$) δ144.6, 134.5, 120.0, 118.9, 55.8, 52.6, 46.0, 43.2, 40.7, 40.0, 38.0, 31.8, 31.5, 26.3, 22.8, 21.4.

HRMS (ESI, m/z): $[M+Na]^+$ Calcd. For $C_{18}H_{31}NONaS$: 332.2024; Found: 332.2027.

HPLC (Chiralpak OD-H Column), *i*-PrOH/hexane = 1/99, flow rate = 1.0 mL/min, λ = 210 nm; t_R = 8.0 min (minor), t_R = 9.8 min (major).

$[\alpha]_D^{20}$ = –71.6 (c = 1.0, $CHCl_3$).

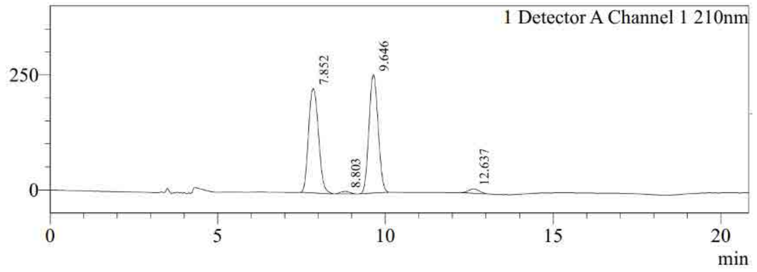

Detector A Channel 1 210nm

Peak#	Ret. Time	USP Width	Area	Height	Area%
1	7.852	0.543	4702229	226893	47.411
2	8.803	0.535	104799	5437	1.057
3	9.646	0.510	4887218	257391	49.276
4	12.637	0.638	223815	9662	2.257
Total			9918060	499383	100.000

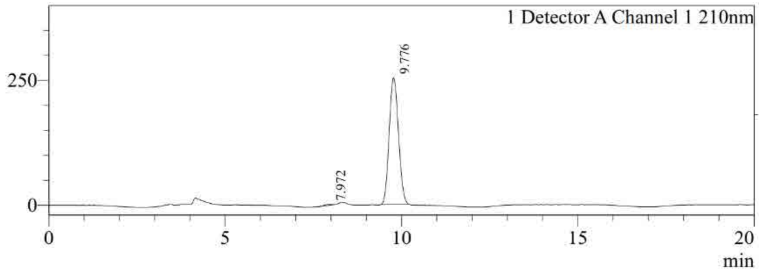

Detector A Channel 1 210nm

Peak#	Ret. Time	USP Width	Area	Height	Area%
1	7.972	0.358	27673	1633	0.590
2	9.776	0.494	4662166	254053	99.410
Total			4689839	255686	100.000

(*S*)-5-(((*S*)-1-((1*R*,5*S*)-6,6-Dimethylbicyclo[3.1.1]hept-2-en-2-yl)pent-4-en-2-yl)amino)-6-(1,1-dioxidobenzoisothiazol-2(3*H*)-yl)-6-oxohexanenitrile (25a)

Prepared according to the **GENERAL PROCEDURE C** with ($R_{S(IV)}$, S_{C-1})-**S25a** (92.8 mg, 0.30 mmol, 99:1 d.r.) and **2k** (52.0 mg, 0.20 mmol) as substrates. Column chromatography: silica gel, petroleum ether/ethyl acetate/dichloromethane = 5:1:1. White solid (67.4 mg, 70% yield, 98:2 d.r.).

^{1}H NMR (400 MHz, $CDCl_3$) δ 7.82 (d, *J* = 7.9 Hz, 1H), 7.72 (t, *J* = 7.6 Hz, 1H), 7.61 (t, *J* = 7.7 Hz, 1H), 7.49 (d, *J* = 7.8 Hz, 1H), 5.73 (dtd, *J* = 17.7, 8.7, 5.8 Hz, 1H), 5.27 (s, 1H), 5.11 (d, *J* = 12.6 Hz, 2H), 5.01 (d, *J* = 15.9 Hz, 1H), 4.90 (d, *J* = 15.9 Hz, 1H), 4.12 (d, *J* = 8.9 Hz, 1H), 2.55 – 2.46 (m, 1H), 2.45 – 2.12 (m, 7H), 2.08 – 1.80 (m, 8H), 1.60 (q, *J* = 9.1 Hz, 1H), 1.21 (s, 3H), 1.14 (d, *J* = 8.5 Hz, 1H), 0.80 (s, 3H).

^{13}C NMR (101 MHz, $CDCl_3$) δ 174.8, 145.6, 135.7, 134.4, 134.3, 130.8, 129.9, 125.2, 122.0, 119.7, 119.5, 117.8, 58.6, 54.2, 47.4, 46.3, 42.1, 40.7, 39.2, 37.9, 33.3, 32.0, 31.6, 26.4, 22.5, 21.5, 17.1.

HRMS (ESI, m/z): $[M+H]^+$ Calcd. For $C_{27}H_{36}N_3O_3S$: 482.2477; Found: 482.2480.

HPLC (Chiralpak AD-H Column), *i*-PrOH/hexane = 10/90, flow rate = 1.0 mL/min, λ = 254 nm; t_R = 13.1 min (minor), t_R = 15.5 min (major).

[α]$_D^{25}$ = −13.6 (c = 0.5, $CHCl_3$).

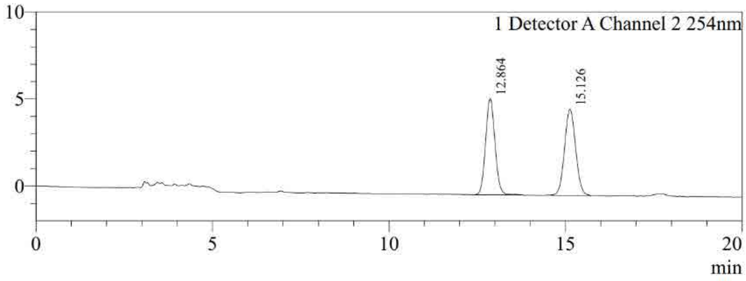

Detector A Channel 2 254nm

Peak#	Ret. Time	USP Width	Area	Height	Area%
1	12.864	0.491	101895	5526	48.440
2	15.126	0.578	108460	4965	51.560
Total			210355	10491	100.000

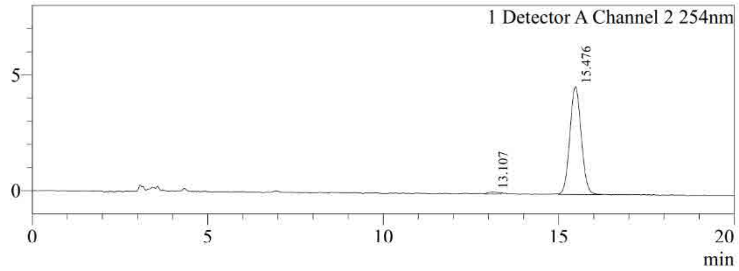

Detector A Channel 2 254nm

Peak#	Ret. Time	USP Width	Area	Height	Area%
1	13.107	0.599	1849	93	1.727
2	15.476	0.600	105228	4662	98.273
Total			107077	4755	100.000

Synthesis of ($R_{C\text{-}1}$, $R_{C\text{-}2}$)-25b

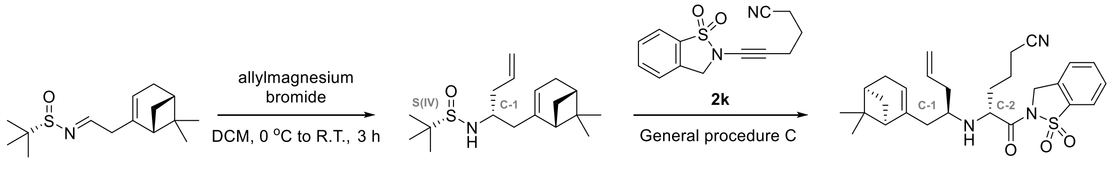

($S_{S(IV)}$, $R_{C\text{-}1}$)-**S25b** ($R_{C\text{-}2}$, $R_{C\text{-}1}$)-**25b**

Synthesis of ($S_{S(IV)}$, $R_{C\text{-}1}$)**-S25b** (*50*)

Under N_2 atmosphere, to the solution of (*S*)-sulfinimine (1.09 g, 4.1 mmol, 1.0 equiv) in dichoromethane (0.13 M) was added allylmagnesium bromide (6.2 mL, 1.0 M in Et_2O, 1.5 equiv) dropwise at 0 °C. The reaction was warmed to room temperature and stirred for 1 h, and then quenched by saturated aqueous NH_4Cl. After extraction with dichoromethane, the combined organic layer was washed with brine and concentrated under vacuo. The crude product was purified through flash chromatography (eluent: petroleum ether/ethyl acetate = 4:1 to 2:1) to afford ($S_{S(IV)}$, $R_{C\text{-}1}$)-**S25b** as colorless oil (610 mg, 48% yield, 99:1 d.r.).

^{1}H NMR (400 MHz, $CDCl_3$) δ 5.81 – 5.70 (m, 1H), 5.26 (s, 1H), 5.14 – 5.10 (m, 2H), 3.35 – 3.27 (m, 2H), 2.48 – 2.42 (m, 1H), 2.37 – 2.32 (m, 1H), 2.28 – 2.14 (m, 4H), 2.07 – 2.01 (m, 3H), 1.24 (s, 3H), 1.16 (s, 9H), 1.09 (d, J = 8.6 Hz, 1H), 0.81 (s, 3H).

HPLC (Chiralpak OD-H Column), *i*-PrOH/hexane = 1/99, flow rate = 1.0 mL/min, λ = 210 nm; t_R = 7.8 min (major), t_R = 9.8 min (minor).

$[\alpha]_D^{20}$ = +64.8 (c = 1.0, $CHCl_3$).

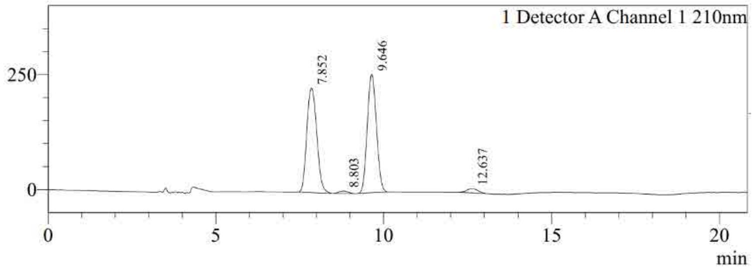

Detector A Channel 1 210nm

Peak#	Ret. Time	USP Width	Area	Height	Area%
1	7.852	0.543	4702229	226893	47.411
2	8.803	0.535	104799	5437	1.057
3	9.646	0.510	4887218	257391	49.276
4	12.637	0.638	223815	9662	2.257
Total			9918060	499383	100.000

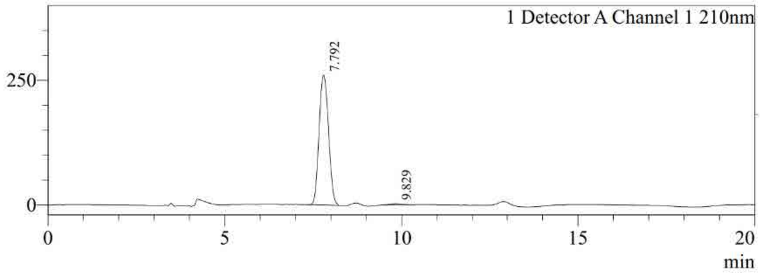

Detector A Channel 1 210nm

Peak#	Ret. Time	USP Width	Area	Height	Area%
1	7.792	0.498	4850651	260574	99.163
2	9.829	0.453	40949	2444	0.837
Total			4891600	263018	100.000

(*R*)-5-(((*R*)-1-((1*R*,5*S*)-6,6-Dimethylbicyclo[3.1.1]hept-2-en-2-yl)pent-4-en-2-yl)amino)-6-(1,1-dioxidobenzoisothiazol-2(3*H*)-yl)-6-oxohexanenitrile (25b)

Prepared according to the **GENERAL PROCEDURE C** with ($S_{S(IV)}$, $R_{C\text{-}1}$)-**S25b** (92.8 mg, 0.30 mmol, 99:1 d.r.) and **2k** (52.0 mg, 0.20 mmol) as substrates. Column chromatography: silica gel, petroleum ether/ethyl acetate/dichloromethane = 5:1:1. White solid (73.2 mg, 76% yield, 99:1 d.r.).

^{1}H NMR (400 MHz, $CDCl_3$) δ 7.83 (d, J = 7.9 Hz, 1H), 7.72 (t, J = 7.6 Hz, 1H), 7.61 (t, J = 7.7 Hz, 1H), 7.49 (d, J = 7.8 Hz, 1H), 5.74 (tdd, J = 17.1, 8.7, 5.5 Hz, 1H), 5.25 (s, 1H), 5.13 (s, 1H), 5.10 (d, J = 6.4 Hz, 1H), 5.02 (d, J = 15.9 Hz, 1H), 4.90 (d, J = 15.9 Hz, 1H), 4.20 – 4.07 (m, 1H), 2.54 (tt, J = 8.5, 3.6 Hz, 1H), 2.46 – 2.16 (m, 7H), 2.12 – 1.81 (m, 8H), 1.59 (qd, J = 12.9, 11.5, 6.4 Hz, 1H), 1.18 (s, 3H), 1.07 (d, J = 8.5 Hz, 1H), 0.84 (s, 3H).

^{13}C NMR (101 MHz, $CDCl_3$) δ 175.0, 145.6, 135.8, 134.3, 130.9, 129.9, 125.2, 122.0, 119.7, 119.3, 117.8, 58.5, 53.6, 47.4, 45.7, 42.3, 40.8, 39.1, 38.1, 33.4, 31.8, 31.6, 26.3, 22.5, 21.1, 17.1.

HRMS (ESI, m/z): $[M+H]^+$ Calcd. For $C_{27}H_{36}N_3O_3S$: 482.2477; Found: 482.2479.

HPLC (Chiralpak AD-H Column), *i*-PrOH/hexane = 10/90, flow rate = 1.0 mL/min, λ = 254 nm; t_R = 13.1 min (major), t_R = 15.5 min (minor).

$[\alpha]_D^{25}$ = +4.80 (c = 0.5, $CHCl_3$).

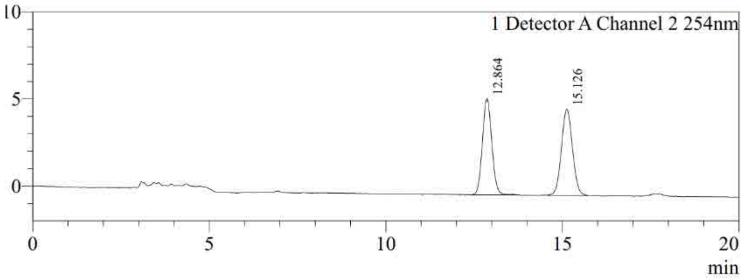

Detector A Channel 2 254nm

Peak#	Ret. Time	USP Width	Area	Height	Area%
1	12.864	0.491	101895	5526	48.440
2	15.126	0.578	108460	4965	51.560
Total			210355	10491	100.000

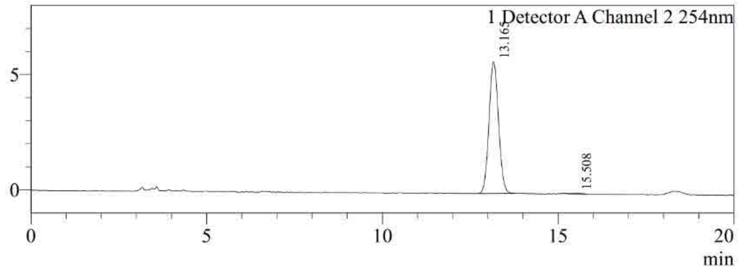

Detector A Channel 2 254nm

Peak#	Ret. Time	USP Width	Area	Height	Area%
1	13.165	0.505	108214	5714	99.274
2	15.508	0.641	792	37	0.726
Total			109005	5751	100.000

Synthesis of ($R_{C\text{-}1}$, $S_{C\text{-}2}$)-26a

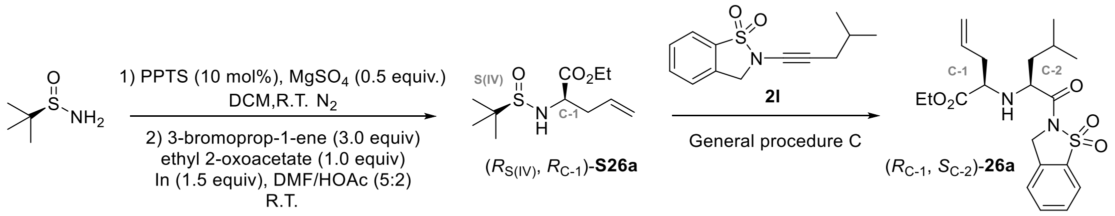

Synthesis of ($R_{S(IV)}$, $R_{C\text{-}1}$)**-S26a** (*52*)

Step1: Under N_2 atmosphere, the mixture of (*R*)-*tert*-butanesulfinamide (2.42 g, 20 mmol, 1.0 equiv), ethyl 2-oxoacetate (4.10 g, 20 mmol, 1.0 equiv), pyridinium *p*-toluenesulfonate (PPTS) (503 mg, 2.0 mmol, 0.1 equiv) and $MgSO_4$ (1.21 g, 10 mmol, 0.5 equiv) in dichoromethane was stirred for 48 hours. After completion, the mixture was filtered through a celite and the filtrate was concentrated under vacuo. The crude product was purified through flash chromatography (eluent: petroleum ether/ethyl acetate = 10:1) to afforded imine as light-yellow oil (2.70 g, 66% yield), which was used for the next step.

Step2: Under N_2 atmosphere, to the solution of imine (820 mg, 4.0 mmol, 1.0 equiv) and 3-bromoprop-1-ene (1.45 g, 12 mmol, 3.0 equiv) in DMF (4.0 mL) and HOAc (1.6 mL) was added indium (689 mg, 6.0 mmol, 1.5 equiv) and the mixture was stirred vigorously at room temperature for 12 h. After that, the reaction was quenched with ice water and extracted with ethyl acetate. The combined organic layer was washed with brine and concentrated under vacuo. The crude product was purified through flash chromatography (eluent: petroleum ether/ethyl acetate = 3:1) to afford the desired products ($R_{S(IV)}$, R_{C-1})-**S26a** as colorless oil (600 mg, 61% yield, >20:1 d.r., signal of diastereomers cannot be observed in ^{1}H NMR).

^{1}H NMR (400 MHz, $CDCl_3$) δ 5.77 – 5.55 (m, 1H), 5.11 – 5.05 (m, 1H), 5.04 (s, 1H), 4.17 (qd, *J* = 7.2, 1.9 Hz, 2H), 4.10 (d, *J* = 6.6 Hz, 1H), 3.98 (q, *J* = 6.1 Hz, 1H), 2.46 (t, *J* = 6.4 Hz, 2H), 1.24 (t, *J* = 7.3 Hz, 3H), 1.19 (s, 9H).

$[\alpha]_D^{20}$ = –69.6 (c = 1.0, $CHCl_3$).

Ethyl-(*R*)-2-(((*S*)-1-(1,1-dioxidobenzo[*d*]isothiazol-2(3*H*)-yl)-4-methyl-1-oxopentan-2-yl)amino)-pent-4-enoate (26a)

Prepared according to the **GENERAL PROCEDURE C** with ($R_{S(IV)}$, R_{C-1})-**S26a** (74.2 mg, 0.30 mmol, >20:1 d.r.) and **2l** (49.8 mg, 0.20 mmol) as substrates. *(**Note:** Work up with 6M HCl (0.6 mL) and 40 mol% $HNTf_2$ overnight after the rearrangement reaction.)* Column chromatography: silica gel, petroleum ether/ethyl acetate/dichloromethane = 15:1:1. (staining using $KMnO_4$) Light-yellow oil (71.4 mg, 87% yield, >20:1 d.r., signal of diastereomers cannot be observed in ^{1}H NMR).

^{1}H NMR (400 MHz, $CDCl_3$) δ 7.79 (d, *J* = 7.9 Hz, 1H), 7.70 (t, *J* = 7.6 Hz, 1H), 7.58 (t, *J* = 7.6 Hz, 1H), 7.47 (d, *J* = 7.8 Hz, 1H), 5.81 (ddt, *J* = 17.1, 10.1, 7.0 Hz, 1H), 5.10 (d, *J* = 17.0 Hz, 1H), 5.05 (d, *J* = 10.0 Hz, 1H), 4.99 (d, *J* = 15.9 Hz, 1H), 4.87 (d, *J* = 15.9 Hz, 1H), 4.21 – 4.00 (m, 3H), 3.39 (t, *J* = 5.8 Hz, 1H), 2.47 (dq, *J* = 26.4, 7.5 Hz, 3H), 2.03 (dtd, *J* = 10.6, 6.7, 4.2 Hz, 1H), 1.55 (dddd, *J* = 32.4, 13.8, 9.7, 3.9 Hz, 2H), 1.19 (t, *J* = 7.2 Hz, 3H), 0.98 (d, *J* = 6.6 Hz, 3H), 0.94 (d, *J* = 6.7 Hz, 3H).

^{13}C NMR (101 MHz, $CDCl_3$) δ 175.2, 173.9, 134.5, 134.2, 133.9, 131.0, 129.8, 125.1, 121.8, 117.8, 60.9, 59.9, 58.9, 47.4, 43.3, 37.2, 24.7, 23.7, 21.3, 14.3.

HRMS (ESI, m/z): $[M+H]^+$ Calcd. For $C_{20}H_{29}N_2O_5S$: 409.1797; Found: 409.1799.

$[\alpha]_D^{25}$ = –12.1 (c = 0.5, $CHCl_3$).

Synthesis of (R_{C-1}, S_{C-2})-27a

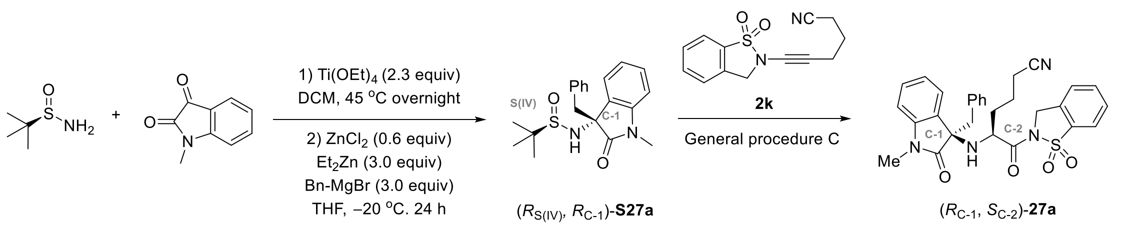

Synthesis of ($R_{S(IV)}$, R_{C-1})-**S27a**

Step1: Preparation of sulfinimine according to the known literature (*77*): The mixture of *N*-methylisatin (805 mg, 5.0 mmol, 1.0 equiv), (*R*)-*tert*-butanesulfinamide (726 mg, 6.0 mmol, 1.2 equiv) and titanium ethoxide (2.60 g, 11.4 mmol. 2.3 equiv) in dichoromethane (20 mL) was refluxed overnight. Once isatin was completely consumed, 10 mL of saturated aqueous $NaHCO_3$ was added to the reaction and the reasulting mixture was stirred vigorously for another 30 min before filtration. The filter was extracted with dichoromethane and the combined organic layer was washed with brine, concentrated under vacuo. The crude product was purified immediately via flash chromatography on silica gel (eluent: petroleum ether/ethyl acetate/dichloromethane = 10:1:1 to 4:1:1) to afford the corresponding ketimine, which was used inmediately (*Note, liable to decomposition*).

Step2: Under N_2 atmosphere, the mixture of (*R*)-sulfinimine (~4.5 mmol, 1.0 equiv) prepared above and $ZnCl_2$ (366 mg, 2.7 mmol, 0.6 equiv) in THF (30 mL) was stirred at −20 °C for 30 minutes, followed by addition of benzylmagnesium bromide (13.5 mL, 1.0 M in THF, 3.0 equiv) and Et_2Zn (13.5 mL, 1.0 M in hexane, 3.0 equiv), dropwise. The resulting solution was stirred at the same temperature for 24 h, and then quenched with saturated aqueous NH_4Cl, extracted with ethyl acetate. The combined organic layer was washed with brine and concentrated under vacuo. The crude product was purified through flash chromatography (eluent: petroleum ether/ethyl acetate = 2:1) to afford ($R_{S(IV)}$, $R_{C\text{-}1}$)-**S27a** as yellow solid (664 mg, 37% yield, 96.5:3.5 d.r.).

^{1}H NMR (400 MHz, $CDCl_3$) δ 7.51 (d, *J* = 7.4 Hz, 1H), 7.20 (t, *J* = 7.7 Hz, 1H), 7.07 (t, *J* = 7.6 Hz, 1H), 7.04 – 6.96 (m, 3H), 6.79 (d, *J* = 7.2 Hz, 2H), 6.53 (d, *J* = 7.9 Hz, 1H), 4.08 (s, 1H), 3.33 (q, *J* = 12.7 Hz, 2H), 2.88 (s, 3H), 1.16 (d, *J* = 2.5 Hz, 9H).

^{13}C NMR (101 MHz, $CDCl_3$) δ 175.4, 143.1, 133.5, 130.0, 129.5, 128.0, 127.7, 126.9, 125.7, 122.6, 108.1, 65.9, 56.6, 45.6, 25.9, 22.4.

HRMS (ESI, m/z): $[M+Na]^+$ Calcd. For $C_{20}H_{24}N_2O_2NaS$: 379.1456; Found: 379.1459.

HPLC (Chiralpak OD-H Column), *i*-PrOH/hexane = 5/95, flow rate = 1.0 mL/min, λ = 210 nm; t_R = 10.8 min (major), t_R = 12.6 min (minor).

$[\alpha]_D^{25}$ = −63.4 (c = 1.0, $CHCl_3$).

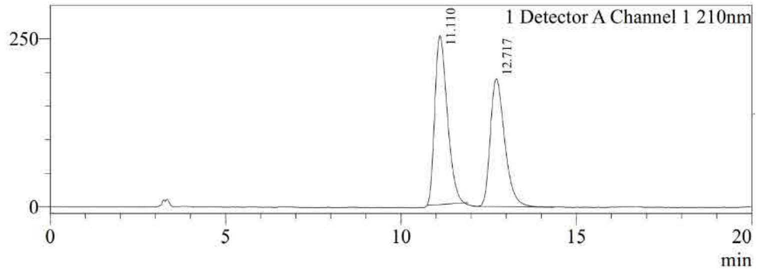

Detector A Channel 1 210nm

Peak#	Ret. Time	USP Width	Area	Height	Area%
1	11.110	0.660	6231686	251627	53.207
2	12.717	0.743	5480387	190554	46.793
Total			11712073	442182	100.000

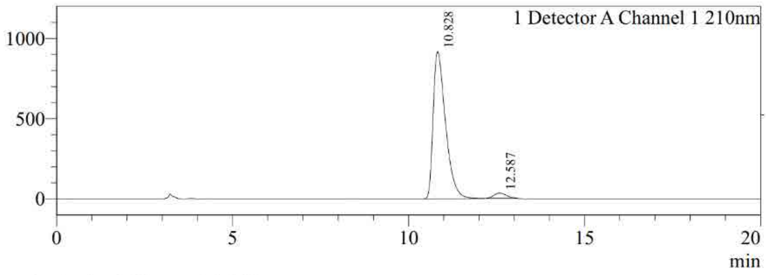

Detector A Channel 1 210nm

Peak#	Ret. Time	USP Width	Area	Height	Area%
1	10.828	0.635	22403641	917015	96.500
2	12.587	0.677	812489	32892	3.500
Total			23216129	949907	100.000

(*S*)-5-(((*R*)-3-Benzyl-1-methyl-2-oxoindolin-3-yl)amino)-6-(1,1-dioxidobenzoisothiazol-2(3*H*)-yl)-6-oxohexanenitrile (27a)

Prepared according to the **GENERAL PROCEDURE C** with ($R_{S(IV)}$, $R_{C\text{-}1}$)-**S27a** (107 mg, 0.30 mmol, 96.5:3.5 d.r.) and **2k** (52.0 mg, 0.20 mmol) as substrates. Column chromatography: silica gel, petroleum ether/ethyl acetate/dichloromethane = 3:1:1. Off-white solid (73.5 mg, 69% yield, >99:1 d.r.).

^{1}H NMR (400 MHz, $CDCl_3$) δ 7.70 – 7.60 (m, 2H), 7.52 (t, *J* = 7.7 Hz, 1H), 7.36 (d, *J* = 7.8 Hz, 1H), 7.14 (d, *J* = 7.3 Hz, 1H), 7.02 (p, *J* = 7.1 Hz, 4H), 6.93 (t, *J* = 7.5 Hz, 1H), 6.82 (d, *J* = 6.9 Hz, 2H), 6.47 (d, *J* = 7.7 Hz, 1H), 4.72 (d, *J* = 15.8 Hz, 1H), 4.37 (d, *J* = 15.8 Hz, 1H), 3.69 (dd, *J* = 9.8, 2.7 Hz, 1H), 3.20 (d, *J* = 12.5 Hz, 1H), 3.13 (d, *J* = 12.6 Hz, 1H), 2.94 (s, 3H), 2.65 – 2.51 (m, 2H), 2.42 (dt, *J* = 16.5, 7.0 Hz, 1H), 2.03 (tp, *J* = 13.8, 6.9 Hz, 2H), 1.89 (dd, *J* = 13.9, 7.6 Hz, 1H), 1.60 (ddd, *J* = 15.7, 10.0, 6.4 Hz, 1H).

^{13}C NMR (101 MHz, $CDCl_3$) δ 176.9, 173.9, 144.0, 134.2, 133.9, 130.6, 130.2, 129.7, 129.0, 127.9, 127.6, 126.8, 125.8, 125.0, 121.7, 121.4, 120.0, 108.5, 67.6, 56.7, 47.0, 44.4, 33.1, 26.0, 22.3, 16.7.

HRMS (ESI, m/z): $[M+H]^+$ Calcd. For $C_{29}H_{29}N_4O_4S$: 529.1910; Found: 529.1911.

HPLC (Chiralpak OD-H Column), *i*-PrOH/hexane = 25/75, flow rate = 1.0 mL/min, λ = 254 nm; t_R = 14.3 min (major), t_R = 16.6 min (minor).

$[\alpha]_D^{25}$ = –100.8 (c = 0.5, $CHCl_3$).

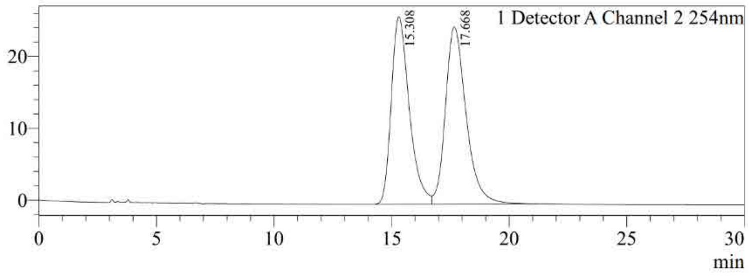

Detector A Channel 2 254nm

Peak#	Ret. Time	USP Width	Area	Height	Area%
1	15.308	1.369	1391485	26133	47.976
2	17.668	1.551	1508922	24668	52.024
Total			2900407	50800	100.000

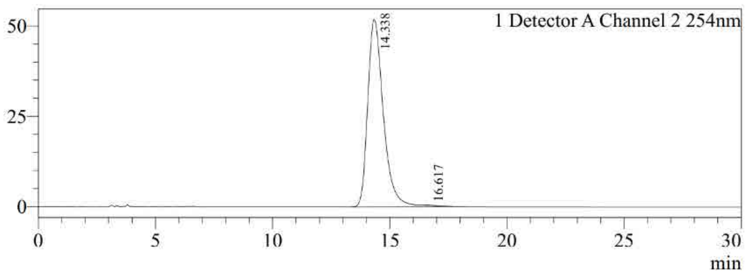

Detector A Channel 2 254nm

Peak#	Ret. Time	USP Width	Area	Height	Area%
1	14.338	1.172	2414319	51876	99.926
2	16.617	0.584	1784	86	0.074
Total			2416103	51962	100.000

Synthesis of ($S_{C\text{-}1}$, $R_{C\text{-}2}$)-27b

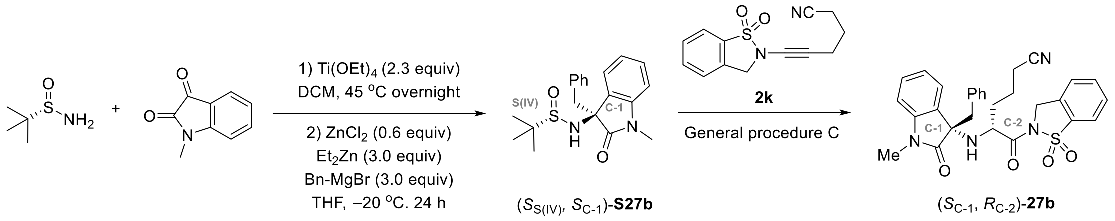

Synthesis of ($S_{S(IV)}$, $S_{C\text{-}1}$)-**S27b** (*78*)

Step1: Procedure is the same as **S27a**.

Step2: Under N_2 atmosphere, the mixture of (*S*)-sulfinimine (~4.5 mmol, 1.0 equiv) prepared above and $ZnCl_2$ (366 mg, 2.7 mmol, 0.6 equiv) in THF (30 mL) was stirred at −20 °C for 30 minutes, followed by addition of benzylmagnesium bromide (13.5 mL, 1.0 M in THF, 3.0 equiv) and Et_2Zn (13.5 mL, 1.0 M in hexane, 3.0 equiv), dropwise. The resulting solution was stirred at the same temperature for 24 h, and then quenched with saturated aqueous NH_4Cl, extracted with ethyl acetate. The combined organic layer was washed with brine and concentrated under vacuo. The crude product was purified through flash chromatography (eluent: petroleum ether/ethyl acetate = 2:1) to afford ($S_{S(IV)}$, $S_{C\text{-}1}$)-^{t}BS-**19b** as yellow solid (883 mg, 49% yield, 90:10 d.r.).

^{1}H NMR (400 MHz, $CDCl_3$) δ 7.53 (dd, *J* = 7.4, 1.3 Hz, 1H), 7.27 – 7.21 (m, 1H), 7.15 – 7.00 (m, 4H), 6.86 – 6.80 (m, 2H), 6.58 (d, *J* = 7.8 Hz, 1H), 4.01 (s, 1H), 3.36 (q, *J* = 12.6 Hz, 2H), 2.93 (s, 3H), 1.19 (s, 9H).

HPLC (Chiralpak OD-H Column), *i*-PrOH/hexane= 5/95, flow rate = 1.0 mL/min, λ = 210 nm; t_R = 10.9 min (minor), t_R = 12.4 min (major).

$[\alpha]_D^{25}$ = +49.8 (c = 1.0, $CHCl_3$).

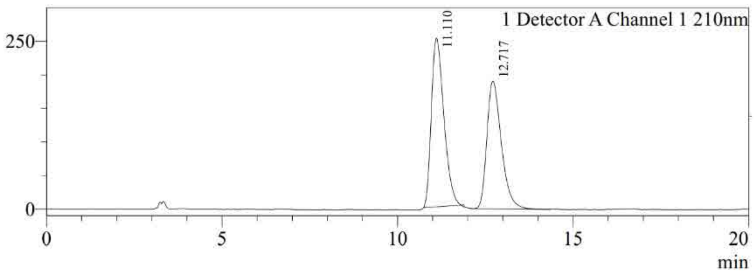

Detector A Channel 1 210nm

Peak#	Ret. Time	USP Width	Area	Height	Area%
1	11.110	0.660	6231686	251627	53.207
2	12.717	0.743	5480387	190554	46.793
Total			11712073	442182	100.000

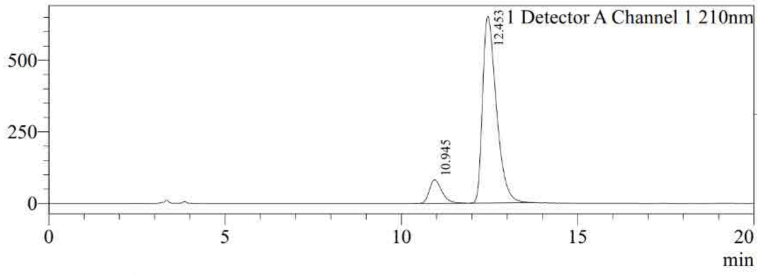

Detector A Channel 1 210nm

Peak#	Ret. Time	USP Width	Area	Height	Area%
1	10.945	0.646	2044207	82586	10.300
2	12.453	0.709	17801530	653037	89.700
Total			19845737	735624	100.000

(*R*)-5-(((*S*)-3-Benzyl-1-methyl-2-oxoindolin-3-yl)amino)-6-(1,1-dioxidobenzoisothiazol-2(3*H*)-yl)-6-oxohexanenitrile (27b)

Prepared according to the **GENERAL PROCEDURE C** with ($S_{S(IV)}$, $S_{C\text{-}1}$)-**S27b** (107 mg, 0.30 mmol, 90:10 d.r.) and **2k** (52.0 mg, 0.30 mmol) as substrates. Column chromatography: silica gel, petroleum ether/ethyl acetate/dichloromethane = 3:1:1. Off-white solid (87.3 mg, 82% yield, 99:1 d.r.).

^{1}H NMR (400 MHz, $CDCl_3$) δ 7.71 – 7.59 (m, 2H), 7.51 (t, *J* = 7.7 Hz, 1H), 7.35 (d, *J* = 7.8 Hz, 1H), 7.13 (d, *J* = 7.3 Hz, 1H), 7.01 (dd, *J* = 13.4, 7.2 Hz, 4H), 6.92 (t, *J* = 7.5 Hz, 1H), 6.82 (d, *J* = 6.9 Hz, 2H), 6.46 (d, *J* = 7.7 Hz, 1H), 4.71 (d, *J* = 15.7 Hz, 1H), 4.36 (d, *J* = 15.8 Hz, 1H), 3.68 (dd, *J* = 9.8, 2.6 Hz, 1H), 3.20 (d, *J* = 12.5 Hz, 1H), 3.13 (d, *J* = 12.5 Hz, 1H), 2.93 (s, 3H), 2.62 (dq, *J* = 15.2, 8.2, 7.1 Hz, 2H), 2.41 (dt, *J* = 16.7, 7.0 Hz, 1H), 2.02 (ddp, *J* = 20.8, 13.9, 6.9 Hz, 2H), 1.89 (dd, *J* = 13.9, 7.6 Hz, 1H), 1.60 (ddd, *J* = 15.9, 10.2, 6.3 Hz, 1H).

^{13}C NMR (101 MHz, $CDCl_3$) δ 176.8, 173.9, 144.0, 134.2, 134.1, 134.0, 130.6, 130.2, 129.6, 128.9, 127.9, 127.6, 126.8, 125.8, 125.0, 121.7, 121.4, 119.9, 108.5, 67.5, 56.7, 47.0, 44.4, 33.0, 25.9, 22.3, 16.7.

HRMS (ESI, m/z): $[M+H]^+$ Calcd. For $C_{29}H_{29}N_4O_4S$: 529.1910; Found: 529.1915.

HPLC (Chiralpak OD-H Column), *i*-PrOH/hexane = 25/75, flow rate = 1.0 mL/min, λ = 254 nm; t_R = 14.3 min (minor), t_R = 16.4 min (major).

$[\alpha]_D^{25}$ = +108.8 (c = 0.5, $CHCl_3$).

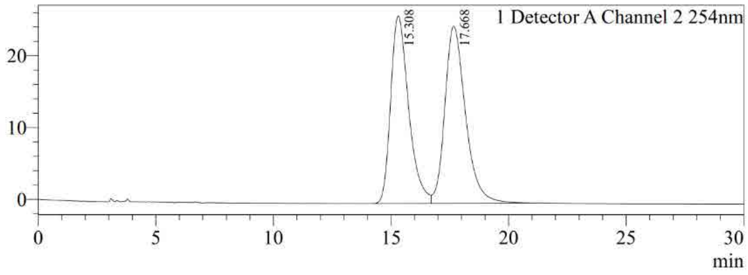

Detector A Channel 2 254nm

Peak#	Ret. Time	USP Width	Area	Height	Area%
1	15.308	1.369	1391485	26133	47.976
2	17.668	1.551	1508922	24668	52.024
Total			2900407	50800	100.000

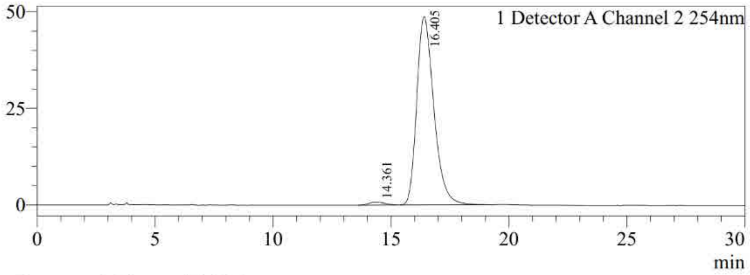

Detector A Channel 2 254nm

Peak#	Ret. Time	USP Width	Area	Height	Area%
1	14.361	1.173	34844	809	1.396
2	16.405	1.305	2461710	48703	98.604
Total			2496554	49512	100.000

Synthesis of ($S_{C\text{-}1}$, $S_{C\text{-}2}$)-28a

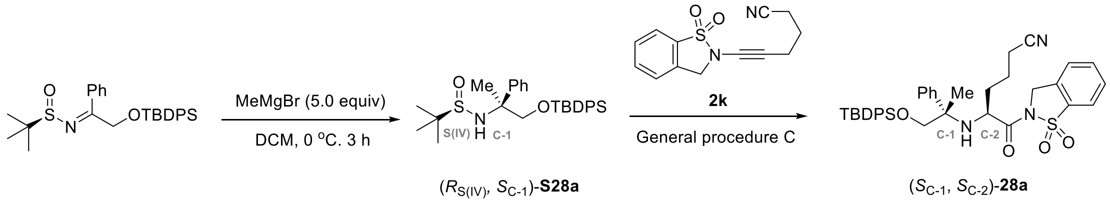

Synthesis of ($R_{S(IV)}$, $S_{C\text{-}1}$)-S28a

Under N_2 atmosphere, to the solution of methylmagnesium bromide (26 mL, 1.0 M in THF, 5.0 equiv) was added (*R*)-sulfinimine (2.50 g, 5.2 mmol, 1.0 equiv) dissolved in dichoromethane (0.13 M) dropwise at 0 °C. The reaction was warmed to room temperature and stirred for 20 minutes, and then quenched by HCl (47 mL, 0.5 M). After extraction with dichloromethane, the combined organic layer was washed with brine and concentrated under vacuo. The crude product was purified through flash chromatography (eluent: petroleum ether/ethyl acetate = 20:1 to 10:1) to afford ($R_{S(IV)}$, $S_{C\text{-}1}$)-**S28a** as colorless oil (1.69 g, 65% yield, >99:1 d.r.).

^{1}H NMR (400 MHz, $CDCl_3$) δ 7.59 (d, *J* = 7.0 Hz, 2H), 7.54 (d, *J* = 7.8 Hz, 2H), 7.45 – 7.29 (m, 11H), 4.39 (s, 1H), 3.91 (d, *J* = 9.5 Hz, 1H), 3.80 (d, *J* = 9.7 Hz, 1H), 1.68 (s, 3H), 1.28 (s, 9H), 1.01 (s, 9H).

^{13}C NMR (101 MHz, $CDCl_3$) δ 143.9, 135.7, 135.6, 133.0, 132.6, 129.9, 129.8, 128.2, 127.8, 127.7, 127.3, 127.1, 72.5, 61.9, 56.1, 26.8, 25.1, 22.8, 19.3.

HRMS (ESI, m/z): $[M+Na]^+$ Calcd. For $C_{29}H_{39}NO_2NaSSi$: 516.2368; Found: 516.2372.

HPLC (Chiralpak OD-H Column), *i*-PrOH/hexane = 5/95, flow rate = 1.0 mL/min, λ = 210 nm; t_R = 4.8 min (major), t_R = 5.3 min (minor).

$[\alpha]_D^{25}$ = −1.00 (c = 1.0, $CHCl_3$).

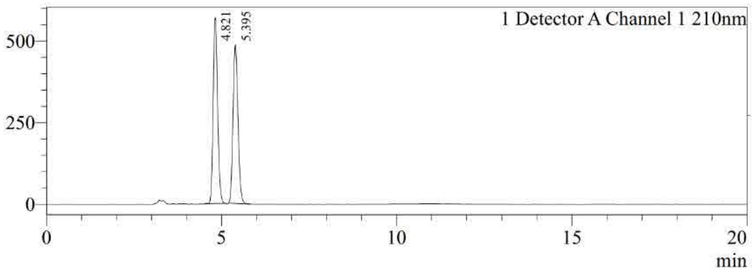

Detector A Channel 1 210nm

Peak#	Ret. Time	USP Width	Area	Height	Area%
1	4.821	0.241	5019146	569184	51.134
2	5.395	0.270	4796447	486291	48.866
Total			9815593	1055474	100.000

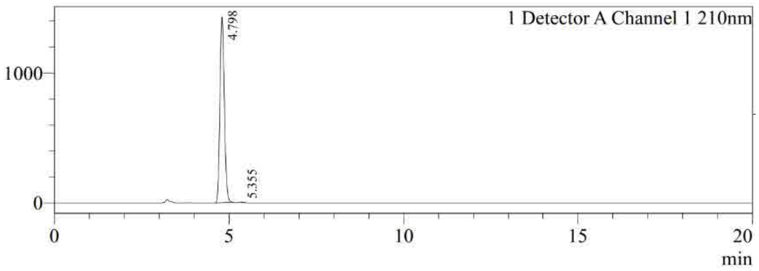

Detector A Channel 1 210nm

Peak#	Ret. Time	USP Width	Area	Height	Area%
1	4.798	0.240	12527697	1428394	99.985
2	5.355	0.110	1921	196	0.015
Total			12529618	1428590	100.000

(*S*)-5-(((*S*)-1-((*tert*-Butyldiphenylsilyl)oxy)-2-phenylpropan-2-yl)amino)-6-(1,1-dioxidobenzol-isothiazol-2(3*H*)-yl)-6-oxohexanenitrile (28a)

Prepared according to the **GENERAL PROCEDURE C** with ($R_{S(IV)}$, $S_{C\text{-}1}$)-**S28a** (148 mg, 0.30 mmol, >99:1 d.r.) and **2k** (52.0 mg, 0.20 mmol) as substrates. Column chromatography: silica gel, petroleum ether/ethyl acetate/dichloromethane = 7:1:1. White solid (99.5 mg, 75% yield, >99:1 d.r.).

^{1}H NMR (400 MHz, $CDCl_3$) δ 7.76 (d, *J* = 7.9 Hz, 1H), 7.70 (t, *J* = 7.6 Hz, 1H), 7.63 – 7.49 (m, 7H), 7.38 (d, *J* = 7.8 Hz, 1H), 7.33 – 7.17 (m, 9H), 4.68 (d, *J* = 15.8 Hz, 1H), 4.27 (d, *J* = 15.8 Hz, 1H), 3.92 – 3.73 (m, 3H), 2.78 (s, 1H), 2.18 (dt, *J* = 15.9, 7.5 Hz, 1H), 2.06 (dt, *J* = 16.4, 7.0 Hz, 1H), 1.97 – 1.70 (m, 3H), 1.60 (s, 3H), 1.52 (t, *J* = 8.4 Hz, 1H), 1.05 (s, 9H).

^{13}C NMR (101 MHz, $CDCl_3$) δ 174.5, 143.7, 135.9, 135.7, 134.1, 133.5, 133.3, 130.9, 129.7, 129.63, 129.60, 127.8, 127.7, 127.61, 127.58, 126.9, 125.1, 121.8, 119.9, 70.6, 59.7, 54.5, 47.2, 34.3, 26.9, 24.2, 22.0, 19.3, 16.5.

HRMS (ESI, m/z): $[M+H]^+$ Calcd. For $C_{38}H_{44}N_3O_4SSi$: 666.2822; Found: 666.2825.

HPLC (Chiralpak OD-H Column), *i*-PrOH/hexane = 10/90, flow rate = 1.0 mL/min, λ = 254 nm; t_R = 13.0 min (major), t_R = 17.7 min (minor).

[α]D[25] = –24.0 (c = 0.5, $CHCl_3$).

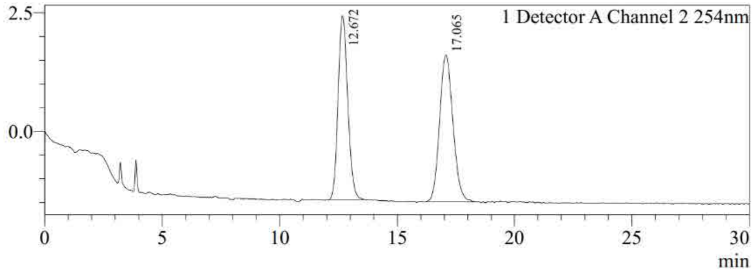

Detector A Channel 2 254nm

Peak#	Ret. Time	USP Width	Area	Height	Area%
1	12.672	0.754	111019	3882	47.631
2	17.065	1.041	122062	3092	52.369
Total			233081	6973	100.000

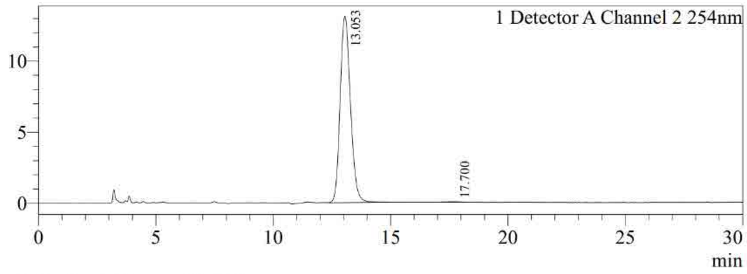

Detector A Channel 2 254nm

Peak#	Ret. Time	USP Width	Area	Height	Area%
1	13.053	0.801	399766	13112	99.656
2	17.700	0.870	1381	43	0.344
Total			401147	13155	100.000

Synthesis of (*R*C-1, *R*C-2)-28b

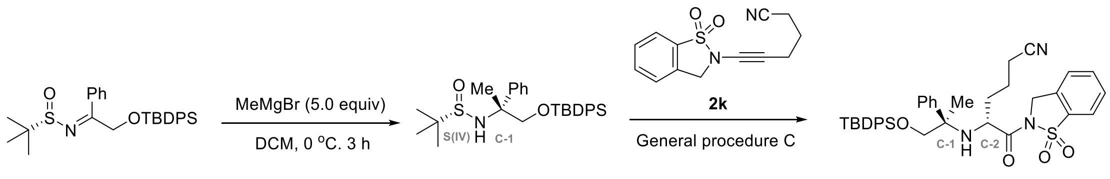

($S_{S(IV)}$, R_{C-1})-**S28b** (R_{C-2}, R_{C-1})-**28b**

Synthesis of ($S_{S(IV)}$, R_{C-1})-**S28b** (*51*)

Under N_2 atmosphere, to the solution of methylmagnesium bromide (7.8 mL, 1.0 M in THF, 5.0 equiv) was added (*S*)-sulfinimine (742 mg, 1.55 mmol, 1.0 equiv) dissolved in dichoromethane (0.13 M) dropwise at 0 °C. The reaction was warmed to room temperature and stirred for 20 minutes, and then quenched by HCl (14 mL, 0.5 M). After extraction with dichoromethane, the combined organic layer was washed with brine and concentrated under vacuo. The crude product was purified through flash chromatography (eluent: petroleum ether/ethyl acetate = 20:1 to 10:1) to afford ($S_{S(IV)}$, R_{C-1})-**S28b** as colorless oil (450 mg, 59% yield, 95:5 d.r.).

^{1}H NMR (400 MHz, $CDCl_3$) δ 7.56 – 7.47 (m, 4H), 7.45 – 7.26 (m, 11H), 4.32 (s, 1H), 3.87 (d, *J* = 9.6 Hz, 1H), 3.76 (d, *J* = 9.6 Hz, 1H), 1.65 (s, 3H), 1.25 (s, 9H), 0.97 (s, 9H).

HPLC (Chiralpak OD-H Column), *i*-PrOH/hexane = 5/95, flow rate = 1.0 mL/min, λ = 210 nm; t_R = 4.8 min (minor), t_R = 5.3 min (major).

[α]D^{25} = +0.20 (c = 1.0, $CHCl_3$).

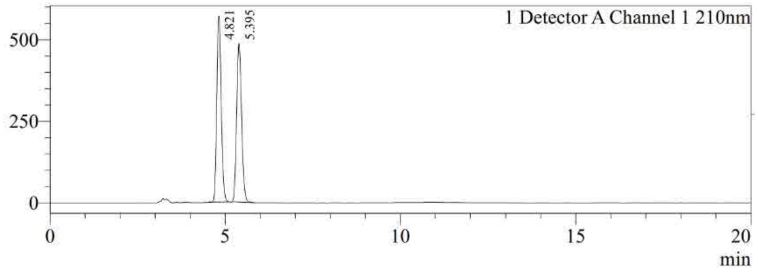

Detector A Channel 1 210nm

Peak#	Ret. Time	USP Width	Area	Height	Area%
1	4.821	0.241	5019146	569184	51.134
2	5.395	0.270	4796447	486291	48.866
Total			9815593	1055474	100.000

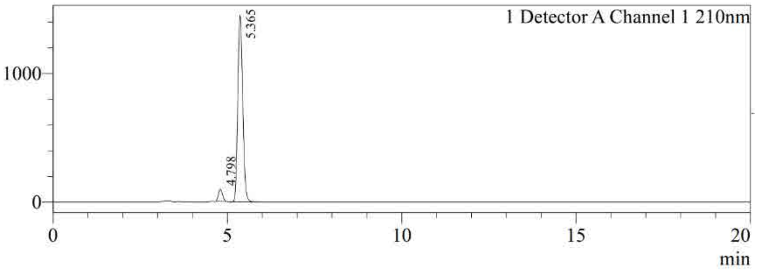

Detector A Channel 1 210nm

Peak#	Ret. Time	USP Width	Area	Height	Area%
1	4.798	0.227	747869	93184	4.938
2	5.365	0.269	14396048	1447836	95.062
Total			15143916	1541020	100.000

(*R*)-5-(((*R*)-1-((*tert*-Butyldiphenylsilyl)oxy)-2-phenylpropan-2-yl)amino)-6-(1,1-dioxidobenzo-isothiazol-2(3*H*)-yl)-6-oxohexanenitrile (28b)

Prepared according to the **GENERAL PROCEDURE C** with ($S_{S(IV)}$, R_{C-1})-**S28b** (148 mg, 0.30 mmol, 95:5 d.r.) and **2k** (52.0 mg, 0.20 mmol) as substrates. Column chromatography: silica gel, petroleum ether/ethyl acetate/dichloromethane = 7:1:1. White solid (106.5 mg, 80% yield, >99:1 d.r.).

^{1}H NMR (400 MHz, $CDCl_3$) δ 7.79 (d, *J* = 7.9 Hz, 1H), 7.72 (t, *J* = 7.8 Hz, 1H), 7.65 – 7.53 (m, 7H), 7.40 (d, *J* = 7.8 Hz, 1H), 7.36 – 7.26 (m, 8H), 7.24 (d, *J* = 7.2 Hz, 1H), 4.71 (d, *J* = 16.0 Hz, 1H), 4.30 (d, *J* = 15.8 Hz, 1H), 3.97 – 3.74 (m, 3H), 2.81 (s, 1H), 2.21 (dt, *J* = 15.6, 7.5 Hz, 1H), 2.09 (dt, *J* = 16.9, 9.1 Hz, 1H), 1.99 -1.73 (m, 4H), 1.63 (s, 3H), 1.08 (s, 9H).

^{13}C NMR (101 MHz, $CDCl_3$) δ 174.4, 143.6, 135.8, 135.7, 134.1, 133.5, 133.2, 130.9, 129.7, 129.61, 129.58, 127.8, 127.7, 127.59, 127.57, 126.9, 125.1, 121.7, 119.8, 70.5, 59.7, 54.5, 47.2, 34.2, 26.9, 24.2, 22.0, 19.3, 16.5.

HRMS (ESI, m/z): $[M+H]^+$ Calcd. For $C_{38}H_{44}N_3O_4SSi$: 666.2822; Found: 666.2824.

HPLC (Chiralpak OD-H Column), *i*-PrOH/hexane = 10/90, flow rate = 1.0 mL/min, λ = 254 nm; t_R = 13.2 min (minor), t_R = 17.6 min (major).

[α]D^{25} = +19.3 (c = 0.5, $CHCl_3$).

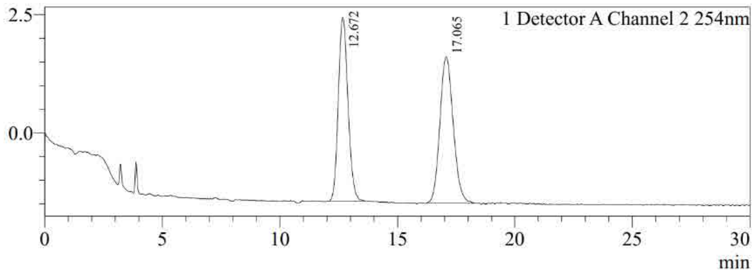

Detector A Channel 2 254nm

Peak#	Ret. Time	USP Width	Area	Height	Area%
1	12.672	0.754	111019	3882	47.631
2	17.065	1.041	122062	3092	52.369
Total			233081	6973	100.000

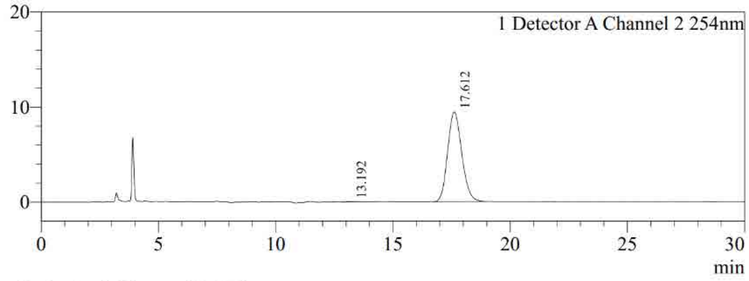

Detector A Channel 2 254nm

Peak#	Ret. Time	USP Width	Area	Height	Area%
1	13.192	0.476	939	36	0.236
2	17.612	1.106	396216	9435	99.764
Total			397155	9471	100.000

Synthesis of ($S_{\text{C-1}}$, $S_{\text{C-2}}$, $S_{\text{C-3}}$)-29a

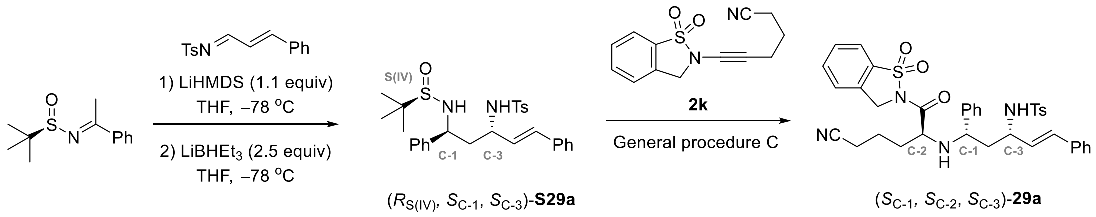

Synthesis of ($R_{\text{S(IV)}}$, $S_{\text{C-1}}$, $S_{\text{C-3}}$)**-S29a** (*53*)

Step 1: Under N_2 atmosphere, to the solution of sulfinimine (90.0 mg, 0.40 mmol, 1.0 equiv) in THF (3.0 mL) was added LiHMDS (0.44 mL, 1.0 M in THF, 1.1 equiv) at −78 °C, dropwise. The resulting mixture was stirred at this temperature for 1 h and imine (217 mg, 0.80 mmol, 2.0 equiv) in THF (3.0 mL) was added in one portion. The mixture was stirred continuely at −78 °C for 2 h, and then quenched by saturated aqueous NH_4Cl. After extraction with ethyl acetate, the combined organic layer was washed with brine and concentrated under vacuo. The crude product was purified through flash chromatography (eluent: petroleum ether/ethyl acetate = 10:1 to 5:1,) to afford *β*-amino-sulfinyl imine, which was used directly for the next step.

Step 2: Under N_2 atmosphere, to the solution of *β*-amino-sulfinyl imine (407 mg, 0.80 mmol, 1.0 equiv) was added lithium triethylborohydride (2.0 mL, 1.0 M in THF, 2.5 equiv) at −78 °C, dropwise. The resulting mixture was stirred at this temperature for 3 h and then quenched by saturated aqueous NH_4Cl. After extraction with ethyl acetate, the combined organic layer was washed with brine and concentrated under vacuo. The crude product was

purified through flash chromatography (eluent: petroleum ether/ethyl acetate = 3:1 to 1:1) to afford ($R_{S(IV)}$, $S_{C\text{-}1}$, $S_{C\text{-}3}$)-**S29a** as white solid (374 mg, 91% yield, >99:1 d.r.).

^{1}H NMR (400 MHz, $CDCl_3$) δ 7.65 (d, J = 7.8 Hz, 2H), 7.36 – 7.18 (m, 8H), 7.12 – 7.06 (m, 4H), 6.55 (d, J = 8.5 Hz, 1H), 6.03 (d, J = 15.9 Hz, 1H), 5.83 (dd, J = 16.0, 7.7 Hz, 1H), 4.58 – 4.54 (m, 1H), 4.37 (d, J = 5.8 Hz, 1H), 4.00 – 4.93 (m, 1H), 2.31 – 2.27 (m, 2H), 2.22 (s, 3H), 2.12 (s, 1H), 1.22 (s, 9H).

HPLC (Chiralpak OD-H Column), *i*-PrOH/hexane = 10/90, flow rate = 1.0 mL/min, λ = 210 nm; t_R = 19.2 min (major), t_R = 24.0 min (minor).

[α]$_D^{20}$ = –49.8 (c = 0.47, DCM).

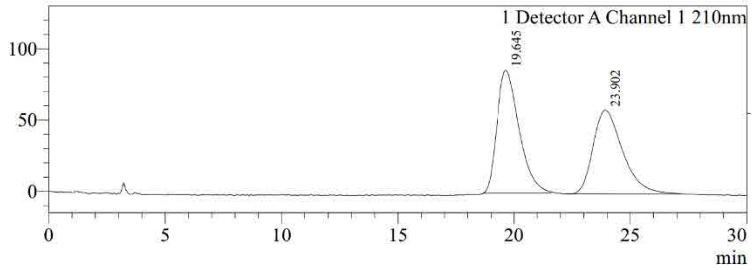

Detector A Channel 1 210nm

Peak#	Ret. Time	USP Width	Area	Height	Area%
1	19.645	1.754	5773457	86332	52.619
2	23.902	2.330	5198649	58928	47.381
Total			10972106	145260	100.000

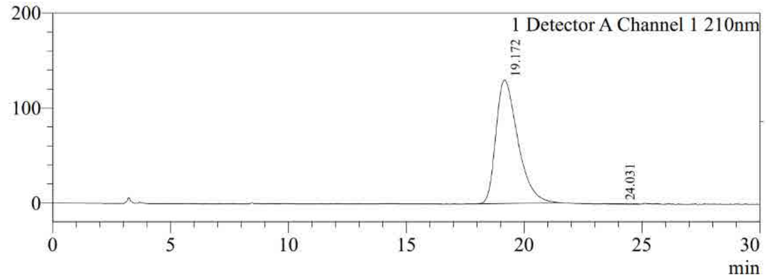

Detector A Channel 1 210nm

Peak#	Ret. Time	USP Width	Area	Height	Area%
1	19.172	1.695	8422194	130192	99.988
2	24.031	0.277	1007	137	0.012
Total			8423201	130329	100.000

***N*-((3*S*,5*S*,*E*)-5-(((*S*)-5-Cyano-1-(1,1-dioxidobenzoisothiazol-2(3*H*)-yl)-1-oxopentan-2-yl)-amino)-1,5-diphenylpent-1-en-3-yl)-4-methylbenzenesulfonamide (29a)**

Prepared according to the **GENERAL PROCEDURE C** with ($R_{S(IV)}$, $S_{C\text{-}1}$, $S_{C\text{-}3}$)-**S29a** (153 mg, 0.30 mmol, >99:1 d.r.) and **2k** (52.0 mg, 0.20 mmol) as substrates. Column chromatography: silica gel, petroleum ether/ethyl acetate/dichloromethane = 1:1:1. White solid (130 mg, 95% yield, >99:1 d.r.).

^{1}H NMR (400 MHz, $CDCl_3$) δ 7.70 (t, J = 8.2 Hz, 4H), 7.55 (t, J = 7.7 Hz, 1H), 7.41 (d, J = 7.8 Hz, 1H), 7.37 – 7.25 (m, 6H), 7.23 – 7.11 (m, 5H), 7.05 (d, J = 7.0 Hz, 2H), 6.16 (d, J = 15.9 Hz, 1H), 6.03 (d, J = 7.9 Hz, 1H), 5.71 (dd, J = 16.3, 7.2 Hz, 1H), 5.03 – 4.77 (m, 2H), 4.10 – 3.97 (m, 1H), 3.90 – 3.72 (m, 2H), 2.51 (s, 1H), 2.27 (s, 3H), 2.22 – 2.09 (m, 2H), 2.05 – 1.96 (m, 1H), 1.95 – 1.83 (m, 3H), 1.82 – 1.71 (m, 1H).

^{13}C NMR (101 MHz, $CDCl_3$) δ 174.8, 143.5, 142.0, 137.6, 136.1, 134.4, 131.9, 129.7, 129.6, 128.7, 128.3, 127.9, 127.7, 127.6, 127.3, 126.4, 125.2, 121.6, 121.3, 119.8, 118.1, 58.3, 58.0, 54.0, 47.5, 43.4, 32.6, 22.0, 21.3, 16.3.

HRMS (ESI, m/z): $[M+H]^+$ Calcd. For $C_{37}H_{39}N_4O_5S_2$: 683.2362; Found: 683.2364.

HPLC (Chiralpak OD-H Column), *i*-PrOH/hexane = 25/75, flow rate = 1.0 mL/min, λ = 210 nm; t_R = 32.6 min (minor), t_R = 38.8 min (major).

$[\alpha]_D^{25}$ = –35.0 (c = 0.5, $CHCl_3$).

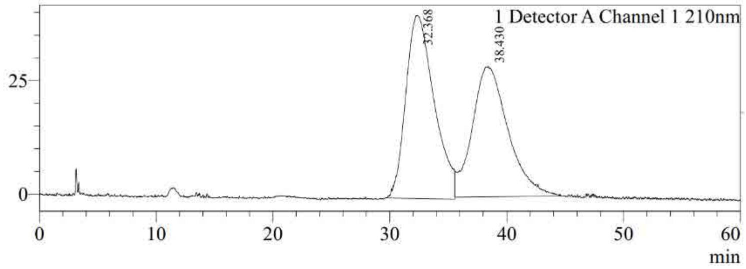

Detector A Channel 1 210nm

Peak#	Ret. Time	USP Width	Area	Height	Area%
1	32.368	4.440	6822440	40305	52.946
2	38.430	5.197	6063212	28701	47.054
Total			12885652	69006	100.000

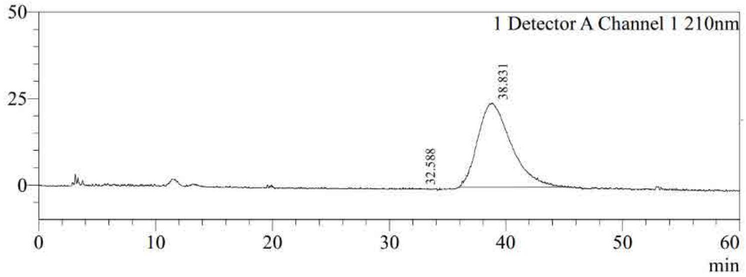

Detector A Channel 1 210nm

Peak#	Ret. Time	USP Width	Area	Height	Area%
1	32.588	0.096	4016	199	0.082
2	38.831	4.703	4898792	24310	99.918
Total			4902808	24508	100.000

Synthesis of ($R_{C\text{-}1}$, $S_{C\text{-}2}$, $S_{C\text{-}3}$)-29b

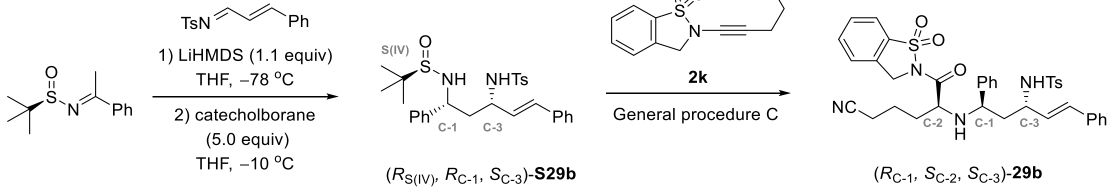

Synthesis of ($R_{S(IV)}$, $R_{C\text{-}1}$, $S_{C\text{-}3}$)-**S29b** (*53*)

Step 1: The imine condensartion procedure is the same as that of **S29a**.

Step 2: Under N_2 atmosphere, to the solution of *β*-amino-sulfinyl imine (650 mg, 1.28 mmol, 1.0 equiv) in THF (25 mL) was added catecholborane (768 mg, 6.4 mmol, 5.0 equiv) at −10 °C. The resulting mixture was stirred at this temperature for 24 h and then quenched by MeOH (10 mL) and a saturated solution of sodium potassium tartrate (10 mL). The mixture was stirred at room temperature and then extracted with dichloromethane. The combined organic layer was washed with brine and concentrated under vacuo. The crude product was purified through flash chromatography (eluent: petroleum ether/ethyl acetate = 3:1 to 1:1) to afford ($R_{S(IV)}$, $R_{C\text{-}1}$, $S_{C\text{-}3}$)-**S29b** as White solid (464 mg, 71% yield, 98:2 d.r.).

^{1}H NMR (400 MHz, $CDCl_3$) δ 7.66 (d, *J* = 7.8 Hz, 2H), 7.35 – 7.28 (m, 5H), 7.23 – 7.15 (m, 3H), 7.08 (d, *J* = 8.0 Hz, 2H), 6.99 (d, *J* = 7.2 Hz, 2H), 6.44 – 6.35 (m, 1H), 6.04 (d, *J* = 15.7 Hz, 1H), 5.65 (dd, *J* = 16.0, 7.8 Hz, 1H), 4.68 – 4.64 (m, 1H), 4.15 – 4.14 (m, 1H), 3.94 – 3.88 (m, 1H), 2.55 – 2.47 (m, 1H), 2.20 (s, 3H), 2.14 – 2.06 (m, 1H), 1.96 – 1.89 (m, 1H), 1.23 (s, 9H).

HPLC (Chiralpak OD-H Column), *i*-PrOH/hexane = 10/90, flow rate = 1.0 mL/min, λ = 210 nm; t_R = 19.5 min (minor), t_R = 23.5 min (major).

$[\alpha]_D^{20}$ = −64.2 (c = 0.47, DCM).

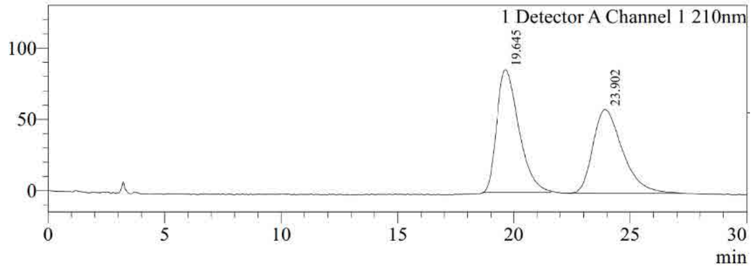

Detector A Channel 1 210nm

Peak#	Ret. Time	USP Width	Area	Height	Area%
1	19.645	1.754	5773457	86332	52.619
2	23.902	2.330	5198649	58928	47.381
Total			10972106	145260	100.000

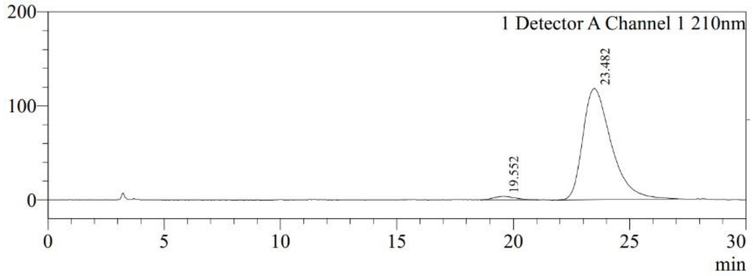

Detector A Channel 1 210nm

Peak#	Ret. Time	USP Width	Area	Height	Area%
1	19.552	1.653	233370	3728	2.267
2	23.482	2.197	10059345	118422	97.733
Total			10292716	122150	100.000

***N*-((3*S*,5*R*,*E*)-5-(((*S*)-5-Cyano-1-(1,1-dioxidobenzoisothiazol-2(3*H*)-yl)-1-oxopentan-2-yl)-amino)-1,5-diphenylpent-1-en-3-yl)-4-methylbenzenesulfonamide (29b)**

Prepared according to the **GENERAL PROCEDURE C** with ($R_{S(IV)}$, $R_{C\text{-}1}$, $S_{C\text{-}3}$)-**S29b** (153 mg, 0.30 mmol, 98:2 d.r.) and **2k** (52.0 mg, 0.20 mmol) as substrates. Column chromatography: silica gel, petroleum ether/ethyl

acetate/dichloromethane = 1:1:1. White solid (117 mg, 86% yield, >99:1 d.r.).

^{1}H NMR (400 MHz, $CDCl_3$) δ 7.83 (d, J = 7.9 Hz, 1H), 7.72 (t, J = 7.6 Hz, 1H), 7.61 (t, J = 7.6 Hz, 1H), 7.56 (d, J = 7.9 Hz, 2H), 7.45 (d, J = 7.8 Hz, 1H), 7.35 – 7.13 (m, 8H), 7.09 (d, J = 7.9 Hz, 2H), 7.00 (d, J = 7.2 Hz, 2H), 6.08 (d, J = 15.8 Hz, 1H), 5.66 (dd, J = 16.0, 7.4 Hz, 1H), 4.83 (d, J = 15.9 Hz, 1H), 4.71 (d, J = 15.7 Hz, 1H), 4.18 (d, J = 8.9 Hz, 1H), 3.99 – 3.89 (m, 1H), 3.82 (t, J = 6.3 Hz, 1H), 2.43 (t, J = 7.0 Hz, 2H), 2.24 (s, 3H), 2.03 (dddd, J = 48.2, 24.4, 13.4, 6.1 Hz, 5H), 1.80 (ddd, J = 14.2, 7.4, 3.7 Hz, 1H), 1.68 (dq, J = 17.5, 5.7, 3.3 Hz, 1H).

^{13}C NMR (101 MHz, $CDCl_3$) δ 173.5, 143.0, 142.7, 138.4, 136.2, 134.5, 133.5, 131.1, 130.8, 129.8, 129.4, 129.1, 128.5, 128.3, 127.7, 127.6, 127.5, 127.2, 126.3, 125.2, 121.7, 119.7, 59.3, 59.1, 54.6, 47.4, 41.6, 33.0, 22.3, 21.3, 17.0.

HRMS (ESI, m/z): $[M+H]^+$ Calcd. For $C_{37}H_{39}N_4O_5S_2$: 683.2362; Found: 683.2365.

HPLC (Chiralpak OD-H Column), *i*-PrOH/hexane = 25/75, flow rate = 1.0 mL/min, λ = 210 nm; t_R = 32.8 min (major), t_R = 38.6 min (minor).

$[\alpha]_D^{25}$ = −77.6 (c = 0.5, $CHCl_3$).

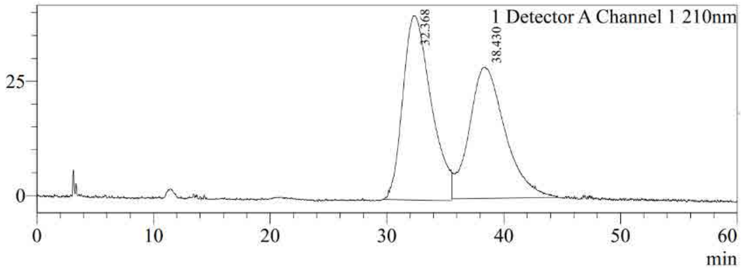

Detector A Channel 1 210nm

Peak#	Ret. Time	USP Width	Area	Height	Area%
1	32.368	4.440	6822440	40305	52.946
2	38.430	5.197	6063212	28701	47.054
Total			12885652	69006	100.000

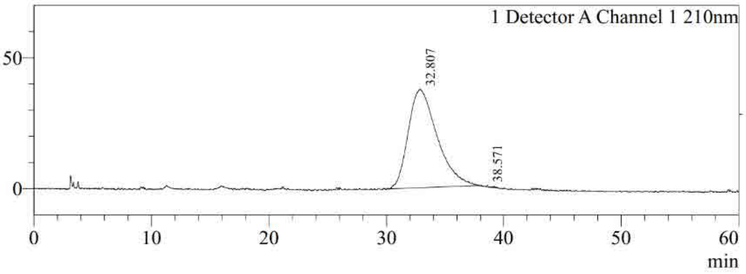

Detector A Channel 1 210nm

Peak#	Ret. Time	USP Width	Area	Height	Area%
1	32.807	4.374	6414725	37600	99.853
2	38.571	0.137	9417	503	0.147
Total			6424141	38103	100.000

Synthesis of (S_{C-1}, S_{C-2}, S_{C-3})-30a

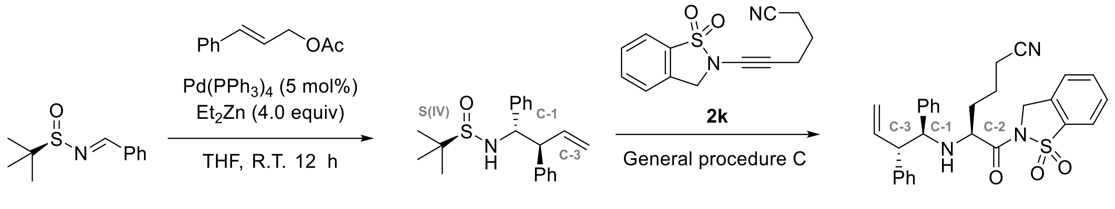

Synthesis of ($R_{S(IV)}$, S_{C-1}, S_{C-3})-**S30a** (*54*)

Under N_2 atmosphere, to the solution of $Pd(PPh_3)_4$ (0.05 mmol, 57.8 mg, 5 mol%) in THF (10 mL) was added (*R*)-sulfinimine (209 mg, 1.0 mmol, 1.0 equiv), cinnamyl acetate (352 mg, 2.0 mmol, 2.0 equiv), followed by addition of Et_2Zn (4.0 mL, 1.0 M in hexane, 4.0 equiv) dropwise. The resulted mixture was stirred at room temperature overnight and then diluted with ethyl acetate. The reaction was quenched with saturated aqueous NH_4Cl and extracted with ethyl acetate. The combined organic layer was concentrated under vacuum. The crude product was purified through flash chromatography (eluent: petroleum ether/ethyl acetate = 20:1:1 to 10:1:1) to afford ($R_{S(IV)}$, S_{C-1}, S_{C-3})-**S30a** as colorless viscous oil (195 mg, 60% yield, >99:1 d.r.).

^{1}H NMR (400 MHz, $CDCl_3$) δ 7.43 – 7.21 (m, 10H), 5.84 (ddd, *J* = 17.0, 10.3, 7.9 Hz, 1H), 4.94 (dt, *J* = 10.4, 1.3 Hz, 1H), 4.78 (dt, *J* = 17.0, 1.4 Hz, 1H), 4.62 (dd, *J* = 9.4, 1.8 Hz, 1H), 3.65 – 3.57 (m, 1H), 3.48 (d, J = 1.7 Hz, 1H), 1.01 (s, 9H).

HPLC (Chiralpak AD-H Column), *i*-PrOH/hexane = 5/95, flow rate = 1.0 mL/min, λ = 254 nm; t_R = 10.3 min (minor), t_R = 13.9 min (major).

$[\alpha]_D^{20}$ = –71.4 (c = 1.56, $CHCl_3$).

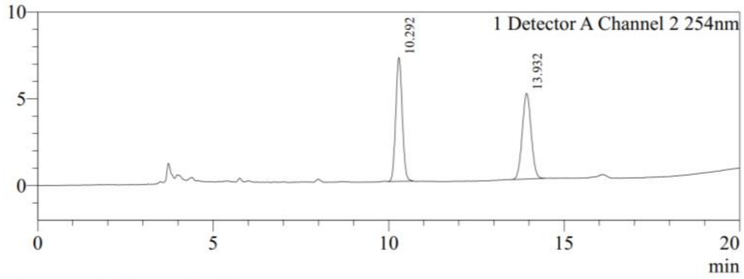

Detector A Channel 2 254nm

Peak#	Ret. Time	USP Width	Area	Height	Area%
1	10.292	0.347	92055	7143	51.411
2	13.932	0.473	87004	4934	48.589
Total			179059	12077	100.000

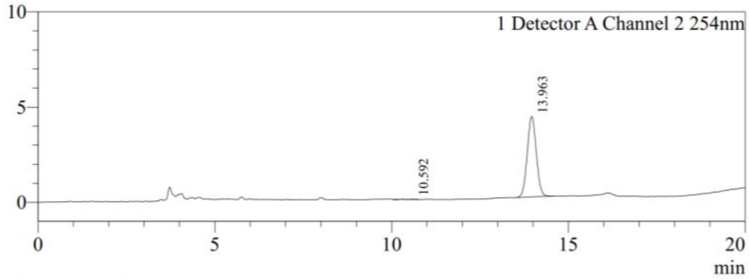

Detector A Channel 2 254nm

Peak#	Ret. Time	USP Width	Area	Height	Area%
1	10.592	0.246	222	28	0.296
2	13.963	0.473	74932	4221	99.704
Total			75154	4249	100.000

(*S*)-6-(1,1-Dioxidobenzoisothiazol-2(3*H*)-yl)-5-(((1*S*,2*S*)-1,2-diphenylbut-3-en-1-yl)amino)-6-oxohexanenitrile (30a)

Prepared according to the **GENERAL PROCEDURE C** with (R_S, $S_{C\text{-}1}$, $S_{C\text{-}3}$)-**S30a** (98.2 mg, 0.30 mmol, >99:1 d.r.) and **2k** (52.0 mg, 0.20 mmol) as substrates. Column chromatography: silica gel, petroleum ether/ethyl acetate = 10:1 to 5:1. Colorless viscous oil (48.4 mg, 48% yield, 99:1 d.r.).

^{1}H NMR (400 MHz, $CDCl_3$) δ 7.75 – 7.65 (m, 2H), 7.57 (t, *J* = 7.6 Hz, 1H), 7.48 – 7.26 (m, 11H), 5.71 (ddd, *J* = 17.5, 10.3, 7.7 Hz, 1H), 5.04 – 4.89 (m, 1H), 4.85 – 4.70 (m, 2H), 4.63 (d, *J* = 17.1 Hz, 1H), 3.83 (d, *J* = 9.8 Hz, 1H), 3.74 – 3.58 (m, 1H), 3.51 (t, *J* = 8.7 Hz, 1H), 2.37 – 2.19 (m, 1H), 2.07 (tq, *J* = 16.9, 8.3, 7.6 Hz, 2H), 1.89 – 1.70 (m, 3H), 1.39 – 1.32 (m, 1H).

^{13}C NMR (101 MHz, $CDCl_3$) δ 174.2, 141.3, 140.7, 139.0, 134.3, 134.2, 130.8, 129.9, 129.1, 128.83, 128.78, 128.3, 127.9, 127.3, 125.1, 121.9, 119.9, 116.4, 65.4, 58.0, 47.4, 32.9, 29.8, 22.2, 16.3.

HRMS (ESI, m/z): $[M+H]^+$ Calcd. For $C_{29}H_{30}N_3O_3S$: 500.2008; Found: 500.2012.

HPLC (Chiralpak AD-H Column), *i*-PrOH/hexane = 30/70, flow rate = 1.0 mL/min, λ = 210 nm; t_R = 6.8 min (major), t_R = 9.5 min (minor).

$[\alpha]_D^{25}$ = –19.6 (c = 0.5, $CHCl_3$).

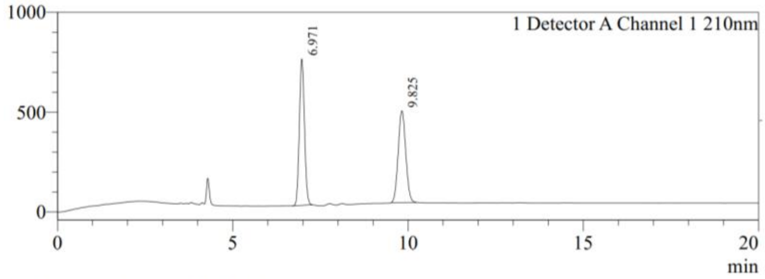

Detector A Channel 1 210nm

Peak#	Ret. Time	USP Width	Area	Height	Area%
1	6.971	0.276	7451737	732974	52.100
2	9.825	0.402	6851002	459072	47.900
Total			14302739	1192046	100.000

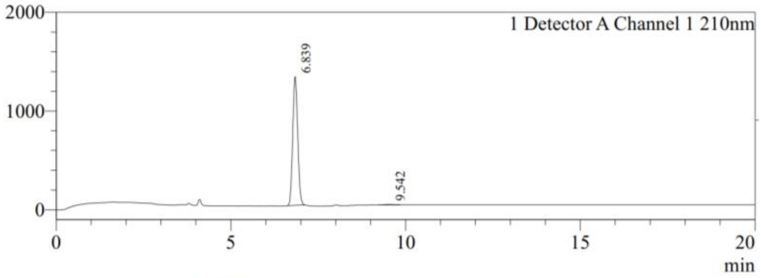

Detector A Channel 1 210nm

Peak#	Ret. Time	USP Width	Area	Height	Area%
1	6.839	0.271	12931404	1301450	99.314
2	9.542	0.397	89309	6139	0.686
Total			13020712	1307589	100.000

Synthesis of (***R***$_{C-1}$, ***R***$_{C-2}$, ***R***$_{C-3}$)-30b

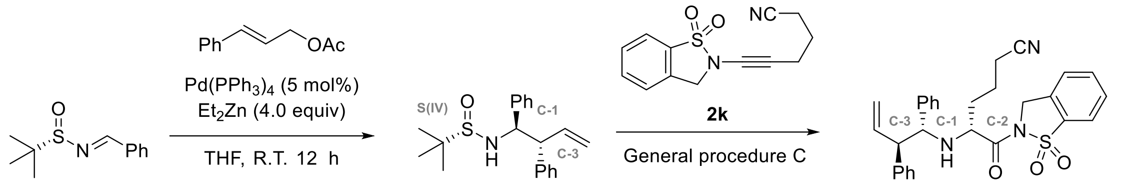

($S_{S(IV)}$, R_{C-1}, R_{C-3})-**S30b** (R_{C-1}, R_{C-2}, R_{C-3})-**30b**

Synthesis of ($S_{S(IV)}$, R_{C-1}, R_{C-3})**-S30b**

Under N_2 atmosphere, to the solution of $Pd(PPh_3)_4$ (0.05 mmol, 57.8 mg, 5 mol%) in THF (10 mL) was added (*S*)-sulfinimine (209 mg, 1.0 mmol, 1.0 equiv), cinnamyl acetate (352 mg, 2.0 mmol, 2.0 equiv), followed by addition of Et_2Zn (4.0 mL, 1.0 M in hexane, 4.0 equiv) dropwise. The resulted mixture was stirred at room temperature overnight and then diluted with ethyl acetate. The reaction was quenched with saturated aqueous NH_4Cl and extracted with ethyl acetate. The combined organic layer was concentrated under vacuum. The crude product was purified through flash chromatography (eluent: petroleum ether/ethyl acetate = 20:1:1 to 10:1:1) to afford ($S_{S(IV)}$, R_{C-1}, R_{C-3})-**S30b** as colorless viscous oil (188 mg, 59% yield, >99:1 d.r.).

^{1}H NMR (400 MHz, $CDCl_3$) δ 7.41 – 7.24 (m, 10H), 5.84 (ddd, *J* = 17.1, 10.4, 7.9 Hz, 1H), 4.94 (dt, *J* = 10.3, 1.3 Hz, 1H), 4.78 (dt, *J* = 17.1, 1.5 Hz, 1H), 4.62 (dd, *J* = 9.5, 1.8 Hz, 1H), 3.65 – 3.56 (m, 1H), 3.51 – 3.44 (m, 2H), 1.01 (s, 9H).

^{13}C NMR (101 MHz, $CDCl_3$) δ 139.8, 139.6, 137.5, 129.2, 129.0, 128.7, 128.3, 128.0, 127.6, 117.7, 61.6, 57.9, 55.4, 22.5.

HRMS (ESI, m/z): $[M+Na]^+$ Calcd. For $C_{20}H_{25}NONaS$: 350.1555; Found: 350.1558.

HPLC (Chiralpak AD-H Column), *i*-PrOH/hexane = 5/95, flow rate = 1.0 mL/min, λ = 254 nm; t_R = 10.3 min (major), t_R = 13.9 min (minor).

[α]$_D^{20}$ = +74.2 (c = 1.56, $CHCl_3$).

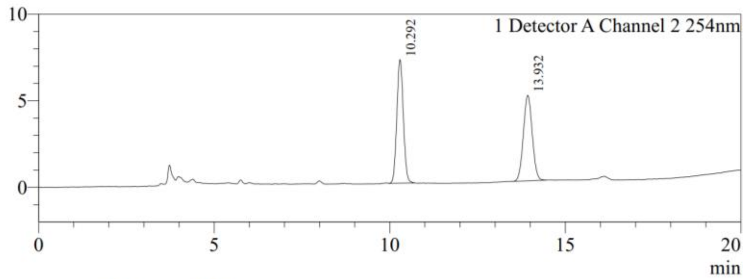

Detector A Channel 2 254nm

Peak#	Ret. Time	USP Width	Area	Height	Area%
1	10.292	0.347	92055	7143	51.411
2	13.932	0.473	87004	4934	48.589
Total			179059	12077	100.000

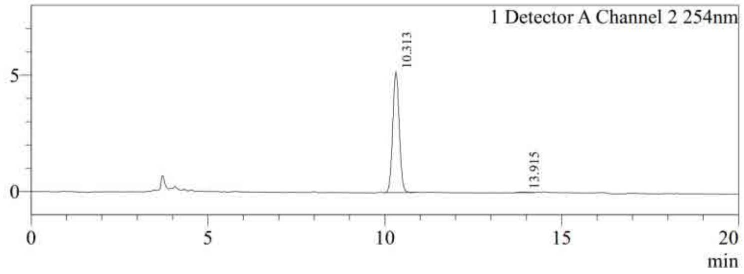

Detector A Channel 2 254nm

Peak#	Ret. Time	USP Width	Area	Height	Area%
1	10.313	0.347	66406	5162	99.635
2	13.915	0.214	243	17	0.365
Total			66650	5179	100.000

(*R*)-6-(1,1-Dioxidobenzoisothiazol-2(3*H*)-yl)-5-(((1*R*,2*R*)-1,2-diphenylbut-3-en-1-yl)amino)-6-oxohexanenitrile (30b)

Prepared according to the **GENERAL PROCEDURE C** with ($S_{S(IV)}$, $R_{C\text{-}1}$, $R_{C\text{-}3}$)-**S30b** (98.2 mg, 0.30 mmol, >99:1 d.r.) and **2k** (52.0 mg, 0.20 mmol) as substrates. Column chromatography: silica gel, petroleum ether/ethyl acetate = 10:1 to 5:1. White solid (45.2 mg, 45% yield, 99:1 d.r.).

^{1}H NMR (400 MHz, $CDCl_3$) δ 7.76 – 7.66 (m, 2H), 7.56 (t, *J* = 7.6 Hz, 1H), 7.49 – 7.27 (m, 11H), 5.71 (ddd, *J* = 17.2, 10.3, 7.7 Hz, 1H), 5.05 – 4.88 (m, 1H), 4.80 – 4.69 (m, 2H), 4.63 (dt, *J* = 17.0, 1.5 Hz, 1H), 3.83 (d, *J* = 9.8 Hz, 1H), 3.72 – 3.57 (m, 1H), 3.51 (t, *J* = 8.7 Hz, 1H), 2.36 – 2.17 (m, 1H), 2.17 – 1.96 (m, 2H), 1.89 – 1.69 (m, 3H), 1.40 – 1.30 (m, 1H).

^{13}C NMR (101 MHz, $CDCl_3$) δ 174.1, 141.3, 140.7, 139.0, 134.3, 134.2, 130.8, 129.8, 129.0, 128.82, 128.78, 128.2, 127.8, 127.3, 125.1, 121.9, 119.9, 116.4, 65.4, 58.0, 47.4, 32.9, 29.8, 22.2, 16.3.

HRMS (ESI, m/z): $[M+H]^+$ Calcd. For $C_{29}H_{30}N_3O_3S$: 500.2008; Found: 500.2013.

HPLC (Chiralpak AD-H Column), *i*-PrOH/hexane = 30/70, flow rate = 1.0 mL/min, λ = 210 nm; t_R = 6.9 min (minor), t_R = 9.6 min (major).

$[\alpha]_D^{25}$ = +19.2 (c = 0.5, $CHCl_3$).

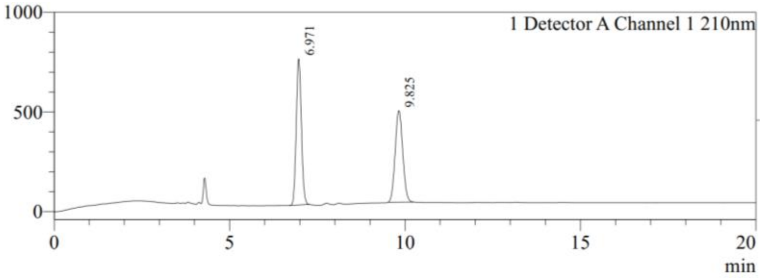

Detector A Channel 1 210nm

Peak#	Ret. Time	USP Width	Area	Height	Area%
1	6.971	0.276	7451737	732974	52.100
2	9.825	0.402	6851002	459072	47.900
Total			14302739	1192046	100.000

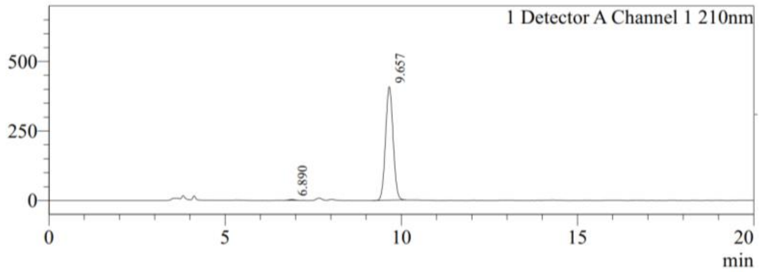

Detector A Channel 1 210nm

Peak#	Ret. Time	USP Width	Area	Height	Area%
1	6.890	0.257	35578	3766	0.579
2	9.657	0.401	6113480	408515	99.421
Total			6149059	412281	100.000

Synthesis of (*S*C-1, *S*C-2)-31a

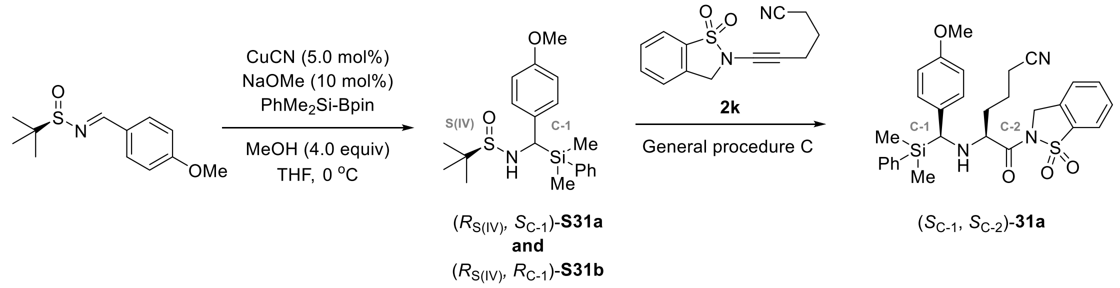

Synthesis of ($R_{S(IV)}$, S_{C-1})-**S31a**

Based on the literature procedure (*79*): Under N_2 atmosphere, the mixture of CuCN (11.3 mg, 0.127 mmol, 5 mol%) and NaOMe (13.7 mg, 0.254 mmol, 10 mol%) in THF (5.0 mL) was stirred at room temperature for 1 h. The resulting mixture was cooled to 0 °C, followed by addition of dimethylphenylsilyl)boronic acid pinacol ester (1.00 g, 3.8 mmol, 1.5 equiv), (*R*)-sulfinimine (608 mg, 2.54 mmol, 1.0 equiv) in THF (5.0 mL) and MeOH (325 mg, 10.2 mmol, 4.0 equiv) seperately. The mixture was stirred continuely at 0 °C until sulfinimine was consumed completely (monitored by TLC), and then quenched by water. After extraction with ethyl acetate, the combined organic layer was washed with brine and concentrated under vacuo. The crude product was purified through flash chromatography (eluent: petroleum ether/ethyl acetate/dichloromethane = 5:1:2, staining using phosphomolybdic acid) to afford ($R_{S(IV)}$, S_{C-1})-**S31a** as colorless oil (260 mg, 27% yield, >99:1 d.r.) and ($R_{S(IV)}$, R_{C-1})-**S31a** as colorless oil (166 mg, 17% yield, >99:1 d.r.). These two compounds were used for the preparation of **31a** and **31b** respectively.

^{1}H NMR (400 MHz, $CDCl_3$) δ 7.49 (dd, *J* = 6.9, 2.4 Hz, 2H), 7.39 (d, *J* = 6.3 Hz, 3H), 7.05 (d, *J* = 8.2 Hz, 2H), 6.81 (d, *J* = 8.3 Hz, 2H), 4.24 (s, 1H), 3.78 (s, 3H), 3.33 (s, 1H), 1.08 (s, 9H), 0.28 (s, 3H), 0.24 (s, 3H).

^{13}C NMR (101 MHz, $CDCl_3$) δ 158.1, 134.9, 134.3, 131.7, 130.1, 128.5, 128.3, 113.7, 55.24, 55.16, 48.2, 22.6, -4.6, -6.1.

HRMS (ESI, m/z): $[M+Na]^+$ Calcd. For $C_{20}H_{29}NO_2NaSSi$: 398.1586; Found: 398.1589.

HPLC (Chiralpak OD-H Column), *i*-PrOH/hexane = 5/95, flow rate = 1.0 mL/min, λ = 210 nm; t_R = 6.5 min (major), t_R = 9.3 min (minor).

$[\alpha]_D^{25}$ = –132.1 (c = 0.47, DCM).

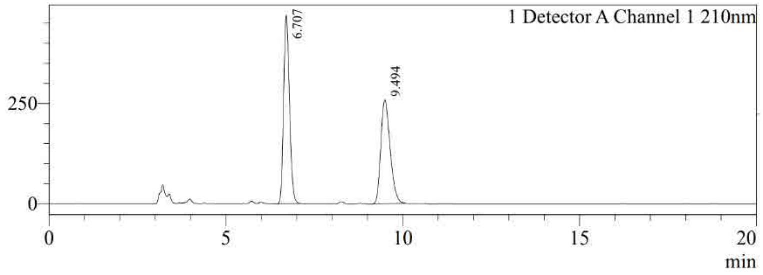

Detector A Channel 1 210nm

Peak#	Ret. Time	USP Width	Area	Height	Area%
1	6.707	0.313	5430985	469039	53.303
2	9.494	0.489	4757947	258894	46.697
Total			10188932	727933	100.000

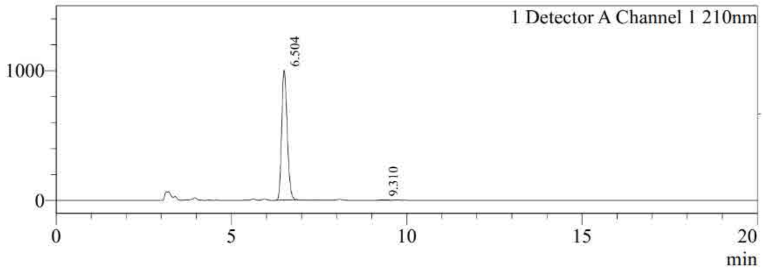

Detector A Channel 1 210nm

Peak#	Ret. Time	USP Width	Area	Height	Area%
1	6.504	0.297	10990507	1001204	99.741
2	9.310	0.321	28584	2432	0.259
Total			11019091	1003635	100.000

(*S*)-5-(((*S*)-(Dimethyl(phenyl)silyl)(4-methoxyphenyl)methyl)amino)-6-(1,1-dioxidobenzol-isothiazol-2(3*H*)-yl)-6-oxohexanenitrile (31a)

Prepared according to the **GENERAL PROCEDURE C** with ($R_{S(IV)}$, $S_{C\text{-}1}$)-**S31a** (113 mg, 0.30 mmol, >99:1 d.r.) and **2k** (52.0 mg, 0.20 mmol) as substrates. Column chromatography: silica gel, petroleum ether/ethyl acetate/dichloromethane = 5:1:1. Colorless oil (66.8 mg, 61% yield, >99:1 d.r.).

^{1}H NMR (400 MHz, $CDCl_3$) δ 7.71 (d, *J* = 8.2 Hz, 1H), 7.67 (d, *J* = 7.6 Hz, 1H), 7.55 (t, *J* = 7.7 Hz, 1H), 7.45 (d, *J* = 6.6 Hz, 3H), 7.41 7.32 (m, 3H), 7.04 (d, *J* = 8.1 Hz, 2H), 6.79 (d, *J* = 8.2 Hz, 2H), 4.93 (d, *J* = 15.4 Hz, 1H), 4.80 (d, *J* = 16.4 Hz, 1H), 3.91 – 3.82 (m, 1H), 3.78 (s, 3H), 3.36 (s, 1H), 2.30 (s, 1H), 2.23 (t, *J* = 7.2 Hz, 2H), 1.98 – 1.76 (m, 3H), 1.51 (tt, *J* = 14.0, 6.6 Hz, 1H), 0.28 (s, 3H), 0.20 (s, 3H).

^{13}C NMR (101 MHz, $CDCl_3$) δ 174.8, 157.8, 136.0, 134.4, 134.2, 133.1, 130.7, 129.8, 129.5, 128.6, 127.8, 125.0, 121.8, 119.9, 113.4, 60.1, 55.2, 53.8, 47.3, 32.7, 22.4, 16.5, -4.6, -6.0.

HRMS (ESI, m/z): $[M+H]^+$ Calcd. For $C_{29}H_{34}N_3O_4SSi$: 548.2039; Found: 548.2042.

HPLC (Chiralpak OD-H Column), *i*-PrOH/hexane = 10/90, flow rate = 1.0 mL/min, λ = 254 nm; t_R = 22.0 min (minor), t_R = 23.7 min (major).

$[\alpha]_D^{25}$ = –45.6 (c = 0.5, $CHCl_3$).

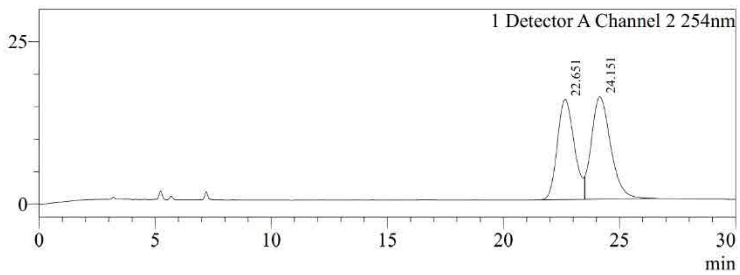

Detector A Channel 2 254nm

Peak#	Ret. Time	USP Width	Area	Height	Area%
1	22.651	1.348	787439	15434	46.410
2	24.151	1.517	909278	15789	53.590
Total			1696717	31223	100.000

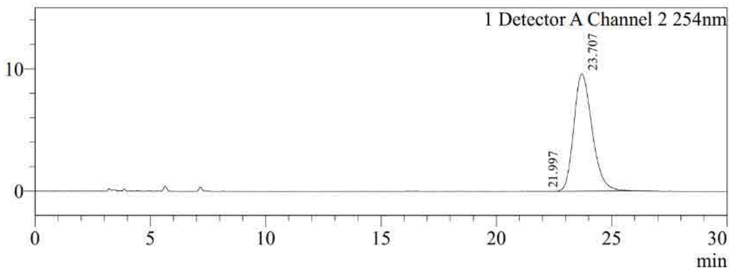

Detector A Channel 2 254nm

Peak#	Ret. Time	USP Width	Area	Height	Area%
1	21.997	0.142	10	7	0.002
2	23.707	1.442	529816	9600	99.998
Total			529825	9607	100.000

Synthesis of ($R_{C\text{-}1}$, $S_{C\text{-}2}$)-31b

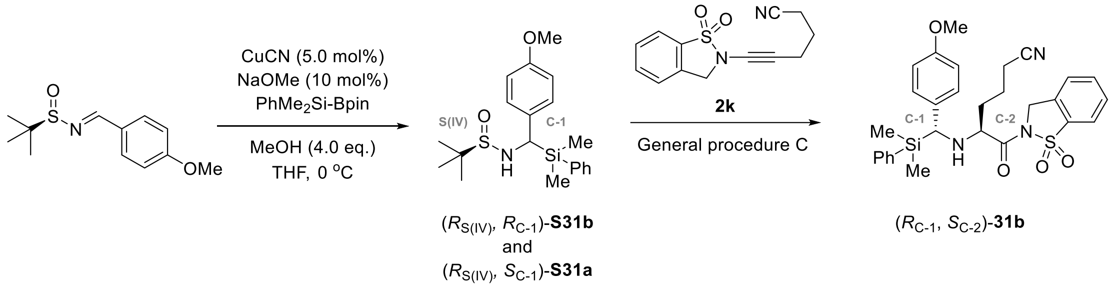

Synthesis of ($R_{S(IV)}$, $R_{C\text{-}1}$)-**S31b** (*55*)

Procedure is the same as **S31a**. Colorless oil (166 mg, 17% yield, >99:1 d.r.).

^{1}H NMR (400 MHz, $CDCl_3$) δ 7.44 – 7.31 (m, 5H), 7.00 – 6.91 (m, 2H), 6.79 (d, *J* = 8.3 Hz, 2H), 4.00 (d, *J* = 8.5 Hz, 1H), 3.77 (s, 3H), 3.40 (d, *J* = 8.5 Hz, 1H), 1.08 (s, 9H), 0.36 (s, 3H), 0.24 (s, 3H).

HPLC (Chiralpak OD-H Column), *i*-PrOH/hexane = 5/95, flow rate = 1.0 mL/min, λ = 210 nm; t_R = 6.3 min (minor), t_R = 9.2 min (major).

$[\alpha]_D^{25}$ = +59.2 (c = 0.47, DCM).

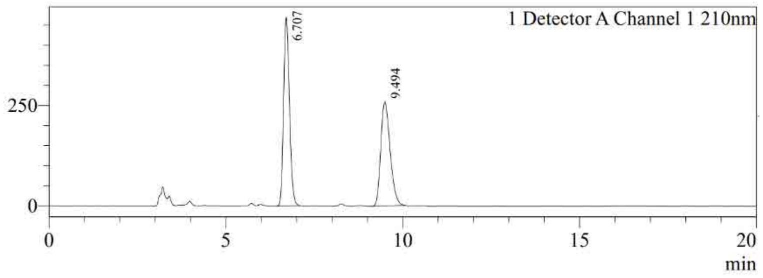

Detector A Channel 1 210nm

Peak#	Ret. Time	USP Width	Area	Height	Area%
1	6.707	0.313	5430985	469039	53.303
2	9.494	0.489	4757947	258894	46.697
Total			10188932	727933	100.000

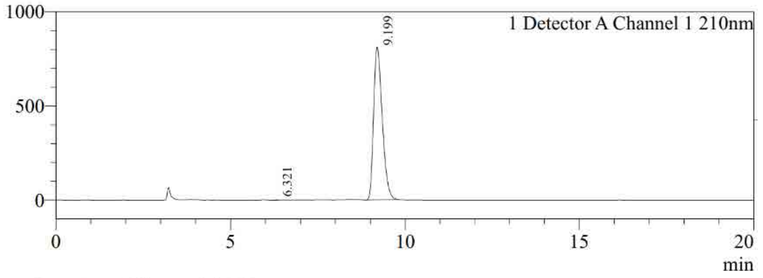

Detector A Channel 1 210nm

Peak#	Ret. Time	USP Width	Area	Height	Area%
1	6.321	0.127	568	119	0.004
2	9.199	0.459	14045079	812044	99.996
Total			14045646	812163	100.000

(*S*)-5-(((*R*)-(Dimethyl(phenyl)silyl)(4-methoxyphenyl)methyl)amino)-6-(1,1-dioxidobenzol-isothiazol-2(3*H*)-yl)-6-oxohexanenitrile (31b)

Prepared according to the **GENERAL PROCEDURE C** with ($R_{S(IV)}$, R_{C-1})-**S31b** (113 mg, 0.30 mmol, >99:1 d.r.) and **2k** (52.0 mg, 0.20 mmol) as substrates. Column chromatography: silica gel, petroleum ether/ethyl acetate/dichloromethane = 5:1:1. Colorless oil (57.1 mg, 52% yield, >99:1 d.r.).

^{1}H NMR (400 MHz, $CDCl_3$) δ 7.76 (d, *J* = 7.9 Hz, 1H), 7.67 (t, *J* = 7.6 Hz, 1H), 7.57 (t, *J* = 7.7 Hz, 1H), 7.49 (dd, *J* = 6.7, 2.9 Hz, 2H), 7.38 – 7.27 (m, 4H), 6.95 (d, *J* = 8.1 Hz, 2H), 6.67 (d, *J* = 8.1 Hz, 2H), 4.61 (d, *J* = 15.9 Hz, 1H), 4.04 (d, *J* = 15.8 Hz, 1H), 3.95 – 3.84 (m, 1H), 3.69 (s, 3H), 3.46 (s, 1H), 2.56 – 2.10 (m, 3H), 1.85 – 1.52 (dtd, *J* = 46.4, 16.7, 12.9, 8.0 Hz, 4H), 0.24 (s, 3H), 0.20 (s, 3H).

^{13}C NMR (101 MHz, $CDCl_3$) δ 173.61, 157.67, 136.64, 134.45, 134.18, 134.10, 130.90, 129.68, 129.40, 128.99, 127.77, 124.97, 121.84, 119.69, 113.15, 62.02, 55.25, 54.25, 46.93, 31.80, 22.04, 16.89, -4.22, -5.89.

HRMS (ESI, m/z): $[M+H]^+$ Calcd. For $C_{29}H_{34}N_3O_4SSi$: 548.2039; Found: 548.2042.

HPLC (Chiralpak OD-H Column), *i*-PrOH/hexane = 10/90, flow rate = 1.0 mL/min, λ = 254 nm; t_R = 22.2 min (major), t_R = 23.6 min (minor).

$[\alpha]_D^{25}$ = +33.5 (c = 0.5, $CHCl_3$).

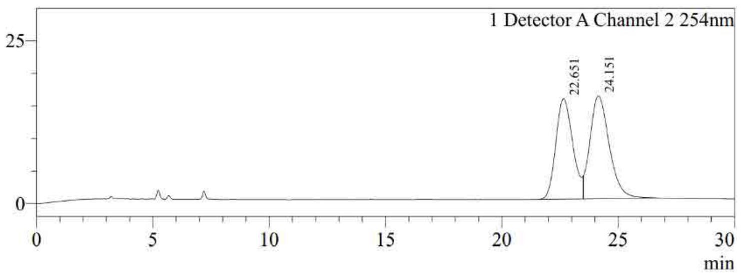

Detector A Channel 2 254nm

Peak#	Ret. Time	USP Width	Area	Height	Area%
1	22.651	1.348	787439	15434	46.410
2	24.151	1.517	909278	15789	53.590
Total			1696717	31223	100.000

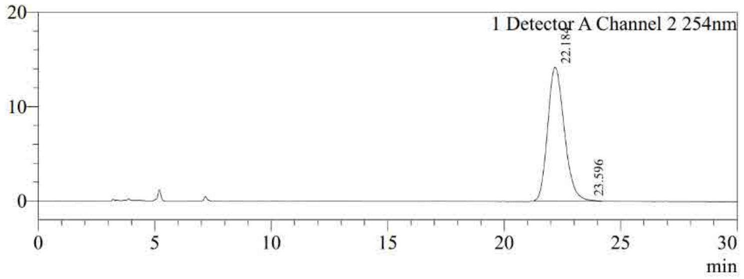

Detector A Channel 2 254nm

Peak#	Ret. Time	USP Width	Area	Height	Area%
1	22.184	1.300	703127	14178	99.610
2	23.596	--	2753	144	0.390
Total			705880	14322	100.000

Synthesis of ($R_{C\text{-}1}$, $S_{C\text{-}2}$)-32a

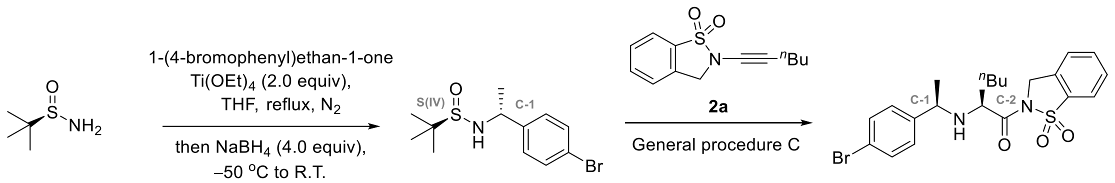

($R_{S(IV)}$, $R_{C\text{-}1}$)-**S32a** ($R_{C\text{-}1}$, $S_{C\text{-}2}$)-**32a**

Synthesis of ($R_{S(IV)}$, $R_{C\text{-}1}$)-S32a

Based on the literature procedure (*57*): Under N_2 atmosphere, the mixture of (*R*)-*tert*-butanesulfinamide (2.42 g, 20 mmol, 1.0 equiv), ketone (3.98 g, 20 mmol, 1.0 equiv) and $Ti(OEt)_4$ (9.12 g, 40 mmol, 2.0 equiv) in THF was refluxed overnight. After completion, the reaction was cooled to –50 °C and $NaBH_4$ (3.03 g, 80 mmol, 4.0 equiv) was added in batches. The reaction mixture was warmed to room temperature and stirred for 2~3 hours (monitored by TLC), followed by quenching with MeOH and saturated brine at 0 °C. The suspension was filtered and washed with ethyl acetate. The filtrate was collected and extracted with ethyl acetate. The combined organic layer was washed with brine and concentrated under vacuo. The crude product was purified through flash chromatography (eluent: petroleum ether/ethyl acetate = 5:1 to 3:1) to afford ($R_{S(IV)}$, $R_{C\text{-}1}$)-**S32a** as white solid (4.33 g, 71% yield, >99:1 d.r.)

¹H NMR (400 MHz, $CDCl_3$) δ 7.45 (d, *J* = 8.4 Hz, 2H), 7.21 (d, *J* = 8.4 Hz, 2H), 4.49 (dp, *J* = 6.8, 3.4 Hz, 1H), 3.45 – 3.34 (m, 1H), 1.47 (d, *J* = 6.6 Hz, 3H), 1.21 (s, 9H).

¹³C NMR (101 MHz, $CDCl_3$) δ 143.1, 131.9, 128.4, 121.7, 55.7, 53.6, 22.8, 22.7.

HRMS (ESI, m/z): $[M+Na]^+$ Calcd. For $C_{12}H_{18}BrNONaS$: 326.0190; Found: 326.0193.

HPLC (Chiralpak OD-H Column), *i*-PrOH/hexane = 3/97, flow rate = 1.0 mL/min, λ = 210 nm; t_R = 11.8 min (minor), t_R = 12.6 min (major).

$[\alpha]_D^{20}$ = –23.4 (c = 1.0, $CHCl_3$).

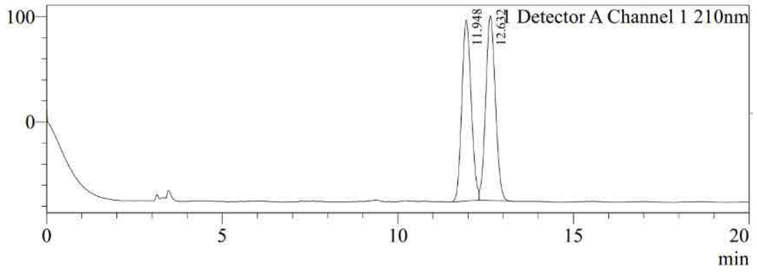

Detector A Channel 1 210nm

Peak#	Ret. Time	USP Width	Area	Height	Area%
1	11.948	0.500	3203155	171793	48.827
2	12.632	0.511	3357111	175571	51.173
Total			6560266	347364	100.000

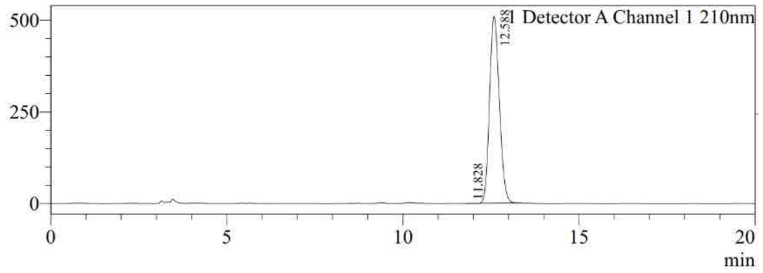

Detector A Channel 1 210nm

Peak#	Ret. Time	USP Width	Area	Height	Area%
1	11.828	0.203	173	124	0.002
2	12.588	0.519	9920095	510460	99.998
Total			9920267	510584	100.000

(*S*)-2-(((*R*)-1-(4-Bromophenyl)ethyl)amino)-1-(1,1-dioxidobenzoisothiazol-2(3*H*)-yl)hexan-1-one (32a)

Prepared according to the **GENERAL PROCEDURE C** with ($R_{S(IV)}$, R_{C-1})-**S32a** (91.3 mg, 0.30 mmol, >99:1 d.r.) and **2a** (50.0 mg, 0.20 mmol) as substrates. Column chromatography: silica gel, petroleum ether/ethyl acetate/dichloromethane = 10:1:1. Colorless viscous oil (80.1 mg, 86% yield, >99:1 d.r.)

¹H NMR (400 MHz, $CDCl_3$) δ 7.81 (d, *J* = 7.9 Hz, 1H), 7.71 (t, *J* = 7.5 Hz, 1H), 7.60 (t, *J* = 7.6 Hz, 1H), 7.46 (d, *J* = 7.8 Hz, 1H), 7.37 (d, *J* = 8.0 Hz, 2H), 7.25 (t, *J* = 6.4 Hz, 2H), 4.82 (d, *J* = 15.9 Hz, 1H), 4.76 (d, *J* = 15.8 Hz, 1H), 4.19 (d, *J* = 7.9 Hz, 1H), 3.77 (q, *J* = 6.5 Hz, 1H), 2.44 – 2.07 (m, 1H), 1.92 – 1.81 (m, 1H), 1.63 – 1.50 (m, 2H), 1.49 – 1.41 (m, 1H), 1.38 (d, *J* = 6.4 Hz, 3H), 1.35 – 1.27 (m, 2H), 0.91 (t, *J* = 7.2 Hz, 3H).

¹³C NMR (101 MHz, $CDCl_3$) δ 174.8, 144.5, 134.5, 134.2, 131.4, 130.9, 129.8, 129.0, 125.1, 121.9, 120.9, 59.8, 56.1, 47.4, 34.0, 28.1, 22.6, 14.1.

HRMS (ESI, m/z): $[M+H]^+$ Calcd. For $C_{21}H_{26}BrN_2O_3S$: 465.0848; Found: 465.0850.

HPLC (Chiralpak AD-H Column), *i*-PrOH/hexane = 10/90, flow rate = 1.0 mL/min, λ = 210 nm; t_R = 12.1 min (minor), t_R = 16.4 min (major).

$[\alpha]_D^{25}$ = +31.5 (c = 0.45, $CHCl_3$).

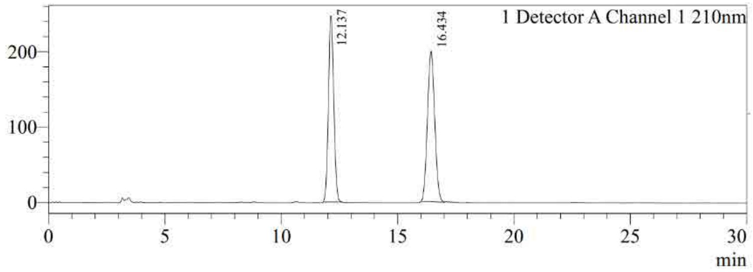

Detector A Channel 1 210nm

Peak#	Ret. Time	USP Width	Area	Height	Area%
1	12.137	0.430	3952477	246894	47.695
2	16.434	0.583	4334427	199867	52.305
Total			8286903	446761	100.000

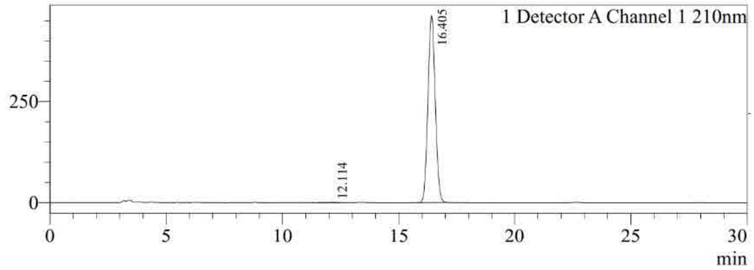

Detector A Channel 1 210nm

Peak#	Ret. Time	USP Width	Area	Height	Area%
1	12.114	0.457	10378	620	0.102
2	16.405	0.583	10119219	463238	99.898
Total			10129597	463857	100.000

Synthesis of ($S_{C\text{-}1}$, $S_{C\text{-}2}$)-32b

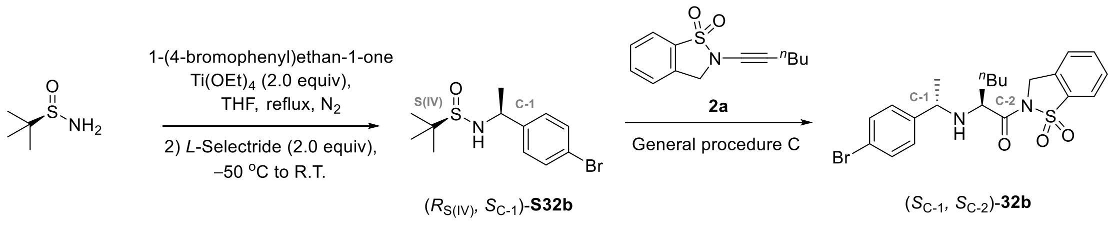

Synthesis of ($R_{S(IV)}$, $S_{C\text{-}1}$)**-S32b** (*67*)

Step1: Under N_2 atmosphere, the reaction mixture of (*R*)-*tert*-butanesulfinamide (1.21 g, 10 mmol, 1.0 equiv), ketone (2.00 g, 10 mmol, 1.0 equiv) and $Ti(OEt)_4$ (4.61 g, 20 mmol, 2.0 equiv) in THF was refluxed overnight. After completion, the mixture was quenched with ice-water and the resulting suspension was filtered and washed with ethyl acetate. The filtrate was collected and extracted with ethyl acetate. The combined organic layer was washed with brine and concentrated under vacuo. The crude product was purified through flash chromatography

(eluent: petroleum ether/ethyl acetate = 10:1) to afford imine as light-yellow oil (2.94 g, 97%), which was used for the next step.

Step2: The solution of imine in THF (25 mL) was cooled to −50 °C and *L*-Selectride (20 mL, 1.0 M in THF, 2.0 equiv) was added dropwise. The resulted mixture was warmed to room temperature and stirred for 2~3 hours (monitored by TLC). After completion, the reaction was quenched with water and saturated aqueous NH_4Cl at 0 °C and extracted with ethyl acetate. The combined organic layer was washed with brine and concentrated under vacuo. The crude product was purified through flash chromatography (eluent: petroleum ether/ethyl acetate = 5:1 to 3:1) to afford ($R_{S(IV)}$, $S_{C\text{-}1}$)-**S32b** as white solid (1.48 g, 48% yield, >99:1 d.r.).

^{1}H NMR (400 MHz, $CDCl_3$) δ 7.44 (d, *J* = 8.3 Hz, 2H), 7.19 (d, *J* = 8.3 Hz, 2H), 4.51 (qd, *J* = 6.6, 3.1 Hz, 1H), 3.34 (d, *J* = 3.4 Hz, 1H), 1.49 (d, *J* = 6.7 Hz, 3H), 1.18 (s, 9H).

HPLC (Chiralpak OD-H Column), *i*-PrOH/hexane = 5/95, flow rate = 1.0 mL/min, λ = 210 nm; t_R = 7.3 min (minor), t_R = 18.0 min (major).

$[\alpha]_D^{20}$ = –92.5 (c = 1.0, $CHCl_3$).

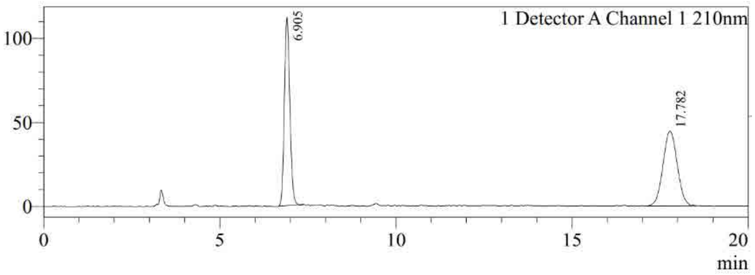

Detector A Channel 1 210nm

Peak#	Ret. Time	USP Width	Area	Height	Area%
1	6.905	0.291	1209341	112100	49.094
2	17.782	0.748	1253999	44531	50.906
Total			2463340	156631	100.000

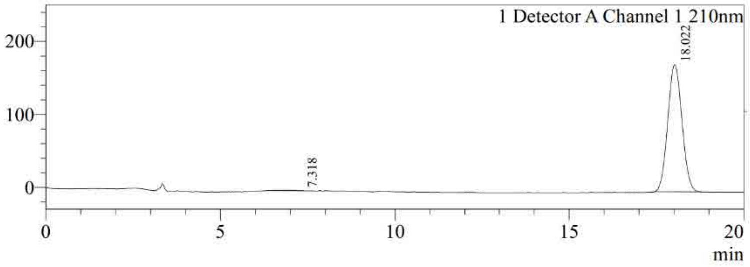

Detector A Channel 1 210nm

Peak#	Ret. Time	USP Width	Area	Height	Area%
1	7.318	0.395	17843	252	0.360
2	18.022	0.761	4942445	174288	99.640
Total			4960289	174540	100.000

(*S*)-2-(((*S*)-1-(4-Bromophenyl)ethyl)amino)-1-(1,1-dioxidobenzoisothiazol-2(3*H*)-yl)hexan-1-one (32b)

Prepared according to the **GENERAL PROCEDURE C** with ($R_{S(IV)}$, $S_{C\text{-}1}$)-**S32b** (91.3 mg, 0.30 mmol, >99:1 d.r.) and **2a** (50.0 mg, 0.20 mmol) as substrates. Column chromatography: silica gel, petroleum ether/ethyl acetate/dichloromethane = 10:1:1. White solid (71.5 mg, 77% yield, >99:1 d.r.)

^{1}H NMR (400 MHz, $CDCl_3$) δ 7.75 (d, *J* = 7.9 Hz, 1H), 7.70 (t, *J* = 7.6 Hz, 1H), 7.57 (t, *J* = 7.7 Hz, 1H), 7.47 (d, *J* = 7.8 Hz, 1H), 7.42 (d, *J* = 8.1 Hz, 2H), 7.29 (d, *J* = 8.1 Hz, 2H), 5.02 (d, *J* = 15.6 Hz, 1H), 4.84 (d, *J* = 15.5 Hz, 1H), 3.80 (s, 1H), 3.70 (q, *J* = 6.6 Hz, 1H), 2.49 – 2.08 (m, 1H), 1.85 – 1.73 (m, 1H), 1.61 – 1.46 (m, 2H), 1.43 – 1.36 (m, 1H), 1.33 (d, *J* = 6.3 Hz, 3H), 1.29 – 1.25 (m, 2H), 0.87 (t, *J* = 7.3 Hz, 3H).

^{13}C NMR (101 MHz, $CDCl_3$) δ 174.5, 143.6, 134.4, 134.2, 131.4, 130.7, 129.9, 129.1, 125.1, 121.9, 120.9, 60.0, 56.6, 47.4, 34.2, 28.2, 25.4, 22.4, 14.0.

HRMS (ESI, m/z): $[M+H]^+$ Calcd. For $C_{21}H_{26}BrN_2O_3S$: 465.0848; Found: 465.0849.

HPLC (Chiralpak AD-H Column), *i*-PrOH/hexane = 10/90, flow rate = 1.0 mL/min, λ = 210 nm; t_R = 10.8 min (major), t_R = 23.3 min (minor).

$[\alpha]_D^{25}$ = –68.0 (c = 0.45, $CHCl_3$).

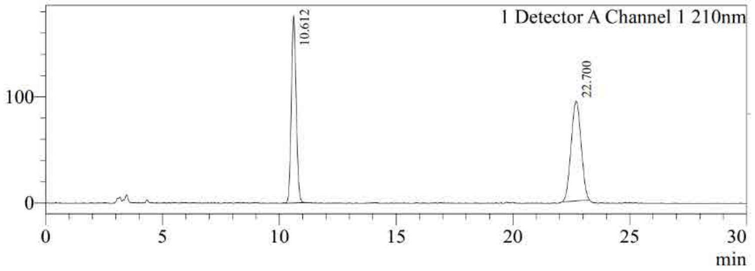

Detector A Channel 1 210nm

Peak#	Ret. Time	USP Width	Area	Height	Area%
1	10.612	0.392	2565587	176042	47.858
2	22.700	0.803	2795235	93990	52.142
Total			5360822	270032	100.000

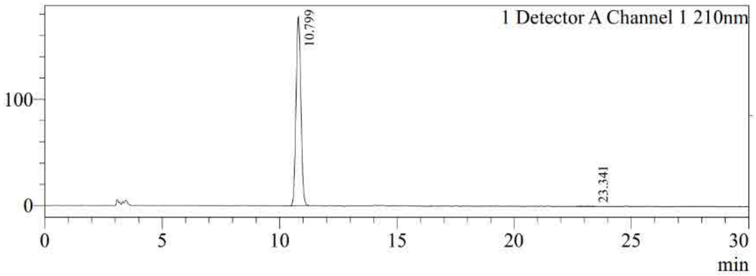

Detector A Channel 1 210nm

Peak#	Ret. Time	USP Width	Area	Height	Area%
1	10.799	0.382	2536802	178267	99.794
2	23.341	0.126	5243	224	0.206
Total			2542045	178491	100.000

Synthesis of (*S*$_{C-1}$, *R*$_{C-2}$)-32c

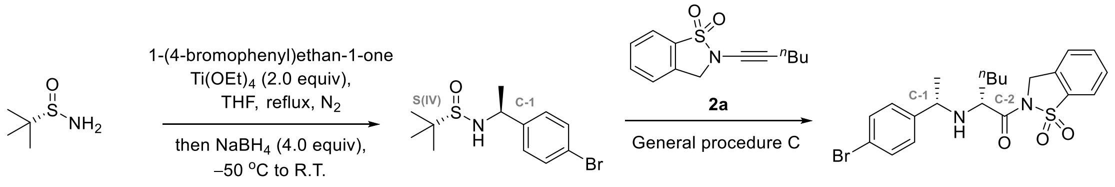

($S_{S(IV)}$, S_{C-1})-**S32c** (S_{C-1}, R_{C-2})-**32c**

Synthesis of ($S_{S(IV)}$, $S_{C\text{-}1}$)-**S32c** (*80*)

Based on the literature procedure (*57*): Under N_2 atmosphere, the mixture of (*S*)-*tert*-butanesulfinamide (2.42 g, 20 mmol, 1.0 equiv), ketone (3.98 g, 20 mmol, 1.0 equiv) and $Ti(OEt)_4$ (9.12 g, 40 mmol, 2.0 equiv) in THF was refluxed overnight. After completion, the reaction was cooled to −50 °C and $NaBH_4$ (3.03 g, 80 mmol, 4.0 equiv) was added in batches. The reaction mixture was warmed to room temperature and stirred for 2~3 hours (monitored by TLC), followed by quenching with MeOH and saturated brine at 0 °C. The suspension was filtered and washed with ethyl acetate. The filtrate was collected and extracted with ethyl acetate. The combined organic layer was washed with brine and concentrated under vacuo. The crude product was purified through flash chromatography (eluent: petroleum ether/ethyl acetate = 5:1 to 3:1) to afford ($S_{S(IV)}$, $S_{C\text{-}1}$)-**S32c** as white solid (3.04 g, 50% yield, >99:1 d.r.).

^{1}H NMR (400 MHz, $CDCl_3$) δ 7.39 (d, *J* = 8.5 Hz, 2H), 7.17 (d, *J* = 8.3 Hz, 2H), 4.43 (qd, *J* = 6.5, 3.2 Hz, 1H), 3.47 (d, *J* = 3.5 Hz, 1H), 1.42 (d, *J* = 6.6 Hz, 3H), 1.16 (s, 9H).

HPLC (Chiralpak OD-H Column), *i*-PrOH/hexane = 5/95, flow rate = 1.0 mL/min, λ = 210 nm; t_R = 6.9 min (major), t_R = 17.7 min (minor).

$[\alpha]_D^{20}$ = +17.4 (c = 1.0, $CHCl_3$).

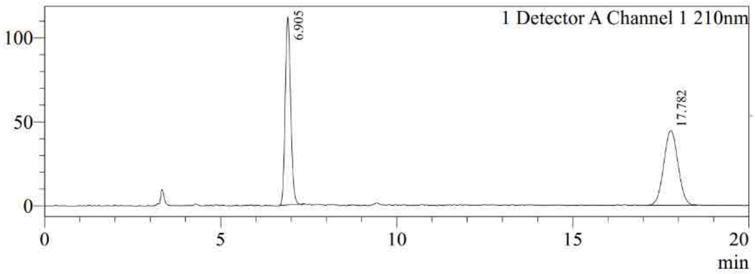

Detector A Channel 1 210nm

Peak#	Ret. Time	USP Width	Area	Height	Area%
1	6.905	0.291	1209341	112100	49.094
2	17.782	0.748	1253999	44531	50.906
Total			2463340	156631	100.000

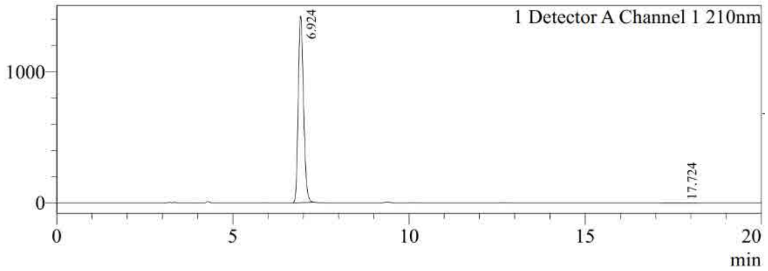

Detector A Channel 1 210nm

Peak#	Ret. Time	USP Width	Area	Height	Area%
1	6.924	0.282	14826187	1423137	99.925
2	17.724	0.617	11105	474	0.075
Total			14837292	1423611	100.000

(*R*)-2-(((*S*)-1-(4-Bromophenyl)ethyl)amino)-1-(1,1-dioxidobenzoisothiazol-2(3*H*)-yl)hexan-1-one (**32c**)

Prepared according to the **GENERAL PROCEDURE C** with ($S_{S(IV)}$, $S_{C\text{-}1}$)-**S32c** (91.3 mg, 0.30 mmol, >99:1 d.r.) and **2a** (50.0 mg, 0.20 mmol) as substrates. Column chromatography: silica gel, petroleum ether/ethyl acetate/dichloromethane = 10:1:1. Colorless viscous oil (82.1 mg, 88% yield, 95:5 d.r.).

^{1}H NMR (400 MHz, $CDCl_3$) δ 7.80 (d, J = 7.9 Hz, 1H), 7.69 (t, J = 7.5 Hz, 1H), 7.58 (t, J = 7.6 Hz, 1H), 7.45 (d, J = 7.8 Hz, 1H), 7.36 (d, J = 8.1 Hz, 2H), 7.22 (d, J = 8.1 Hz, 2H), 4.82 (d, J = 15.9 Hz, 1H), 4.76 (d, J = 15.8 Hz, 1H), 4.17 (s, 1H), 3.75 (q, J = 6.5 Hz, 1H), 2.06 (s, 1H), 1.90 – 1.80 (m, 1H), 1.64 – 1.30 (m, 8H), 0.90 (t, J = 7.3 Hz, 3H).

^{13}C NMR (101 MHz, $CDCl_3$) δ 175.0, 144.8, 134.4, 134.2, 131.3, 130.8, 129.8, 128.8, 125.1, 121.8, 120.7, 59.7, 55.9, 47.3, 34.1, 28.1, 22.6, 22.5, 14.0.

HRMS (ESI, m/z): $[M+H]^+$ Calcd. For $C_{21}H_{26}BrN_2O_3S$: 465.0848; Found: 465.0848.

HPLC (Chiralpak AD-H Column), *i*-PrOH/hexane = 10/90, flow rate = 1.0 mL/min, λ = 210 nm; t_R = 10.7 min (minor), t_R = 22.6 min (major).

$[\alpha]_D^{25}$ = –30.2 (c = 0.45, $CHCl_3$).

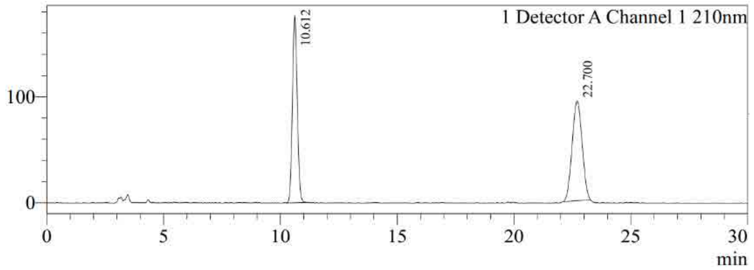

Detector A Channel 1 210nm

Peak#	Ret. Time	USP Width	Area	Height	Area%
1	10.612	0.392	2565587	176042	47.858
2	22.700	0.803	2795235	93990	52.142
Total			5360822	270032	100.000

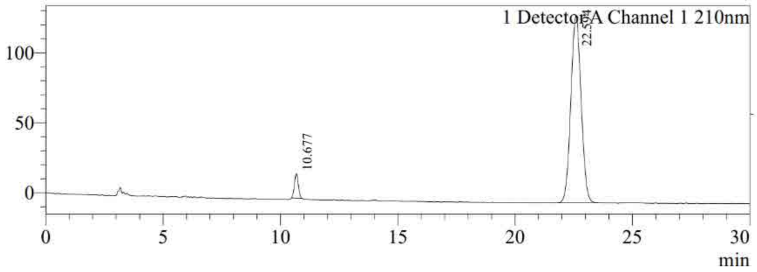

Detector A Channel 1 210nm

Peak#	Ret. Time	USP Width	Area	Height	Area%
1	10.677	0.321	203864	17456	4.784
2	22.594	0.810	4057553	133284	95.216
Total			4261418	150740	100.000

Synthesis of ($R_{C\text{-}1}$, $R_{C\text{-}2}$)-32d

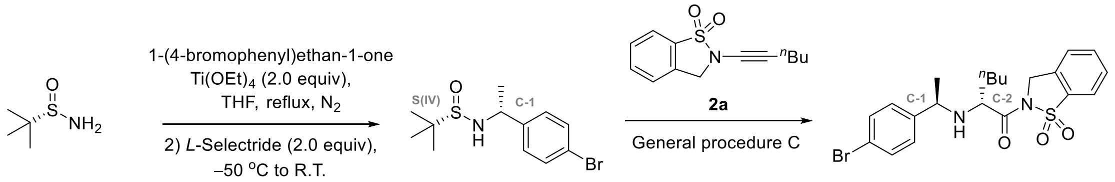

($S_{S(IV)}$, $R_{C\text{-}1}$)-**S32d**

($R_{C\text{-}1}$, $R_{C\text{-}2}$)-**32d**

Synthesis of ($S_{S(IV)}$, R_{C-1})-S32d (*67*)

Step1: Under N_2 atmosphere, the mixture of (*S*)-*tert*-butanesulfinamide (1.21 g, 10 mmol, 1.0 equiv), ketone (2.00 g, 10 mmol, 1.0 equiv) and $Ti(OEt)_4$ (4.61 g, 20 mmol, 2.0 equiv) in THF was refluxed overnight. After completion, the mixture was quenched with ice-water and the resulting suspension was filtered and washed with ethyl acetate. The filtrate was collected and extracted with ethyl acetate. The combined organic layer was washed with brine and concentrated under vacuo. The crude product was purified through flash chromatography (eluent: petroleum ether/ethyl acetate = 10:1) to afford imine as light-yellow oil (2.64 g, 87% yield), which was used for the next step.

Step2: The solution of imine in THF (25 mL) was cooled to −50 °C and *L*-Selectride (20 mL, 1.0 M in THF, 2.0 equiv) was added dropwise. The resulted mixture was warmed to room temperature and stirred for 2~3 hours (monitored by TLC). After completion, the reaction was quenched with water and saturated aqueous NH_4Cl at 0 °C and extracted with ethyl acetate. The combined organic layer was washed with brine and concentrated under vacuo. The crude product was purified through flash chromatography (eluent: petroleum ether/ethyl acetate = 5:1 to 3:1) to afford the ($S_{S(IV)}$, R_{C-1})-**S32d** as white solid (1.41 g, 46% yield, >99:1 d.r.).

^{1}H NMR (400 MHz, $CDCl_3$) δ7.42 (d, J = 8.4 Hz, 2H), 7.17 (d, J = 8.4 Hz, 2H), 4.53 – 4.47 (m, 1H), 3.36 (d, J = 3.2 Hz, 1H), 1.47 (d, J = 6.7 Hz, 3H), 1.16 (s, 9H).

HPLC: Chiralpak OD-H Column, *i*-PrOH/hexane = 3/97, flow rate = 1.0 mL/min, λ = 210 nm; t_R = 11.9 min (major), t_R = 13.0 min (minor).

$[\alpha]_D^{20}$ = +86.8 (c = 1.0, $CHCl_3$).

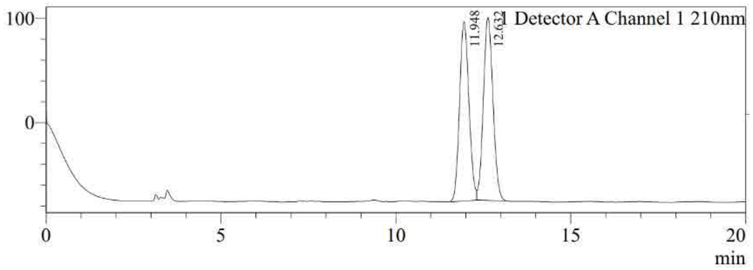

Detector A Channel 1 210nm

Peak#	Ret. Time	USP Width	Area	Height	Area%
1	11.948	0.500	3203155	171793	48.827
2	12.632	0.511	3357111	175571	51.173
Total			6560266	347364	100.000

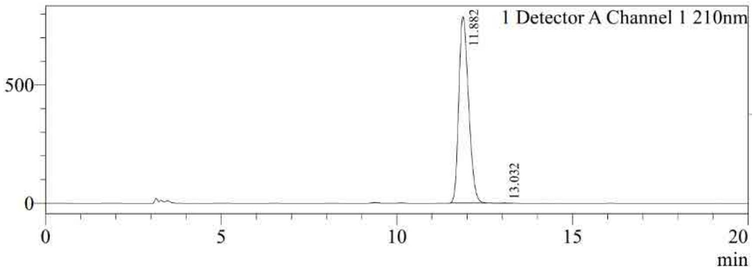

Detector A Channel 1 210nm

Peak#	Ret. Time	USP Width	Area	Height	Area%
1	11.882	0.517	15221429	788874	99.962
2	13.032	0.168	5851	757	0.038
Total			15227280	789632	100.000

(*R*)-2-(((*R*)-1-(4-Bromophenyl)ethyl)amino)-1-(1,1-dioxidobenzoisothiazol-2(3*H*)-yl)hexan-1-one (32d)

Prepared according to the **GENERAL PROCEDURE C** with ($S_{S(IV)}$, R_{C-1})-**S32d** (91.3 mg, 0.30 mmol, >99:1 d.r.) and **2a** (50.0 mg, 0.20 mmol) as substrates. Column chromatography: silica gel, petroleum ether/ethyl acetate/dichloromethane = 10:1:1. White solid (71.1 mg, 76% yield, >99:1 d.r.).

^{1}H NMR (400 MHz, $CDCl_3$) δ 7.75 (d, J = 7.9 Hz, 1H), 7.69 (t, J = 7.6 Hz, 1H), 7.57 (t, J = 7.7 Hz, 1H), 7.46 (d, J = 7.8 Hz, 1H), 7.42 (d, J = 8.1 Hz, 2H), 7.28 (d, J = 8.1 Hz, 2H), 5.01 (d, J = 15.6 Hz, 1H), 4.85 (d, J = 15.6 Hz, 1H), 3.78 (s, 1H), 3.68 (q, J = 6.5 Hz, 1H), 2.26 (s, 1H), 1.88 – 1.69 (m, 1H), 1.60 – 1.45 (m, 2H), 1.43 – 1.34 (m, 1H), 1.31 (d, J = 6.5 Hz, 3H), 1.28 – 1.23 (m, 2H), 0.87 (t, J = 7.3 Hz, 3H).

^{13}C NMR (101 MHz, $CDCl_3$) δ 173.3, 144.1, 134.5, 134.2, 131.4, 130.8, 129.8, 129.1, 125.1, 121.9, 120.8, 60.0, 56.5, 47.4, 34.2, 28.2, 25.4, 22.4, 14.0.

HRMS (ESI, m/z): $[M+H]^+$ Calcd. For $C_{21}H_{26}BrN_2O_3S$: 465.0848; Found: 465.0848.

HPLC (Chiralpak AD-H Column), *i*-PrOH/hexane = 10/90, flow rate = 1.0 mL/min, λ = 210 nm; t_R = 12.1 min (major), t_R = 16.4 min (minor).

$[\alpha]_D^{25}$ = +68.0 (c = 0.45, $CHCl_3$).

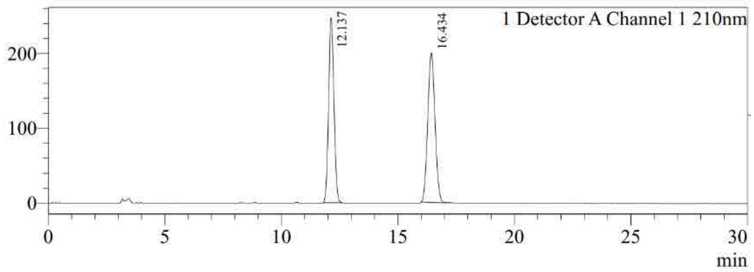

Detector A Channel 1 210nm

Peak#	Ret. Time	USP Width	Area	Height	Area%
1	12.137	0.430	3952477	246894	47.695
2	16.434	0.583	4334427	199867	52.305
Total			8286903	446761	100.000

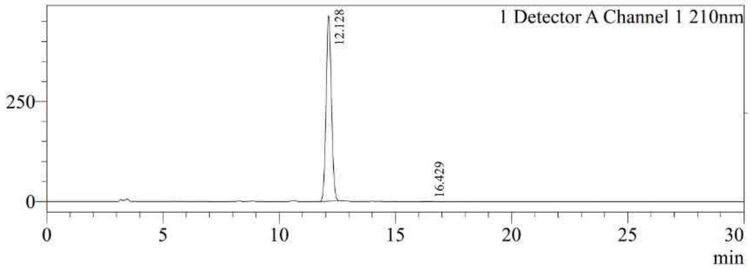

Detector A Channel 1 210nm

Peak#	Ret. Time	USP Width	Area	Height	Area%
1	12.128	0.430	7429731	463564	99.914
2	16.429	0.376	6389	378	0.086
Total			7436120	463942	100.000

Synthesis of ($R_{C\text{-}1}$, $S_{C\text{-}2}$)-33a

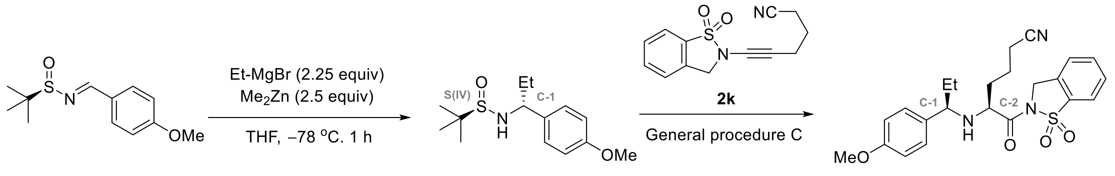

($R_{S(IV)}$, $R_{C\text{-}1}$)-**S33a** ($R_{C\text{-}1}$, $S_{C\text{-}2}$)-**33a**

Synthesis of ($R_{S(IV)}$, $R_{C\text{-}1}$)-**S33a** (*81*)

Under N_2 atmosphere, to the solution of dimethylzinc (12.5 mL, 1.0 M in hexane, 2.5 equiv) was added ethylmagnesium bromide (3.75 mL, 3.0 M in THF, 2.25 equiv). The resulting mixture was stirred at room temperature for 15 minutes and then transfered to the solution of (*R*)-sulfinimine (1.19 g, 5.0 mmol, 1.0 equiv) in THF (0.15 M) dropwise at −78 °C. The reaction was stirred at this temperature for 1 h, and then quenched by saturated aqueous NH_4Cl. After extraction with ethyl acetate, the combined organic layer was washed with brine and concentrated under vacuo. The crude product was purified through flash chromatography (eluent: petroleum ether/ethyl acetate = 4:1 to 2:1) to afford ($R_{S(IV)}$, $R_{C\text{-}1}$)-**S33a** as colorless viscous oil (1.13 g, 83% yield, >99:1 d.r.).

^{1}H NMR (400 MHz, $CDCl_3$) δ 7.20 (d, *J* = 8.5 Hz, 1H), 6.84 (d, *J* = 8.7 Hz, 2H), 4.30 – 4.10 (m, 1H), 3.76 (s, 3H), 3.33 (d, *J* = 3.4 Hz, 1H), 2.01 (tdd, *J* = 14.3, 10.0, 6.4 Hz, 1H), 1.74 – 1.61 (m, 1H), 1.19 (s, 9H), 0.75 (t, *J* = 7.3 Hz, 3H).

HPLC (Chiralpak OD-H Column), *i*-PrOH/hexane = 5/95, flow rate = 1.0 mL/min, λ = 210 nm; t_R = 7.3 min (major), t_R = 8.4 min (minor).

$[\alpha]_D^{20}$ = –47.4 (c = 1.0, $CHCl_3$).

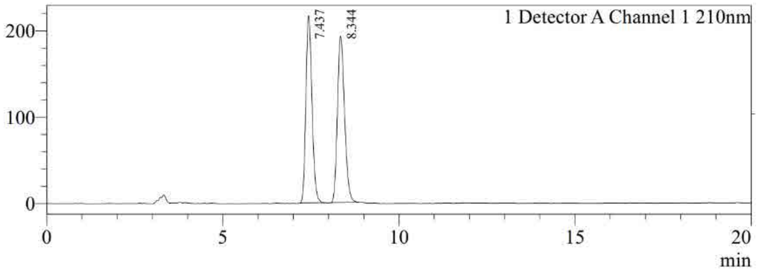

Detector A Channel 1 210nm

Peak#	Ret. Time	USP Width	Area	Height	Area%
1	7.437	0.325	2633858	217059	49.176
2	8.344	0.376	2722082	192712	50.824
Total			5355939	409771	100.000

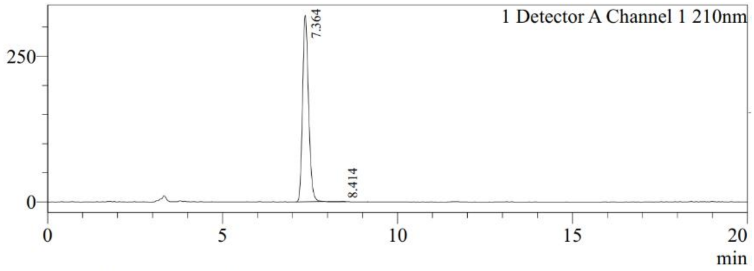

Detector A Channel 1 210nm

Peak#	Ret. Time	USP Width	Area	Height	Area%
1	7.364	0.319	3823923	319917	99.970
2	8.414	0.206	1133	174	0.030
Total			3825056	320091	100.000

(*S*)-6-(1,1-Dioxidobenzoisothiazol-2(3*H*)-yl)-5-(((*R*)-1-(4-methoxyphenyl)propyl)amino)-6-oxohexanenitrile (33a)

Prepared according to the **GENERAL PROCEDURE C** with ($R_{S(IV)}$, R_{C-1})-**S33a** (81.0 mg, 0.30 mmol, >99:1 d.r.) and **2k** (52.0 mg, 0.20 mmol) as substrates. Column chromatography: silica gel, petroleum ether/ethyl acetate/dichloromethane = 5:1:1. Colorless viscous oil (76.1 mg, 86% yield, >99:1 d.r.).

^{1}H NMR (400 MHz, $CDCl_3$) δ 7.81 (d, *J* = 7.9 Hz, 1H), 7.71 (t, *J* = 7.6 Hz, 1H), 7.60 (t, *J* = 7.7 Hz, 1H), 7.44 (d, *J* = 7.9 Hz, 1H), 7.18 (d, *J* = 8.1 Hz, 2H), 6.78 (d, *J* = 8.1 Hz, 2H), 4.79 (d, *J* = 15.9 Hz, 1H), 4.63 (d, *J* = 15.8 Hz, 1H), 4.10 (d, *J* = 9.3 Hz, 1H), 3.72 (s, 3H), 3.42 (dd, *J* = 8.4, 5.3 Hz, 1H), 2.41 (t, *J* = 6.9 Hz, 2H), 1.93 (tdd, *J* = 28.1, 15.7, 7.1 Hz, 5H), 1.61 (dp, *J* = 14.0, 7.4, 7.0 Hz, 2H), 0.75 (t, *J* = 7.4 Hz, 3H).

^{13}C NMR (101 MHz, $CDCl_3$) δ 174.3, 158.6, 135.8, 134.3, 134.1, 130.8, 129.8, 128.7, 125.13, 125.09, 121.9, 119.8, 113.5, 63.0, 59.2, 55.3, 47.2, 33.1, 29.3, 22.4, 16.9, 10.6.

HRMS (ESI, m/z): $[M+H]^+$ Calcd. For $C_{23}H_{28}N_3O_4S$: 442.1801; Found: 442.1803.

HPLC (Chiralpak OD-H Column), *i*-PrOH/hexane = 20/80, flow rate = 1.0 mL/min, λ = 254 nm; t_R = 17.6 min (major), t_R = 23.4 min (minor).

$[\alpha]_D^{25}$ = +13.2 (c = 0.5, $CHCl_3$).

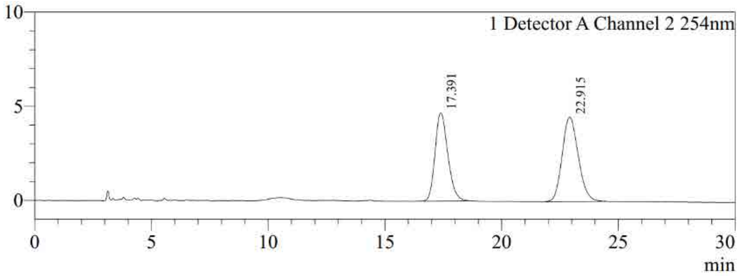

Detector A Channel 2 254nm

Peak#	Ret. Time	USP Width	Area	Height	Area%
1	17.391	0.994	177972	4683	45.035
2	22.915	1.264	217215	4495	54.965
Total			395187	9178	100.000

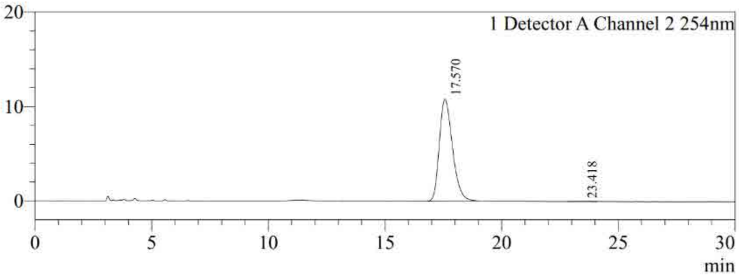

Detector A Channel 2 254nm

Peak#	Ret. Time	USP Width	Area	Height	Area%
1	17.570	1.021	423790	10825	99.876
2	23.418	0.344	528	22	0.124
Total			424318	10847	100.000

Synthesis of (S_{C-1}, S_{C-2})-33b

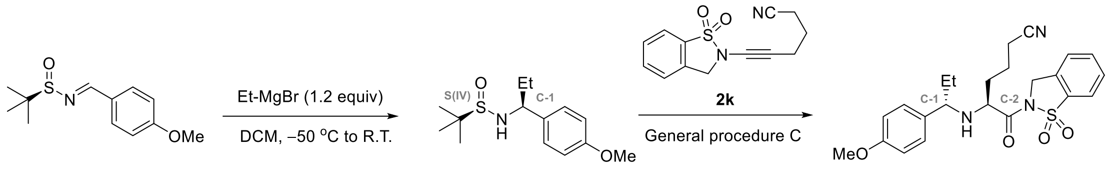

($R_{S(IV)}$, S_{C-1})-**S33b** (S_{C-1}, S_{C-2})-**33b**

Synthesis of ($R_{S(IV)}$, S_{C-1})-**S33b** (*58*)

Under N_2 atmosphere, to the solution of (*R*)-sulfinimine (478 mg, 2.0 mmol, 1.0 equiv) in dichoromethane (0.15 M) was added ethylmagnesium bromide (0.8 mL, 3.0 M in THF, 1.2 equiv) dropwise at −50 °C. The reaction was stirred at this temperature for 5 h and then warmed to room temperature and stirred overnight. Once the sulfinimine was consumed completely, the reaction was quenched by saturated aqueous NH_4Cl and extracted with dichoromethane. The combined organic layer was washed with brine and concentrated under vacuo. The crude product was purified through flash chromatography (eluent: petroleum ether/ethyl acetate = 4:1 to 2:1) to afford ($R_{S(IV)}$, S_{C-1})-**S33b** as white solid (302 mg, 56% yield, >99:1 d.r.).

^{1}H NMR (400 MHz, $CDCl_3$) δ 7.18 (d, *J* = 8.2 Hz, 2H), 6.84 (d, *J* = 8.2 Hz, 2H), 4.21 (ddd, *J* = 8.3, 5.8, 2.4 Hz, 1H), 3.77 (s, 3H), 3.37 (d, *J* = 2.8 Hz, 1H), 1.88 – 1.65 (m, 2H), 1.15 (s, 9H), 0.80 (t, *J* = 7.4 Hz, 3H).

HPLC (Chiralpak OD-H Column), *i*-PrOH/hexane = 5/95, flow rate = 1.0 mL/min, λ = 210 nm; t_R = 7.6 min (major), t_R = 8.3 min (minor).

$[\alpha]_D^{20}$ = +101.8 (c = 1.0, $CHCl_3$).

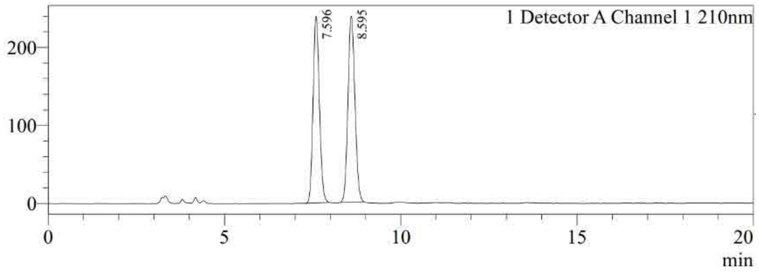

Detector A Channel 1 210nm

Peak#	Ret. Time	USP Width	Area	Height	Area%
1	7.596	0.334	2970731	238912	47.655
2	8.595	0.369	3263095	238247	52.345
Total			6233826	477159	100.000

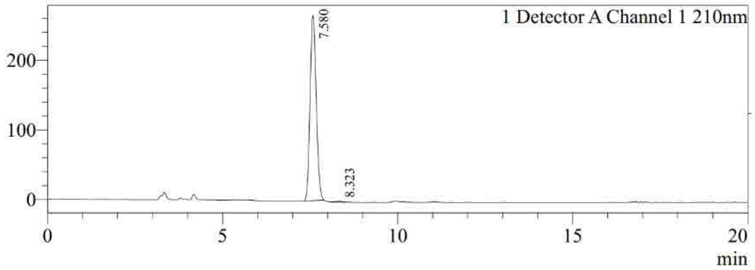

Detector A Channel 1 210nm

Peak#	Ret. Time	USP Width	Area	Height	Area%
1	7.580	0.332	3257955	265548	99.642
2	8.323	0.251	11689	1391	0.358
Total			3269645	266939	100.000

(*S*)-6-(1,1-Dioxidobenzoisothiazol-2(3*H*)-yl)-5-(((*S*)-1-(4-methoxyphenyl)propyl)amino)-6-oxohexanenitrile (33b)

Prepared according to the **GENERAL PROCEDURE C** with ($R_{S(IV)}$, $S_{C\text{-}1}$)-**S33b** (81.0 mg, 0.30 mmol, >99:1 d.r.) and **2k** (52.0 mg, 0.20 mmol) as substrates. Column chromatography: silica gel, petroleum ether/ethyl acetate/dichloromethane = 5:1:1. Colorless viscous oil (70.1 mg, 79% yield, 99:1 d.r.).

^{1}H NMR (400 MHz, $CDCl_3$) δ 7.75 (d, *J* = 7.8 Hz, 1H), 7.70 (t, *J* = 7.4 Hz, 1H), 7.57 (t, *J* = 7.6 Hz, 1H), 7.47 (d, *J* = 7.8 Hz, 1H), 7.25 (dd, *J* = 9.3, 2.4 Hz, 2H), 6.90 – 6.82 (m, 2H), 5.02 (d, *J* = 16.3 Hz, 1H), 4.86 (d, *J* = 16.1 Hz, 1H), 3.79 (s, 3H), 3.76 – 3.66 (m, 1H), 3.31 (t, *J* = 7.0 Hz, 1H), 2.32 (s, 1H), 2.22 – 2.05 (m, 2H), 1.95 – 1.77 (m, 3H), 1.74 – 1.46 (m, 3H), 0.78 (t, *J* = 7.4 Hz, 3H).

^{13}C NMR (101 MHz, $CDCl_3$) δ 175.3, 158.8, 135.3, 134.3, 134.2, 129.8, 129.0, 125.1, 121.8, 119.9, 113.6, 63.1, 58.4, 55.2, 47.4, 33.1, 31.7, 22.3, 16.3, 10.8.

HRMS (ESI, m/z): $[M+H]^+$ Calcd. For $C_{23}H_{28}N_3O_4S$: 442.1801; Found: 442.1803.

HPLC (Chiralpak OD-H Column), *i*-PrOH/hexane = 20/80, flow rate = 1.0 mL/min, λ = 210 nm; t_R = 14.5 min (minor), t_R = 15.7 min (major).

$[\alpha]_D^{25}$ = +46.8 (c = 0.5, $CHCl_3$).

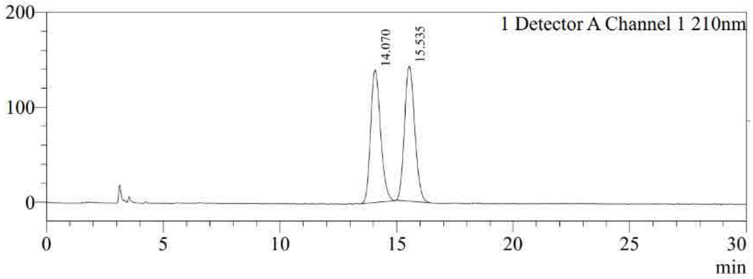

Detector A Channel 1 210nm

Peak#	Ret. Time	USP Width	Area	Height	Area%
1	14.070	0.783	4135927	139651	48.747
2	15.535	0.816	4348508	142276	51.253
Total			8484435	281927	100.000

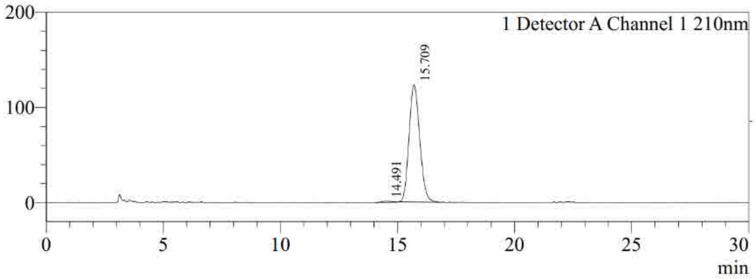

Detector A Channel 1 210nm

Peak#	Ret. Time	USP Width	Area	Height	Area%
1	14.491	0.472	36251	1339	0.915
2	15.709	0.841	3924012	123236	99.085
Total			3960263	124574	100.000

Synthesis of ($S_{C\text{-}1}$, $R_{C\text{-}2}$)-33c

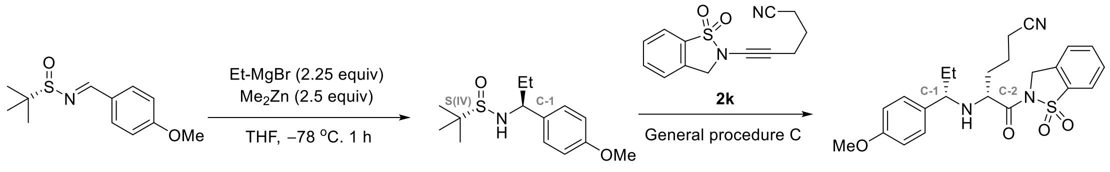

($S_{S(IV)}$, $S_{C\text{-}1}$)-**S33c** ($S_{C\text{-}1}$, $R_{C\text{-}2}$)-**33c**

Synthesis of ($S_{S(IV)}$, $S_{C\text{-}1}$)-**S33c**

Under N_2 atmosphere, to the solution of dimethylzinc (12.5 mL, 1.0 M in hexane, 2.5 equiv) was added ethylmagnesium bromide (3.75 mL, 3.0 M in THF, 2.25 equiv). The resulting mixture was stirred at room temperature for 15 minutes and then transfered to the solution of (*S*)-sulfinimine (1.19 g, 5.0 mmol, 1.0 equiv) in THF (0.15 M) dropwise at −78 °C. The reaction was stirred at this temperature for 1 h and then quenched by saturated aqueous NH_4Cl. After extraction with ethyl acetate, the combined organic layer was washed with brine and concentrated under vacuo. The crude product was purified through flash chromatography (eluent: petroleum ether/ethyl acetate = 4:1 to 2:1) to afford ($S_{S(IV)}$, $S_{C\text{-}1}$)-**S33c** as colorless oil (1.06 g, 79% yield, >99:1 d.r.).

^{1}H NMR (400 MHz, $CDCl_3$) δ 7.21 (d, *J* = 8.7 Hz, 2H), 6.85 (d, *J* = 8.8 Hz, 2H), 4.20 (ddd, *J* = 8.7, 5.3, 3.1 Hz, 1H), 3.77 (s, 3H), 3.32 (d, *J* = 3.3 Hz, 1H), 2.02 (ddd, *J* = 15.2, 7.4, 3.8 Hz, 1H), 1.75 – 1.61 (m, 1H), 1.20 (s, 9H), 0.76 (t, *J* = 7.4 Hz, 3H).

^{13}C NMR (101 MHz, $CDCl_3$) δ 159.1, 134.4, 128.5, 114.0, 59.9, 55.6, 55.3, 29.3, 22.7, 10.2.

HRMS (ESI, m/z): $[M+Na]^+$ Calcd. For $C_{14}H_{23}NO_2NaS$: 292.1347; Found: 292.1350.

HPLC (Chiralpak OD-H Column), *i*-PrOH/hexane = 5/95, flow rate = 1.0 mL/min, λ = 210 nm; t_R = 7.5 min (minor), t_R = 8.3 min (major).

$[\alpha]_D^{20}$ = +49.0 (c = 1.0, $CHCl_3$).

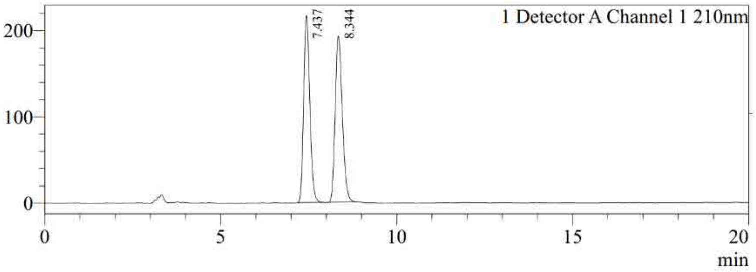

Detector A Channel 1 210nm

Peak#	Ret. Time	USP Width	Area	Height	Area%
1	7.437	0.325	2633858	217059	49.176
2	8.344	0.376	2722082	192712	50.824
Total			5355939	409771	100.000

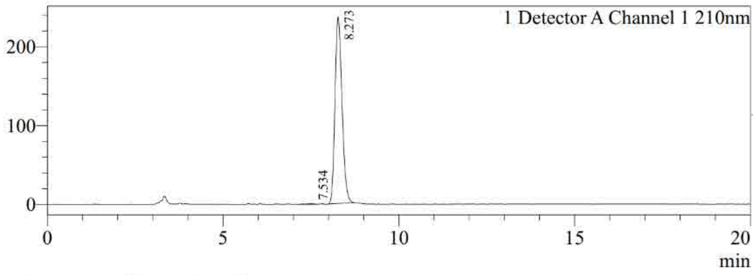

Detector A Channel 1 210nm

Peak#	Ret. Time	USP Width	Area	Height	Area%
1	7.534	0.409	11965	759	0.363
2	8.273	0.370	3288185	237015	99.637
Total			3300150	237774	100.000

(*R*)-6-(1,1-Dioxidobenzoisothiazol-2(3*H*)-yl)-5-(((*S*)-1-(4-methoxyphenyl)propyl)amino)-6-oxohexanenitrile (33c)

Prepared according to the **GENERAL PROCEDURE C** with ($S_{S(IV)}$, $S_{C\text{-}1}$)-**S33c** (81.0 mg, 0.30 mmol, >99:1 d.r.) and **2k** (52.0 mg, 0.20 mmol) as substrates. Column chromatography: silica gel, petroleum ether/ethyl acetate/dichloromethane = 5:1:1. Colorless viscous oil (73.1 mg, 82% yield, >99:1 d.r.).

^{1}H NMR (400 MHz, $CDCl_3$) δ 7.81 (d, *J* = 7.9 Hz, 1H), 7.71 (t, *J* = 7.7 Hz, 1H), 7.60 (t, *J* = 7.7 Hz, 1H), 7.44 (d, *J* = 7.8 Hz, 1H), 7.18 (d, *J* = 8.2 Hz, 2H), 6.78 (d, *J* = 8.1 Hz, 2H), 4.79 (d, *J* = 15.9 Hz, 1H), 4.63 (d, *J* = 15.8 Hz, 1H), 4.10 (d, *J* = 8.9 Hz, 1H), 3.73 (s, 3H), 3.42 (dd, *J* = 8.4, 5.2 Hz, 1H), 2.42 (dt, *J* = 7.7, 4.0 Hz, 2H), 2.15 – 1.76 (m, 5H), 1.68 – 1.52 (m, 2H), 0.75 (t, *J* = 7.4 Hz, 3H).

^{13}C NMR (101 MHz, $CDCl_3$) δ 174.3, 158.6, 135.8, 134.3, 134.1, 130.8, 129.8, 128.7, 125.1, 121.91, 121.86, 119.8, 113.5, 63.0, 59.2, 55.3, 47.2, 33.1, 29.3, 22.4, 17.0, 10.6.

HRMS (ESI, m/z): $[M+H]^+$ Calcd. For $C_{23}H_{28}N_3O_4S$: 442.1801; Found: 442.1801.

HPLC (Chiralpak OD-H Column), *i*-PrOH/hexane = 20/80, flow rate = 1.0 mL/min, λ = 254 nm; t_R = 17.5 min (minor), t_R = 23.2 min (major).

$[\alpha]_D^{25}$ = −16.0 (c = 0.5, $CHCl_3$).

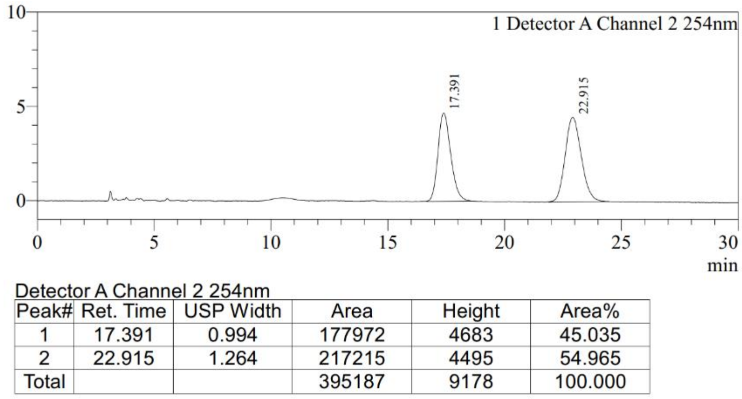

Detector A Channel 2 254nm

Peak#	Ret. Time	USP Width	Area	Height	Area%
1	17.391	0.994	177972	4683	45.035
2	22.915	1.264	217215	4495	54.965
Total			395187	9178	100.000

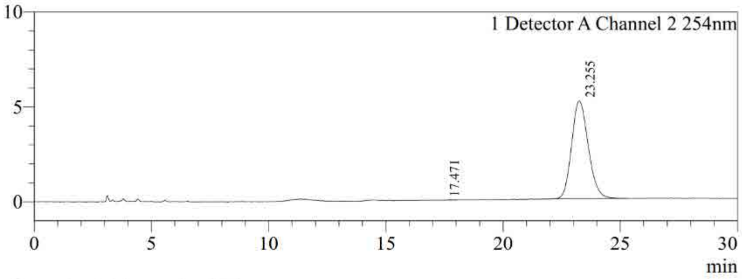

Detector A Channel 2 254nm

Peak#	Ret. Time	USP Width	Area	Height	Area%
1	17.471	0.595	385	1	0.151
2	23.255	1.286	254444	5145	99.849
Total			254829	5145	100.000

Synthesis of (*R*$_{C-1}$, *R*$_{C-2}$)-33d

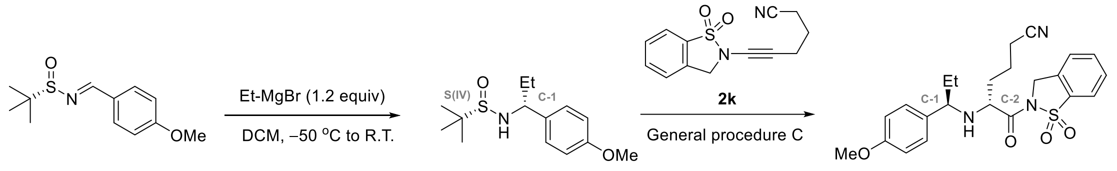

($S_{S(IV)}$, R_{C-1})-**S33d** (R_{C-1}, R_{C-2})-**33d**

Synthesis of ($S_{S(IV)}$, R_{C-1})-**S33d**

Under N_2 atmosphere, to the solution of (*S*)-sulfinimine (478 mg, 2.0 mmol, 1.0 equiv) in dichoromethane (0.15 M) was added ethylmagnesium bromide (0.8 mL, 3.0 M in THF, 1.2 equiv) dropwise at −50 °C. The reaction was stirred at this temperature for 5 h and then warmed to room temperature and stirred overnight. Once the sulfinimine was consumed completely, the reaction was quenched by saturated aqueous NH_4Cl and extracted with dichoromethane. The combined organic layer was washed with brine and concentrated under vacuo. The crude product was purified through flash chromatography (eluent: petroleum ether/ethyl acetate = 4:1 to 2:1) to afford ($S_{S(IV)}$, R_{C-1})-**S33d** as white solid (297 mg, 55% yield, >99:1 d.r.).

^{1}H NMR (400 MHz, $CDCl_3$) δ 7.19 (d, *J* = 8.8 Hz, 2H), 6.86 (d, *J* = 8.8 Hz, 2H), 4.23 (t, *J* = 7.0 Hz, 1H), 3.79 (s, 3H), 3.37 (s, 1H), 1.87 – 1.69 (m, 2H), 1.17 (s, 9H), 0.81 (t, *J* = 7.4 Hz, 3H).

^{13}C NMR (101 MHz, $CDCl_3$) δ 159.1, 133.7, 129.0, 113.8, 60.1, 55.4, 55.3, 31.9, 22.7, 10.6.

HRMS (ESI, m/z): $[M+Na]^+$ Calcd. For $C_{14}H_{23}NO_2NaS$: 292.1347; Found: 292.1347.

HPLC (Chiralpak OD-H Column), *i*-PrOH/hexane = 5/95, flow rate = 1.0 mL/min, λ = 210 nm; t_R = 7.5 min (minor), t_R = 8.5 min (major).

$[\alpha]_D^{20}$ = −94.2 (c = 1.0, $CHCl_3$).

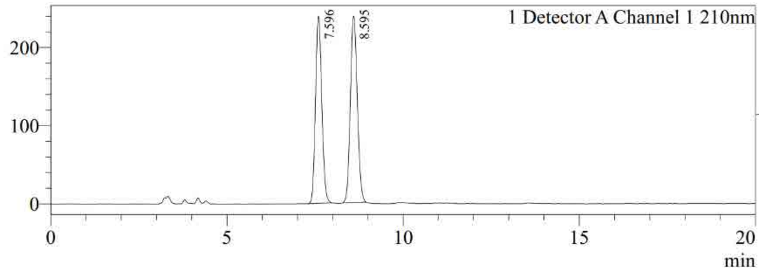

Detector A Channel 1 210nm

Peak#	Ret. Time	USP Width	Area	Height	Area%
1	7.596	0.334	2970731	238912	47.655
2	8.595	0.369	3263095	238247	52.345
Total			6233826	477159	100.000

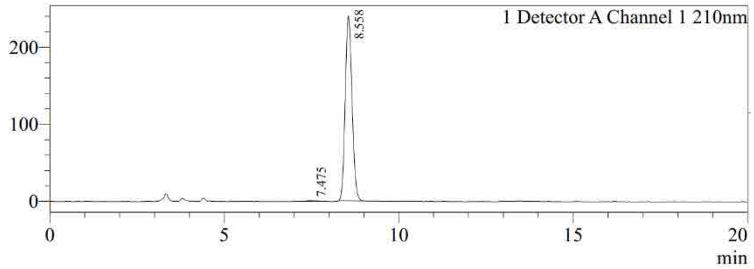

Detector A Channel 1 210nm

Peak#	Ret. Time	USP Width	Area	Height	Area%
1	7.475	0.321	12461	1057	0.381
2	8.558	0.366	3261215	239690	99.619
Total			3273676	240748	100.000

(*R*)-6-(1,1-Dioxidobenzoisothiazol-2(3*H*)-yl)-5-(((*R*)-1-(4-methoxyphenyl)propyl)amino)-6-oxohexanenitrile (33d)

Prepared according to the **GENERAL PROCEDURE C** with ($S_{S(IV)}$, $R_{C\text{-}1}$)-**S33d** (81.0 mg, 0.30 mmol, >99:1 d.r.) and **2k** (52.0 mg, 0.20 mmol) as substrates. Column chromatography: silica gel, petroleum ether/ethyl acetate/dichloromethane = 5:1:1. Colorless viscous oil (82.2 mg, 93% yield, >99:1 d.r.).

^{1}H NMR (400 MHz, $CDCl_3$) δ 7.76 (d, *J* = 7.9 Hz, 1H), 7.71 (t, *J* = 7.6 Hz, 1H), 7.58 (t, *J* = 7.6 Hz, 1H), 7.48 (d, *J* = 7.8 Hz, 1H), 7.25 (dd, *J* = 8.6, 1.8 Hz, 2H), 6.92 – 6.84 (m, 2H), 5.03 (d, *J* = 15.4 Hz, 1H), 4.87 (d, *J* = 16.4 Hz, 1H), 3.81 (s, 3H), 3.77 – 3.62 (m, 1H), 3.32 (t, *J* = 6.9 Hz, 1H), 2.33 (s, 1H), 2.23 – 2.05 (m, 2H), 1.97 – 1.77 (m, 3H), 1.73 – 1.47 (m, 3H), 0.78 (t, *J* = 7.4 Hz, 3H).

^{13}C NMR (101 MHz, $CDCl_3$) δ 175.4, 158.9, 135.3, 134.32, 134.26, 130.8, 129.9, 129.1, 125.1, 121.9, 119.9, 113.6, 63.2, 58.5, 55.3, 47.4, 33.1, 31.7, 22.3, 16.4, 10.8.

HRMS (ESI, m/z): $[M+H]^+$ Calcd. For $C_{23}H_{28}N_3O_4S$: 442.1801; Found: 442.1803.

HPLC (Chiralpak OD-H Column), *i*-PrOH/hexane = 20/80, flow rate = 1.0 mL/min, λ = 210 nm; t_R = 14.2 min (major), t_R = 15.4 min (minor).

$[\alpha]_D^{25}$ = –49.2 (c = 0.5, $CHCl_3$).

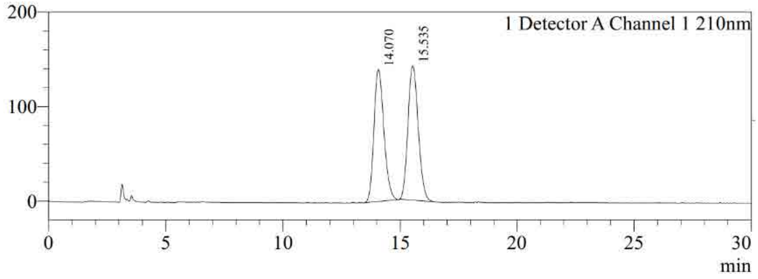

Detector A Channel 1 210nm

Peak#	Ret. Time	USP Width	Area	Height	Area%
1	14.070	0.783	4135927	139651	48.747
2	15.535	0.816	4348508	142276	51.253
Total			8484435	281927	100.000

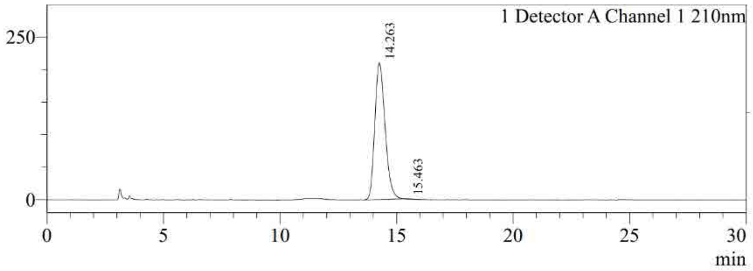

Detector A Channel 1 210nm

Peak#	Ret. Time	USP Width	Area	Height	Area%
1	14.263	0.805	6441885	210501	99.929
2	15.463	0.244	4602	238	0.071
Total			6446486	210739	100.000

7. Transformations & Applications

Synthesis of (R_{C-1}, S_{C-2})-34

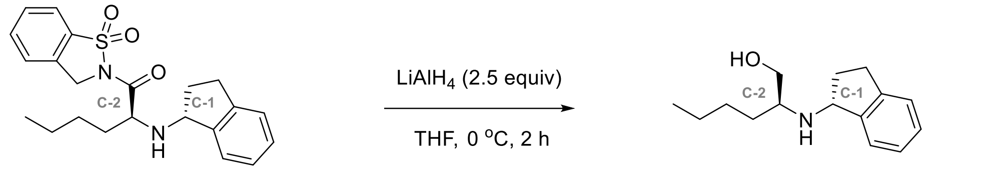

(R_{C-1}, S_{C-2})-**10b**, 98:2 d.r. (R_{C-1}, S_{C-2})-**34**, 99% yield, > 20:1 d.r.

Under N_2 atmosphere, a 50 mL flask charged with *α*-amino amide (R_{C-1}, S_{C-2})-**10b** (200 mg, 0.50 mmol, 1.0 equiv, 98:2 d.r.) and THF (2.0 mL) was cooled to 0 °C, followed by addition of $LiAlH_4$ (1.25 mL, 1.0 mol/mL in THF, 2.5 equiv) dropwise. The mixture was stirred at the same temperature for 2 h and then quenched with saturated sodium potassium tartrate solution. After extraction with ethyl acetate, the combined organic layer was washed with water, brine, and concentrated under reduced pressure to give the crude product. The residue was purified by flash chromatography over silica gel (eluent: dichloromethane/ethyl acetate = 5:1, then dichloromethane/methanol = 10:1) to give the desired product (R_{C-1}, S_{C-2})-**34** (115 mg, 99% yield, >20:1 d.r., signal of diastereomers cannot be observed in ^{1}H NMR) as white solid.

^{1}H NMR (400 MHz, $CDCl_3$) δ 7.50 (d, *J* = 7.6 Hz, 1H), 7.20 (dt, *J* = 8.4, 4.3 Hz, 1H), 6.95 – 6.78 (m, 2H), 4.63 (dd, *J* = 7.6, 3.7 Hz, 1H), 3.74 (d, *J* = 12.1 Hz, 1H), 3.58 (dd, *J* = 12.3, 6.4 Hz, 1H), 3.22 (dt, *J* = 15.7, 7.6 Hz, 1H), 3.06 – 2.94 (m, 1H), 2.85 (ddt, *J* = 13.7, 8.9, 4.6 Hz, 1H), 2.45 (dq, *J* = 15.3, 7.7 Hz, 1H), 2.21 (tq, *J* = 8.4, 4.4 Hz, 1H), 1.75 (s, 1H), 1.70 – 1.56 (m, 2H), 1.29 (ddh, *J* = 24.5, 16.8, 7.9 Hz, 4H), 0.87 (t, *J* = 6.9 Hz, 3H).

^{13}C NMR (101 MHz, $CDCl_3$) δ 144.9, 139.4, 129.2, 126.7, 125.7, 125.3, 61.2, 61.0, 58.4, 31.2, 30.5, 29.3, 28.2, 22.6, 14.0.

HRMS (ESI, m/z): $[M+H]^+$ Calcd. For $C_{15}H_{24}NO$: 234.1858; Found: 234.1859.

$[\alpha]_D^{25}$ = –2.65 (c = 1.0, $CHCl_3$).

Synthesis of (R_{C-1}, S_{C-2})-35

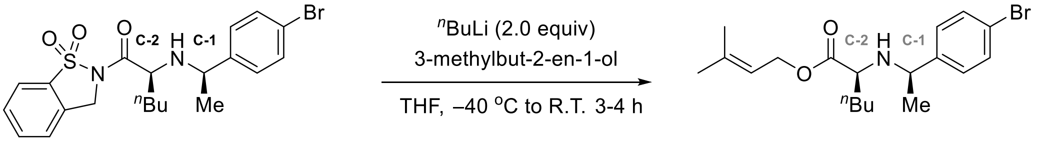

(R_{C-1}, S_{C-2})-**32a**, >99:1 d.r. (R_{C-1}, S_{C-2})-**35**, 88%, >20:1 d.r.

Under N_2 atmosphere, a 10 mL Schlenk tube charged with 3-methylbut-2-en-1-ol (91.3 mg, 1.06 mmol, 2.0 equiv) and THF (1.0 mL) was cooled to –40 °C, followed by addition of ^{n}BuLi (0.43 mL, 2.0 M in THF, 2.5 equiv). The resulting mixture was stirred at this temperature for 30 minutes and then *α*-amino amide (R_{C-1}, S_{C-2})-**32a** (246 mg, 0.53 mmol, 1.0 equiv, >99:1 d.r.) dissolved in THF (1.0 mL) was added dropwise. The reaction was warmed to room temperature and stirred continually for 3~4 h. After that, the reaction mixture was quenched with saturated aqueous NH_4Cl and extracted with ethyl acetate. The combined organic layer was washed with brine and concentrated under vacuo. The residue was purified by column chromatography on silica gel (eluent: petroleum

ether/ethyl acetate = 40:1 to 20:1) to give allyl ester ($R_{C\text{-}1}$, $S_{C\text{-}2}$)-**35** (179.1 mg, 88% yield, >20:1 d.r., signal of diastereomers cannot be observed in ^{1}H NMR) as a colorless oil.

^{1}H NMR (400 MHz, $CDCl_3$) δ 7.41 (d, *J* = 8.3 Hz, 2H), 7.19 (d, *J* = 8.3 Hz, 2H), 5.28 (t, *J* = 7.4 Hz, 1H), 4.50 (qd, *J* = 12.3, 7.2 Hz, 2H), 3.71 (q, *J* = 6.6 Hz, 1H), 3.25 (t, *J* = 6.6 Hz, 1H), 1.83 (s, 1H), 1.75 (s, 3H), 1.69 (s, 3H), 1.58 (dt, *J* = 12.3, 6.2 Hz, 2H), 1.30 (d, *J* = 6.5 Hz, 7H), 0.88 (t, *J* = 7.0 Hz, 3H).

^{13}C NMR (101 MHz, $CDCl_3$) δ 175.4, 144.5, 139.4, 131.5, 128.7, 120.8, 118.5, 61.5, 59.1, 55.9, 33.3, 27.8, 25.8, 22.8, 22.6, 18.1, 14.0.

HRMS (ESI, m/z): $[M+Na]^+$ Calcd. For $C_{19}H_{28}BrNO_2Na$: 404.1201; Found: 404.1206.

$[\alpha]_D^{25}$ = +30..6 (c = 1.0, $CHCl_3$).

Synthesis of ($R_{C\text{-}1}$, $S_{C\text{-}2}$)-36

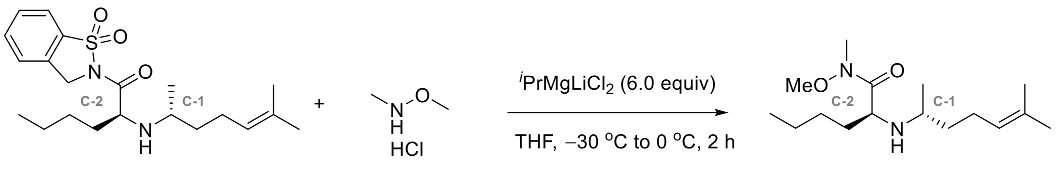

($R_{C\text{-}1}$, $S_{C\text{-}2}$)-**8a**, >99:1 d.r. ($R_{C\text{-}1}$, $S_{C\text{-}2}$)-**36**, 59% yield, >20:1 d.r.

Under N_2 atmosphere (*82*), a 10 mL Schlenk tube charged with *N*,*O*-dimethylhydroxylamine hydrochloride (58.5 mg, 0.60 mmol, 3.0 equiv) and THF (1.0 mL) was cooled to −30 °C, followed by addition of ^{i}PrMgLiCl$_2$ (0.92 mL, 1.3 M in THF, 6.0 equiv) and *α*-amino amide ($R_{C\text{-}1}$, $S_{C\text{-}2}$)-**8a** (78.5 mg, 0.20 mmol, 1.0 equiv, >99:1 d.r.) dissolved in THF (1.0 mL), separately. The reaction mixture was slowly warmed to 0 °C and stirred continually for 2 h. After that, the reaction mixture was quenched with saturated aqueous $NaHCO_3$ and extracted with ethyl acetate. The combined organic layer was washed with brine and concentrated under vacuo. The residue was purified by column chromatography on silica gel (eluent: petroleum ether/ethyl acetate = 8:1, then dichloromethane/ ethyl acetate = 10:1) to give Weinreb amide ($R_{C\text{-}1}$, $S_{C\text{-}2}$)-**36** (33.4 mg, 59% yield, >20:1 d.r., signal of diastereomers cannot be observed in ^{1}H NMR) as a colorless oil.

^{1}H NMR (400 MHz, $CDCl_3$) δ 5.07 (t, *J* = 7.3 Hz, 1H), 3.80 – 3.61 (m, 4H), 3.21 (s, 3H), 2.43 (q, *J* = 6.3 Hz, 1H), 1.97 (dd, *J* = 17.7, 9.8 Hz, 4H), 1.66 (s, 3H), 1.59 (s, 3H), 1.56 – 1.45 (m, 2H), 1.44 – 1.31 (m, 4H), 1.00 (d, *J* = 6.0 Hz, 3H), 0.88 (t, *J* = 6.6 Hz, 3H).

^{13}C NMR (101 MHz, $CDCl_3$) δ 177.4, 131.5, 124.5, 61.5, 54.7, 51.2, 38.2, 34.0, 32.4, 28.2, 25.8, 24.8, 22.8, 20.2, 17.8, 14.1.

HRMS (ESI, m/z): $[M+H]^+$ Calcd. For $C_{16}H_{33}N_2O_2$: 285.2542; Found: 285.2546.

$[\alpha]_D^{25}$ = −8.40 (c = 0.5, $CHCl_3$).

Synthesis of ($R_{C\text{-}1}$, $R_{C\text{-}2}$)-37

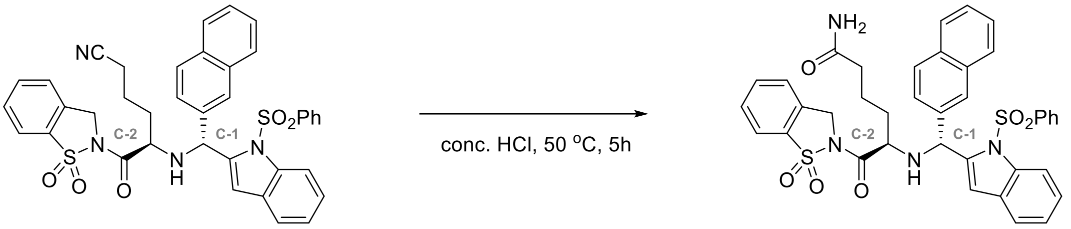

($R_{C\text{-}1}$, $R_{C\text{-}2}$)-**18b**, >99:1 d.r. ($R_{C\text{-}1}$, $R_{C\text{-}2}$)-**37**, 90% yield, >20:1 d.r.

Under N_2 atmosphere, the mixture of *α*-amino amide ($R_{C\text{-}1}$, $R_{C\text{-}2}$)-**18b** (69.0 mg, 0.10 mmol, >99:1 d.r.) and concentrated HCl (1.0 mL, 37% wt) was stirred at 50 °C for 5 h. After being cooled to room temperature, the reaction solution was transferred to saturated aqueous $NaHCO_3$ dropwise. The organic layer was extracted with ethyl acetate and concentrated under vacuo. The residue was purified by column chromatography on silica gel (eluent: petroleum ether/ethyl acetate/dichloromethane = 1:4:4, then ethyl acetate) to give amide ($R_{C\text{-}1}$, $R_{C\text{-}2}$)-**37** (63.2 mg, 90% yield, >20:1 d.r., signal of diastereomers cannot be observed in ^{1}H NMR) as a white solid.

^{1}H NMR (400 MHz, $CDCl_3$) δ 8.11 (d, J = 7.9 Hz, 1H), 7.81 – 7.70 (m, 3H), 7.69 – 7.62 (m, 2H), 7.61 – 7.51 (m, 3H), 7.45 (dd, J = 13.3, 7.7 Hz, 2H), 7.35 (t, J = 7.8 Hz, 2H), 7.32 – 7.26 (m, 4H), 7.17 (s, 1H), 7.09 (t, J = 7.6 Hz, 1H), 6.87 (t, J = 7.8 Hz, 2H), 5.88 (s, 1H), 5.81 (s, 1H), 5.70 – 5.55 (m, 1H), 4.76 (d, J = 15.8 Hz, 1H), 4.62 (d, J = 15.8 Hz, 1H), 4.43 (s, 1H), 2.58 (s, 1H), 2.42 – 2.20 (m, 2H), 2.15 – 1.89 (m, 3H), 1.76 – 1.57 (m, 1H).

^{13}C NMR (101 MHz, $CDCl_3$) δ 175.4, 174.7, 143.0, 138.8, 138.4, 137.8, 134.2, 134.1, 133.13, 133.07, 132.9, 130.8, 129.7, 129.3, 128.6, 128.2, 128.1, 127.8, 127.5, 126.6, 126.3, 126.1, 126.0, 125.1, 124.5, 123.6, 121.8, 121.1, 114.8, 110.9, 60.2, 59.4, 47.3, 35.1, 33.5, 21.9.

HRMS (ESI, m/z): $[M+H]^+$ Calcd. For $C_{38}H_{35}N_4O_6S_2$: 707.1998; Found: 707.1996.

$[α]_D^{25}$ = +58.4 (c = 0.5, $CHCl_3$).

Synthesis of ($S_{C\text{-}1}$, $S_{C\text{-}2}$, $S_{C\text{-}3}$)-38

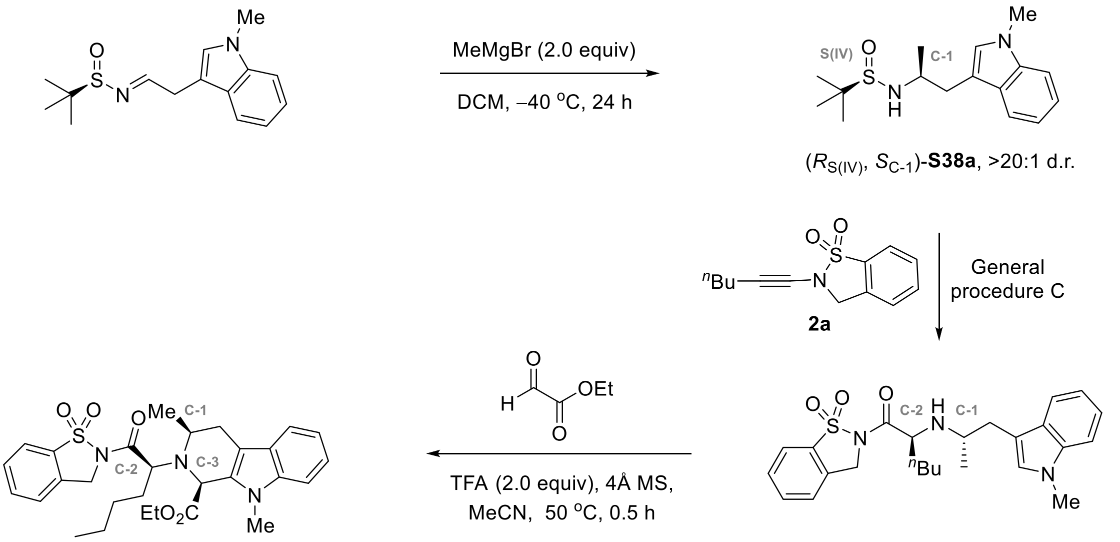

($S_{C\text{-}1}$, $S_{C\text{-}2}$, $S_{C\text{-}3}$)-**38**, >20:1 d.r. ($S_{C\text{-}1}$, $S_{C\text{-}2}$)-**S38b**, >20:1 d.r.

Synthesis of ($R_{S(IV)}$, $S_{C\text{-}1}$)-S38a

Based on the literature procedure (*83* and *84*): Under N_2 atmosphere, the solution of imine (1.42 mg, 5.14 mmol, 1.0 equiv) in dichloromethane (50 mL) was cooled at –40 °C, followed by addition of methylmagnesium bromide (3.43 mL, 3.0 M in THF, 2.0 equiv). The resulted mixture was stirred at the same temperature for 24 h and then quenched by saturated aqueous NH_4Cl. After extraction with ethyl acetate, the combined organic layer was washed with brine and concentrated under vacuo. The crude product was purified through flash chromatography (eluent: petroleum ether/ethyl acetate = 3:1 to 2:1) to afford ($R_{S(IV)}$, $S_{C\text{-}1}$)-**S38a** as reddish-brown oil (520 mg, 33% yield, >20:1 d.r., signal of diastereomers cannot be observed in ^{1}H NMR).

^{1}H NMR (400 MHz, $CDCl_3$) δ 7.61 (d, *J* = 7.9 Hz, 1H), 7.28 (d, *J* = 8.2 Hz, 1H), 7.25 – 7.19 (m, 1H), 7.12 (ddd, *J* = 8.1, 6.8, 1.3 Hz, 1H), 6.89 (s, 1H), 3.75 – 3.62 (m, 4H), 3.26 (d, *J* = 6.4 Hz, 1H), 3.03 (dd, *J* = 14.3, 5.9 Hz, 1H), 2.87 (dd, *J* = 14.3, 6.8 Hz, 1H), 1.33 (d, *J* = 6.5 Hz, 2H), 1.16 (s, 9H).

^{13}C NMR (101 MHz, $CDCl_3$) δ 136.8, 128.0, 127.6, 121.4, 118.9, 118.7, 110.4, 109.0, 55.4, 52.8, 33.6, 32.4, 22.4, 22.2.

HRMS (ESI, m/z): $[M+Na]^+$ Calcd. For $C_{16}H_{24}N_2ONaS$: 315.1507; Found: 315.1510.

$[\alpha]_D^{20}$ = –16.8 (c = 1.5, $CHCl_3$).

Synthesis of ($S_{C\text{-}1}$, $S_{C\text{-}2}$)-S38b

Prepared according to the **GENERAL PROCEDURE C** with ($R_{S(IV)}$, $S_{C\text{-}1}$)-**S38a** (300 mg, 1.03 mmol, >20:1 d.r.) and **2a** (213 mg, 0.856 mmol) as substrates. Column chromatography: silica gel, petroleum ether/ethyl acetate/dichloromethane = 10:1:1 to 5:1:1. Colorless viscous oil (180 mg, 46% yield, >20:1 d.r., signal of diastereomers cannot be observed in ^{1}H NMR).

^{1}H NMR (400 MHz, $CDCl_3$) δ 7.83 (d, *J* = 7.8 Hz, 1H), 7.70 (t, *J* = 7.6 Hz, 1H), 7.65 – 7.55 (m, 2H), 7.42 (d, *J* = 8.4 Hz, 1H), 7.22 – 7.12 (m, 2H), 7.09 – 7.02 (m, 1H), 6.93 (s, 1H), 5.04 – 4.56 (m, 2H), 4.37 – 4.13 (m, 1H), 3.65 (s, 3H), 3.08 – 2.90 (m, 2H), 2.80 – 2.66 (m, 1H), 2.04 (d, *J* = 7.9 Hz, 1H), 1.90 – 1.74 (m, 1H), 1.66 – 1.53 (m, 1H), 1.52 – 1.41 (m, 1H), 1.38 – 1.24 (m, 3H), 1.12 (d, *J* = 6.1 Hz, 3H), 0.86 (t, *J* = 6.5 Hz, 3H).

^{13}C NMR (101 MHz, $CDCl_3$) δ 175.2, 136.9, 134.4, 134.0, 130.9, 129.7, 128.4, 127.7, 125.1, 121.8, 121.3, 119.3, 118.6, 112.0, 109.0, 60.0, 53.0, 47.3, 33.6, 32.5, 28.0, 22.6, 21.9, 14.0.

HRMS (ESI, m/z): $[M+H]^+$ Calcd. For $C_{25}H_{32}N_3O_3S$: 425.2164; Found: 425.2169.

$[\alpha]_D^{25}$ = +30.4(c = 0.5, $CHCl_3$).

Synthesis of ($S_{C\text{-}1}$, $S_{C\text{-}2}$, $S_{C\text{-}3}$)-38

Under N_2 atmosphere, to the mixture of ($S_{C\text{-}1}$, $S_{C\text{-}2}$)-**S38b** (22.7 mg, 0.05 mmol, 1.0 equiv) and 4Å MS (10 mg) in dry acetonitrile was added ethyl glyoxylate (50% in toluene, 0.05 mL, 0.25 mmol, 5.0 equiv) and trifluoroacetic acid (7.2 ųL, 0.10 mmol, 2.0 equiv) separately. The resulted solution was stirred at 50 °C for 30 minutes and then quenched with saturated aqueous $NaHCO_3$. After extraction with ethyl acetate, the combined organic layer was washed with brine and concentrated under vacuo. The crude product was purified through flash chromatography (eluent: petroleum ether/ethyl acetate/dichloromethane = 20:1:2) to afford ($S_{C\text{-}1}$, $S_{C\text{-}2}$, $S_{C\text{-}3}$)-**38** as white solid (7.0

mg, 26% yield, >20:1 d.r., signal of diastereomers cannot be observed in ^{1}H NMR). The stereochemistry of **38** was determined by H-H COSY and H-H NOESY experiments.

^{1}H NMR (400 MHz, $CDCl_3$) δ 7.79 (d, *J* = 7.2 Hz, 1H), 7.63 (t, *J* = 7.6 Hz, 1H), 7.56 (t, *J* = 7.6 Hz, 1H), 7.37 (d, *J* = 7.0 Hz, 1H), 7.32 (d, *J* = 8.2 Hz, 1H), 7.23 (d, *J* = 7.7 Hz, 1H), 7.19 (ddd, *J* = 8.3, 7.0, 1.2 Hz, 1H), 7.05 – 6.97 (m, 1H), 5.51 (s, 1H), 4.65 (d, *J* = 15.9 Hz, 1H), 4.42 – 4.35 (m, 1H), 4.34 – 4.17 (m, 2H), 3.98 – 3.86 (m, 1H), 3.75 (s, 3H), 3.72 – 3.62 (m, 1H), 2.75 – 2.63 (m, 2H), 1.98 – 1.68 (m, 3H), 1.57 (d, *J* = 6.8 Hz, 3H), 1.54 – 1.38 (m, 3H), 1.34 (t, *J* = 7.2 Hz, 3H), 0.99 (t, *J* = 7.3 Hz, 3H).

^{13}C NMR (101 MHz, $CDCl_3$) δ 173.0, 171.7, 137.4, 134.4, 134.1, 133.1, 131.1, 129.6, 126.7, 125.0, 121.7, 121.1, 118.5, 118.2, 110.0, 109.1, 61.3, 55.8, 55.4, 51.7, 47.1, 31.8, 30.2, 28.2, 26.7, 22.6, 18.4, 14.3, 14.2.

HRMS (ESI, m/z): $[M+H]^+$ Calcd. For $C_{29}H_{36}N_3O_5S$: 538.2376; Found: 538.2379.

$[\alpha]_D^{25}$ = +89.4 (c = 1.0, $CHCl_3$).

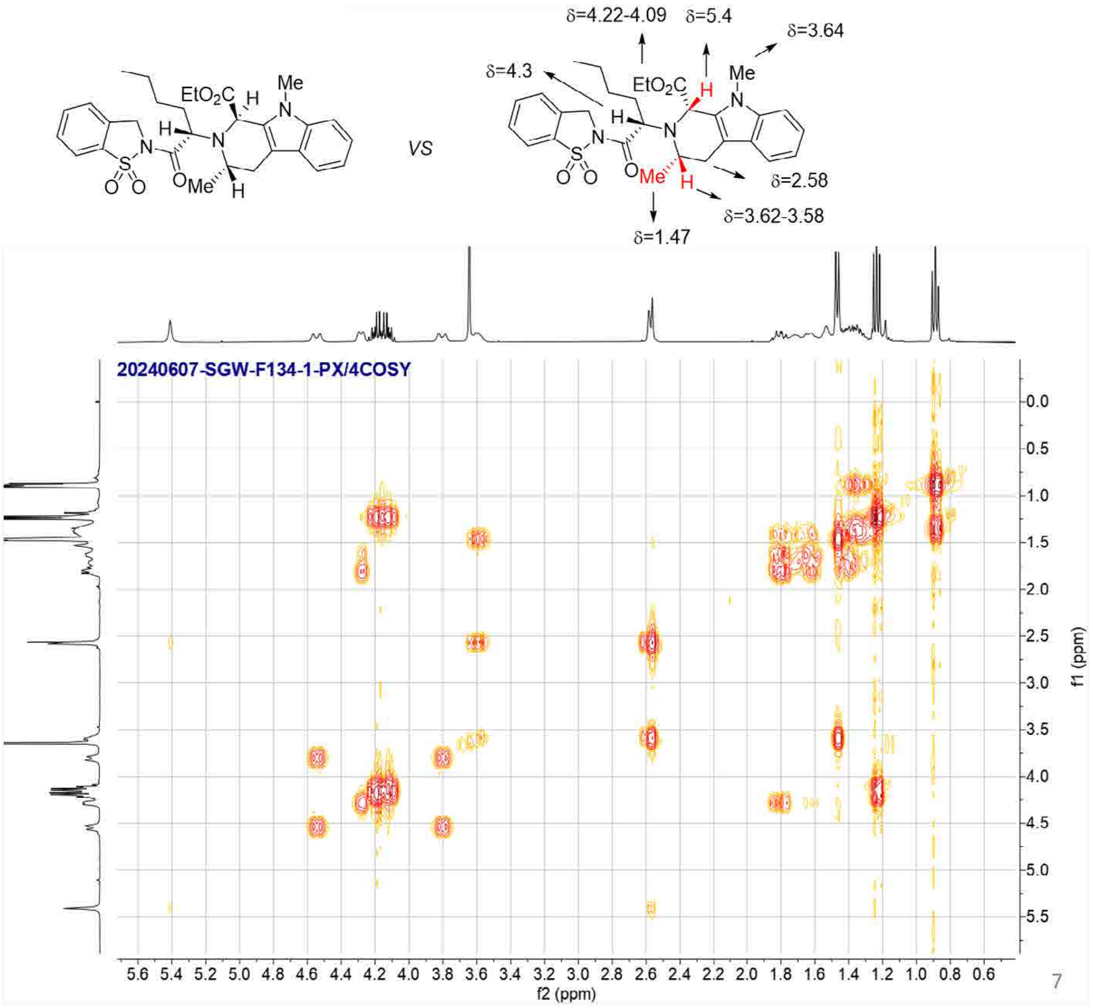

Figure S9. H-H COSY spectra of **38.**

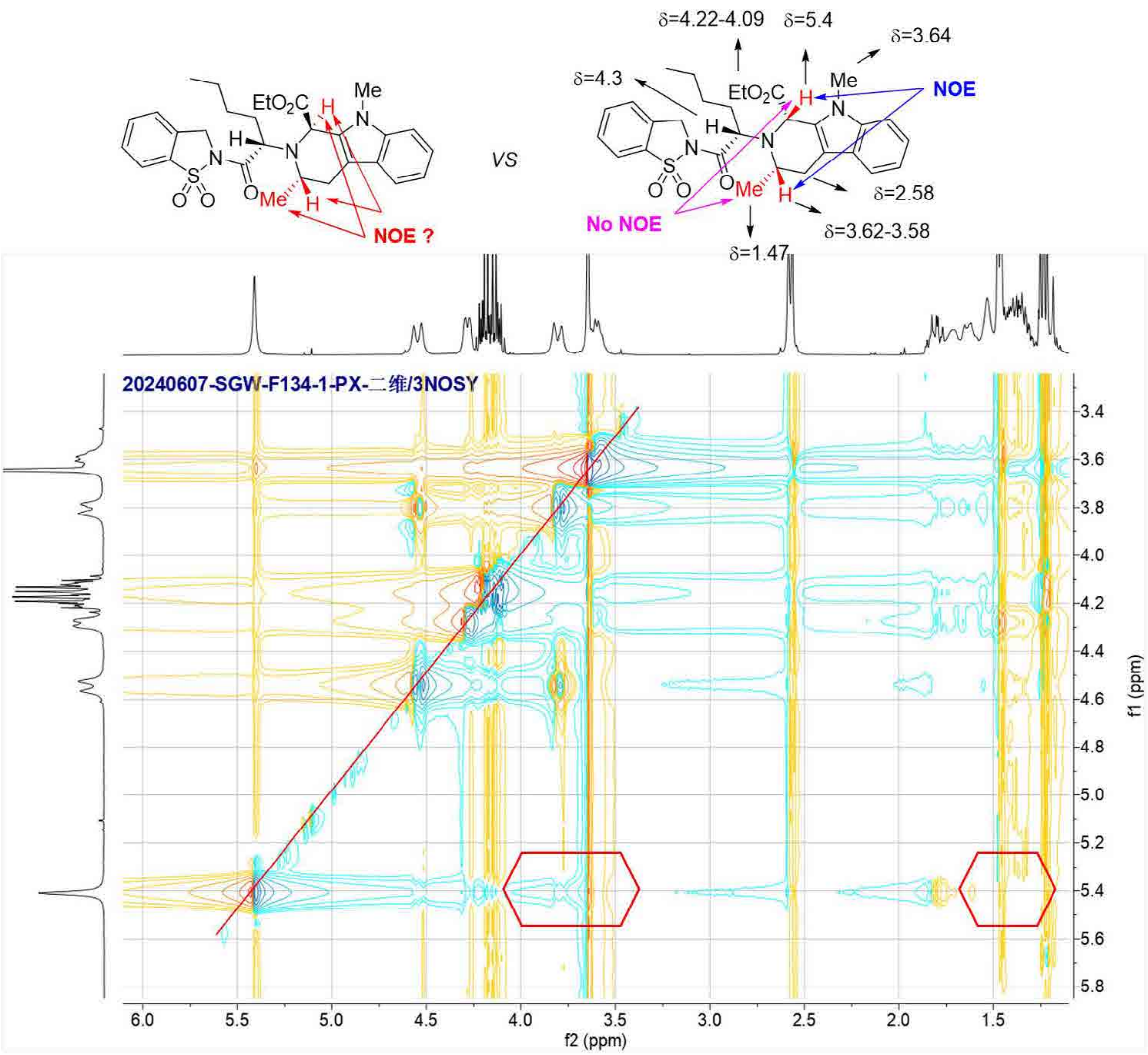

Figure S10. H-H NOESY spectrum of **38.**

Synthesis of (R_{C-1}, S_{C-2})-39

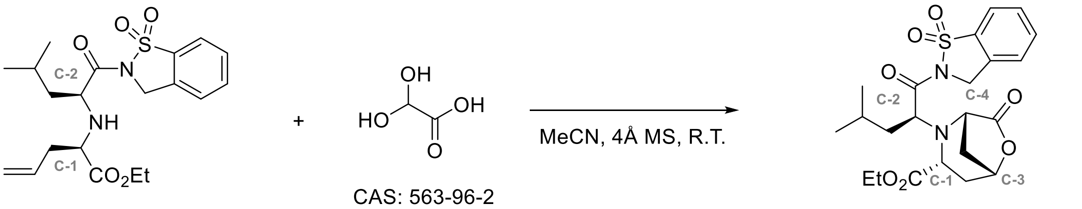

Based on the known literature procedure (*59*): Under N_2 atmosphere, the mixture of *α*-amino amide (R_{C-1}, S_{C-2})-**26a** (45.1 mg, 0.11 mmol, 1.0 equiv, >20:1 d.r.), glyoxylic acid monohydrate (12.6 mg, 0.137 mmol, 1.25 equiv) and 4Å molecular sieves (20 mg) in MeCN (1.0 mL) was stirred at room temperature overnight. After that, the mixture was concentrated under vacuo and purified by column chromatography on silica gel (eluent: petroleum

ether/ethyl acetate/dichloromethane = 4:1:1) to give the desired product ($R_{C\text{-}1}$, $S_{C\text{-}2}$, $S_{C\text{-}3}$, $S_{C\text{-}4}$)-**39** (27.1 mg, 51% yield, >20:1 d.r., signal of diastereomers cannot be observed in ^{1}H NMR) as a colorless viscous oil. The stereochemistry of **39** was determined by H-H COSY and H-C HMBC experiments.

^{1}H NMR (400 MHz, $CDCl_3$) δ 7.75 (d, J = 7.8 Hz, 1H), 7.69 (td, J = 7.6, 1.2 Hz, 1H), 7.57 (t, J = 7.4 Hz, 1H), 7.48 (d, J = 7.7 Hz, 1H), 5.03 (d, J = 16.0 Hz, 1H), 4.92 (d, J = 16.3 Hz, 1H), 4.80 (t, J = 5.3 Hz, 1H), 4.18 (dd, J = 10.0, 4.3 Hz, 1H), 4.14 – 4.04 (m, 2H), 4.00 (q, J = 7.2 Hz, 2H), 2.61 (ddd, J = 14.2, 4.8, 2.1 Hz, 1H), 2.25 (dd, J = 13.6, 9.1 Hz, 1H), 2.16 (dddd, J = 12.0, 6.1, 4.2, 2.0 Hz, 1H), 1.90 – 1.76 (m, 3H), 1.49 (ddd, J = 14.7, 10.3, 4.1 Hz, 1H), 1.13 (t, J = 7.2 Hz, 3H), 0.96 (t, J = 6.1 Hz, 6H).

^{13}C NMR (101 MHz, $CDCl_3$) δ 173.7, 173.6, 172.5, 134.3, 134.2, 131.1, 129.7, 125.2, 121.7, 63.1, 61.3, 58.0, 54.1, 47.3, 40.4, 37.3, 32.2, 24.8, 23.6, 21.5, 14.0.

HRMS (ESI, m/z): $[M+Na]^+$ Calcd. For $C_{22}H_{28}N_2O_7NaS$: 487.1515; Found: 487.1519.

$[\alpha]_D^{25}$ = −7.6 (c = 0.5, $CHCl_3$).

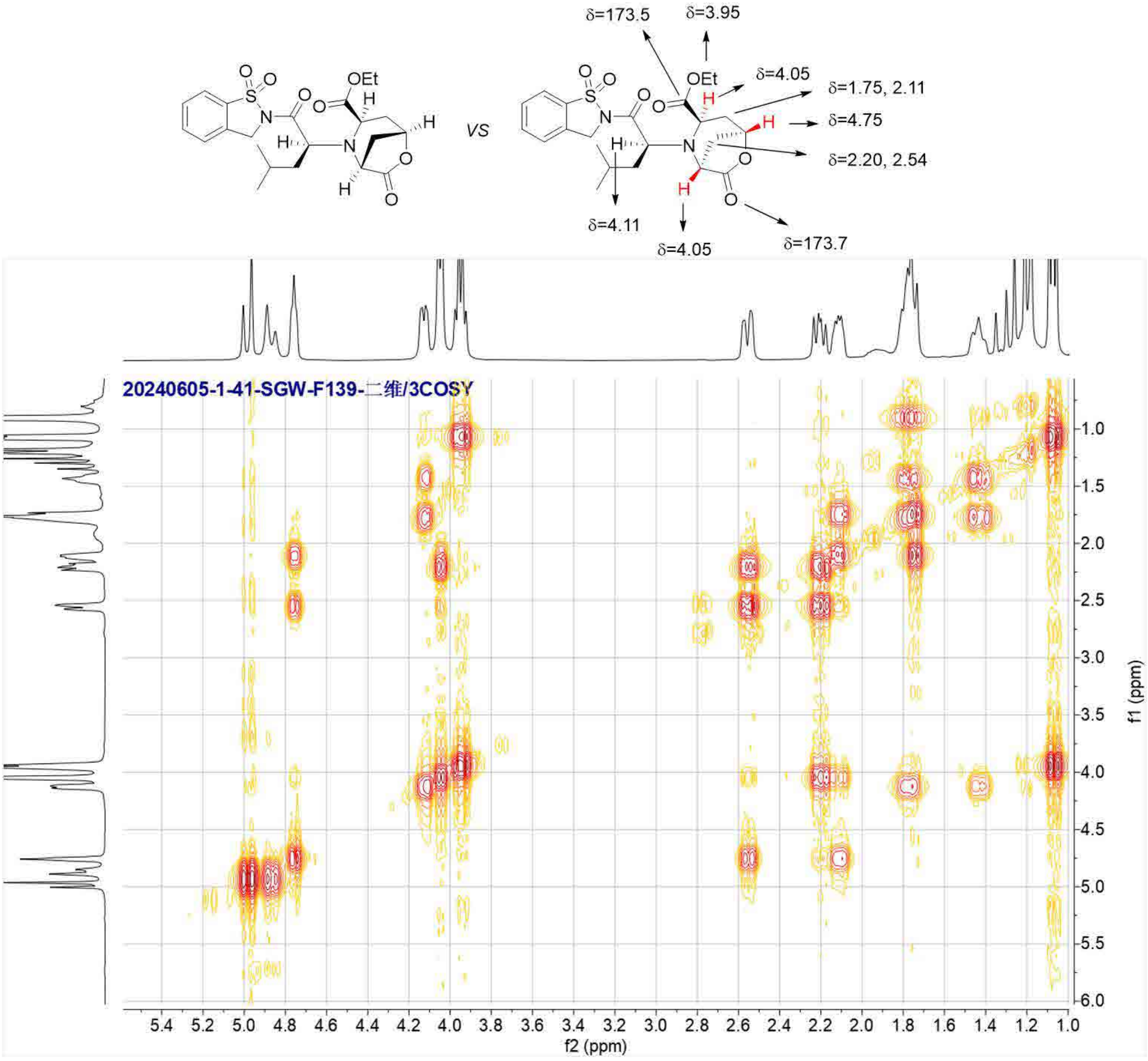

Figure S11. H-H COSY spectra of **39.**

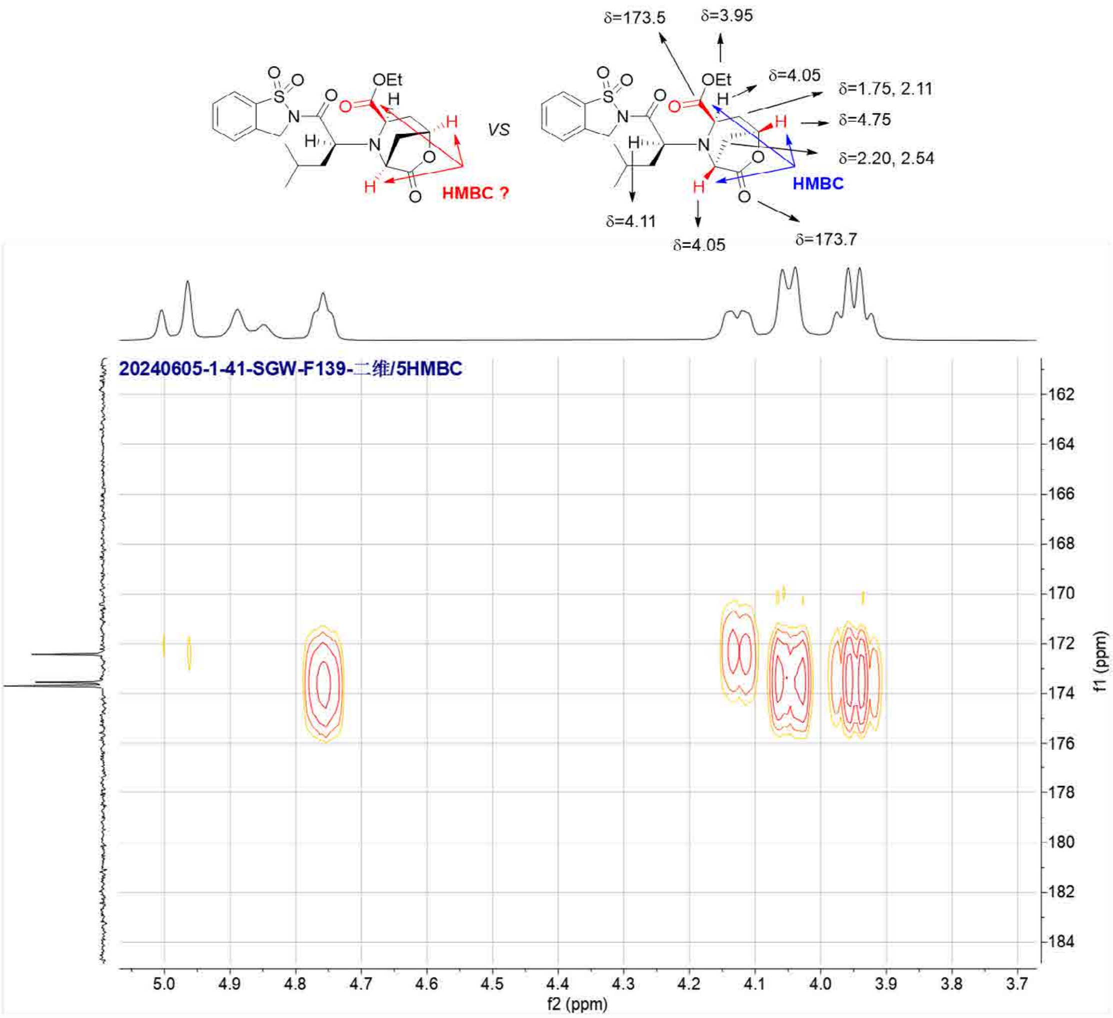

Figure S12. H-C HMBC spectra of **39.**

Synthesis of (R_{C-1}. S_{C-2})-40

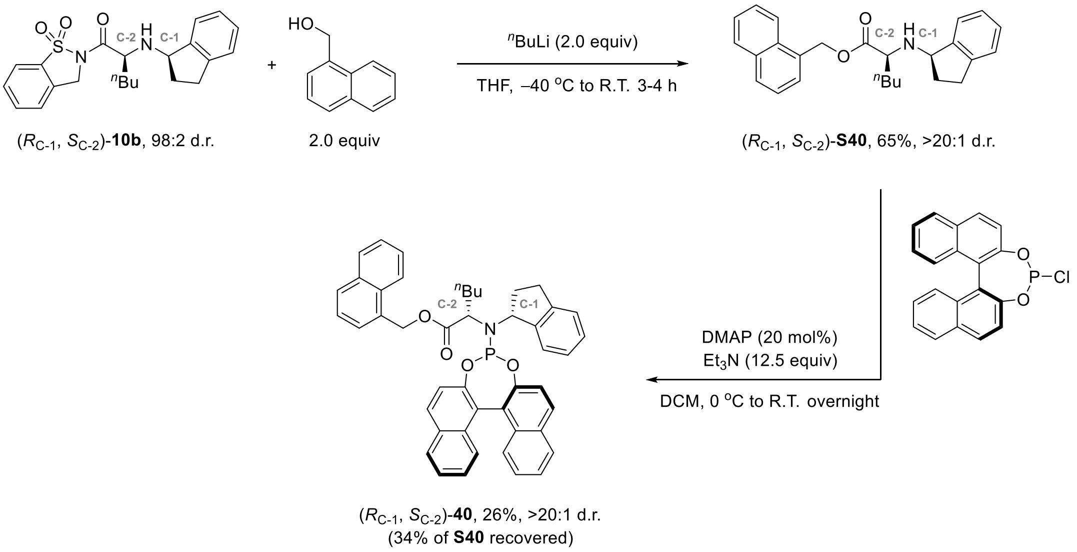

Synthesis of ($R_{C\text{-}1}$, $S_{C\text{-}2}$)-**S40**

Under N_2 atmosphere, a 50 mL round bottom flask charged with naphthalen-1-ylmethanol (158 mg, 1.00 mmol, 2.0 equiv) and THF (2.0 mL) was cooled to –40 °C, followed by addition of ^{n}BuLi (0.4 mL, 2.5 M in THF, 2.0 equiv). The resulting mixture was stirred at this temperature for 30 minutes and then α-amino amide ($R_{C\text{-}1}$, $S_{C\text{-}2}$)-**10b** (199 mg, 0.50 mmol, 1.0 equiv, 98:2 d.r.) dissolved in THF (1.0 mL) was added dropwise. The reaction was warmed to room temperature and stirred continually for 3–4 hours. After that, the reaction mixture was quenched with saturated aqueous NH_4Cl and extracted with ethyl acetate. The combined organic layer was washed with brine and concentrated under vacuo. The residue was purified by column chromatography on silica gel (eluent: petroleum ether/ethyl acetate = 100:1 to 40:1) to give ester ($R_{C\text{-}1}$, $S_{C\text{-}2}$)-**S40** (126 mg, 65% yield, >20:1 d.r., signal of diastereomers cannot be observed in ^{1}H NMR) as a white solid.

^{1}H NMR (400 MHz, $CDCl_3$) δ 8.07 (d, J = 7.7 Hz, 1H), 7.91 (t, J = 9.0 Hz, 2H), 7.65 – 7.46 (m, 4H), 7.28 – 7.14 (m, 4H), 5.72 (d, J = 12.5 Hz, 1H), 5.67 (d, J = 12.5 Hz, 1H), 4.17 (t, J = 6.2 Hz, 1H), 3.46 (t, J = 6.7 Hz, 1H), 3.03 (dt, J = 14.9, 6.9 Hz, 1H), 2.78 (dt, J = 15.4, 7.3 Hz, 1H), 2.23 (dq, J = 13.5, 6.8 Hz, 1H), 1.98 (s, 1H), 1.83 (dq, J = 13.3, 6.6 Hz, 1H), 1.65 (dh, J = 21.5, 7.0 Hz, 2H), 1.30 (ddd, J = 25.1, 12.6, 6.7 Hz, 4H), 0.83 (t, J = 6.9 Hz, 3H).

^{13}C NMR (101 MHz, $CDCl_3$) δ 176.1, 144.8, 143.8, 133.8, 131.7, 131.4, 129.6, 128.8, 128.0, 127.6, 126.7, 126.4, 126.1, 125.3, 124.8, 123.7, 123.6, 64.9, 61.7, 59.8, 33.9, 33.0, 30.5, 28.0, 22.5, 13.9.

Synthesis of ($R_{C\text{-}1}$, $S_{C\text{-}2}$)-**40**

Step1: According to the reported procedure (*85*): The mixture of (*S*)-[1,1'-binaphthalene]-2,2'-diol (573 mg, 2.00 mmol, 1.0 equiv), phosphorus trichloride (1.92 mL, 11 equiv) and one drop of DMF was stirred at 50 °C for 20 minutes, under N_2 atmosphere. After being cooled to room temperature, all volatiles were removed under reduced pressure and the traces of phosphorus trichloride was removed with dry CH_2Cl_2 (3×5 mL). The resulting **phosphorous chloride** was kept under vacuo for 1 h and was used directly for next step.

Step2: A 50 mL round bottom flask charged with ester ($R_{C\text{-}1}$, $S_{C\text{-}2}$)-**S40** (126 mg, 0.32 mmol, 1.0 equiv, >20:1 d.r.), Et_3N (404 mg, 4.0 mmol, 12.5 equiv), DMAP (7.82 mg, 0.064 mmol, 0.2 equiv) and dichloromethane (1.0 mL) was cooled to 0 °C, followed by addition of **Phosphorous chloride** (dissolved in 1.0 mL dichloromethane), dropwise. The reaction was then warmed to room temperature and stirred overnight. After that, the reaction mixture was quenched with saturated aqueous NH_4Cl and extracted with dichloromethane. The combined organic layer was washed with brine and concentrated under vacuo. The residue was purified by column chromatography on silica gel (eluent: petroleum ether/ethyl acetate/dichloromethane = 100:1:1 to 50:1:1) to give phosphor amide ($R_{C\text{-}1}$, $S_{C\text{-}2}$)-**40** (58.5 mg, 26% yield, >20:1 d.r., signal of diastereomers cannot be observed in ^{1}H NMR) as a white solid.

^{1}H NMR (400 MHz, $CDCl_3$) δ 8.14 (d, J = 8.2 Hz, 1H), 8.00 (d, J = 8.7 Hz, 1H), 7.95 (t, J = 8.4 Hz, 3H), 7.85 (d, J = 8.4 Hz, 1H), 7.74 (t, J = 7.6 Hz, 2H), 7.64 – 7.56 (m, 3H), 7.54 (t, J = 7.8 Hz, 1H), 7.43 (t, J = 7.5 Hz, 1H), 7.36 (dd, J = 13.7, 8.2 Hz, 4H), 7.28 – 7.18 (m, 3H), 7.13 (p, J = 7.2 Hz, 2H), 7.03 (d, J = 7.1 Hz, 1H), 5.85 – 5.71 (m, 2H), 4.70 (t, J = 8.8 Hz, 1H), 3.36 (dt, J = 16.4, 7.9 Hz, 1H), 2.72 (dt, J = 15.4, 7.3 Hz, 1H), 2.37 (dt, J = 16.0, 7.8 Hz, 1H), 2.04 (h, J = 7.9, 7.2 Hz, 1H), 1.92 (p, J = 8.1, 7.0 Hz, 1H), 1.80 – 1.61 (m, J = 7.8 Hz, 2H), 1.01 (qt, J = 20.9, 11.6 Hz, 3H), 0.74 (t, J = 7.0 Hz, 3H).

^{13}**C NMR** (101 MHz, $CDCl_3$) δ 175.5, 150.0, 149.71 (d, J = 6.6 Hz), 143.7, 142.5, 133.9, 132.9, 132.7, 131.8, 131.5, 131.3, 130.7, 130.3, 129.7, 129.3, 128.9, 128.4, 128.2, 127.7, 127.3, 127.2, 126.8, 126.6, 126.2, 126.1, 126.0, 125.6, 125.5, 124.8, 124.6, 124.4, 124.12 (d, J = 5.2 Hz), 123.8, 123.0, 122.43, 122.36, 65.3, 60.2, 58.19 (d, J = 20.7 Hz), 32.96 (d, J = 23.8 Hz), 30.4, 28.34 (d, J = 3.0 Hz), 22.0, 13.9.

^{31}**P NMR** (162 MHz, $CDCl_3$) δ 143.02.

HRMS (ESI, m/z): $[M+Na]^+$ Calcd. For $C_{46}H_{40}NO_4NaP$: 724.2593; Found: 724.2591.

$[\alpha]_D^{25}$ = +229.5 (c = 0.4, $CHCl_3$).

Synthesis of ($S_{C\text{-}1}$, $S_{C\text{-}2}$)-41a & ($S_{C\text{-}1}$, $S_{C\text{-}2}$)-42a

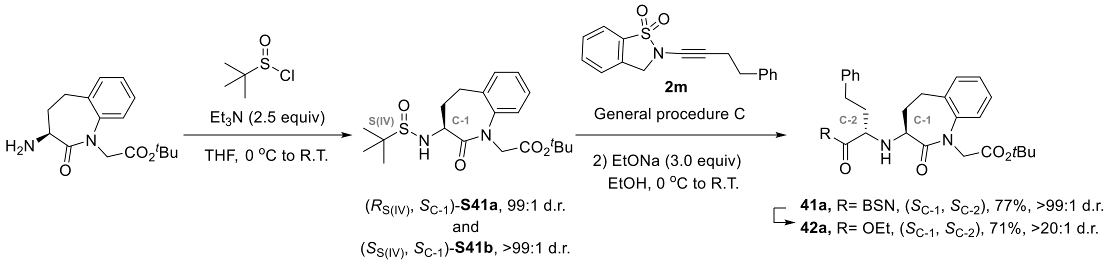

Synthesis of ($R_{S(IV)}$, $S_{C\text{-}1}$)-S41a

Under N_2 atmosphere, the mixture of (S)-ATBA (2.90 g, 10 mmol, 1.0 equiv) and Et_3N (2.53 g, 25 mmol, 2.5 equiv) in THF was cooled at 0 °C and then 2-methylpropane-2-sulfinic chloride (2.0 equiv) dissolved in THF was added slowly. The resulted mixture was stirred at room temperature overnight and then quenched with saturated aqueous NH_4Cl, extracted with ethyl acetate. The combined organic layer was washed with brine and concentrated under vacuo. The crude product was purified through flash chromatography (eluent: petroleum ether/ethyl acetate = 3:1 to 1:1) to afford ($R_{S(IV)}$, $S_{C\text{-}1}$)-**S41a** as colorless viscous oil (640 mg, 16% yield, 99:1 d.r.) and ($S_{S(IV)}$, $S_{C\text{-}1}$)-**S41b** as white solid (2.70 g, 68% yield, >99:1 d.r.). These two compounds were used for the preparation of **41a** (**42a**) and **41b** (**42b**) respectively.

^{1}**H NMR** (400 MHz, $CDCl_3$) δ 7.32 – 7.26 (m, 2H), 7.24 – 7.17 (m, 2H), 7.12 (d, J = 7.9 Hz, 1H), 4.60 (d, J = 17.0 Hz, 1H), 4.26 (d, J = 17.0 Hz, 1H), 4.19 (d, J = 9.4 Hz, 1H), 4.00 – 3.89 (m, 1H), 3.38 – 3.25 (m, 1H), 2.69 – 2.56 (m, 2H), 2.21 (td, J = 11.7, 7.9 Hz, 1H), 1.40 (s, 9H), 1.16 (s, 9H).

^{13}**C NMR** (101 MHz, $CDCl_3$) δ 171.9, 167.7, 140.8, 135.8, 129.7, 128.0, 127.3, 122.6, 82.3, 56.6, 55.2, 51.3, 39.3, 28.4, 28.1, 22.6.

HRMS (ESI, m/z): $[M+Na]^+$ Calcd. For $C_{20}H_{30}N_2O_4NaS$: 417.1824; Found: 417.1826.

HPLC (Chiralpak OD-H Column), i-PrOH/hexane = 3/97, flow rate = 1.0 mL/min, λ = 210 nm; t_R = 27.4 min (major), t_R = 30.0 min (minor).

$[\alpha]_D^{20}$ = –214.8 (c = 1.0, $CHCl_3$).

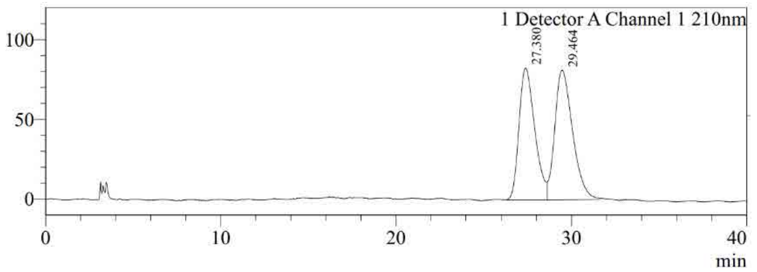

Detector A Channel 1 210nm

Peak#	Ret. Time	USP Width	Area	Height	Area%
1	27.380	1.664	5211082	82883	47.535
2	29.464	1.835	5751443	81268	52.465
Total			10962526	164151	100.000

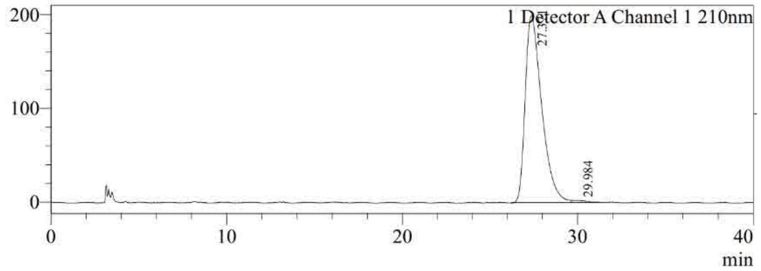

Detector A Channel 1 210nm

Peak#	Ret. Time	USP Width	Area	Height	Area%
1	27.371	1.689	13004024	198957	99.087
2	29.984	2.948	119802	2185	0.913
Total			13123826	201142	100.000

Synthesis of ($S_{C\text{-}1}$, $S_{C\text{-}2}$)-41a

Prepared according to the **GENERAL PROCEDURE C** with ($R_{S(IV)}$, $S_{C\text{-}1}$)-**S41a** (118 mg, 0.30 mmol, 99:1 d.r.) and **2m** (59.4 mg, 0.20 mmol) as substrates. Column chromatography: silica gel, petroleum ether/ethyl acetate/dichloromethane = 4:1:1. White solid (93.2 mg, 77% yield, >99:1 d.r.).

^{1}H NMR (400 MHz, $CDCl_3$) δ 7.79 (d, J = 7.9 Hz, 1H), 7.67 (t, J = 7.5 Hz, 1H), 7.57 (t, J = 7.6 Hz, 1H), 7.40 (d, J = 7.8 Hz, 1H), 7.24 – 7.10 (m, 8H), 7.08 (d, J = 8.2 Hz, 1H), 4.83 (d, J = 16.3 Hz, 1H), 4.74 (d, J = 15.5 Hz, 1H), 4.27 – 4.05 (m, 2H), 3.42 – 3.19 (m, 2H), 2.88 (dp, J = 11.6, 5.3 Hz, 1H), 2.75 – 2.61 (m, 3H), 2.55 (dd, J = 13.5, 6.8 Hz, 1H), 2.35 (dh, J = 13.2, 6.8, 6.2 Hz, 1H), 2.02 (dddd, J = 35.9, 24.1, 12.9, 6.3 Hz, 3H), 1.38 (s, 9H).

^{13}C NMR (101 MHz, $CDCl_3$) δ 173.5, 173.0, 168.0, 141.5, 141.0, 136.2, 134.4, 134.1, 130.9, 129.7, 129.4, 128.5, 128.4, 127.7, 126.6, 125.9, 125.0, 122.5, 121.9, 81.9, 60.1, 55.9, 50.9, 47.4, 37.9, 34.9, 32.2, 28.5, 28.1.

HRMS (ESI, m/z): $[M+H]^+$ Calcd. For $C_{33}H_{38}N_3O_6S$: 604.2481; Found: 604.2482.

HPLC (Chiralpak OD-H Column), *i*-PrOH/hexane = 10/90, flow rate = 1.0 mL/min, λ = 254 nm; t_R = 22.7 min (minor), t_R = 29.2 min (major).

$[\alpha]_D^{25}$ = –106.9 (c = 0.5, $CHCl_3$).

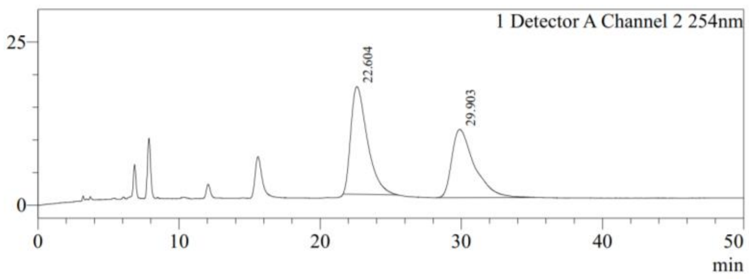

Detector A Channel 2 254nm

Peak#	Ret. Time	USP Width	Area	Height	Area%
1	22.604	2.120	1351560	16515	53.647
2	29.903	2.706	1167804	10456	46.353
Total			2519364	26971	100.000

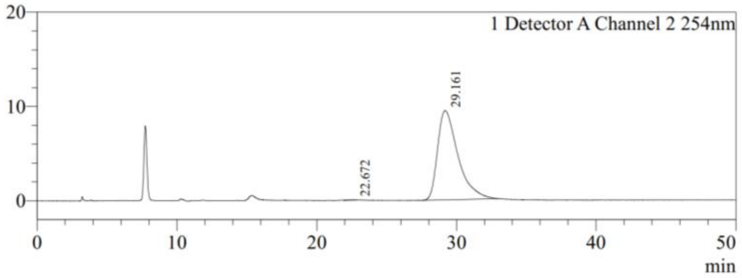

Detector A Channel 2 254nm

Peak#	Ret. Time	USP Width	Area	Height	Area%
1	22.672	0.613	1895	41	0.196
2	29.161	2.517	963288	9473	99.804
Total			965183	9514	100.000

Synthesis of ($S_{C\text{-}1}$, $S_{C\text{-}2}$)**-42a** (*61*)

Under N_2 atmosphere, a 10 mL Schlenk tube charged with α-amino amide ($S_{C\text{-}1}$, $S_{C\text{-}2}$)-**41a** (93.2 mg, 0.15 mmol, 1.0 equiv, >99:1 d.r.) and dry ethanol (2.0 mL) was cooled to 0 °C, followed by addition of sodium ethanol (31.4 mg, 0.45 mmol, 3.0 equiv). After addition, the reaction was warmed to room temperature and stirred continually for 2~4 h (monitored by TLC). Once amide was consumed completely, the reaction mixture was quenched with saturated aqueous NH_4Cl and extracted with ethyl acetate. The combined organic layer was washed with brine and concentrated under vacuo. The residue was purified by column chromatography on silica gel (eluent: petroleum ether = 5:1 to 4:1) to give the ester ($S_{C\text{-}1}$, $S_{C\text{-}2}$)-**42a** as white solid (52.8 mg, 71% yield, >20:1 d.r., signal of diastereomers cannot be observed in ^{1}H NMR).

^{1}H NMR (400 MHz, $CDCl_3$) δ 7.30 – 7.13 (m, 8H), 7.10 (d, *J* = 8.0 Hz, 1H), 4.60 (d, *J* = 17.0 Hz, 1H), 4.30 (d, *J* = 17.0 Hz, 1H), 4.13 – 3.93 (m, 2H), 3.33 – 3.16 (m, 3H), 2.76 – 2.62 (m, 2H), 2.57 (dd, *J* = 13.7, 6.7 Hz, 1H), 2.39 (tt, *J* = 13.7, 7.3 Hz, 1H), 2.23 (s, 1H), 1.98 (dp, *J* = 27.0, 9.0, 8.5 Hz, 3H), 1.42 (s, 9H), 1.11 (t, *J* = 7.1 Hz, 3H).

^{13}C NMR (101 MHz, $CDCl_3$) δ 174.2, 173.9, 167.9, 141.5, 141.1, 136.2, 129.5, 128.5, 128.4, 127.8, 126.8, 126.0, 122.3, 82.1, 60.6, 60.3, 57.0, 51.3, 37.9, 35.2, 32.2, 28.6, 28.1, 14.2.

HRMS (ESI, m/z): $[M+H]^+$ Calcd. For $C_{28}H_{37}N_2O_5$: 481.2702; Found: 481.2705.

$[\alpha]_D^{25}$ = –151.2 (c = 0.5, $CHCl_3$).

Synthesis of ($S_{\text{C-1}}$, $R_{\text{C-2}}$)-41b & ($S_{\text{C-1}}$, $R_{\text{C-2}}$)-42b

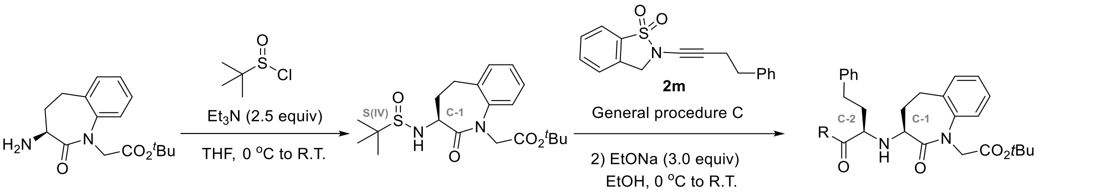

($S_{\text{S(IV)}}$, $S_{\text{C-1}}$)-**S41b**, >99:1 d.r.
and
($R_{\text{S(IV)}}$, $S_{\text{C-1}}$)-**S41a**, 99:1 d.r.

41b, R= BSN, ($S_{\text{C-1}}$, $R_{\text{C-2}}$), 85%, >99:1 d.r.
42b, R= OEt, ($S_{\text{C-1}}$, $R_{\text{C-2}}$), 77%, >20:1 d.r.

Synthesis of ($S_{\text{S(IV)}}$, $S_{\text{C-1}}$)-**S41b**

The title compound was obtained as an isomer during the the preparation of **S41a**. White solid (2.70 g, 68% yield, >99:1 d.r.).

^{1}H NMR (400 MHz, $CDCl_3$) δ 7.29 – 7.25 (m, 1H), 7.22 – 7.14 (m, 2H), 7.09 (d, J = 7.9 Hz, 1H), 4.81 (d, J = 17.0 Hz, 1H), 4.56 (d, J = 6.1 Hz, 1H), 4.12 (d, J = 17.1 Hz, 1H), 3.90 (ddd, J = 11.1, 8.0, 6.1 Hz, 1H), 3.41 (td, J = 13.3, 8.0 Hz, 1H), 2.55 (dd, J = 13.6, 7.0 Hz, 1H), 2.39 (tt, J = 12.9, 7.5 Hz, 1H), 2.18 – 1.99 (m, 1H), 1.37 (s, 9H), 1.17 (s, 9H).

^{13}C NMR (101 MHz, $CDCl_3$) δ 172.0, 167.5, 140.4, 135.9, 129.5, 127.9, 127.1, 122.9, 82.1, 55.8, 54.6, 51.1, 39.0, 28.0, 22.7.

HRMS (ESI, m/z): $[M+Na]^+$ Calcd. For $C_{20}H_{30}N_2O_4NaS$: 417.1824; Found: 417.1827.

HPLC (Chiralpak OD-H Column), *i*-PrOH/hexane = 3/97, flow rate = 1.0 mL/min, λ = 210 nm; t_R = 27.2 min (minor), t_R = 28.6 min (major).

$[\alpha]_D^{20}$ = –87.6 (c = 1.0, $CHCl_3$).

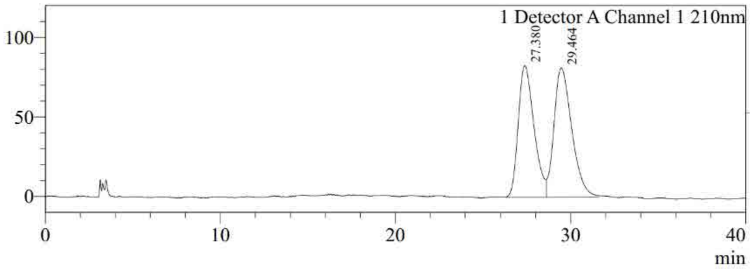

Detector A Channel 1 210nm

Peak#	Ret. Time	USP Width	Area	Height	Area%
1	27.380	1.664	5211082	82883	47.535
2	29.464	1.835	5751443	81268	52.465
Total			10962526	164151	100.000

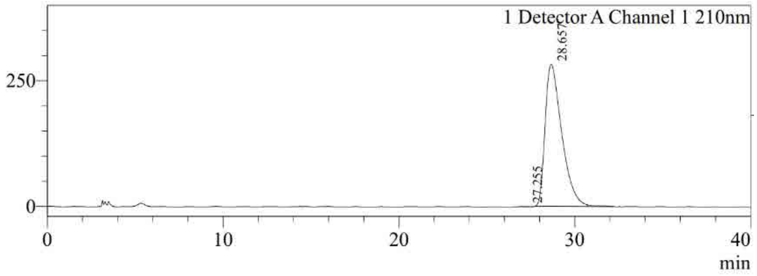

Detector A Channel 1 210nm

Peak#	Ret. Time	USP Width	Area	Height	Area%
1	27.255	0.197	6699	297	0.036
2	28.657	1.710	18645579	282712	99.964
Total			18652278	283009	100.000

Synthesis of ($S_{C\text{-}1}$, $R_{C\text{-}2}$)**-41b**

Prepared according to the **GENERAL PROCEDURE C** with ($S_{S(IV)}$, $S_{C\text{-}1}$)**-S41b** (118 mg, 0.30 mmol, >99:1 d.r.) and **2m** (59.4 mg, 0.20 mmol) as substrates. Column chromatography: silica gel, petroleum ether/ethyl acetate/dichloromethane = 4:1:1. White solid (103.7 mg, 85% yield, >99:1 d.r.).

^{1}**H NMR** (400 MHz, $CDCl_3$) δ 7.78 (d, J = 7.9 Hz, 1H), 7.69 (t, J = 7.5 Hz, 1H), 7.57 (t, J = 7.7 Hz, 1H), 7.46 (d, J = 7.8 Hz, 1H), 7.18 (dtd, J = 25.7, 14.0, 12.3, 7.7 Hz, 8H), 7.04 (d, J = 7.8 Hz, 1H), 4.99 (d, J = 16.2 Hz, 1H), 4.88 (d, J = 15.7 Hz, 1H), 4.67 (d, J = 17.2 Hz, 1H), 4.04 (d, J = 17.2 Hz, 1H), 3.81 – 3.62 (m, 1H), 3.40 (dd, J = 11.3, 7.6 Hz, 1H), 3.27 (td, J = 13.3, 7.7 Hz, 1H), 2.89 (ddd, J = 14.9, 11.0, 4.6 Hz, 1H), 2.81 – 2.52 (m, 3H), 2.46 (tt, J = 13.6, 7.2 Hz, 1H), 1.99 (ddtd, J = 29.6, 24.5, 11.7, 5.3 Hz, 2H), 1.31 (s, 9H).

^{13}**C NMR** (101 MHz, $CDCl_3$) δ 174.1, 173.8, 168.0, 141.6, 141.0, 136.3, 134.5, 134.0, 131.2, 129.6, 129.2, 128.5, 128.2, 127.5, 126.5, 125.7, 125.1, 122.3, 121.7, 81.7, 61.0, 58.9, 50.4, 47.3, 37.8, 36.7, 32.1, 28.4, 27.9.

HRMS (ESI, m/z): $[M+H]^+$ Calcd. For $C_{33}H_{38}N_3O_6S$: 604.2481; Found: 604.2479.

HPLC (Chiralpak OD-H Column), i-PrOH/hexane = 10/90, flow rate = 1.0 mL/min, λ = 254 nm; t_R = 22.3 min (major), t_R = 31.4 min (minor).

$[\alpha]_D^{25}$ = –67.6 (c = 0.5, $CHCl_3$).

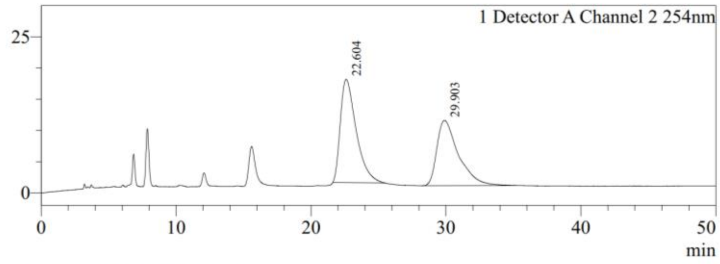

Detector A Channel 2 254nm

Peak#	Ret. Time	USP Width	Area	Height	Area%
1	22.604	2.120	1351560	16515	53.647
2	29.903	2.706	1167804	10456	46.353
Total			2519364	26971	100.000

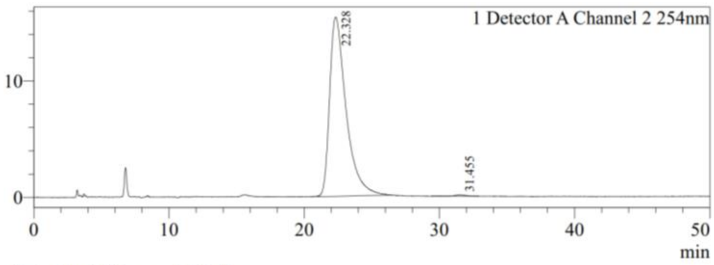

Detector A Channel 2 254nm

Peak#	Ret. Time	USP Width	Area	Height	Area%
1	22.328	2.144	1303854	15400	99.541
2	31.455	1.239	6010	115	0.459
Total			1309865	15514	100.000

Synthesis of (*S*$_{C\text{-}1}$, *R*$_{C\text{-}2}$)-42b

Under N_2 atmosphere, a 10 mL Schlenk tube charged with *α*-amino amide (*S*$_{C\text{-}1}$, *R*$_{C\text{-}2}$)-**41b** (103.7 mg, 0.17 mmol, 1.0 equiv, >99:1 d.r.) and dry ethanol (2.0 mL) was cooled to 0 °C, followed by addition of sodium ethanol (34.7 mg, 0.51 mmol, 3.0 equiv). After addition, the reaction was warmed to room temperature and stirred continually for 2~4 h (monitored by TLC). Once amide was consumed completely, the reaction mixture was quenched with saturated aqueous NH_4Cl and extracted with ethyl acetate. The combined organic layer was washed with brine and concentrated under vacuo. The residue was purified by column chromatography on silica gel (eluent: petroleum ether = 5:1) to give the ester (*S*$_{C\text{-}1}$, *R*$_{C\text{-}2}$)-**42b** as white solid (63.0 mg, 77% yield, >20:1 d.r., signal of diastereomers cannot be observed in ^{1}H NMR).

^{1}H NMR (400 MHz, $CDCl_3$) δ 7.29 (d, *J* = 8.3 Hz, 1H), 7.24 – 7.11 (m, 5H), 7.10 – 7.04 (m, 3H), 4.59 (d, *J* = 17.0 Hz, 1H), 4.27 (d, *J* = 17.0 Hz, 1H), 4.20 – 4.04 (m, 2H), 3.32 – 3.18 (m, 2H), 3.10 (t, *J* = 6.1 Hz, 1H), 2.61 (dtd, *J* = 29.1, 14.4, 7.8 Hz, 4H), 2.36 (tt, *J* = 13.8, 7.4 Hz, 1H), 2.00 (td, *J* = 11.8, 7.6 Hz, 1H), 1.91 – 1.78 (m, 2H), 1.41 (d, *J* = 2.0 Hz, 9H), 1.22 (t, *J* = 7.0 Hz, 3H).

^{13}C NMR (101 MHz, $CDCl_3$) δ 174.1, 174.0, 168.0, 141.7, 141.2, 136.3, 129.5, 128.5, 128.4, 127.8, 126.7, 125.9, 122.2, 82.0, 60.8, 59.3, 56.8, 51.0, 38.0, 34.4, 31.5, 28.6, 28.1, 14.3.

HRMS (ESI, m/z): $[M+H]^+$ Calcd. For $C_{28}H_{37}N_2O_5$: 481.2702; Found: 481.2705.

$[\alpha]_D^{25}$ = −136.8 (c = 0.5, $CHCl_3$).

Synthesis of (*S*$_{C\text{-}1}$, *S*$_{C\text{-}2}$, *S*$_{C\text{-}3}$)-43 & (*S*$_{C\text{-}1}$, *S*$_{C\text{-}2}$, *S*$_{C\text{-}3}$)-44

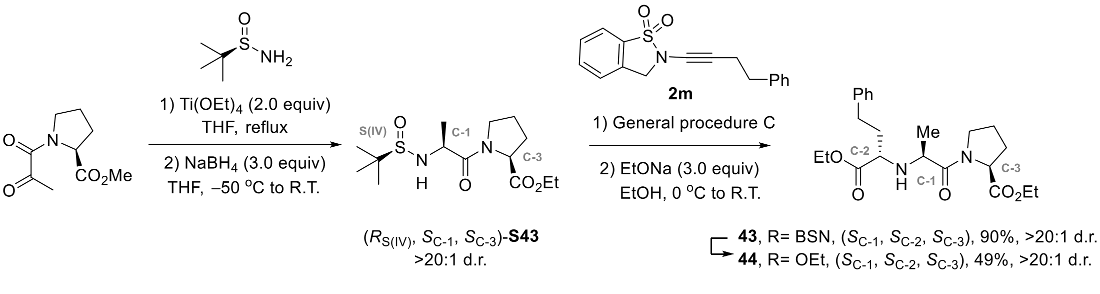

Synthesis of (*R*$_{S(IV)}$, *S*$_{C\text{-}1}$, *S*$_{C\text{-}3}$)-S43

Step 1: Under N_2 atmosphere, the mixture of (*R*)-*tert*-butanesulfinamide (850 mg, 7.0 mmol, 1.0 equiv), methyl (2-oxopropanoyl)-*L*-prolinate (1.40 g, 7.0 mmol, 1.0 equiv) and $Ti(OEt)_4$ (3.21 g, 14.0 mmol, 2.0 equiv) in THF

was refluxed overnight. After completion, the mixture was quenched with ice water and the resulting suspension was filtered and washed with ethyl acetate. The filtrate was collected and extracted with ethyl acetate. The combined organic layer was washed with brine and concentrated under vacuo. The crude product was purified through flash chromatography (eluent: petroleum ether/ethyl acetate = 2:1) to afford imine as light-yellow oil (1.31 g, 62% yield), which was used for the next step.

Step 2: Under N_2 atmosphere, to the solution of imine (1.31 g, 4.14 mmol, 1.0 equiv) in THF (15 mL) was added $NaBH_4$ (470 mg, 12.4 mmol, 3.0 equiv) in batches at –50 °C, and the reaction mixture was stirred at the same temperature over a 3 hours period (monitored by TLC), followed by quenching with MeOH and saturated brine. The resulting suspension was filtered and washed with ethyl acetate. The filtrate was collected and extracted with ethyl acetate. The combined organic layer was washed with brine and concentrated under vacuo. The crude product was purified through flash chromatography (eluent: petroleum ether/ethyl acetate = 1:1) to afforded the primary product ($R_{S(IV)}$, $S_{C\text{-}1}$, $S_{C\text{-}3}$)-**S43** as colorless viscous oil (535 mg, 40% yield, >20:1 d.r., signal of diastereomers cannot be observed in ^{1}H NMR). The absolute configuration of C-1 in **S43** was confirmed by removing its *tert*-butanesulfinyl group to give the corresponding free amines, which was identical to the commercial standard.

^{1}H NMR (400 MHz, $CDCl_3$) δ 4.48 (dd, *J* = 8.8, 4.2 Hz, 1H), 4.17 – 4.10 (m, 4H), 3.70 – 3.60 (m, 1H), 3.60 – 3.52 (m, 1H), 2.26 – 1.87 (m, 4H), 1.44 (d, *J* = 5.4 Hz, 3H), 1.22 (t, *J* = 8.4 Hz, 3H), 1.16 (s, 9H).

^{13}C NMR (101 MHz, $CDCl_3$) δ 171.9, 171.8, 61.2, 59.0, 56.6, 51.3, 46.7, 29.0, 24.9, 22.6, 20.3, 14.2.

HRMS (ESI, m/z): $[M+Na]^+$ Calcd. For $C_{14}H_{26}N_2O_4NaS$: 341.1511; Found: 341.1512.

$[\alpha]_D^{20}$ = –114.6 (c = 1.0, $CHCl_3$).

Synthesis of ($S_{C\text{-}1}$, $S_{C\text{-}2}$, $S_{C\text{-}3}$)-**43**

Prepared according to the **GENERAL PROCEDURE C** with ($R_{S(IV)}$, $S_{C\text{-}1}$, $S_{C\text{-}3}$)-**S43** (91.3 mg, 0.30 mmol, >20:1 d.r.) and **2m** (59.4 mg, 0.20 mmol) as substrates. Column chromatography: silica gel, petroleum ether/ethyl acetate/dichloromethane = 5:8:5. (staining using $KMnO_4$) White solid (95.5 mg, 90% yield, >20:1 d.r., signal of diastereomers cannot be observed in ^{1}H NMR).

^{1}H NMR (400 MHz, $CDCl_3$) δ 7.78 (d, *J* = 7.9 Hz, 1H), 7.68 (t, *J* = 7.6 Hz, 1H), 7.57 (t, *J* = 7.7 Hz, 1H), 7.44 (d, *J* = 8.1 Hz, 1H), 7.25 – 7.10 (m, 5H), 4.94 (d, *J* = 12.0 Hz, 1H), 4.82 (d, *J* = 15.5 Hz, 1H), 4.55 (dd, *J* = 9.0, 3.5 Hz, 1H), 4.20 – 4.03 (m, 3H), 3.67 – 3.45 (m, 3H), 2.90 (td, *J* = 12.0, 5.2 Hz, 1H), 2.70 (td, *J* = 13.4, 12.5, 5.6 Hz, 1H), 2.43 (s, 1H), 2.27 – 2.07 (m, 2H), 2.04 – 1.86 (m, 4H), 1.28 (d, *J* = 6.8 Hz, 3H), 1.21 (t, *J* = 7.2 Hz, 3H).

^{13}C NMR (101 MHz, $CDCl_3$) δ 173.6, 173.5, 172.3, 141.5, 134.24, 134.16, 130.7, 129.7, 128.5, 128.3, 125.9, 125.1, 121.8, 61.0, 58.9, 53.0, 47.5, 46.5, 35.3, 32.2, 28.9, 24.9, 19.1, 14.1.

HRMS (ESI, m/z): $[M+H]^+$ Calcd. For $C_{27}H_{34}N_3O_6S$: 528.2168; Found: 528.2170.

$[\alpha]_D^{25}$ = –56.4° (c = 0.5, $CHCl_3$).

Synthesis of ($S_{C\text{-}1}$, $S_{C\text{-}2}$, $S_{C\text{-}3}$)-**44** (*86*)

Under N_2 atmosphere, a 10 mL Schlenk tube charged with *α*-amino amide ($S_{C\text{-}1}$, $S_{C\text{-}2}$, $S_{C\text{-}3}$)-**43** (95.5 mg, 0.18 mmol, 1.0 equiv, >20:1 d.r.) and dry ethanol (2.0 mL) was cooled to 0 °C, followed by addition of sodium ethanol (36.7

mg, 0.54 mmol, 3.0 equiv). After addition, the reaction was warmed to room temperature and stirred continually for 2~4 h (monitored by TLC). Once amide was consumed completely, the reaction mixture was quenched with saturated aqueous NH_4Cl and extracted with ethyl acetate. The combined organic layer was washed with brine and concentrated under vacuo. The residue was purified by column chromatography on silica gel (eluent: petroleum ether/ethyl acetate/dichloromethane = 3:1:1 to 1:1:1, staining using $KMnO_4$) to give the ester ($S_{C\text{-}1}$, $S_{C\text{-}2}$, $S_{C\text{-}3}$)-**44** as white solid (38.1 mg, 49% yield, >20:1 d.r., signal of diastereomers cannot be observed in ^{1}H NMR).

^{1}H NMR (400 MHz, $CDCl_3$) δ 7.28 – 7.21 (m, 2H), 7.19 – 7.11 (m, 3H), 4.50 (dd, *J* = 8.9, 3.5 Hz, 1H), 4.22 – 4.07 (m, 4H), 3.61 – 3.46 (m, 3H), 3.30 – 3.18 (m, 1H), 2.76 – 2.56 (m, 2H), 2.25 – 1.82 (m, 6H), 1.30 – 1.20 (m, 9H).

^{13}C NMR (101 MHz, $CDCl_3$) δ 174.5, 173.7, 172.1, 141.4, 128.54, 128.51, 128.45, 128.42, 126.0, 61.2, 60.9, 60.1, 59.0, 53.7, 46.6, 35.2, 32.1, 29.0, 25.0, 18.8, 14.4, 14.2.

HRMS (ESI, m/z): $[M+H]^+$ Calcd. For $C_{22}H_{33}N_2O_5$: 405.2389; Found: 405.2390.

$[\alpha]_D^{25}$ = –62.8 (c = 0.5, $CHCl_3$).

8. Crystal Data

X-ray Data for ($S_{C\text{-}1}$, $S_{C\text{-}2}$)-**25a**

The crystal was obtained from the solution of dichloromethane and petroleum ether (1:4).

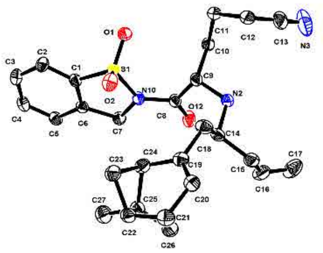

≡

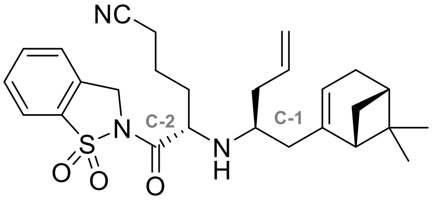

($S_{C\text{-}1}$, $S_{C\text{-}2}$)-**25a**
CCDC: 2349525

ORTEP drawing of ($S_{C\text{-}1}$, $S_{C\text{-}2}$)-**25a** showing thermal ellipsoids at the 40% probability level

Crystal Data and Structure Refinement.

Identification code	test2
Empirical formula	$C_{27}H_{35}N_3O_3S$
Formula weight	481.64
Temperature/K	293(2)
Crystal system	monoclinic
Space group	$P2_1$
a/Å	18.3543(9)
b/Å	9.9990(5)
c/Å	28.5939(13)
α/°	90
β/°	90.968(2)
γ/°	90
Volume/Å^3	5246.9(4)
Z	8
ρ_{calc}g/cm^3	1.219
μ/mm^{-1}	0.156
F(000)	2064.0
Crystal size/mm^3	undefined
Radiation	MoKα (λ = 0.71073)

2Θ range for data collection/°	4.274 to 50.13
Index ranges	-21 ≤ h ≤ 21, -11 ≤ k ≤ 11, -34 ≤ l ≤ 34
Reflections collected	76020
Independent reflections	18488 [R_{int} = 0.0470, R_{sigma} = 0.0408]
Data/restraints/parameters	18488/1/1257
Goodness-of-fit on F^2	1.042
Final R indexes [I>=2σ (I)]	R_1 = 0.0335, wR_2 = 0.0828
Final R indexes [all data]	R_1 = 0.0382, wR_2 = 0.0865
Largest diff. peak/hole / e $Å^{-3}$	0.40/-0.20
Flack parameter	0.040(17)

X-ray Data for ($R_{C\text{-}1}$, $R_{C\text{-}2}$)**-25b**

The crystal was obtained from the solution of dichloromethane and petroleum ether (1:4).

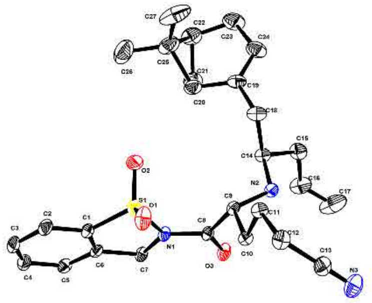

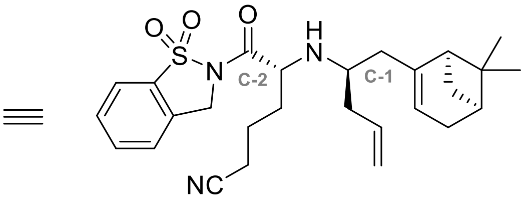

(*R*C-1, *R*C-2)-**25b**
CCDC: 2349519

ORTEP drawing of ($R_{C\text{-}1}$, $R_{C\text{-}2}$)-**25b** showing thermal ellipsoids at the 40% probability level

Crystal Data and Structure Refinement.

Identification code	2023122501_0m_a
Empirical formula	$C_{27}H_{35}N_3O_3S$
Formula weight	481.64
Temperature/K	150.15
Crystal system	orthorhombic
Space group	$P2_12_12_1$
a/Å	6.909(2)

b/Å	9.969(3)
c/Å	39.178(12)
α/°	90
β/°	90
γ/°	90
Volume/Å^3	2698.3(15)
Z	4
ρ_{calc}g/cm^3	1.186
μ/mm^{-1}	0.151
F(000)	1032.0
Crystal size/mm^3	0.24 × 0.21 × 0.17
Radiation	MoKα (λ = 0.71073)
2Θ range for data collection/°	4.216 to 59.18
Index ranges	$-9 \leq h \leq 9$, $-13 \leq k \leq 13$, $-54 \leq l \leq 53$
Reflections collected	29500
Independent reflections	7227 [R_{int} = 0.0417, R_{sigma} = 0.0376]
Data/restraints/parameters	7227/2/320
Goodness-of-fit on F^2	1.053
Final R indexes [I>=2σ (I)]	R_1 = 0.0367, wR_2 = 0.0983
Final R indexes [all data]	R_1 = 0.0401, wR_2 = 0.1008
Largest diff. peak/hole / e Å^{-3}	0.27/-0.29
Flack parameter	0.04(2)

9. Spectra

¹H NMR (400 MHz, $CDCl_3$) – **2e**

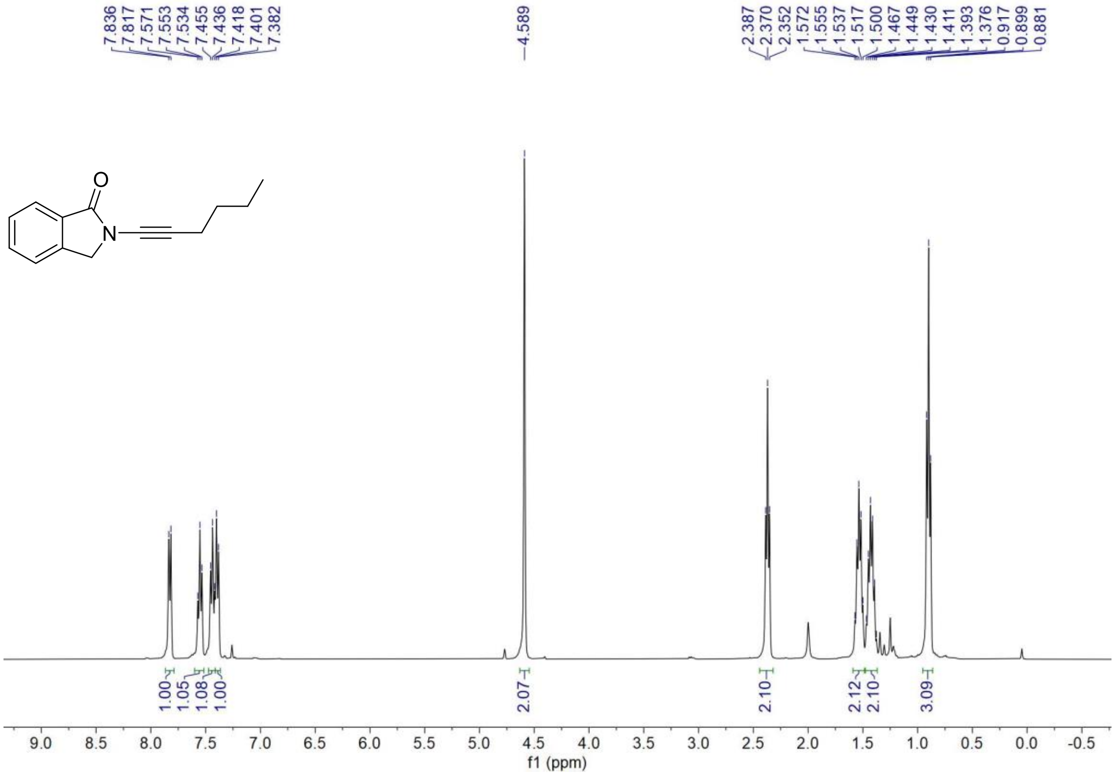

¹³C NMR (101 MHz, $CDCl_3$) – **2e**

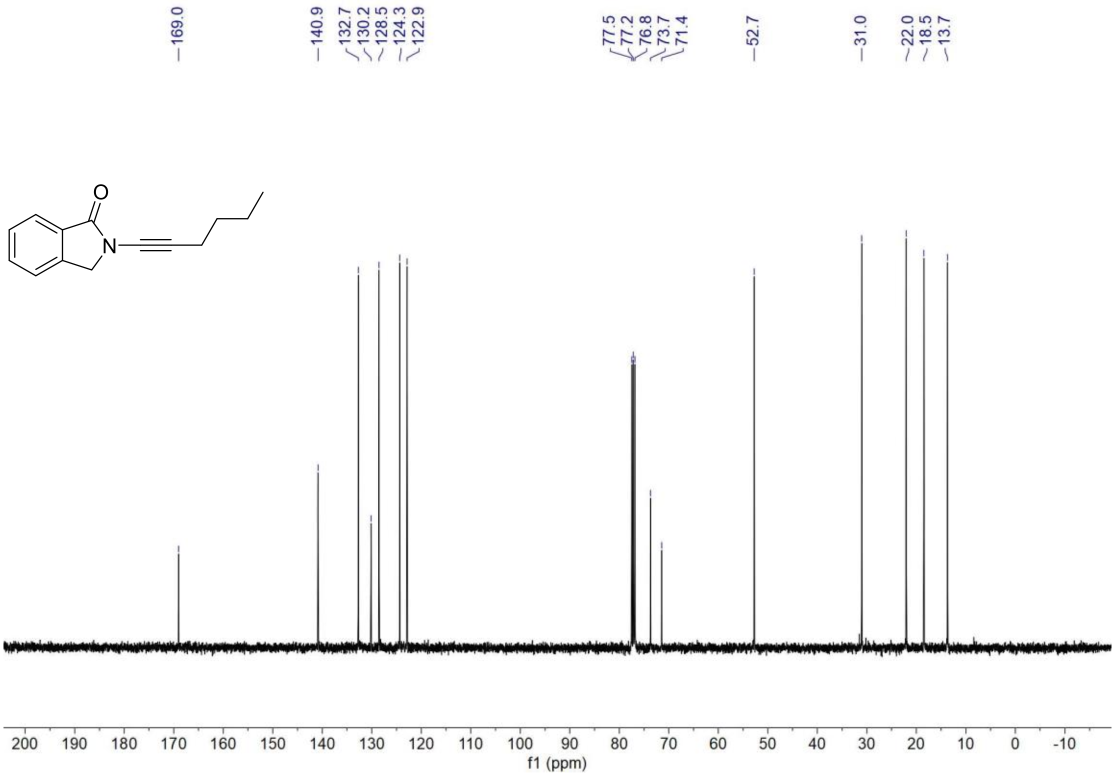

^{1}H NMR (400 MHz, $CDCl_3$) – **2m**

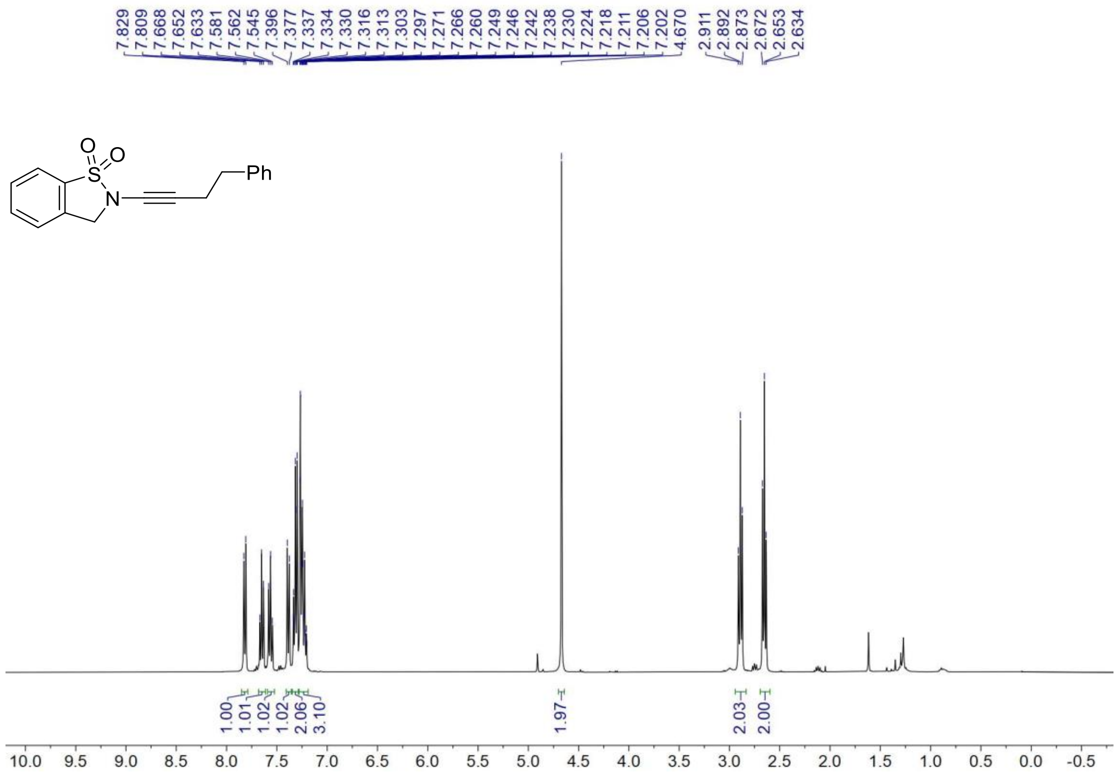

^{13}C NMR (101 MHz, $CDCl_3$) – **2m**

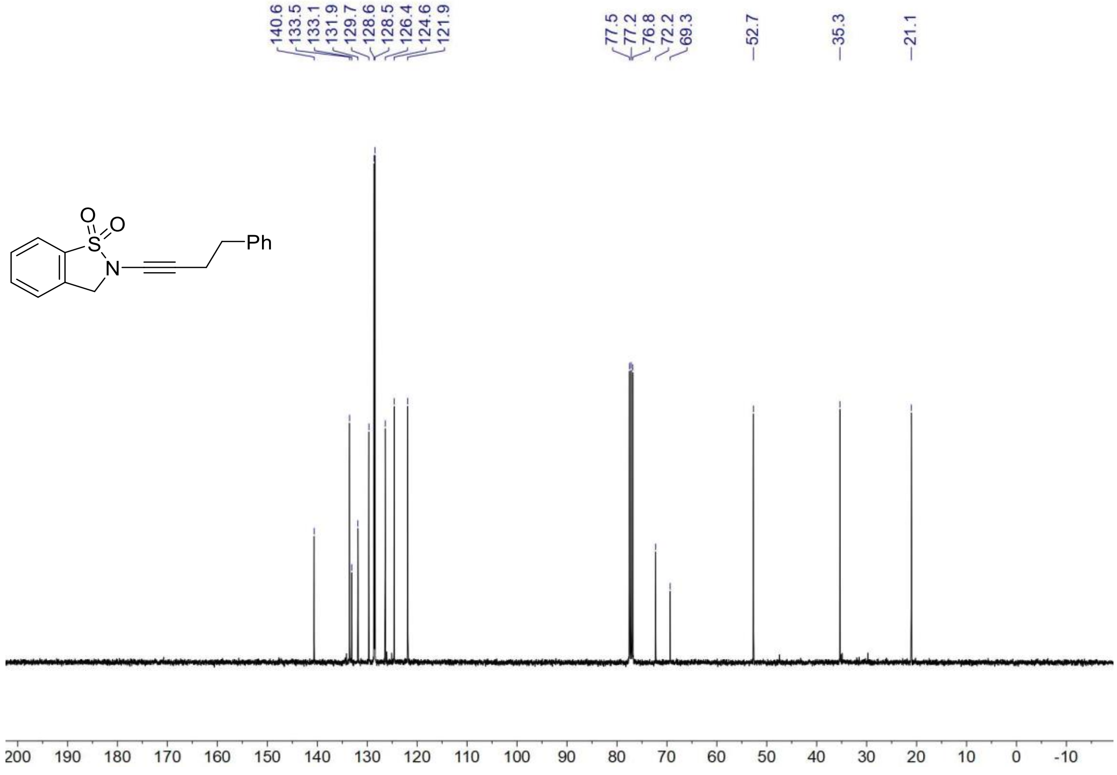

^{1}H NMR (400 MHz, $CDCl_3$) – ($S_{S(IV)}$, $R_{C\text{-}1}$) **-S4a**

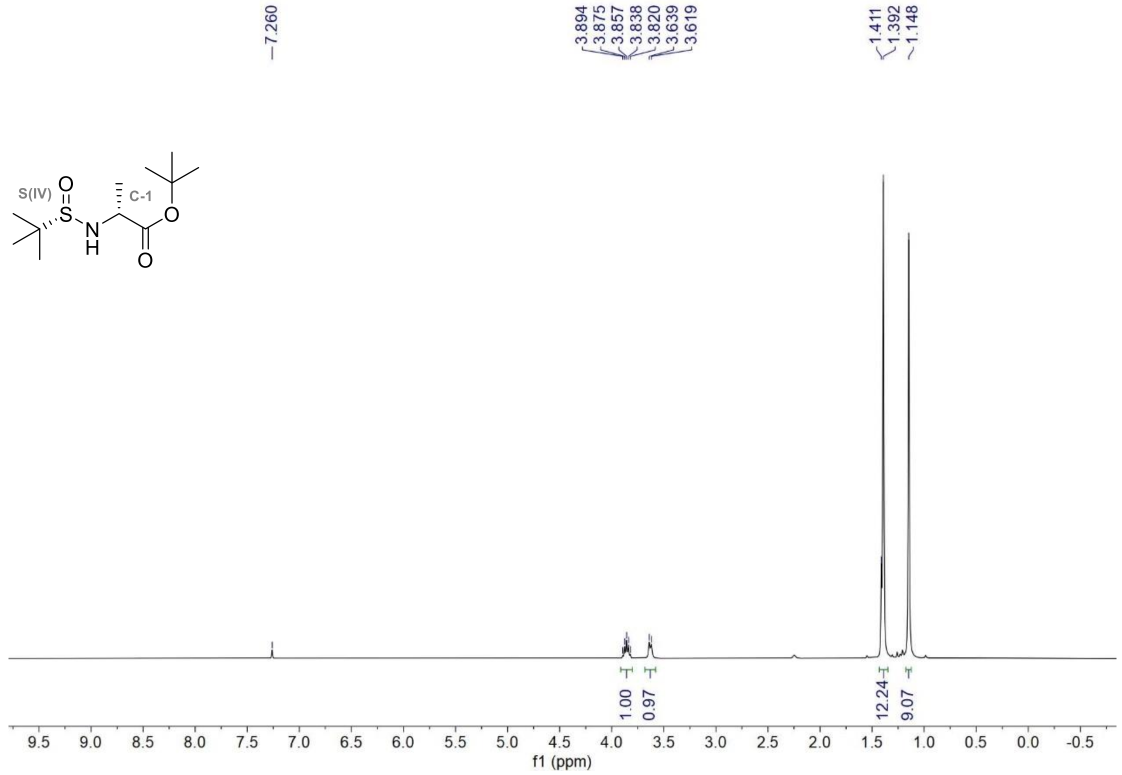

^{13}C NMR (101 MHz, $CDCl_3$) – ($S_{S(IV)}$, $R_{C\text{-}1}$) **-S4a**

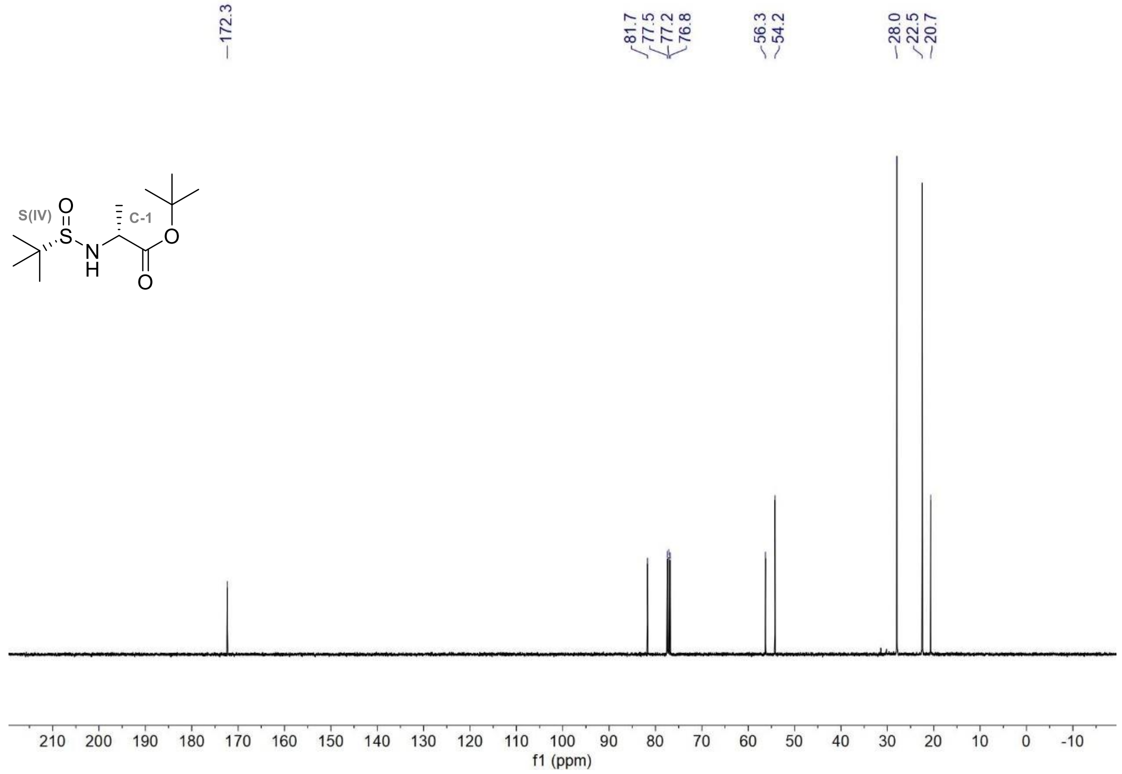

^{1}H NMR (400 MHz, $CDCl_3$) – ($R_{C\text{-}1}$, $R_{C\text{-}2}$)-**4a**

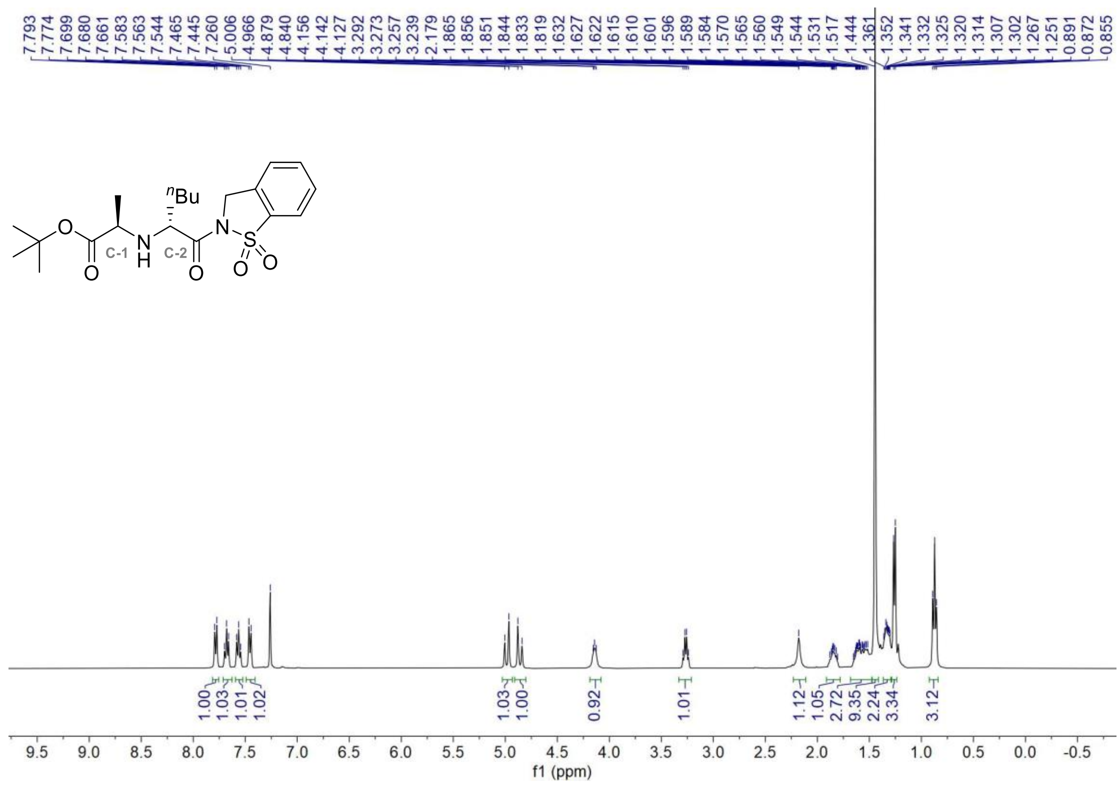

^{13}C NMR (101 MHz, $CDCl_3$) - ($R_{C\text{-}1}$, $R_{C\text{-}2}$)-**4a**

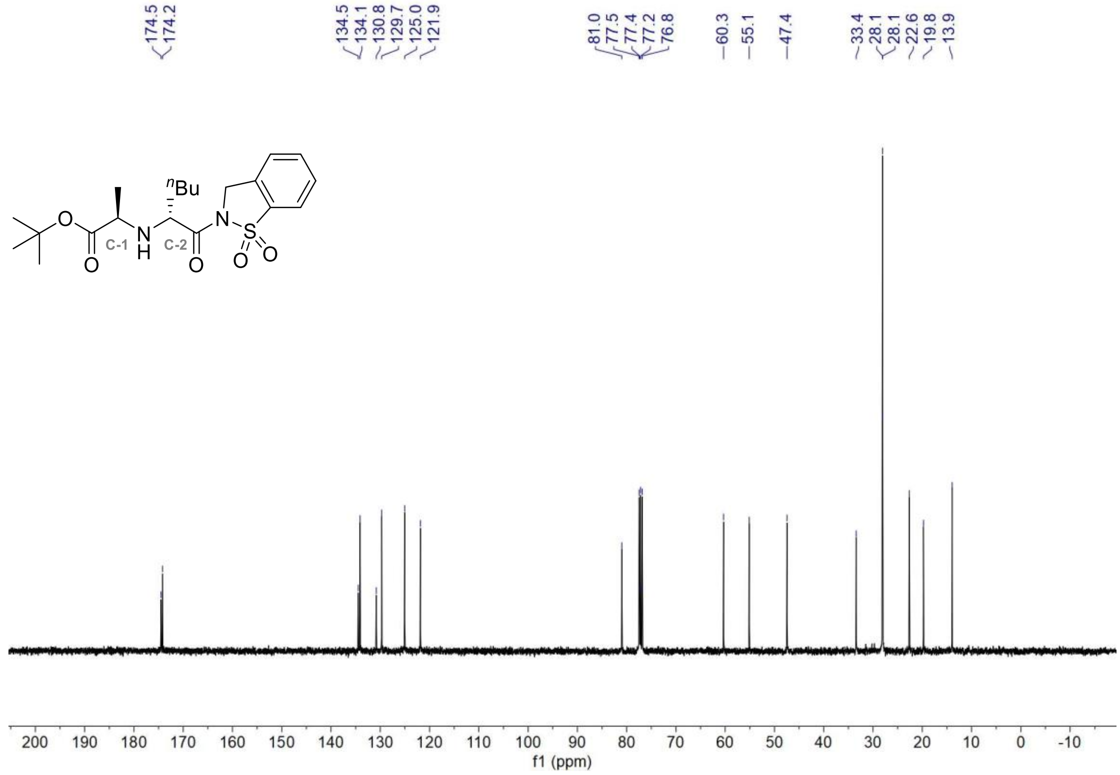

^{1}H NMR (400 MHz, $CDCl_3$) – ($S_{S(IV)}$, $S_{C\text{-}1}$)-**S4b**

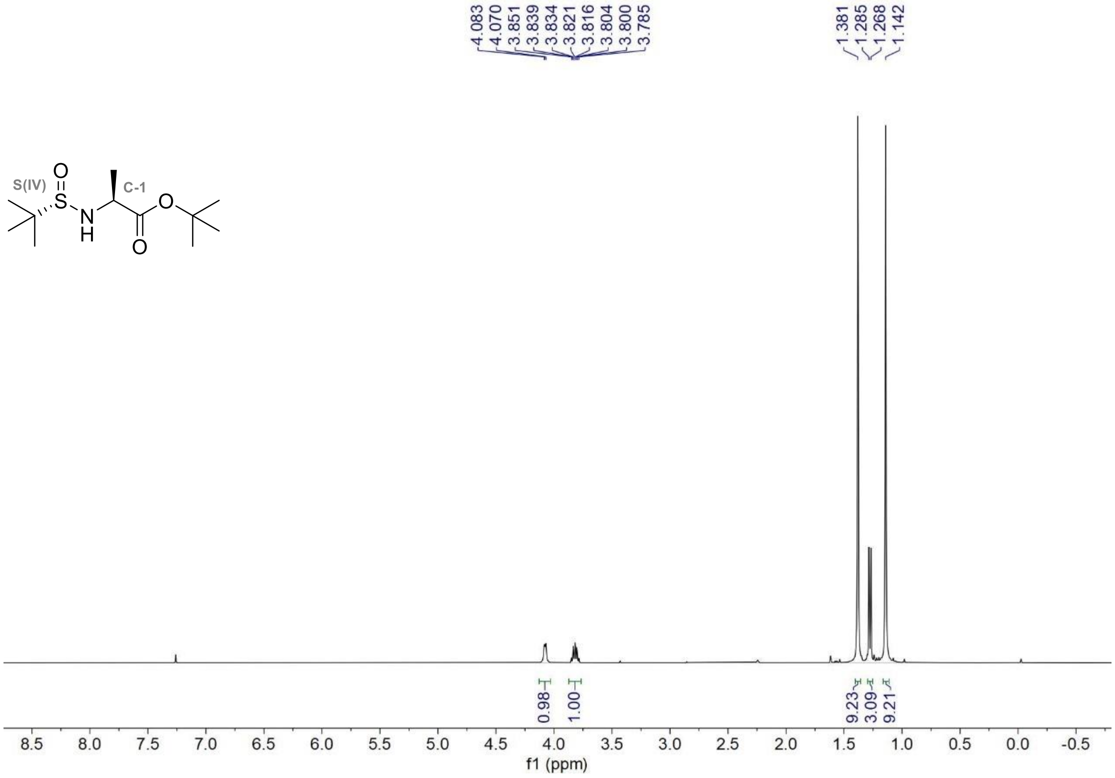

^{13}C NMR (101 MHz, $CDCl_3$) – ($S_{S(IV)}$, $S_{C\text{-}1}$)-**S4b**

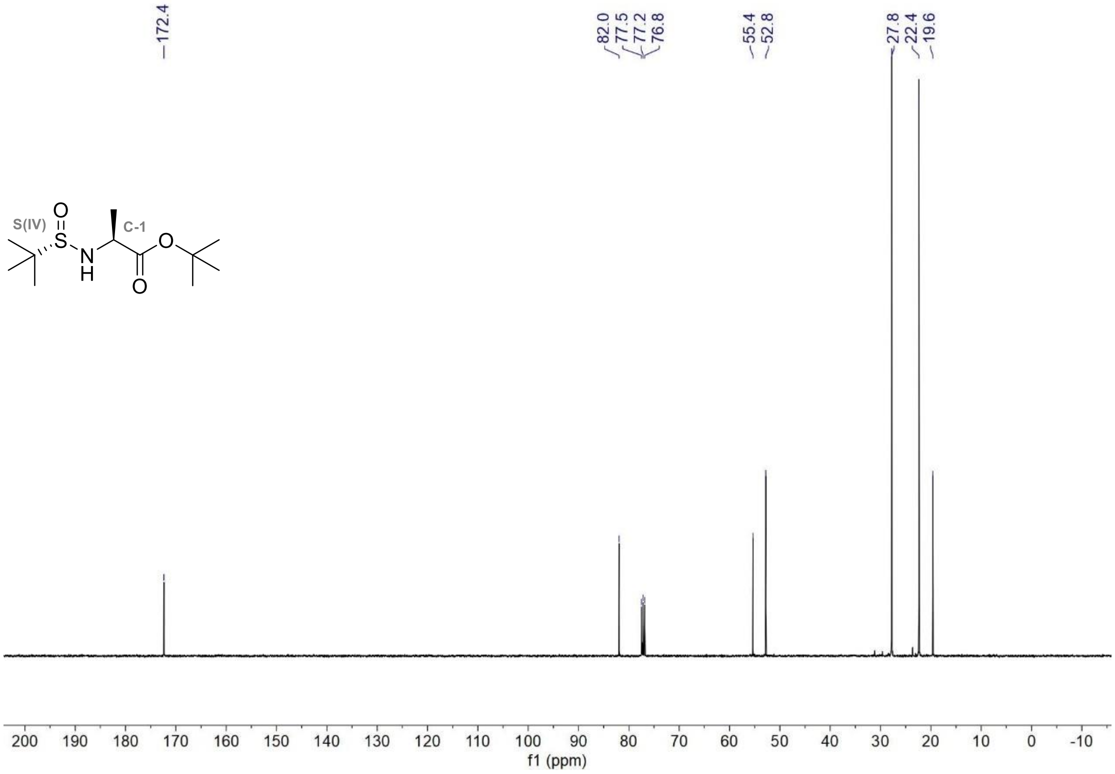

^{1}H NMR (400 MHz, $CDCl_3$) – ($S_{C\text{-}1}$, $R_{C\text{-}2}$)-**4b**

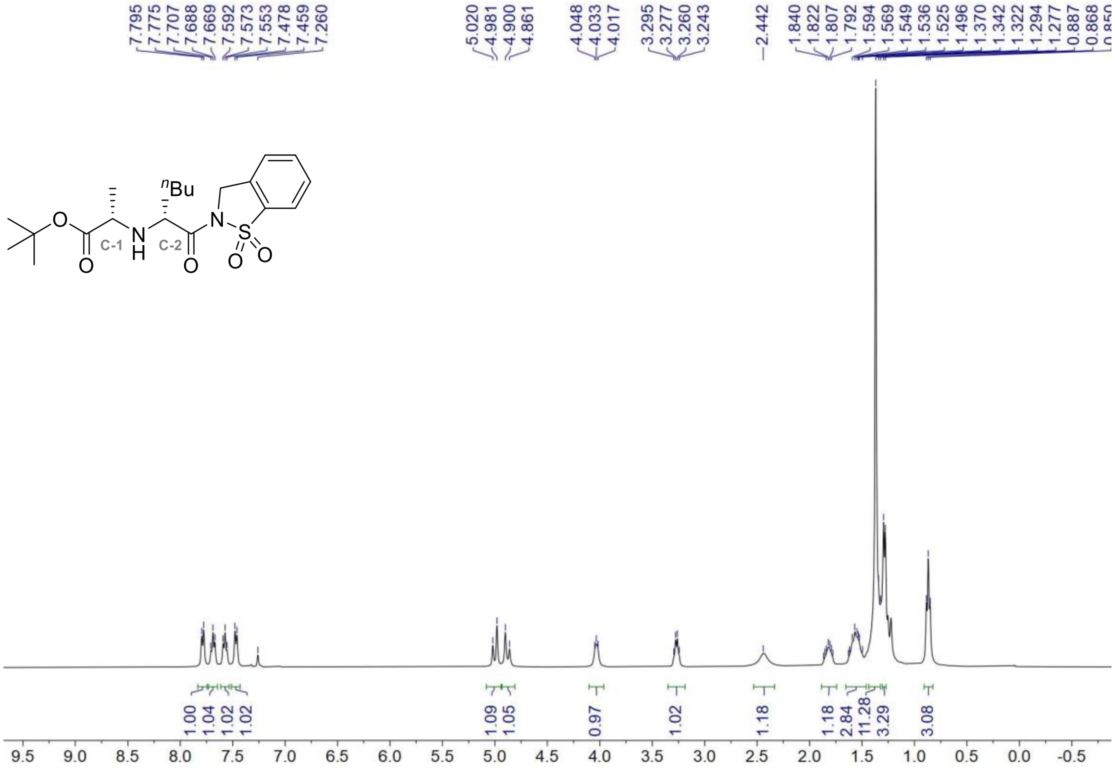

^{13}C NMR (101 MHz, $CDCl_3$) – ($S_{C\text{-}1}$, $R_{C\text{-}2}$)-**4b**

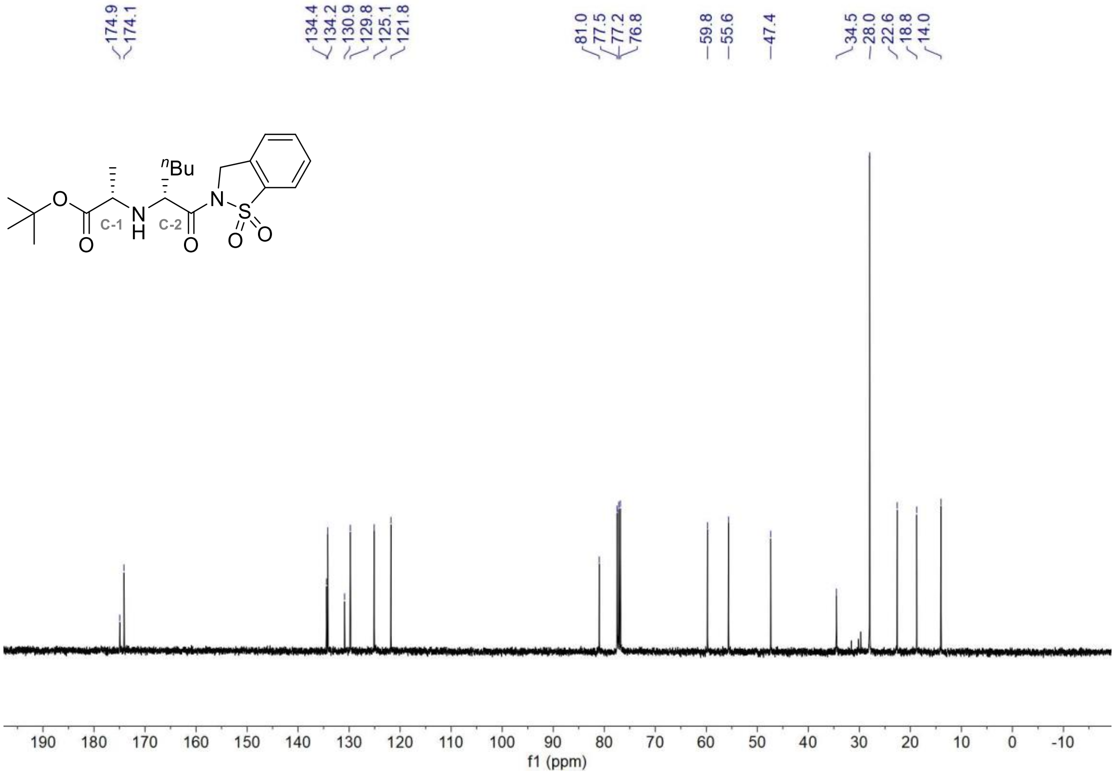

^{1}H NMR (400 MHz, $CDCl_3$) – ($R_{S(IV)}$, R_{C-1})-**S4c**

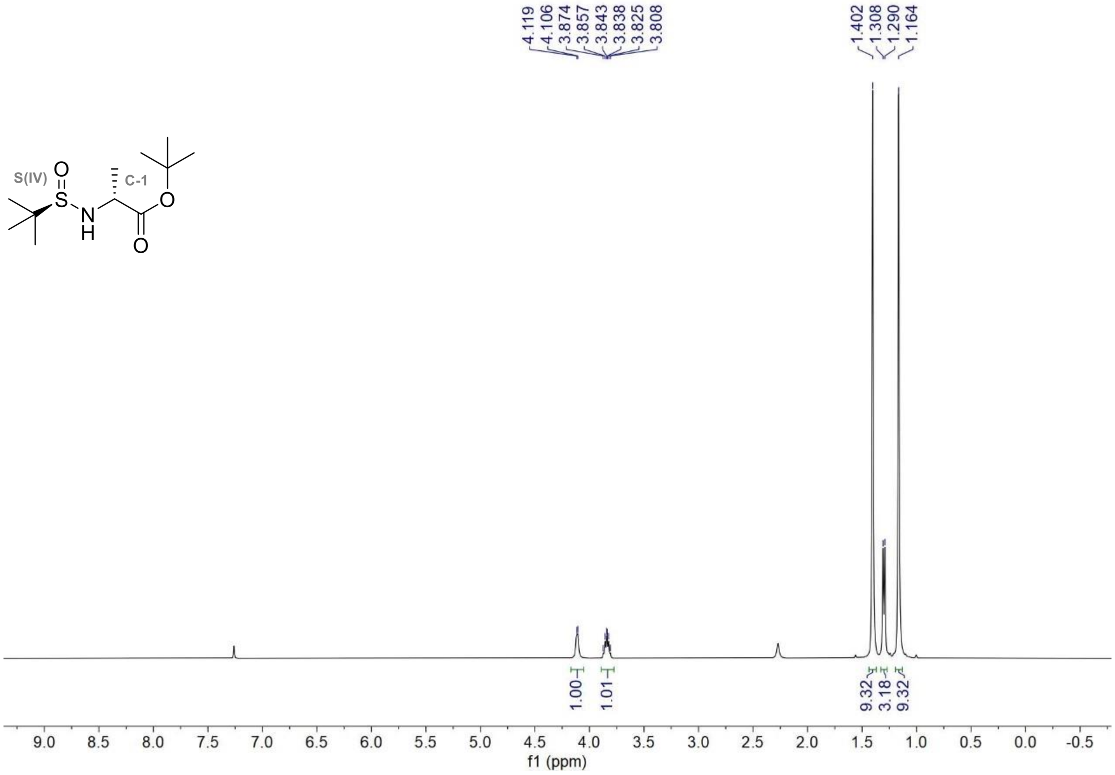

^{13}C NMR (101 MHz, $CDCl_3$) – ($R_{S(IV)}$, R_{C-1})-**S4c**

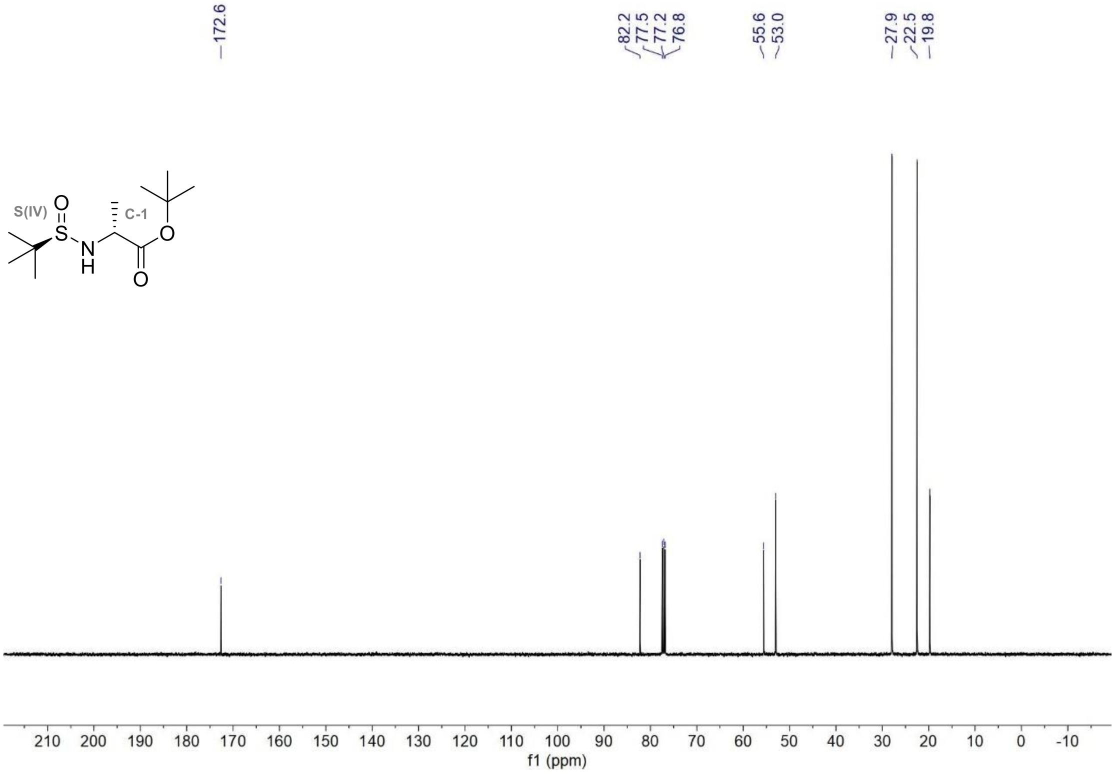

^{1}H NMR (400 MHz, $CDCl_3$) – ($R_{C\text{-}1}$, $S_{C\text{-}2}$)-**4c**

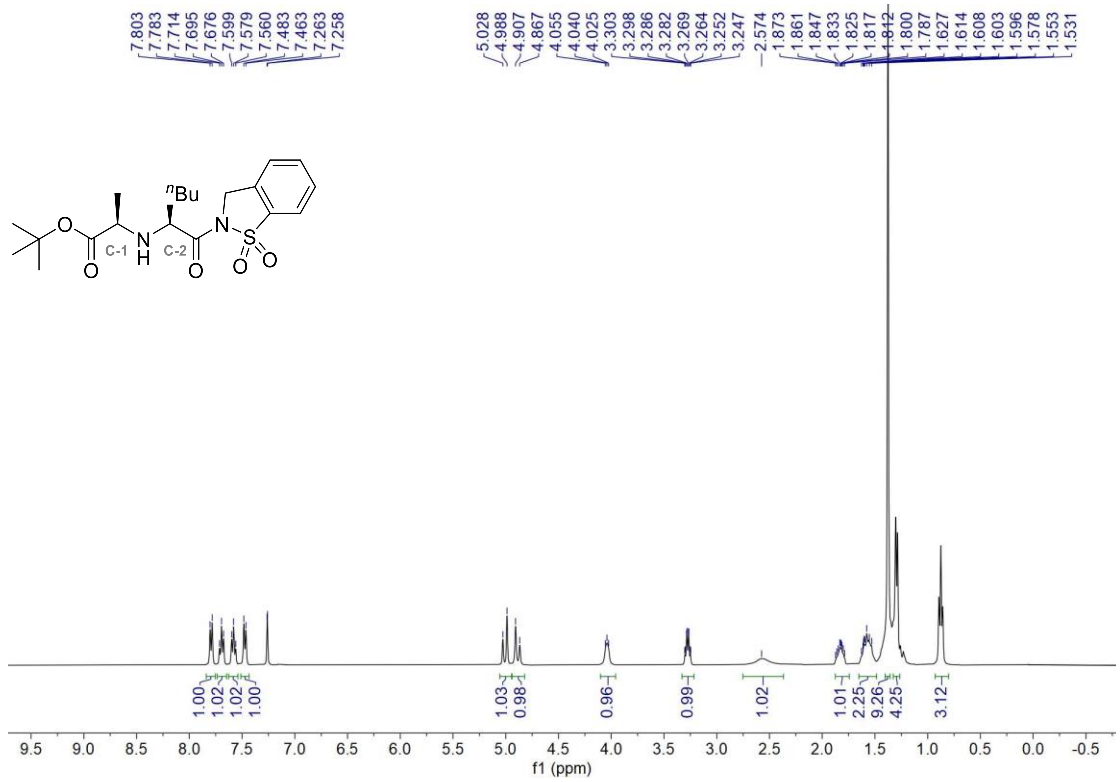

^{13}C NMR (101 MHz, $CDCl_3$) – ($R_{C\text{-}1}$, $S_{C\text{-}2}$)-**4c**

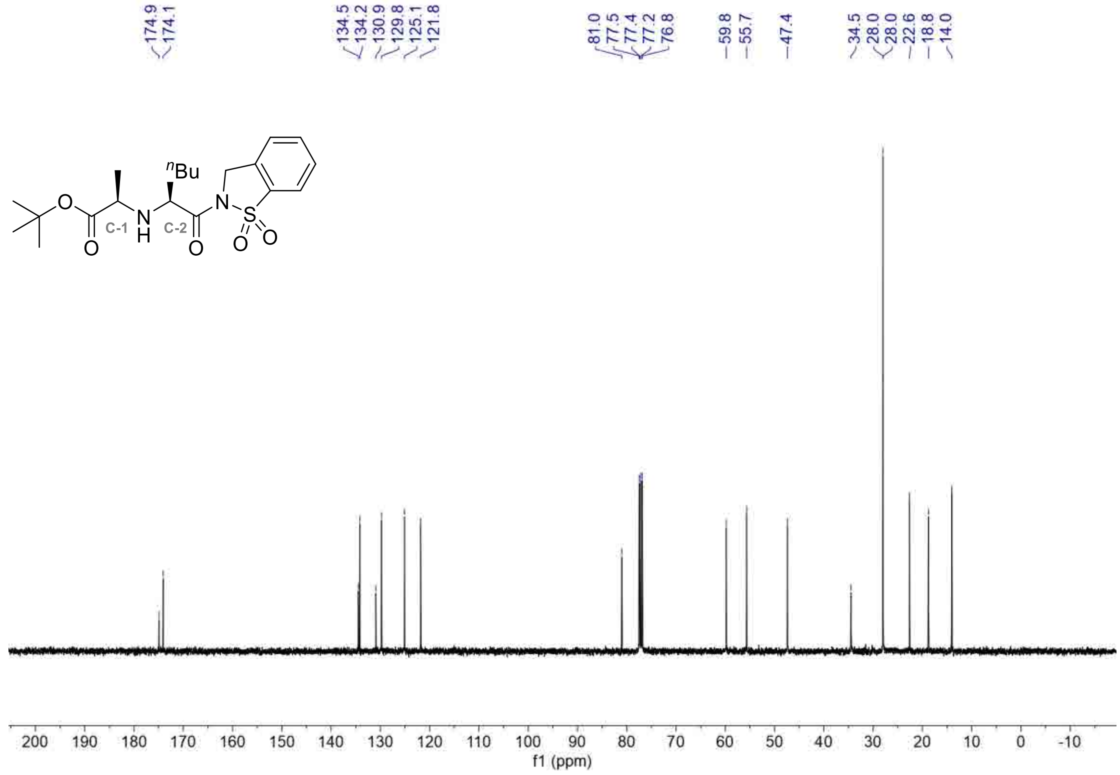

^{1}H NMR (400 MHz, $CDCl_3$) – ($R_{S(IV)}$, $S_{C\text{-}1}$)-**S4d**

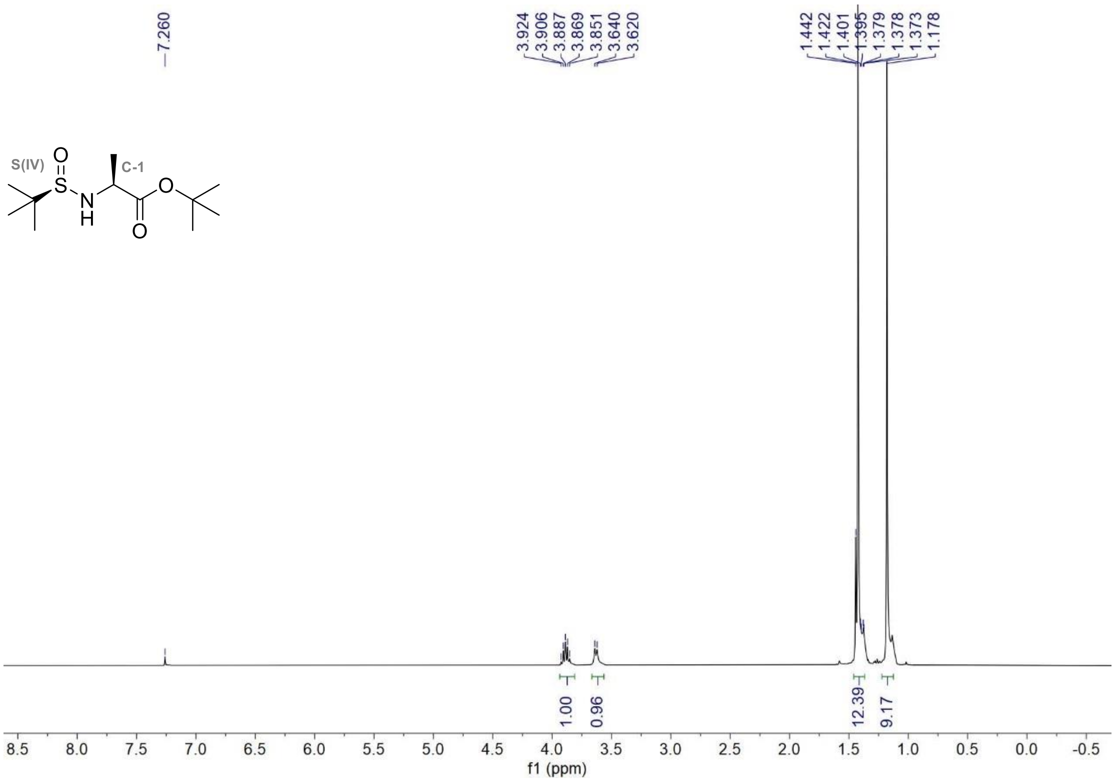

^{13}C NMR (101 MHz, $CDCl_3$) – ($R_{S(IV)}$, $S_{C\text{-}1}$)-**S4d**

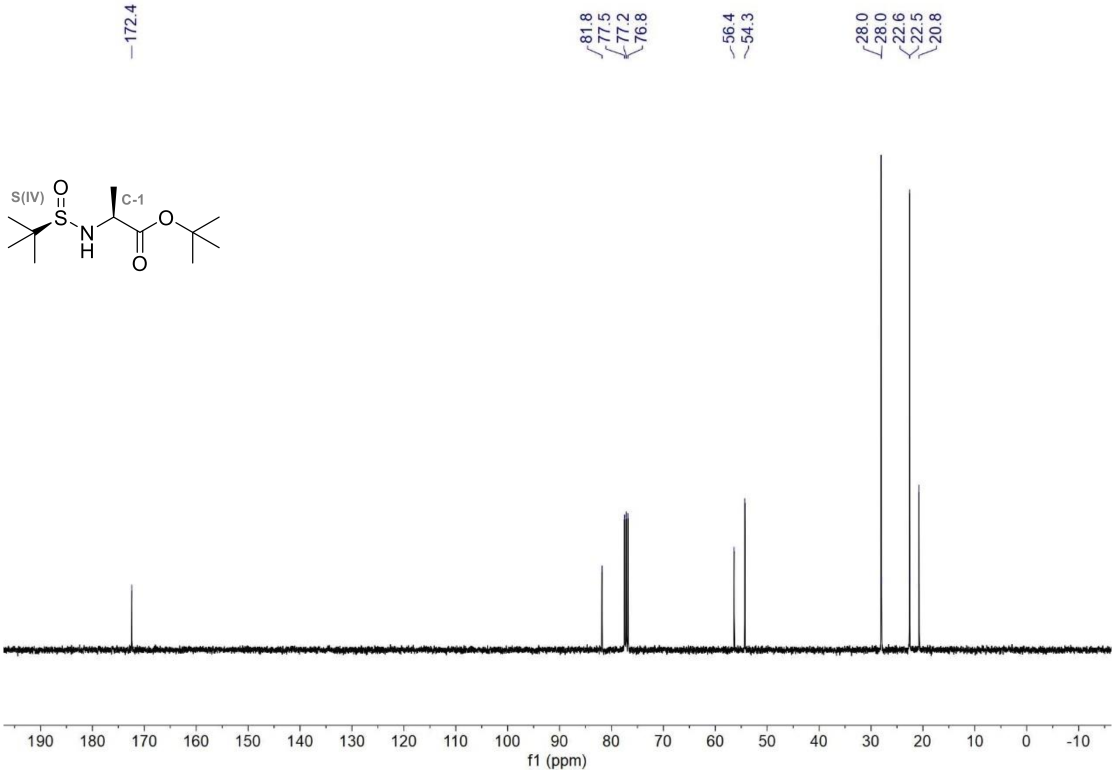

^{1}H NMR (400 MHz, $CDCl_3$) – (S_{C-1}, S_{C-2})-**4d**

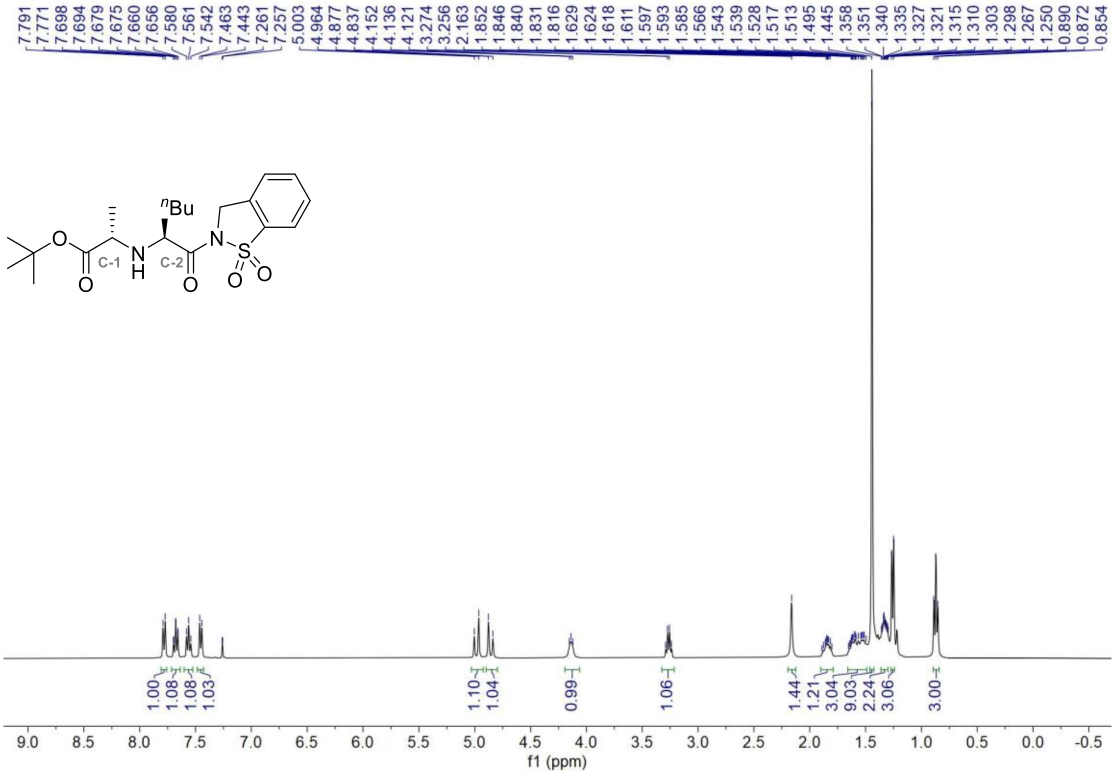

^{13}C NMR (101 MHz, $CDCl_3$) – (S_{C-1}, S_{C-2})-**4d**

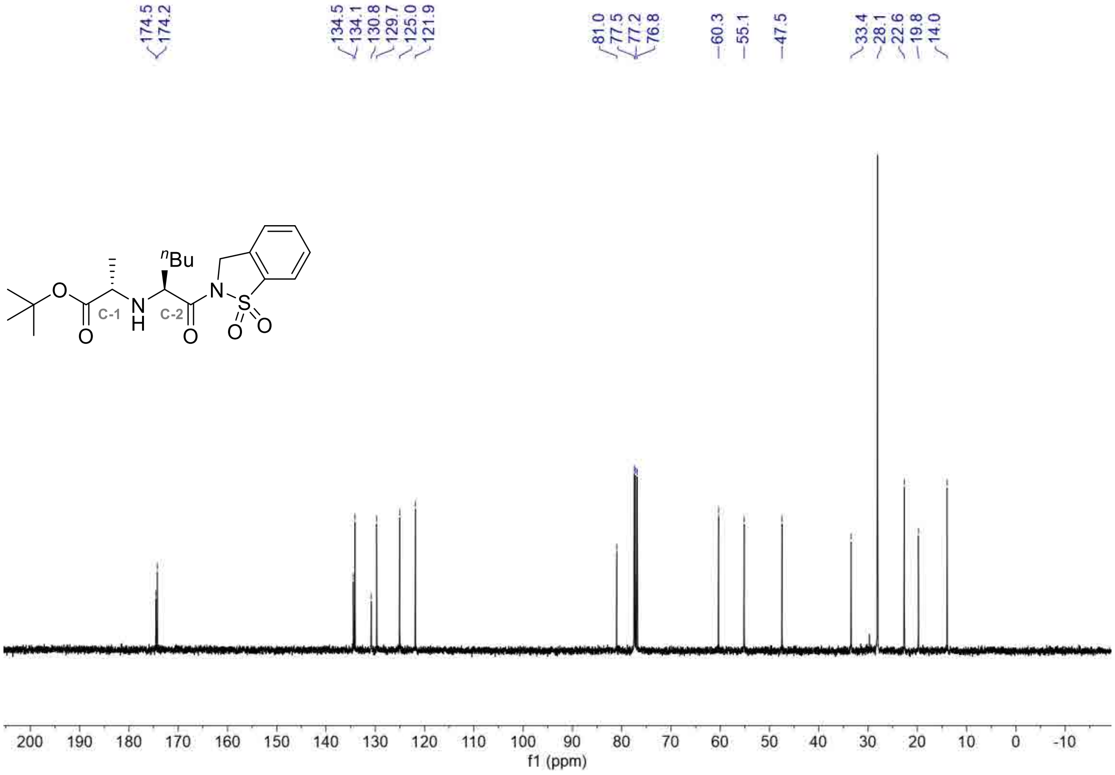

^{1}H NMR (400 MHz, $CDCl_3$) – ($R_{C\text{-}1}$, $S_{C\text{-}2}$)-**5a**

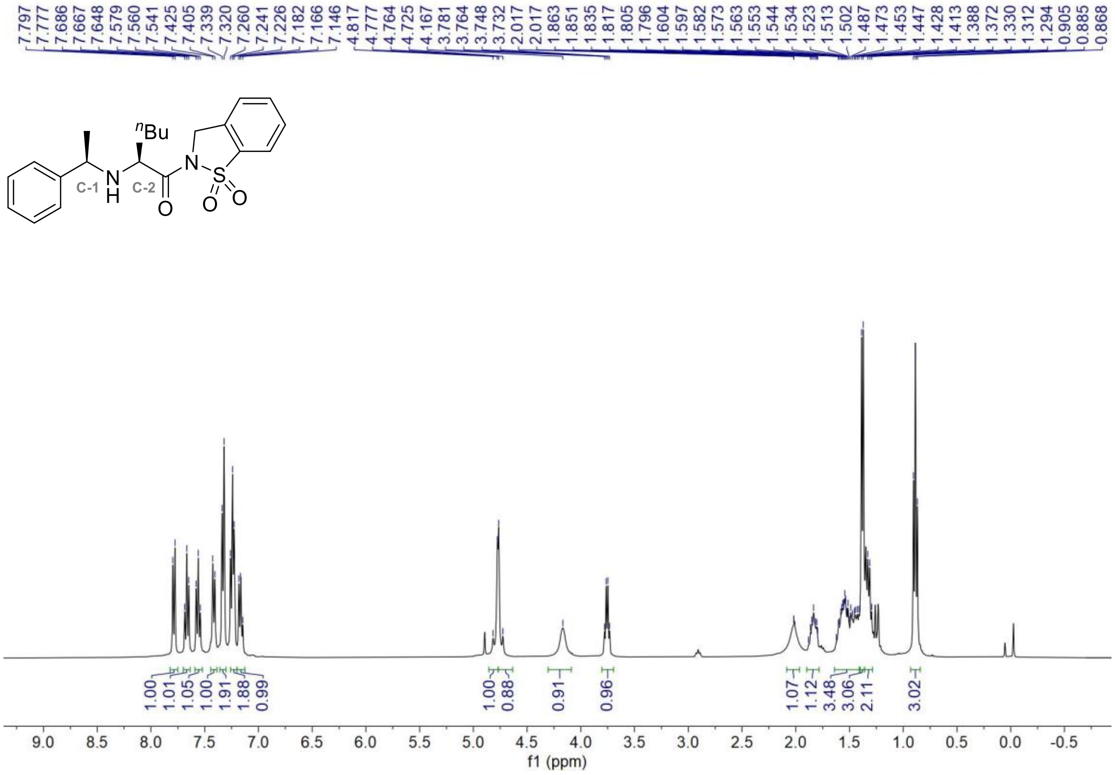

^{13}C NMR (101 MHz, $CDCl_3$) – ($R_{C\text{-}1}$, $S_{C\text{-}2}$)-**5a**

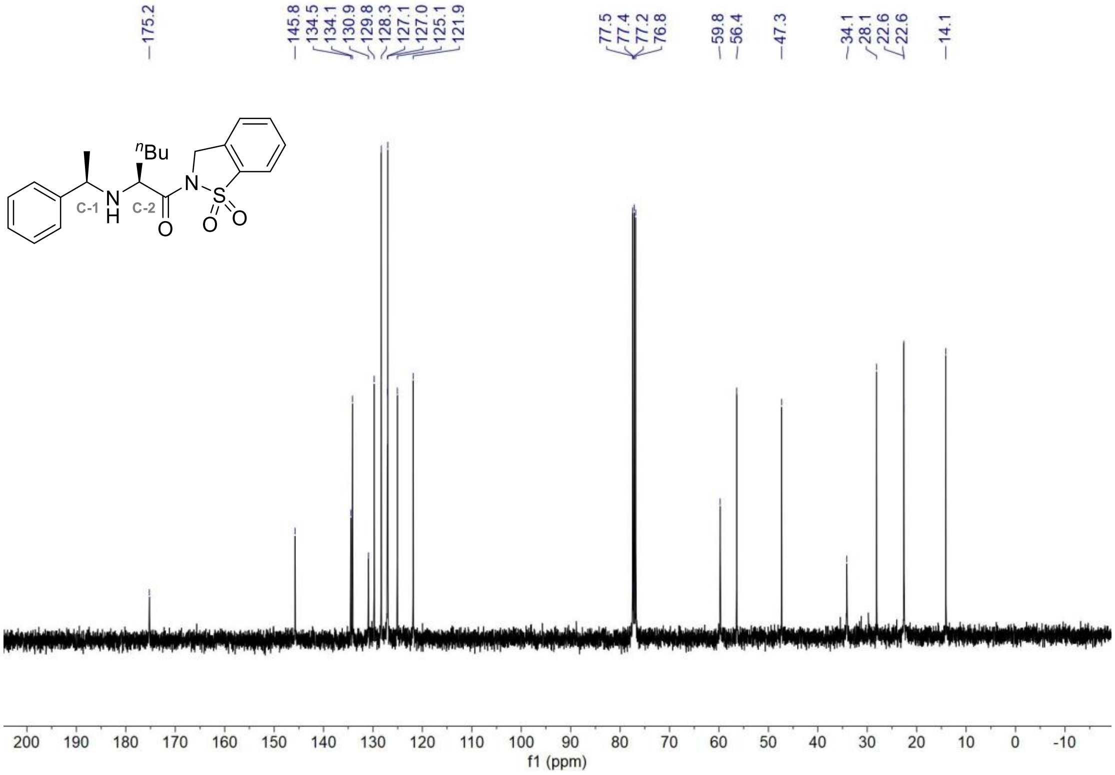

^{1}H NMR (400 MHz, $CDCl_3$) – ($S_{S(IV)}$, $R_{C\text{-}1}$)-**S5b**

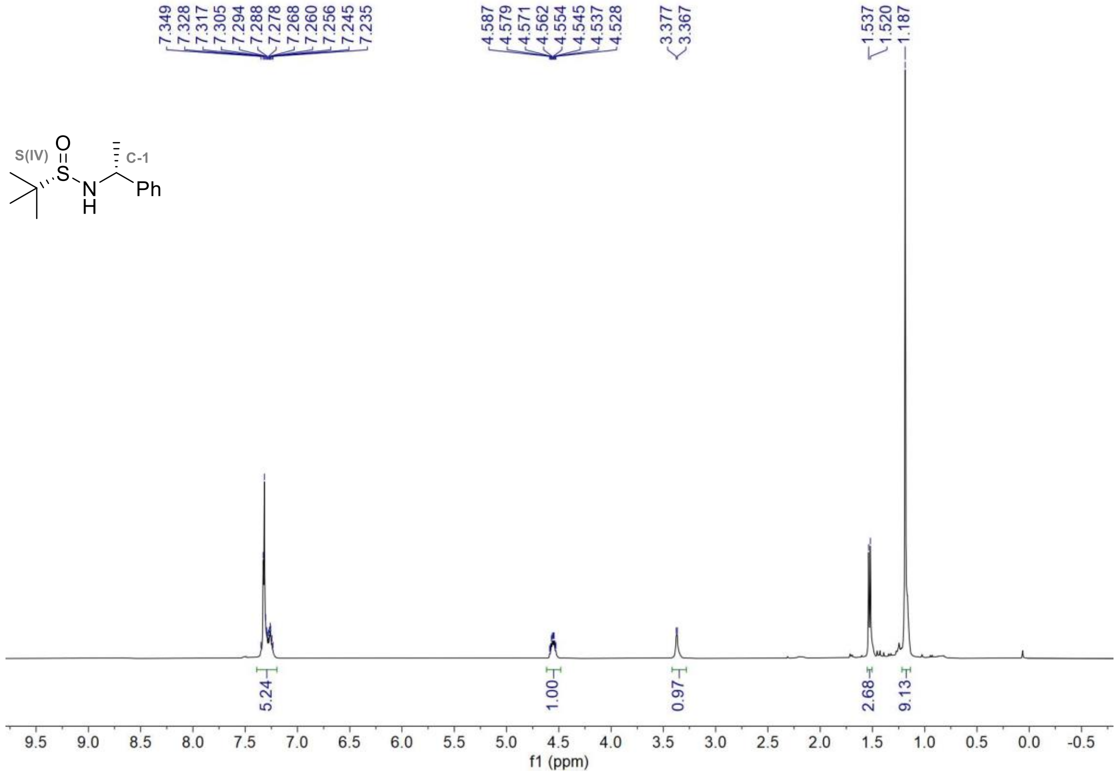

^{13}C NMR (101 MHz, $CDCl_3$) – ($S_{S(IV)}$, $R_{C\text{-}1}$)-**S5b**

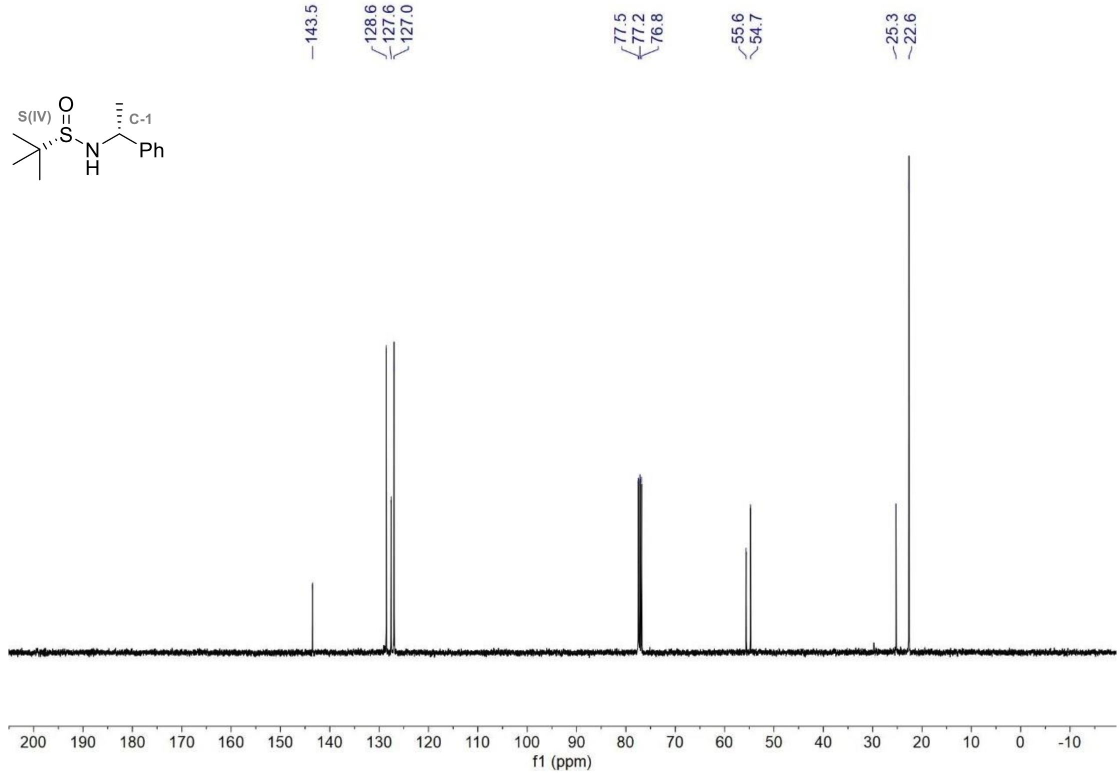

^{1}H NMR (400 MHz, $CDCl_3$) – ($R_{C\text{-}1}$, $R_{C\text{-}2}$)-**5b**

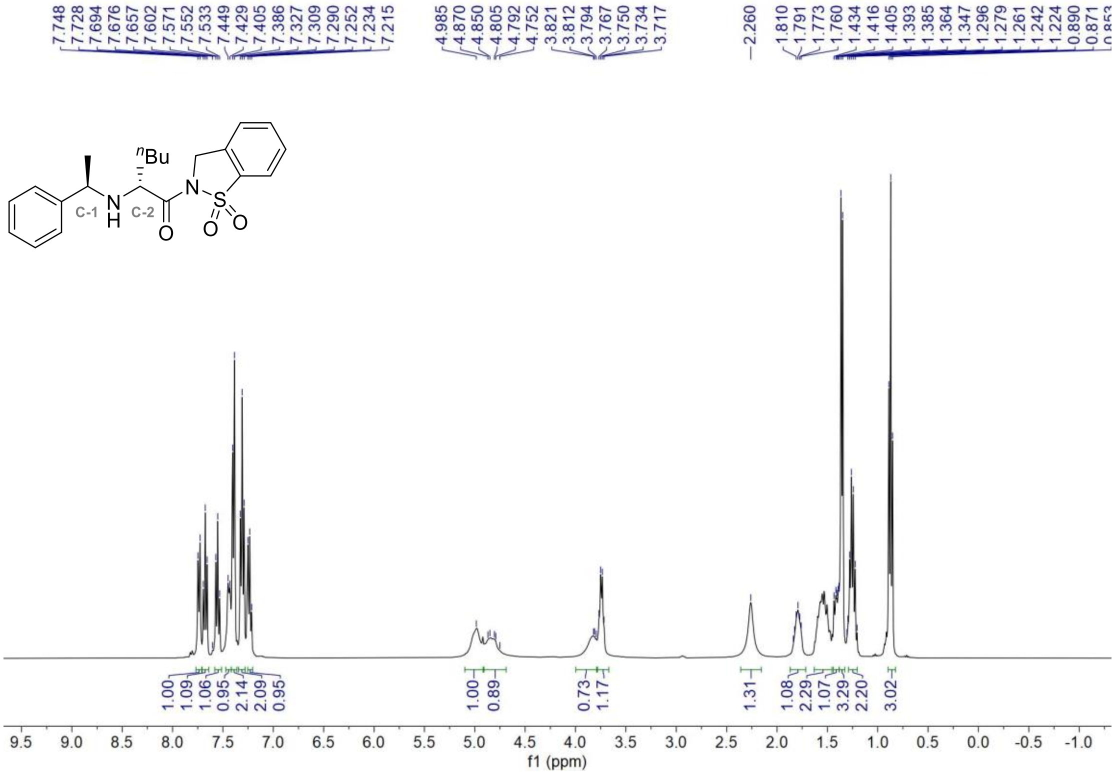

^{13}C NMR (101 MHz, $CDCl_3$) – ($R_{C\text{-}1}$, $R_{C\text{-}2}$)-**5b**

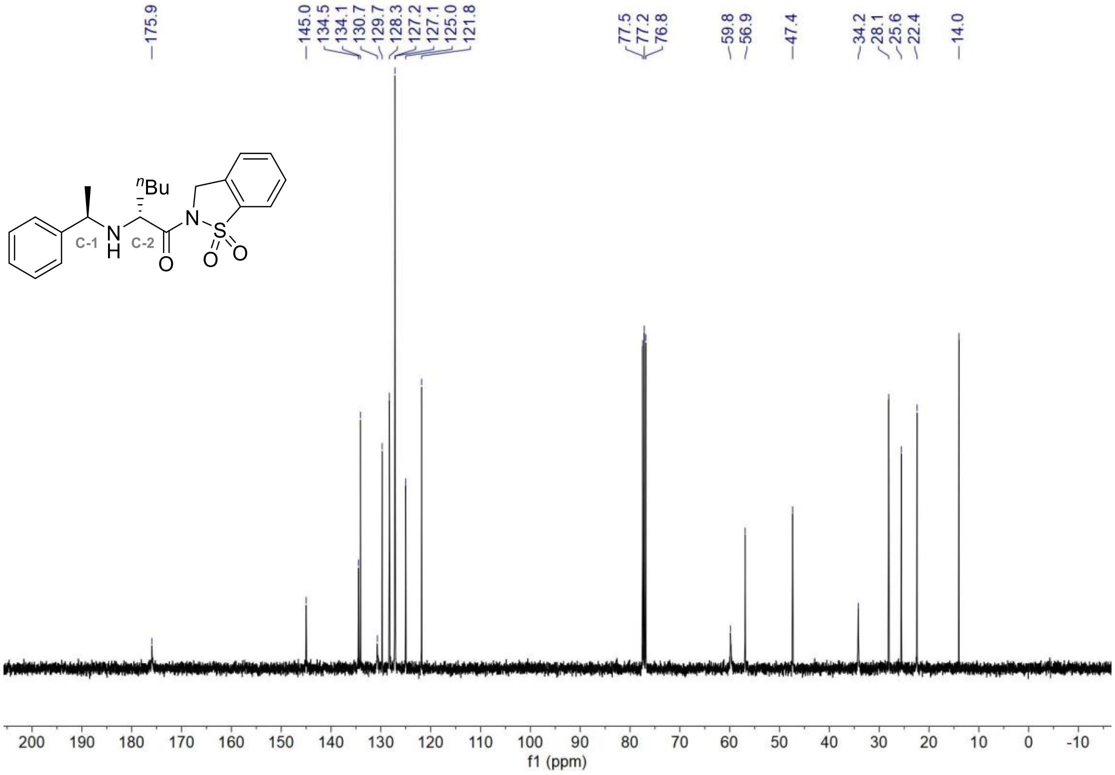

^{1}H NMR (400 MHz, $CDCl_3$) – (S_{C-1}, S_{C-2})-**6a**

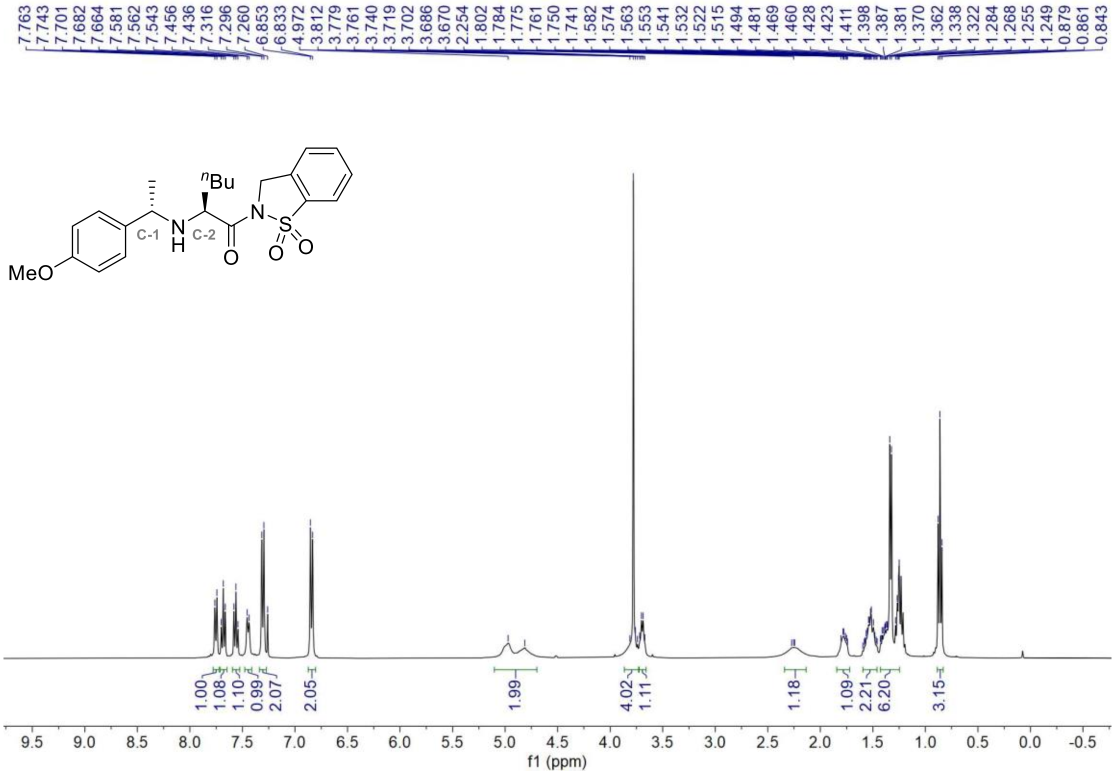

^{13}C NMR (101 MHz, $CDCl_3$) – (S_{C-1}, S_{C-2})-**6a**

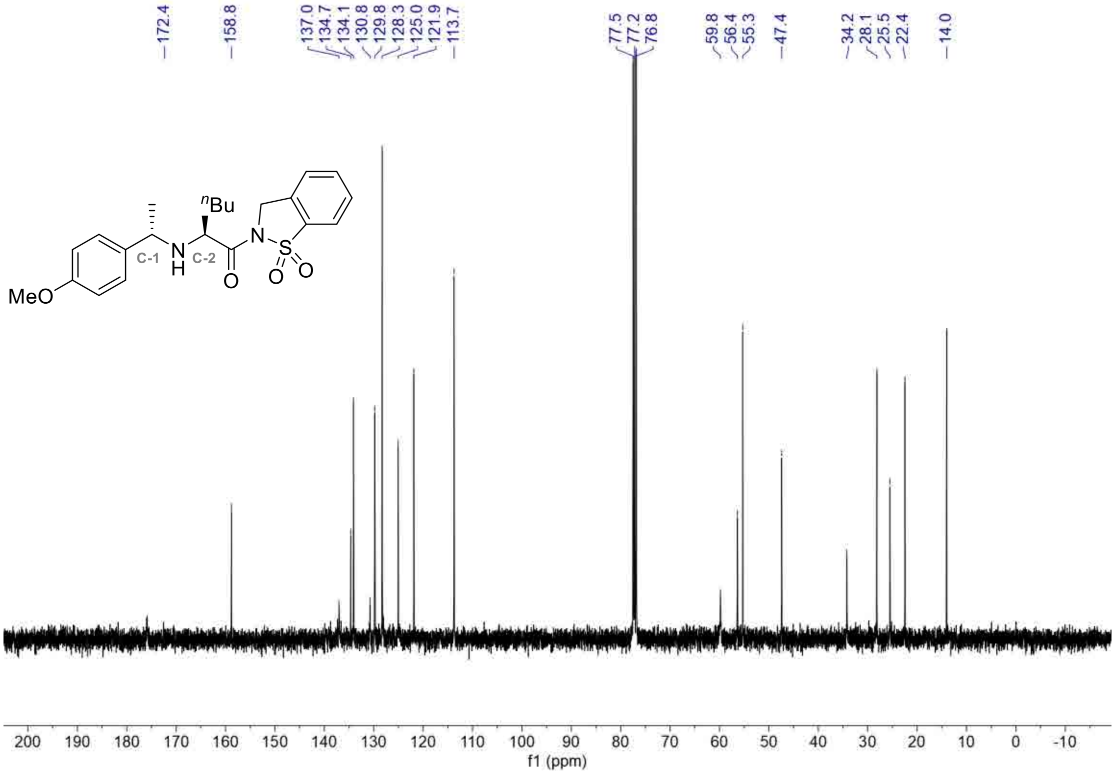

[1]**H NMR** (400 MHz, $CDCl_3$) – ($S_{C\text{-}1}$, $R_{C\text{-}2}$)-**6b**

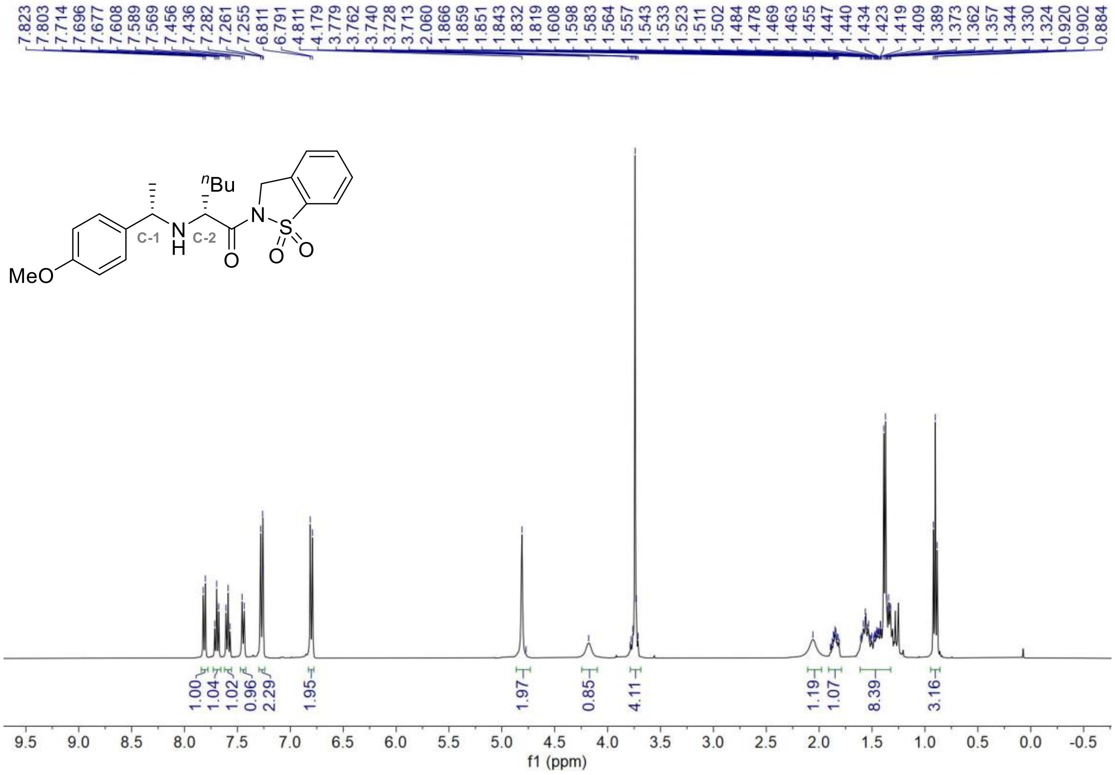

[13]**C NMR** (101 MHz, $CDCl_3$) – ($S_{C\text{-}1}$, $R_{C\text{-}2}$)-**6b**

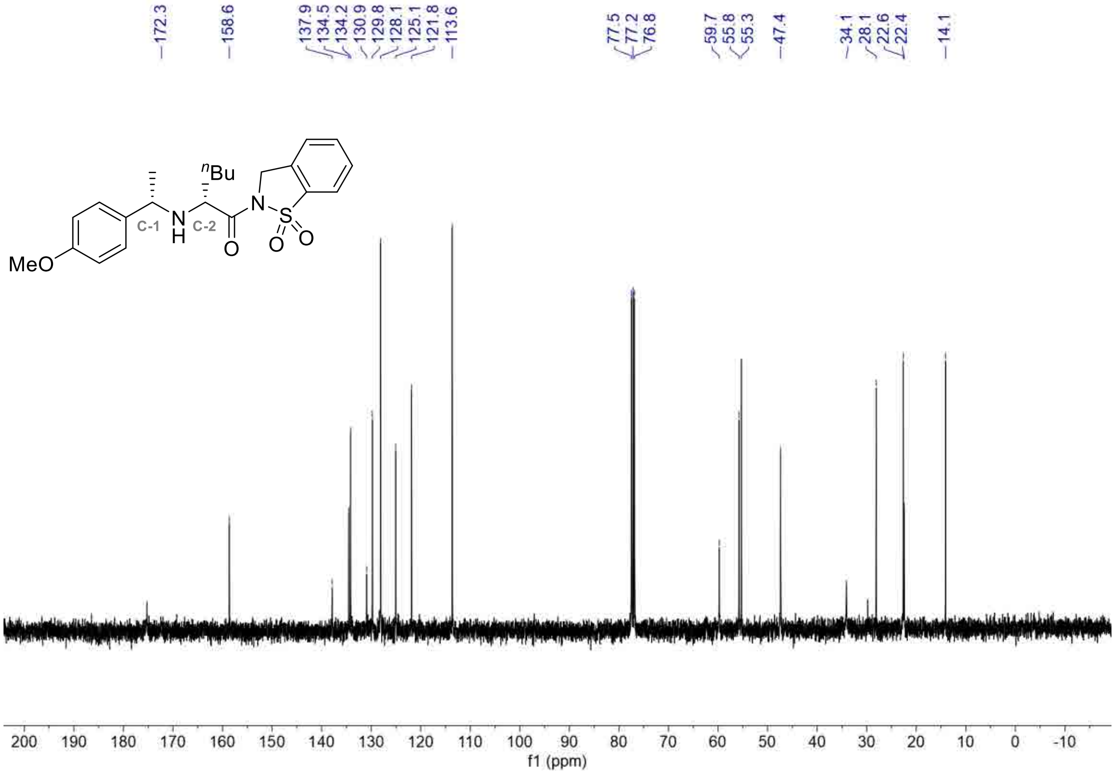

^{1}H NMR (400 MHz, $CDCl_3$) – ($R_{S(IV)}$, $R_{C\text{-}1}$)-**S7a**

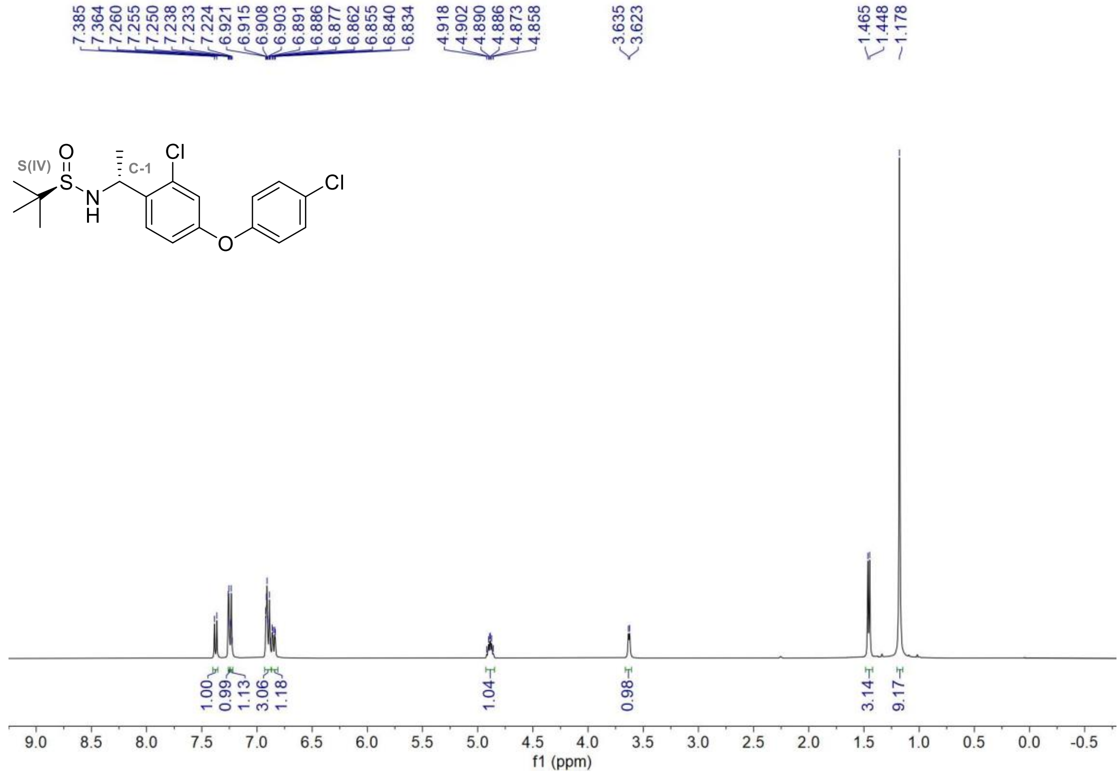

^{13}C NMR (101 MHz, $CDCl_3$) – ($R_{S(IV)}$, $R_{C\text{-}1}$)-**S7a**

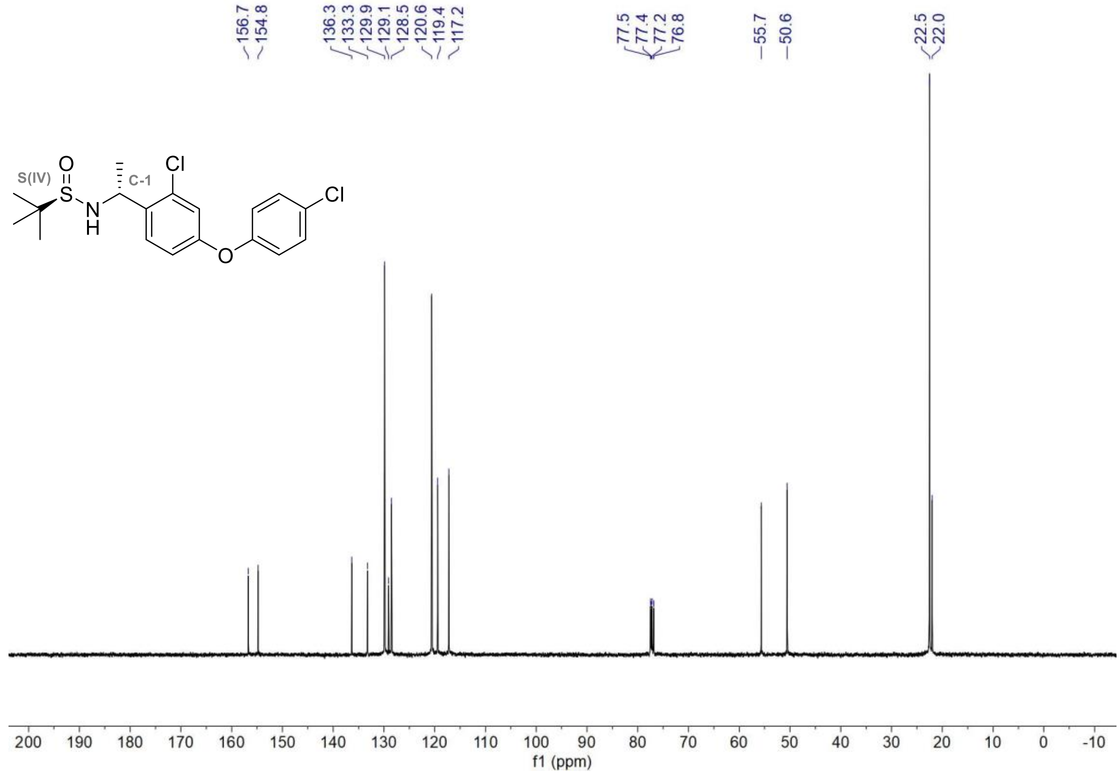

^{1}H NMR (400 MHz, $CDCl_3$) – ($R_{C\text{-}1}$, $S_{C\text{-}2}$)-**7a**

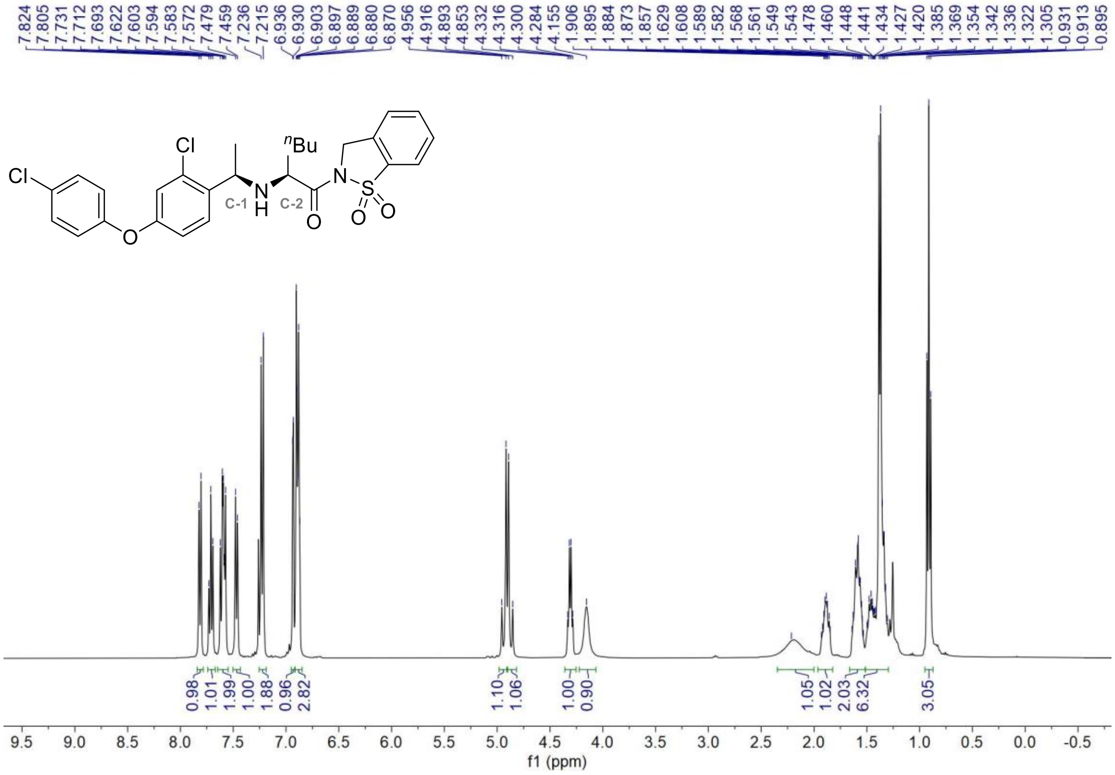

^{13}C NMR (101 MHz, $CDCl_3$) – ($R_{C\text{-}1}$, $S_{C\text{-}2}$)-**7a**

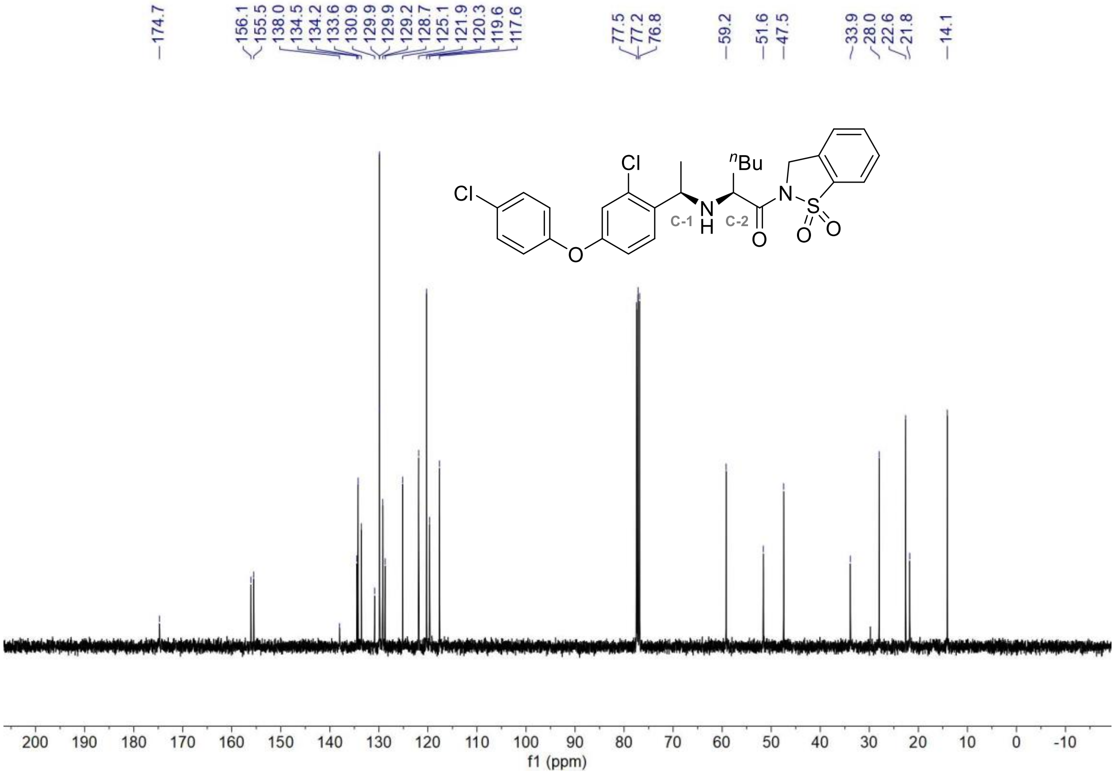

^{1}H NMR (400 MHz, $CDCl_3$) – ($S_{S(IV)}$, $S_{C\text{-}1}$)-**S7b**

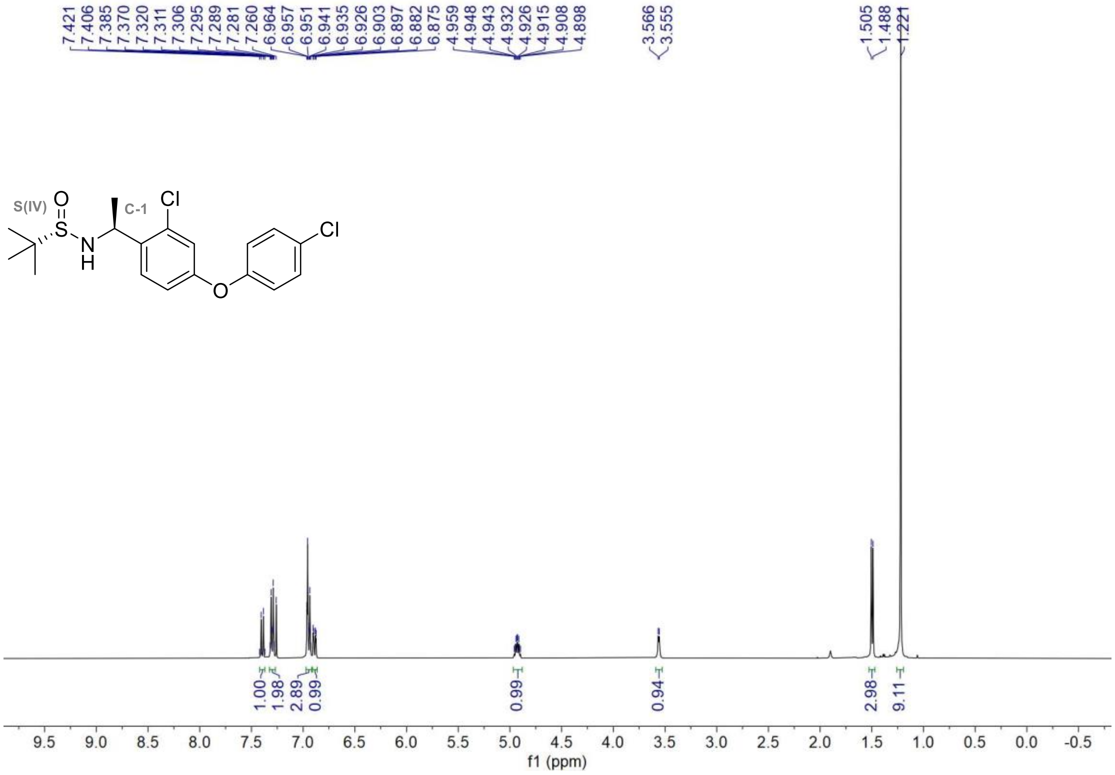

^{13}C NMR (101 MHz, $CDCl_3$) – ($S_{S(IV)}$, $S_{C\text{-}1}$)-**S7b**

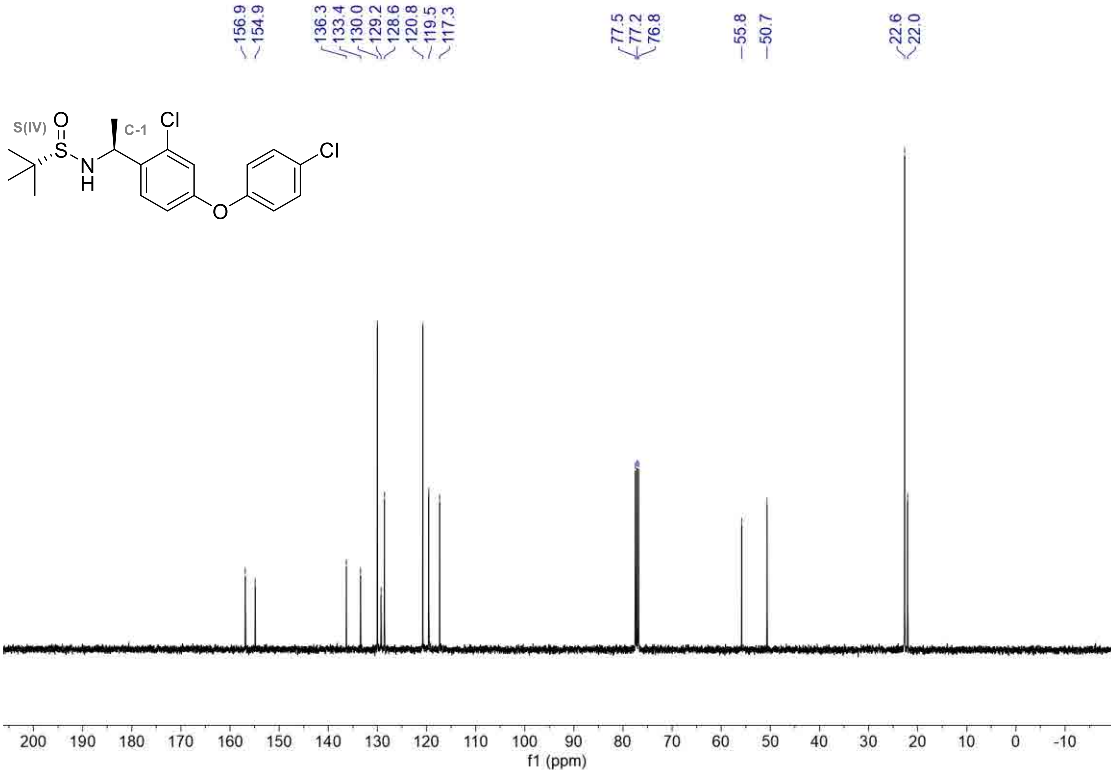

^{1}H NMR (400 MHz, $CDCl_3$) – ($S_{C\text{-}1}$, $R_{C\text{-}2}$)-**7b**

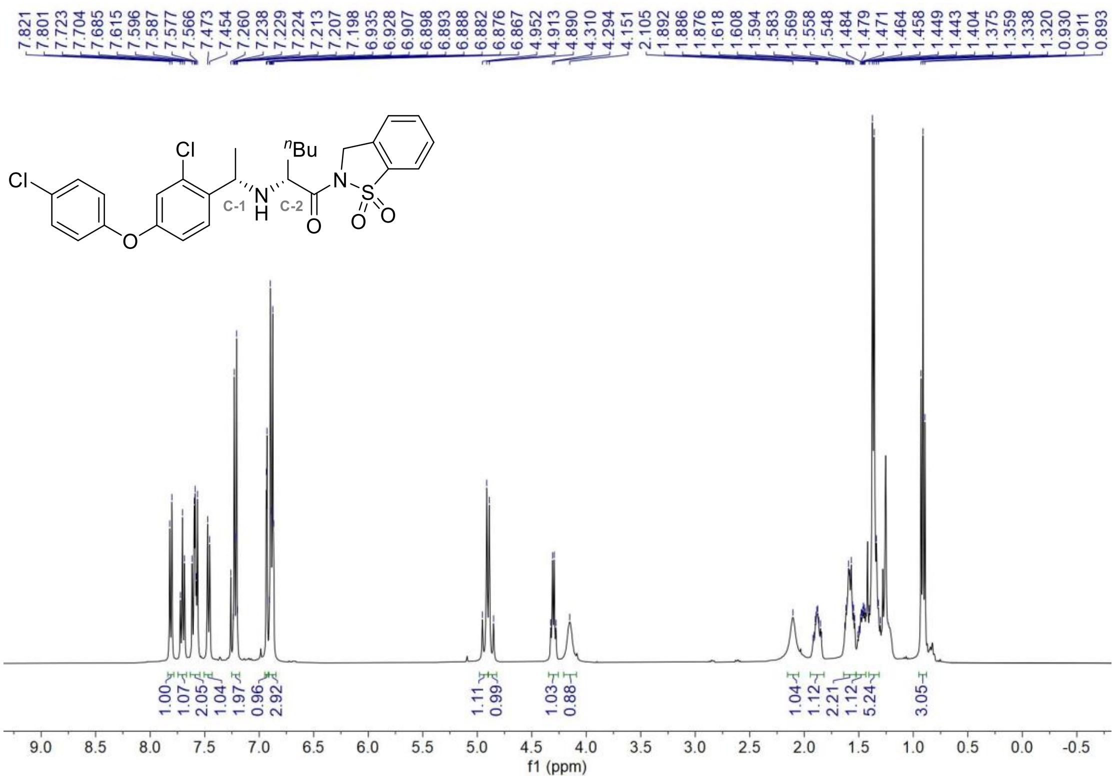

^{13}C NMR (101 MHz, $CDCl_3$) – ($S_{C\text{-}1}$, $R_{C\text{-}2}$)-**7b**

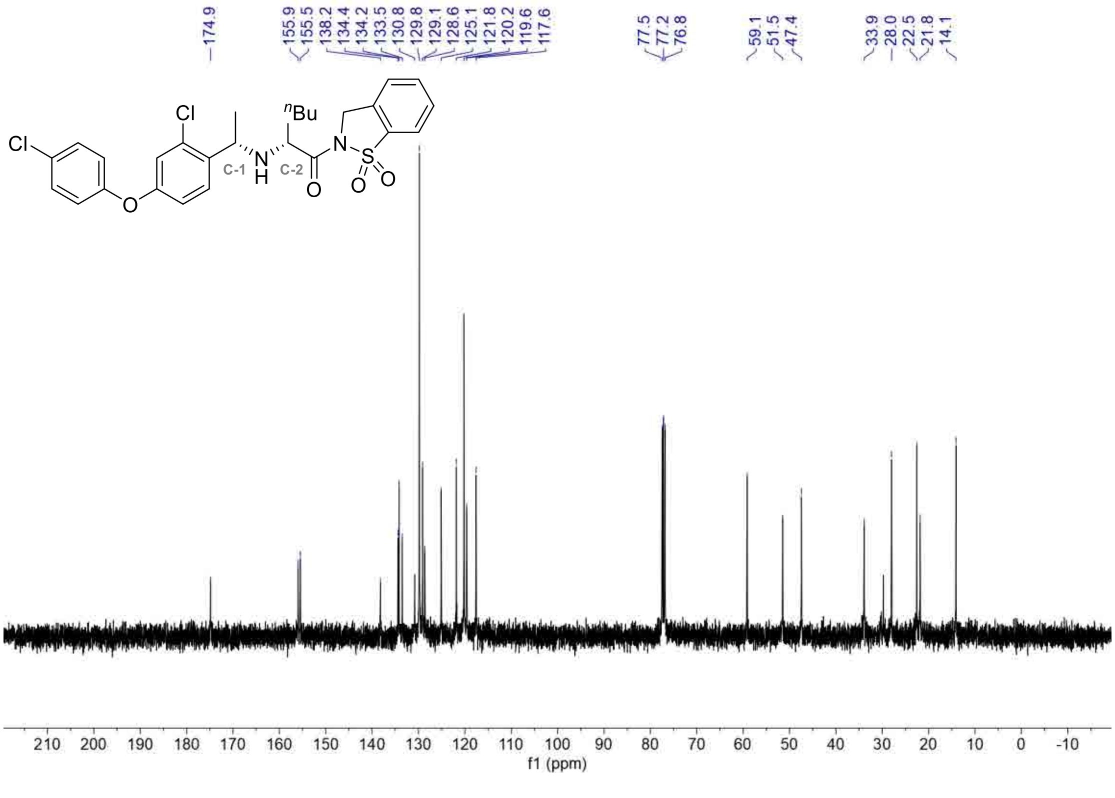

^{1}H NMR (400 MHz, $CDCl_3$) – ($R_{S(IV)}$, $R_{C\text{-}1}$)-**S8a**

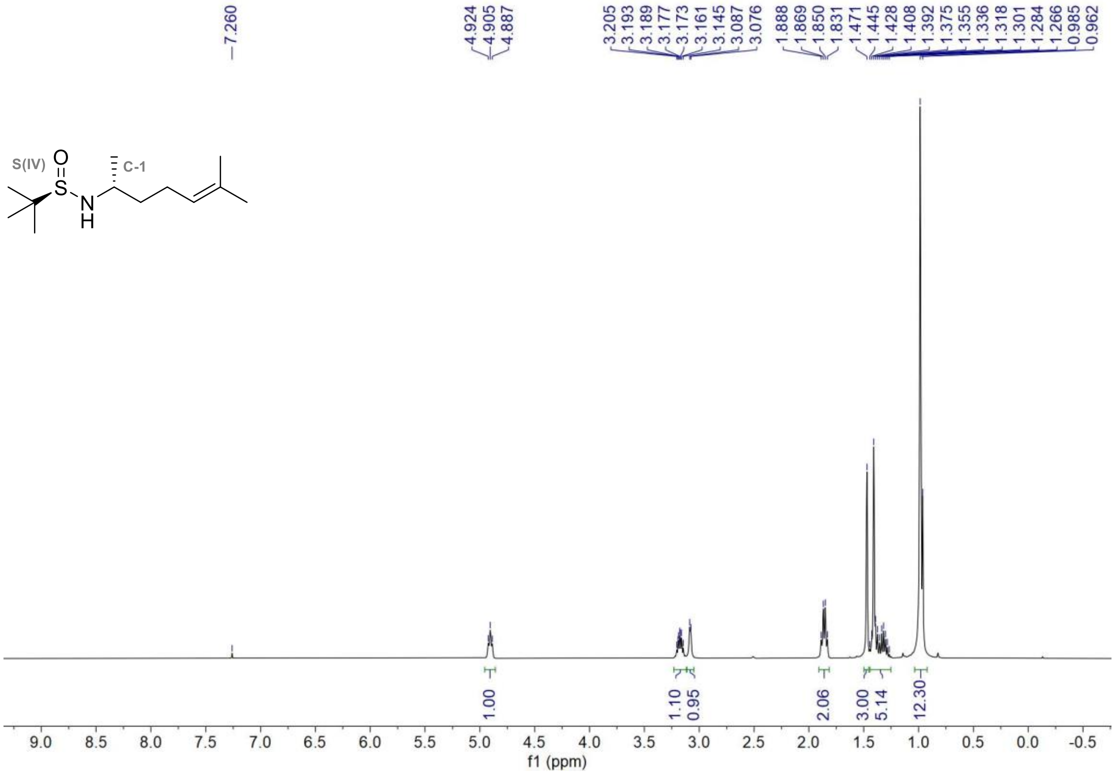

^{13}C NMR (101 MHz, $CDCl_3$) – ($R_{S(IV)}$, $R_{C\text{-}1}$)-**S8a**

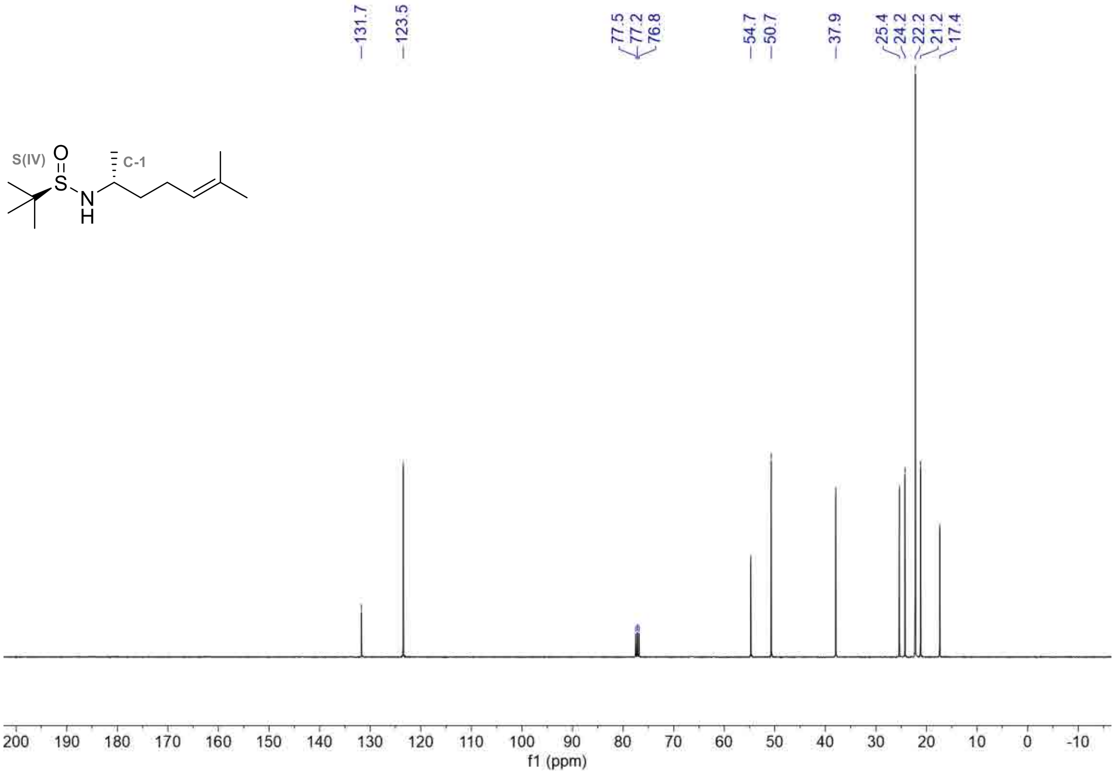

^{1}H NMR (400 MHz, $CDCl_3$) – ($R_{C\text{-}1}$, $S_{C\text{-}2}$)-**8a**

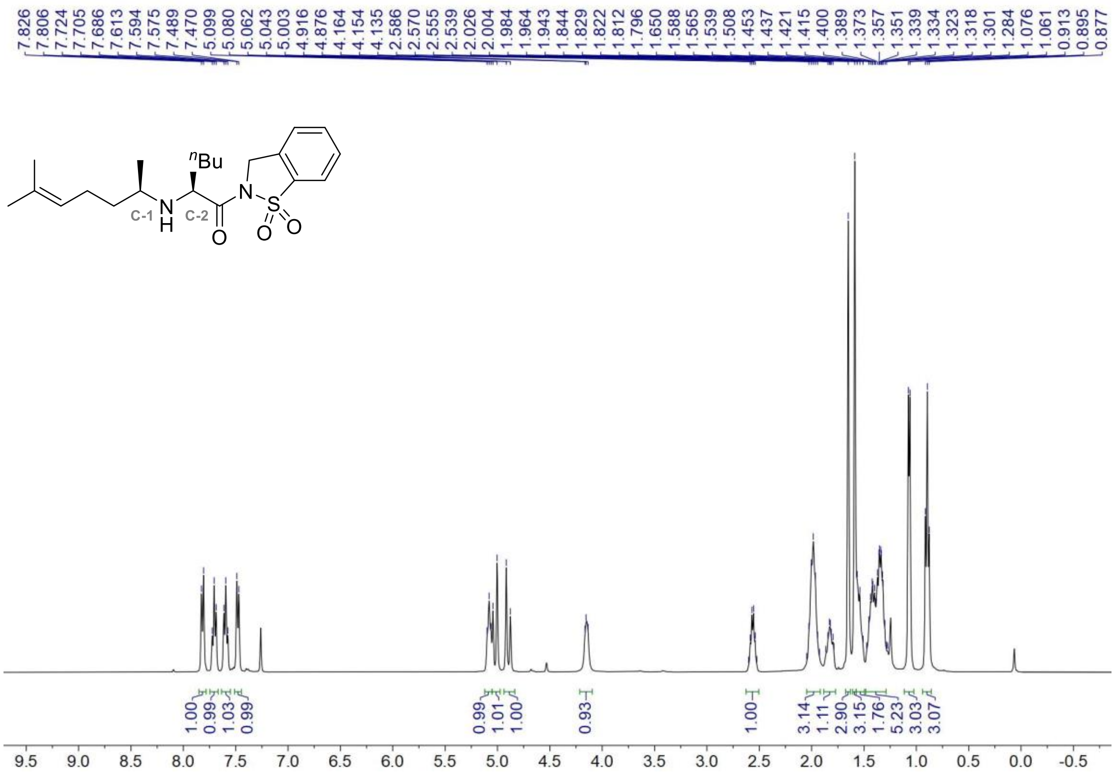

^{13}C NMR (101 MHz, $CDCl_3$) – ($R_{C\text{-}1}$, $S_{C\text{-}2}$)-**8a**

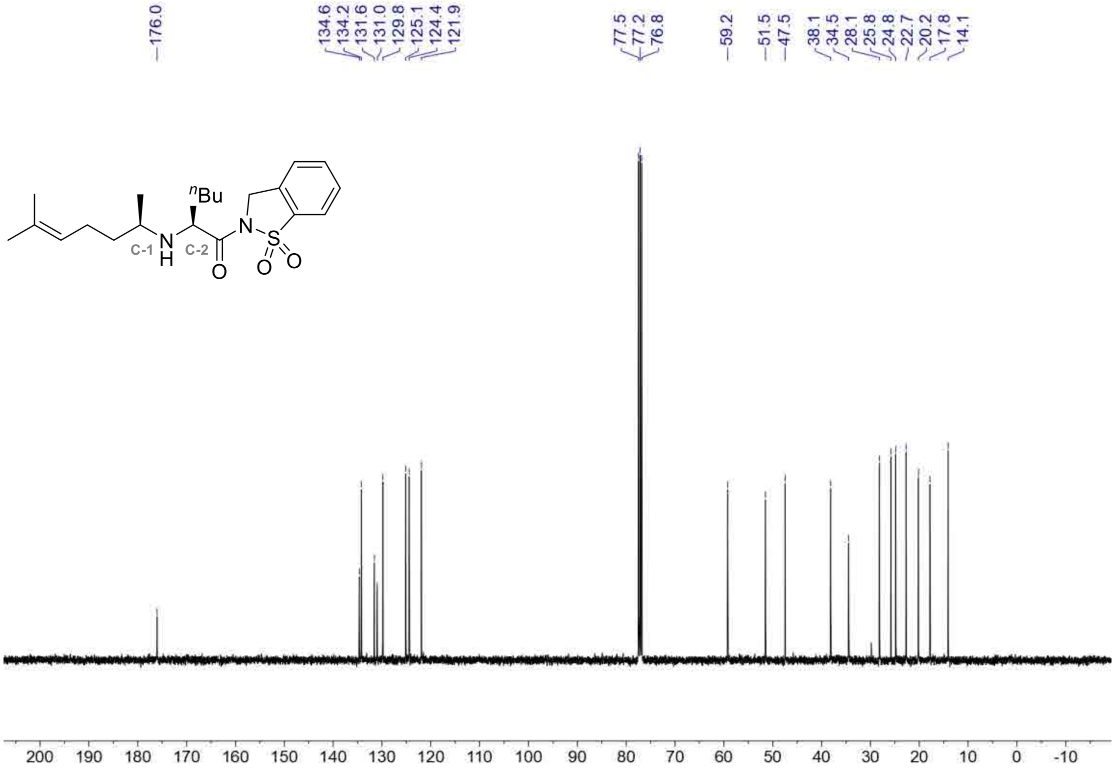

^{1}H NMR (400 MHz, $CDCl_3$) – ($S_{S(IV)}$, $S_{C\text{-}1}$)-**S8b**

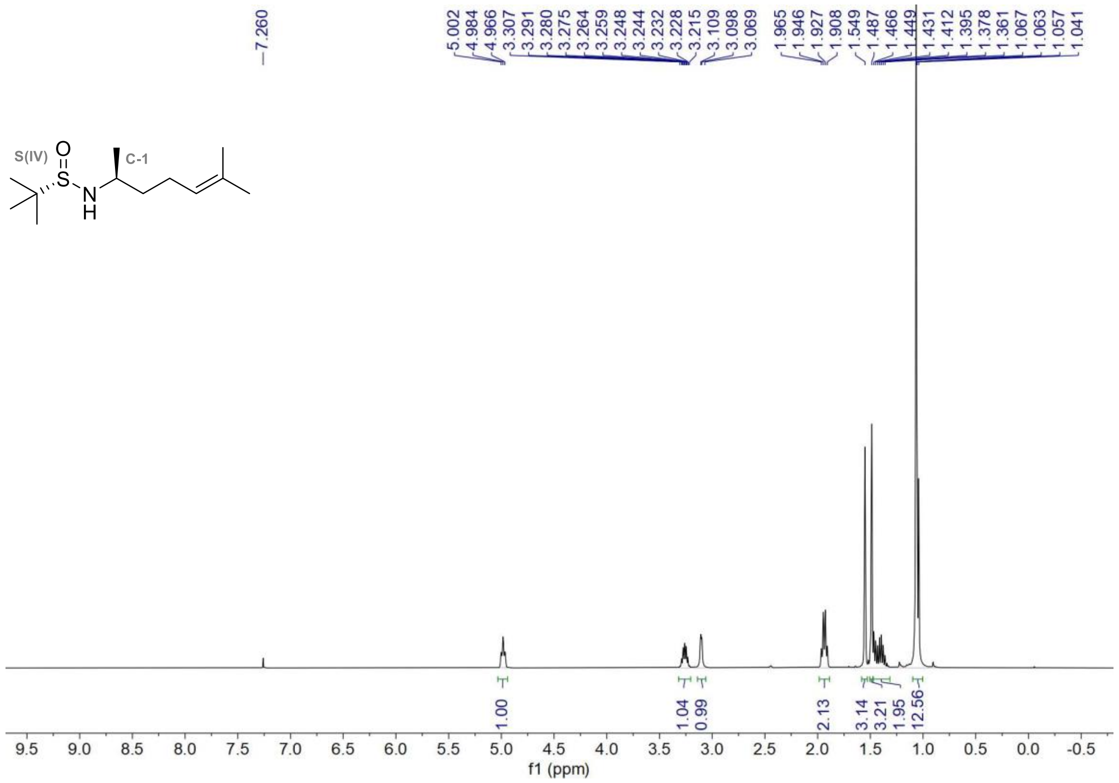

^{13}C NMR (101 MHz, $CDCl_3$) – ($S_{S(IV)}$, $S_{C\text{-}1}$)-**S8b**

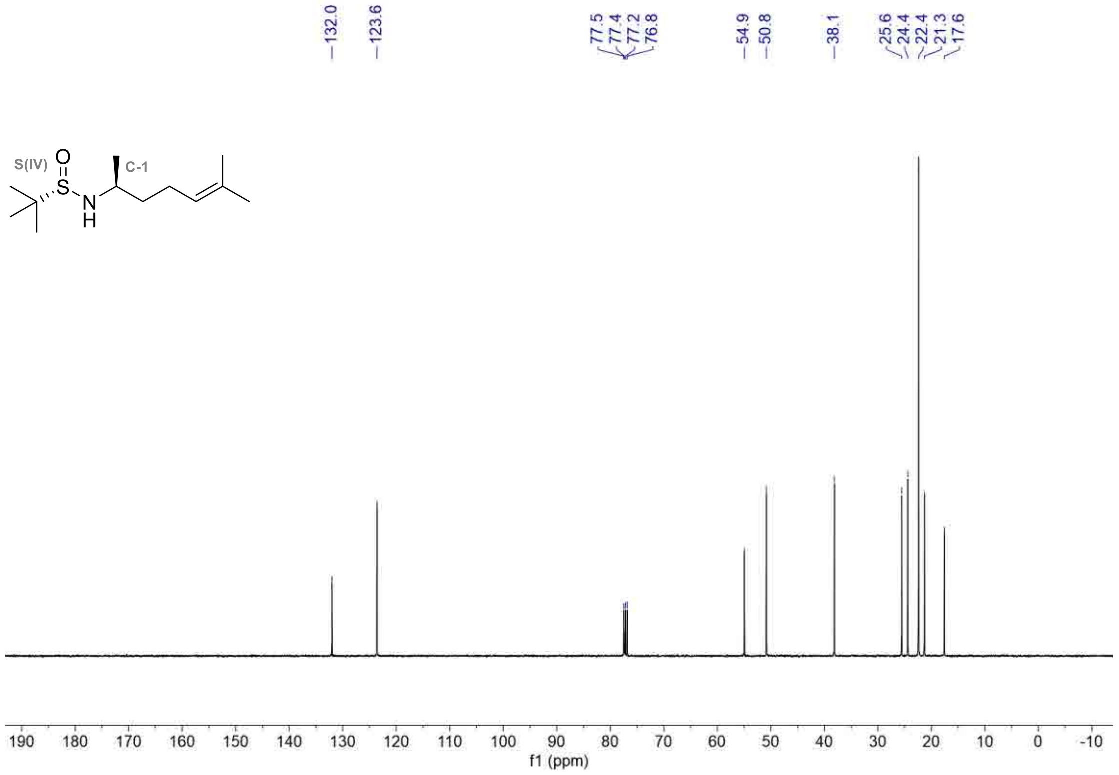

^{1}H NMR (400 MHz, $CDCl_3$) – ($S_{C\text{-}1}$, $R_{C\text{-}2}$)-**8b**

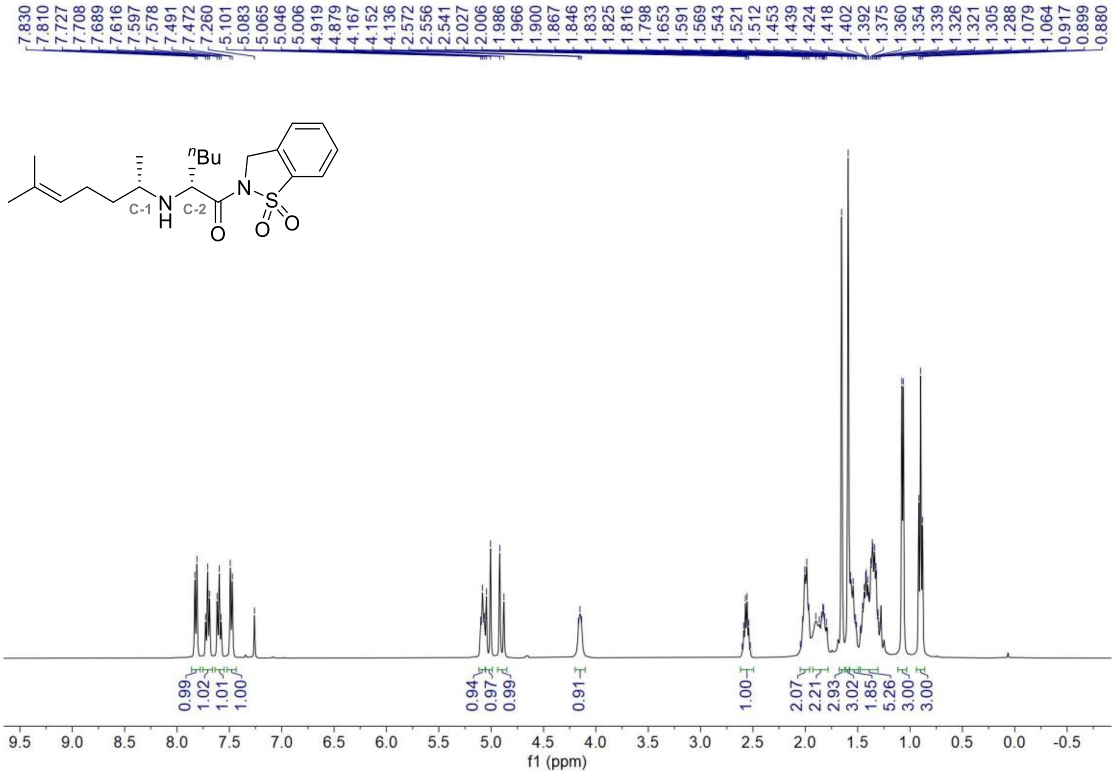

^{13}C NMR (101 MHz, $CDCl_3$) – ($S_{C\text{-}1}$, $R_{C\text{-}2}$)-**8b**

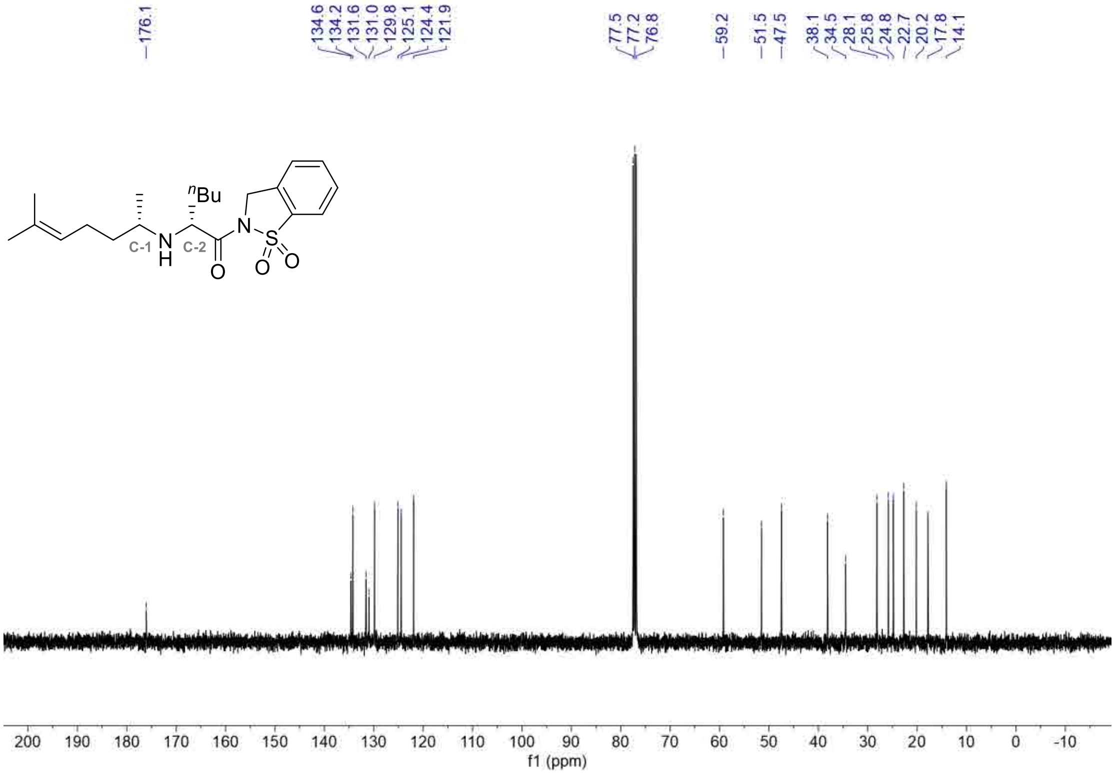

^{1}**H NMR** (400 MHz, $CDCl_3$) – ($R_{S(IV)}$, R_{C-1})-**S9a**

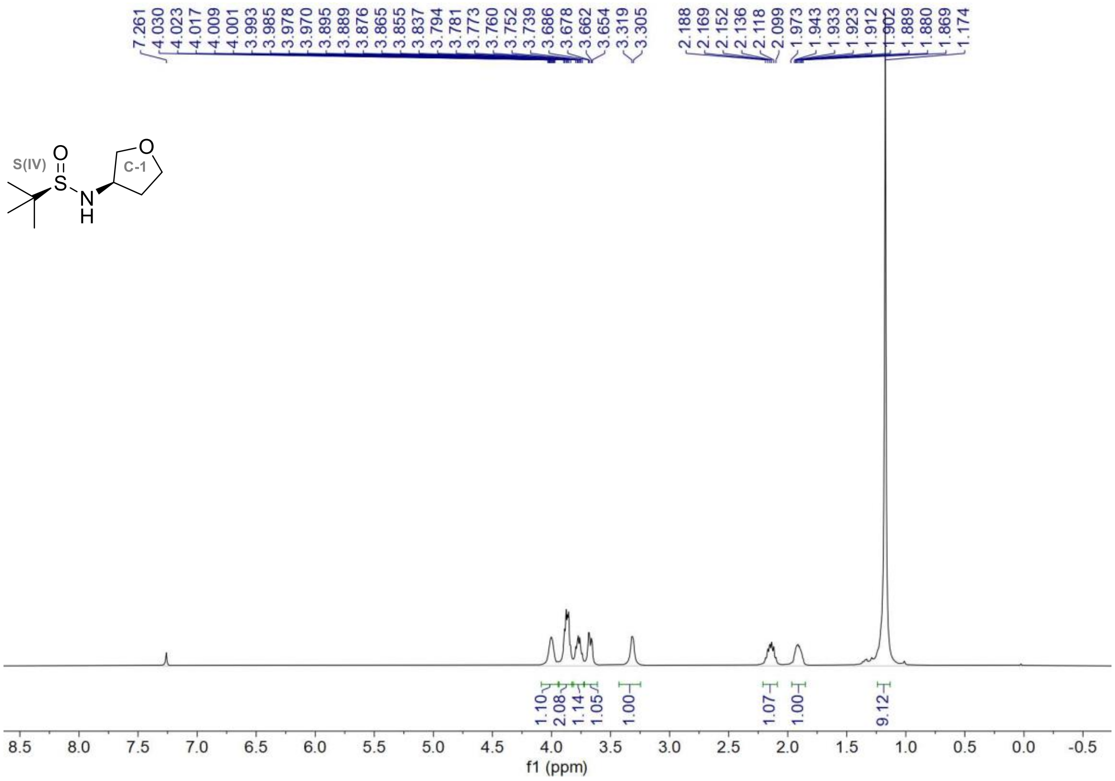

^{13}**C NMR** (101 MHz, $CDCl_3$) – ($R_{S(IV)}$, R_{C-1})-**S9a**

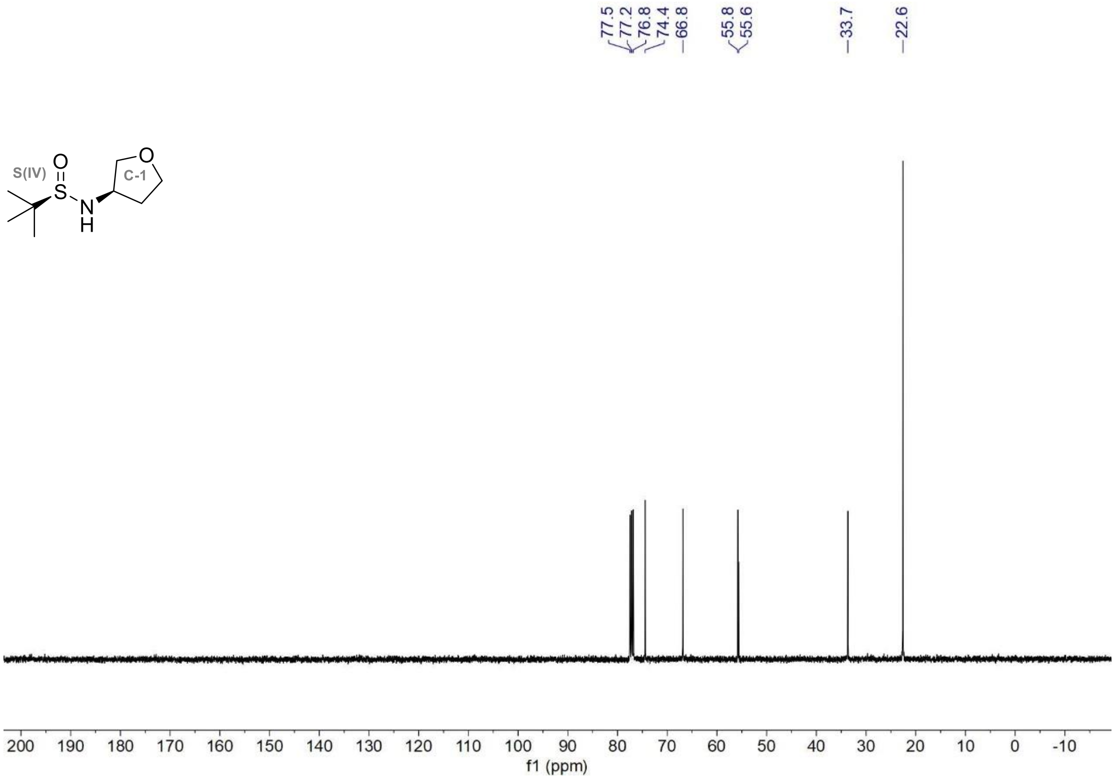

^{1}H NMR (400 MHz, $CDCl_3$) – (R_{C-1}, S_{C-2})-**9a**

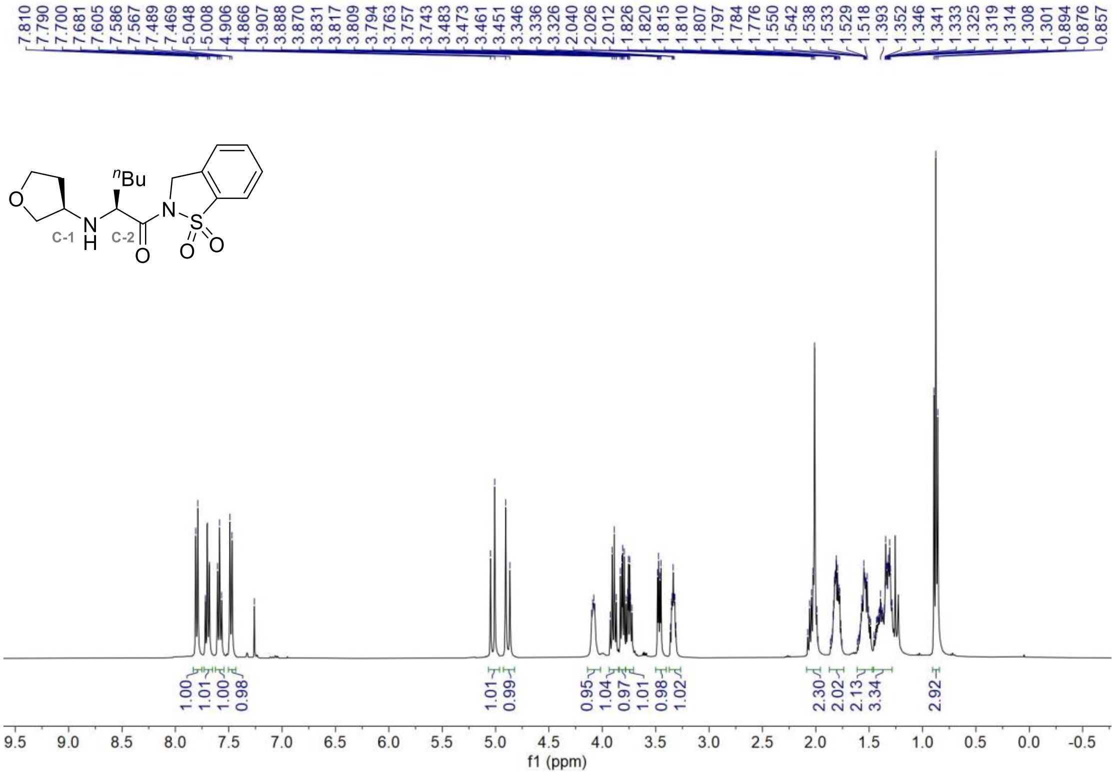

^{13}C NMR (101 MHz, $CDCl_3$) – (R_{C-1}, S_{C-2})-**9a**

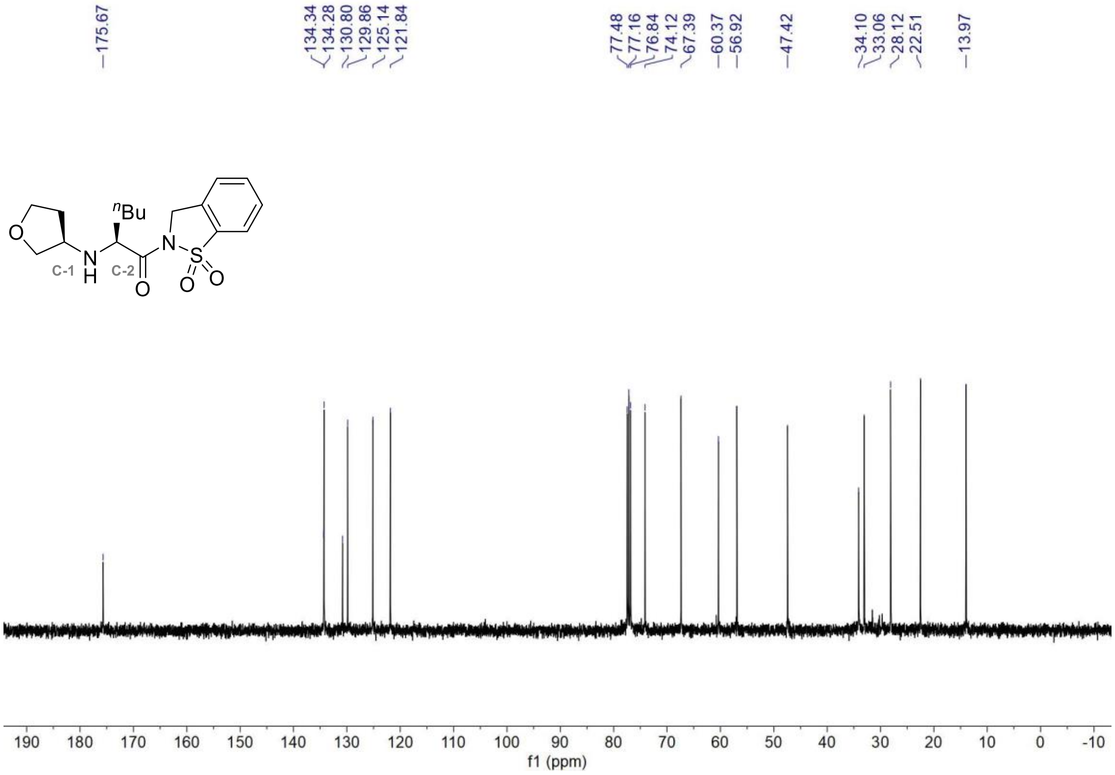

¹H NMR (400 MHz, $CDCl_3$) – (±$_{S(IV)}$, $R_{C\text{-}1}$)-**S9b**

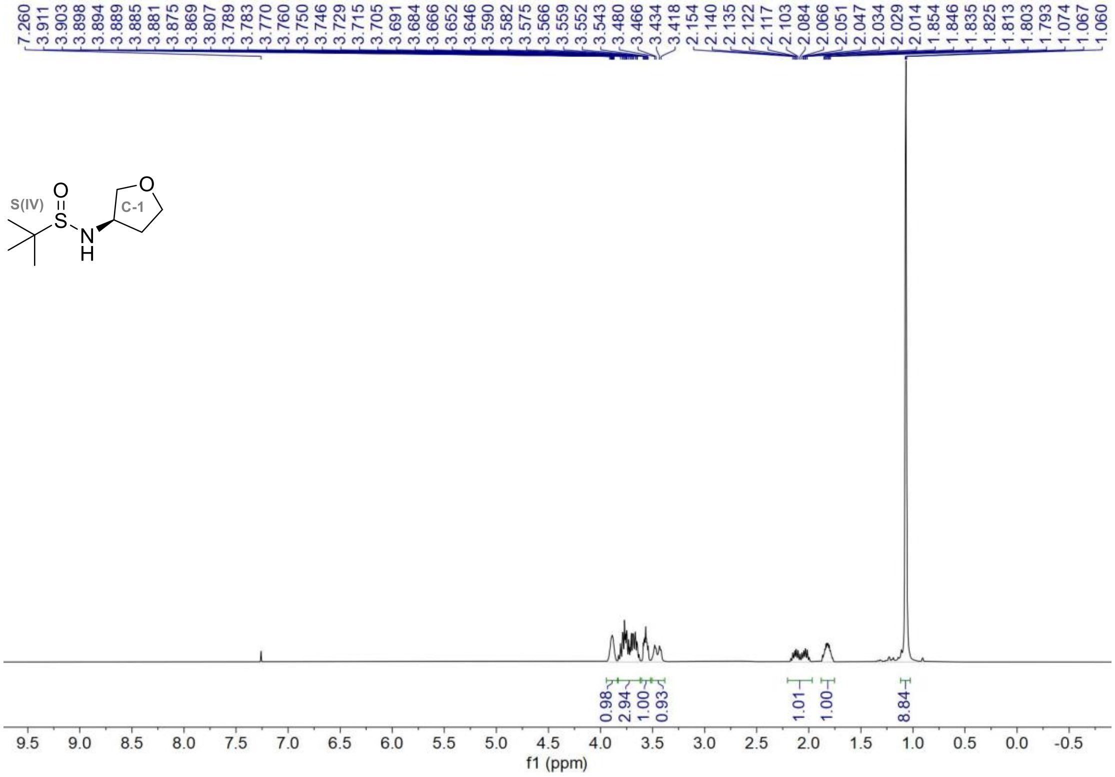

¹³C NMR (101 MHz, $CDCl_3$) – (±$_{S(IV)}$, $R_{C\text{-}1}$)-**S9b**

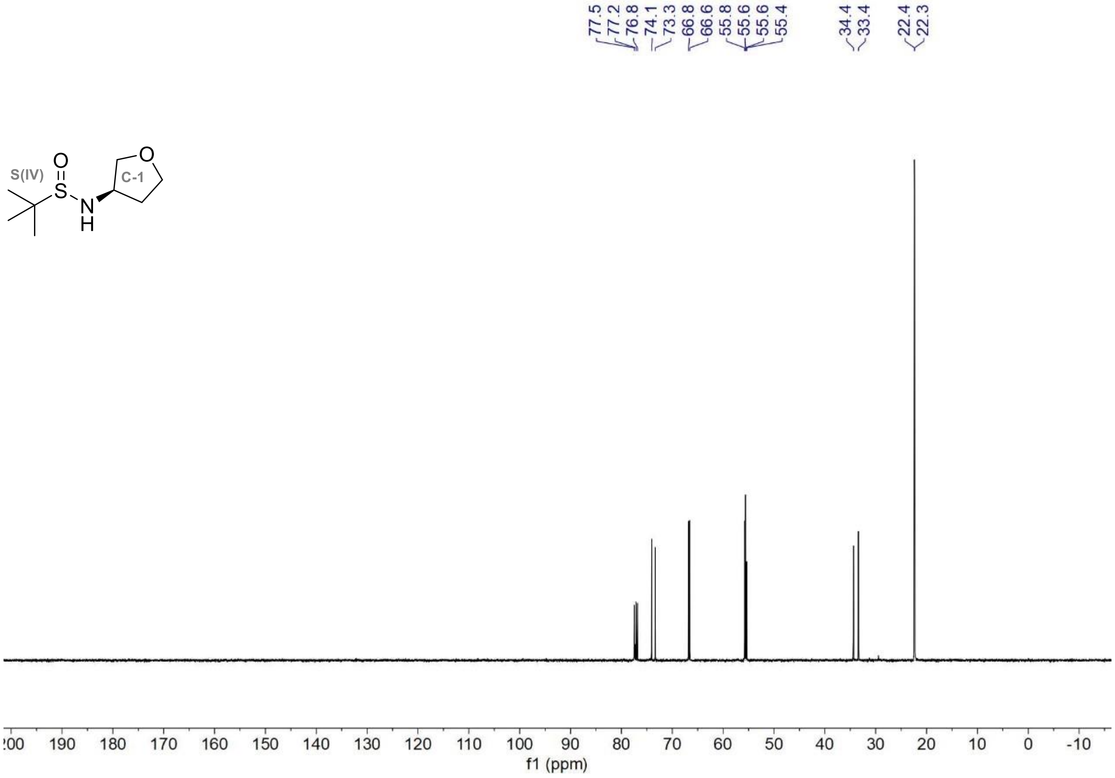

^{1}H NMR (400 MHz, $CDCl_3$) – ($S_{S(IV)}$, $R_{C\text{-}1}$)-**S10a**

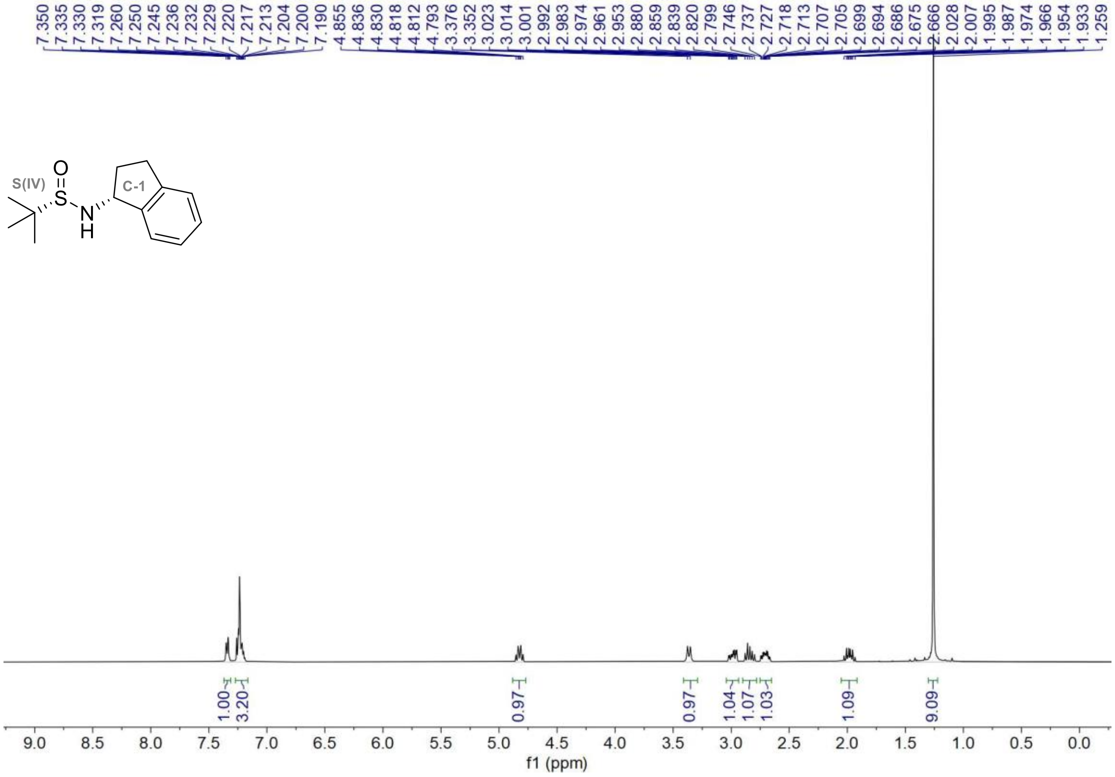

^{13}C NMR (101 MHz, $CDCl_3$) – ($S_{S(IV)}$, $R_{C\text{-}1}$)-**S10a**

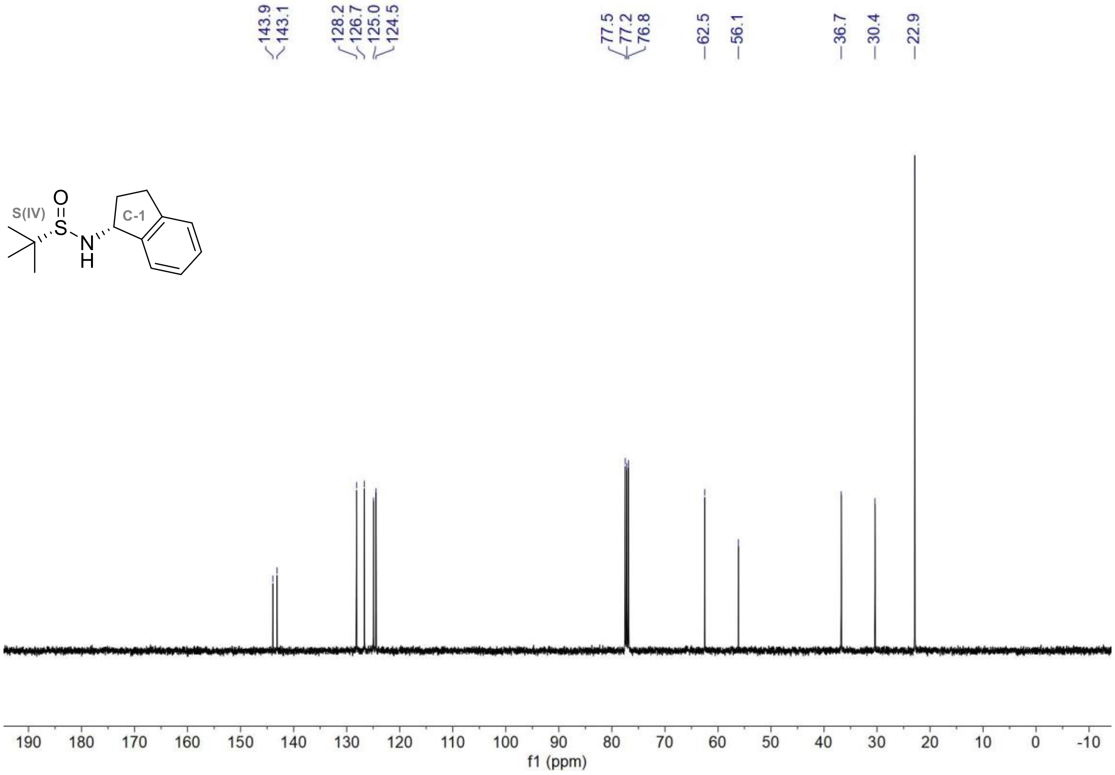

^{1}H NMR (400 MHz, $CDCl_3$) – ($R_{C\text{-}1}$, $R_{C\text{-}2}$)-**10a**

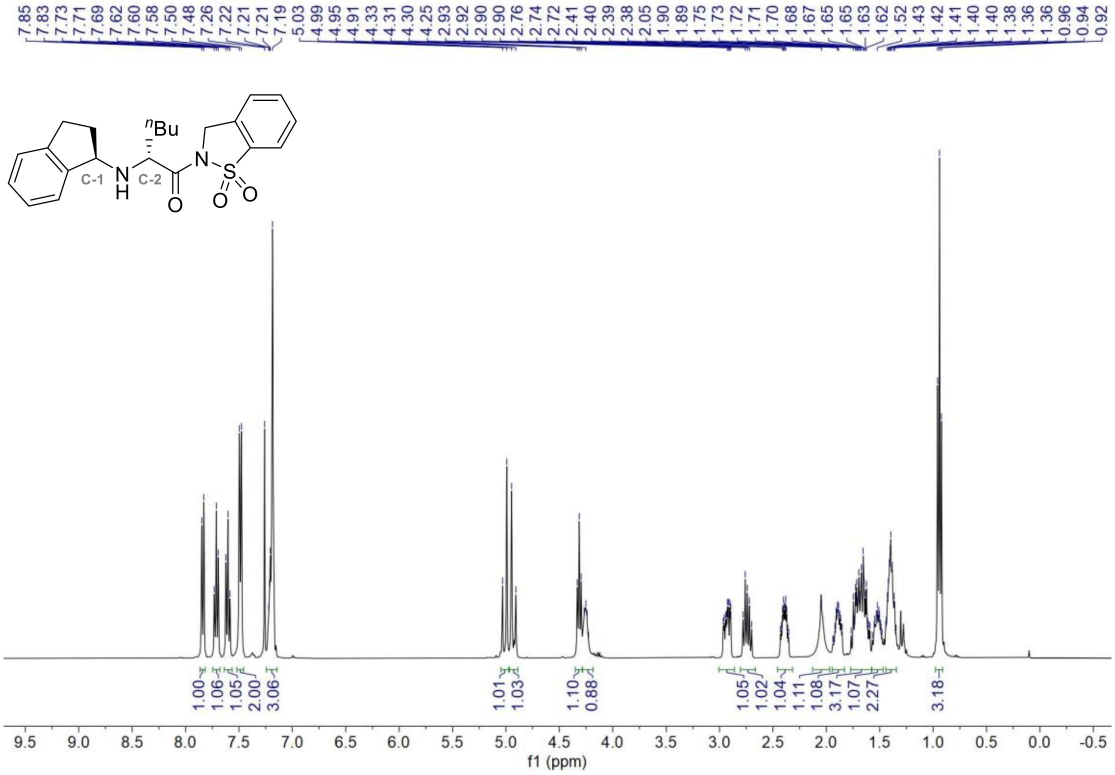

^{13}C NMR (101 MHz, $CDCl_3$) – ($R_{C\text{-}1}$, $R_{C\text{-}2}$)-**10a**

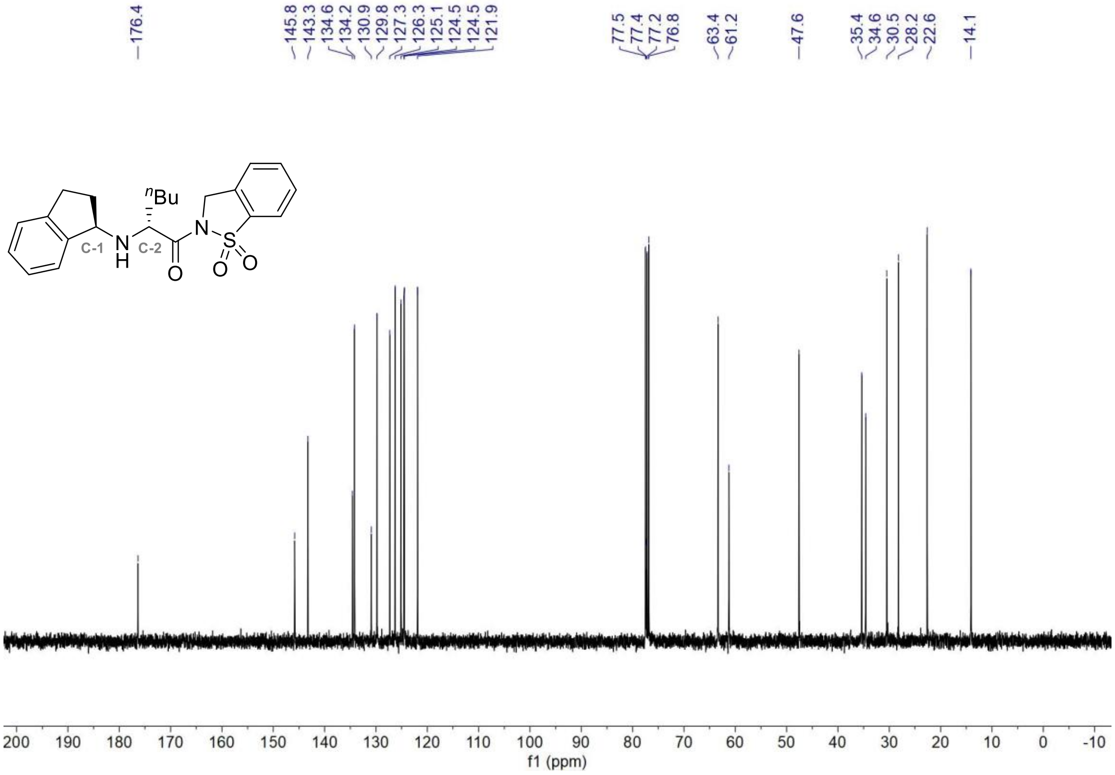

^{1}H NMR (400 MHz, $CDCl_3$) – (R_{C-1}, S_{C-2})-**10b**

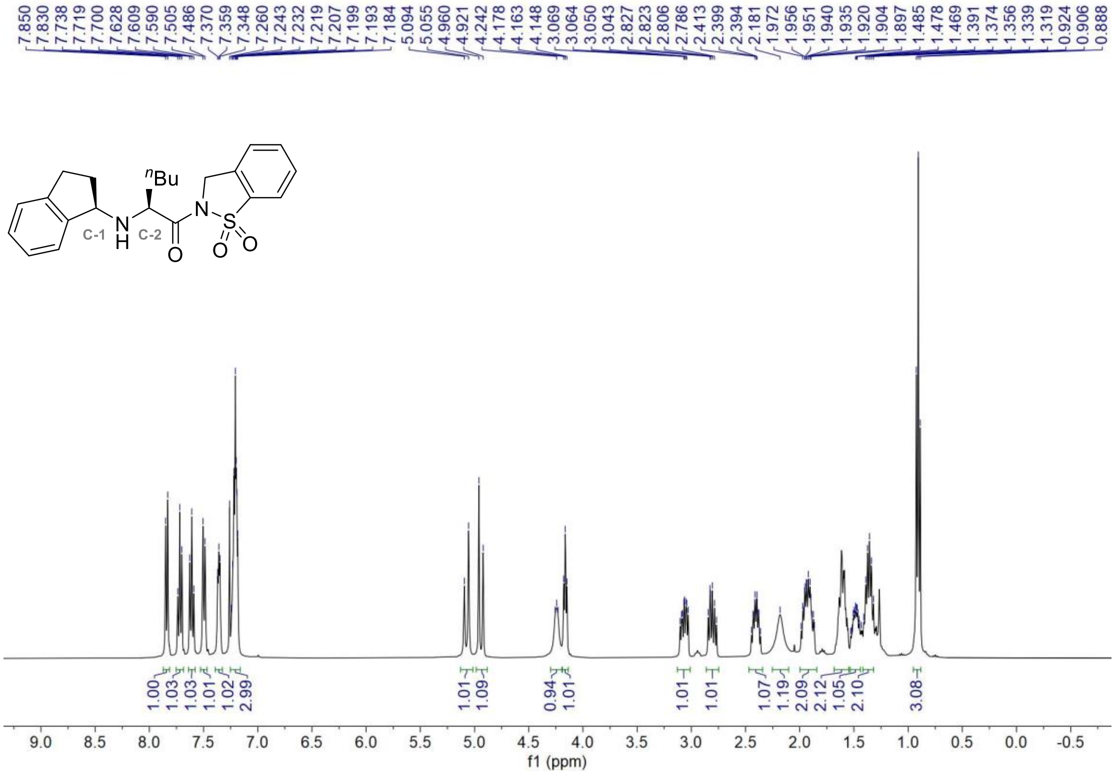

^{13}C NMR (101 MHz, $CDCl_3$) – (R_{C-1}, S_{C-2})-**10b**

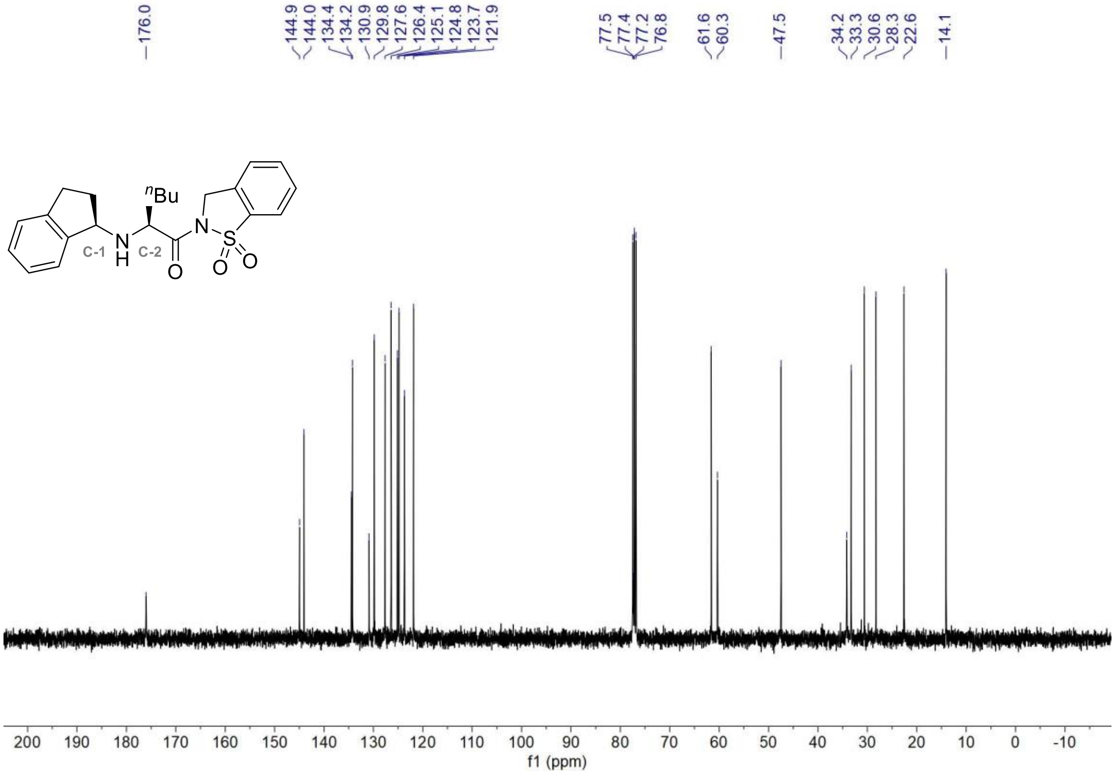

^{1}H NMR (400 MHz, $CDCl_3$) – (S_{C-1}, S_{C-2})-**11a**

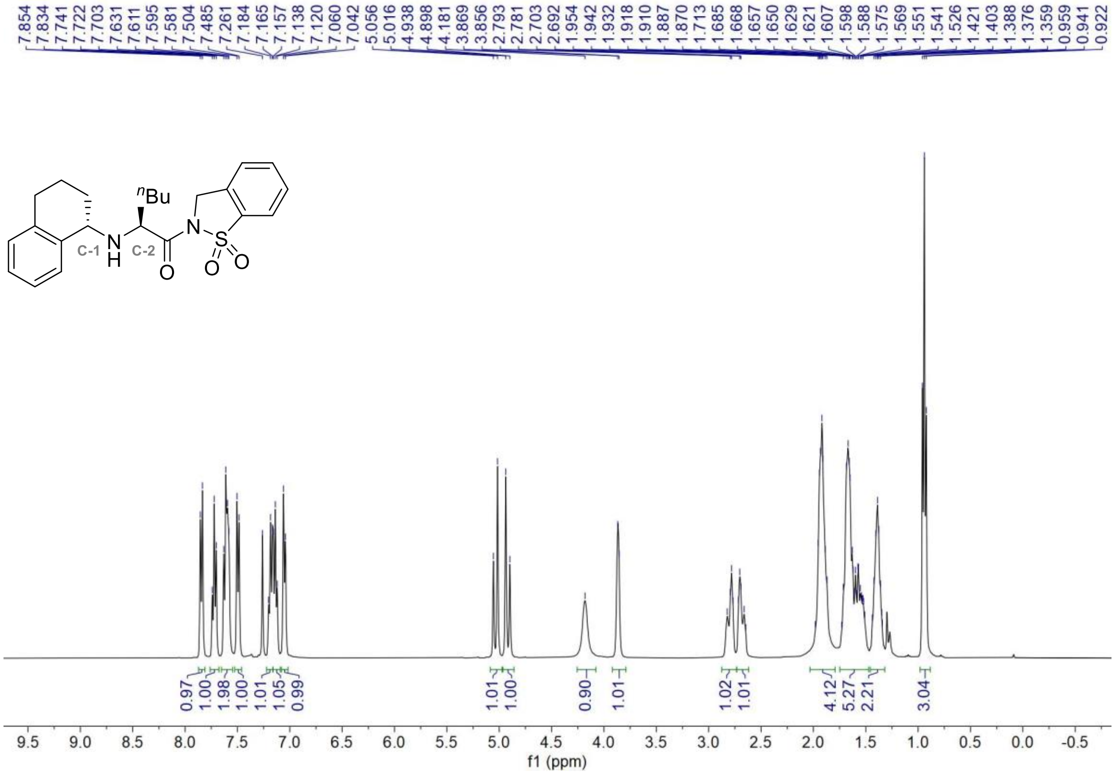

^{13}C NMR (101 MHz, $CDCl_3$) – (S_{C-1}, S_{C-2})-**11a**

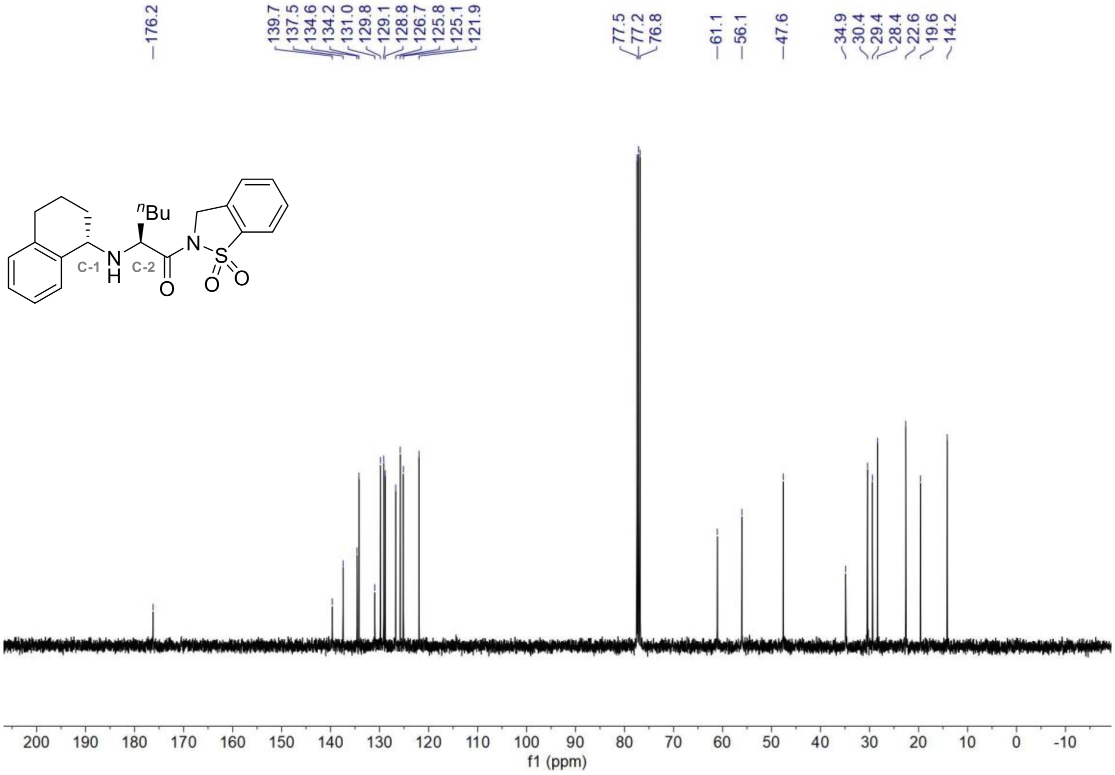

^{1}H NMR (400 MHz, $CDCl_3$) – ($S_{S(IV)}$, $S_{C\text{-}1}$)-**S11b**

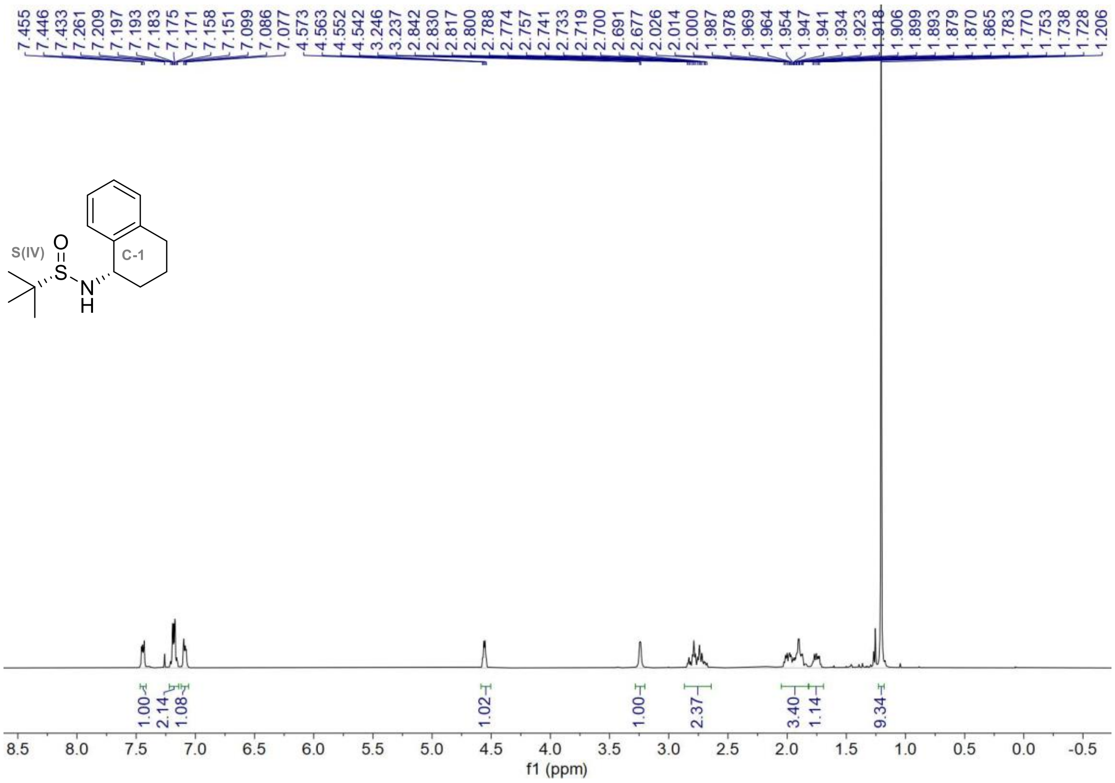

^{13}C NMR (101 MHz, $CDCl_3$) – ($S_{S(IV)}$, $S_{C\text{-}1}$)-**S11b**

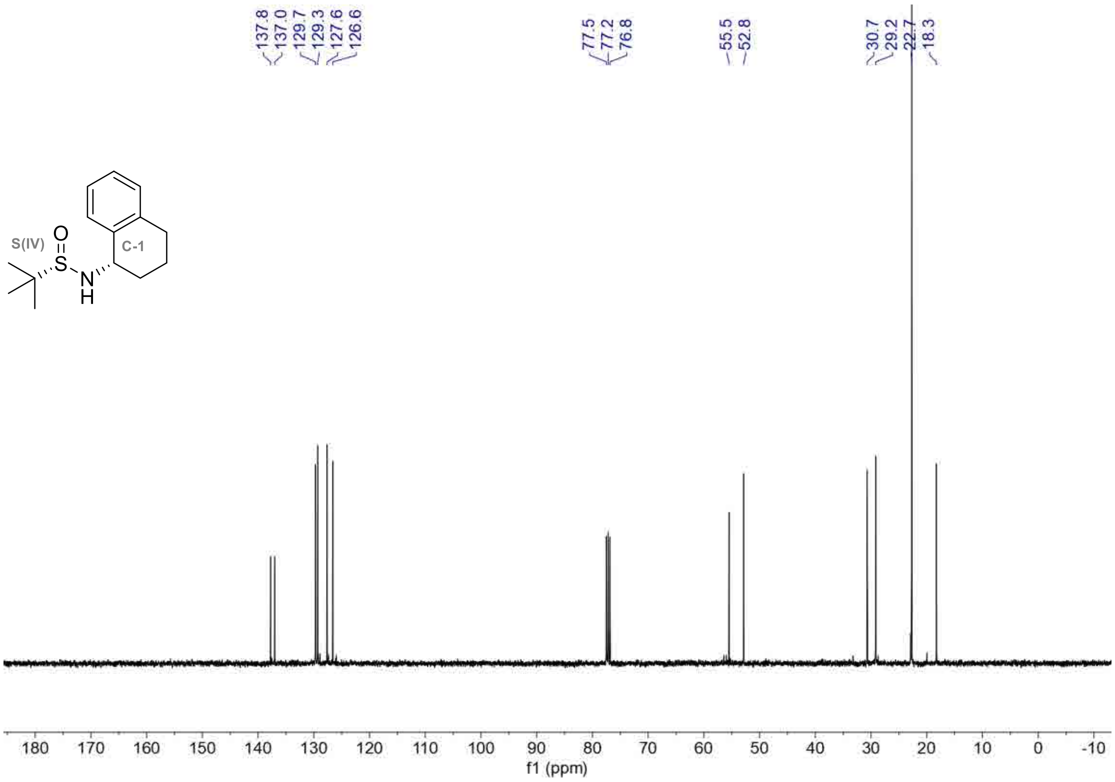

^{1}H NMR (400 MHz, $CDCl_3$) – ($S_{C\text{-}1}$, $R_{C\text{-}2}$)-**11b**

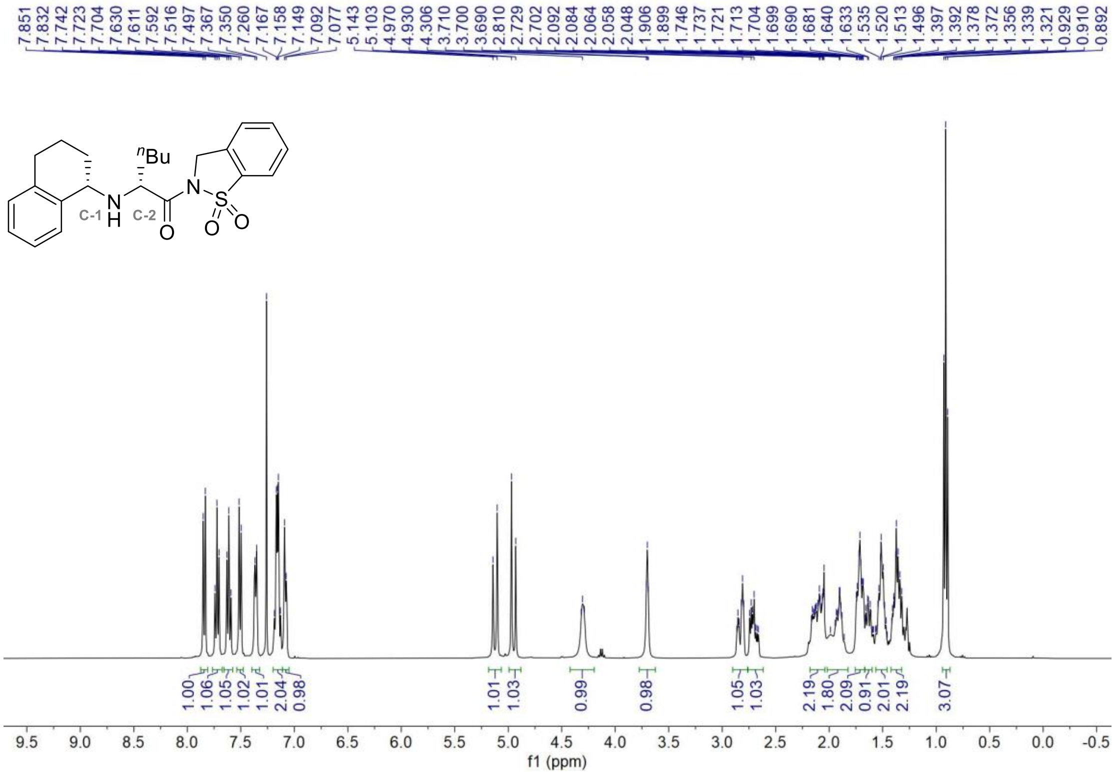

^{13}C NMR (101 MHz, $CDCl_3$) – ($S_{C\text{-}1}$, $R_{C\text{-}2}$)-**11b**

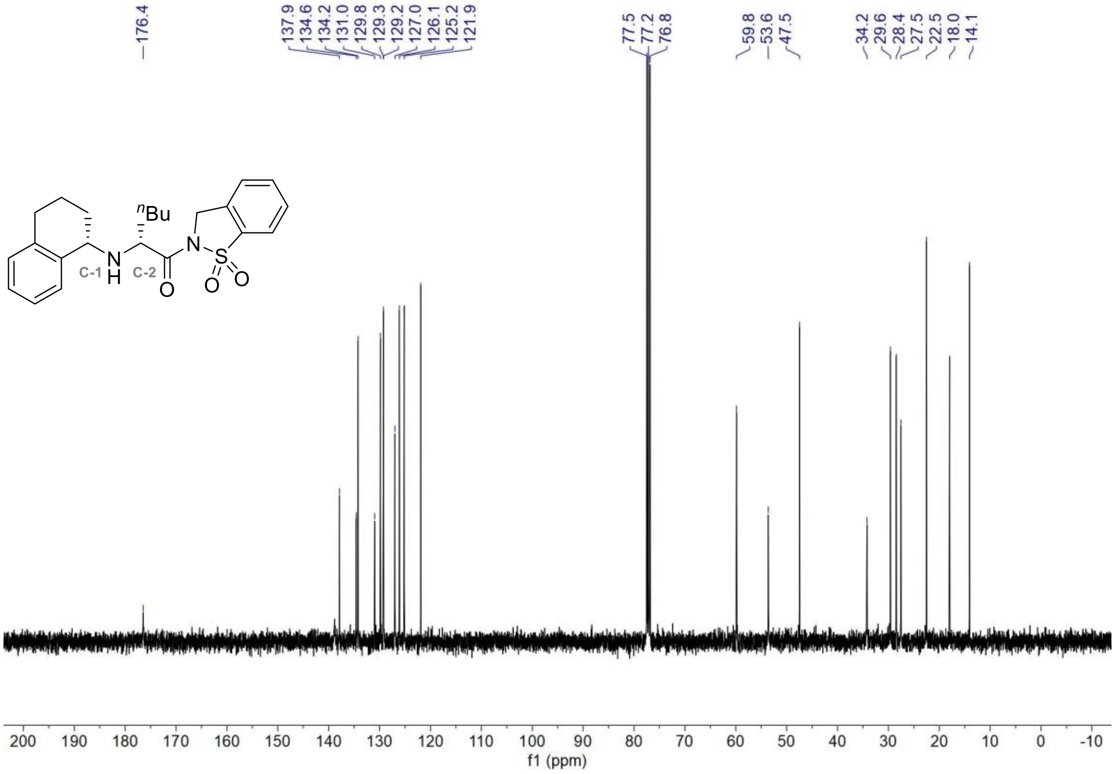

^{1}H NMR (400 MHz, $CDCl_3$) – ($R_{C\text{-}1}$, $S_{C\text{-}2}$)-**12a**

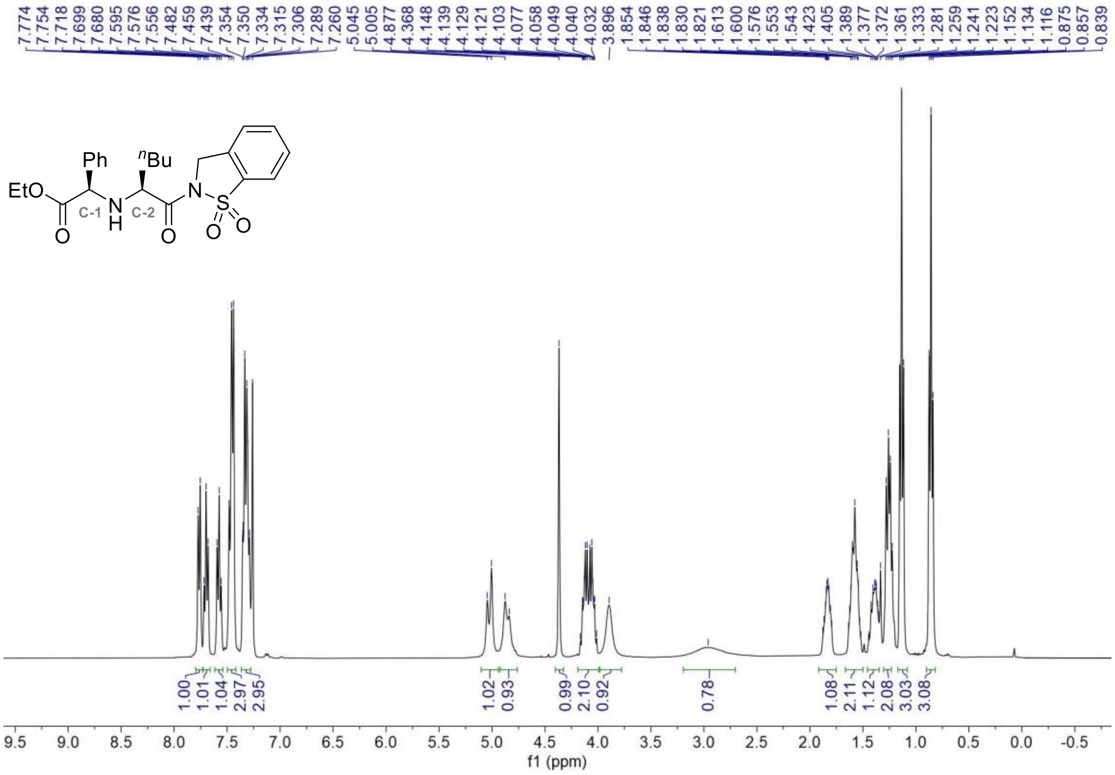

^{13}C NMR (101 MHz, $CDCl_3$) – ($R_{C\text{-}1}$, $S_{C\text{-}2}$)-**12a**

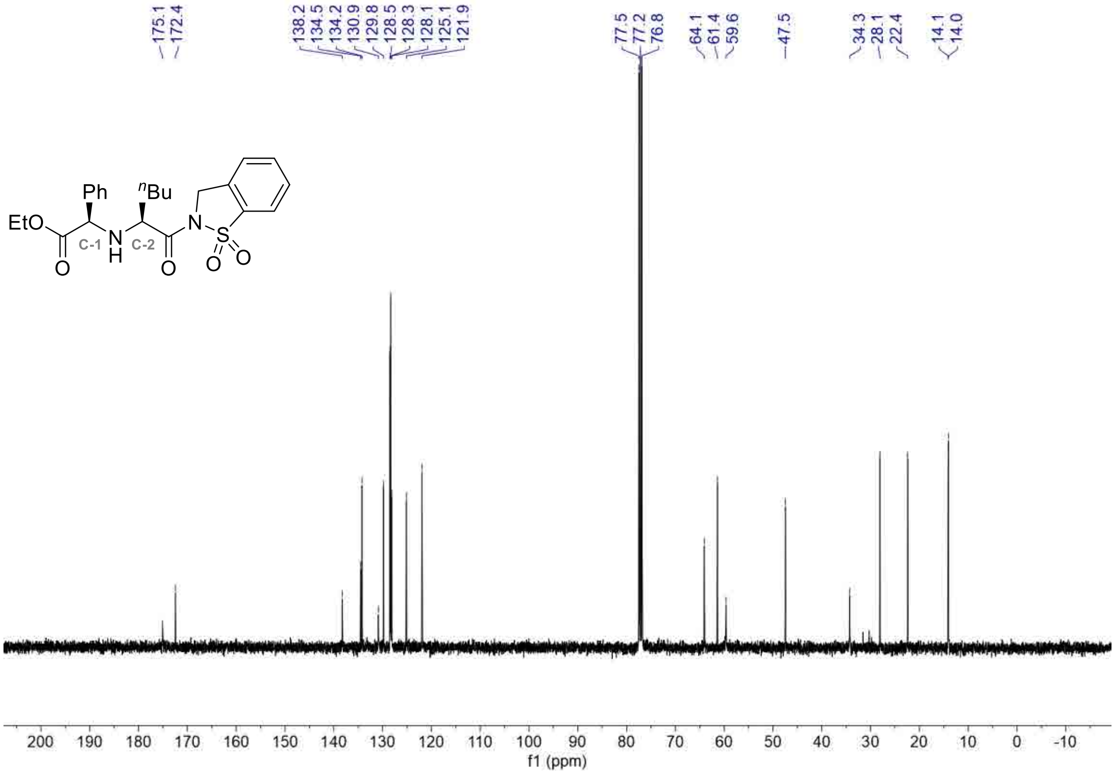

^{1}H NMR (400 MHz, $CDCl_3$) – (R_{C-1}, R_{C-2})-**12b**

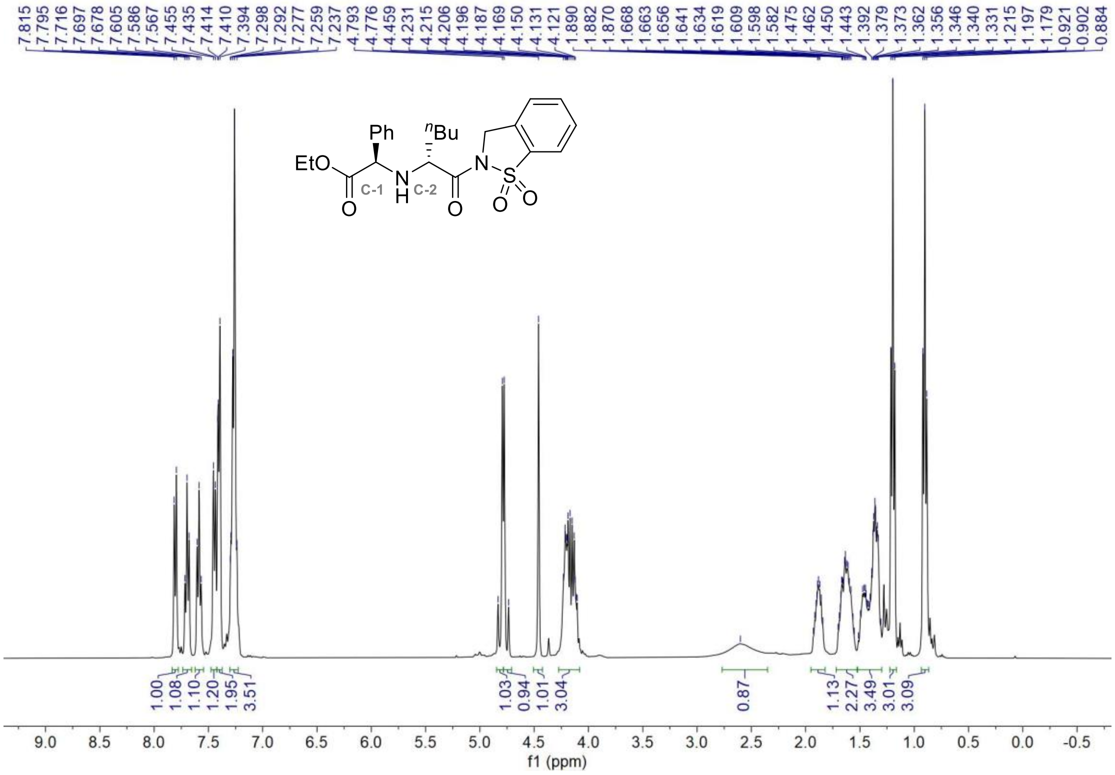

^{13}C NMR (101 MHz, $CDCl_3$) – (R_{C-1}, R_{C-2})-**12b**

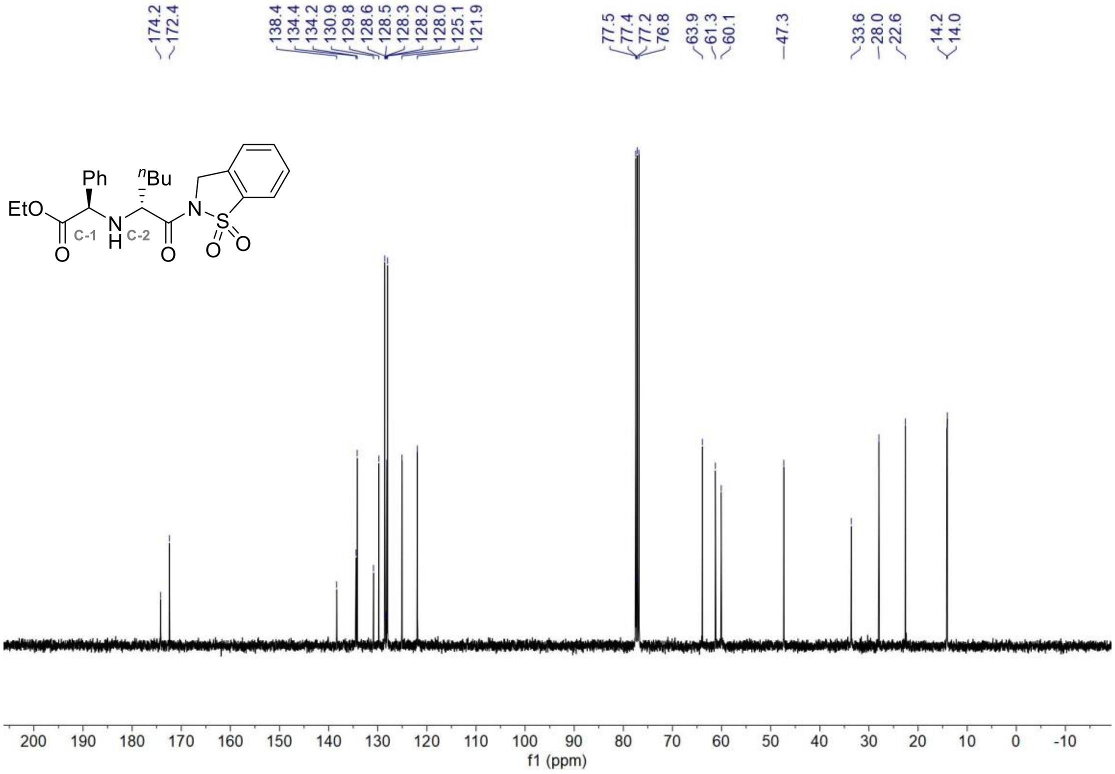

^{1}H NMR (400 MHz, $CDCl_3$) – ($S_{C\text{-}1}$, $S_{C\text{-}2}$)-**13a**

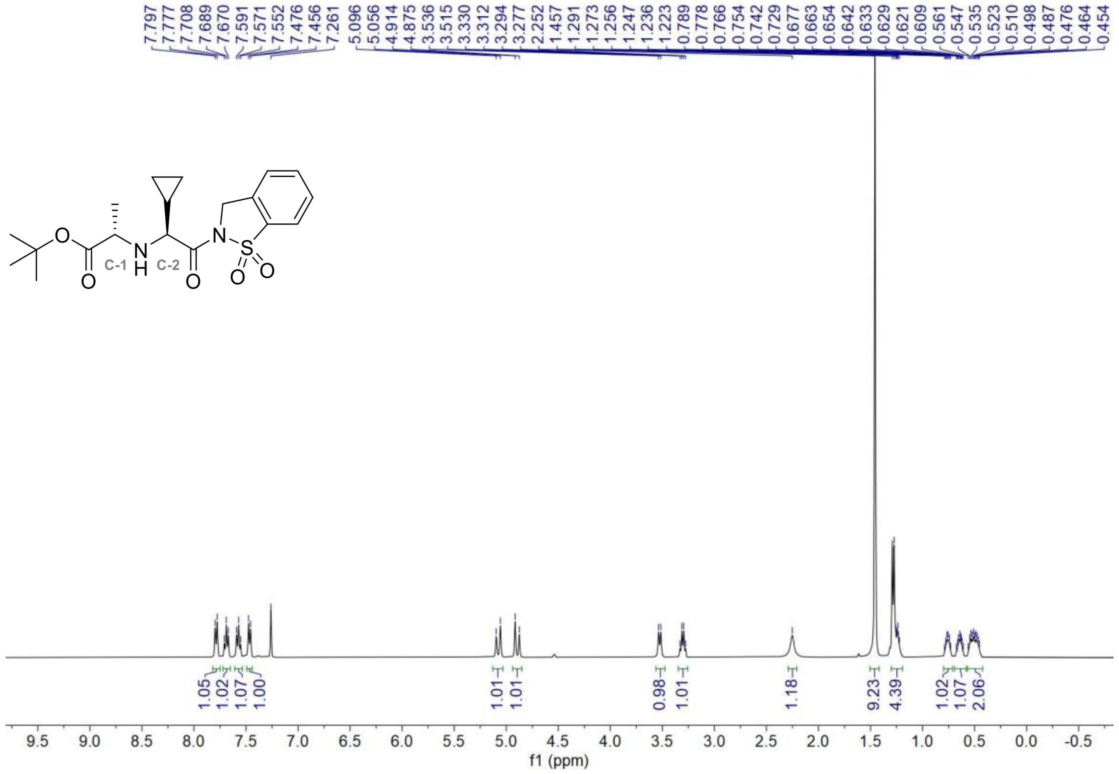

^{13}C NMR (101 MHz, $CDCl_3$) – ($S_{C\text{-}1}$, $S_{C\text{-}2}$)-**13a**

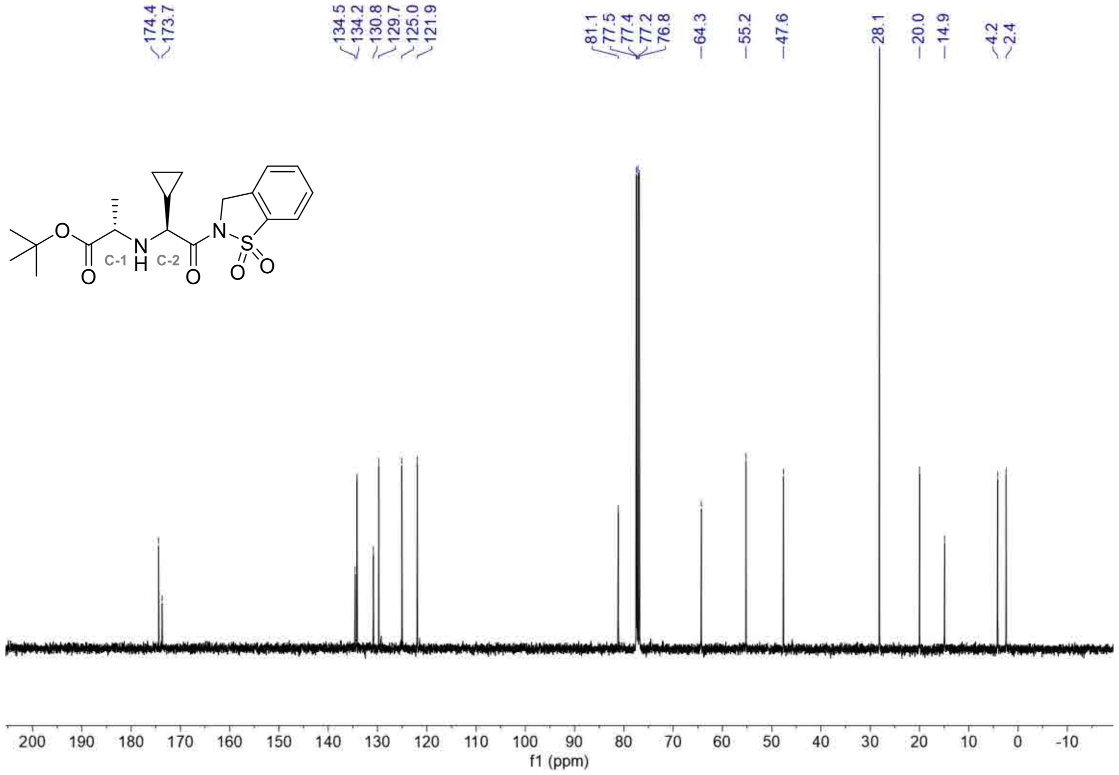

^{1}H NMR (400 MHz, $CDCl_3$) – ($S_{C\text{-}1}$, $R_{C\text{-}2}$)-**13b**

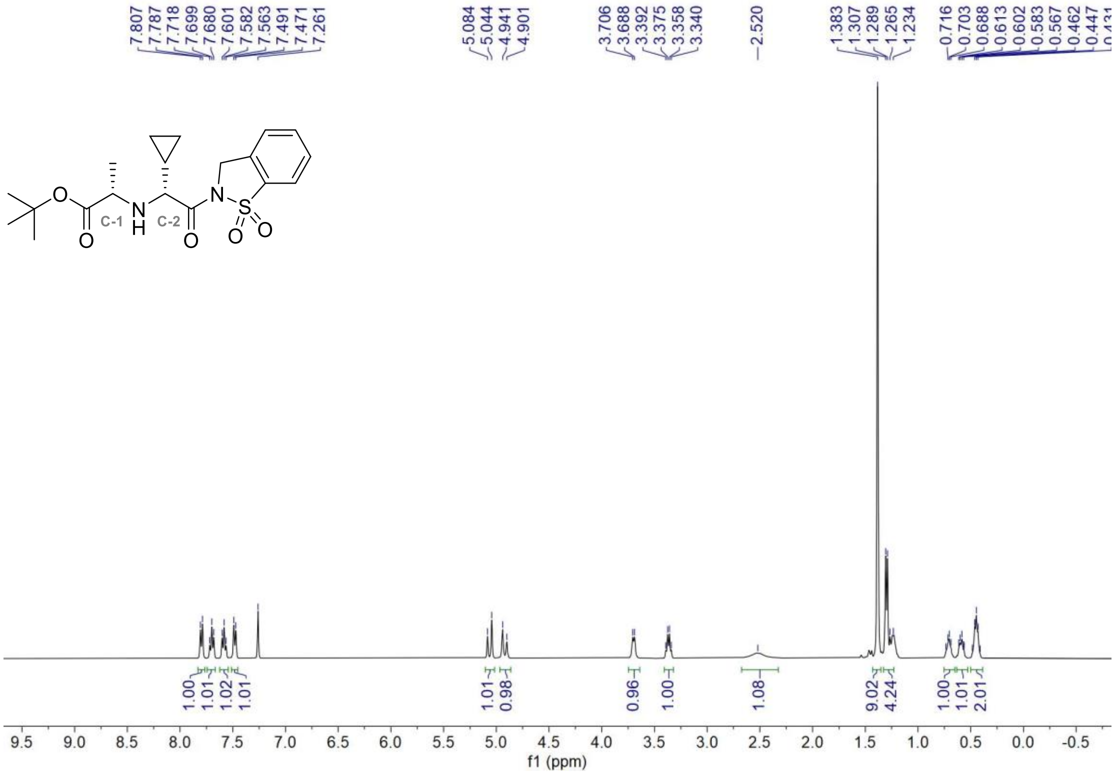

^{13}C NMR (101 MHz, $CDCl_3$) – ($S_{C\text{-}1}$, $R_{C\text{-}2}$)-**13b**

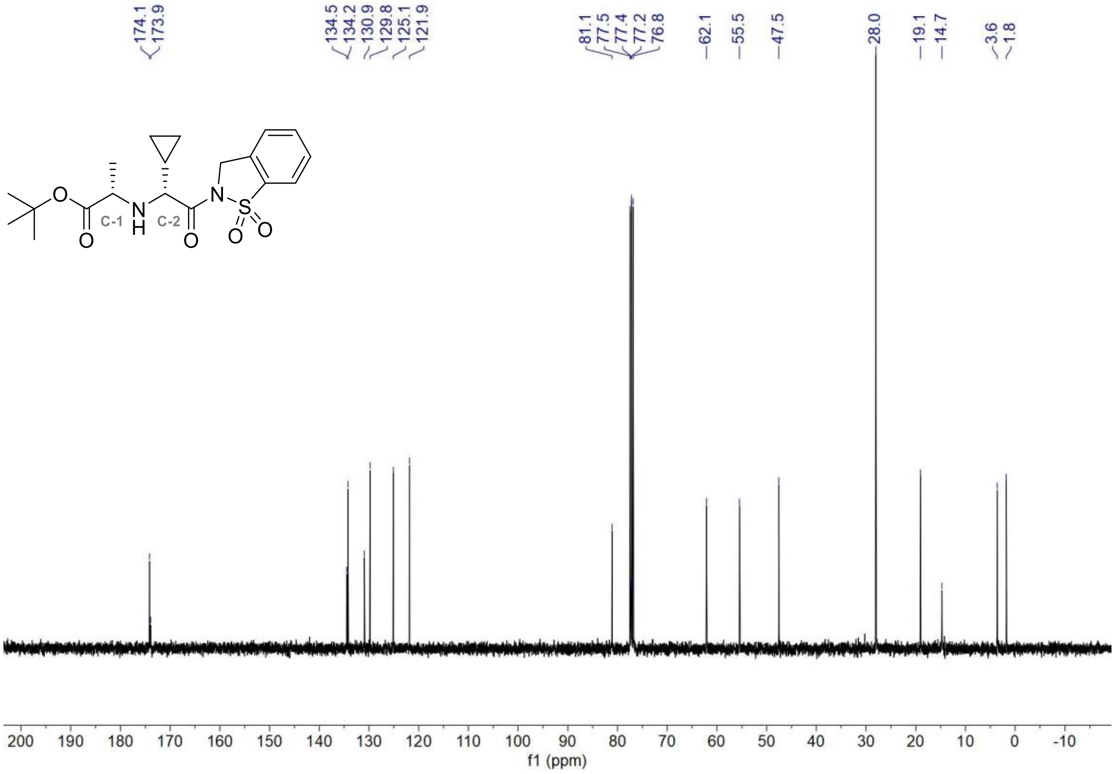

^{1}H NMR (400 MHz, $CDCl_3$) – (R_{C-1}, S_{C-2})-**14a**

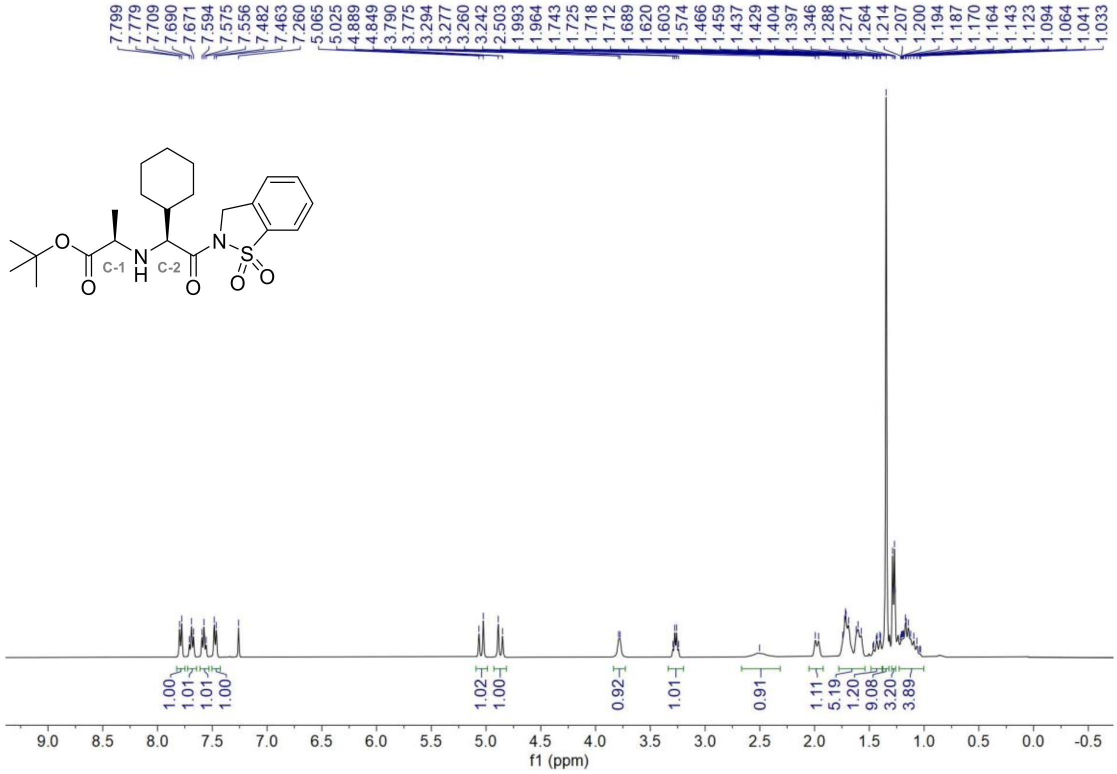

^{13}C NMR (101 MHz, $CDCl_3$) – (R_{C-1}, S_{C-2})-**14a**

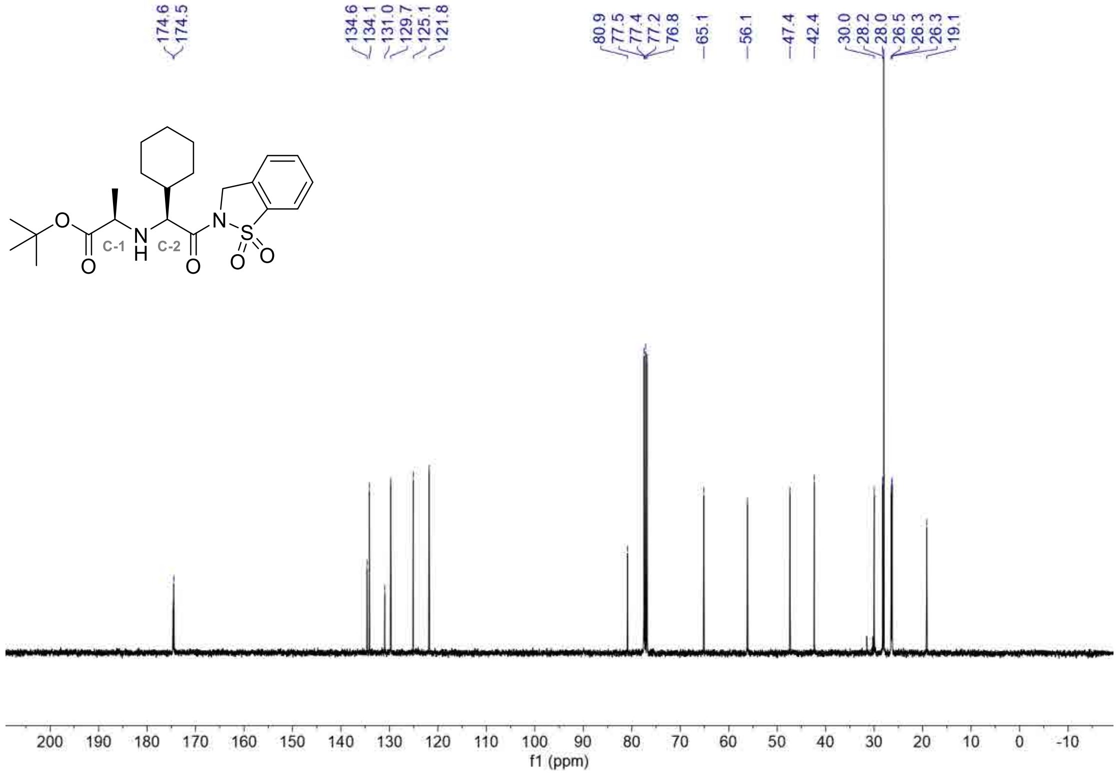

^{1}H NMR (400 MHz, CDCl$_3$) – ($R_{C\text{-}1}$, $R_{C\text{-}2}$)-**14b**

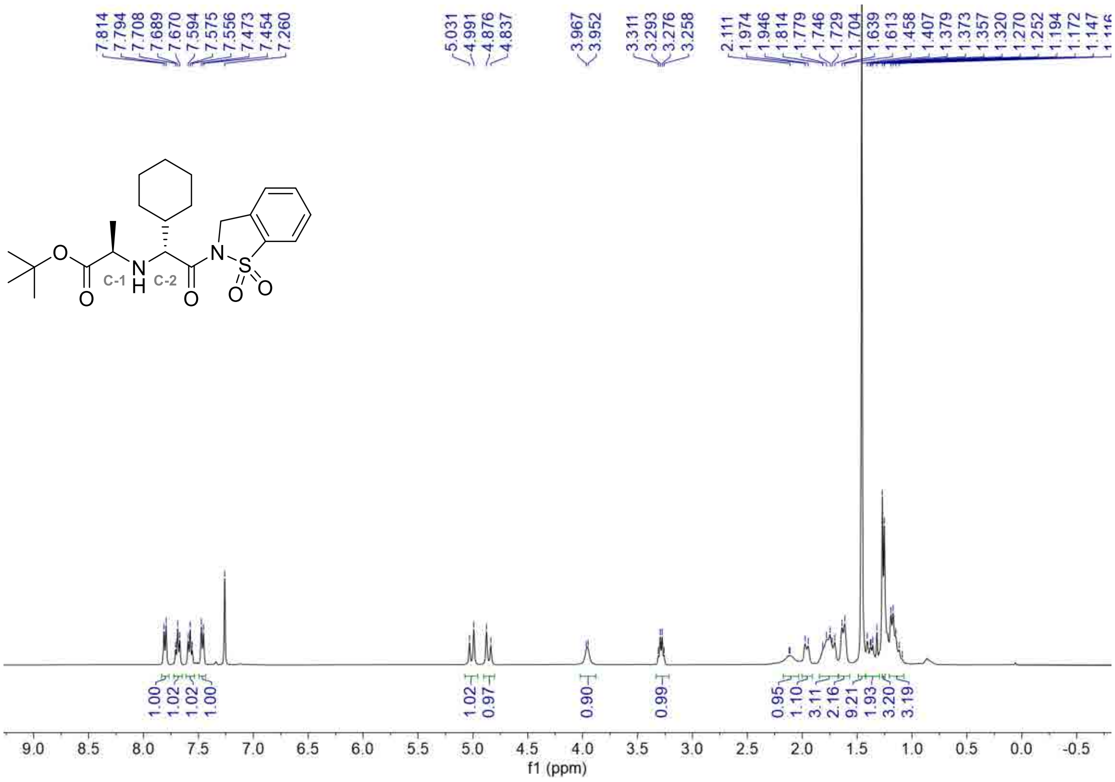

^{13}C NMR (101 MHz, CDCl$_3$) – ($R_{C\text{-}1}$, $R_{C\text{-}2}$)-**14b**

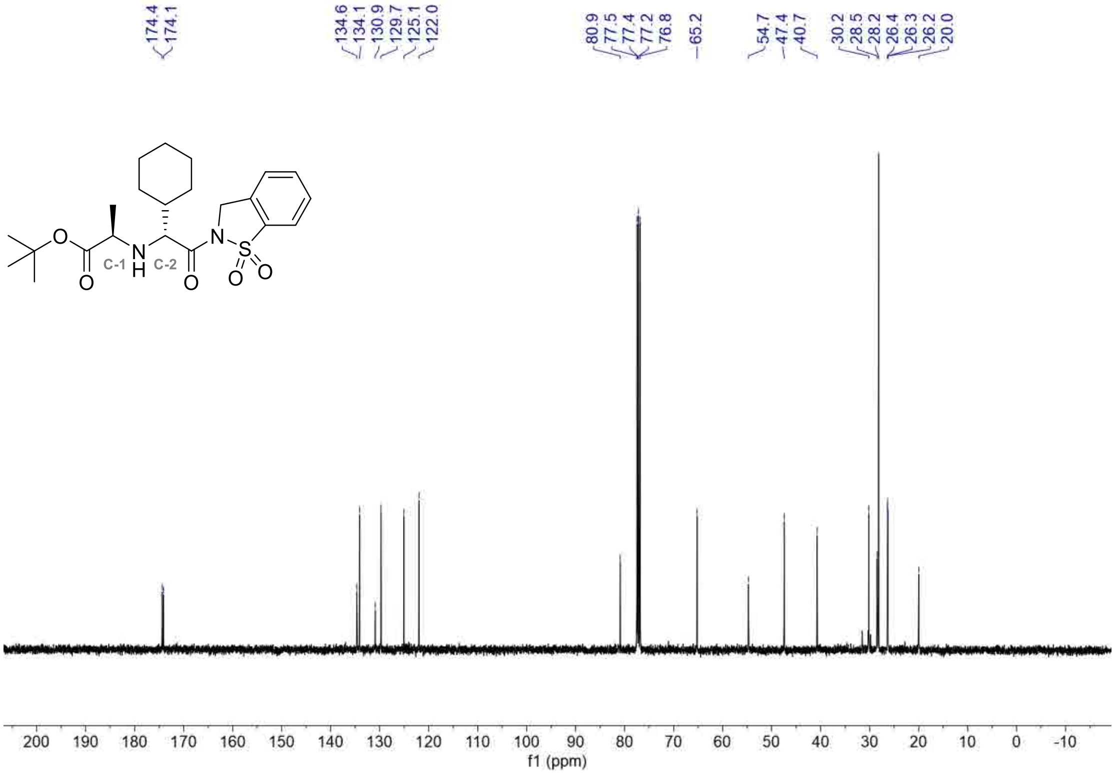

^{1}H NMR (400 MHz, $CDCl_3$) – ($R_{C\text{-}1}$, $S_{C\text{-}2}$)-**15a**

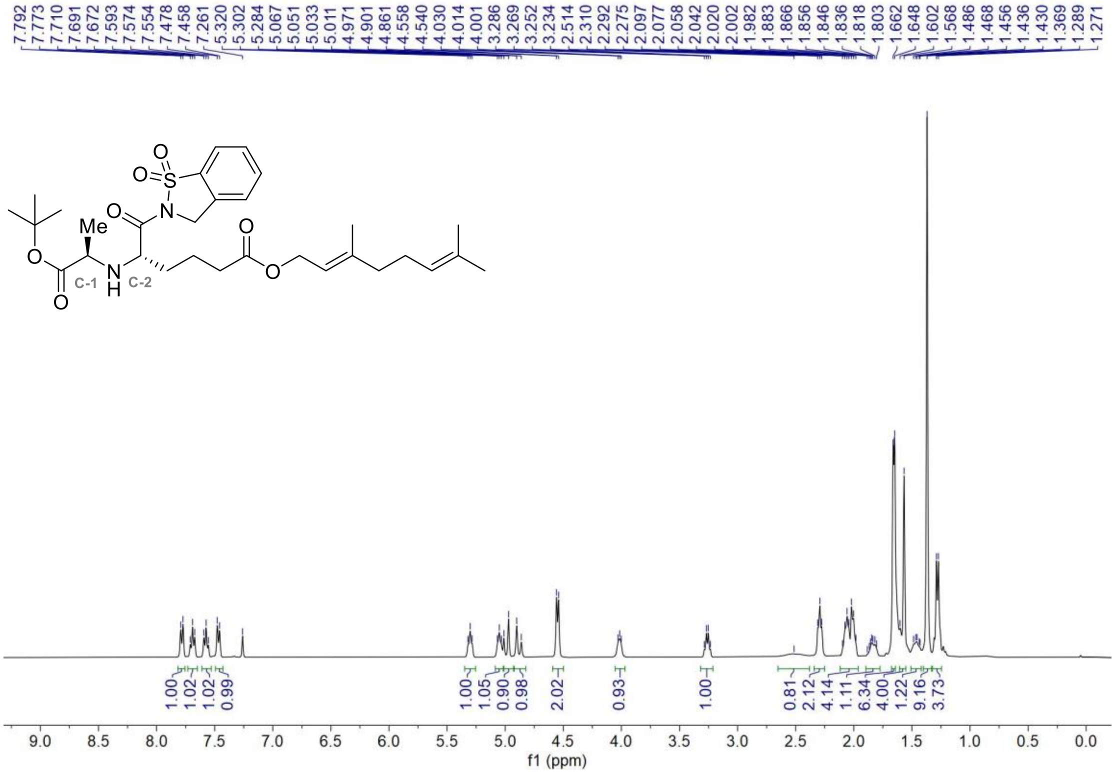

^{13}C NMR (101 MHz, $CDCl_3$) – ($R_{C\text{-}1}$, $S_{C\text{-}2}$)-**15a**

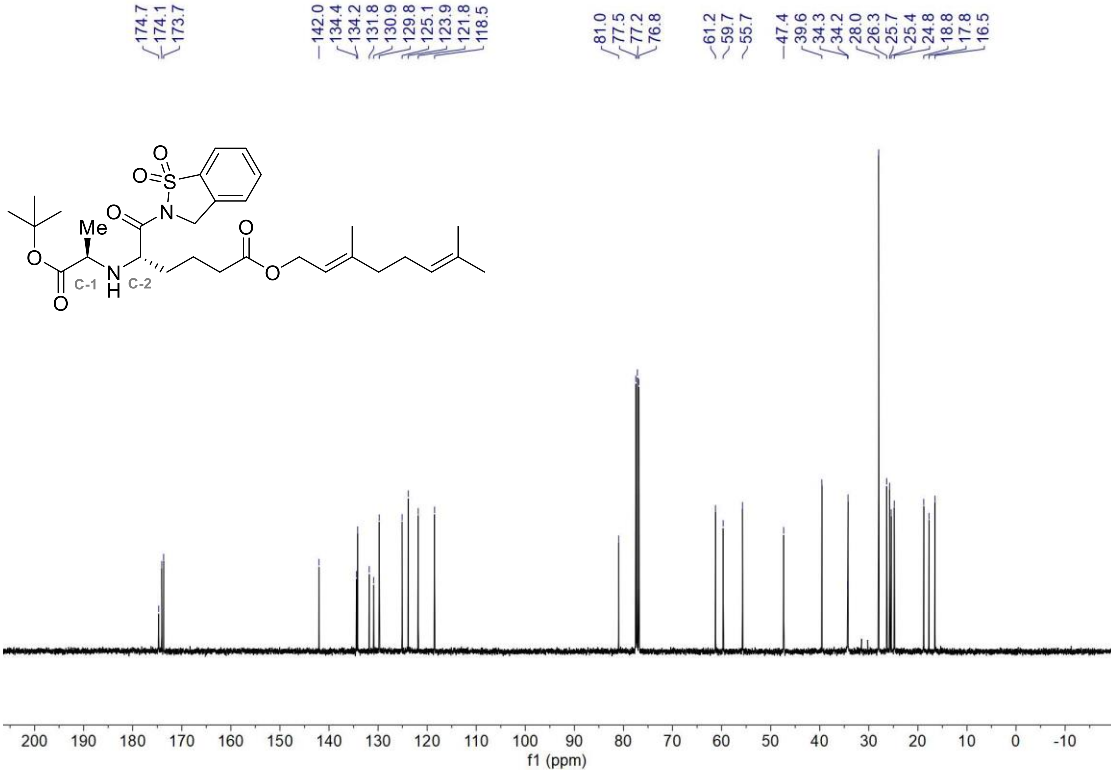

^{1}H NMR (400 MHz, $CDCl_3$) – ($R_{C\text{-}1}$, $R_{C\text{-}2}$)-**15b**

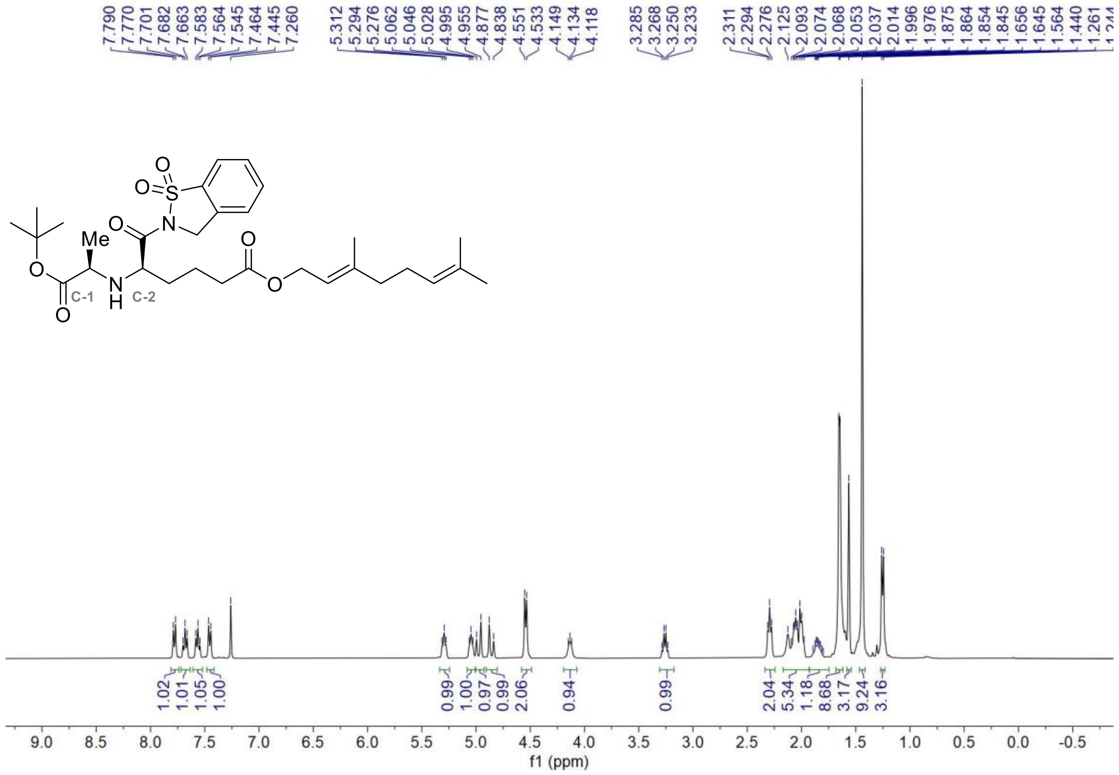

^{13}C NMR (101 MHz, $CDCl_3$) – ($R_{C\text{-}1}$, $R_{C\text{-}2}$)-**15b**

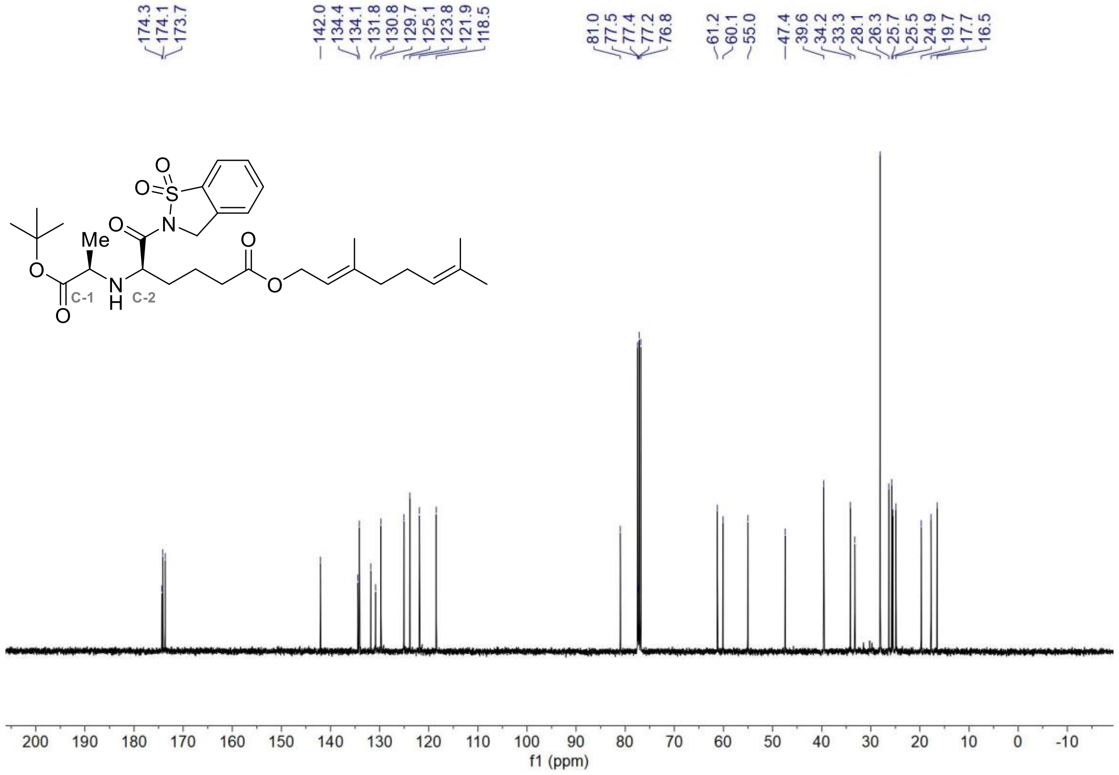

^{1}**H NMR** (400 MHz, $CDCl_3$) – (R_{C-1}, S_{C-2})-**16a**

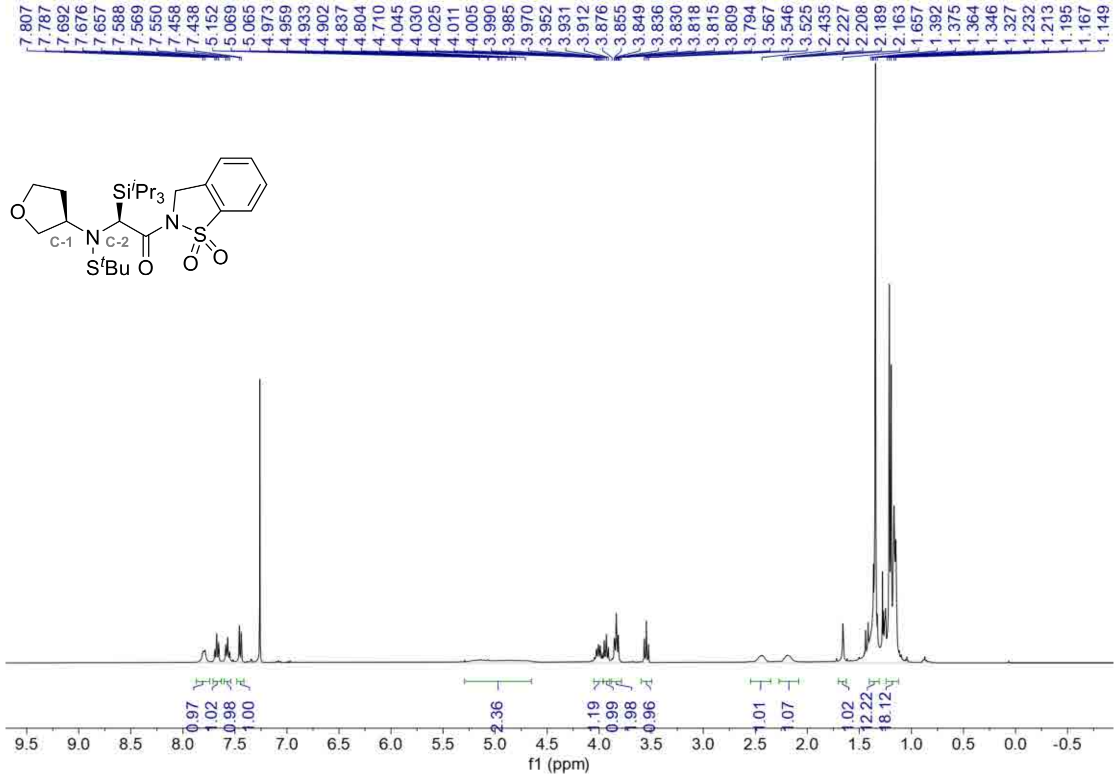

^{13}**C NMR** (101 MHz, $CDCl_3$) – (R_{C-1}, S_{C-2})-**16a**

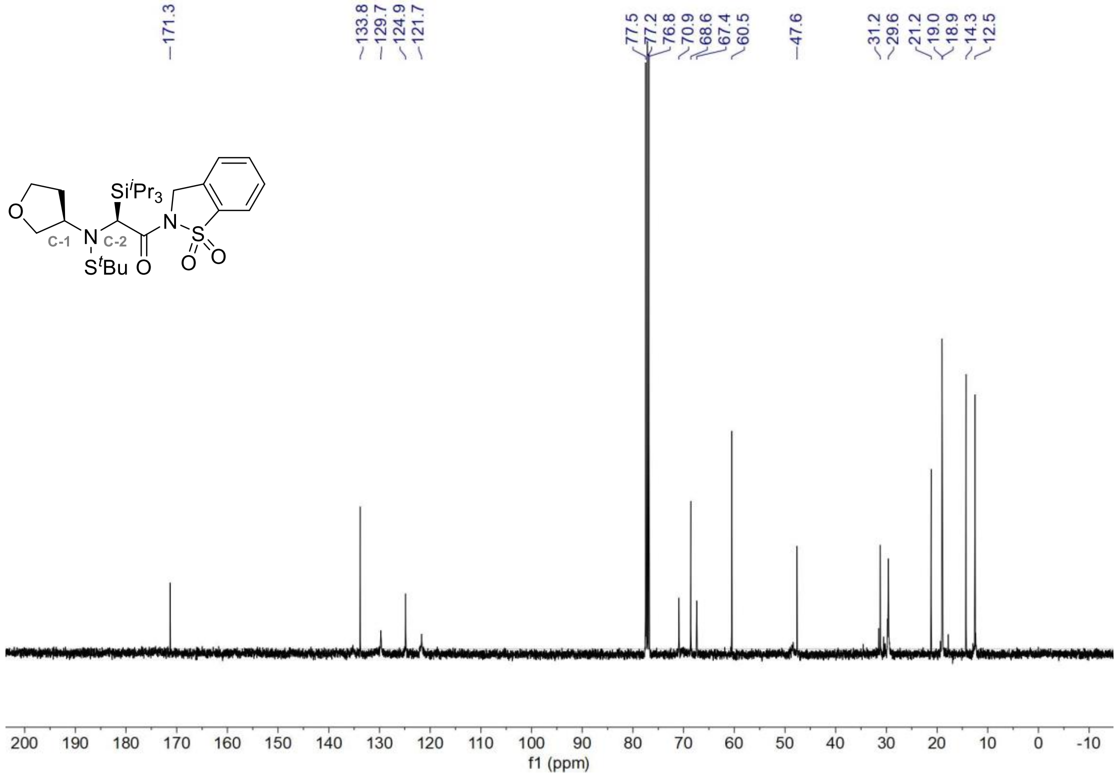

^{1}H NMR (400 MHz, $CDCl_3$) – (R_{C-1}, S_{C-2})-**17a**

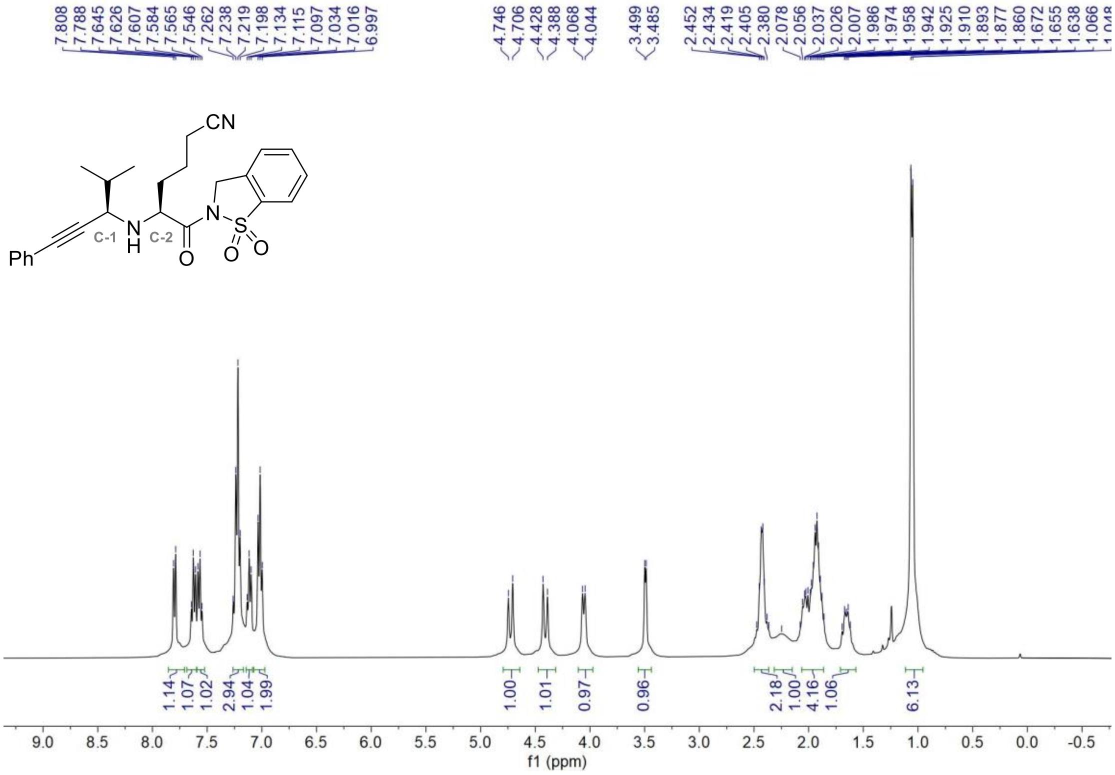

^{13}C NMR (101 MHz, $CDCl_3$) – (R_{C-1}, S_{C-2})-**17a**

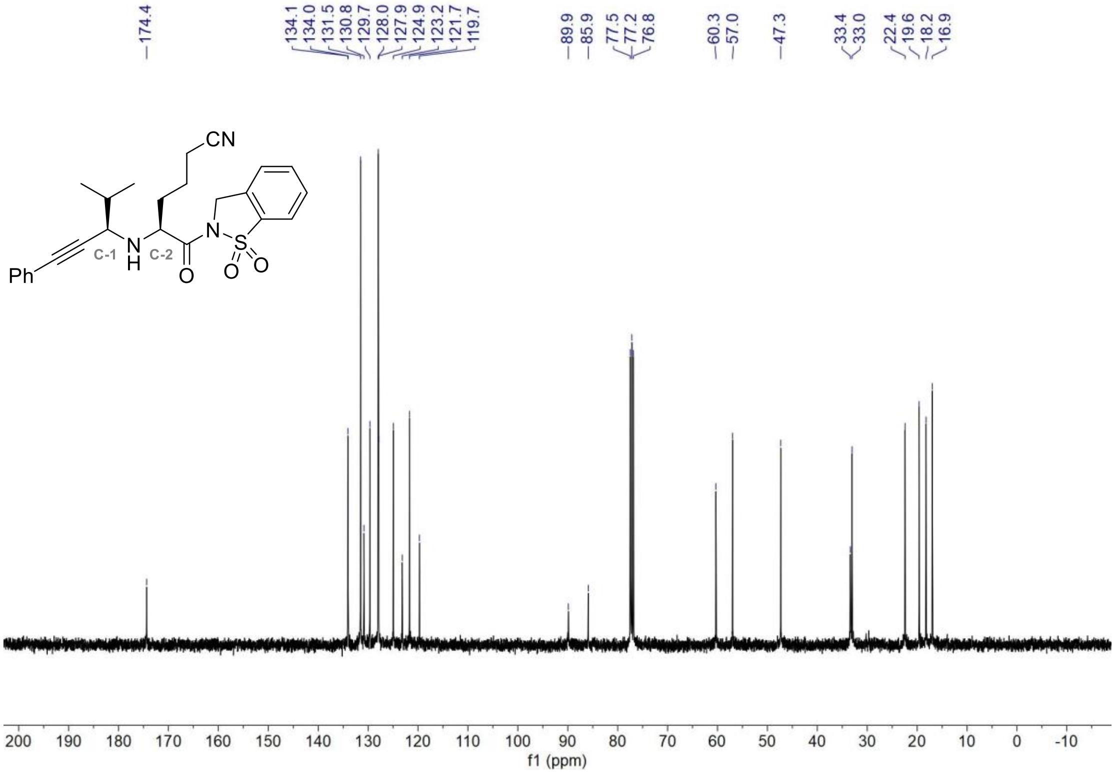

^{1}**H NMR** (400 MHz, $CDCl_3$) – (*S*$_{S(IV)}$, *S*$_{C-1}$)-**S17b**

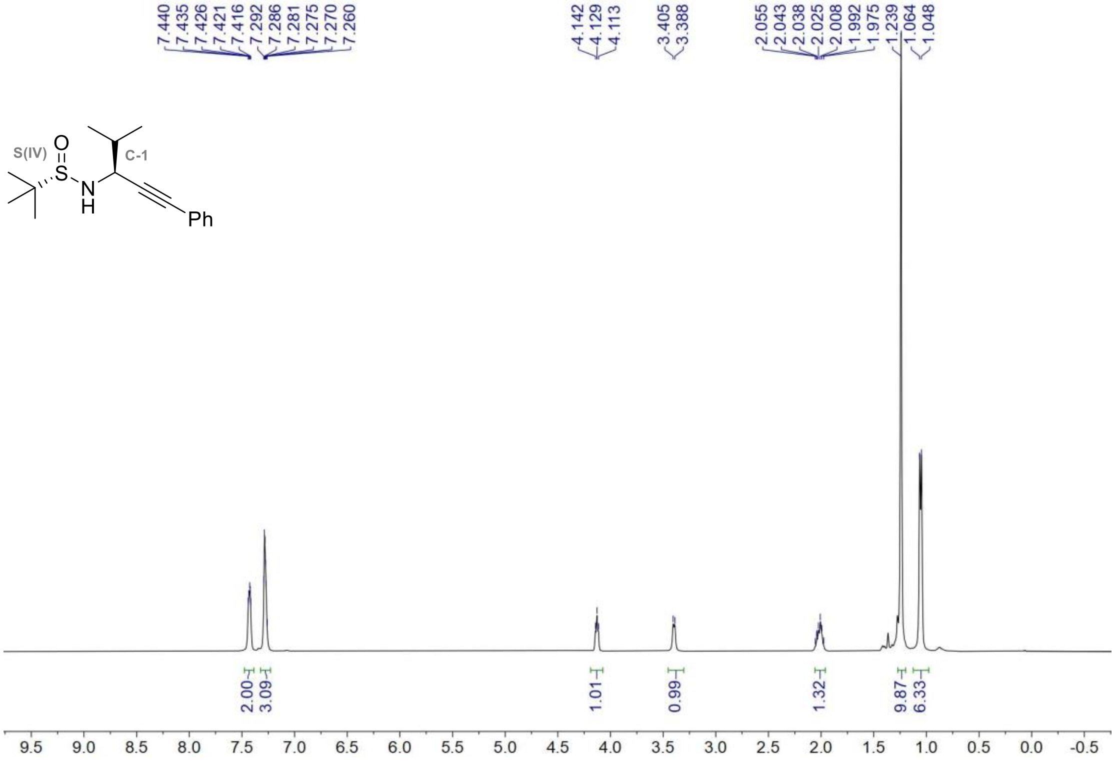

^{13}**C NMR** (101 MHz, $CDCl_3$) – (*S*$_{S(IV)}$, *S*$_{C-1}$)-**S17b**

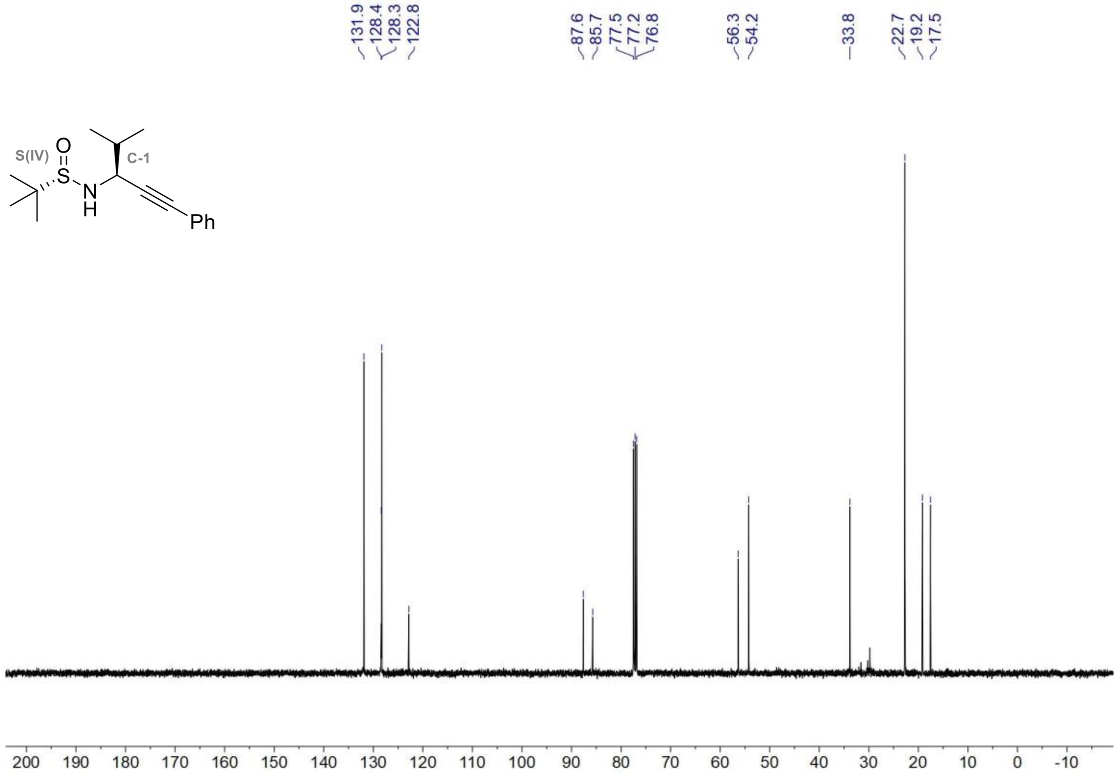

^{1}H NMR (400 MHz, $CDCl_3$) – (S_{C-1}, R_{C-2})-**17b**

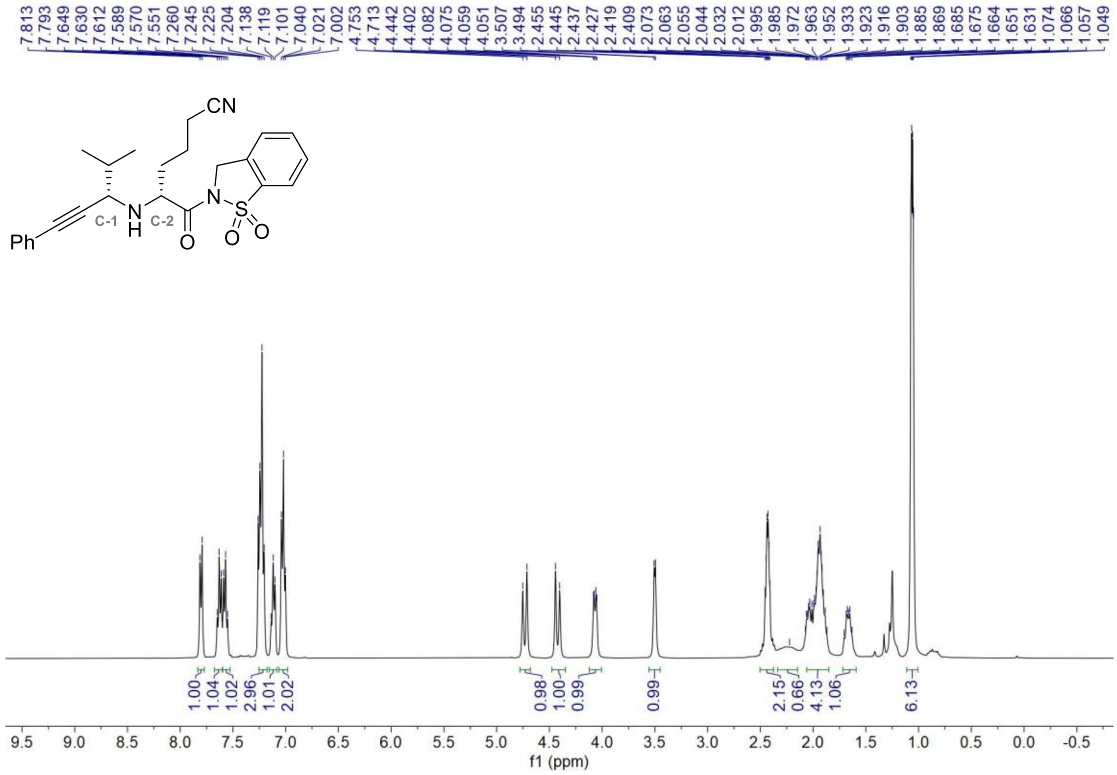

^{13}C NMR (101 MHz, $CDCl_3$) – (S_{C-1}, R_{C-2})-**17b**

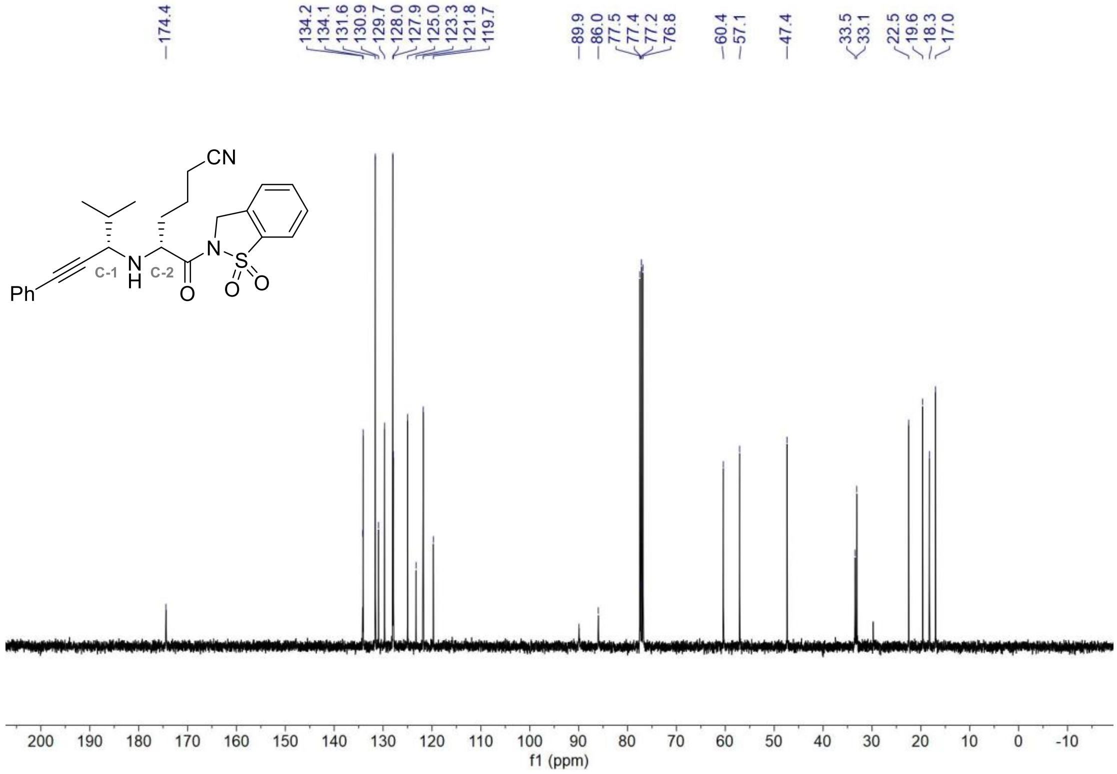

^{1}H NMR (400 MHz, $CDCl_3$) – (S_{C-1}, S_{C-2})-**18a**

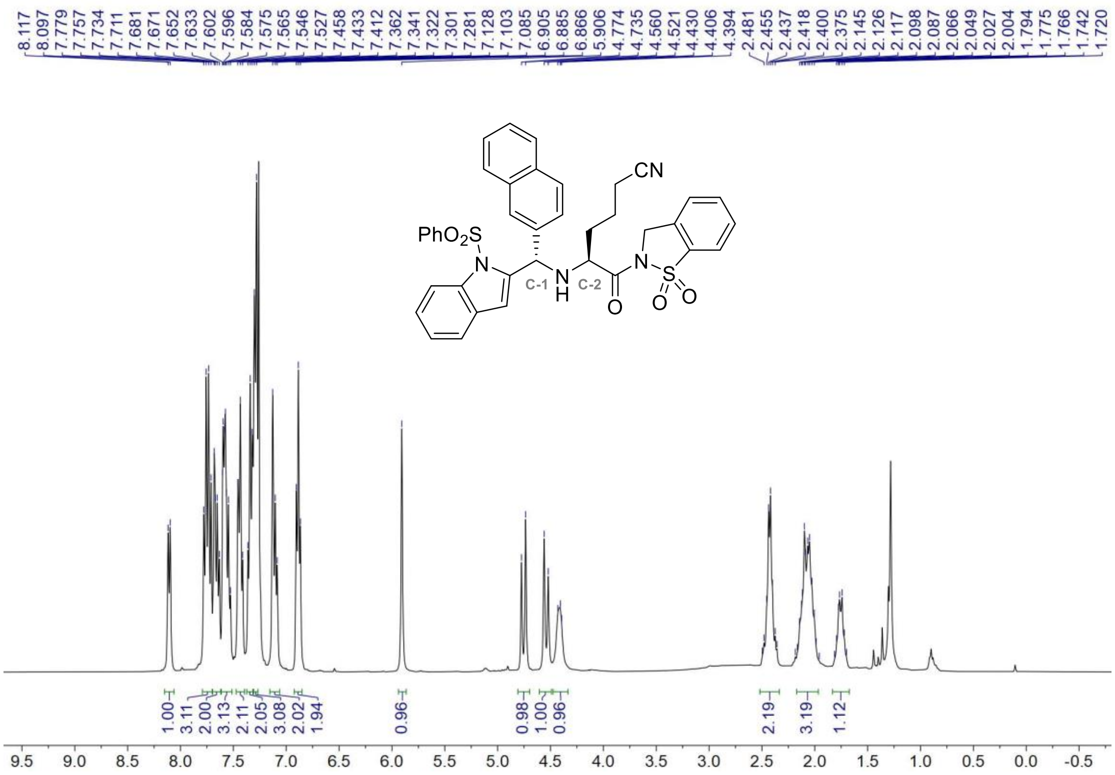

^{13}C NMR (101 MHz, $CDCl_3$) – (S_{C-1}, S_{C-2})-**18a**

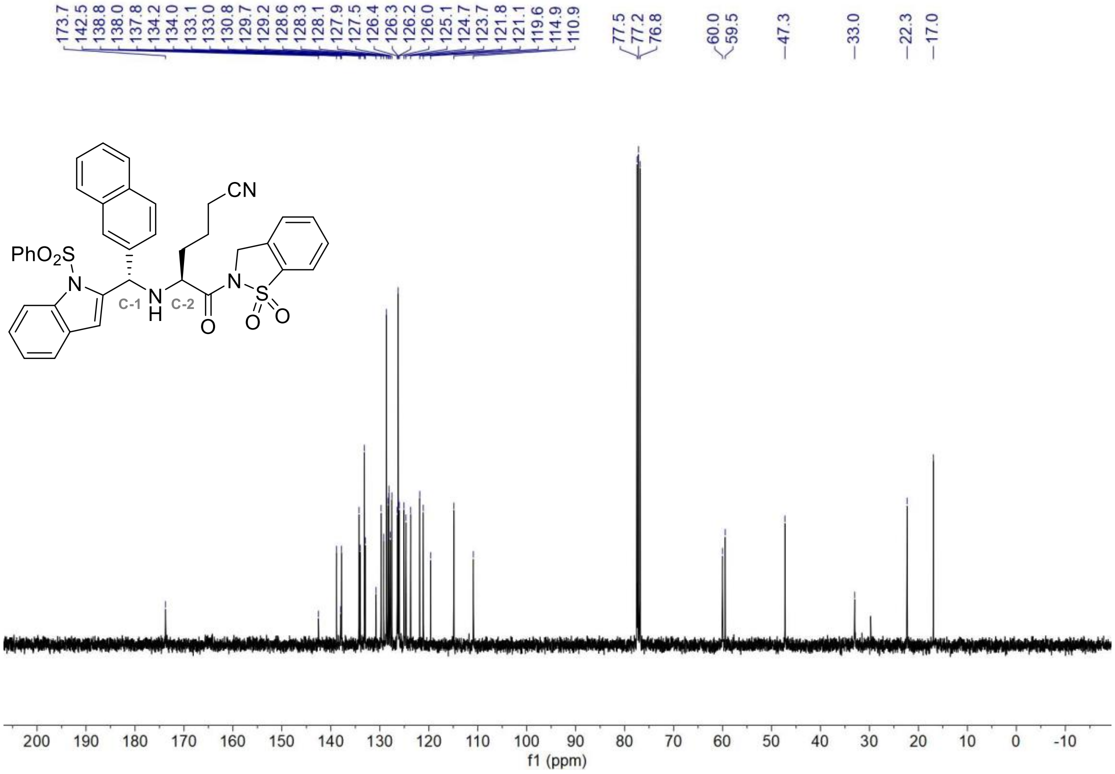

^{1}H NMR (400 MHz, $CDCl_3$) – ($S_{S(IV)}$, R_{C-1})-**S18b**

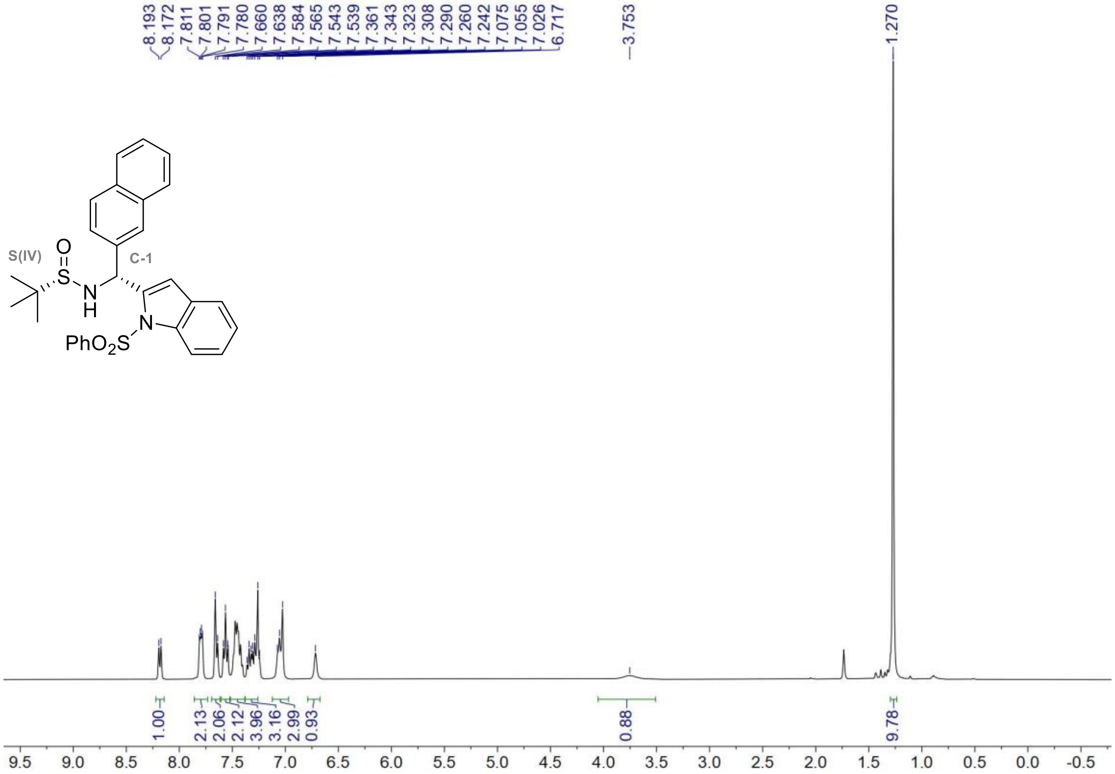

^{13}C NMR (101 MHz, $CDCl_3$) – ($S_{S(IV)}$, R_{C-1})-**S18b**

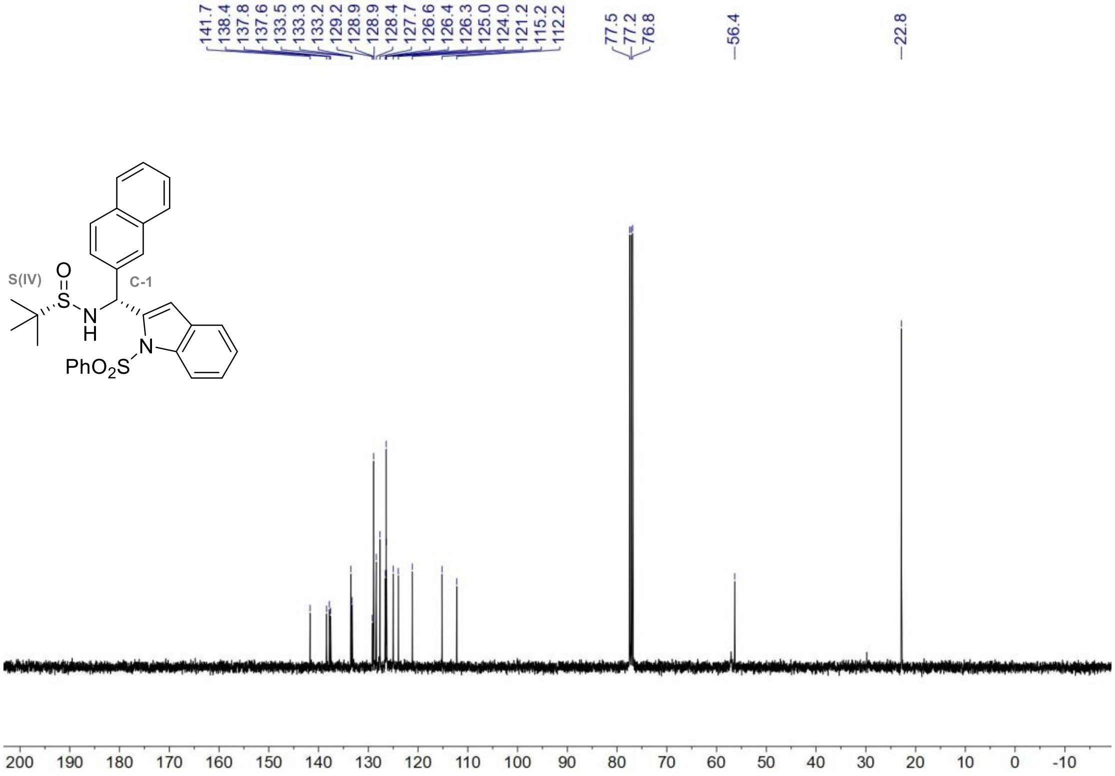

^{1}H NMR (400 MHz, $CDCl_3$) – ($R_{C\text{-}1}$, $R_{C\text{-}2}$)-**18b**

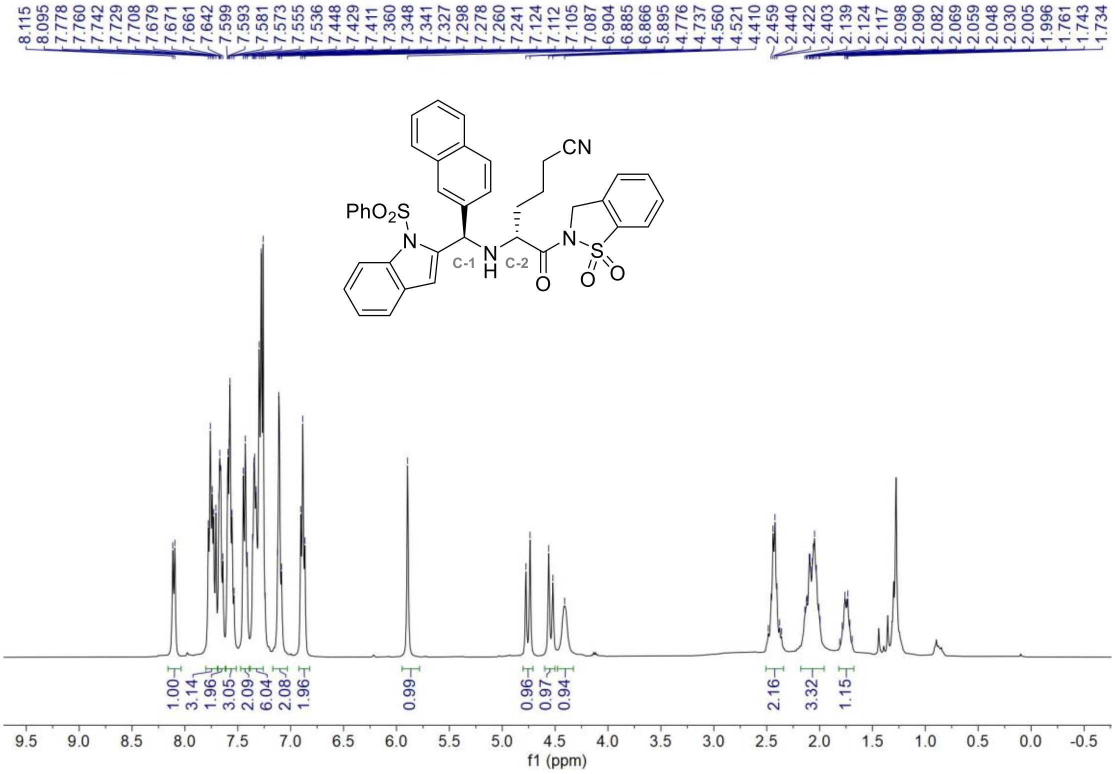

^{13}C NMR (101 MHz, $CDCl_3$) – ($R_{C\text{-}1}$, $R_{C\text{-}2}$)-**18b**

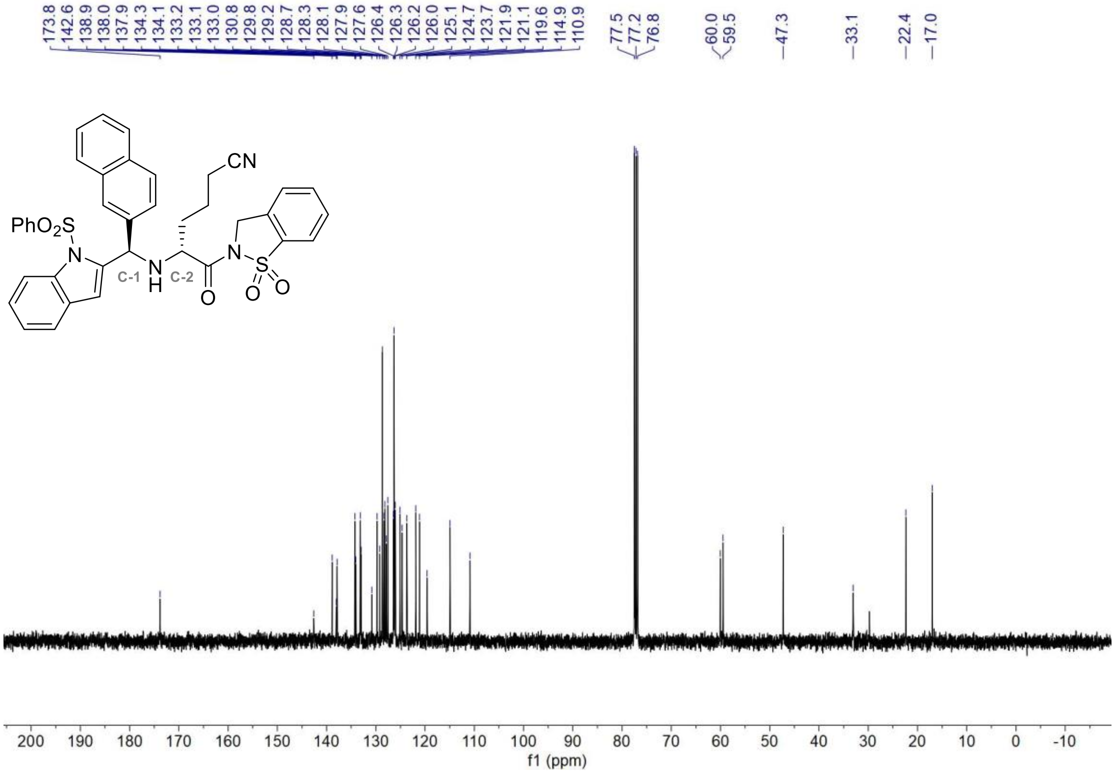

^{1}H NMR (400 MHz, $CDCl_3$) – ($R_{S(IV)}$, $R_{C\text{-}1}$)-**S19a**

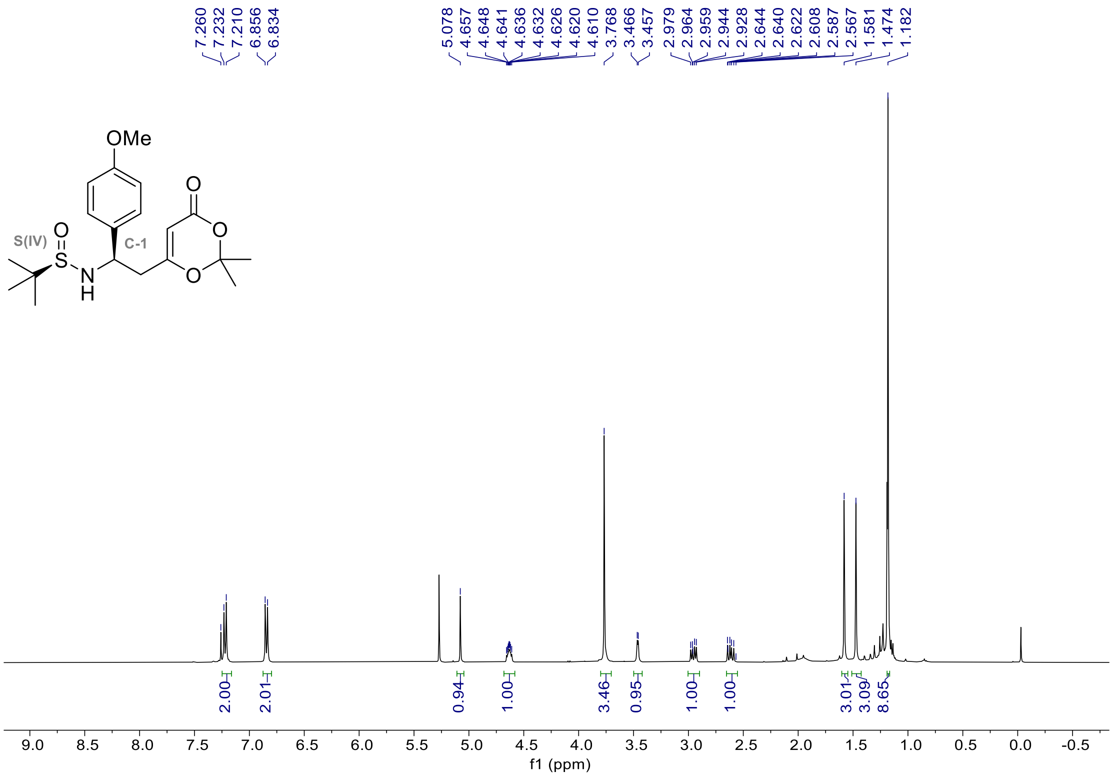

^{13}C NMR (101 MHz, $CDCl_3$) – ($R_{S(IV)}$, $R_{C\text{-}1}$)-**S19a**

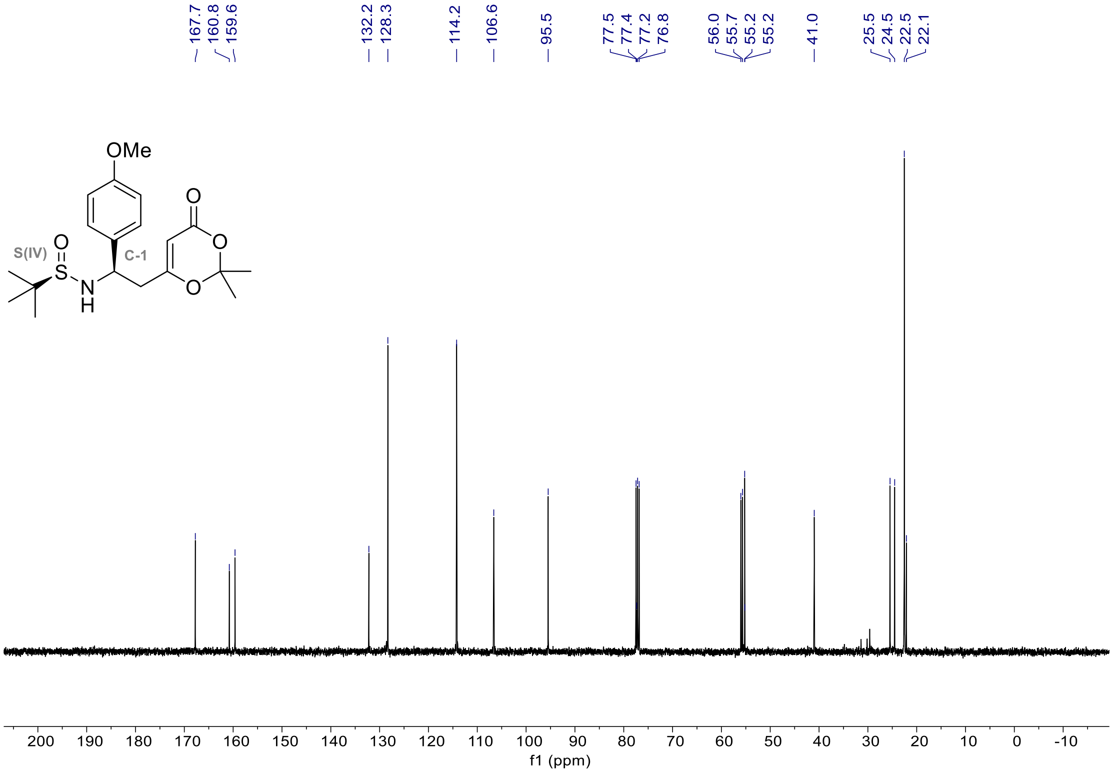

^{1}H NMR (400 MHz, CDCl$_3$) – (R_{C-1}, S_{C-2})-**19a**

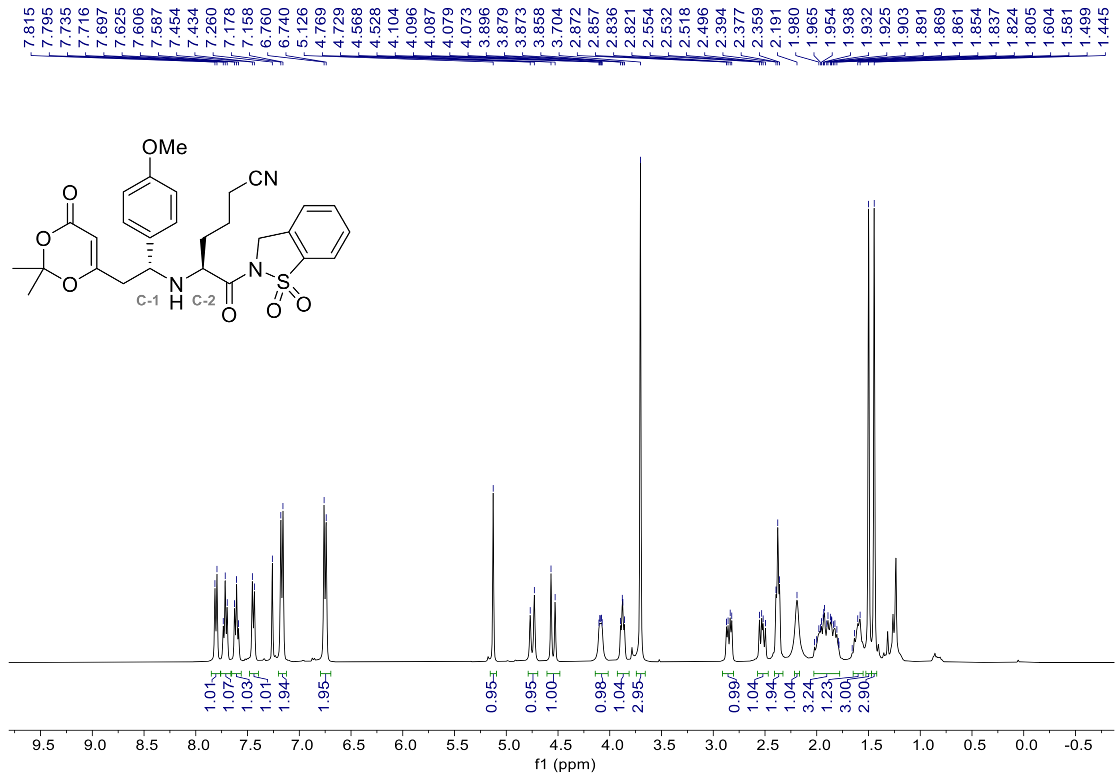

^{13}C NMR (101 MHz, CDCl$_3$) – (R_{C-1}, S_{C-2})-**19a**

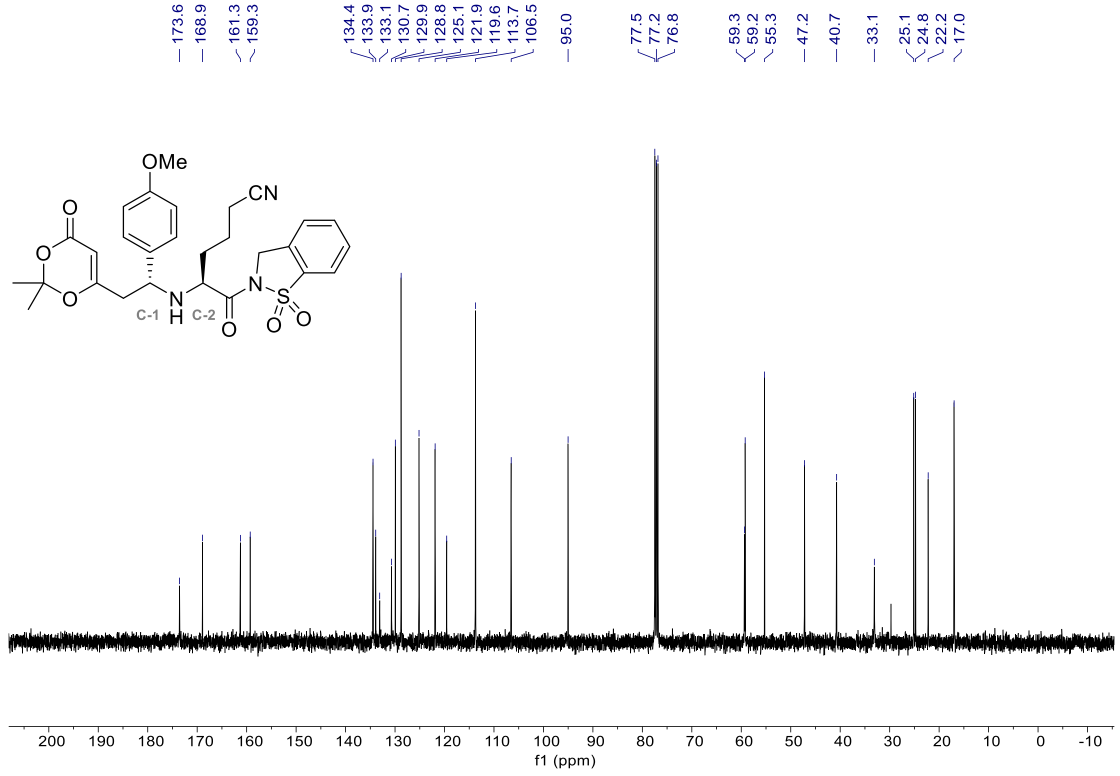

^{1}H NMR (400 MHz, CDCl$_3$) – ($S_{C\text{-}1}$, $R_{C\text{-}2}$)-**19b**

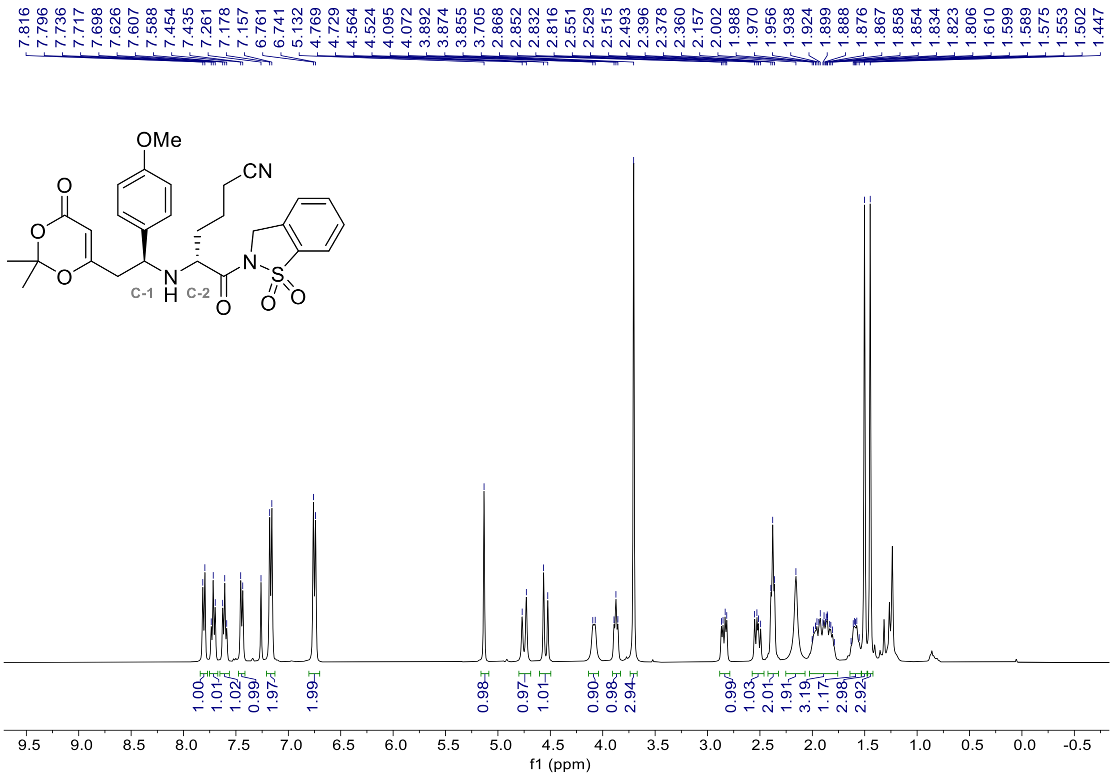

^{13}C NMR (101 MHz, CDCl$_3$) – ($S_{C\text{-}1}$, $R_{C\text{-}2}$)-**19b**

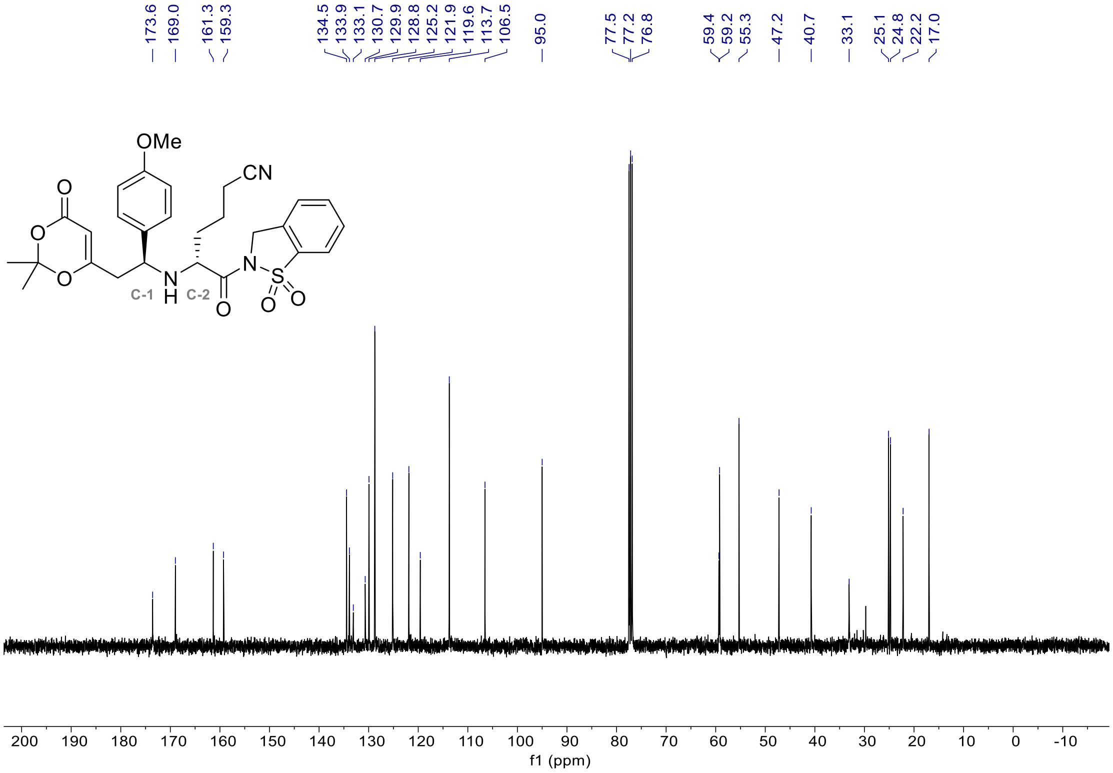

^{1}H NMR (400 MHz, $CDCl_3$) – (R_{C-1}, S_{C-2})-**20a**

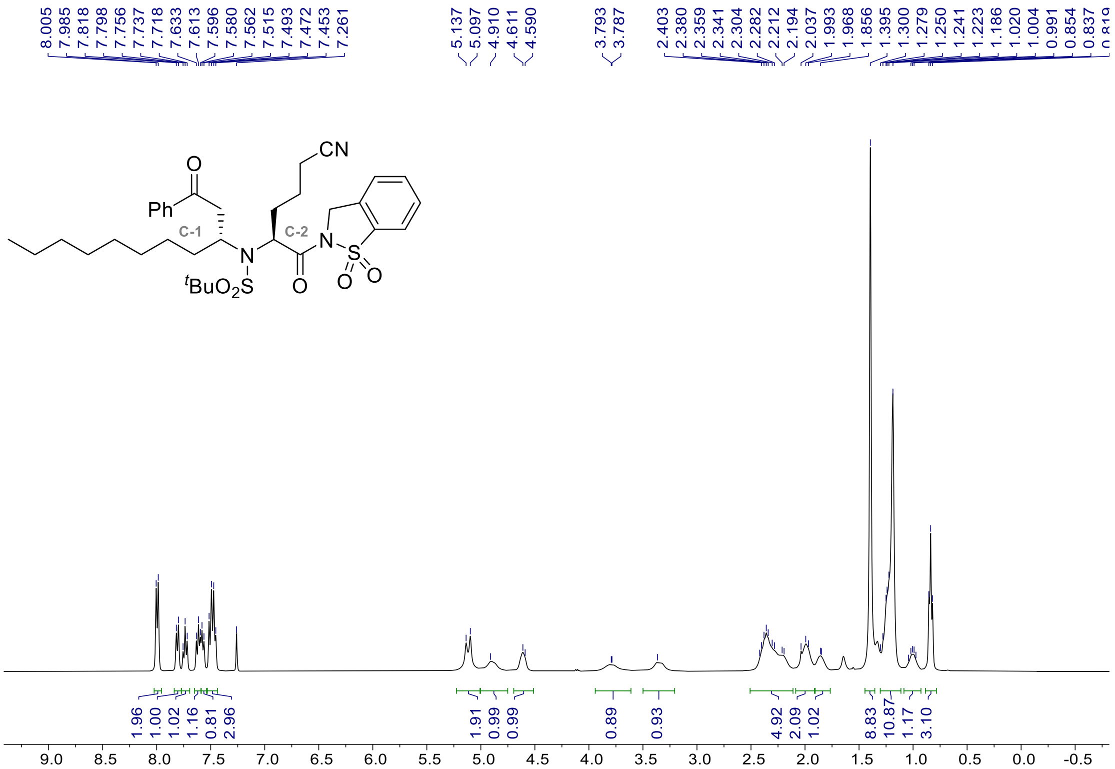

^{13}C NMR (101 MHz, $CDCl_3$) – (R_{C-1}, S_{C-2})-**20a**

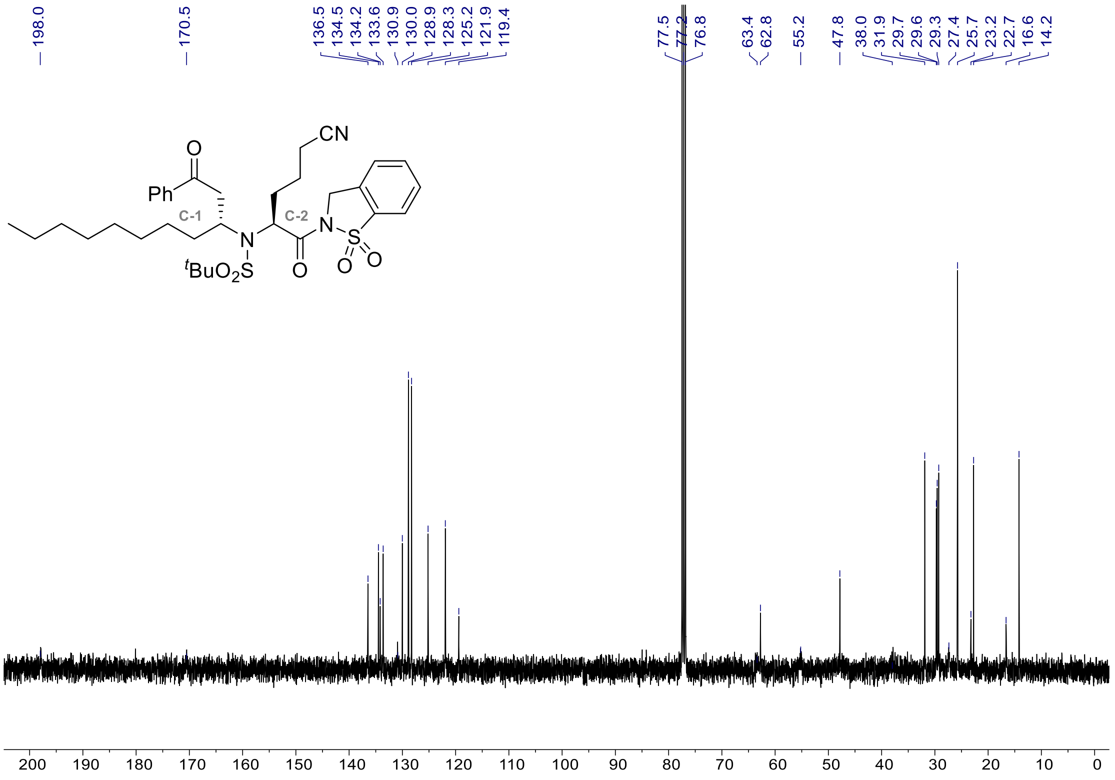

^{1}H NMR (400 MHz, CDCl$_3$) – ($S_{S(IV)}$, S_{C-1})-**S20b**

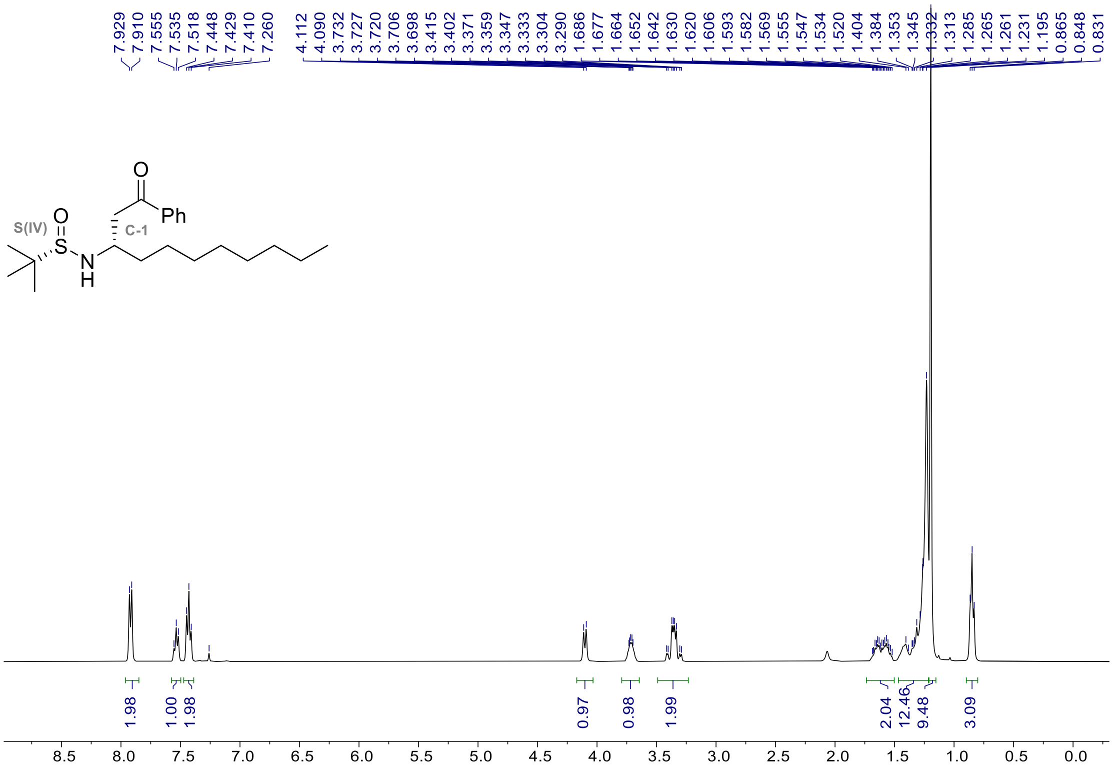

^{13}C NMR (101 MHz, CDCl$_3$) – ($S_{S(IV)}$, S_{C-1})-**S20b**

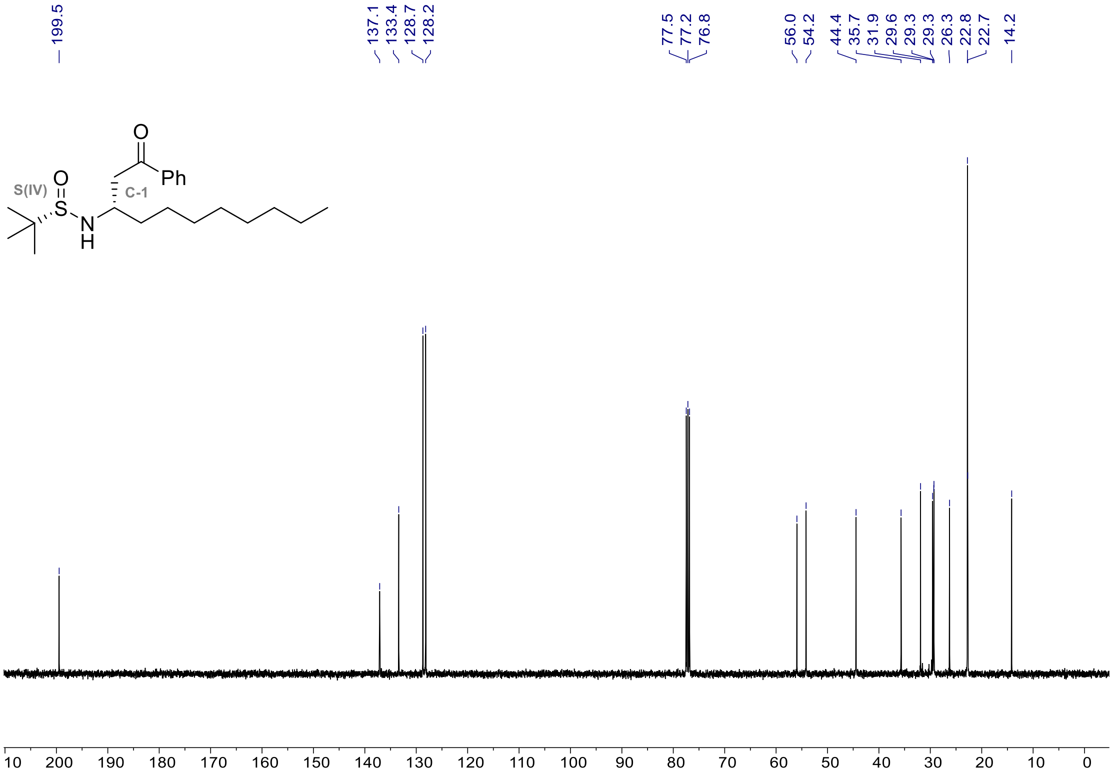

^{1}H NMR (400 MHz, $CDCl_3$) – ($S_{C\text{-}1}$, $R_{C\text{-}2}$)-**20b**

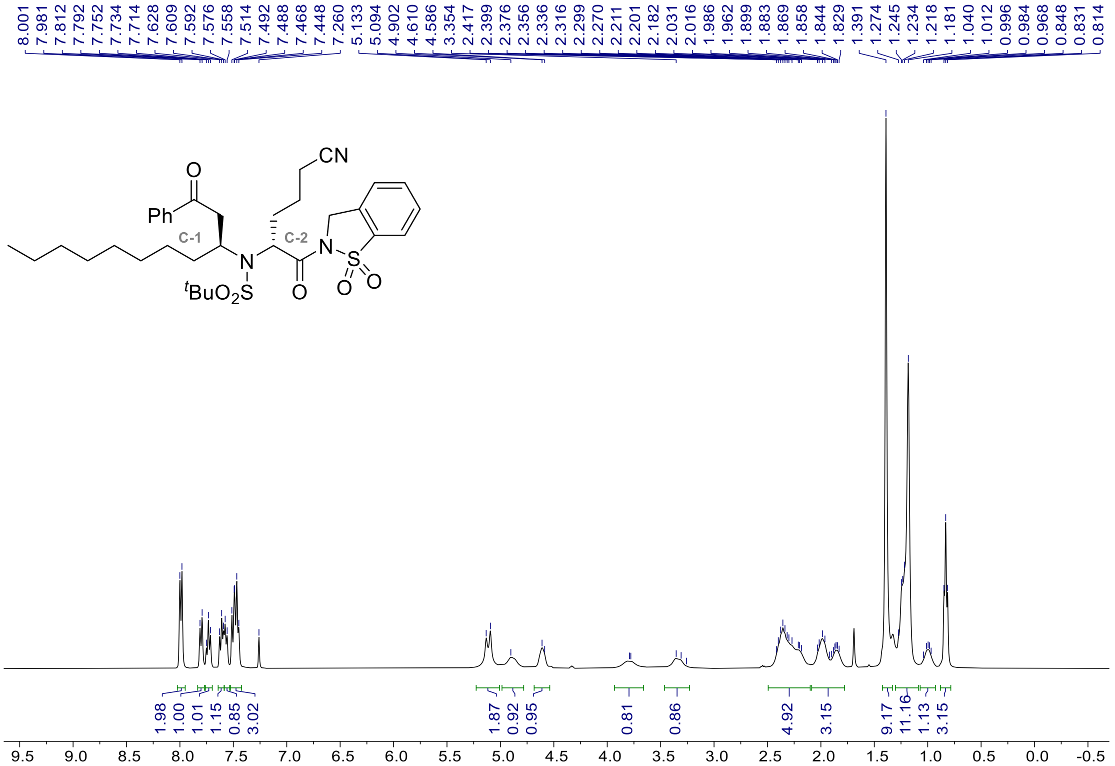

^{13}C NMR (101 MHz, $CDCl_3$) – ($S_{C\text{-}1}$, $R_{C\text{-}2}$)-**20b**

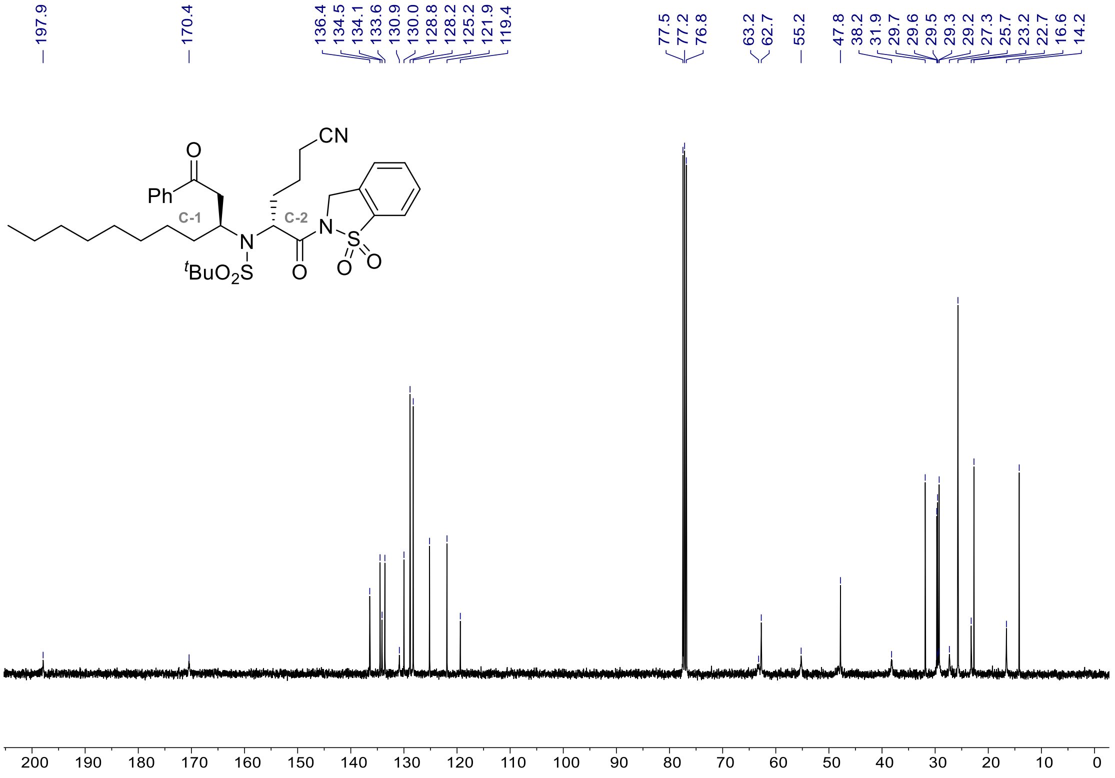

^{1}H NMR (400 MHz, $CDCl_3$) – (S_{C-1}, S_{C-2})-**21a**

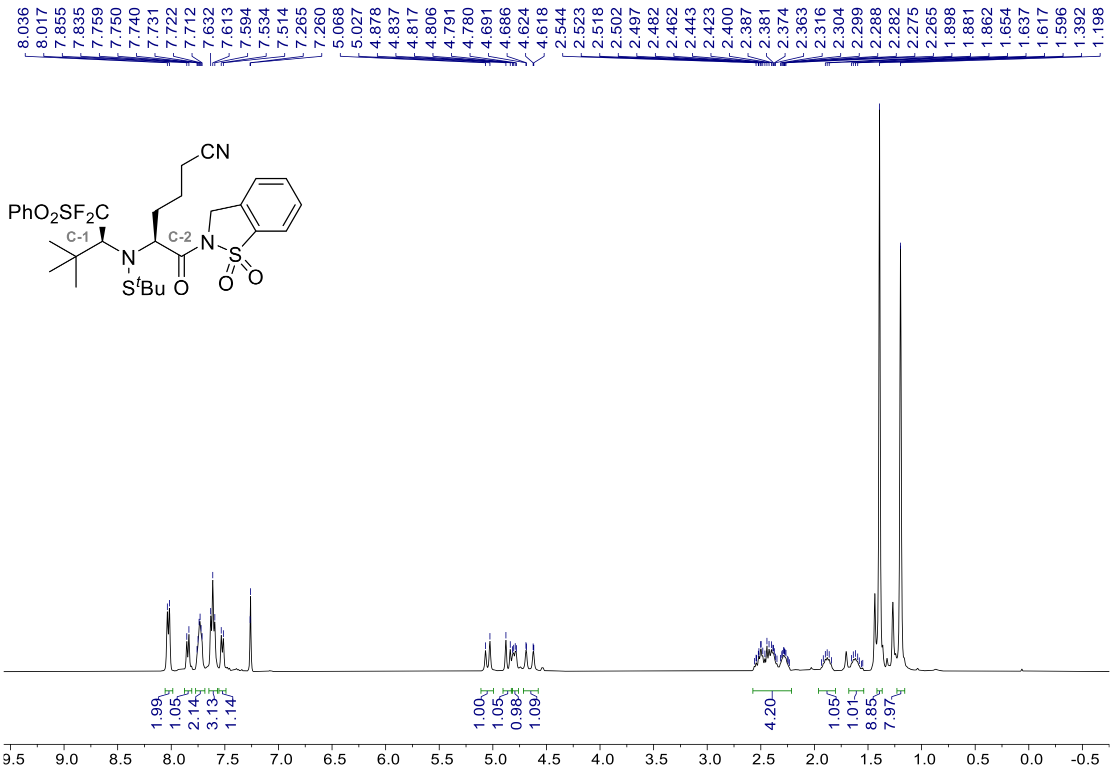

^{13}C NMR (101 MHz, $CDCl_3$) – (S_{C-1}, S_{C-2})-**21a**

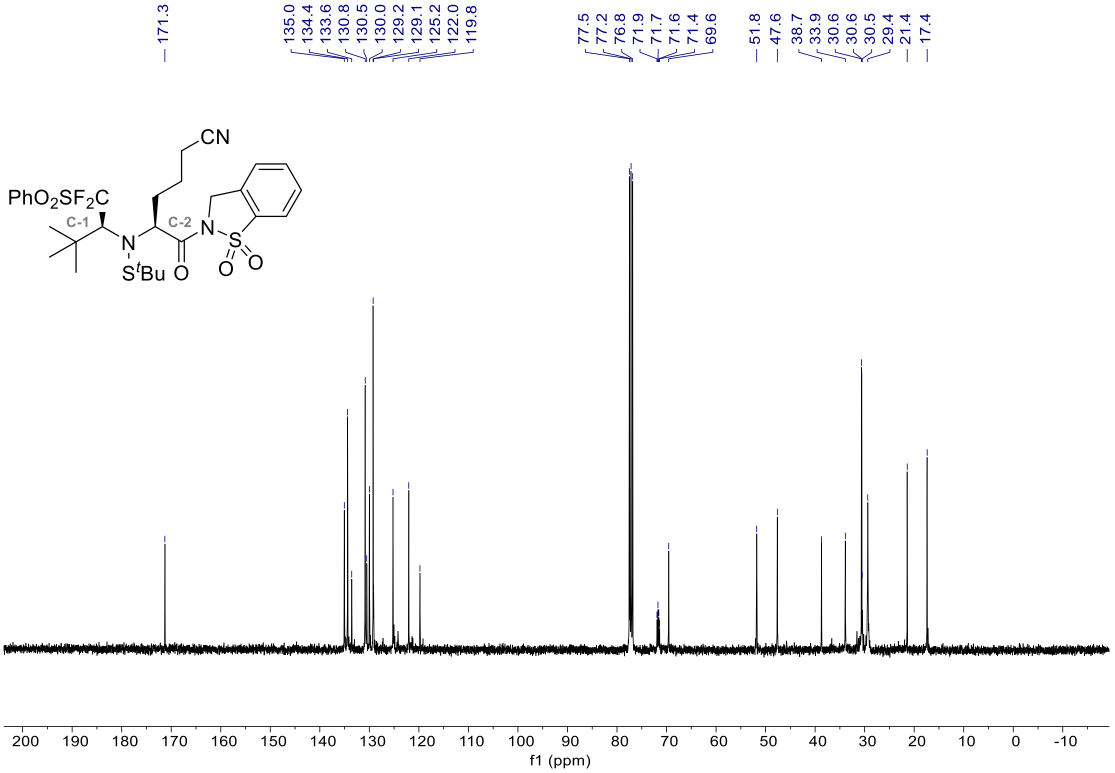

^{19}F NMR (376 MHz, $CDCl_3$) – (*S*$_{C\text{-}1}$, *S*$_{C\text{-}2}$)-**21a**

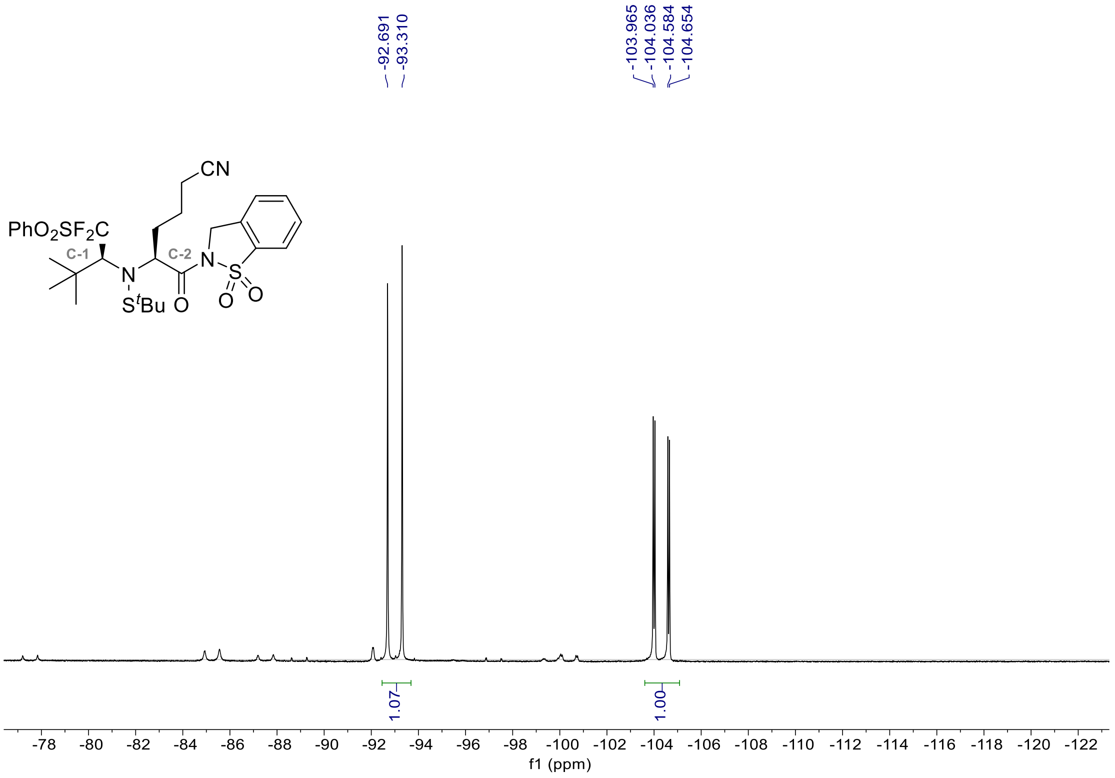

^{1}H NMR (400 MHz, $CDCl_3$) – (*S*$_{S(IV)}$, *R*$_{C\text{-}1}$)-**S21b**

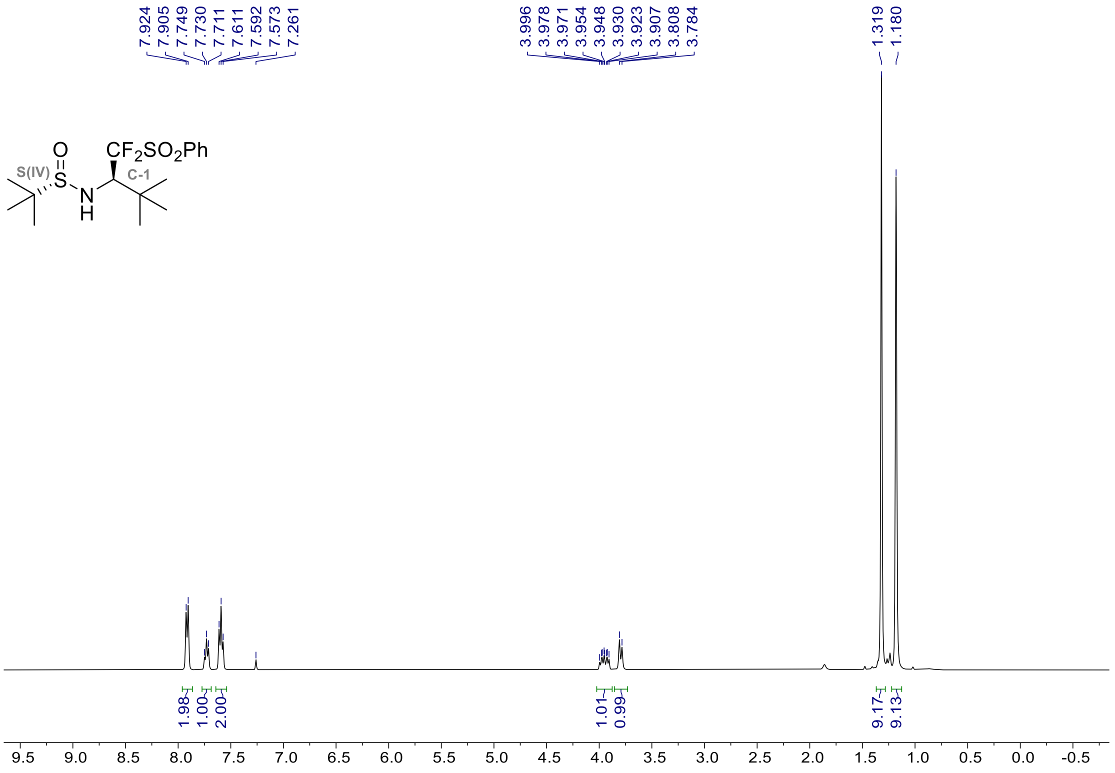

^{13}C NMR (101 MHz, $CDCl_3$) – ($S_{S(IV)}$, $R_{C\text{-}1}$)-**S21b**

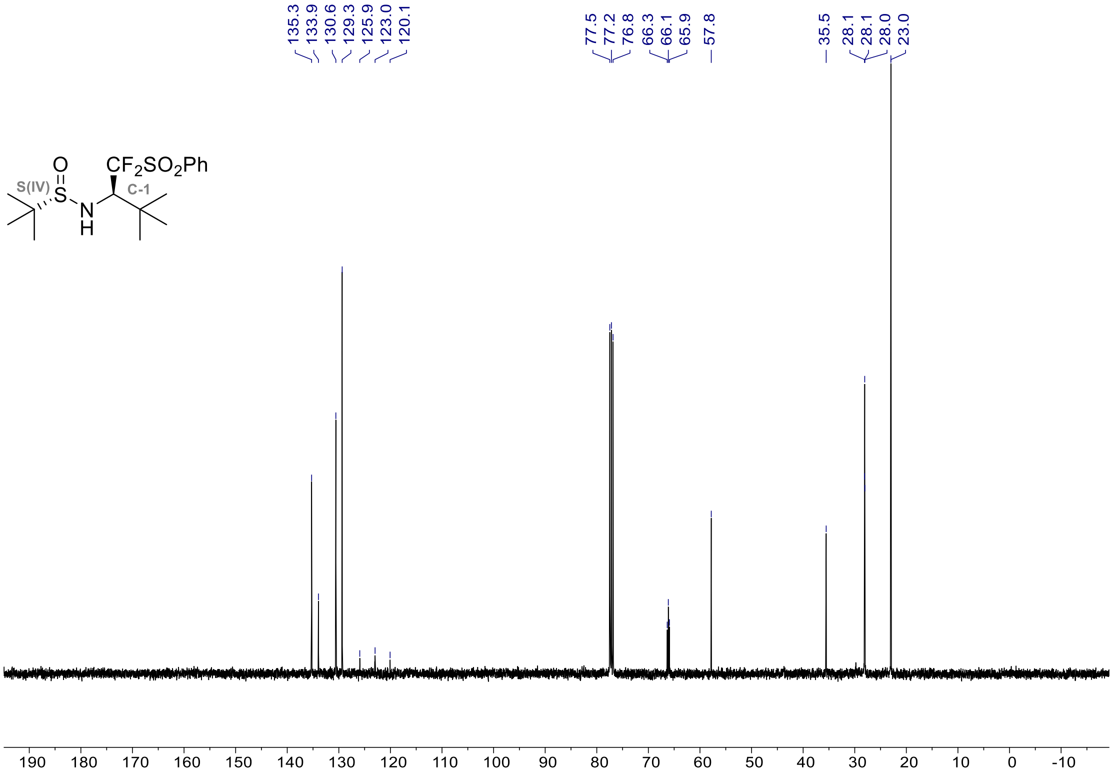

^{19}F NMR (376 MHz, $CDCl_3$) – ($S_{S(IV)}$, $R_{C\text{-}1}$)-**S21b**

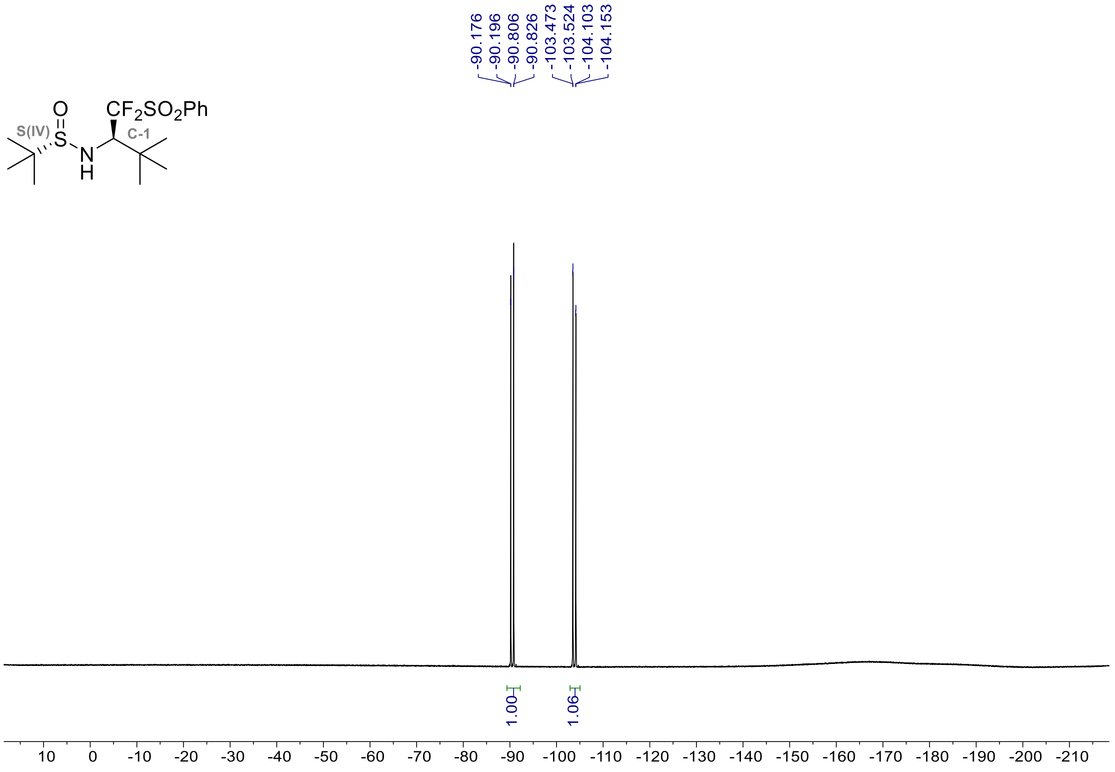

^{1}H NMR (400 MHz, $CDCl_3$) – ($R_{C\text{-}1}$, $R_{C\text{-}2}$)-**21b**

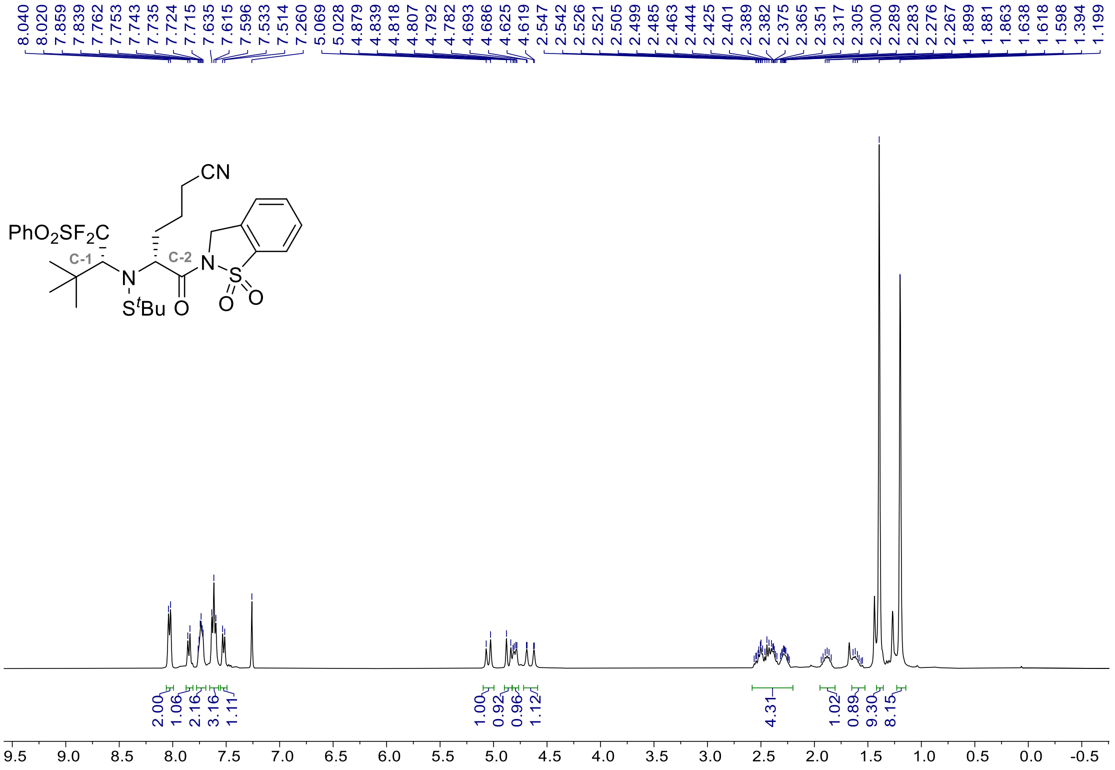

^{13}C NMR (101 MHz, $CDCl_3$) – ($R_{C\text{-}1}$, $R_{C\text{-}2}$)-**21b**

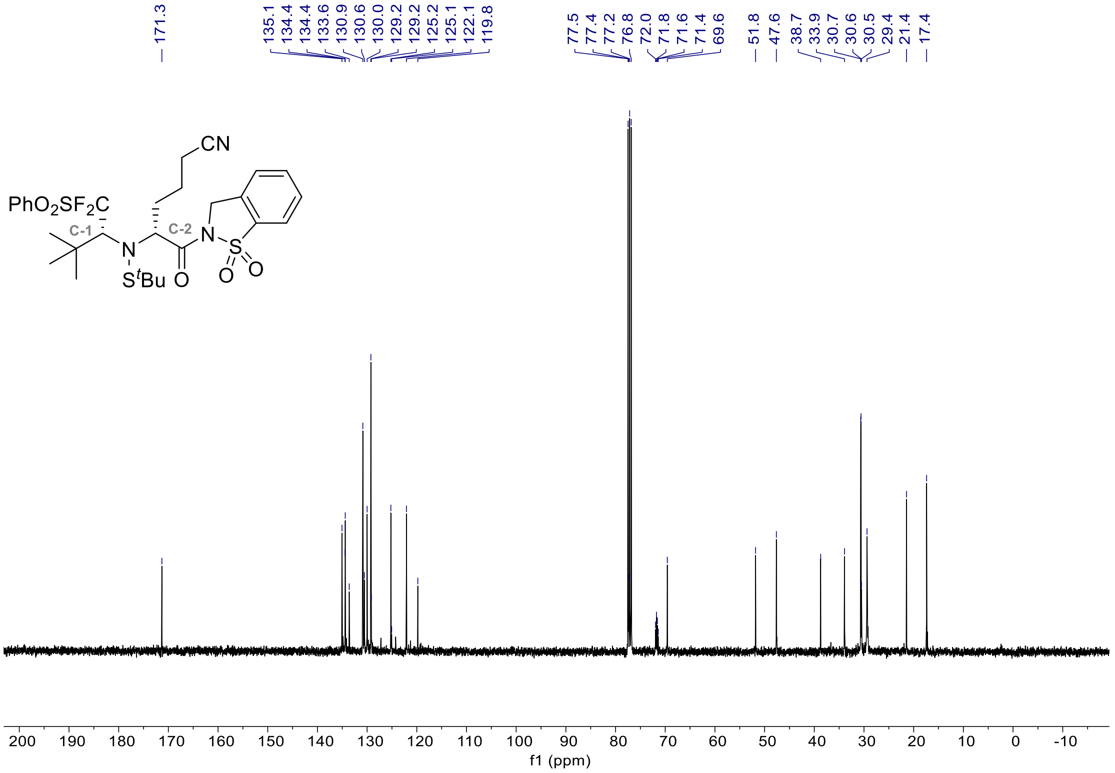

^{19}F NMR (376 MHz, $CDCl_3$) – ($R_{C\text{-}1}$, $R_{C\text{-}2}$)-**21b**

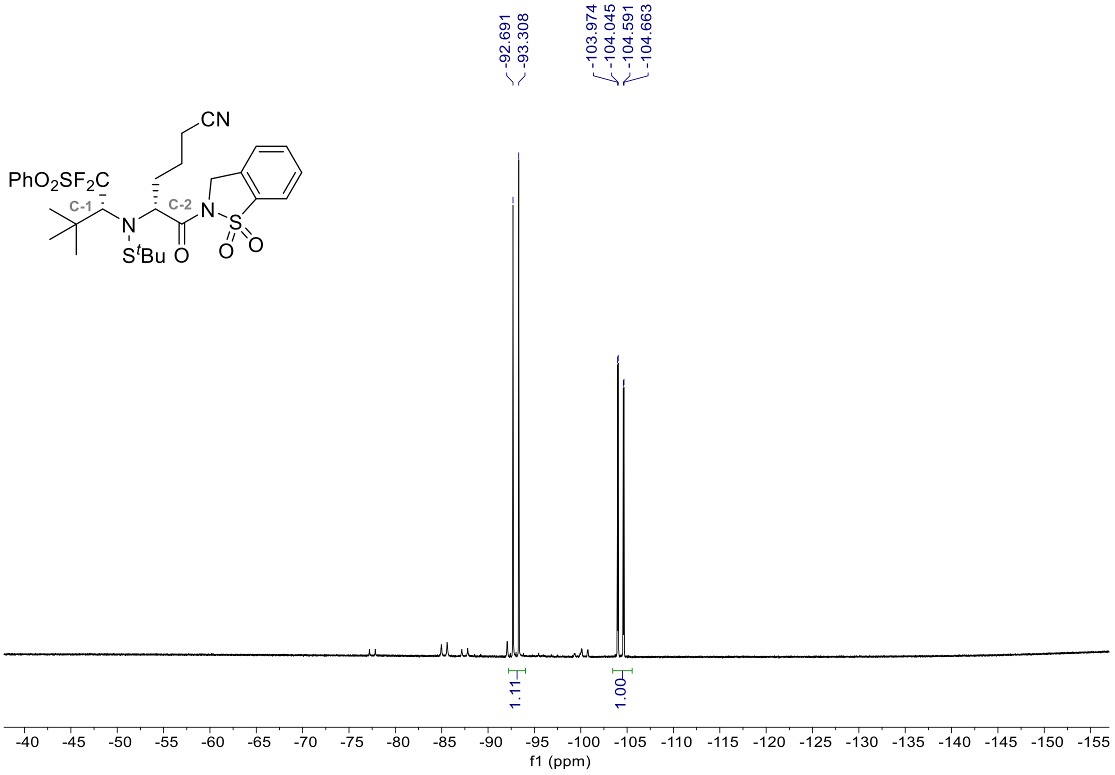

^{1}H NMR (400 MHz, $CDCl_3$) – ($R_{S(IV)}$, $S_{C\text{-}1}$)-**S22a**

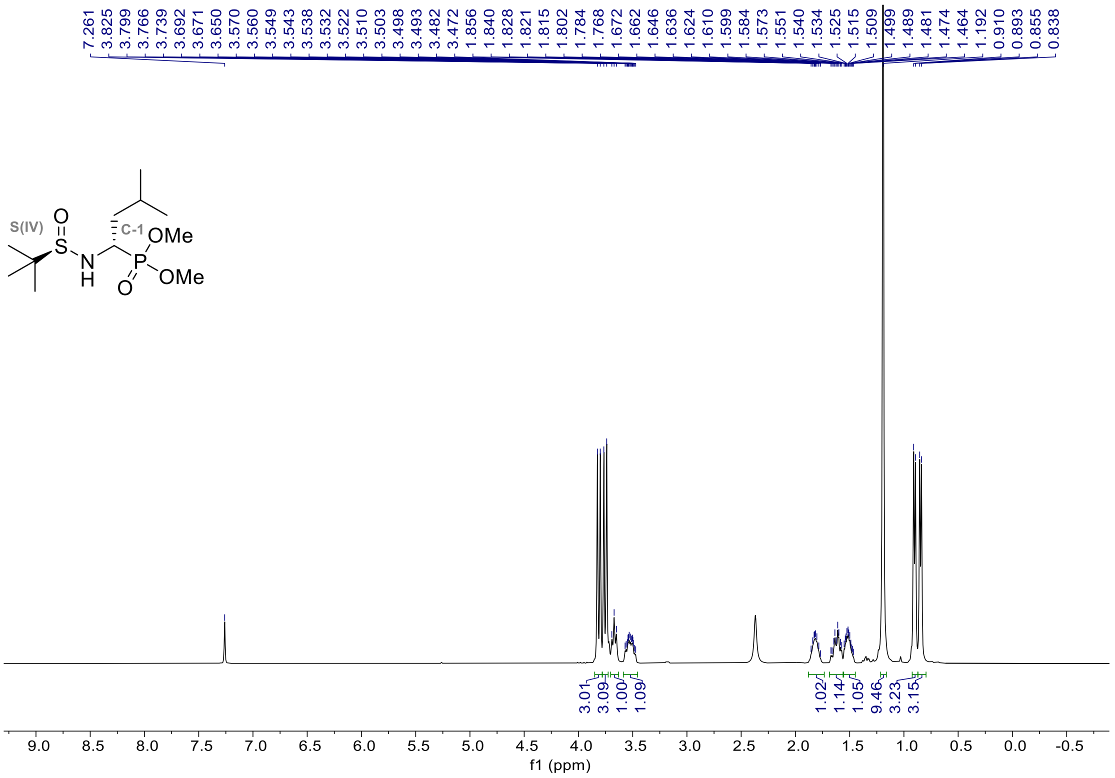

^{13}**C NMR** (101 MHz, CDCl$_3$) – ($R_{S(IV)}$, S_{C-1})-**S22a**

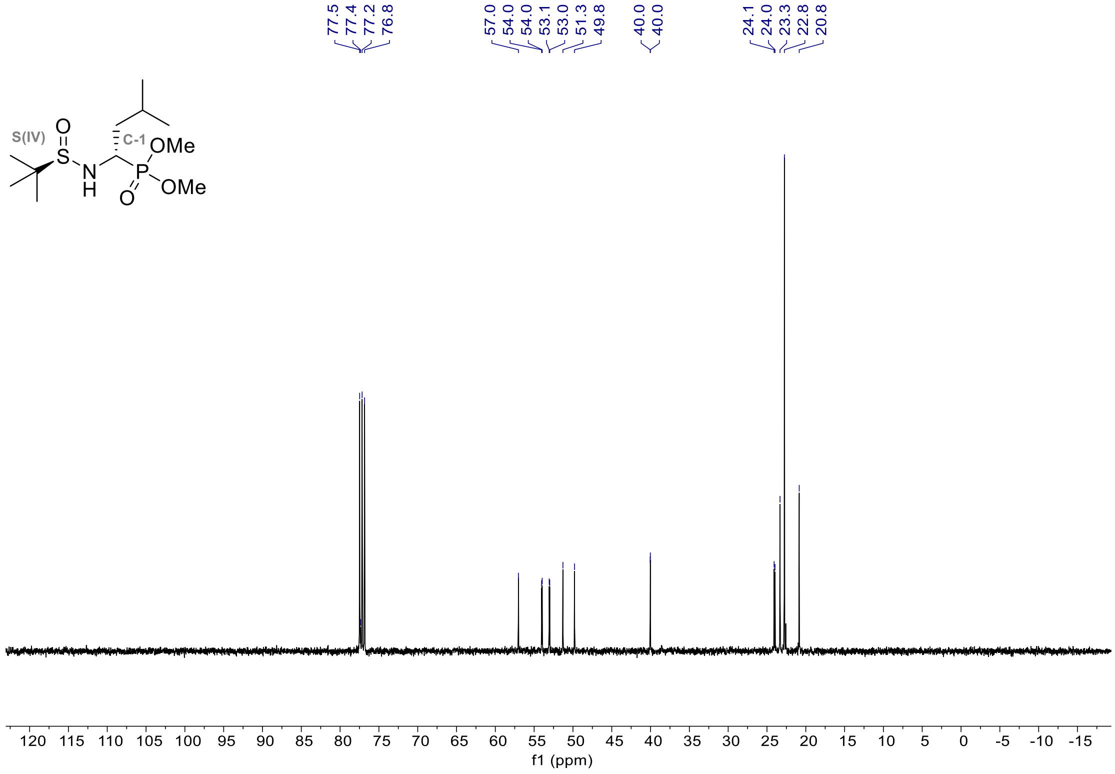

^{31}**P NMR** (162 MHz, CDCl$_3$) – ($R_{S(IV)}$, S_{C-1})-**S22a**

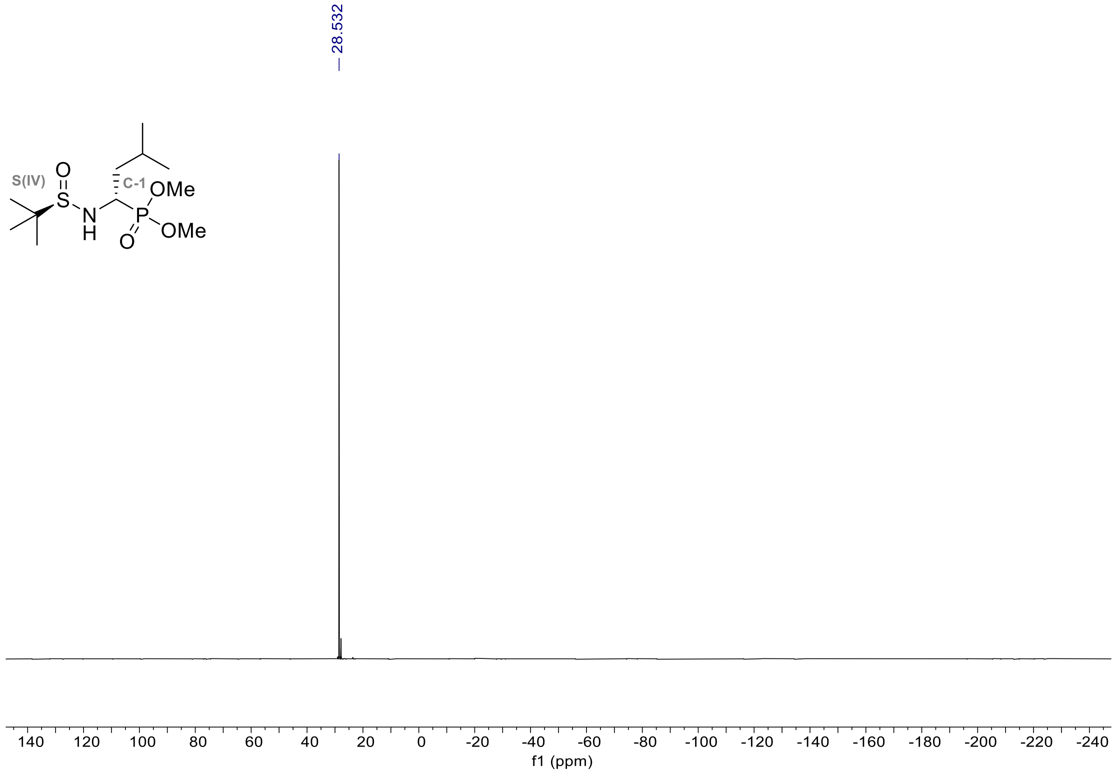

^{1}H NMR (400 MHz, $CDCl_3$) – ($S_{C\text{-}1}$, $S_{C\text{-}2}$)-**22a**

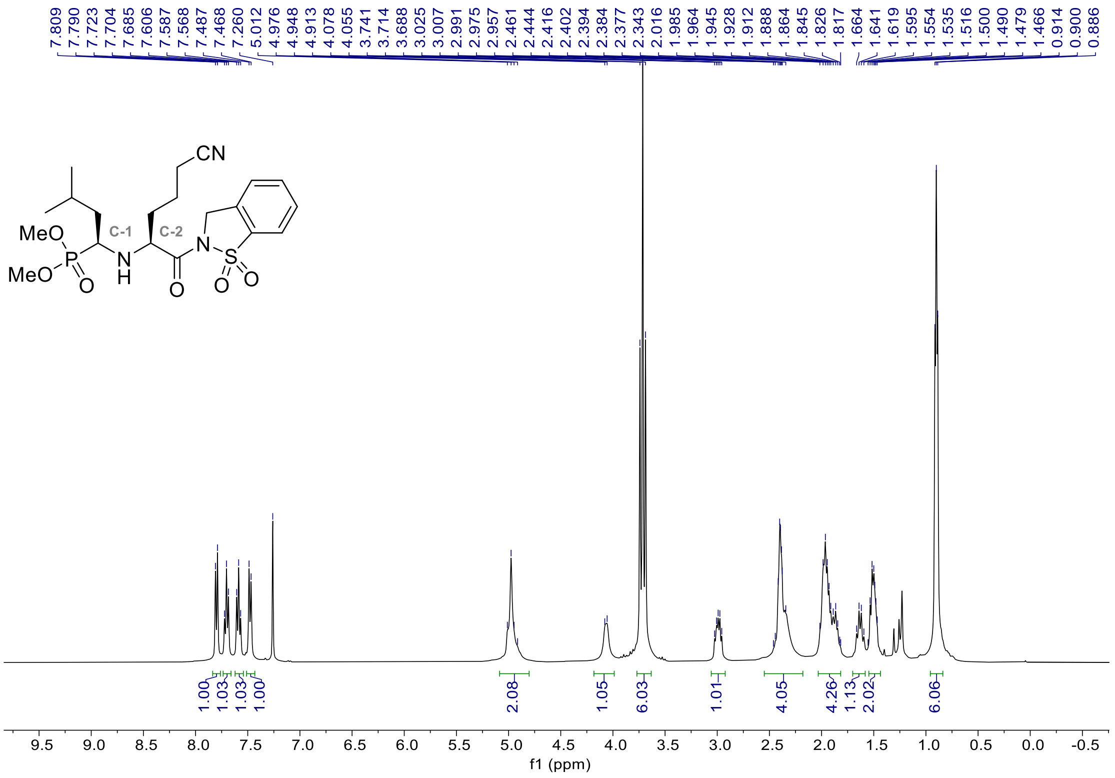

^{13}C NMR (101 MHz, $CDCl_3$) – ($S_{C\text{-}1}$, $S_{C\text{-}2}$)-**22a**

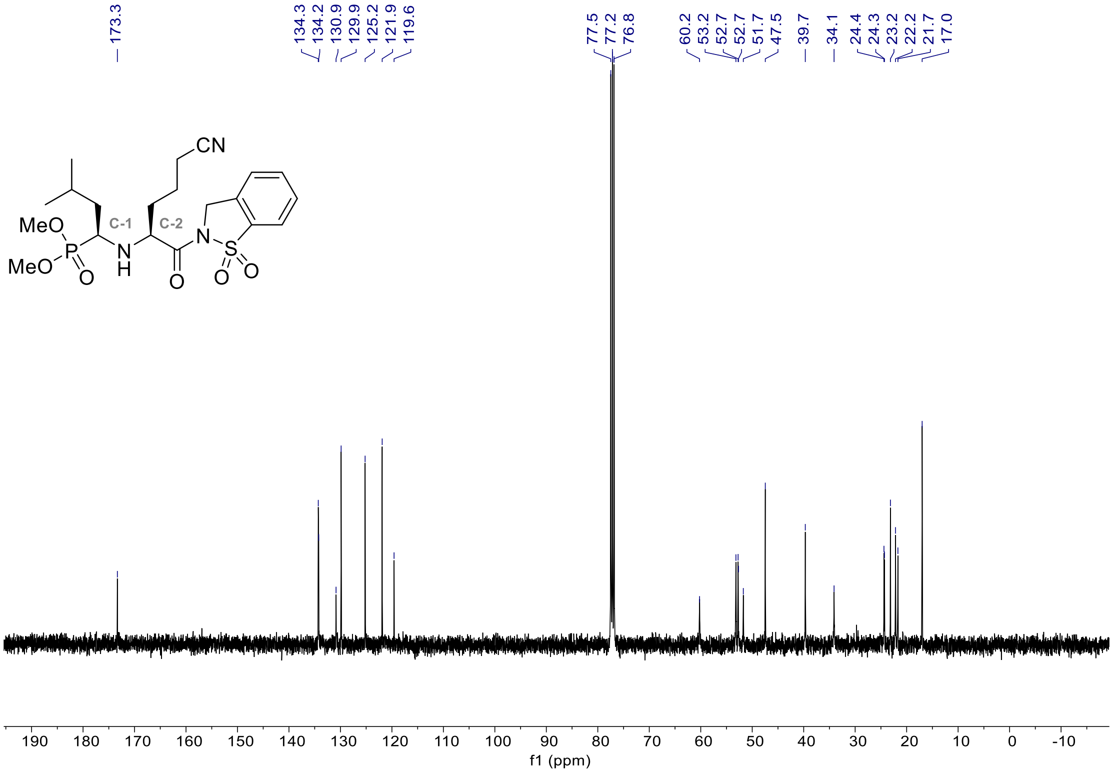

^{31}P NMR (162 MHz, CDCl$_3$) – (S_{C-1}, S_{C-2})-**22a**

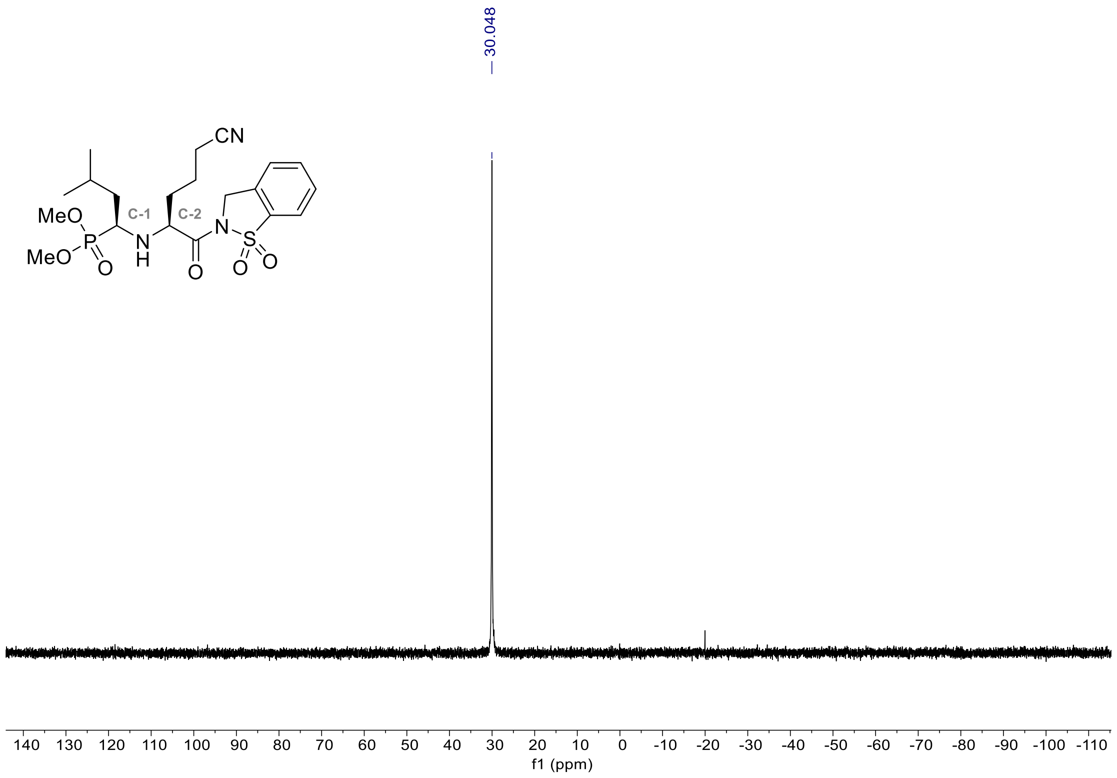

^{1}H NMR (400 MHz, CDCl$_3$) – (R_{C-1}, R_{C-2})-**22b**

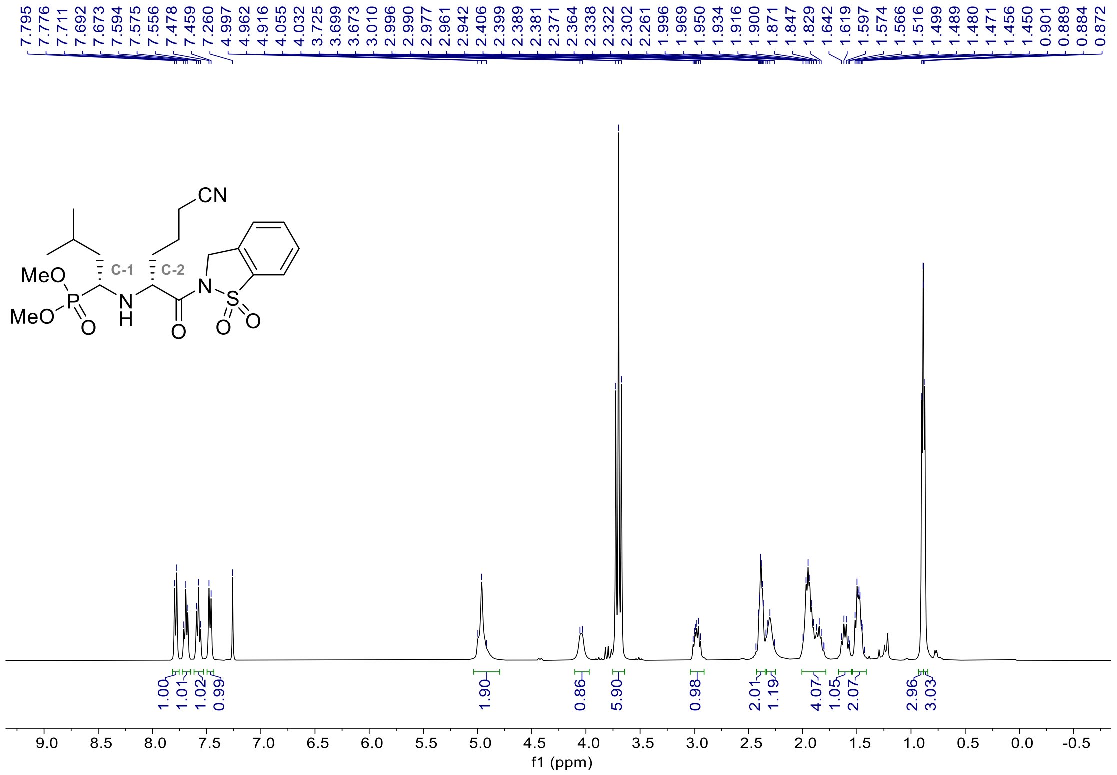

^{13}C NMR (101 MHz, $CDCl_3$) – ($R_{C\text{-}1}$, $R_{C\text{-}2}$)-**22b**

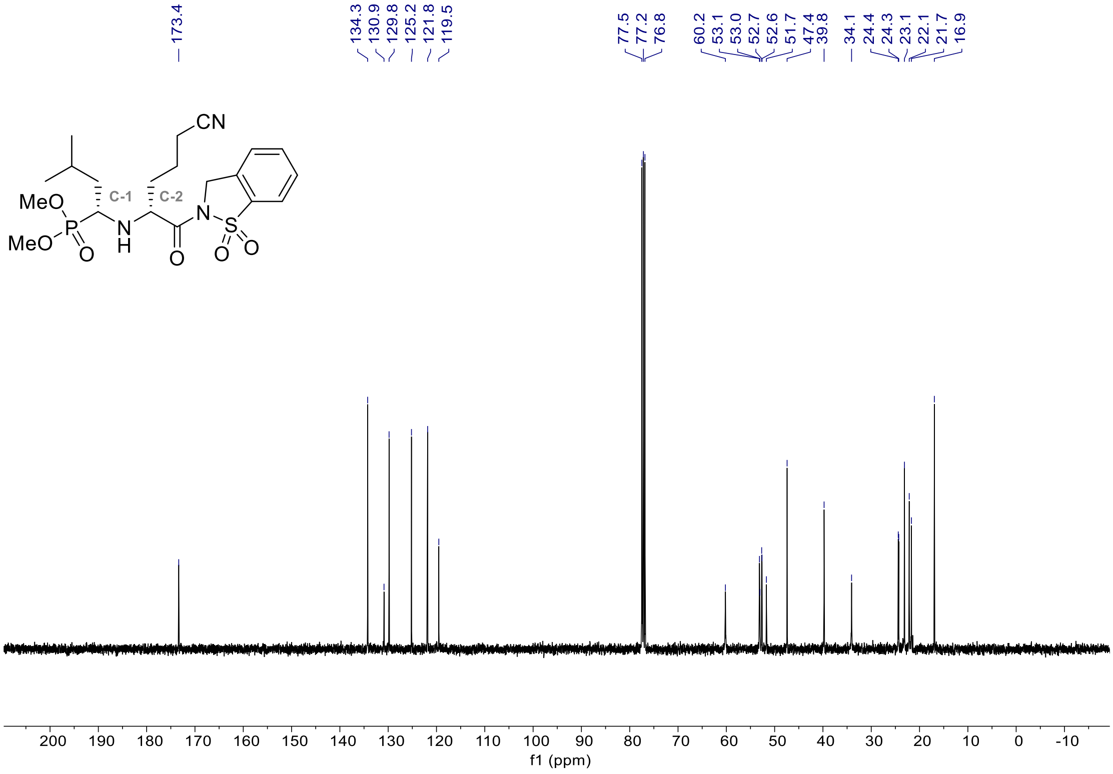

^{31}P NMR (162 MHz, $CDCl_3$) – ($R_{C\text{-}1}$, $R_{C\text{-}2}$)-**22b**

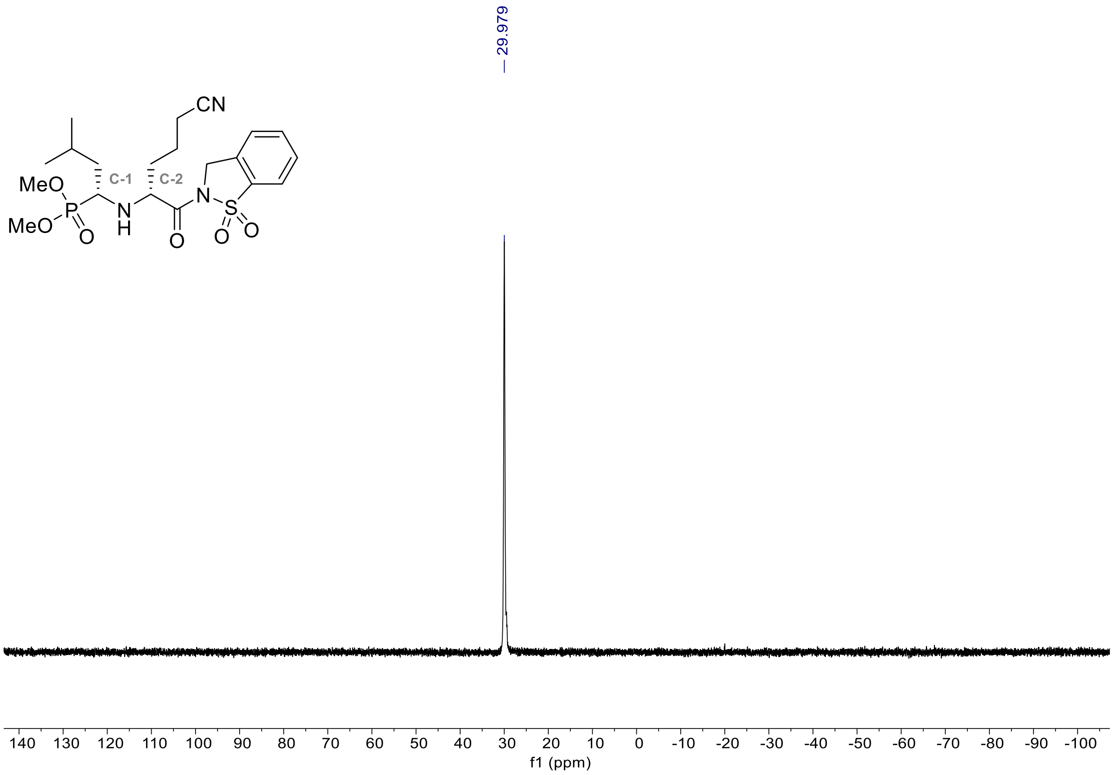

^{1}H NMR (400 MHz, $CDCl_3$) – ($R_{C\text{-}1}$, $S_{C\text{-}2}$, $S_{C\text{-}3}$)-**23**

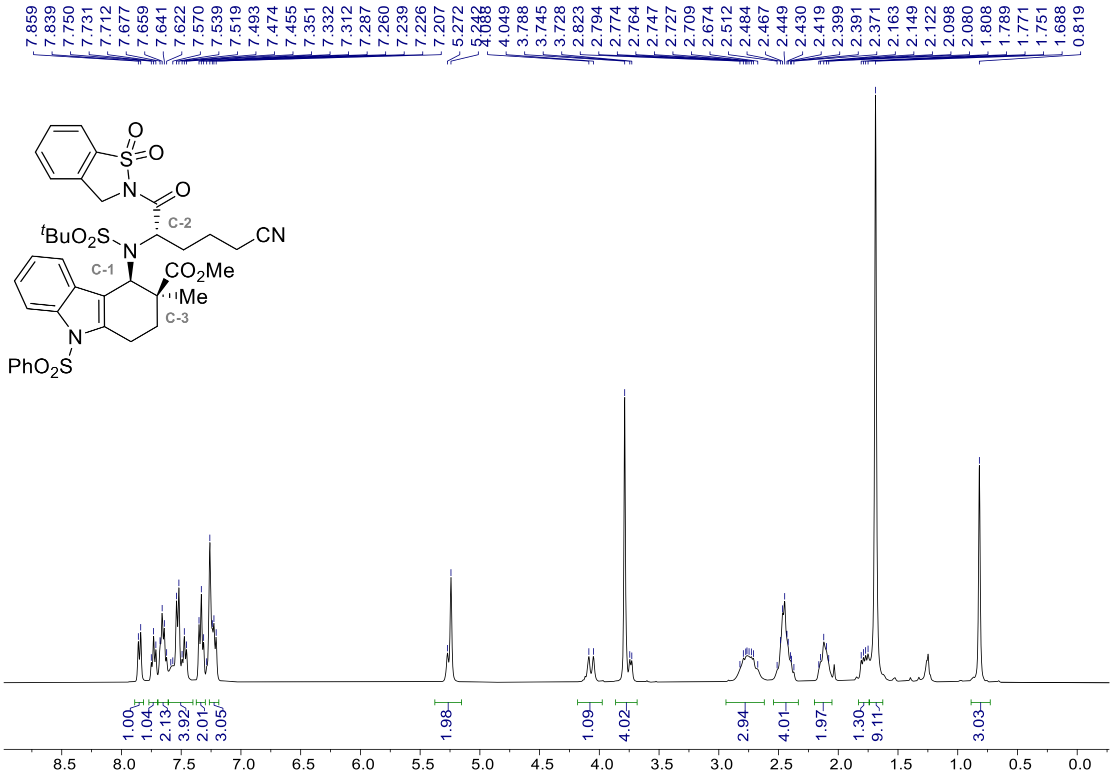

^{13}C NMR (101 MHz, $CDCl_3$) – ($R_{C\text{-}1}$, $S_{C\text{-}2}$, $S_{C\text{-}3}$)-**23**

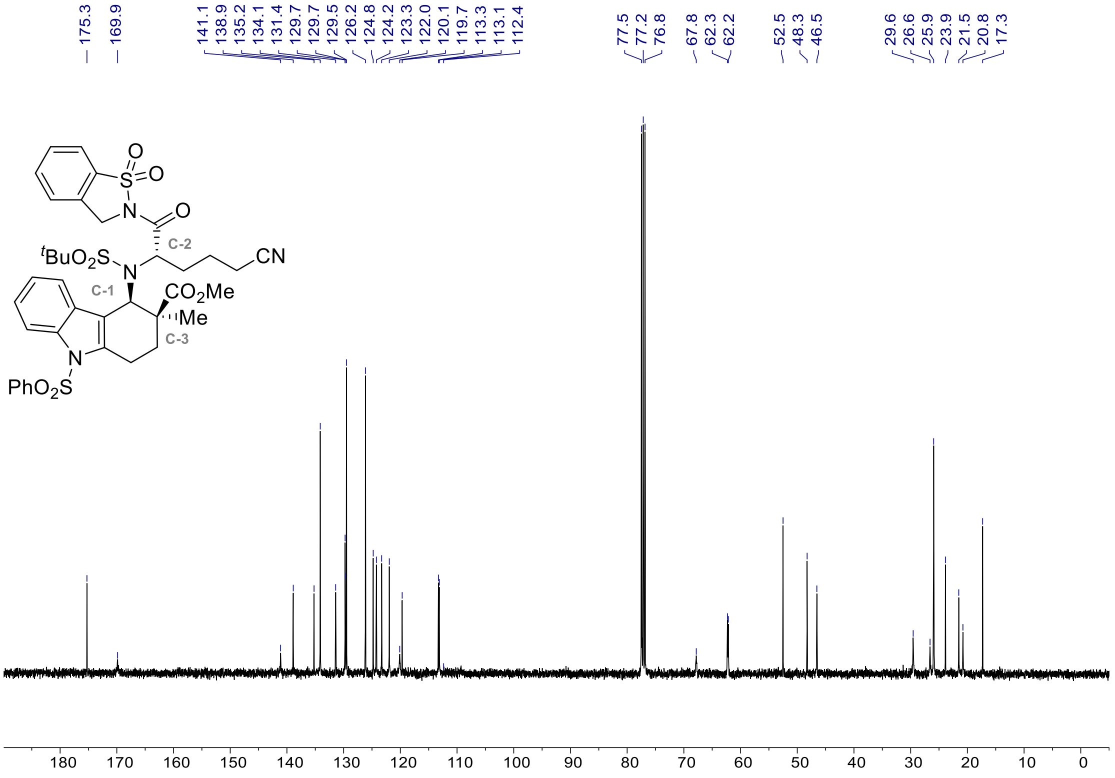

^{1}H NMR (400 MHz, $CDCl_3$) – ($R_{C\text{-}1}$, $S_{C\text{-}2}$)-**24a**

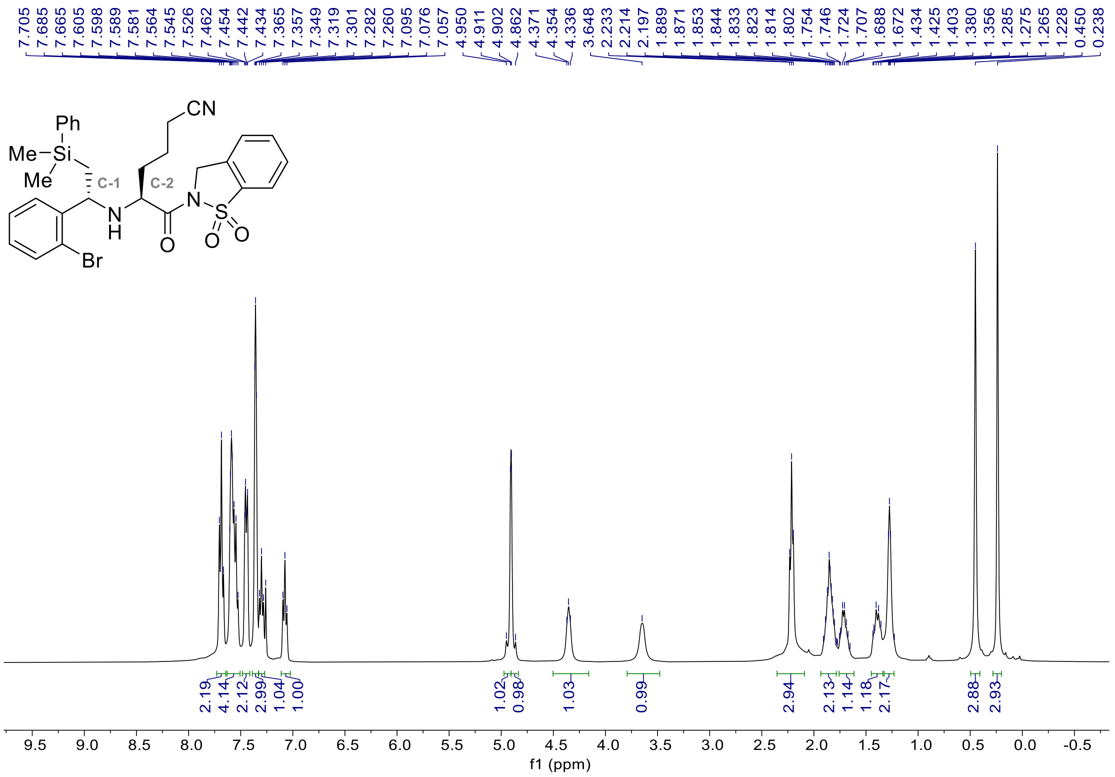

^{13}C NMR (101 MHz, $CDCl_3$) – ($R_{C\text{-}1}$, $S_{C\text{-}2}$)-**24a**

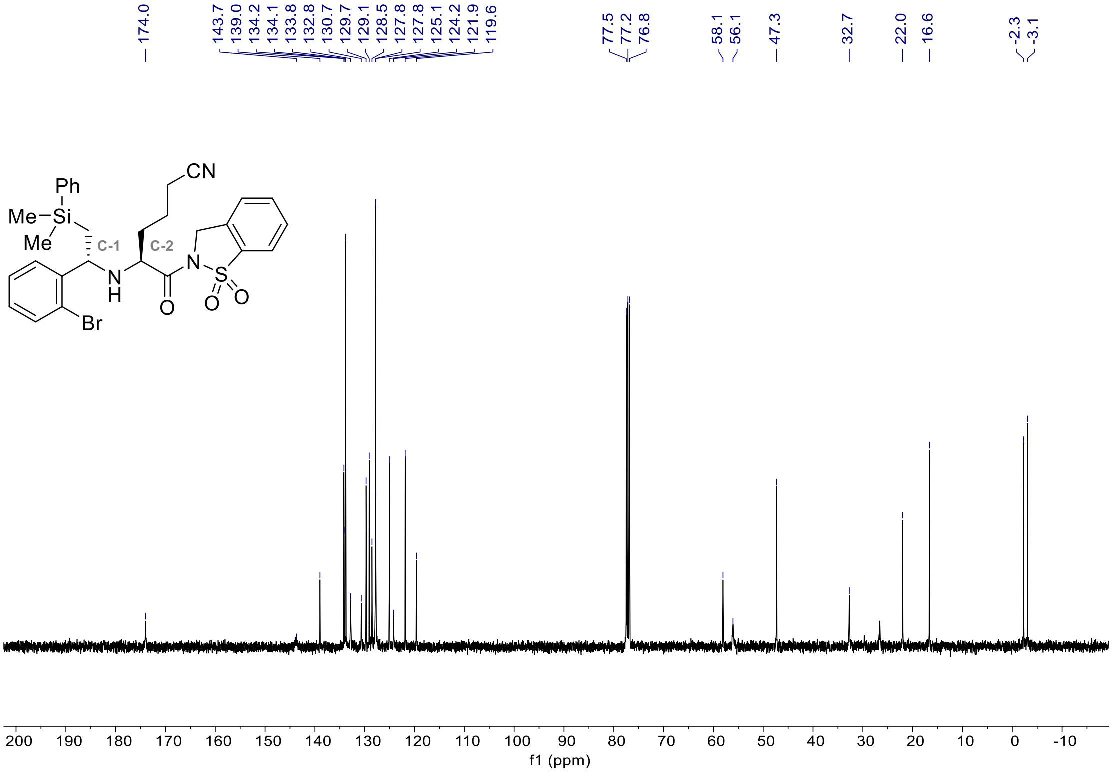

^{1}H NMR (400 MHz, $CDCl_3$) – ($S_{S(IV)}$, $S_{C\text{-}1}$)-**S24b**

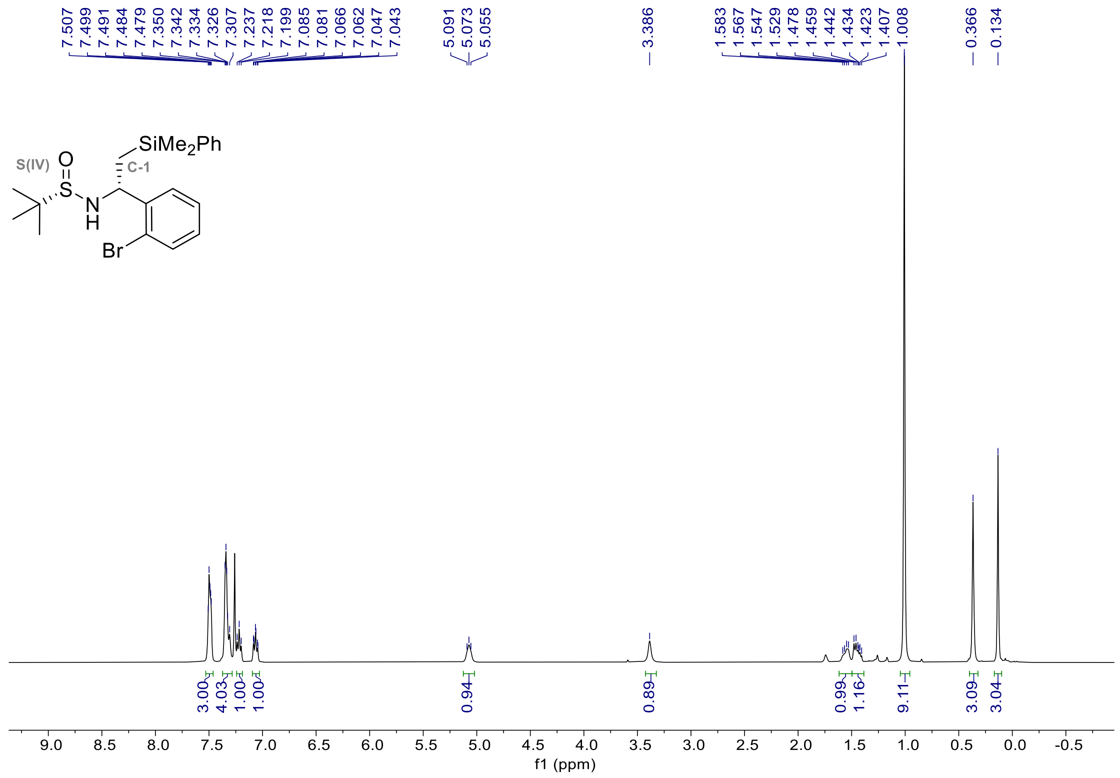

^{13}C NMR (101 MHz, $CDCl_3$) – ($S_{S(IV)}$, $S_{C\text{-}1}$)-**S24b**

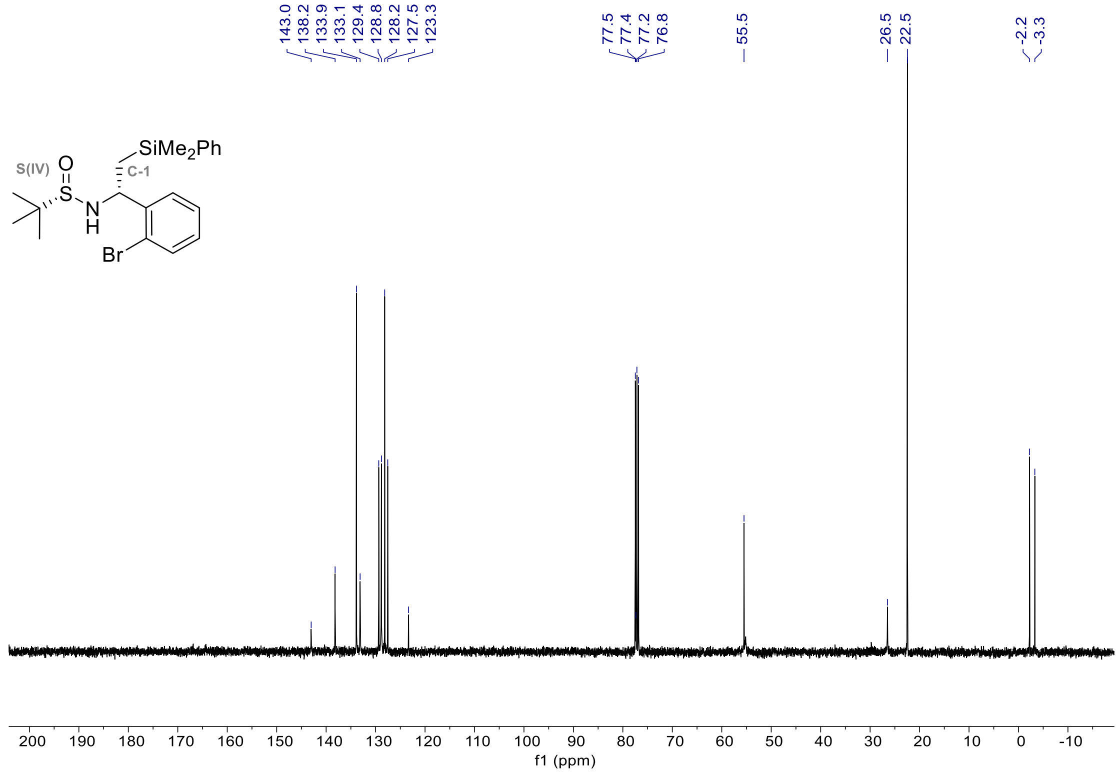

^{1}H NMR (400 MHz, $CDCl_3$) – ($S_{C\text{-}1}$, $R_{C\text{-}2}$)-**24b**

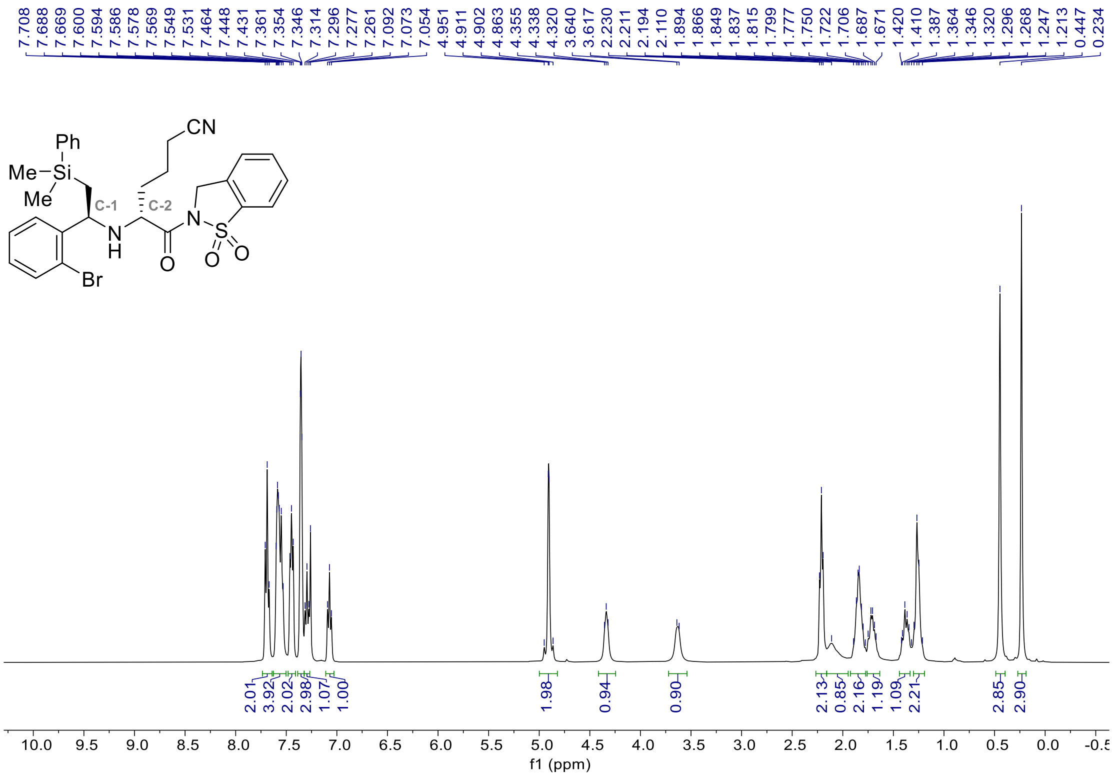

^{13}C NMR (101 MHz, $CDCl_3$) – ($S_{C\text{-}1}$, $R_{C\text{-}2}$)-**24b**

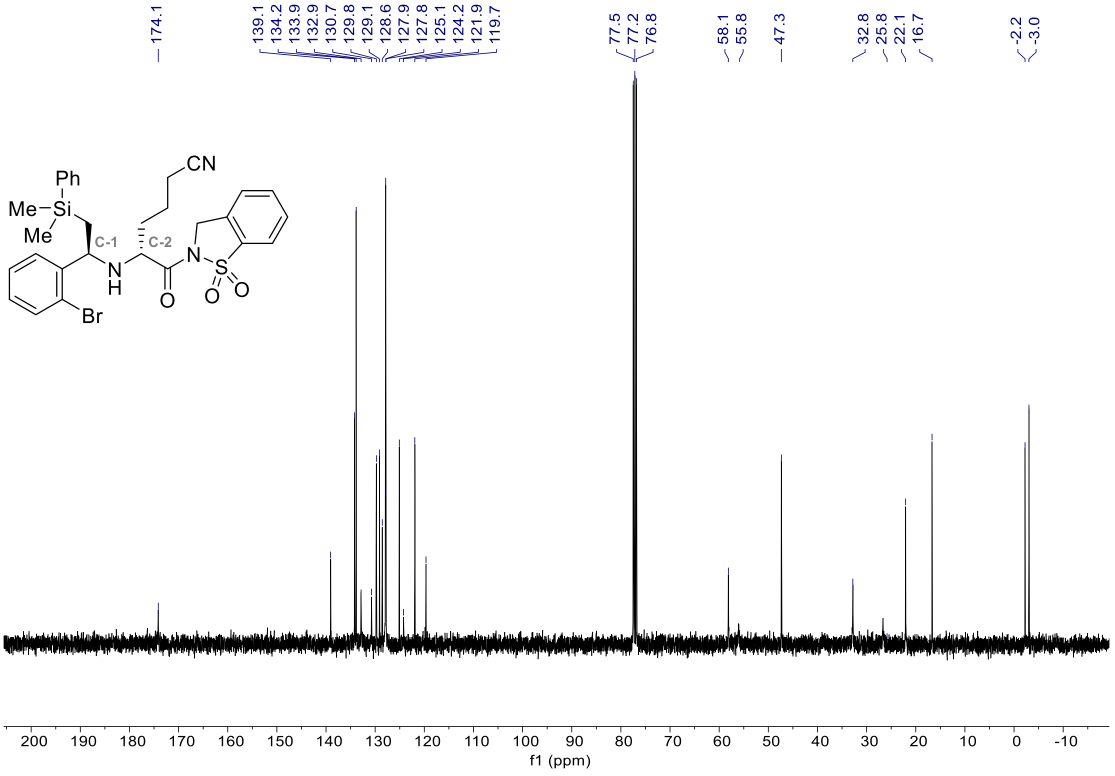

^{1}**H NMR** (400 MHz, $CDCl_3$) – (*R*$_{S(IV)}$, *S*$_{C-1}$)-**S25a**

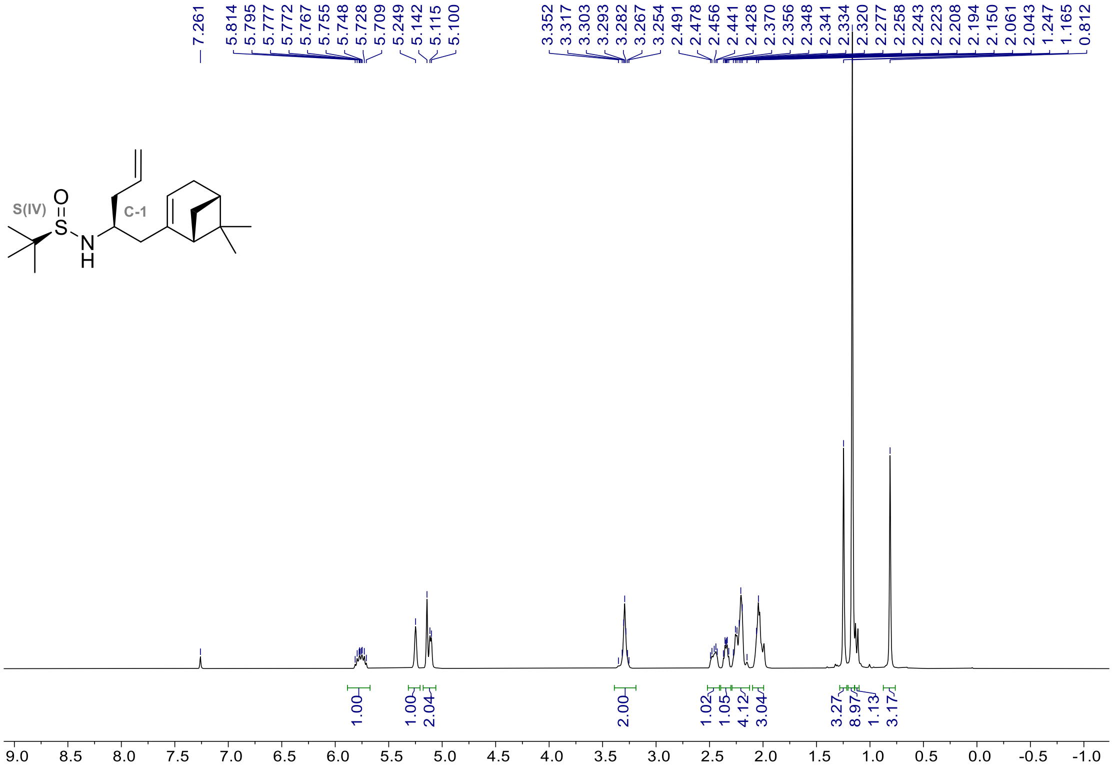

^{13}**C NMR** (101 MHz, $CDCl_3$) – (*R*$_{S(IV)}$, *S*$_{C-1}$)-**S25a**

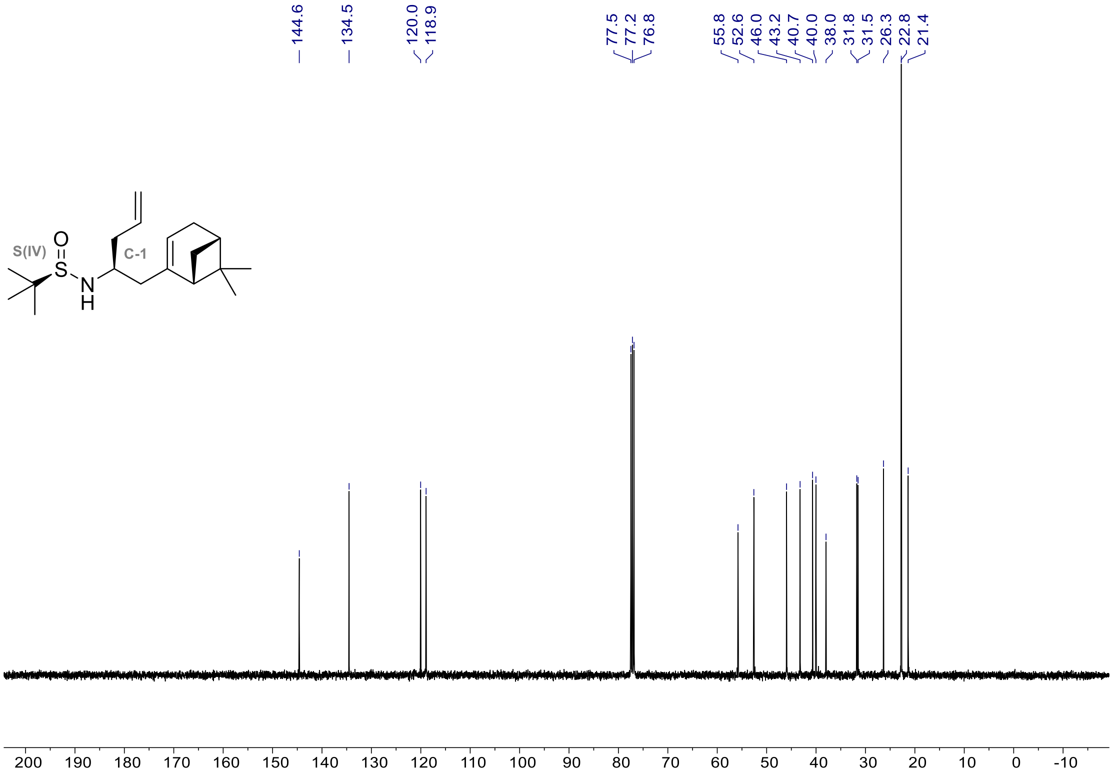

^{1}H NMR (400 MHz, $CDCl_3$) – ($S_{C\text{-}1}$, $S_{C\text{-}2}$)-**25a**

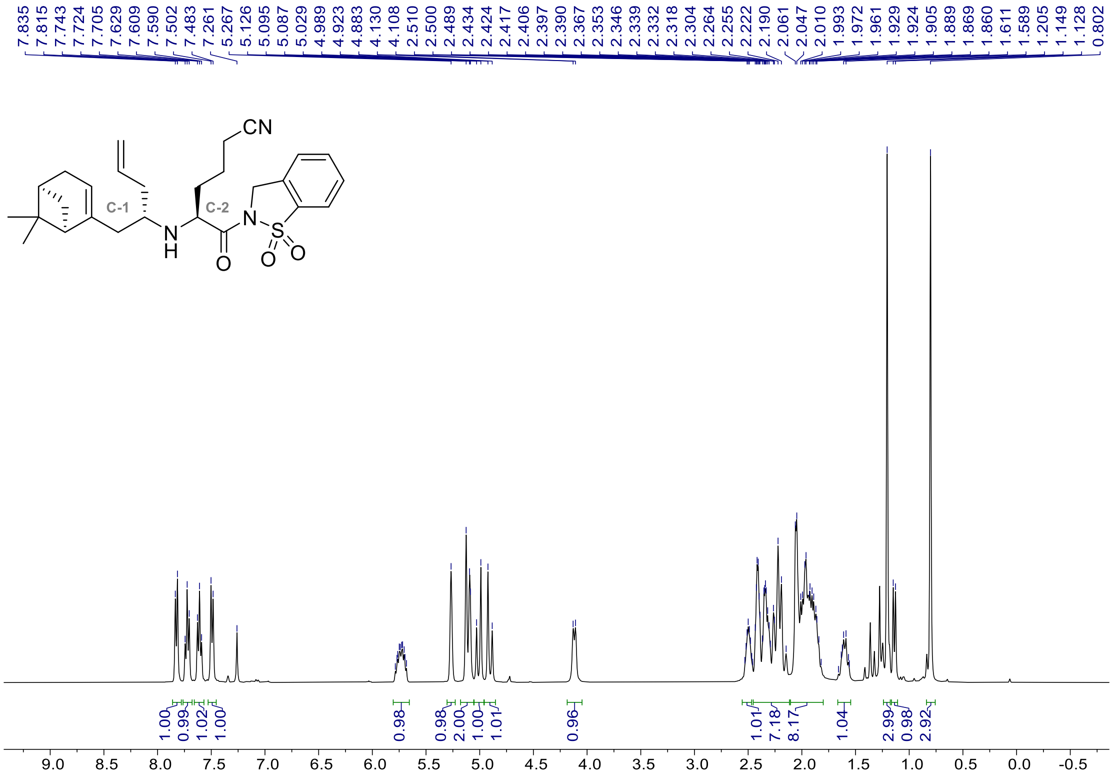

^{13}C NMR (101 MHz, $CDCl_3$) – ($S_{C\text{-}1}$, $S_{C\text{-}2}$)-**25a**

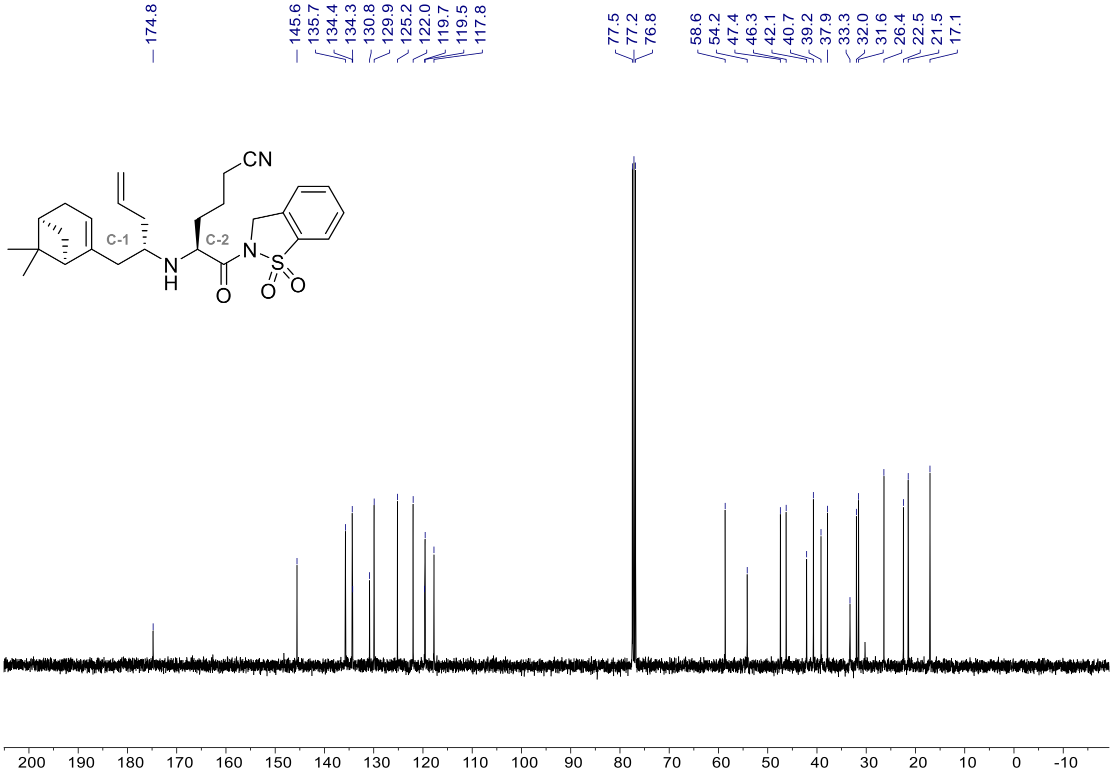

^{1}H NMR (400 MHz, CDCl$_3$) – ($R_{C\text{-}1}$, $R_{C\text{-}2}$)-**25b**

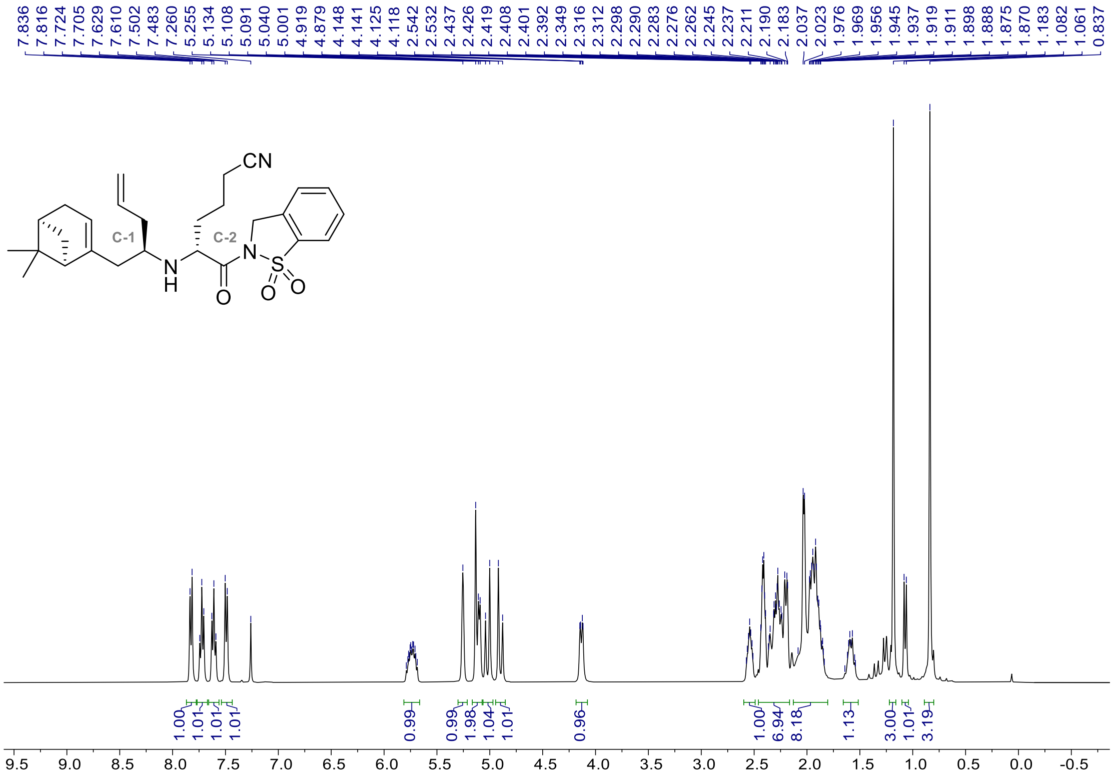

^{13}C NMR (101 MHz, CDCl$_3$) – ($R_{C\text{-}1}$, $R_{C\text{-}2}$)-**25b**

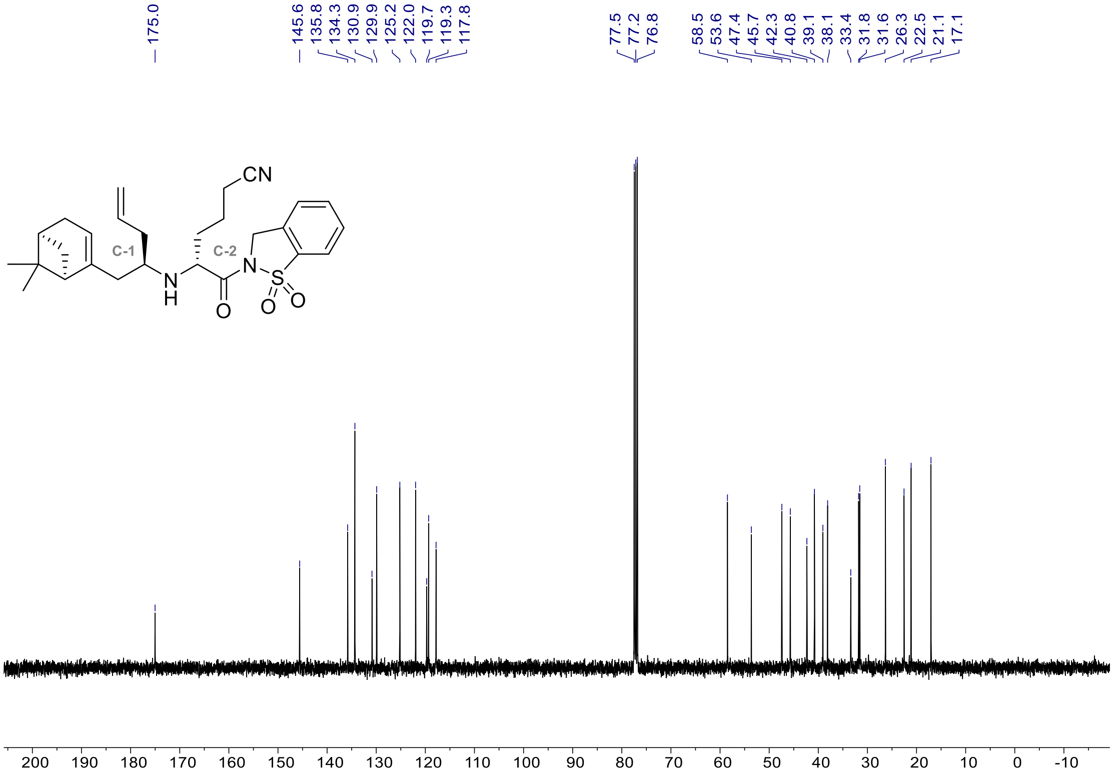

^{1}H NMR (400 MHz, $CDCl_3$) – (R_{C-1}, S_{C-2})-**26a**

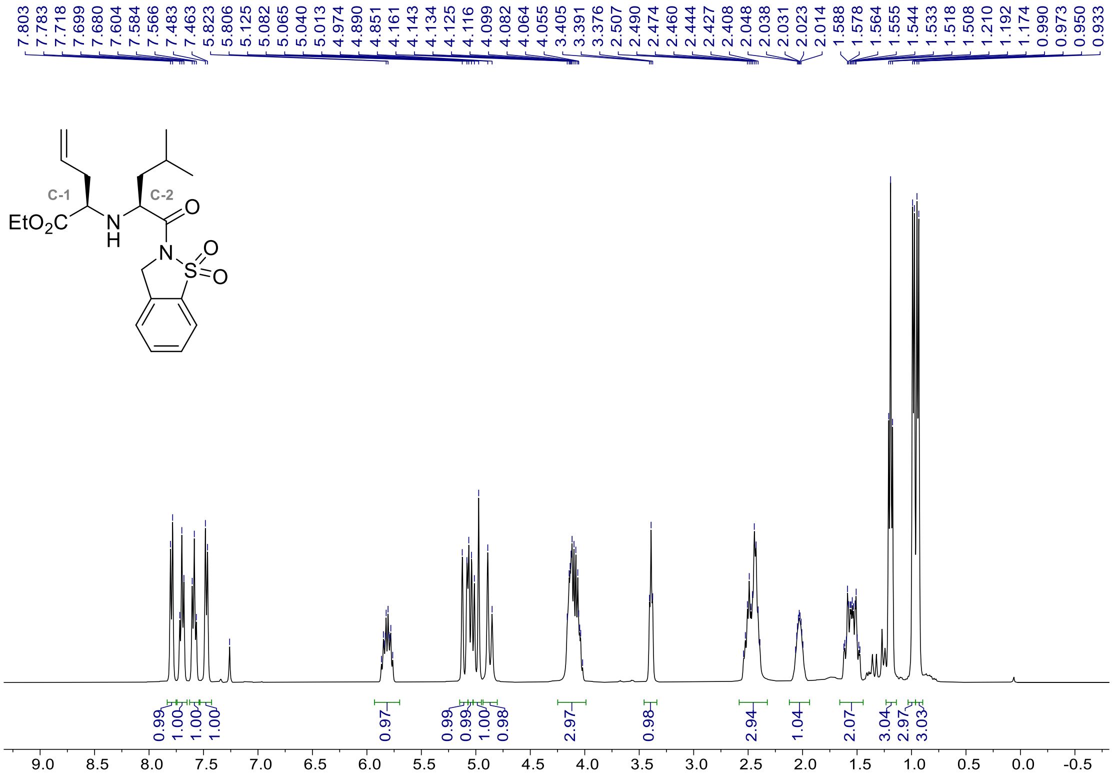

^{13}C NMR (101 MHz, $CDCl_3$) – (R_{C-1}, S_{C-2})-**26a**

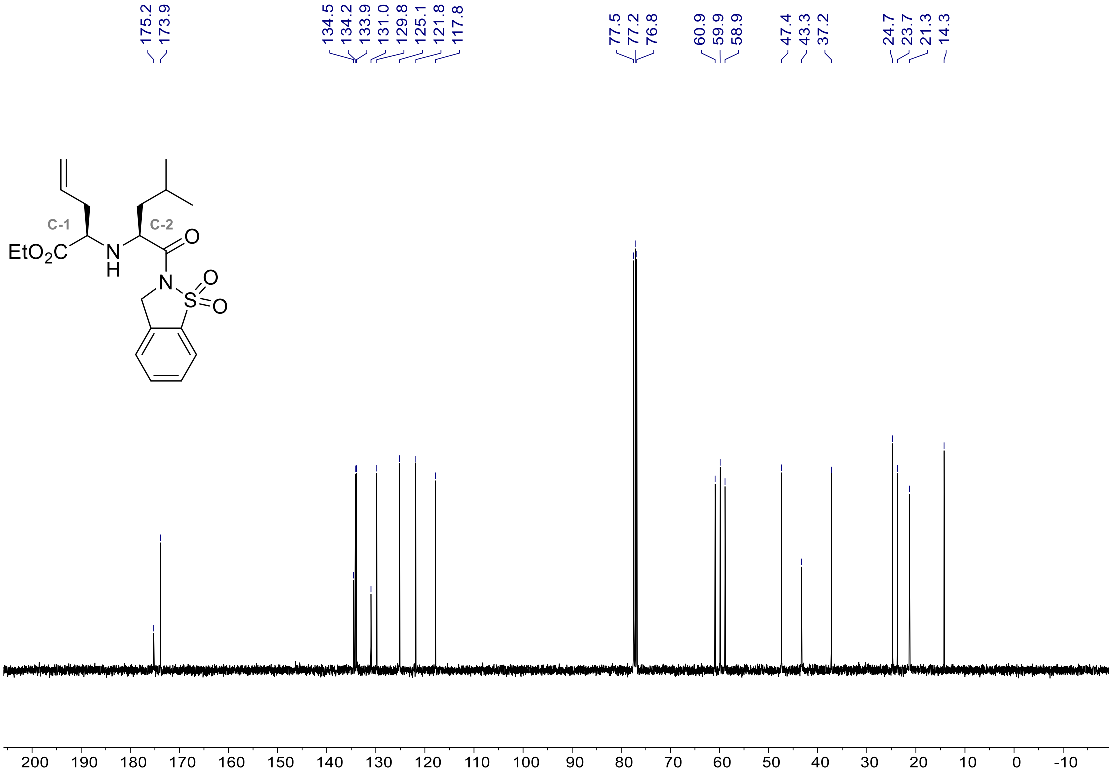

^{1}H NMR (400 MHz, $CDCl_3$) – ($R_{S(IV)}$, $R_{C\text{-}1}$)-**S27a**

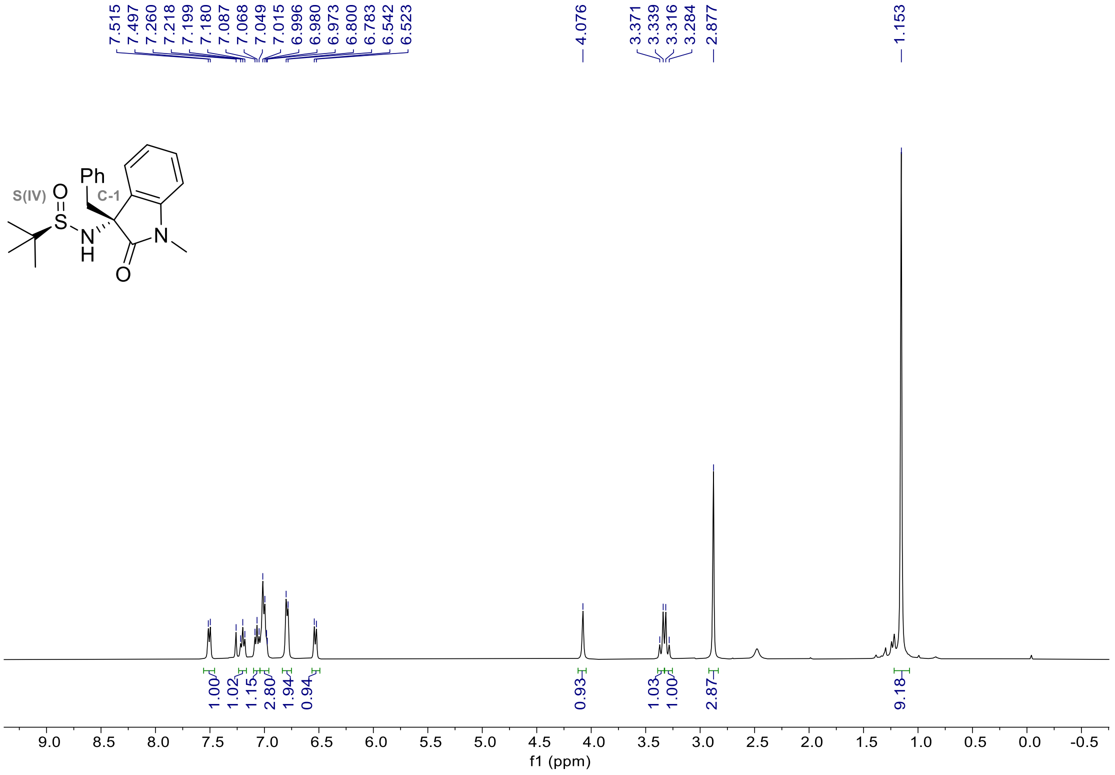

^{13}C NMR (101 MHz, $CDCl_3$) – ($R_{S(IV)}$, $R_{C\text{-}1}$)-**S27a**

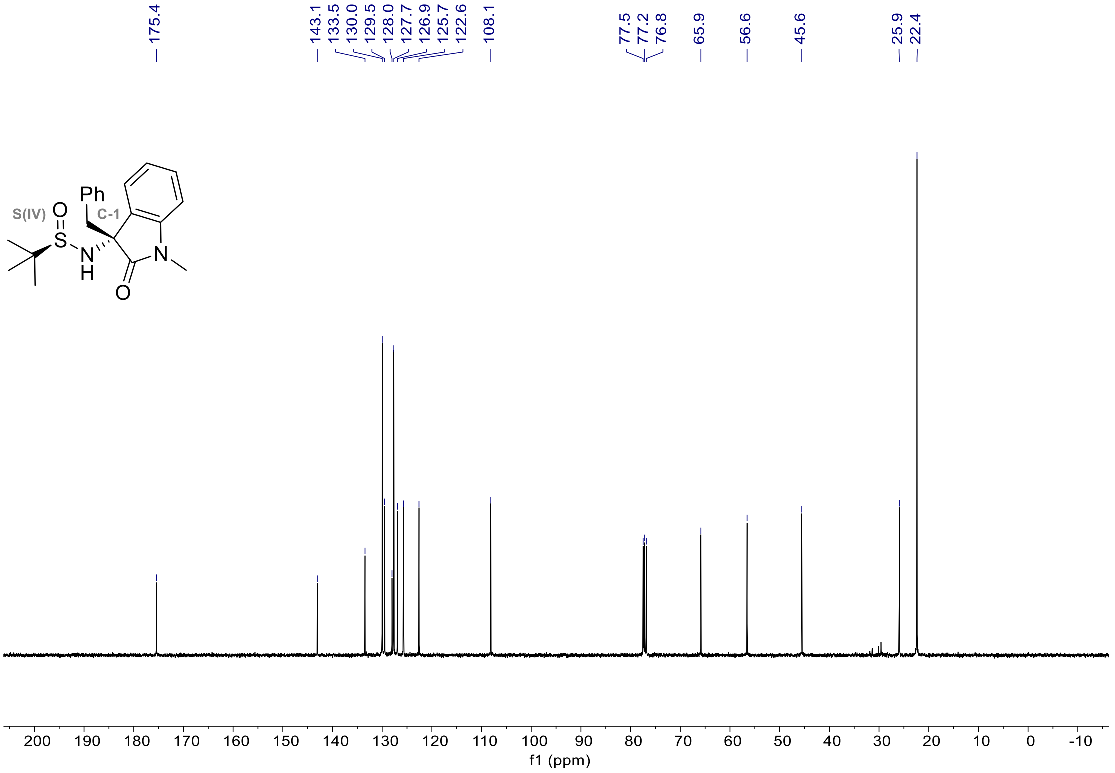

^{1}H NMR (400 MHz, $CDCl_3$) – ($R_{C\text{-}1}$, $S_{C\text{-}2}$)-**27a**

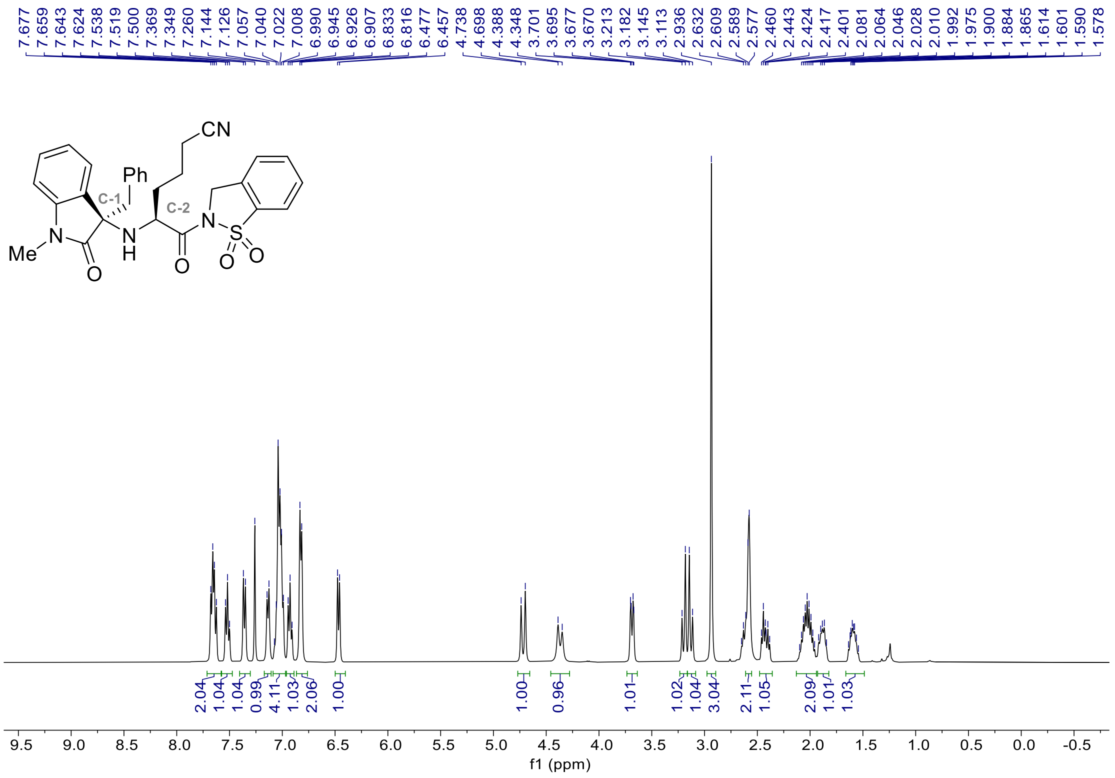

^{13}C NMR (101 MHz, $CDCl_3$) – ($R_{C\text{-}1}$, $S_{C\text{-}2}$)-**27a**

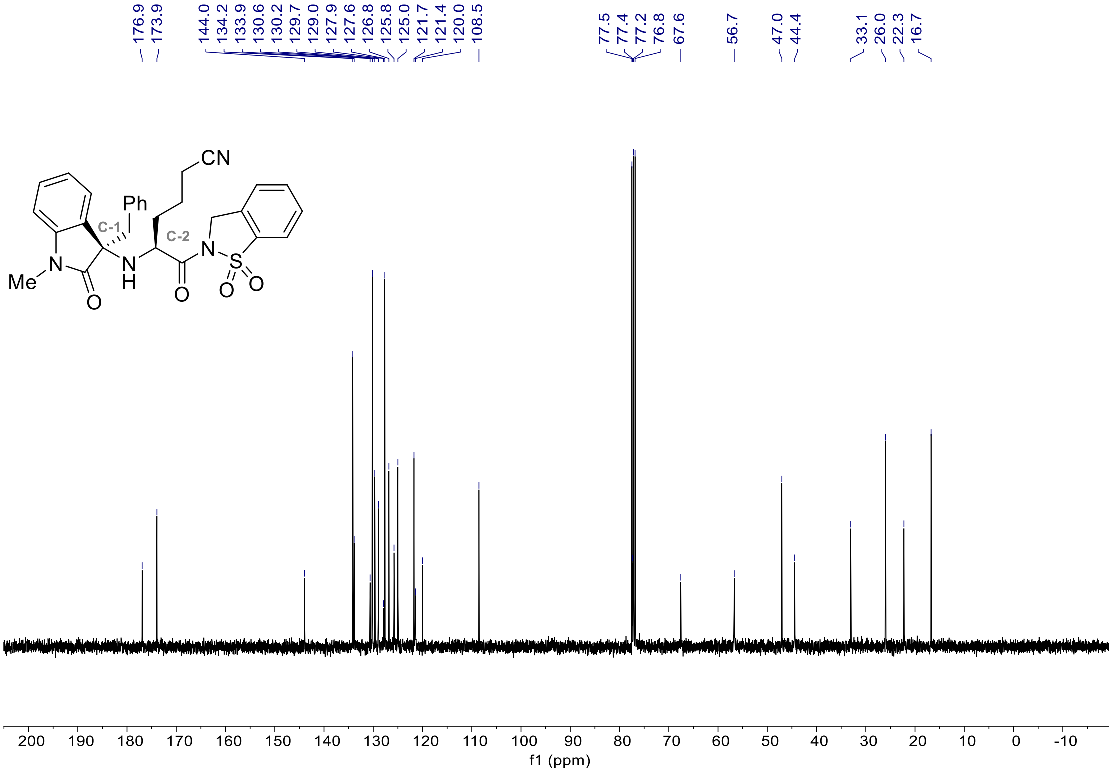

^{1}H NMR (400 MHz, $CDCl_3$) – ($S_{C\text{-}1}$, $R_{C\text{-}2}$)-**27b**

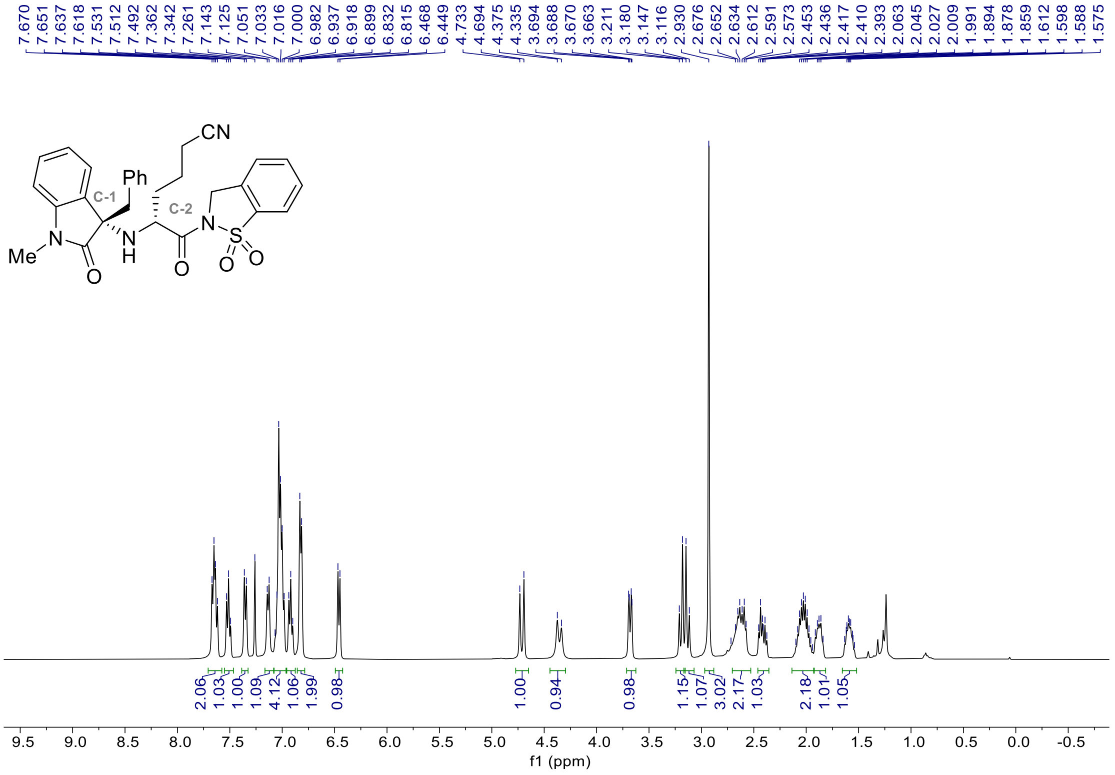

^{13}C NMR (101 MHz, $CDCl_3$) – ($S_{C\text{-}1}$, $R_{C\text{-}2}$)-**27b**

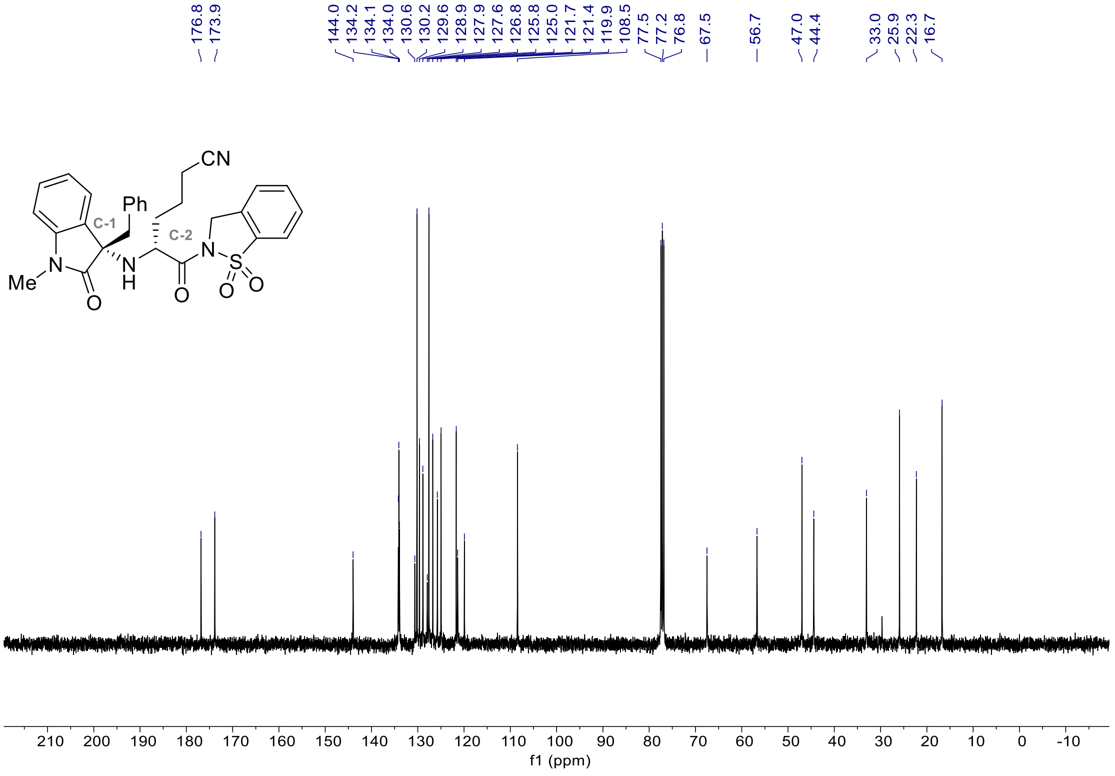

^{1}H NMR (400 MHz, $CDCl_3$) – ($R_{S(IV)}$, S_{C-1})-**S28a**

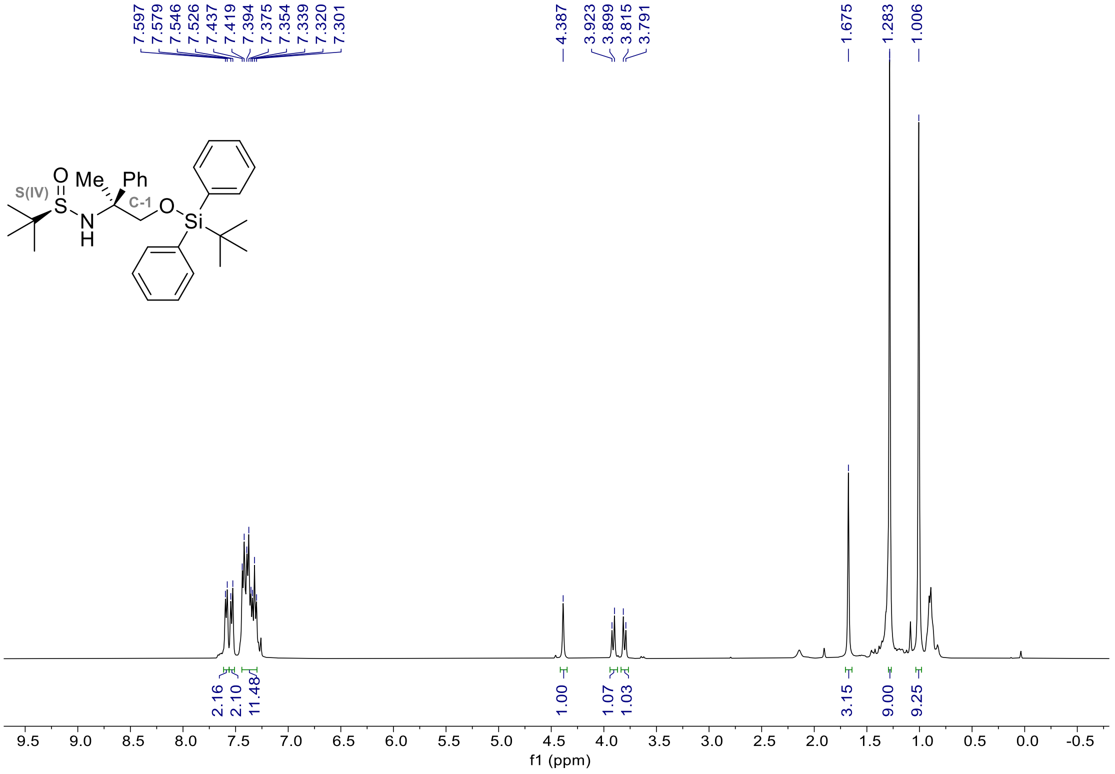

^{13}C NMR (101 MHz, $CDCl_3$) – ($R_{S(IV)}$, S_{C-1})-**S28a**

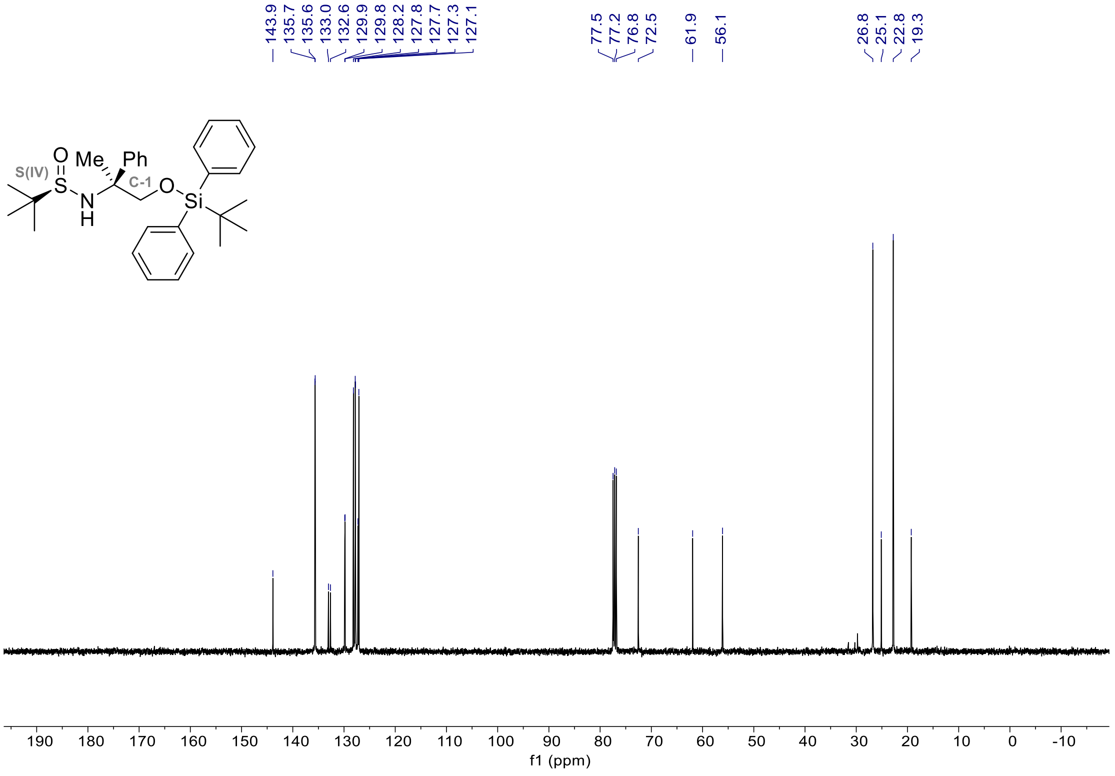

^{1}H NMR (400 MHz, $CDCl_3$) – ($S_{C\text{-}1}$, $S_{C\text{-}2}$)-**28a**

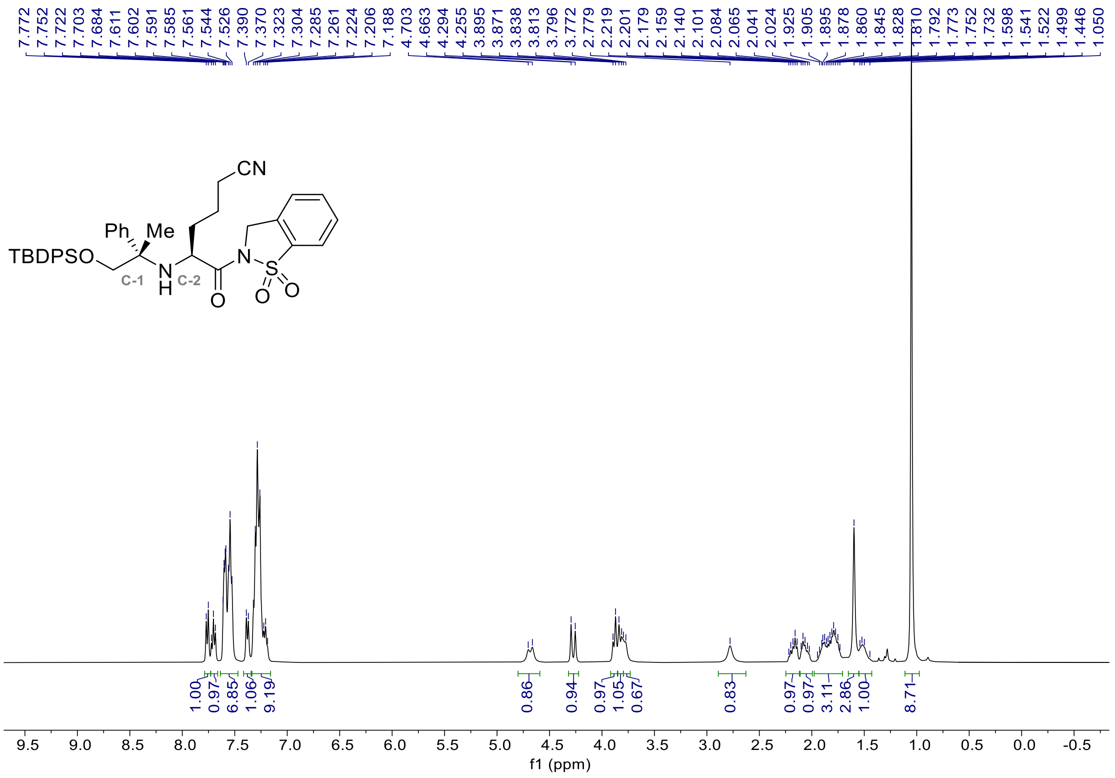

^{13}C NMR (101 MHz, $CDCl_3$) – ($S_{C\text{-}1}$, $S_{C\text{-}2}$)-**28a**

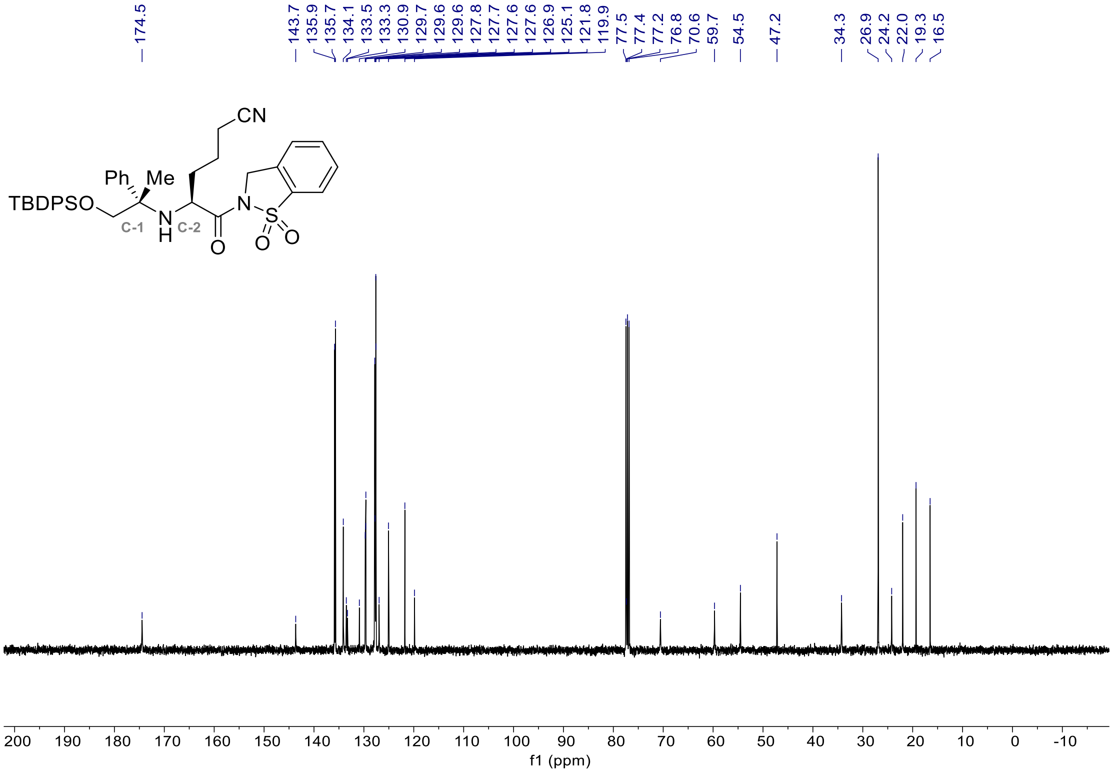

^{1}H NMR (400 MHz, $CDCl_3$) – ($R_{C\text{-}1}$, $R_{C\text{-}2}$)-**28b**

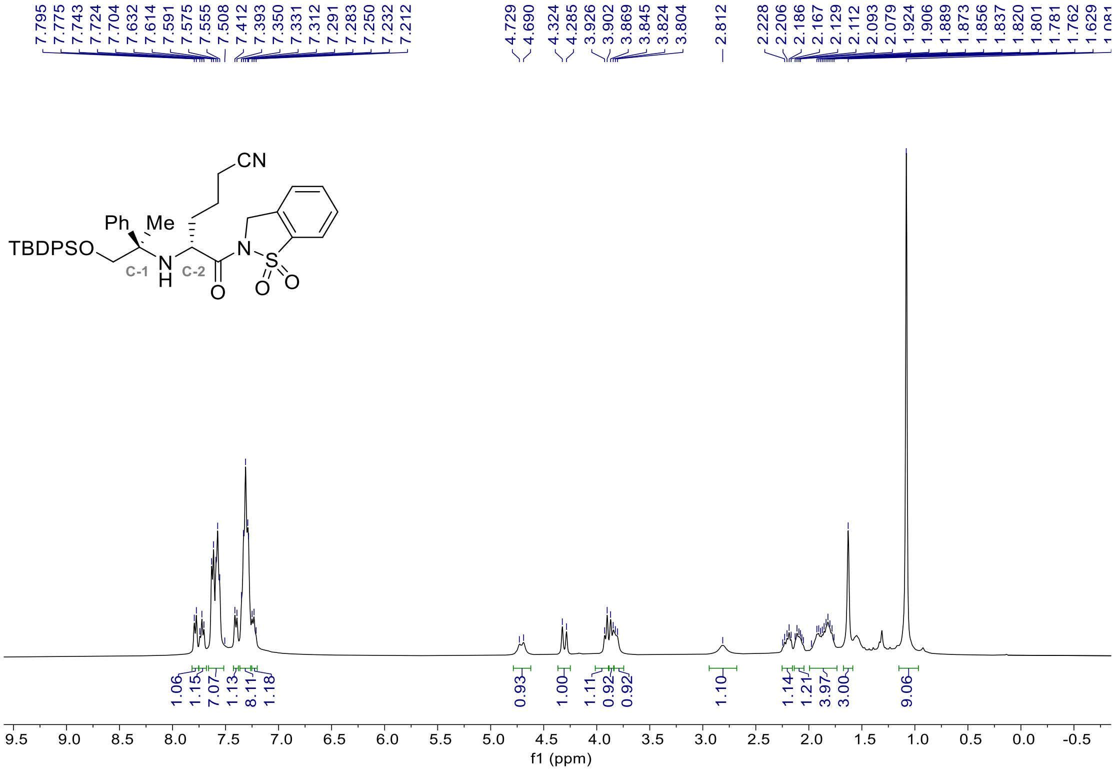

^{13}C NMR (101 MHz, $CDCl_3$) – ($R_{C\text{-}1}$, $R_{C\text{-}2}$)-**28b**

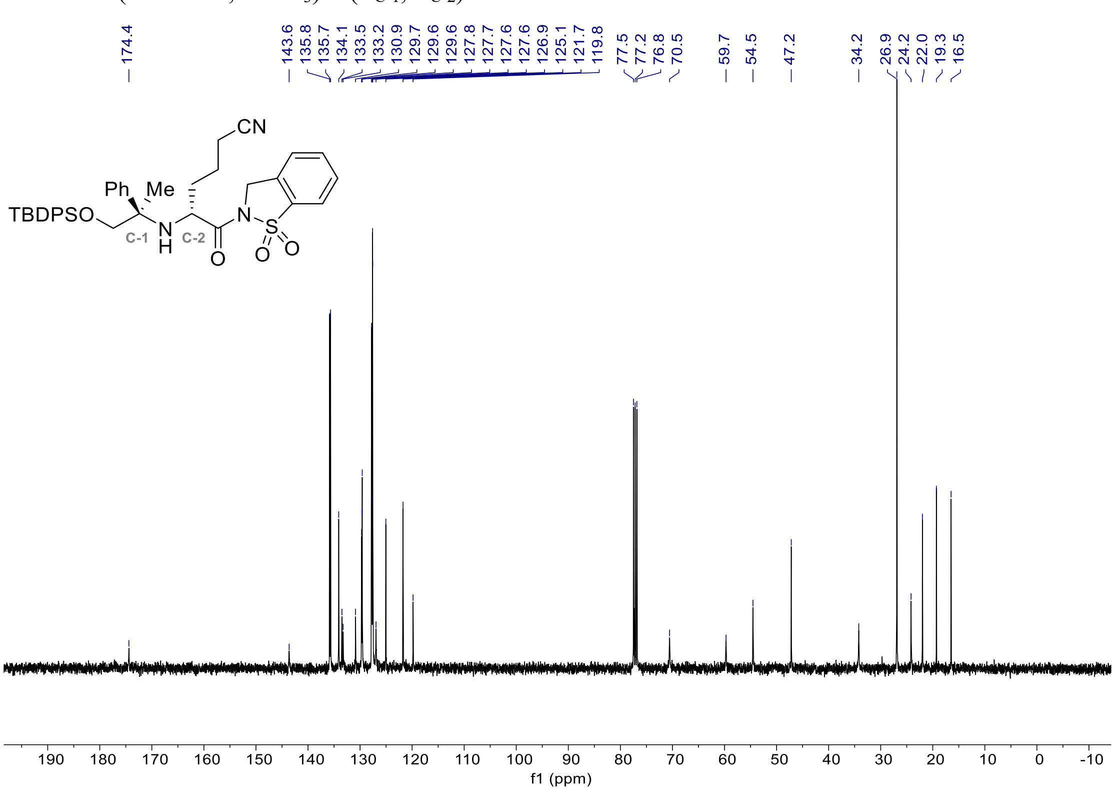

^{1}H NMR (400 MHz, $CDCl_3$) – ($S_{C\text{-}1}$, $S_{C\text{-}2}$, $S_{C\text{-}3}$)-**29a**

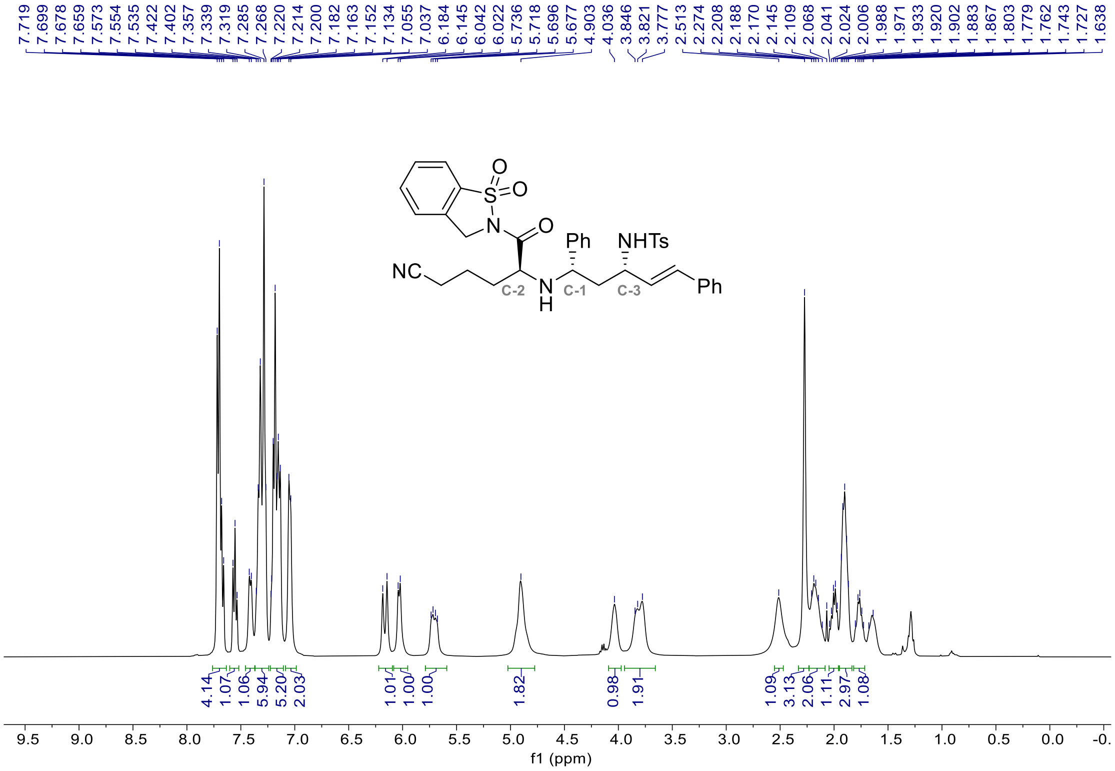

^{13}C NMR (101 MHz, $CDCl_3$) – ($S_{C\text{-}1}$, $S_{C\text{-}2}$, $S_{C\text{-}3}$)-**29a**

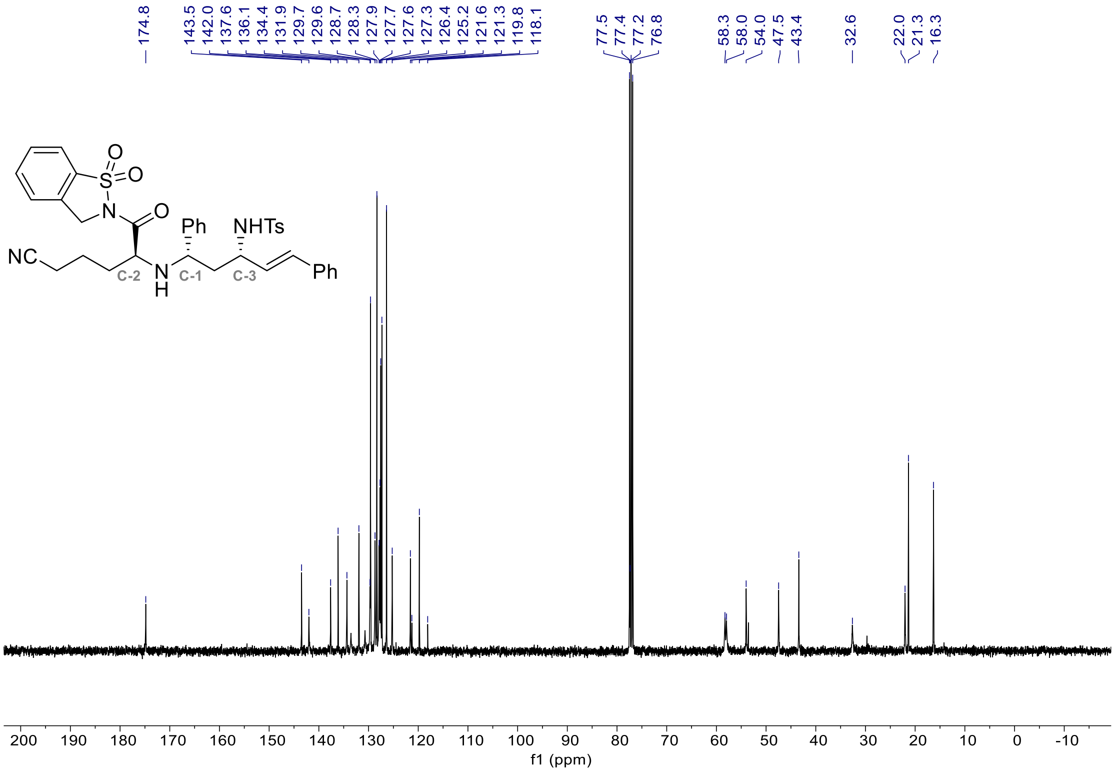

^{1}H NMR (400 MHz, $CDCl_3$) – (R_{C-1}, S_{C-2}, S_{C-3})-**29b**

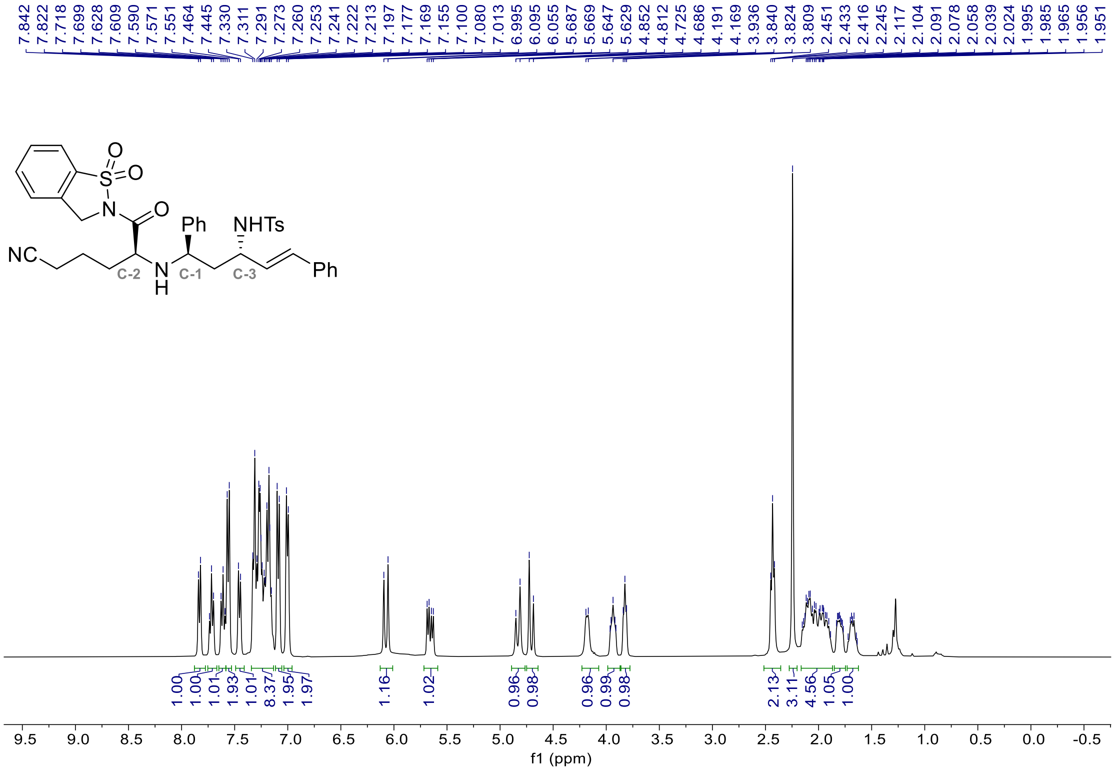

^{13}C NMR (101 MHz, $CDCl_3$) – (R_{C-1}, S_{C-2}, S_{C-3})-**29b**

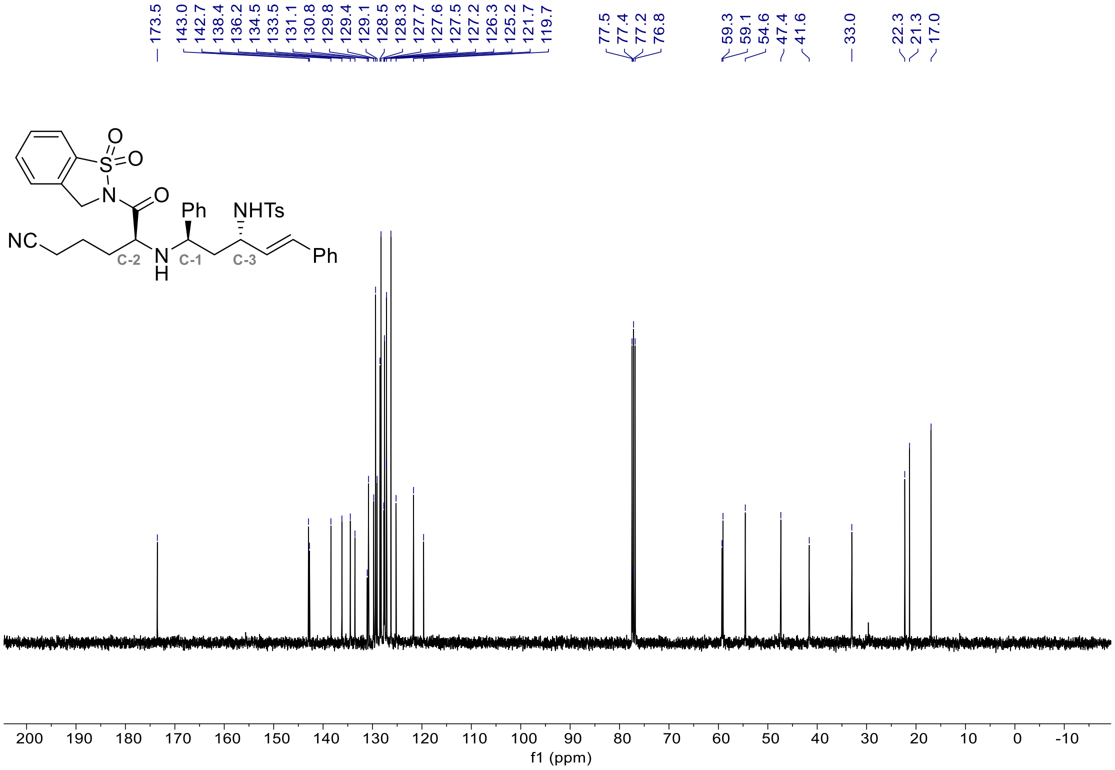

^{1}H NMR (400 MHz, $CDCl_3$) – (S_{C-1}, S_{C-2}, S_{C-3})-**30a**

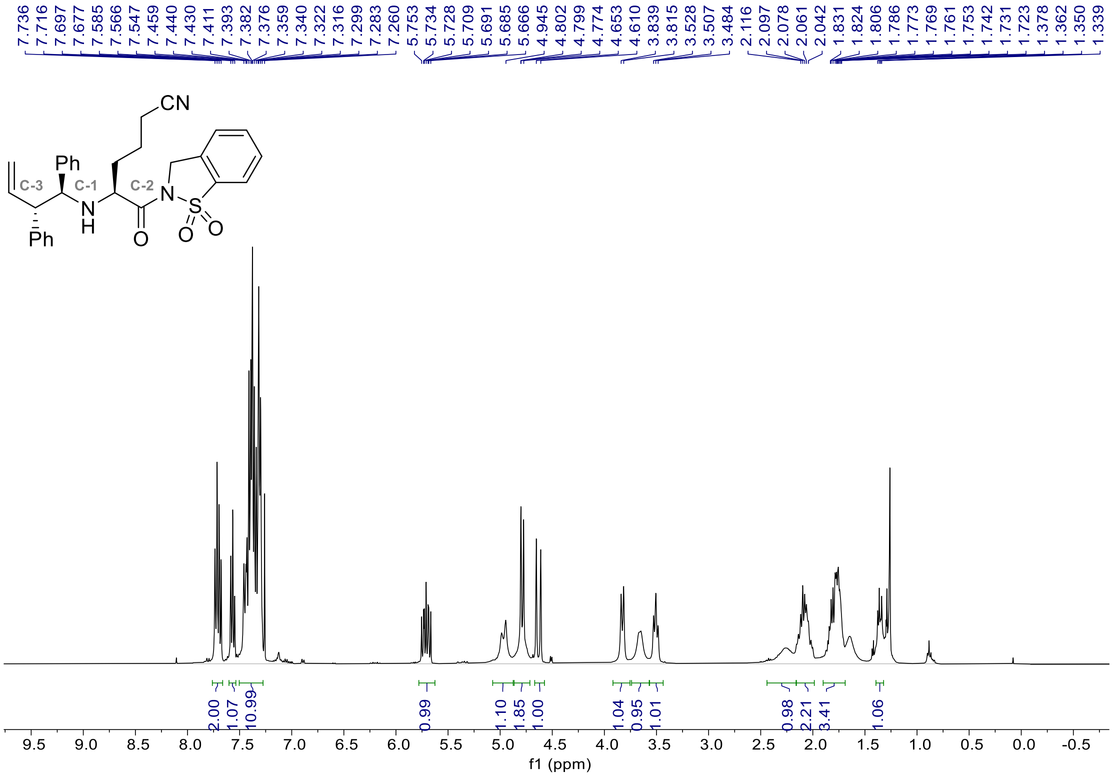

^{13}C NMR (101 MHz, $CDCl_3$) – (S_{C-1}, S_{C-2}, S_{C-3})-**30a**

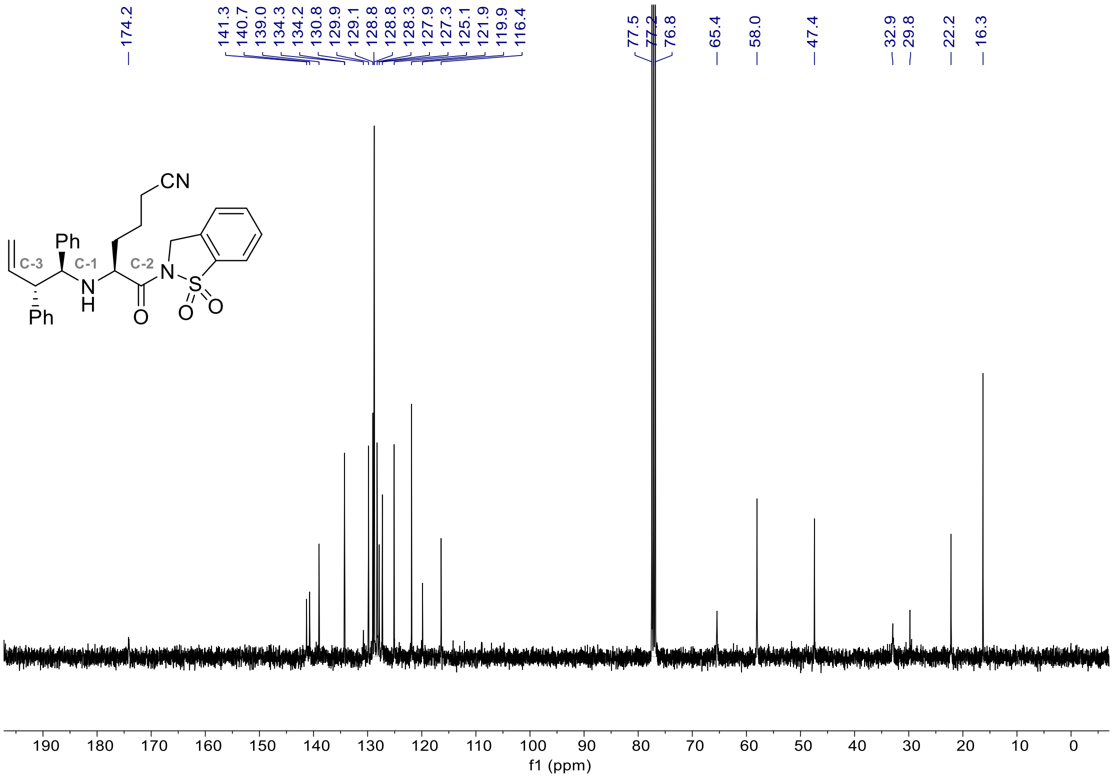

¹H NMR (400 MHz, $CDCl_3$) – ($S_{S(IV)}$, $R_{C\text{-}1}$, $R_{C\text{-}3}$)-**S30b**

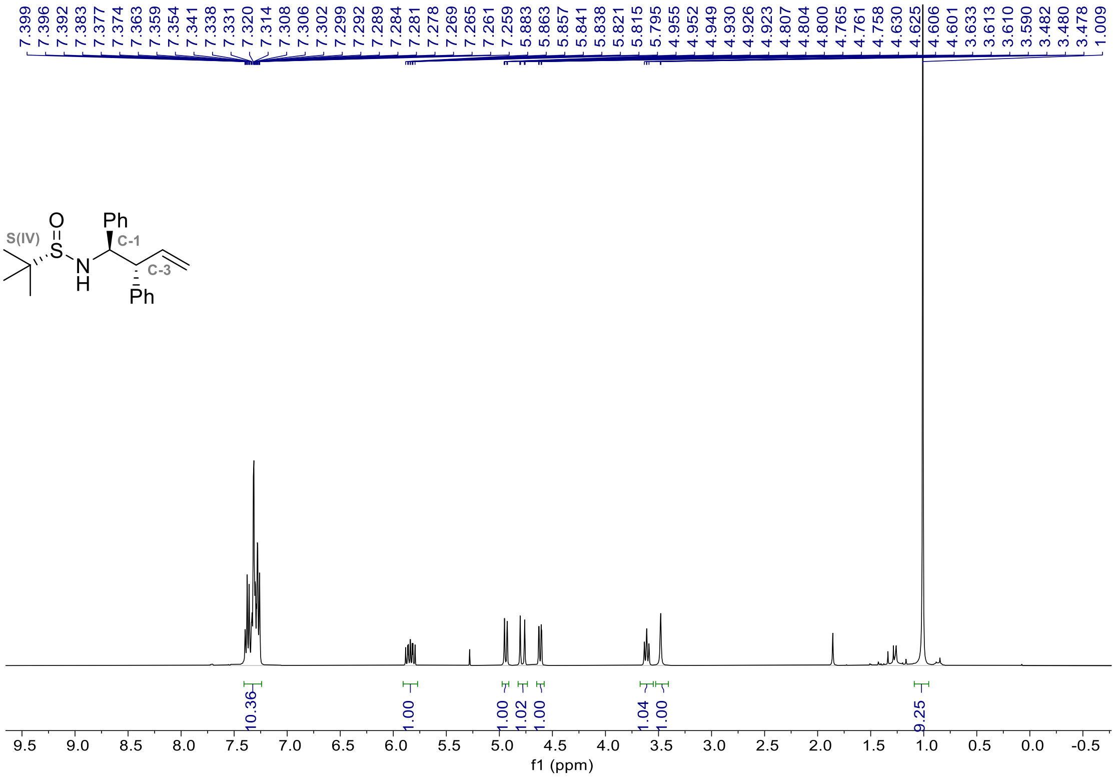

¹³C NMR (101 MHz, $CDCl_3$) – ($S_{S(IV)}$, $R_{C\text{-}1}$, $R_{C\text{-}3}$)-**S30b**

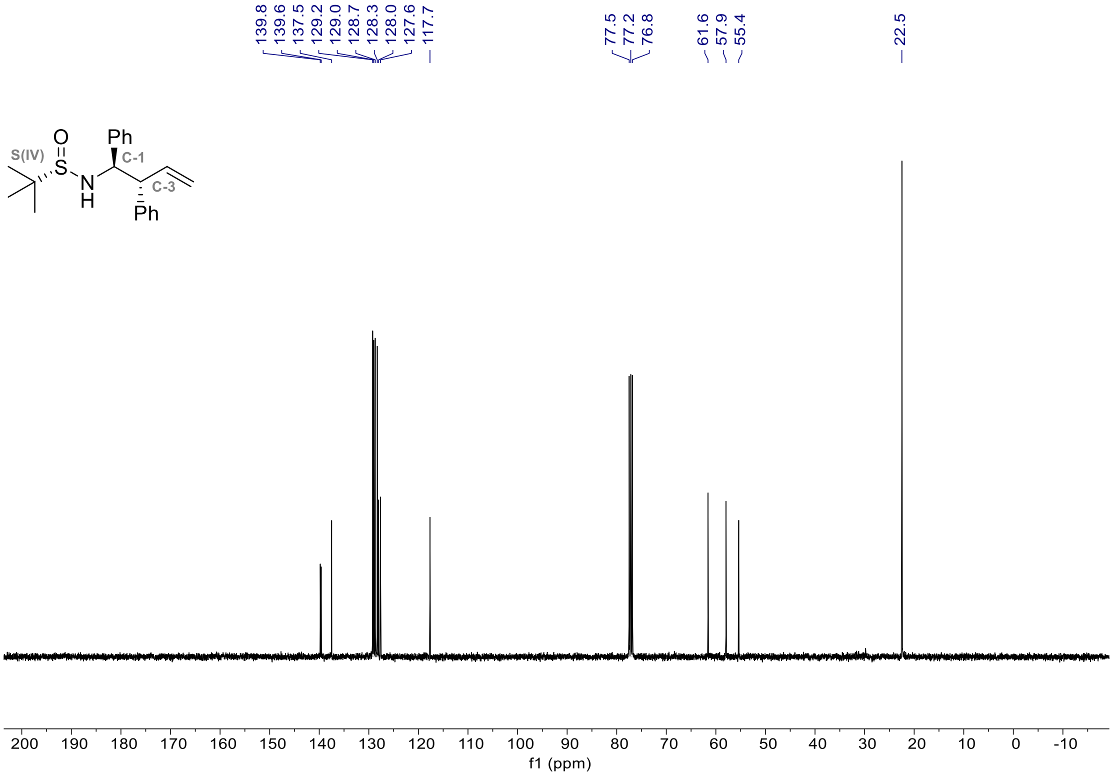

^{1}H NMR (400 MHz, $CDCl_3$) – ($R_{C\text{-}1}$, $R_{C\text{-}2}$, $R_{C\text{-}3}$)-**30b**

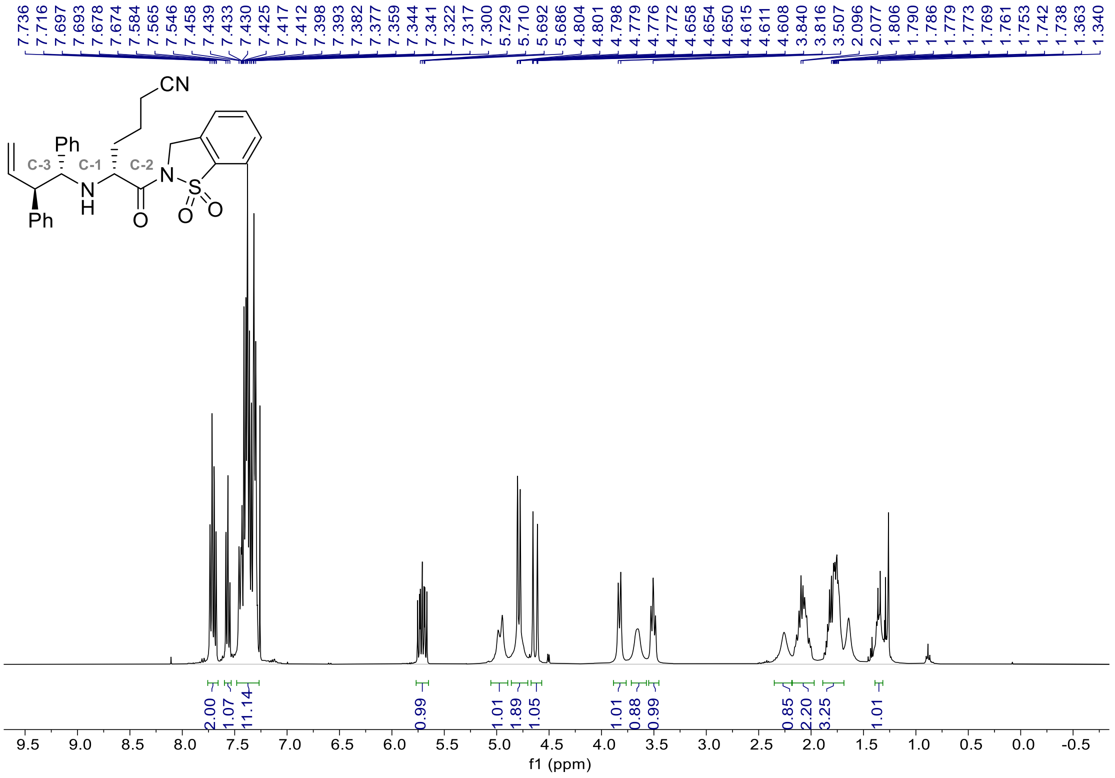

^{13}C NMR (101 MHz, $CDCl_3$) – ($R_{C\text{-}1}$, $R_{C\text{-}2}$, $R_{C\text{-}3}$)-**30b**

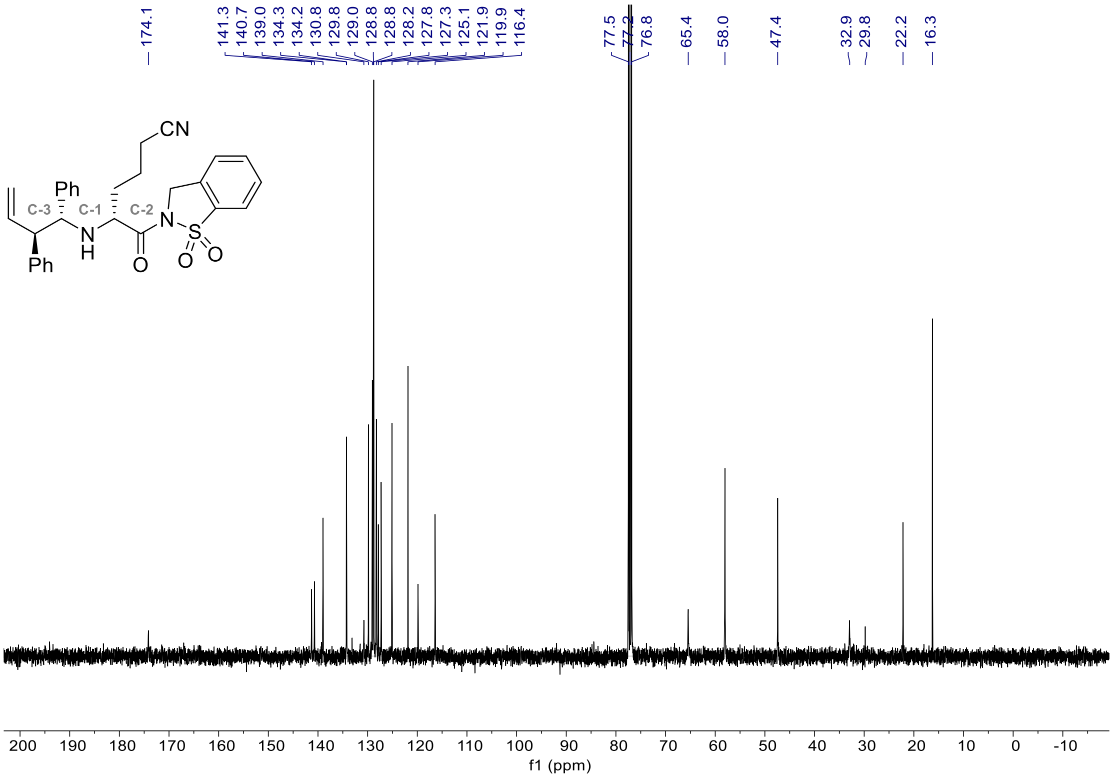

^{1}H NMR (400 MHz, $CDCl_3$) – ($R_{S(IV)}$, S_{C-1})-**S31a**

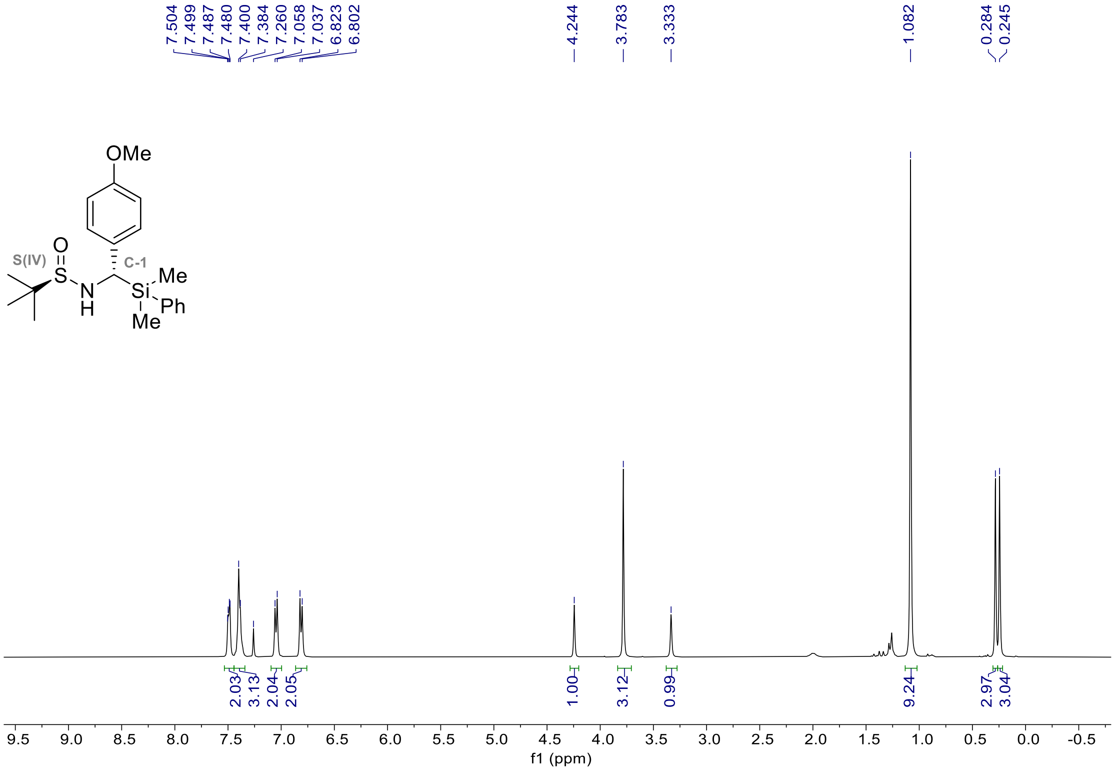

^{13}C NMR (101 MHz, $CDCl_3$) – ($R_{S(IV)}$, S_{C-1})-**S31a**

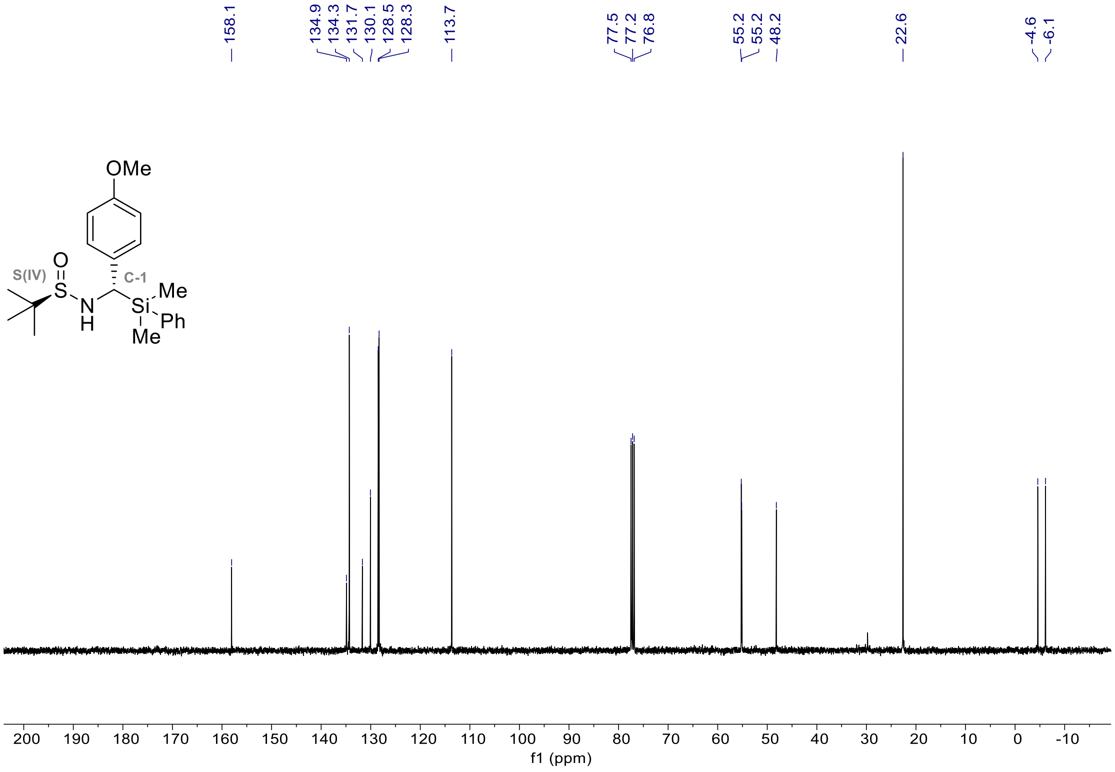

^{1}H NMR (400 MHz, $CDCl_3$) – ($S_{C\text{-}1}$, $S_{C\text{-}2}$)-**31a**

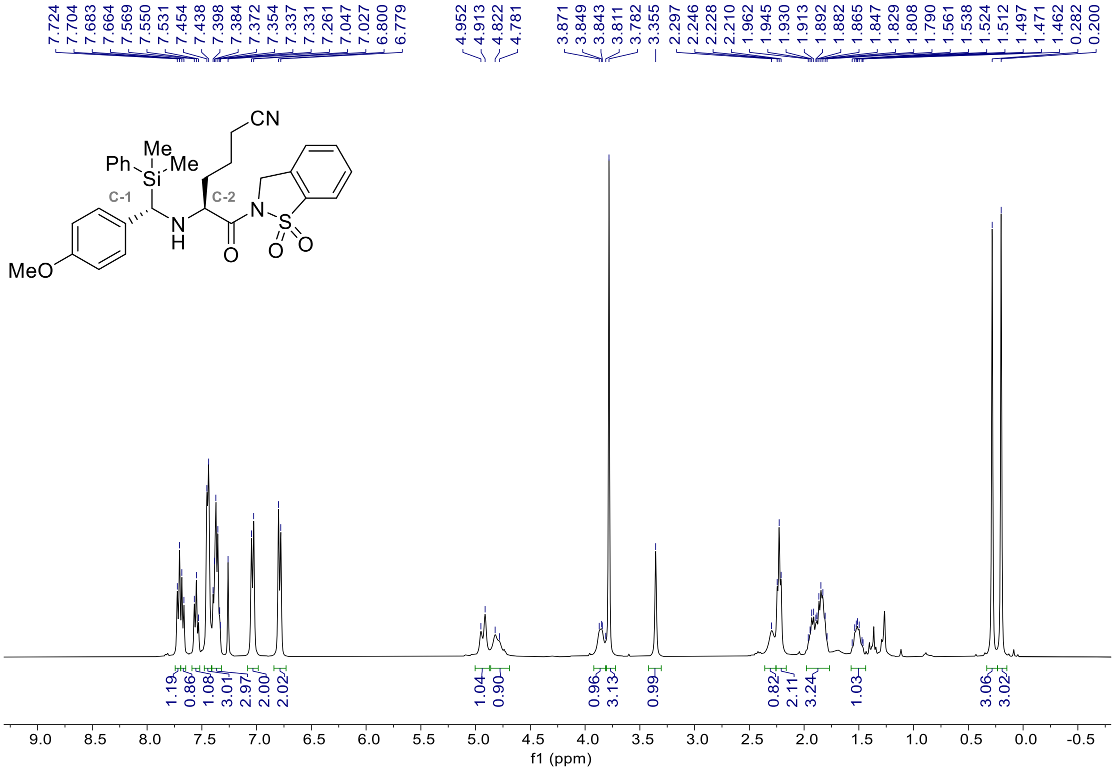

^{13}C NMR (101 MHz, $CDCl_3$) – ($S_{C\text{-}1}$, $S_{C\text{-}2}$)-**31a**

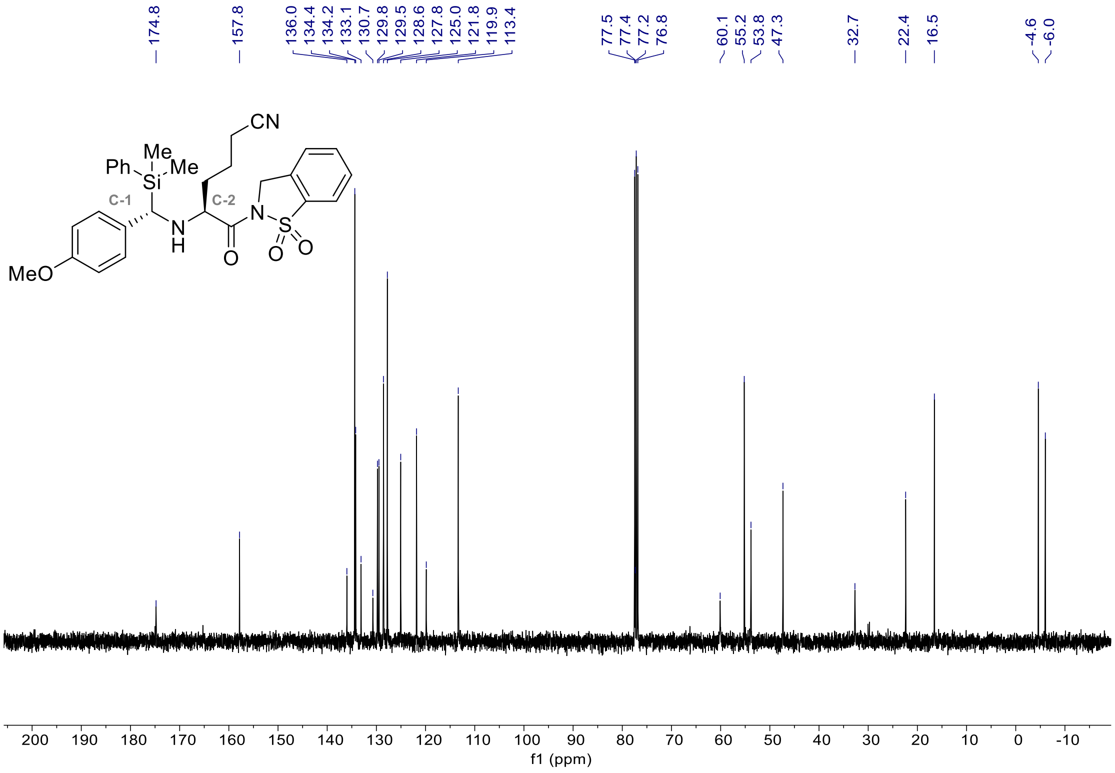

^{1}H NMR (400 MHz, $CDCl_3$) – ($R_{C\text{-}1}$, $S_{C\text{-}2}$)-**31b**

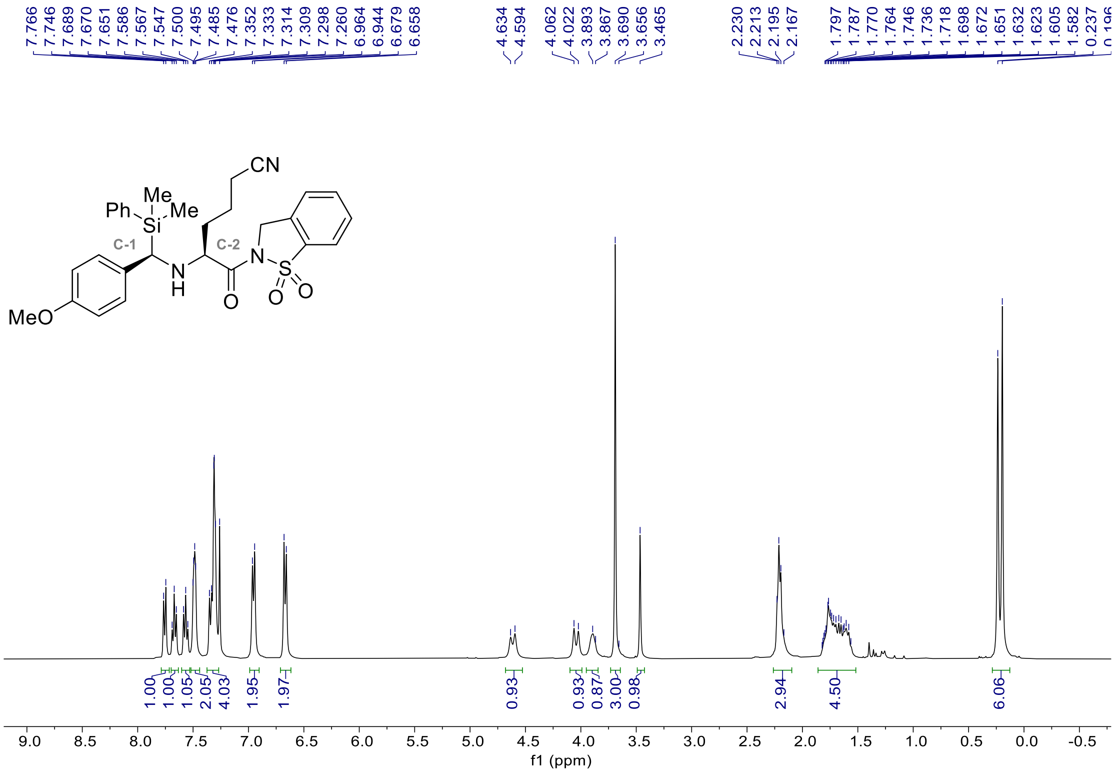

^{13}C NMR (101 MHz, $CDCl_3$) – ($R_{C\text{-}1}$, $S_{C\text{-}2}$)-**31b**

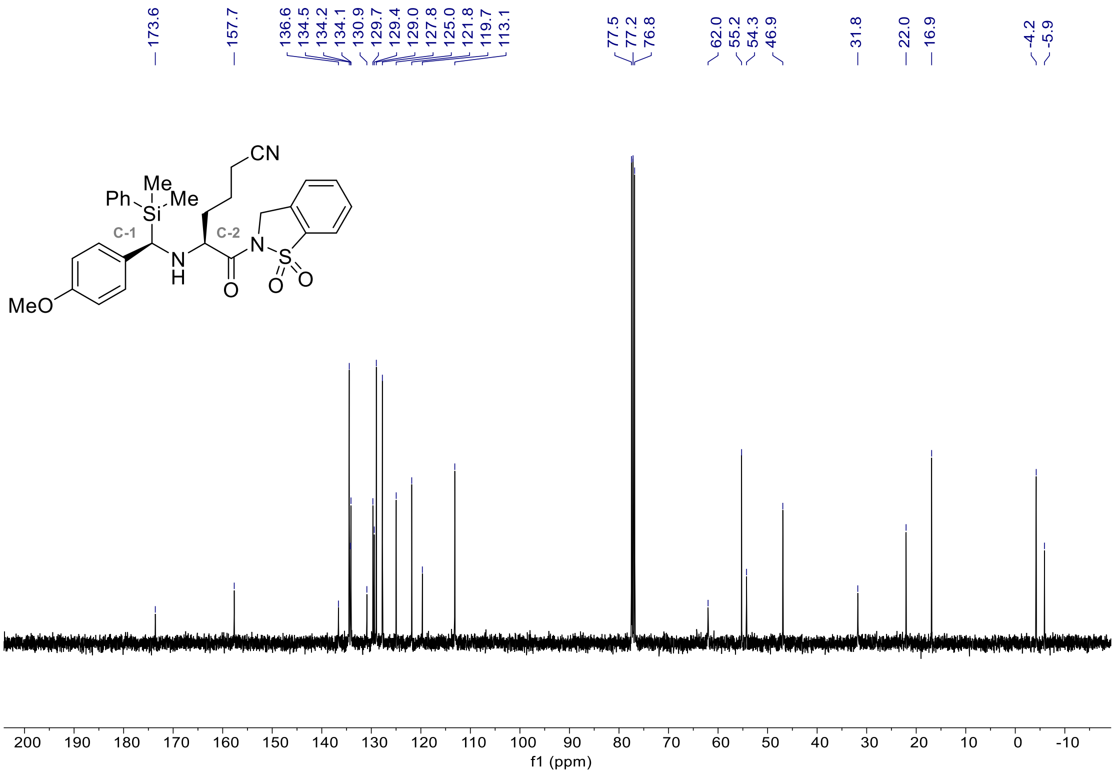

^{1}H NMR (400 MHz, $CDCl_3$) – ($R_{S(IV)}$, $R_{C\text{-}1}$)-**S32a**

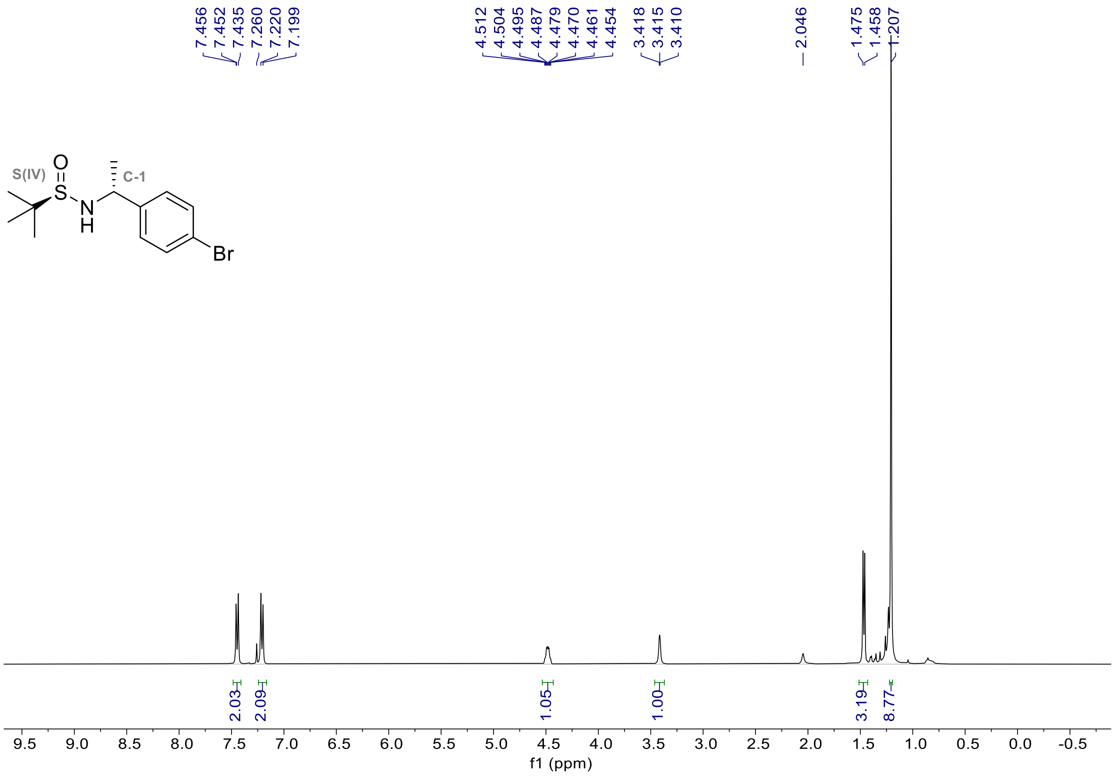

^{13}C NMR (101 MHz, $CDCl_3$) – ($R_{S(IV)}$, $R_{C\text{-}1}$)-**S32a**

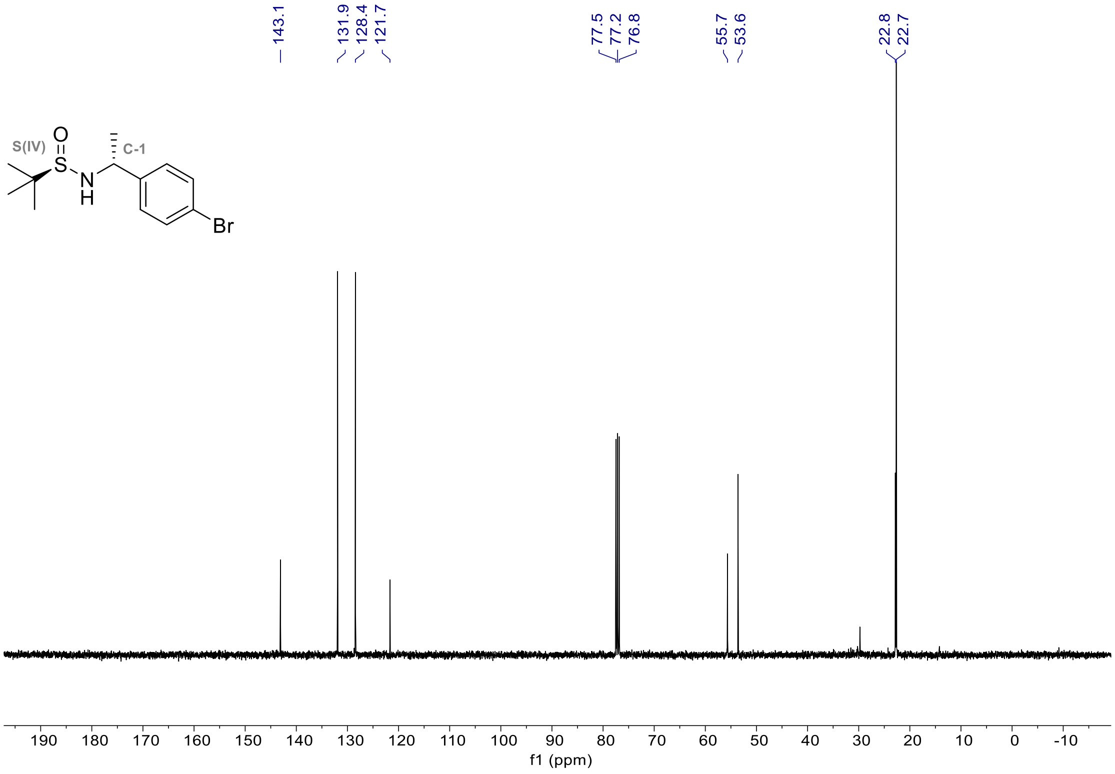

^{1}H NMR (400 MHz, $CDCl_3$) – ($R_{C\text{-}1}$, $S_{C\text{-}2}$)-**32a**

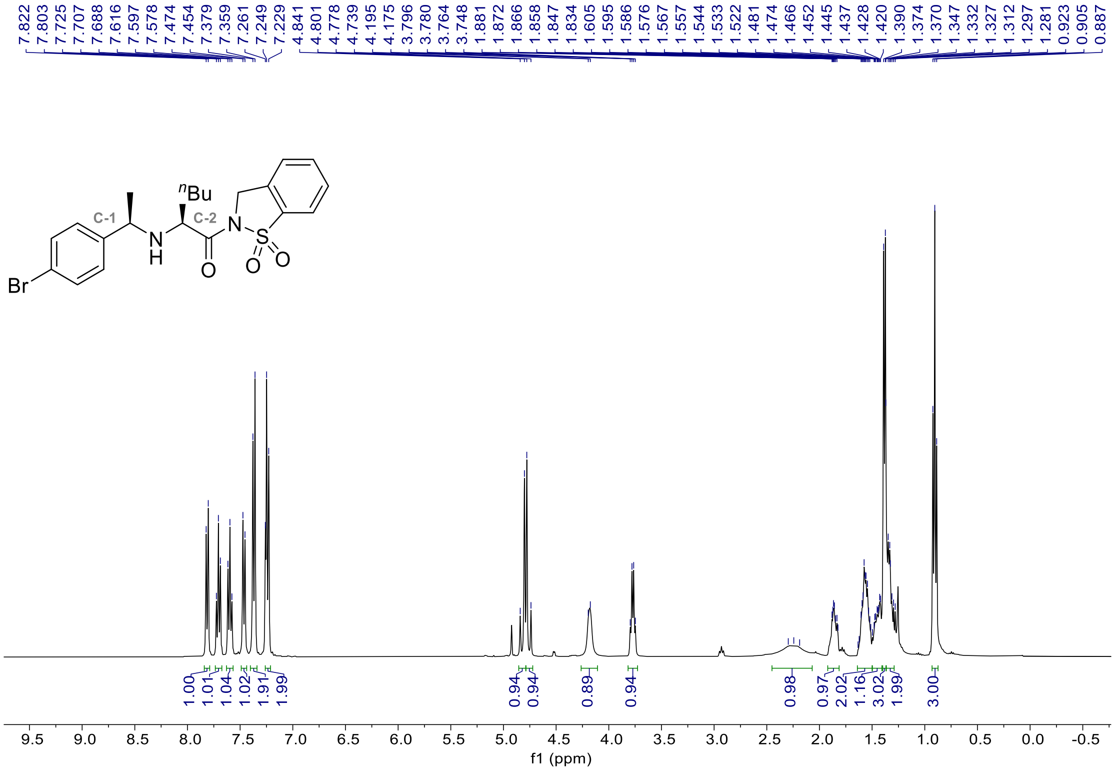

^{13}C NMR (101 MHz, $CDCl_3$) – ($R_{C\text{-}1}$, $S_{C\text{-}2}$)-**32a**

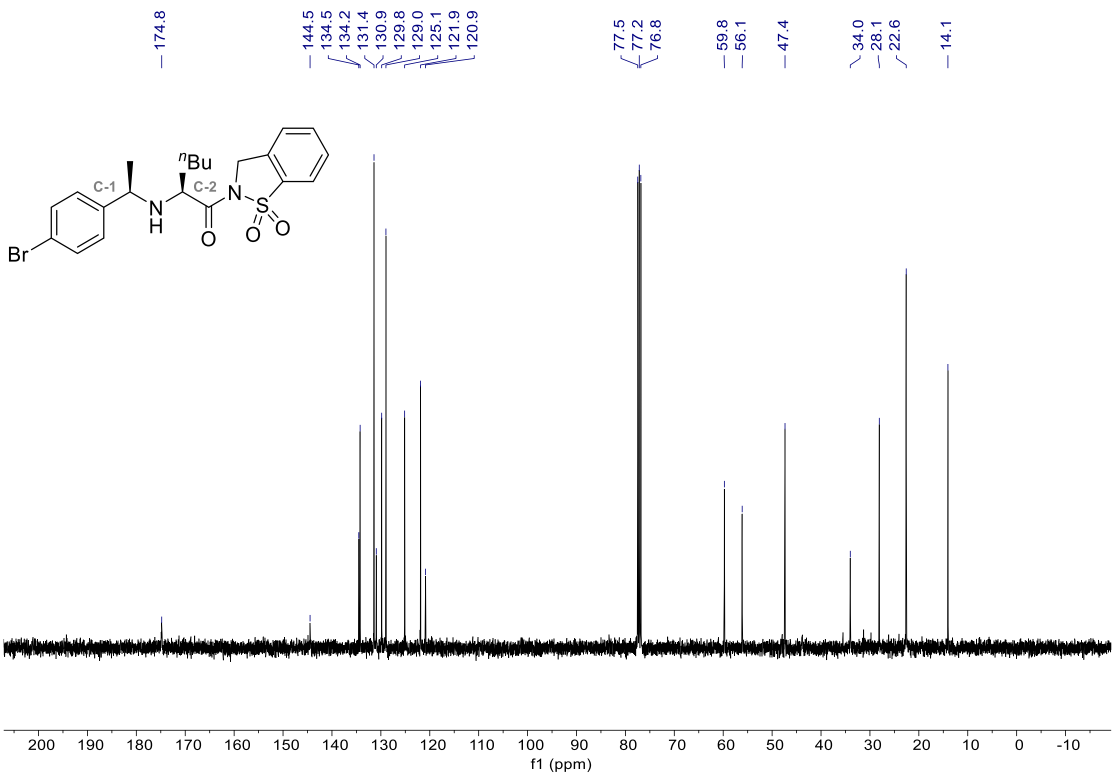

^{1}H NMR (400 MHz, $CDCl_3$) – ($S_{C\text{-}1}$, $S_{C\text{-}2}$)-**32b**

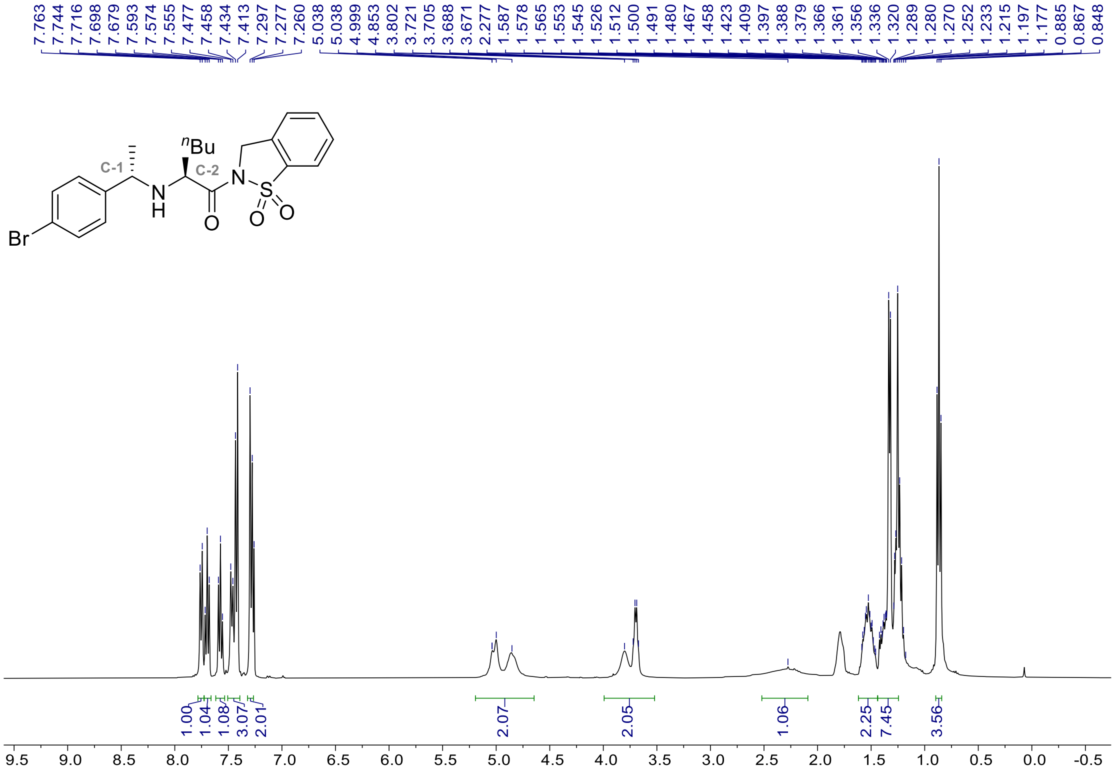

^{13}C NMR (101 MHz, $CDCl_3$) – ($S_{C\text{-}1}$, $S_{C\text{-}2}$)-**32b**

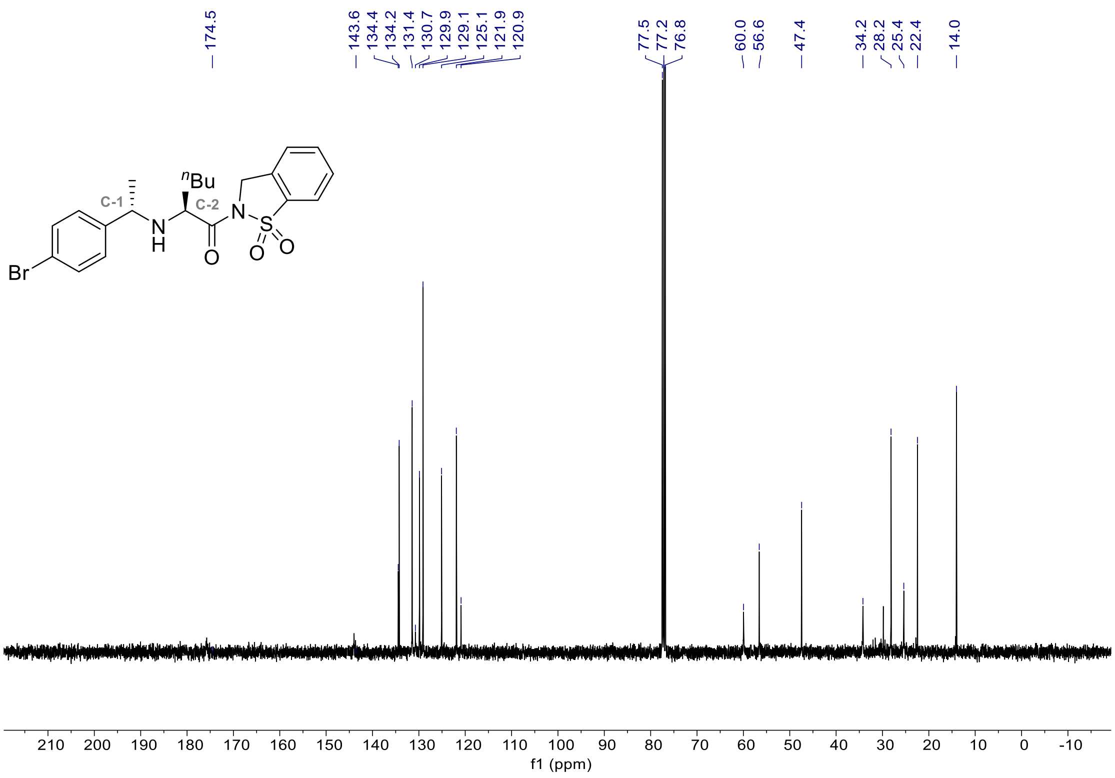

^{1}H NMR (400 MHz, $CDCl_3$) – (S_{C-1}, R_{C-2})-**32c**

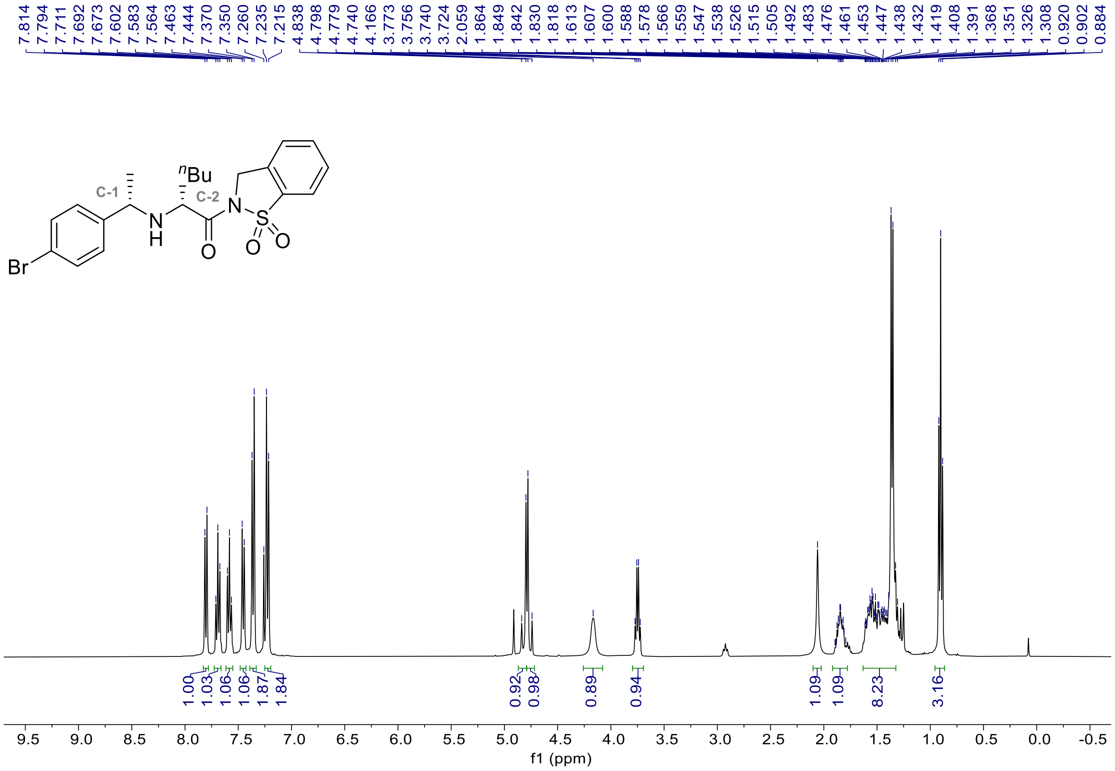

^{13}C NMR (101 MHz, $CDCl_3$) – (S_{C-1}, R_{C-2})-**32c**

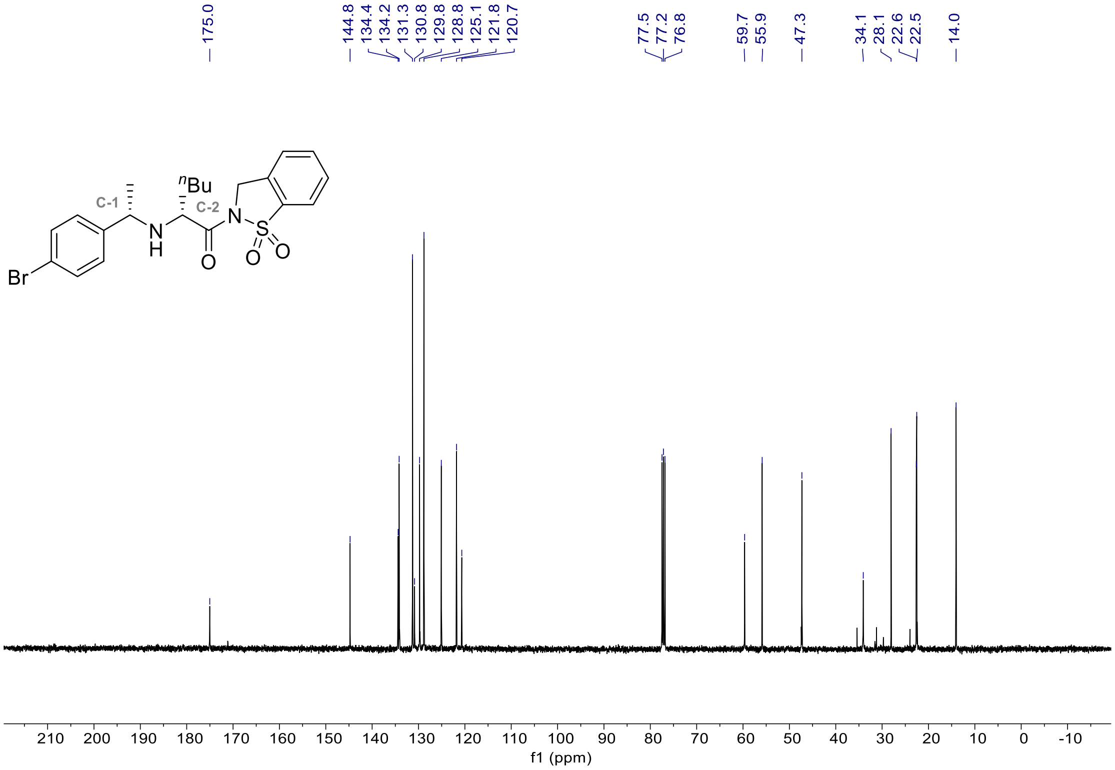

^{1}H NMR (400 MHz, $CDCl_3$) – ($R_{C\text{-}1}$, $R_{C\text{-}2}$)-**32d**

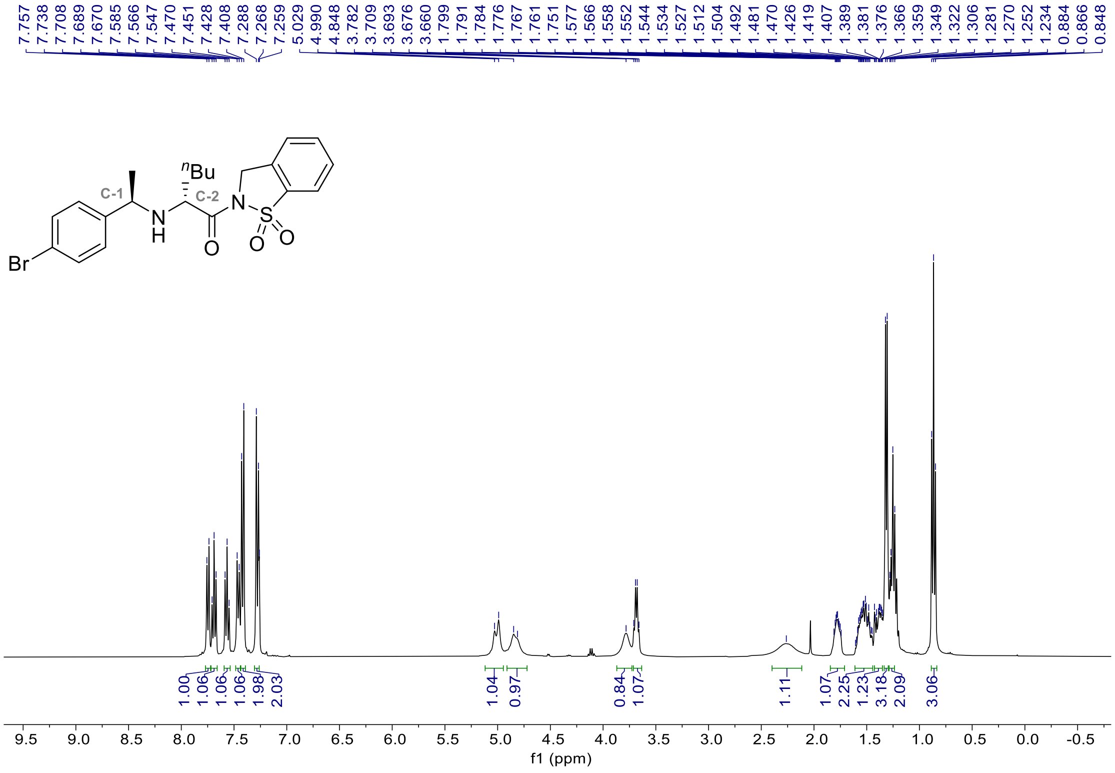

^{13}C NMR (101 MHz, $CDCl_3$) – ($R_{C\text{-}1}$, $R_{C\text{-}2}$)-**32d**

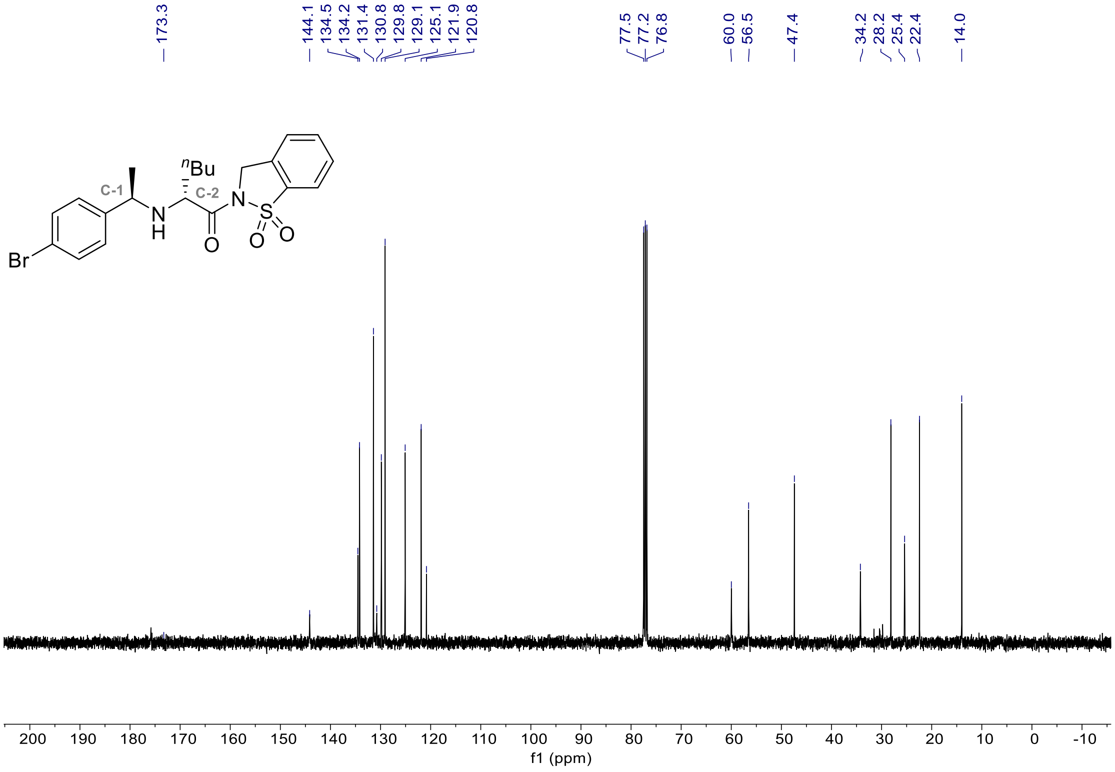

^{1}H NMR (400 MHz, $CDCl_3$) – ($R_{C\text{-}1}$, $S_{C\text{-}2}$)-**33a**

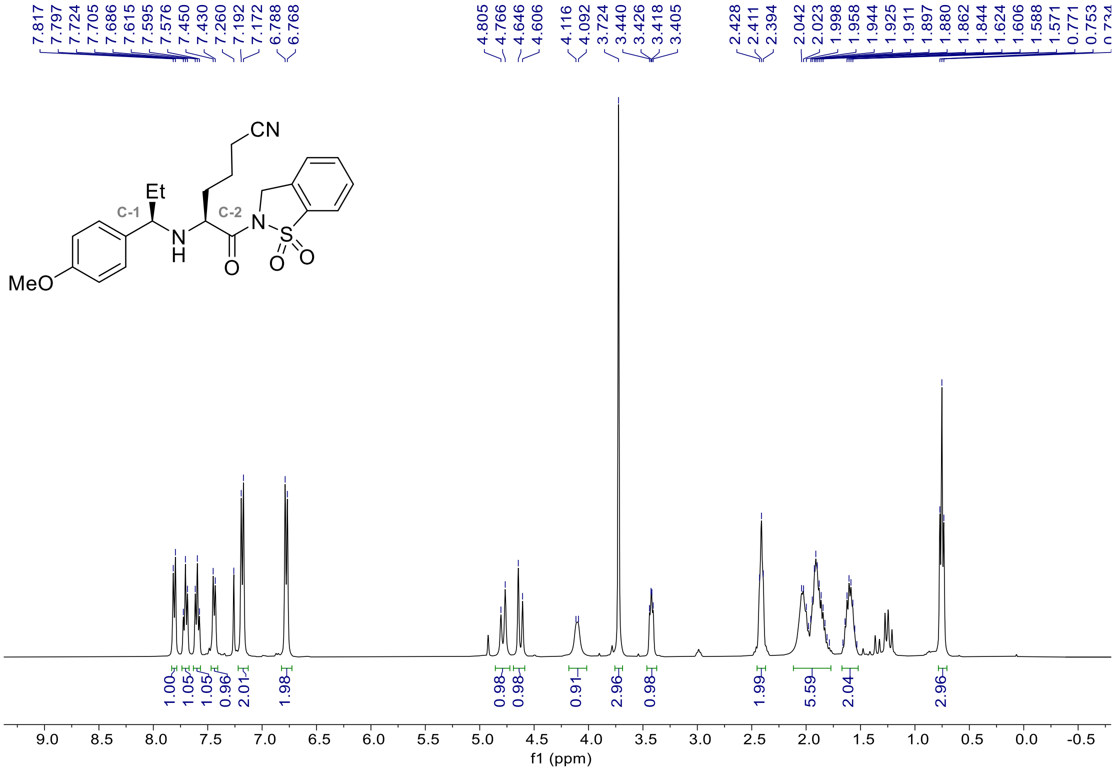

^{13}C NMR (101 MHz, $CDCl_3$) – ($R_{C\text{-}1}$, $S_{C\text{-}2}$)-**33a**

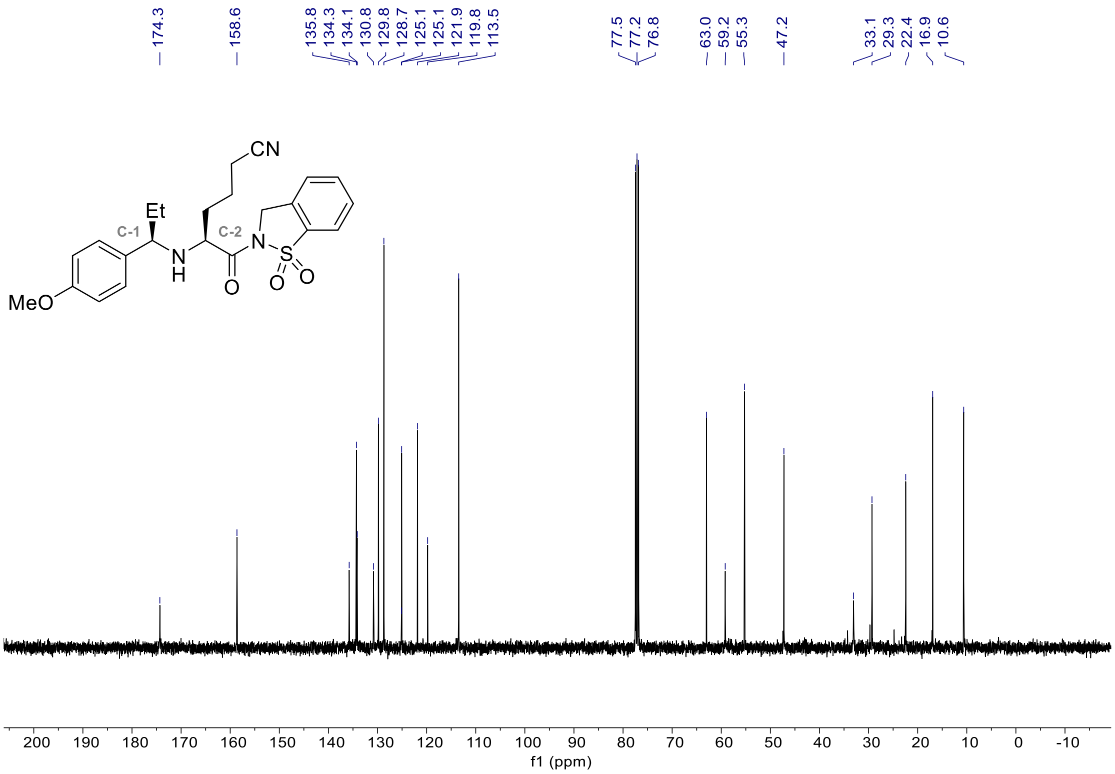

^{1}H NMR (400 MHz, $CDCl_3$) – (*S*$_{C-1}$, *S*$_{C-2}$)-**33b**

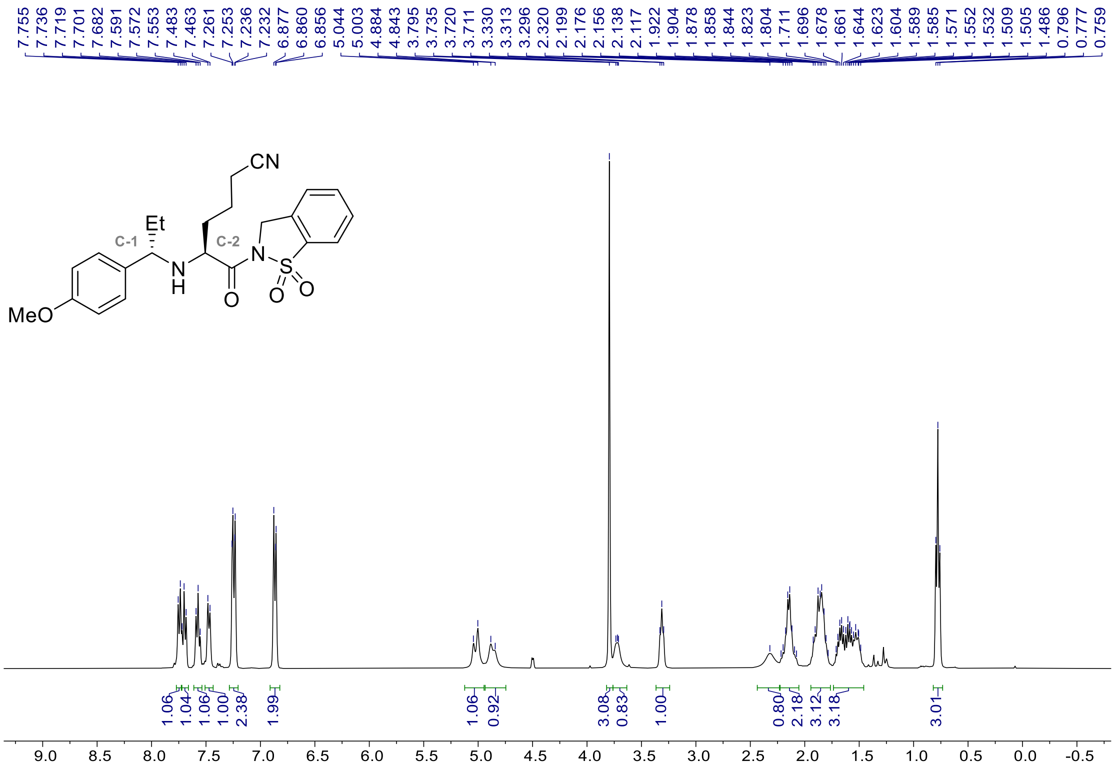

^{13}C NMR (101 MHz, $CDCl_3$) – (*S*$_{C-1}$, *S*$_{C-2}$)-**33b**

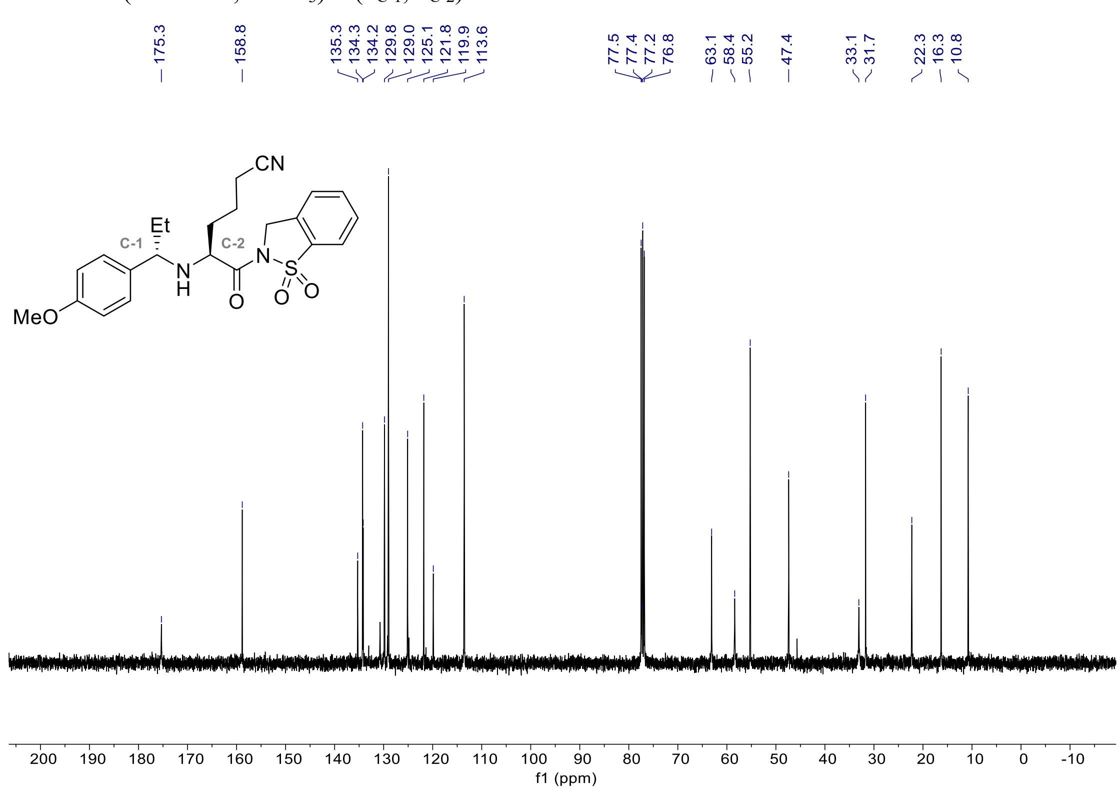

^{1}H NMR (400 MHz, $CDCl_3$) – ($S_{S(IV)}$, S_{C-1})-**S33c**

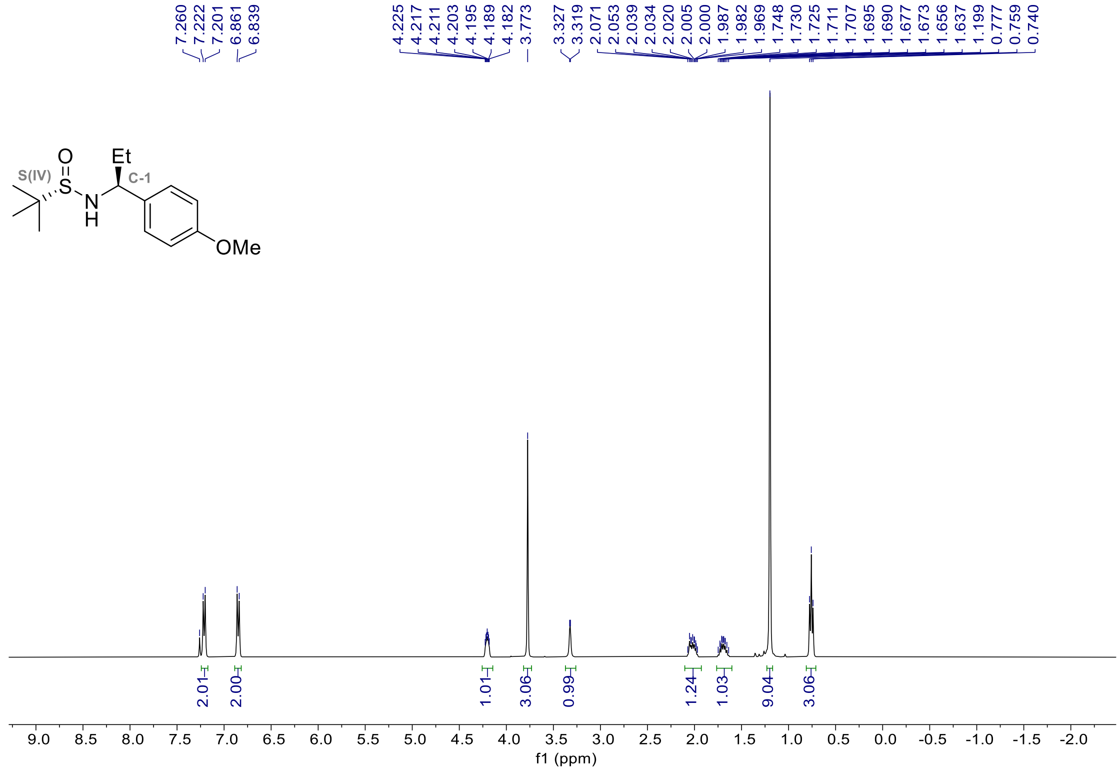

^{13}C NMR (101 MHz, $CDCl_3$) – ($S_{S(IV)}$, S_{C-1})-**S33c**

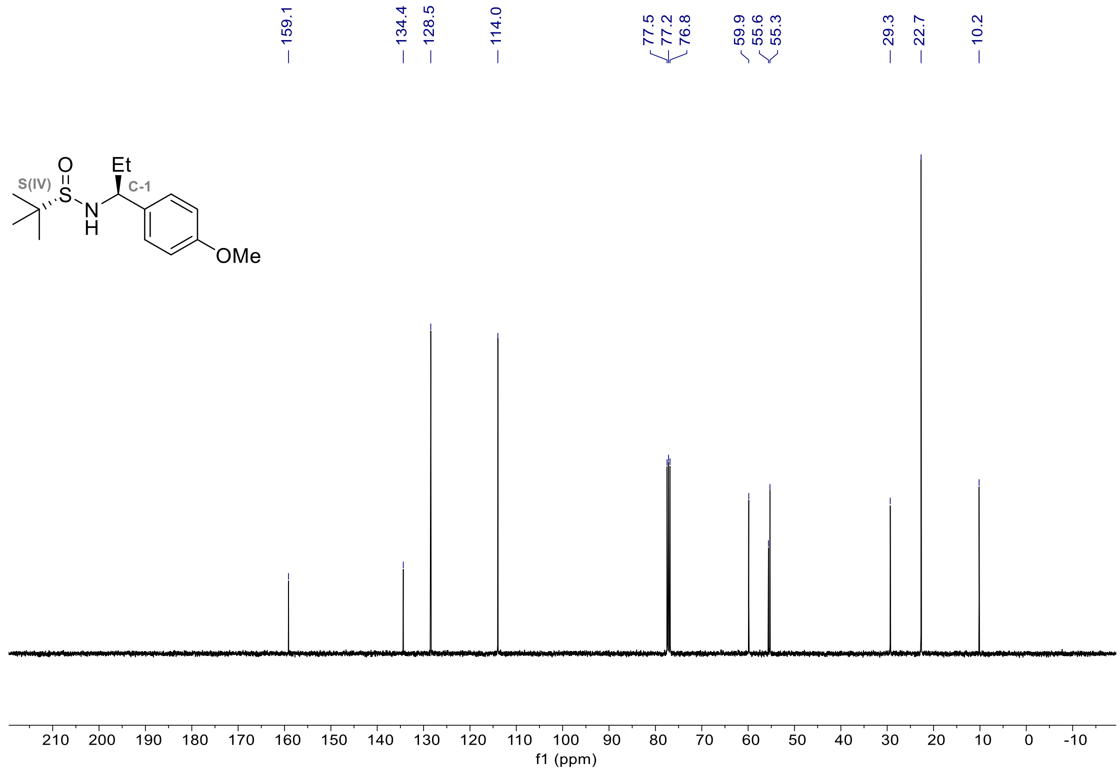

^{1}H NMR (400 MHz, CDCl$_3$) – ($S_{\text{C-1}}$, $R_{\text{C-2}}$)-**33c**

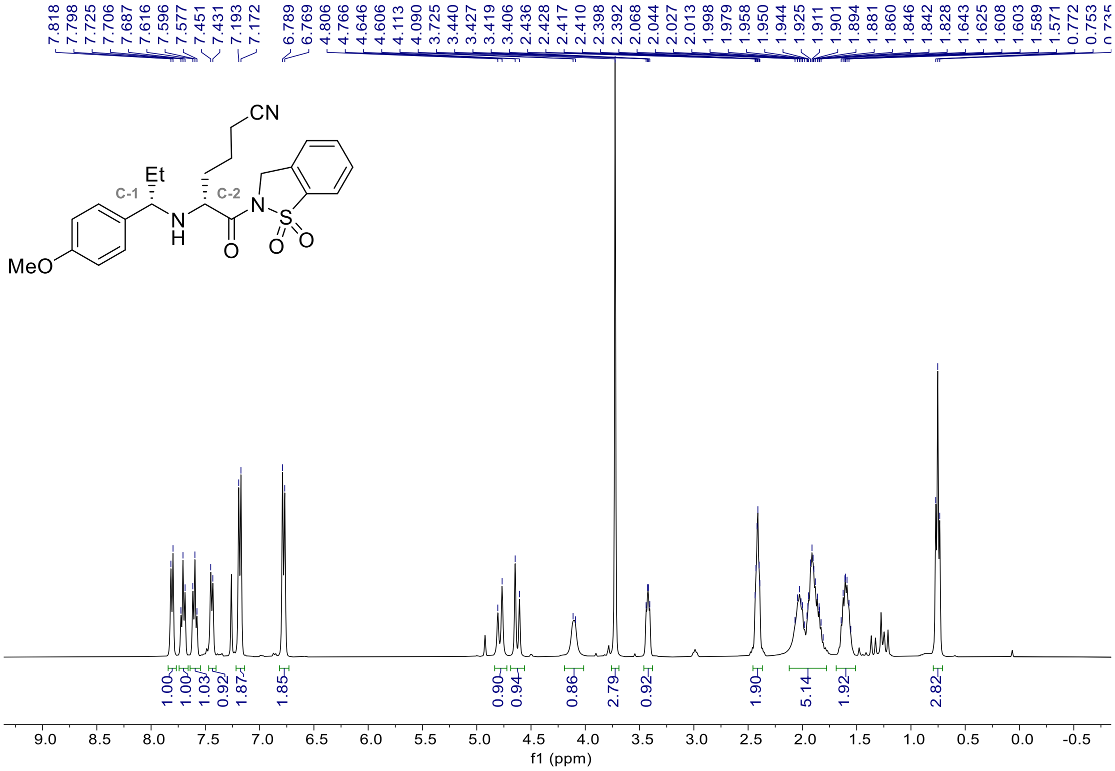

^{13}C NMR (101 MHz, CDCl$_3$) – ($S_{\text{C-1}}$, $R_{\text{C-2}}$)-**33c**

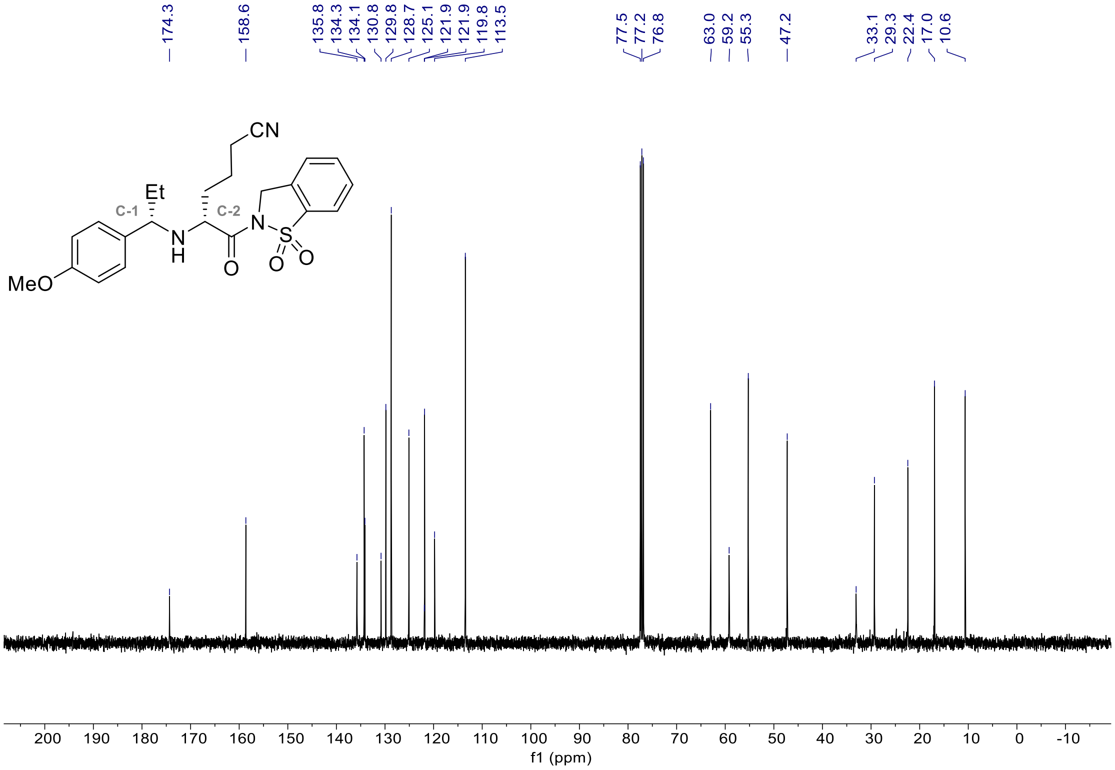

^{1}H NMR (400 MHz, $CDCl_3$) – ($S_{S(IV)}$, R_{C-1})-**S33d**

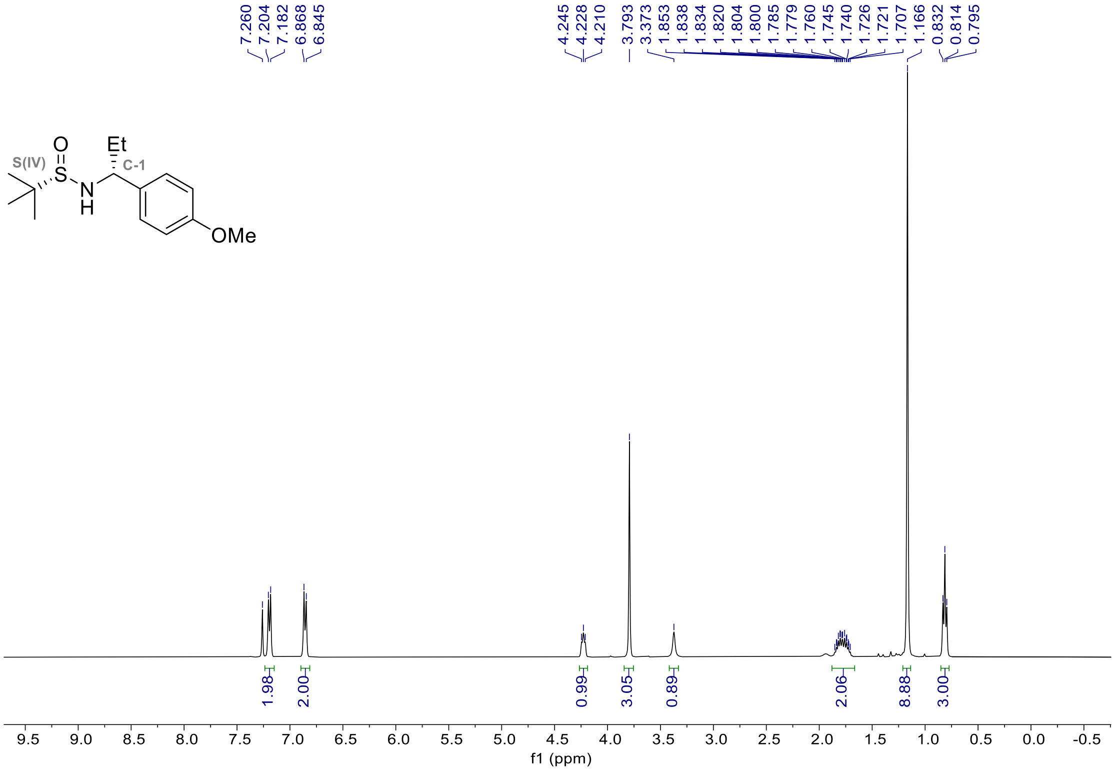

^{13}C NMR (101 MHz, $CDCl_3$) – ($S_{S(IV)}$, R_{C-1})-**S33d**

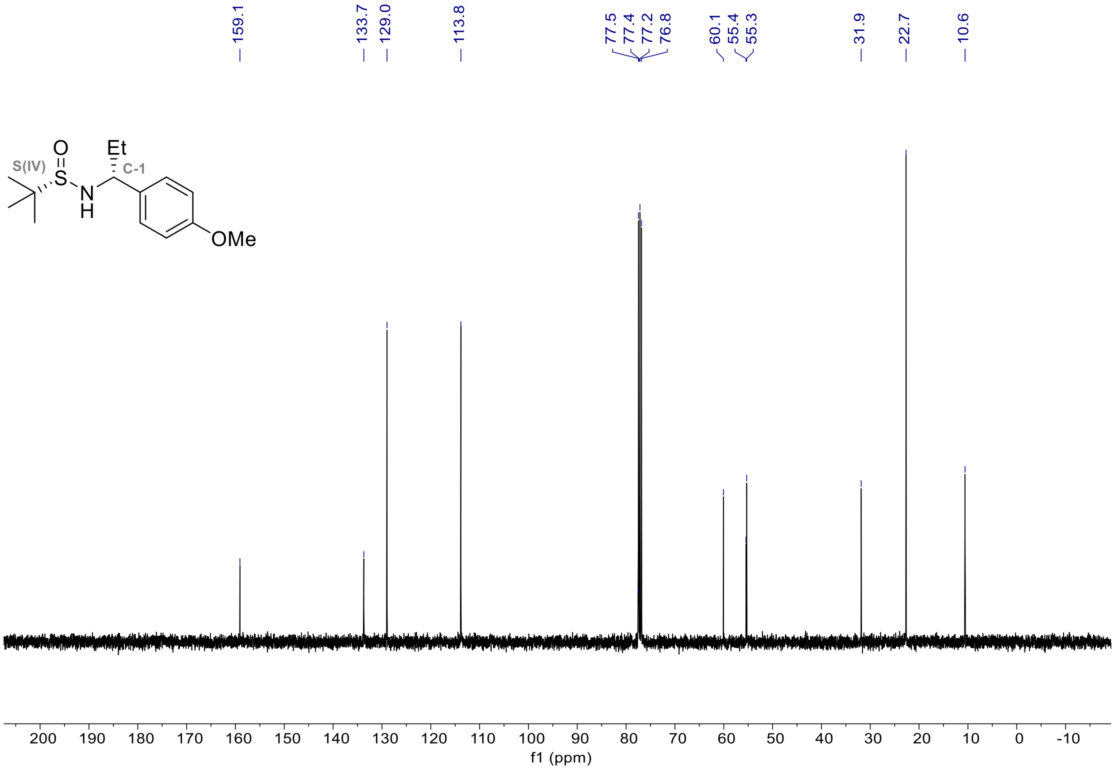

^{1}H NMR (400 MHz, $CDCl_3$) – ($R_{C\text{-}1}$, $R_{C\text{-}2}$)-**33d**

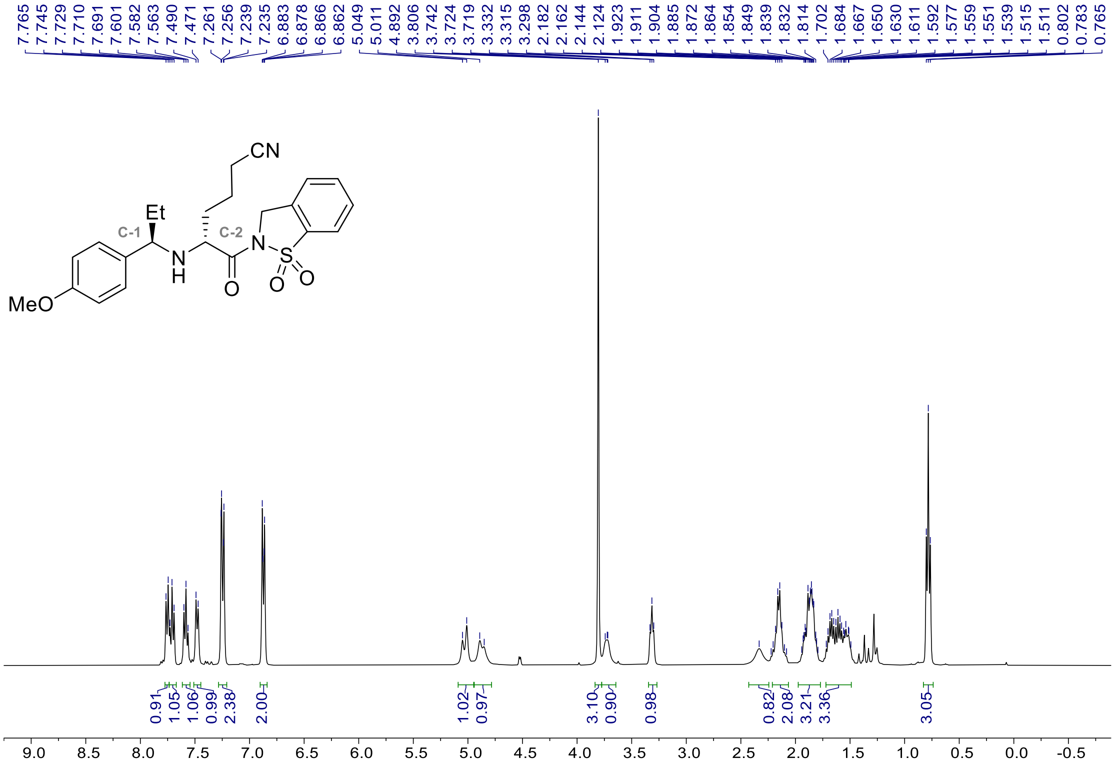

^{13}C NMR (101 MHz, $CDCl_3$) – ($R_{C\text{-}1}$, $R_{C\text{-}2}$)-**33d**

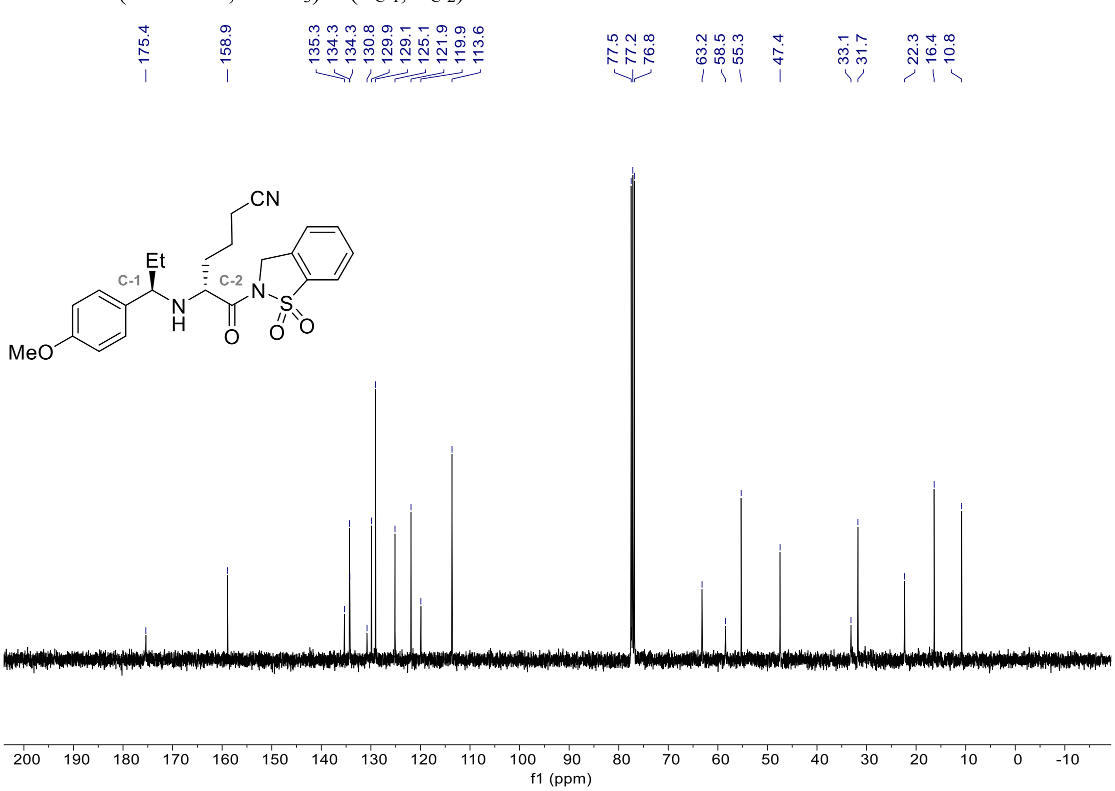

^{1}H NMR (400 MHz, $CDCl_3$) – ($R_{C\text{-}1}$, $S_{C\text{-}2}$)-**34**

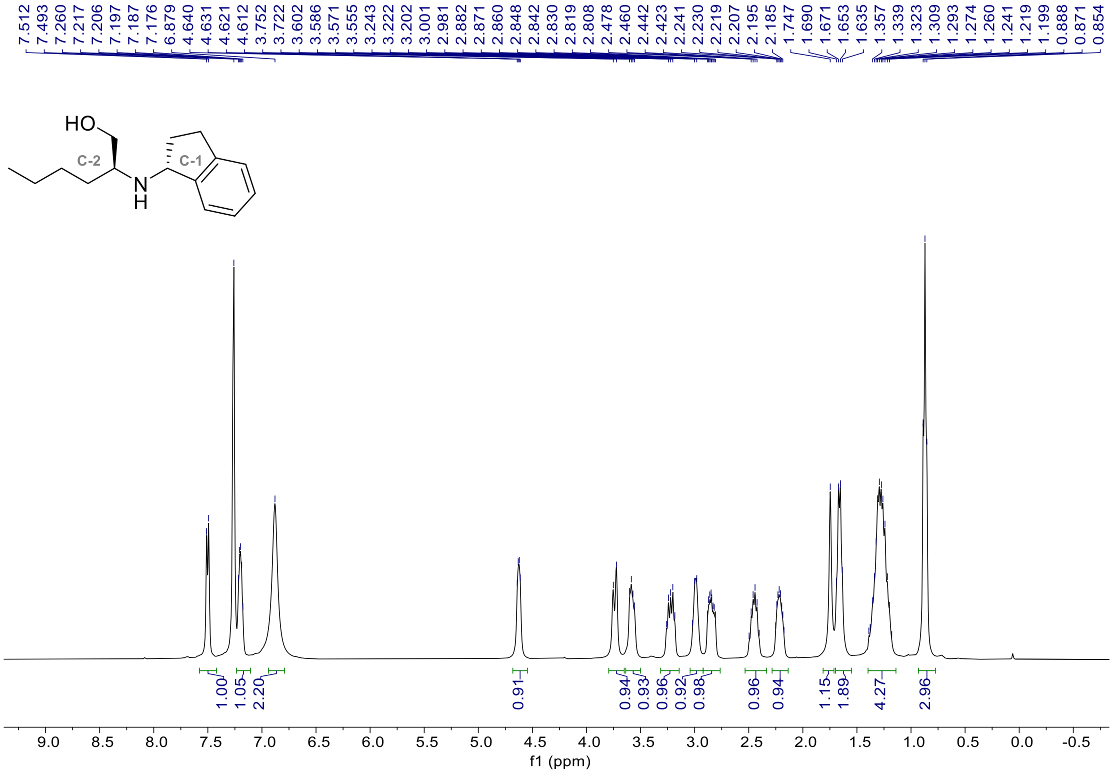

^{13}C NMR (101 MHz, $CDCl_3$) – ($R_{C\text{-}1}$, $S_{C\text{-}2}$)-**34**

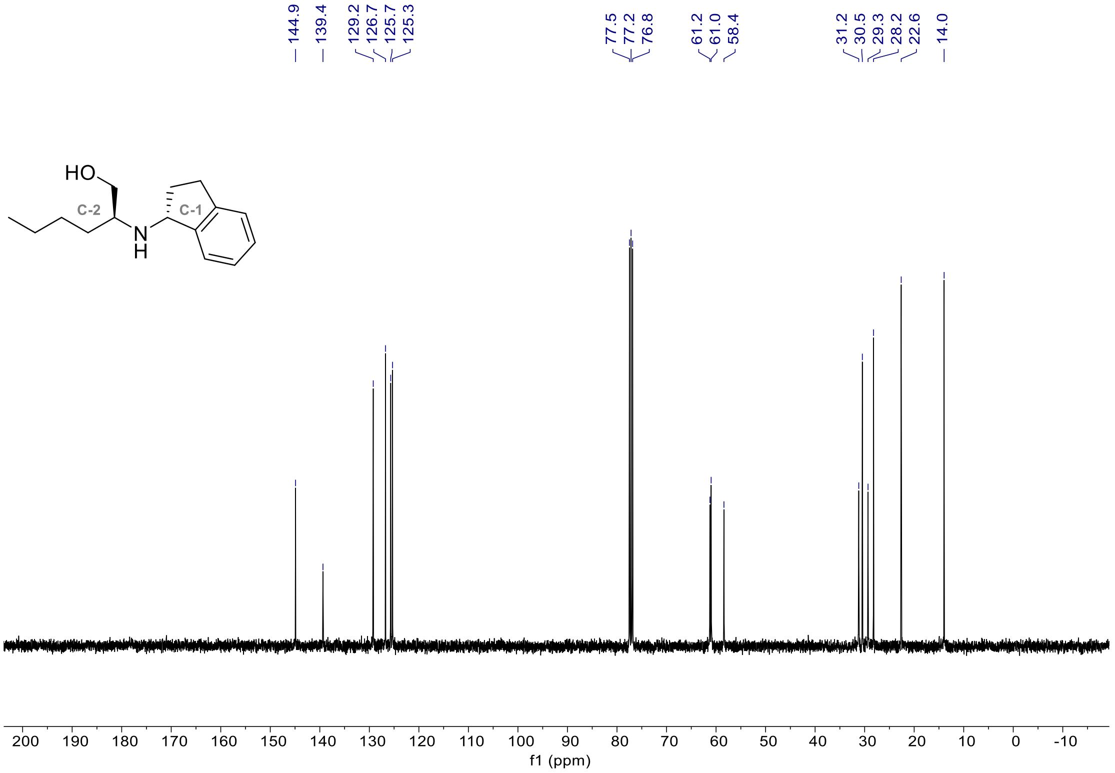

^{1}H NMR (400 MHz, CDCl$_3$) – (R_{C-1}, S_{C-2})-**35**

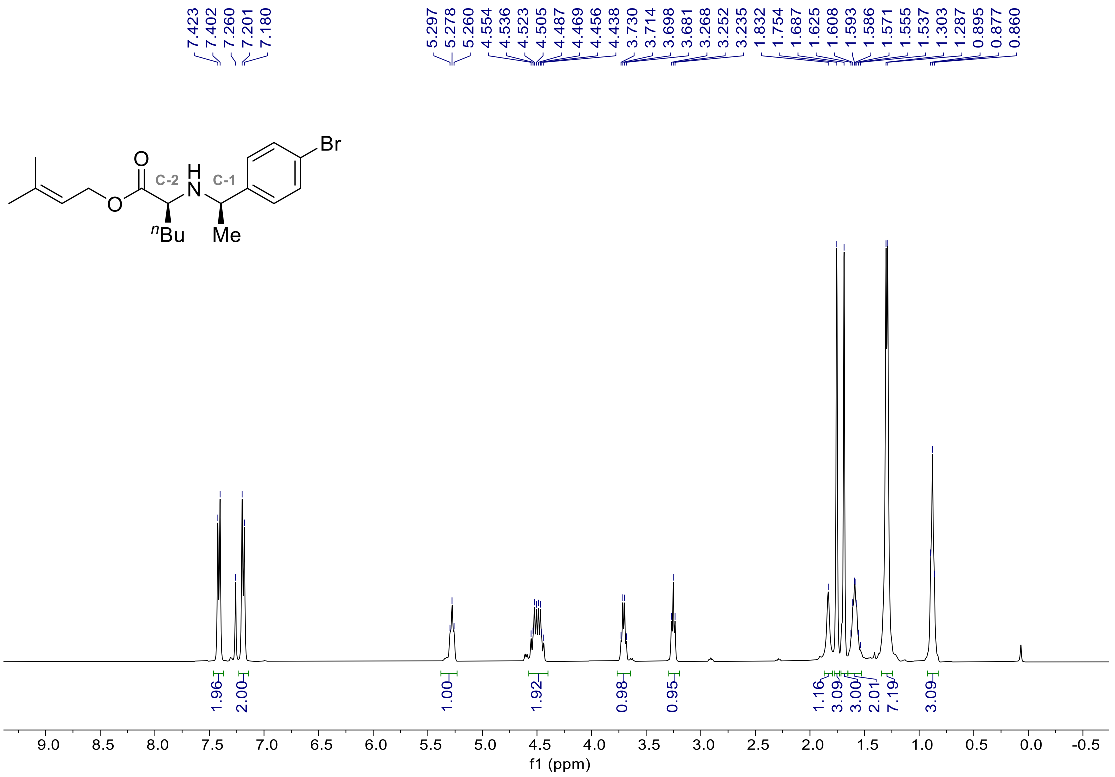

^{13}C NMR (101 MHz, CDCl$_3$) – (R_{C-1}, S_{C-2})-**35**

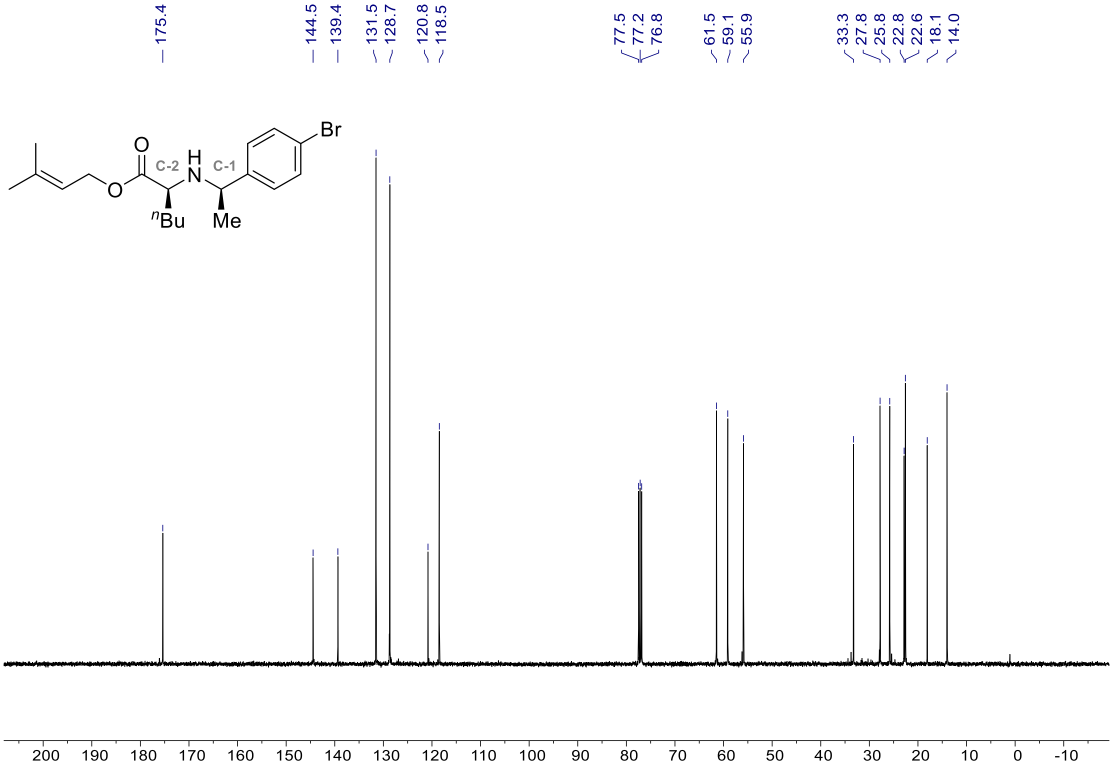

^{1}H NMR (400 MHz, $CDCl_3$) – ($R_{C\text{-}1}$, $S_{C\text{-}2}$)-**36**

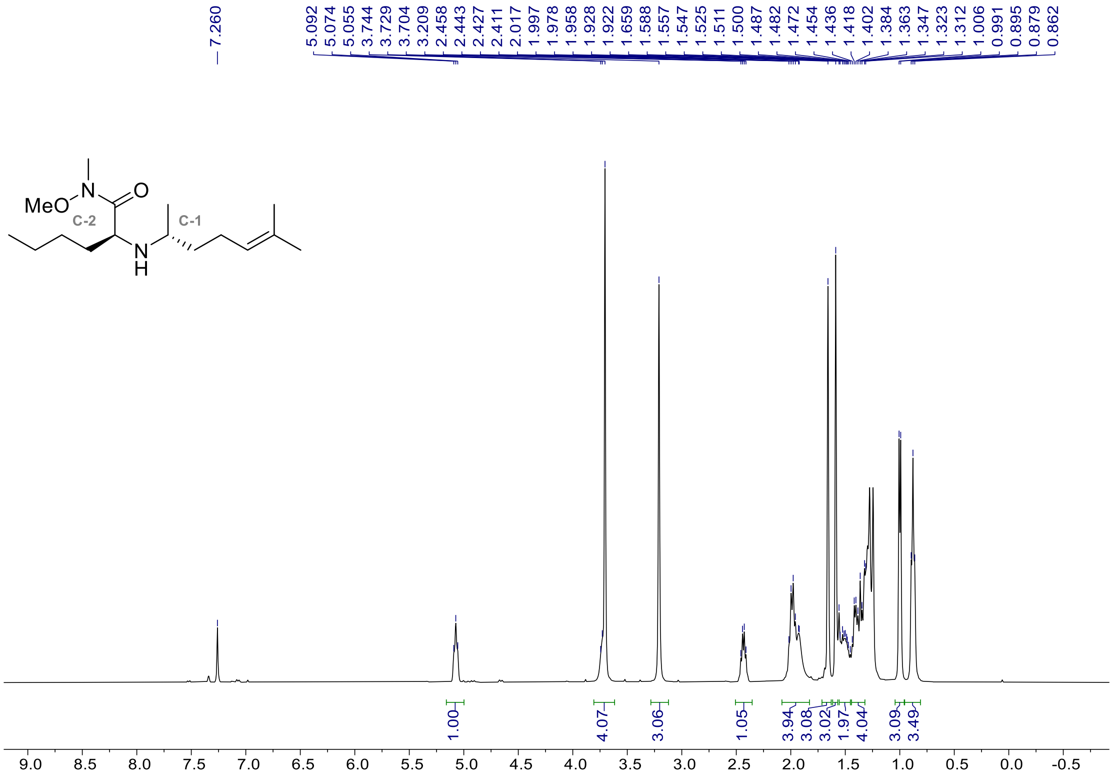

^{13}C NMR (101 MHz, $CDCl_3$) – ($R_{C\text{-}1}$, $S_{C\text{-}2}$)-**36**

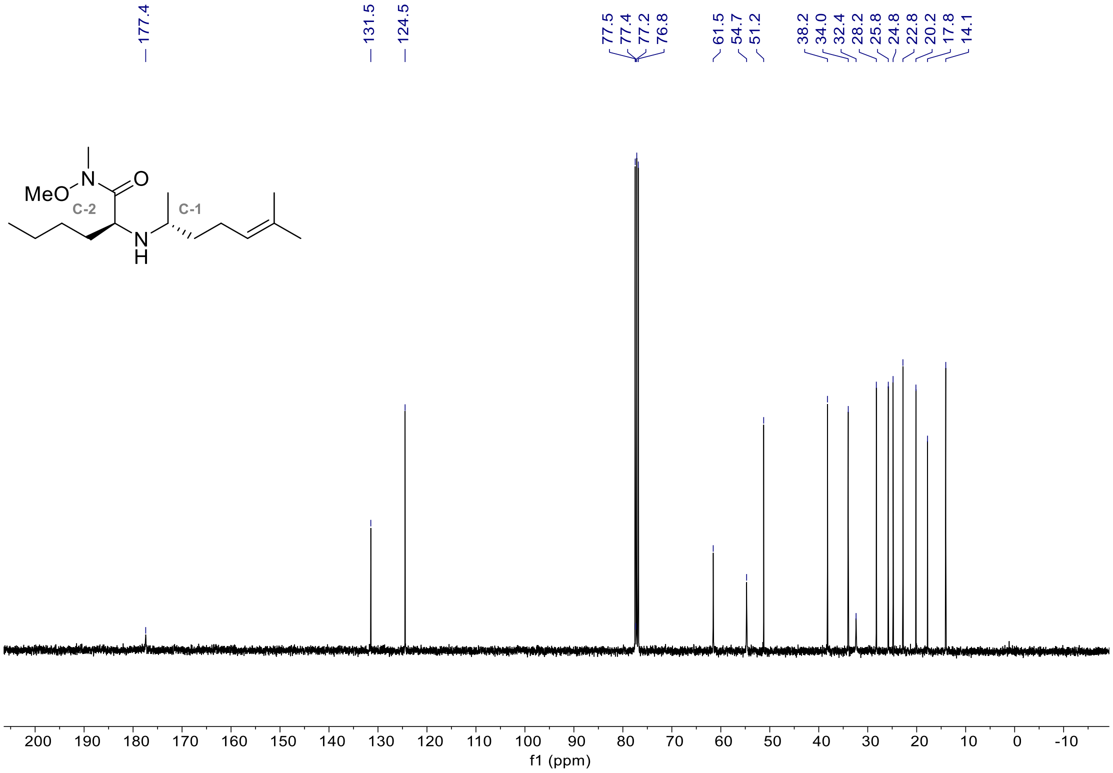

^{1}H NMR (400 MHz, $CDCl_3$) – ($R_{C\text{-}1}$, $R_{C\text{-}2}$)-**37**

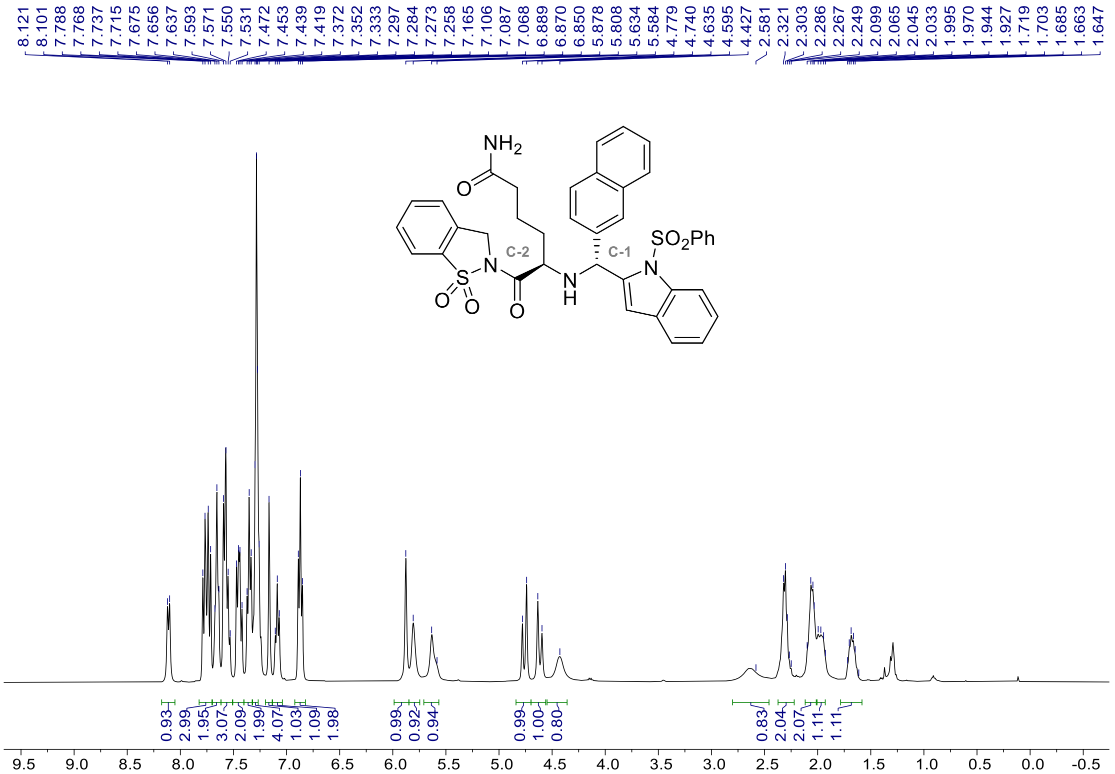

^{13}C NMR (101 MHz, $CDCl_3$) – ($R_{C\text{-}1}$, $R_{C\text{-}2}$)-**37**

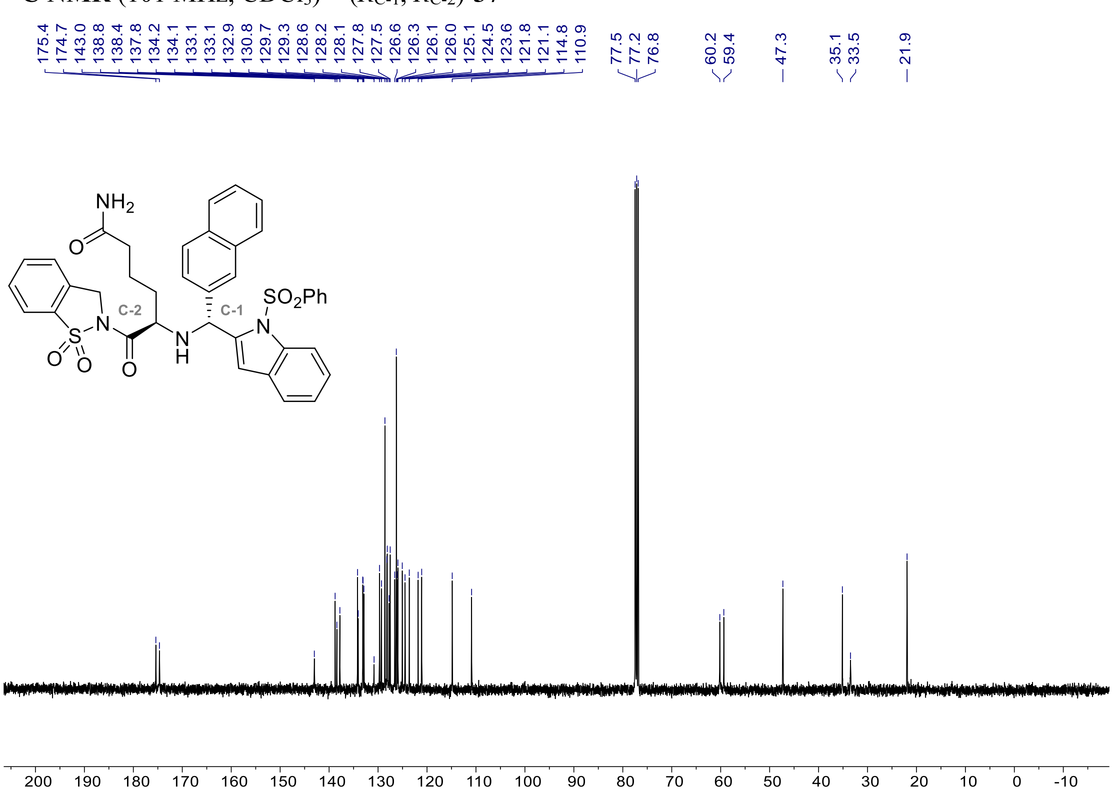

^{1}H NMR (400 MHz, $CDCl_3$) - ($R_{S(IV)}$, S_{C-1})-**S38a**

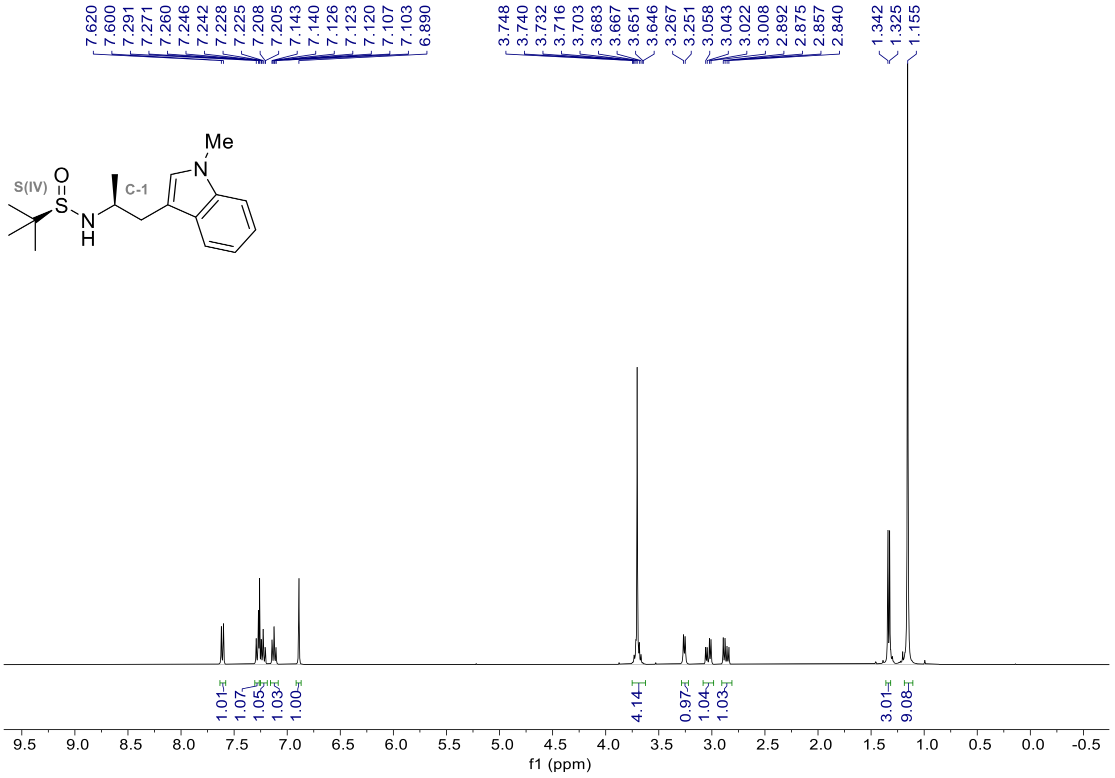

^{13}C NMR (101 MHz, $CDCl_3$) - ($R_{S(IV)}$, S_{C-1})-**S38a**

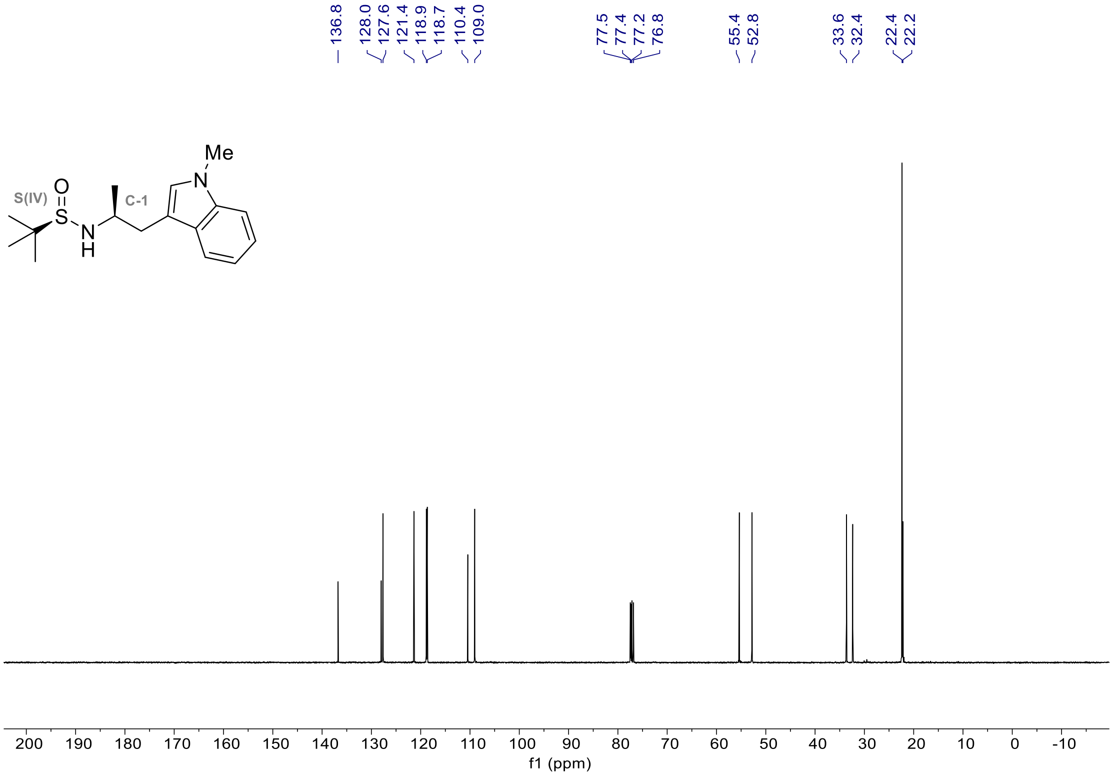

^{1}H NMR (400 MHz, CDCl$_3$) - ($S_{C\text{-}1}$, $S_{C\text{-}2}$)-**S38b**

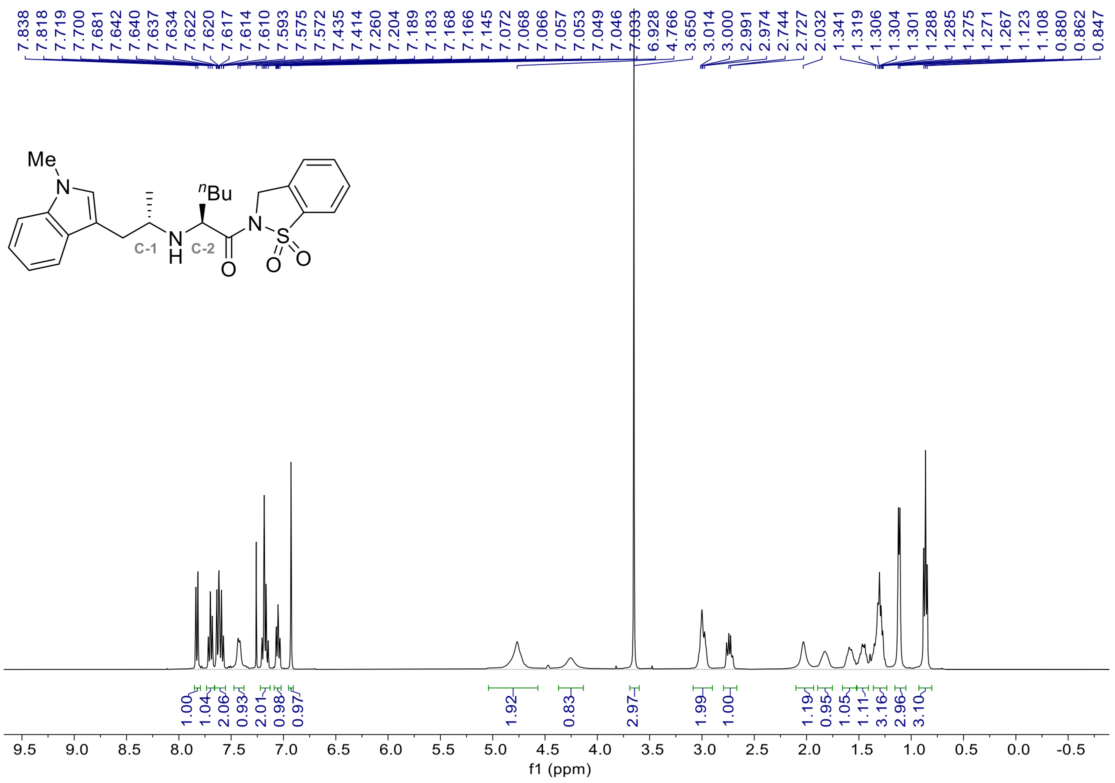

^{13}C NMR (101 MHz, CDCl$_3$) - ($S_{C\text{-}1}$, $S_{C\text{-}2}$)-**S38b**

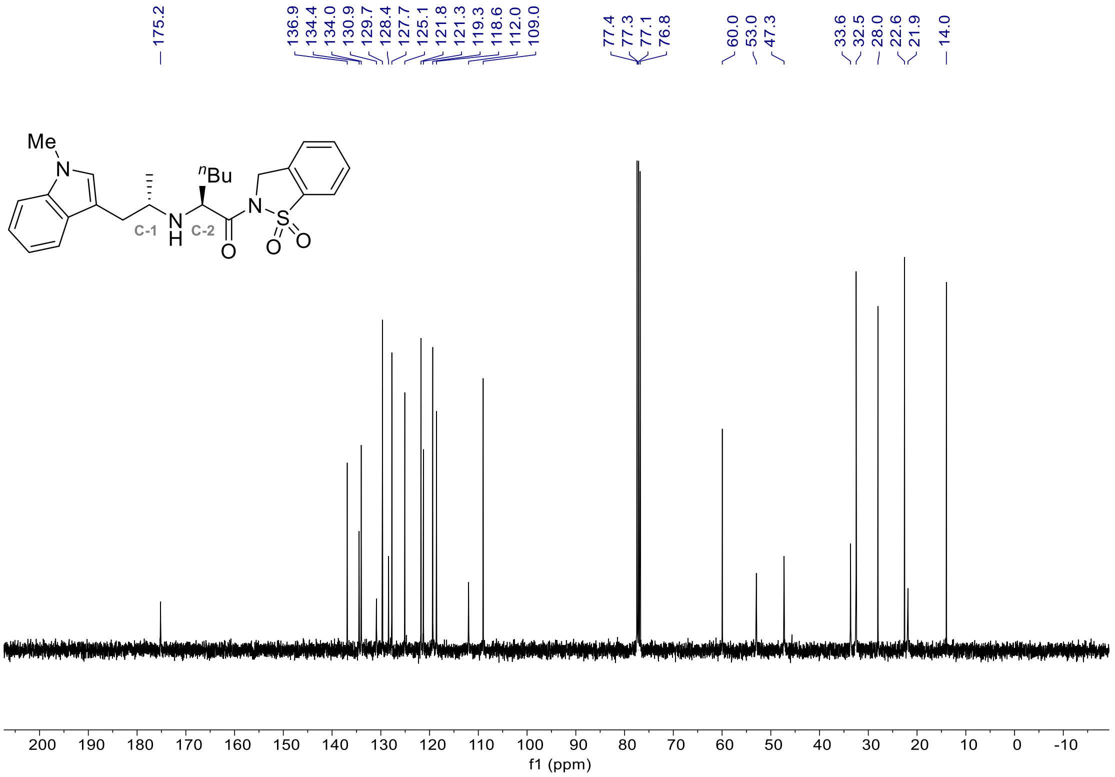

^{1}H NMR (400 MHz, $CDCl_3$) - ($S_{C\text{-}1}$, $S_{C\text{-}2}$, $S_{C\text{-}3}$)-**38**

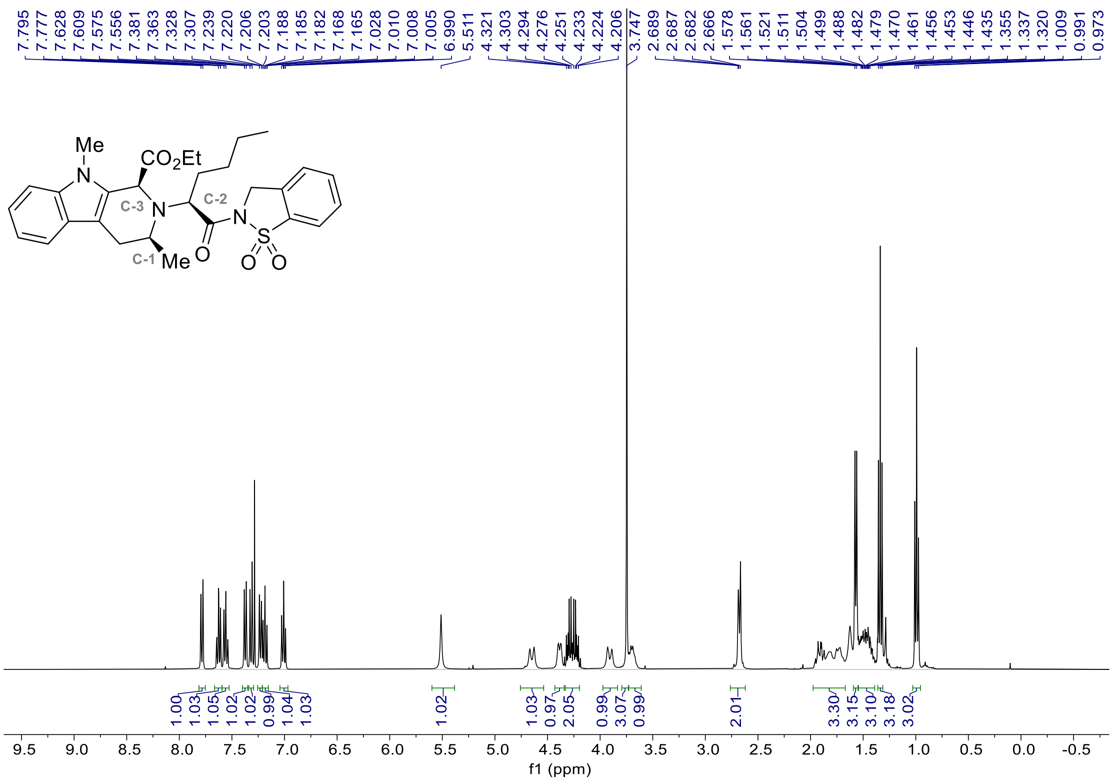

^{13}C NMR (101 MHz, $CDCl_3$) - ($S_{C\text{-}1}$, $S_{C\text{-}2}$, $S_{C\text{-}3}$)-**38**

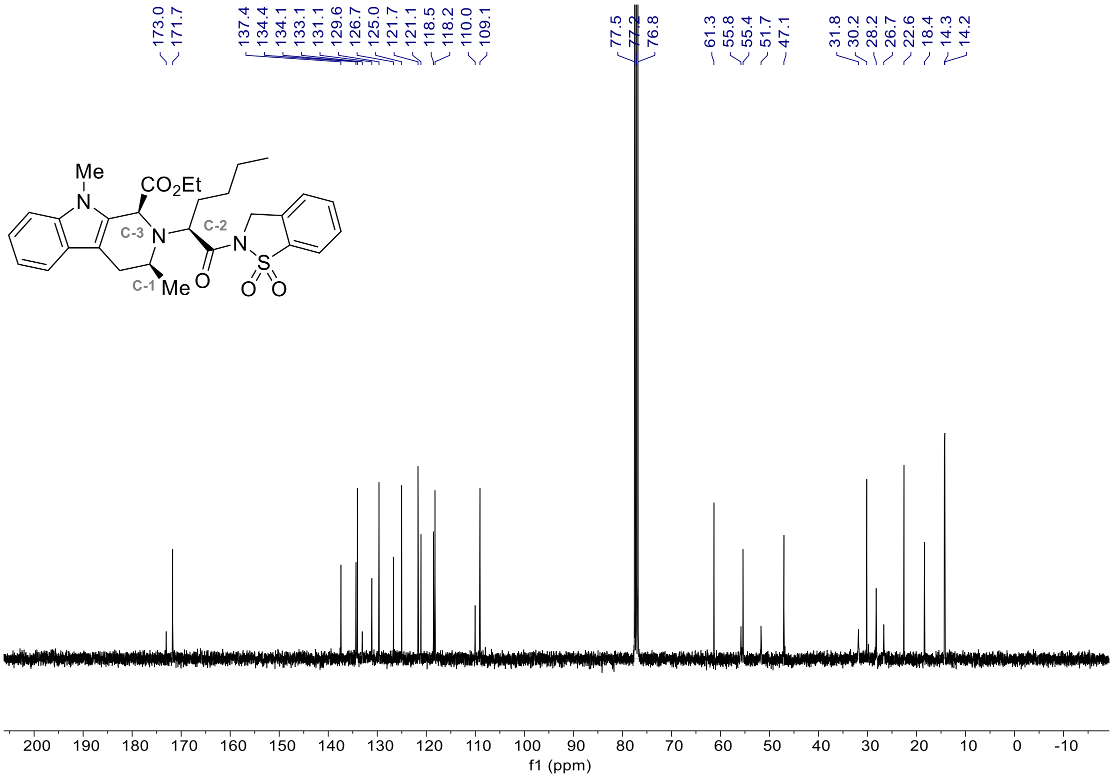

^{1}H NMR (400 MHz, $CDCl_3$) – (R_{C-1}, S_{C-2}, S_{C-3}, S_{C-4})-**39**

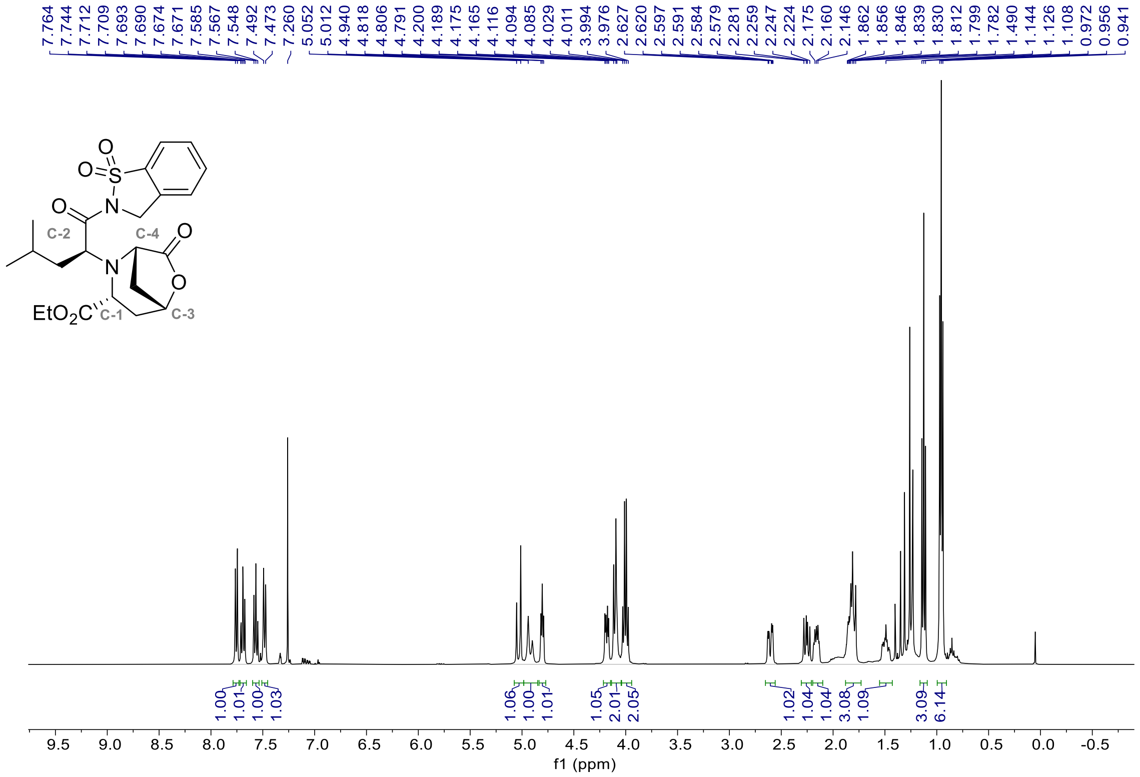

^{13}C NMR (101 MHz, $CDCl_3$) – (R_{C-1}, S_{C-2}, S_{C-3}, S_{C-4})-**39**

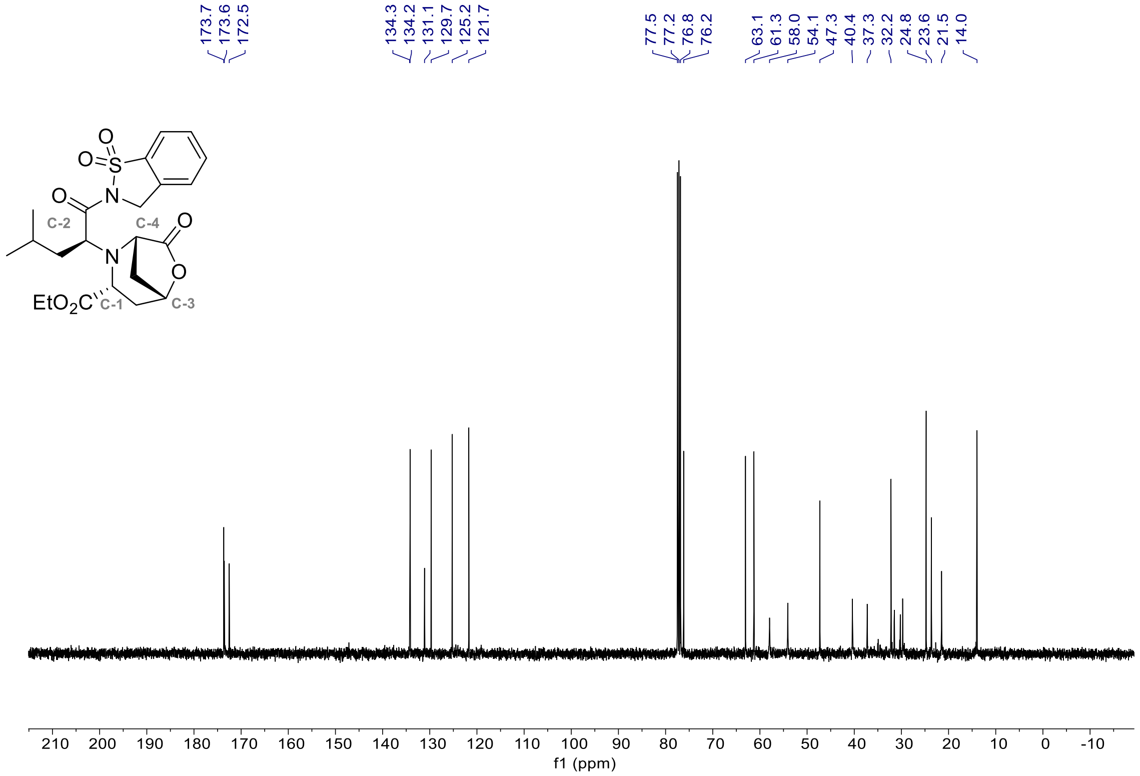

^{1}H NMR (400 MHz, $CDCl_3$) – ($R_{C\text{-}1}$, $S_{C\text{-}2}$)-**S40**

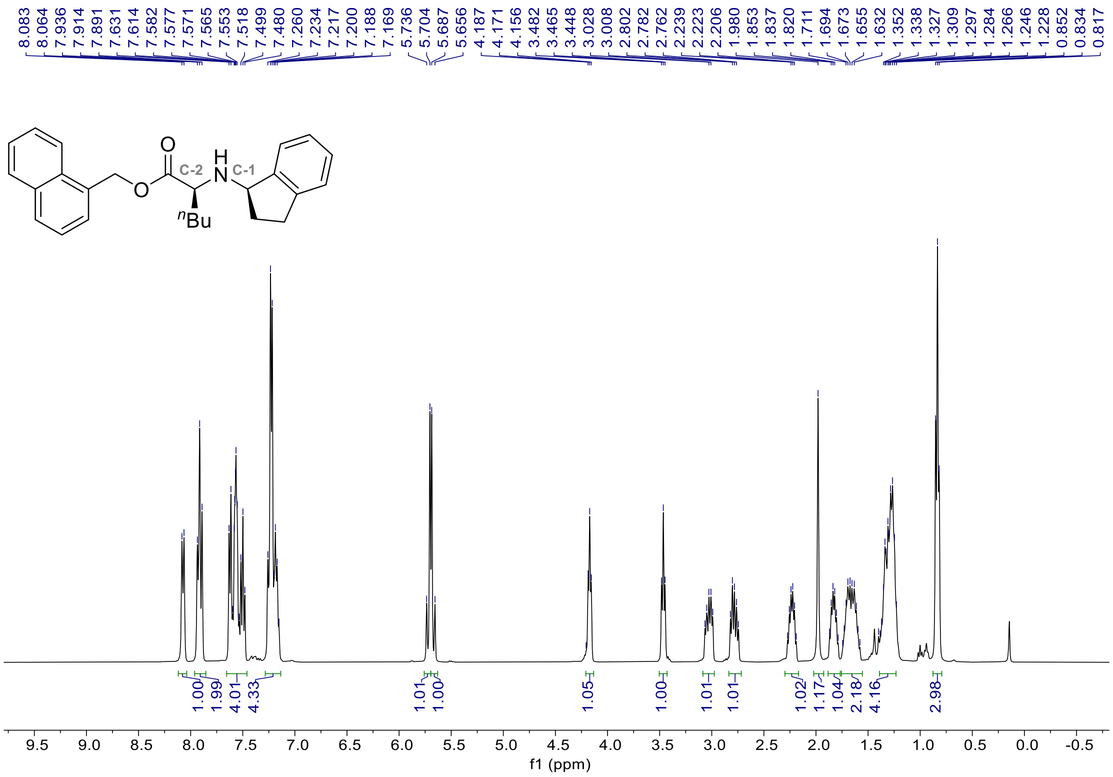

^{13}C NMR (101 MHz, $CDCl_3$) – ($R_{C\text{-}1}$, $S_{C\text{-}2}$)-**S40**

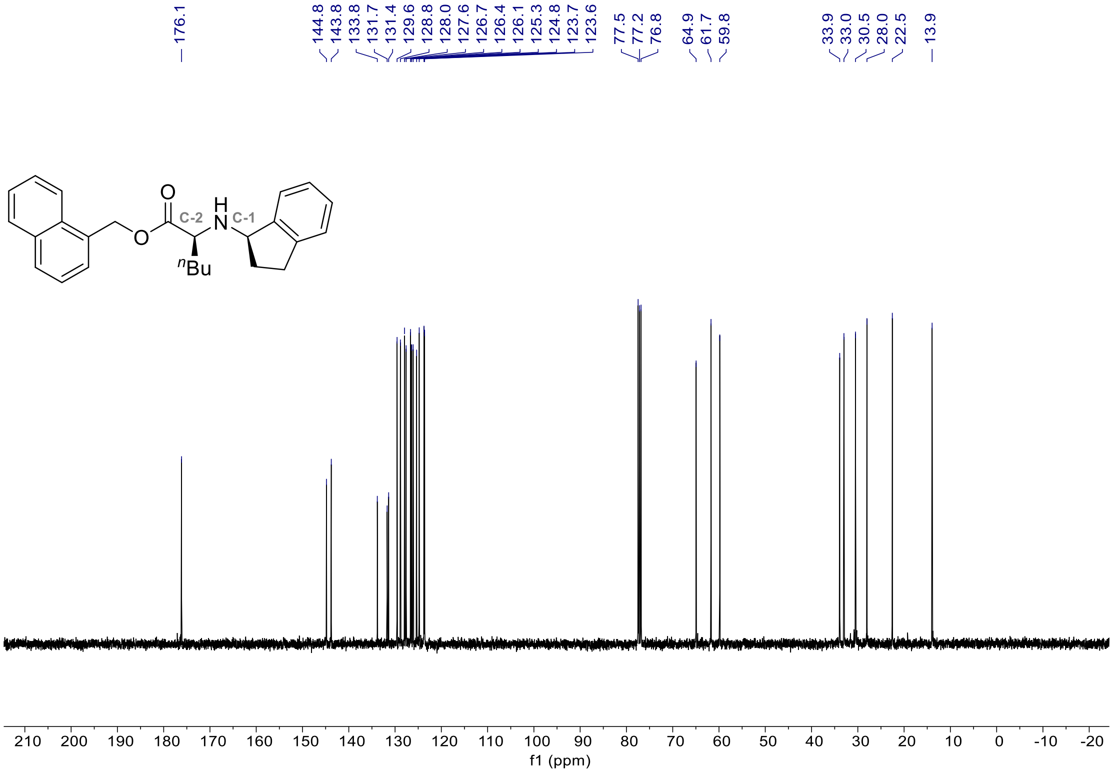

^{1}H NMR (400 MHz, $CDCl_3$) – (R_{C-1}, S_{C-2})-**40**

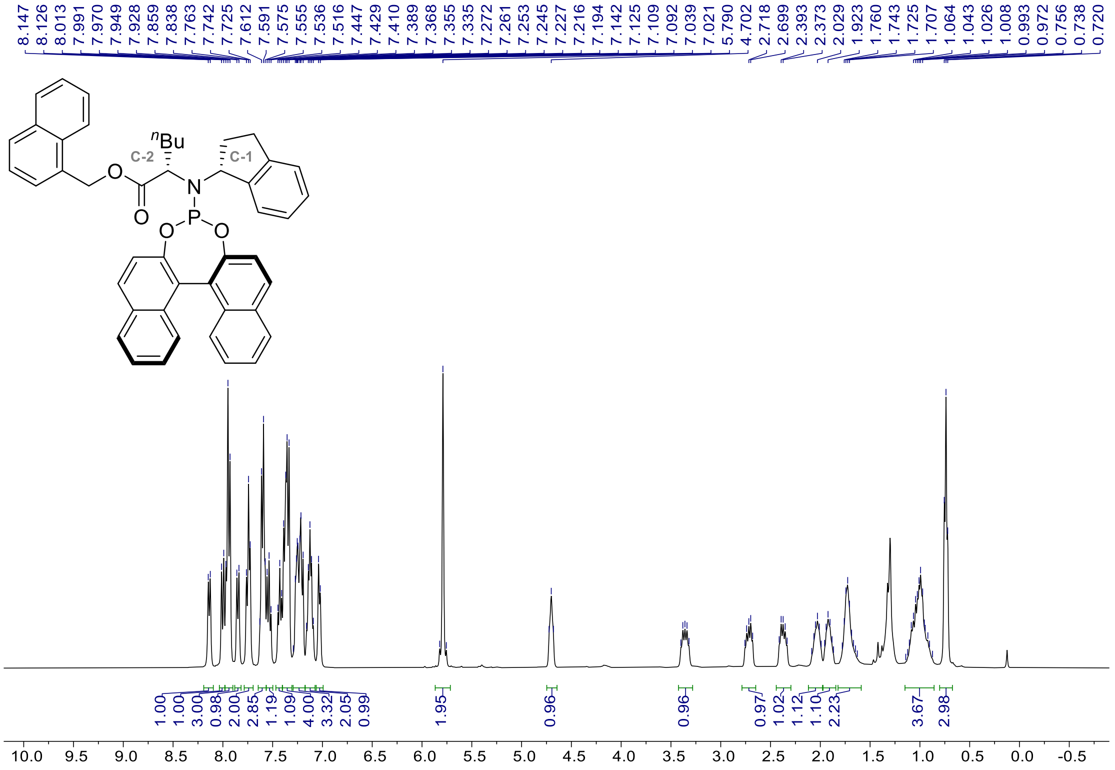

^{13}C NMR (101 MHz, $CDCl_3$) – (R_{C-1}, S_{C-2})-**40**

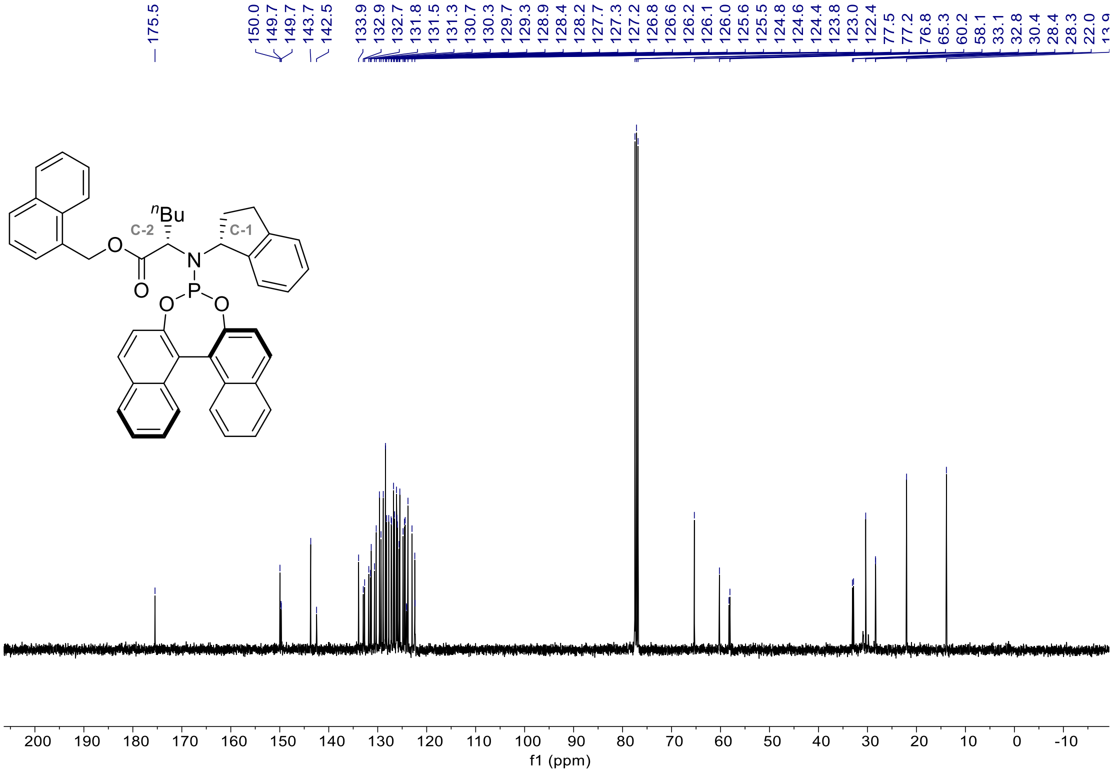

^{31}P NMR (162 MHz, $CDCl_3$) – ($R_{C\text{-}1}$, $S_{C\text{-}2}$)-**40**

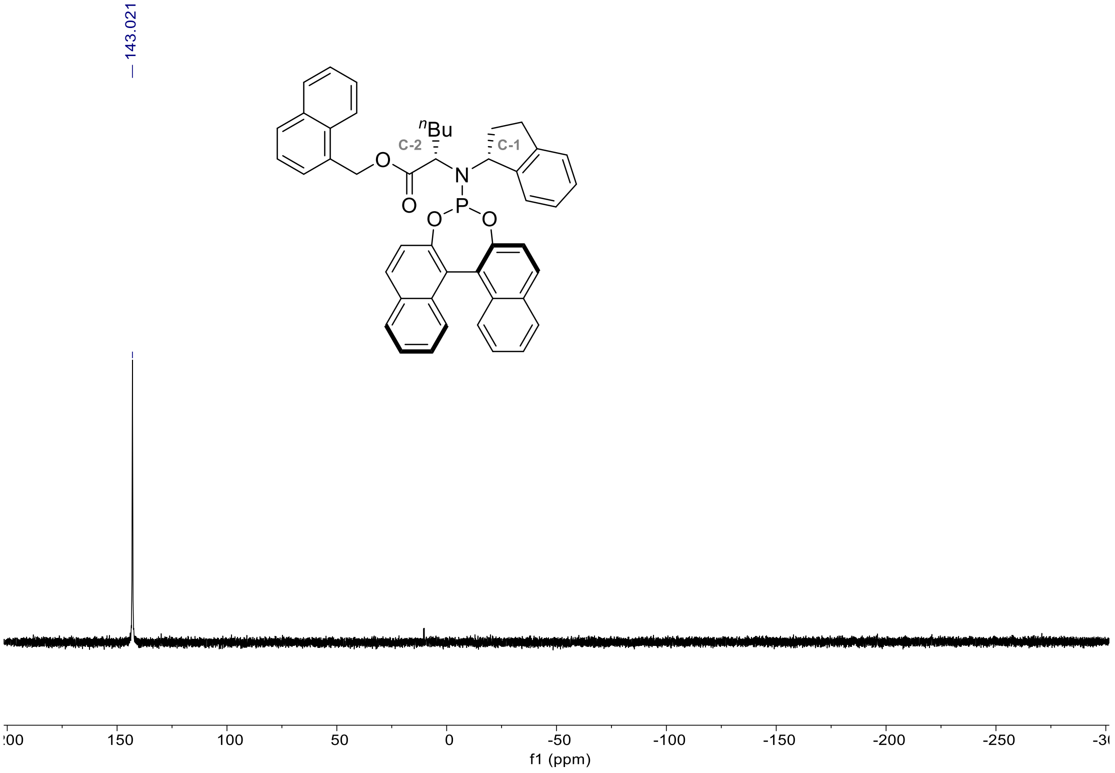

^{1}H NMR (400 MHz, $CDCl_3$) – ($R_{S(IV)}$, $S_{C\text{-}1}$)-**S41a**

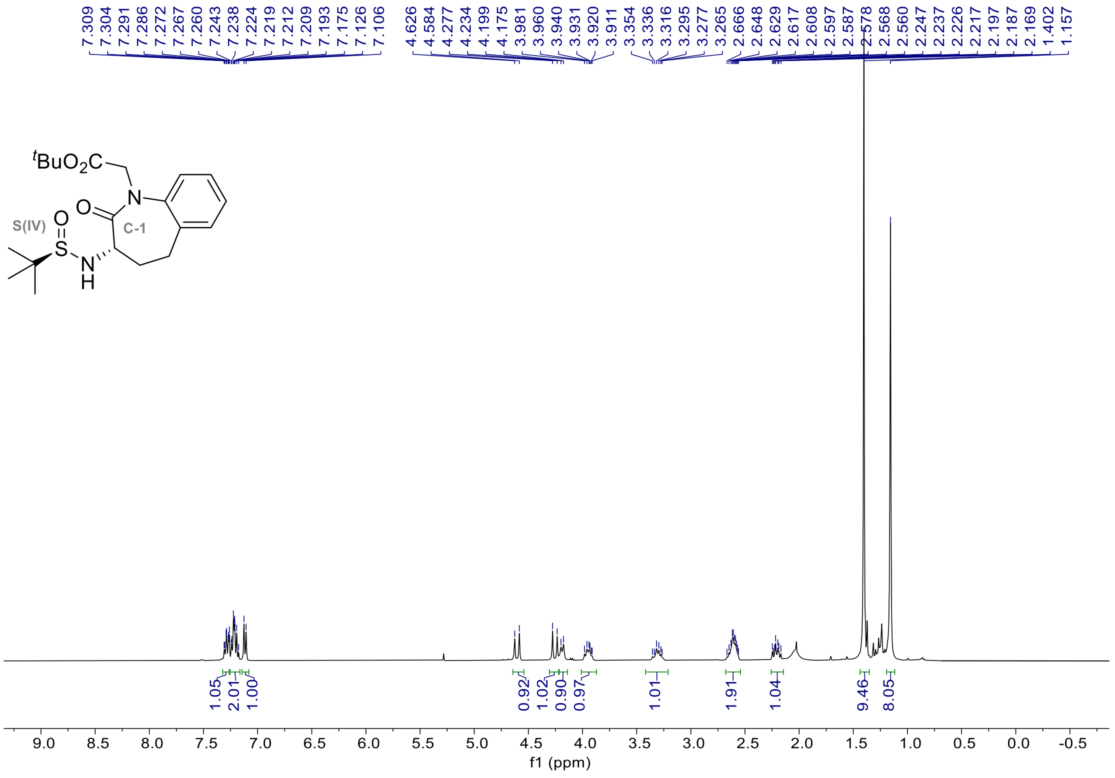

^{13}C NMR (101 MHz, $CDCl_3$) – ($R_{S(IV)}$, $S_{C\text{-}1}$)-**S41a**

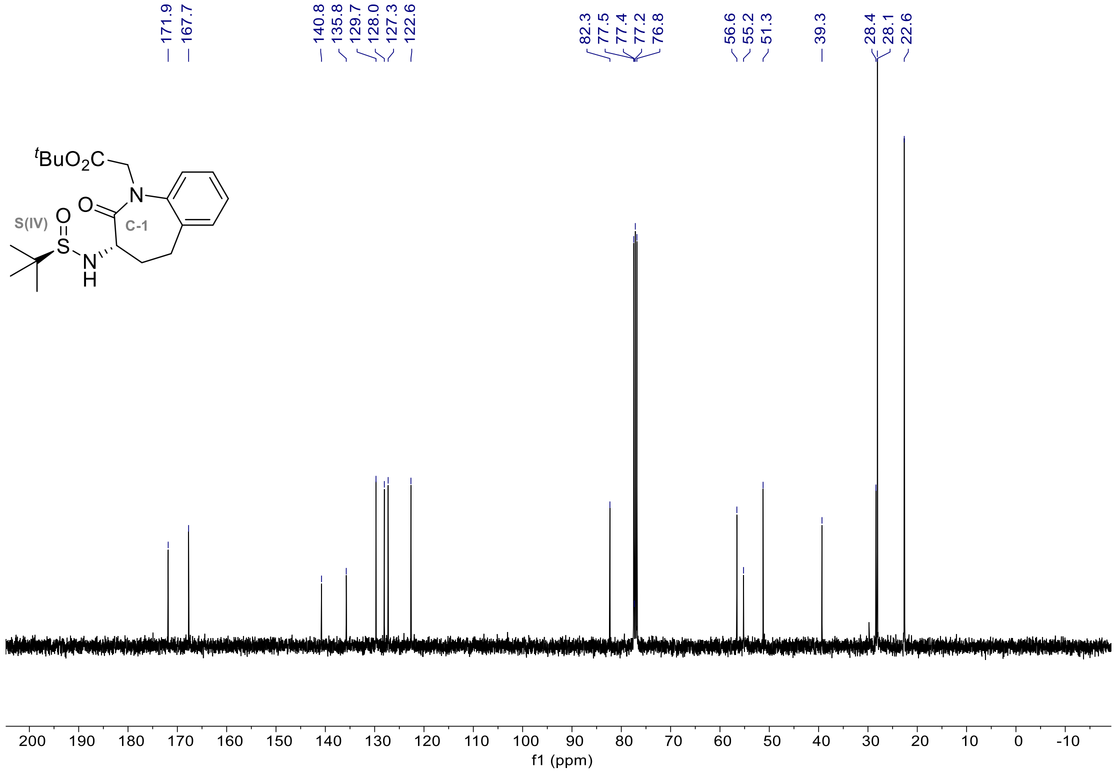

^{1}H NMR (400 MHz, $CDCl_3$) – ($S_{C\text{-}1}$, $S_{C\text{-}2}$)-**41a**

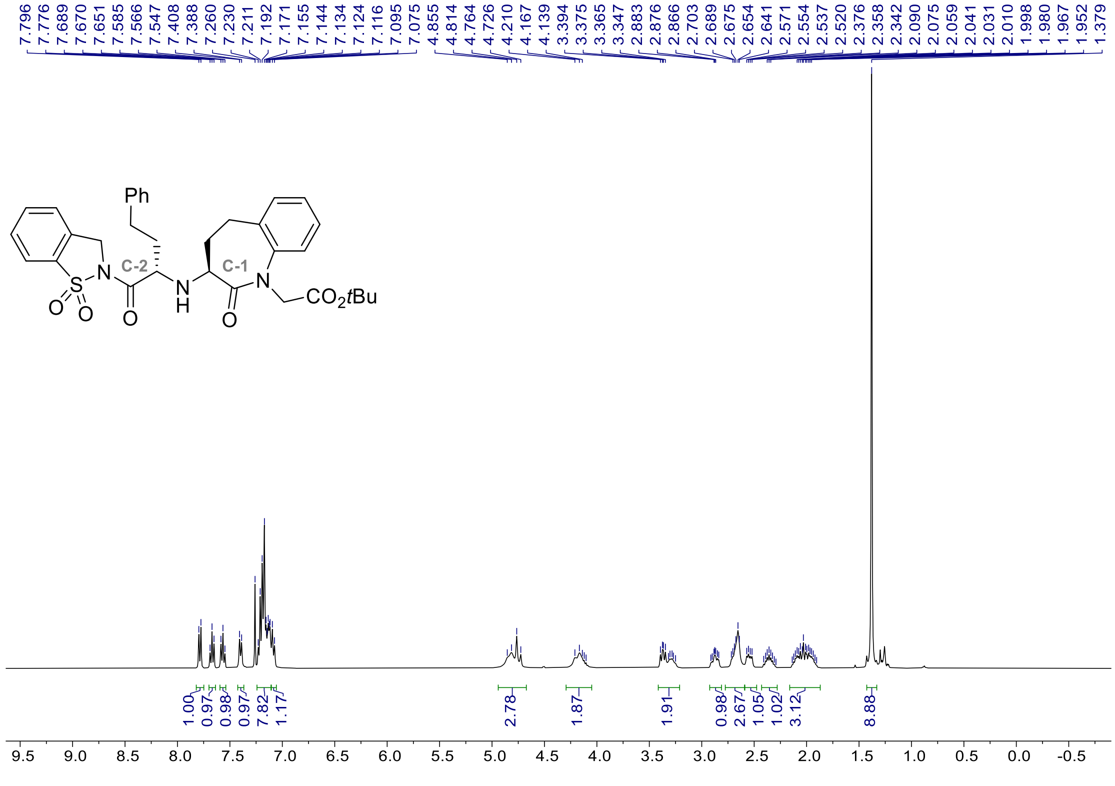

^{13}C NMR (101 MHz, $CDCl_3$) – ($S_{C\text{-}1}$, $S_{C\text{-}2}$)-**41a**

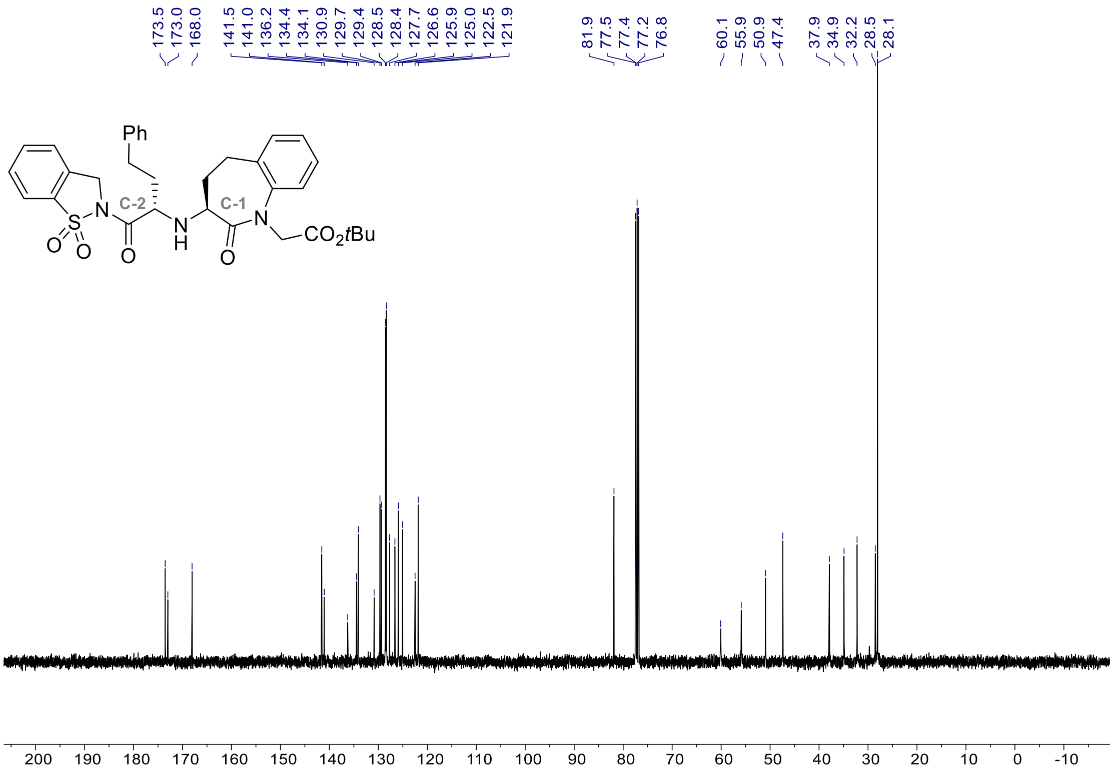

^{1}H NMR (400 MHz, $CDCl_3$) – ($S_{C\text{-}1}$, $S_{C\text{-}2}$)-**42a**

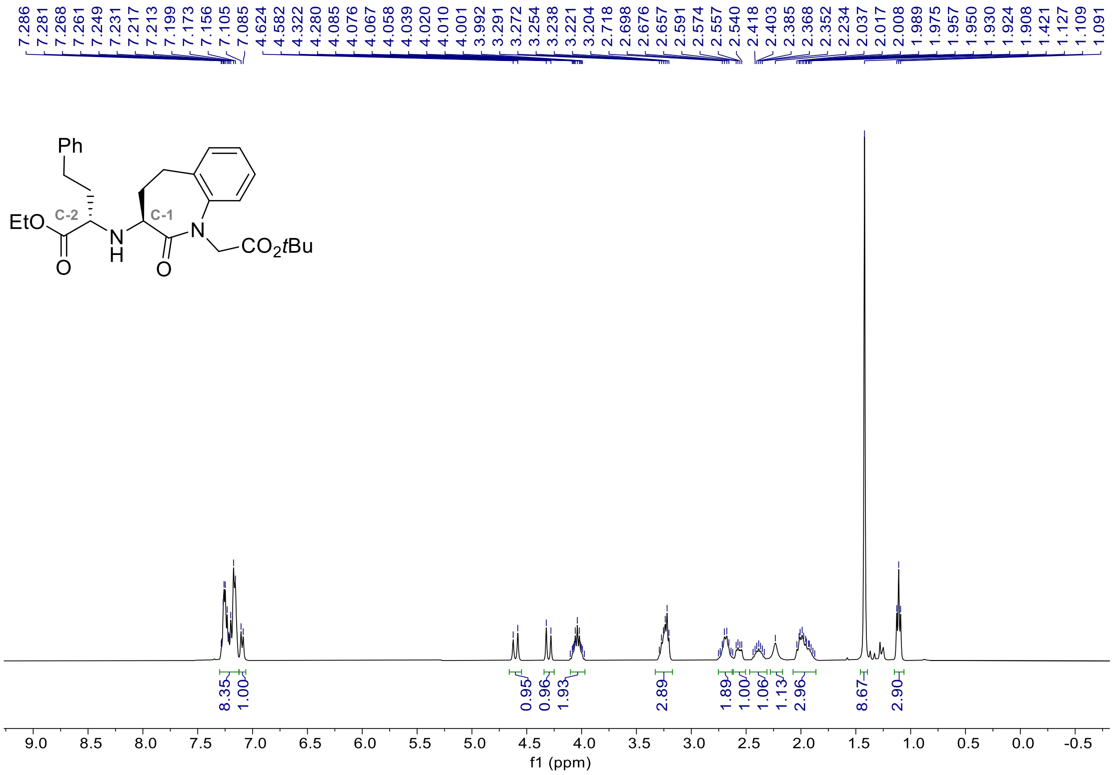

^{13}C NMR (101 MHz, CDCl$_3$) – ($S_{C\text{-}1}$, $S_{C\text{-}2}$)-**42a**

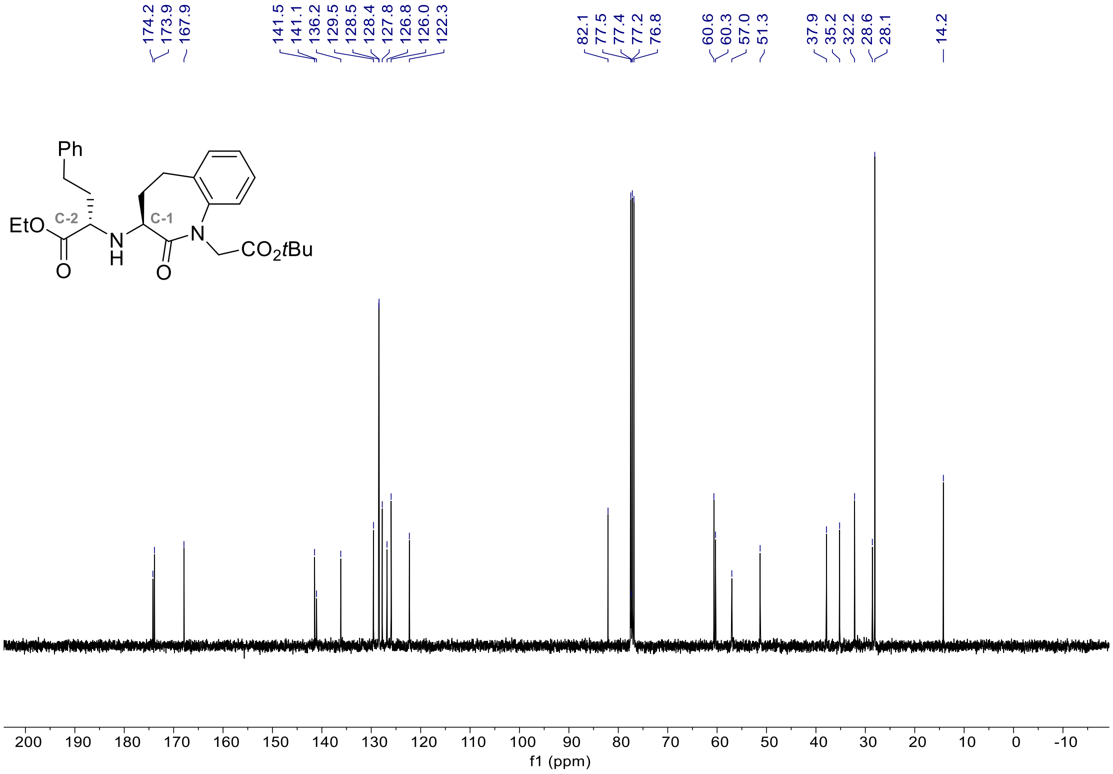

^{1}H NMR (400 MHz, CDCl$_3$) – ($S_{S(IV)}$, $S_{C\text{-}1}$)-**S41b**

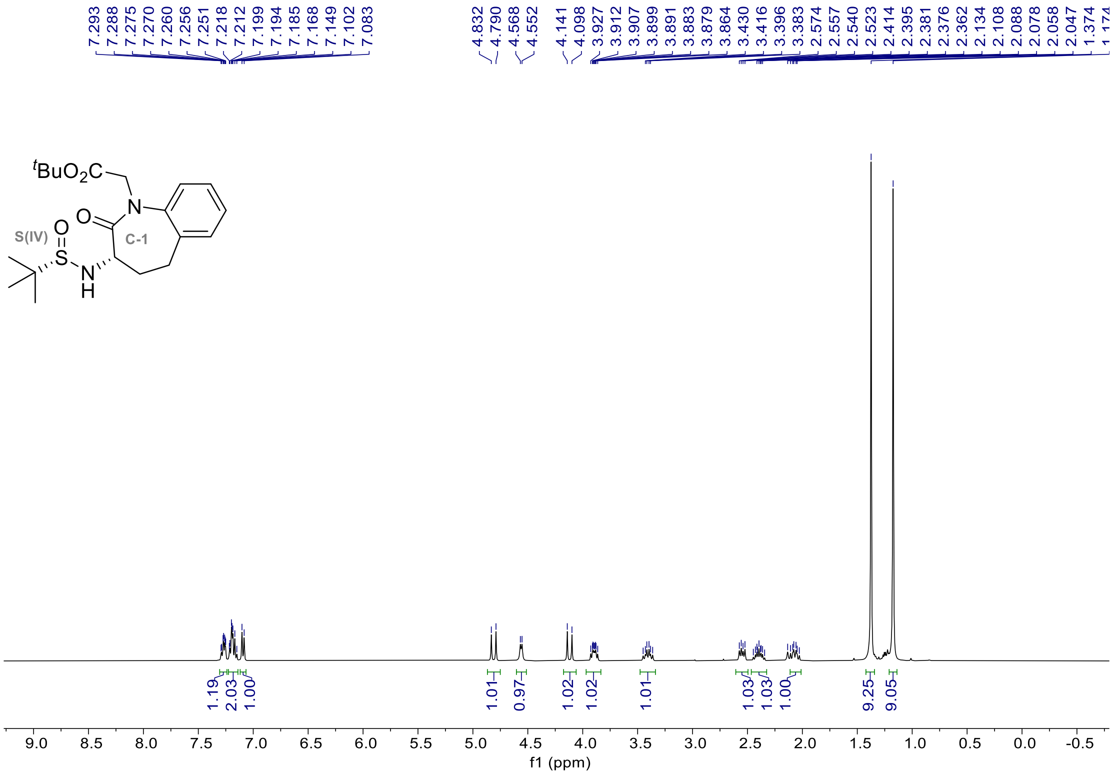

^{13}C NMR (400 MHz, $CDCl_3$) – ($S_{S(IV)}$, $S_{C\text{-}1}$)-**S41b**

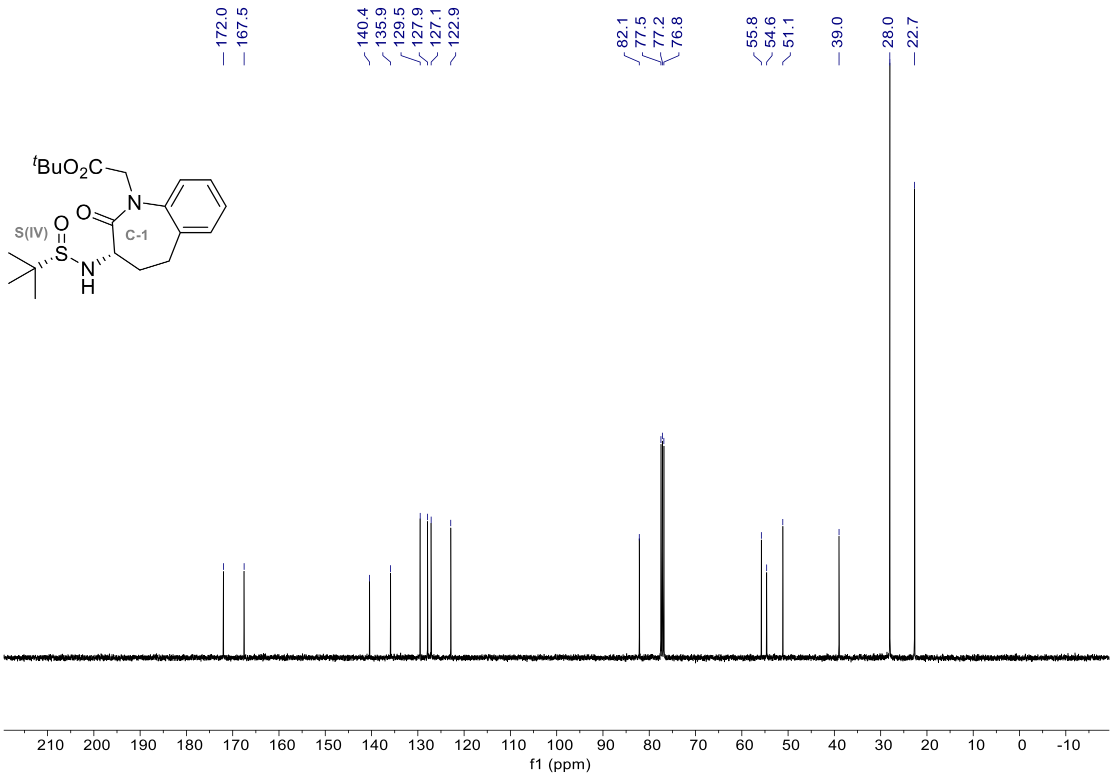

^{1}H NMR (400 MHz, $CDCl_3$) – ($S_{C\text{-}1}$, $R_{C\text{-}2}$)-**41b**

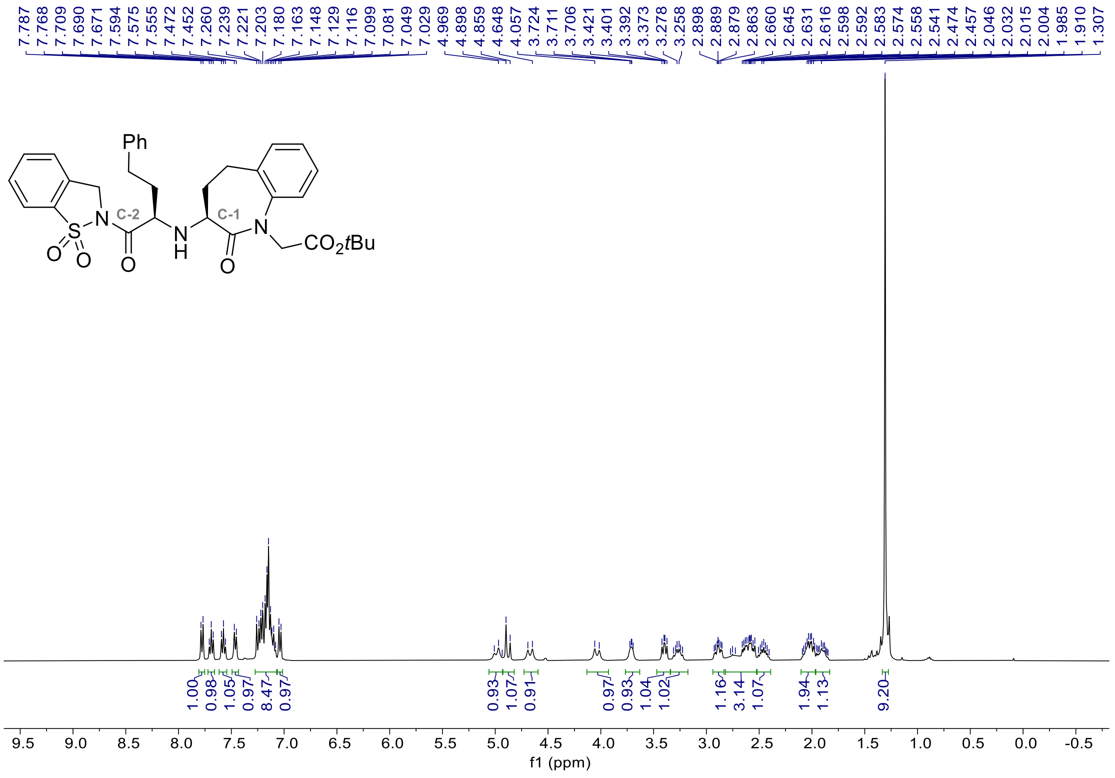

^{13}C NMR (101 MHz, $CDCl_3$) – ($S_{C\text{-}1}$, $R_{C\text{-}2}$)-**41b**

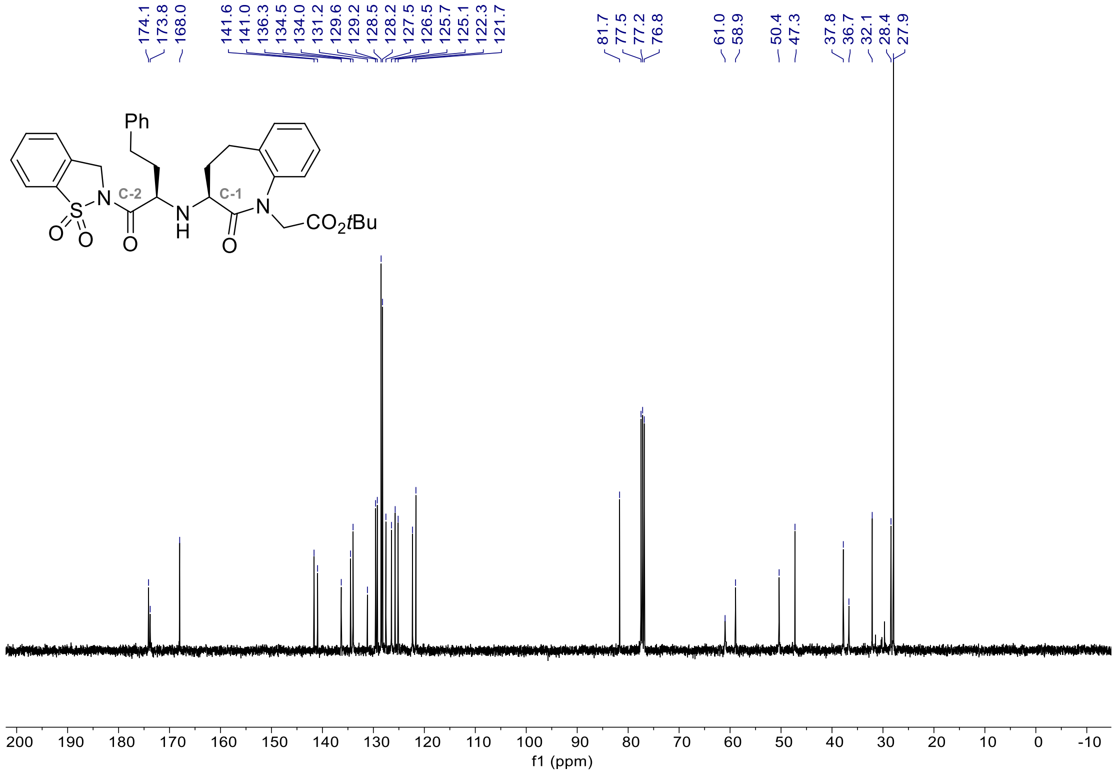

^{1}H NMR (400 MHz, $CDCl_3$) – ($S_{C\text{-}1}$, $R_{C\text{-}2}$)-**42b**

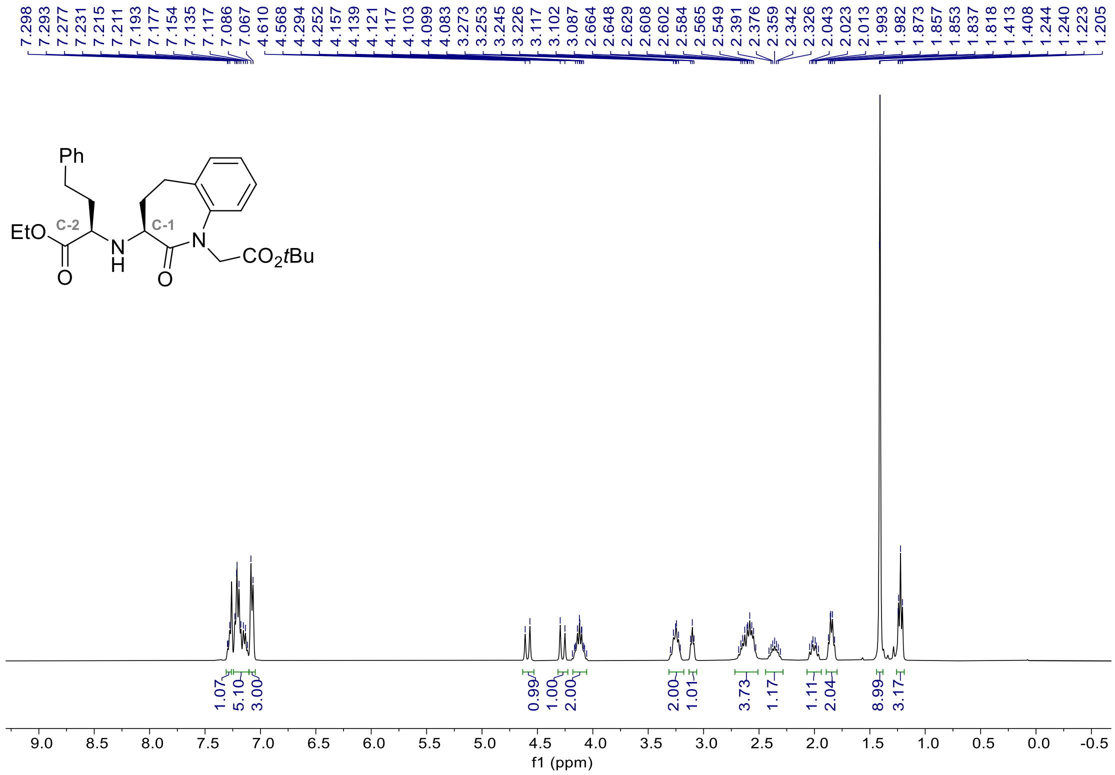

^{13}C NMR (101 MHz, $CDCl_3$) – ($S_{C\text{-}1}$, $R_{C\text{-}2}$)-**42b**

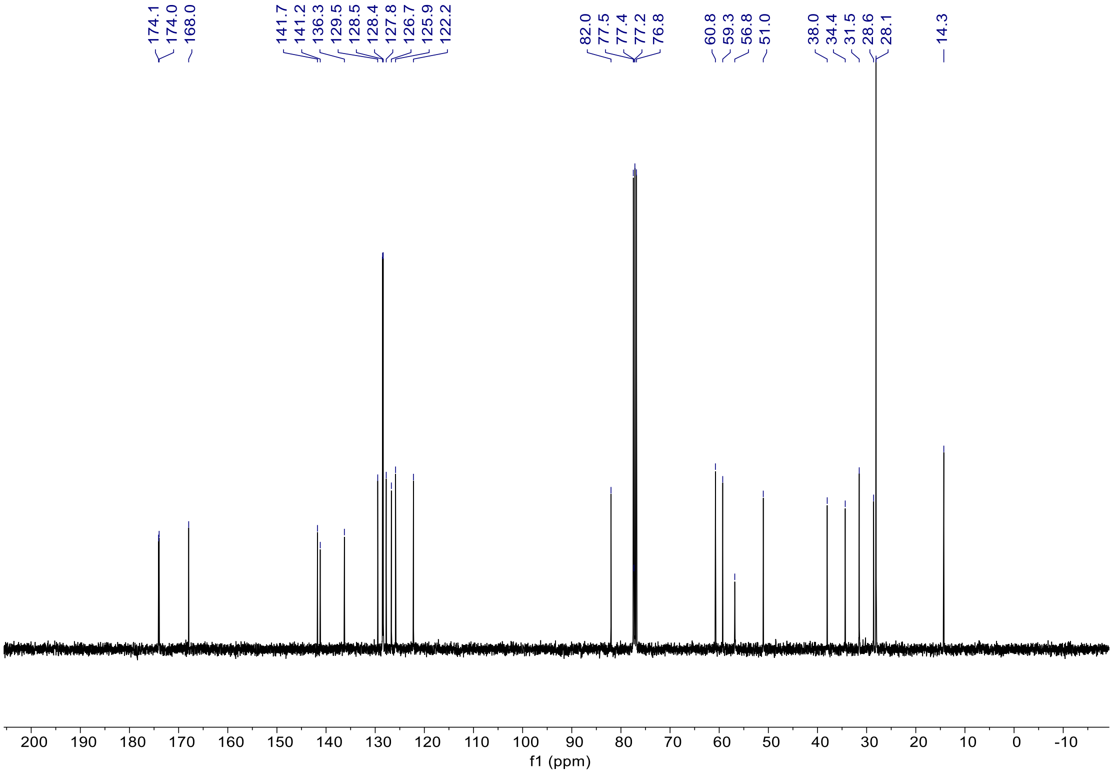

^{1}H NMR (400 MHz, $CDCl_3$) – ($R_{S(IV)}$, $S_{C\text{-}1}$, $S_{C\text{-}3}$)-**S43**

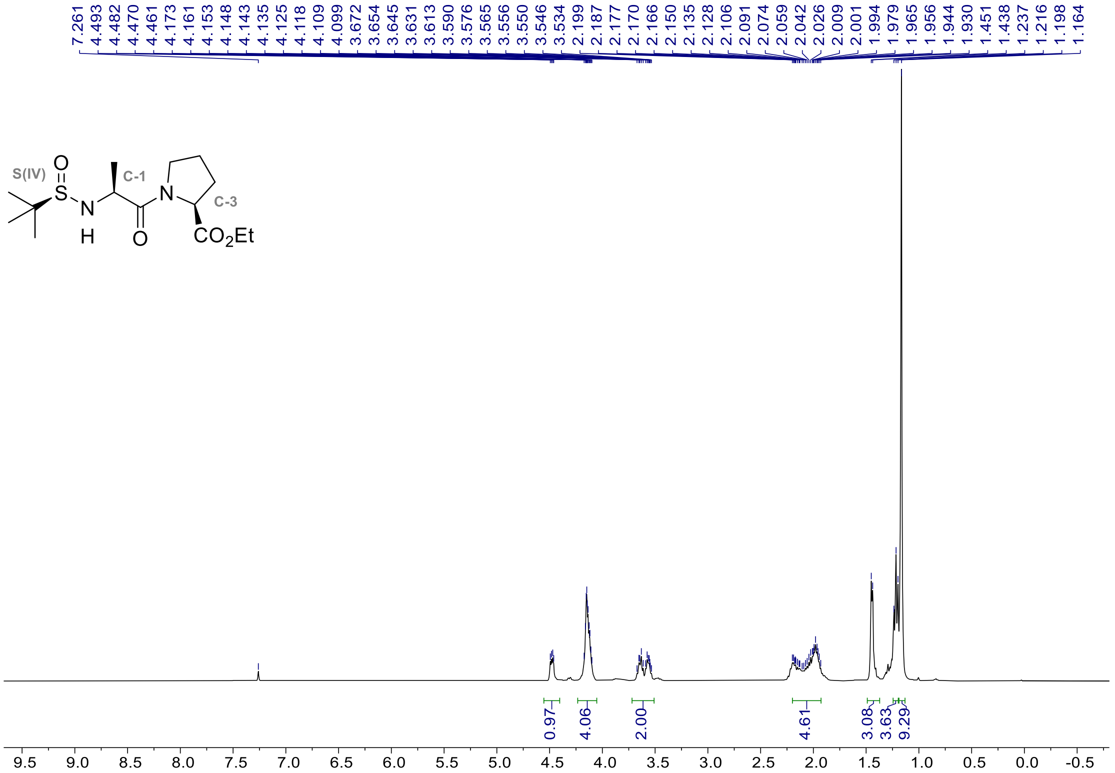

^{13}C NMR (101 MHz, CDCl$_3$) – ($R_{S(IV)}$, S_{C-1}, S_{C-3})-**S43**

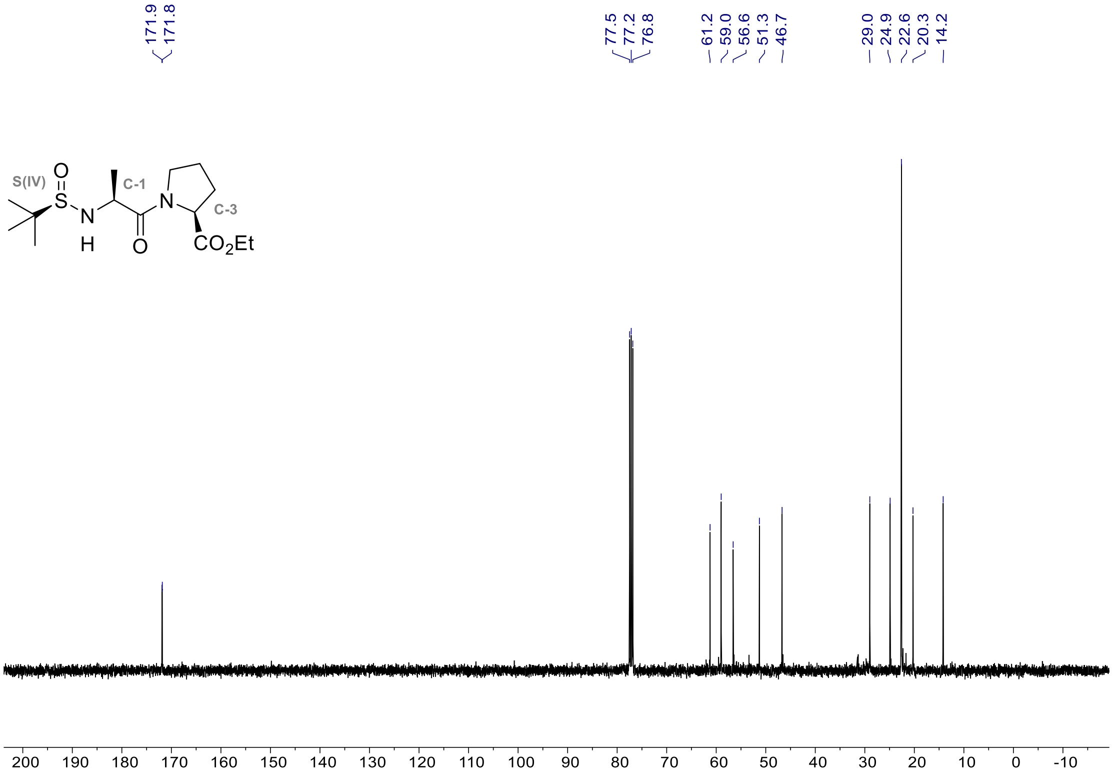

^{1}H NMR (400 MHz, CDCl$_3$) – (S_{C-1}, S_{C-2}, S_{C-3})-**43**

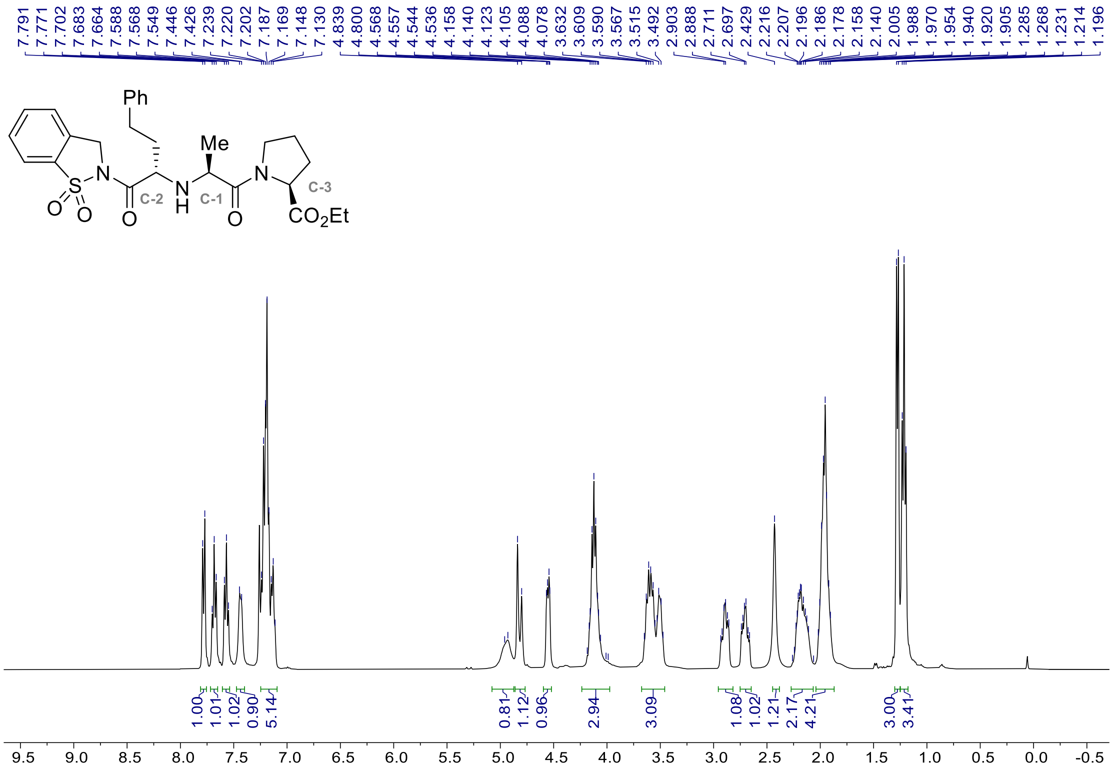

^{13}C NMR (101 MHz, $CDCl_3$) – (S_{C-1}, S_{C-2}, S_{C-3})-**43**

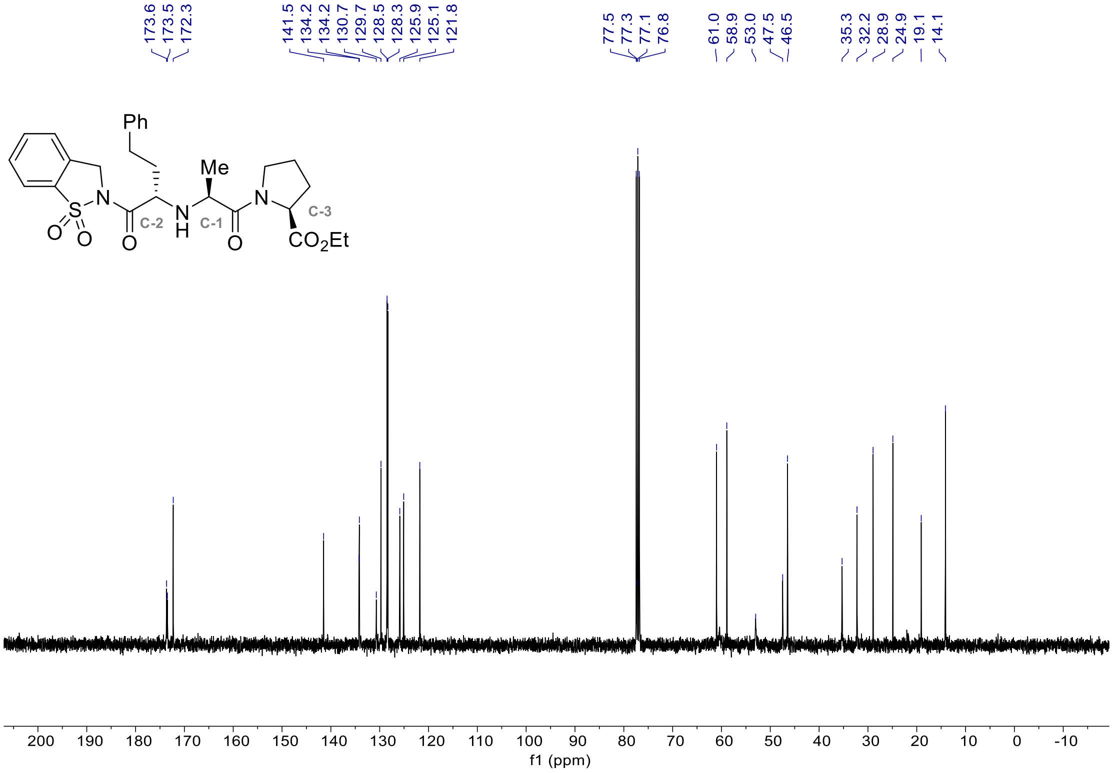

^{1}H NMR (400 MHz, $CDCl_3$) - (S_{C-1}, S_{C-2}, S_{C-3})-**44**

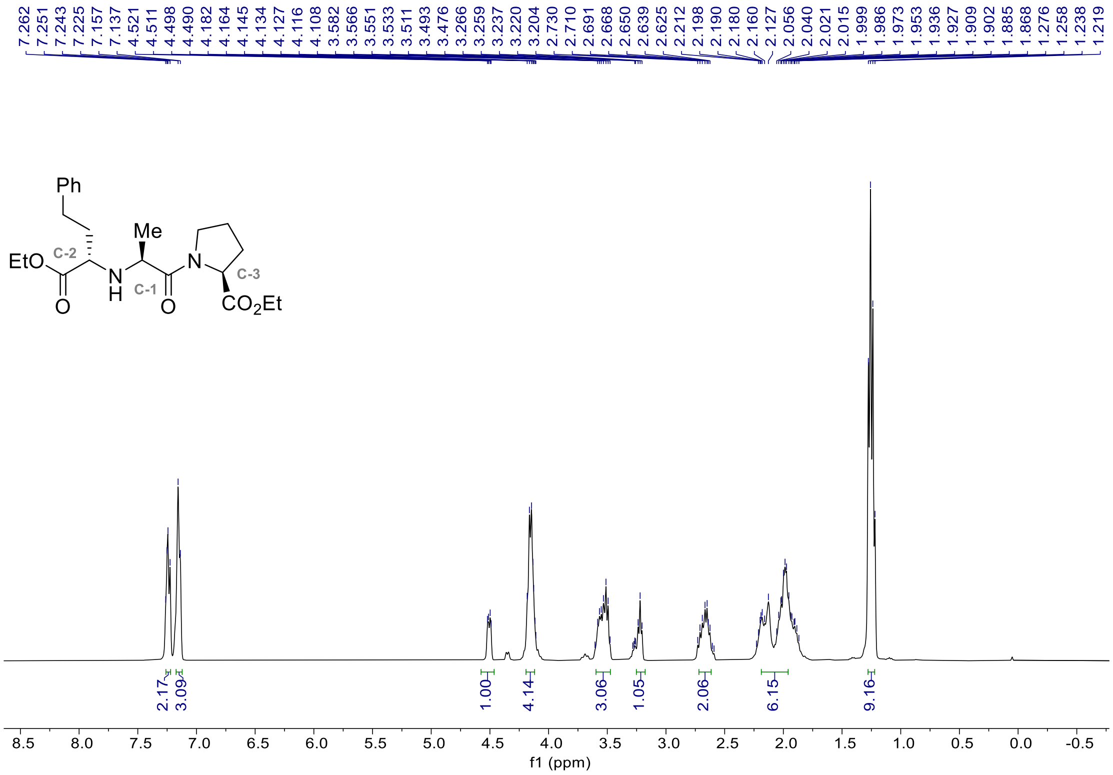

^{13}C NMR (101 MHz, $CDCl_3$) - ($S_{C\text{-}1}$, $S_{C\text{-}2}$, $S_{C\text{-}3}$)-**44**

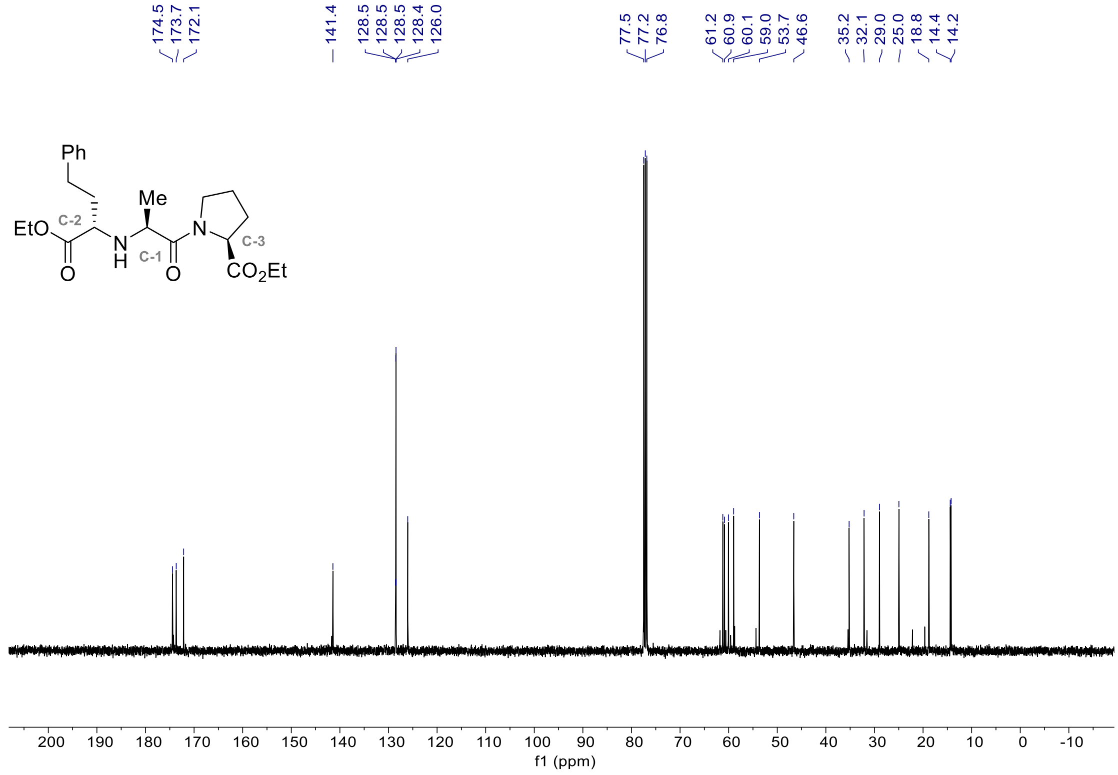

REFERENCES AND NOTES

1. Y. Wang, I. Haight, R. Gupta, A. Vasudevan, What is in our kit? An analysis of building blocks used in medicinal chemistry parallel libraries. *J. Med. Chem.* **64**, 17115–17122 (2021).

2. N. A. McGrath, M. Brichacek, J. T. Njardarson, A graphical journey of innovative organic architectures that have improved our lives. *J. Chem. Educ.* **87**, 1348–1349 (2010).

3. T. C. Nugent, Ed., *Chiral Amine Synthesis*: *Methods, Developments and Applications* (Wiley, 2010).

4. A. Cabré, X. Verdaguer, A. Riera, Recent advances in the enantioselective synthesis of chiral amines via transition metal-catalyzed asymmetric hydrogenation. *Chem. Rev.* **122**, 269–339 (2022).

5. T. W. Thorpe, J. R. Marshall, V. Harawa, R. E. Ruscoe, A. Cuetos, J. D. Finnigan, A. Angelastro, R. S. Heath, F. Parmeggiani, S. J. Charnock, R. M. Howard, R. Kumar, D. S. B. Daniels, G. Grogan, N. J. Turner, Multifunctional biocatalyst for conjugate reduction and reductive amination. *Nature* **604**, 86–91 (2022).

6. M. Wang, S. Liu, H. Liu, Y. Wang, Y. Lan, Q. Liu, Asymmetric hydrogenation of ketimines with minimally different alkyl groups. *Nature* **631**, 556–562 (2024).

7. B. Li, J. Chen, D. Liu, I. D. Gridnev, W. Zhang, Nickel-catalysed asymmetric hydrogenation of oximes. *Nat. Chem.* **14**, 920–927 (2022).

8. Q. Yin, Y. Shi, J. Wang, X. Zhang, Direct catalytic asymmetric synthesis of α-chiral primary amines. *Chem. Soc. Rev.* **49**, 6141–6153 (2020).

9. G. Li, Y. Kanda, S. Y. Hong, A. T. Radosevich, Enabling reductive C–N cross-coupling of nitroalkanes and boronic acids by steric design of P(III)/P(V)═O catalysts. *J. Am. Chem. Soc.* **144**, 8242–8248 (2022).

10. S. Kobayashi, Y. Mori, J. S. Fossey, M. M. Salter, Catalytic enantioselective formation of C-C bonds by addition to imines and hydrazones: A ten-year update. *Chem. Rev.* **111**, 2626–2704 (2011).

11. Y. Wu, L. Hu, Z. Li, L. Deng, Catalytic asymmetric umpolung reactions of imines. *Nature* **523**, 445–450 (2015).

12. Y. Xi, S. Ma, J. F. Hartwig, Catalytic asymmetric addition of an amine N-H bond across internal alkenes. *Nature* **588**, 254–260 (2020).

13. S.-L. Shi, Z. L. Wong, S. L. Buchwald, Copper-catalysed enantioselective stereodivergent synthesis of amino alcohols. *Nature* **532**, 353–356 (2016).

14. M.-L. Li, J.-H. Yu, Y.-H. Li, S.-F. Zhu, Q.-L. Zhou, Highly enantioselective carbene insertion into N-H bonds of aliphatic amines. *Science* **366**, 990–994 (2019).

15. Y. Zhu, X. Liu, S. Dong, Y. Zhou, W. Li, L. Lin, X. Feng, Asymmetric N-H insertion of secondary and primary anilines under the catalysis of palladium and chiral guanidine derivatives. *Angew. Chem. Int. Ed. Engl.* **53**, 1636–1640 (2014).

16. Q. M. Kainz, C. D. Matier, A. Bartoszewicz, S. L. Zultanski, J. C. Peters, G. C. Fu, Asymmetric copper-catalyzed C-N cross-couplings induced by visible light. *Science* **351**, 681–684 (2016).

17. C. Chen, J. C. Peters, G. C. Fu, Photoinduced copper-catalysed asymmetric amidation via ligand cooperativity. *Nature* **596**, 250–256 (2021).

18. J.-J. Chen, J.-H. Fang, X.-Y. Du, J.-Y. Zhang, J.-Q. Bian, F.-L. Wang, C. Luan, W.-L. Liu, J.-R. Liu, X.-Y. Dong, Z.-L. Li, Q.-S. Gu, Z. Dong, X.-Y. Liu, Enantioconvergent Cu-catalysed *N*-alkylation of aliphatic amines. *Nature* **618**, 294–300 (2023).

19. A. Trowbridge, S. M. Walton, M. J. Gaunt, New strategies for the transition-metal catalyzed synthesis of aliphatic amines. *Chem. Rev.* **120**, 2613–2692 (2020).

20. A. J. Musacchio, B. C. Lainhart, X. Zhang, S. G. Naguib, T. C. Sherwood, R. R. Knowles, Catalytic intermolecular hydroaminations of unactivated olefins with secondary alkyl amines. *Science* **355**, 727–730 (2017).

21. R. Kumar, N. J. Flodén, W. G. Whitehurst, M. J. Gaunt, A general carbonyl alkylative amination for tertiary amine synthesis. *Nature* **581**, 415–420 (2020).

22. X. Zhang, R. T. Smith, C. Le, S. J. McCarver, B. T. Shireman, N. I. Carruthers, D. W. C. MacMillan, Copper-mediated synthesis of drug-like bicyclopentanes. *Nature* **580**, 220–226 (2020).

23. F. Lovering, J. Bikker, C. Humblet, Escape from flatland: Increasing saturation as an approach to improving clinical success. *J. Med. Chem.* **52**, 6752–6756 (2009).

24. H. Jin, S. T. Kim, G.-S. Hwang, D. H. Ryu, L-proline derived bifunctional organocatalysts: Enantioselective Michael addition of dithiomalonates to trans-β-nitroolefins. *J. Org. Chem.* **81**, 3263–3274 (2016).

25. R. T. Simons, "Radical remote C–H functionalization for installation of C–C single and double Bonds," thesis, The Ohio State University, Columbus, OH (2023).

26. G. Pandey, P. Banerjee, S. R. Gadre, Construction of enantiopure pyrrolidine ring system via asymmetric [3+2]-cycloaddition of azomethine ylides. *Chem. Rev.* **106**, 4484–4517 (2006).

27. T. Hashimoto, K. Maruoka, Recent advances of catalytic asymmetric 1,3-dipolar cycloadditions. *Chem. Rev.* **115**, 5366–5412 (2015).

28. V. N. Wakchaure, B. List, Catalytic asymmetric reductive condensation of N–H imines: Synthesis of C_2-symmetric secondary amines. *Angew. Chem. Int. Ed. Engl.* **55**, 15775–15778 (2016).

29. T. C. Nugent, M. El-Shazly, V. N. Wakchaure, Ytterbium acetate promoted asymmetric reductive amination: Significantly enhanced stereoselectivity. *J. Org. Chem.* **73**, 1297–1305 (2008).

30. R. Sarges, H. R. Howard Jr., P. R. Kelbaugh, Synthesis of optically active spirohydantoins by asymmetric induction. Hydantoin formation from amino nitriles and chlorosulfonyl isocyanates. *J. Org. Chem.* **47**, 4081–4085 (1982).

31. A. G. Steinig, D. M. Spero, Highly diastereoselective addition of Grignard reagents to aliphatic, enolizable *N*-alkylketimines and 2,2-disubstituted 1,3-oxazolidines. Asymmetric synthesis of the antidepressant Cericlamine. *J. Org. Chem.* **64**, 2406–2410 (1999).

32. G. Liu, D. A. Cogan, J. A. Ellman, Catalytic asymmetric synthesis of *tert*-butanesulfinamide. Application to the asymmetric synthesis of amines. *J. Am. Chem. Soc.* **119**, 9913–9914 (1997).

33. M. T. Robak, M. A. Herbage, J. A. Ellman, Synthesis and applications of *tert*-butanesulfinamide. *Chem. Rev.* **110**, 3600–3740 (2010).

34. H.-C. Xu, S. Chowdhury, J. A. Ellman, Asymmetric synthesis of amines using *tert*-butanesulfinamide. *Nat. Protoc.* **8**, 2271–2280 (2013).

35. Z.-M. Zhang, P. Chen, W. Li, Y. Niu, X.-L. Zhao, J. Zhang, A new type of chiral sulfinamide monophosphine ligands: Stereodivergent synthesis and application in enantioselective gold(I)-catalyzed cycloaddition reactions. *Angew. Chem. Int. Ed. Engl.* **53**, 4350–4354 (2014).

36. H. Liu, G. Sun, Y. Zhang, Y. Li, B. Dong, B. Gao, Acid-catalyzed highly enantioselective synthesis of α-amino acid derivatives from sulfinamides and alkynes. *Org. Lett.* **26**, 1601–1606 (2024).

37. M. Feng, R. Tinelli, R. Meyrelles, L. González, B. Maryasin, N. Maulide, Direct synthesis of α-amino acid derivatives by hydrative amination of alkynes. *Angew. Chem. Int. Ed. Engl.* **62**, e202212399 (2023).

38. M. Feng, A. J. Fernandes, R. Meyrelles, N. Maulide, Direct enantioselective α-amination of amides guided by DFT prediction of *E/Z* selectivity in a sulfonium intermediate. *Chem* **9**, 1538–1548 (2023).

39. M. Wakayama, J. A. Ellman, Recycling the *tert*-butanesulfinyl group in the synthesis of amines using *tert*-butanesulfinamide. *J. Org. Chem.* **74**, 2646–2650 (2009).

40. V. K. Aggarwal, N. Barbero, E. M. McGarrigle, G. Mickle, R. Navas, J. R. Suárez, M. G. Unthank, M. Yar, The fate of the *tert*-butylsulfinyl auxiliary after acid-promoted cleavage—A method for recycling *t*-BuSONH$_2$. *Tetrahedron Lett.* **50**, 3482–3484 (2009).

41. B.-L. Chen, B. Wang, G.-Q. Lin, Highly diastereoselective addition of alkynylmagnesium chlorides to *N*-*tert*-butanesulfinyl aldimines: A practical and general access to chiral α-branched amines. *J. Org. Chem.* **75**, 941–944 (2010).

42. L. Cheng, L. Liu, Y. Sui, D. Wang, Y.-J. Chen, Highly diastereoselective reactions of 2-lithiated indoles with chiral *N*-*tert*-butanesulfinyl aldimines for the synthesis of chiral (2-indolyl) methanamine derivatives. *Tetrahedron: Asymmetry* **18**, 1833–1843 (2007).

43. G. Li, X. Xu, H. Tian, X. Liu, W. Chen, X. Yang, H. Zhang, Asymmetric synthesis of δ-amino acid derivatives via diastereoselective vinylogous Mannich reactions between *N*-*tert*-butanesulfinyl imines and dioxinone-derived lithium dienolate. *RSC Adv.* **7**, 50822–50828 (2017).

44. A. Lahosa, T. Soler, A. Arrieta, F. P. Cossío, F. Foubelo, M. Yus, Stereoselective coupling of *N*-*tert*-butanesulfinyl aldimines and β-keto acids: Access to β-amino ketones. *J. Org. Chem.* **82**, 7481–7491 (2017).

45. Y. Li, J. Hu, Facile synthesis of chiral α-difluoromethyl amines from *N*-(*tert*-butylsulfinyl) aldimines. *Angew. Chem. Int. Ed. Engl.* **44**, 5882–5886 (2005).

46. Q. Chen, C. Yuan, A new and convenient asymmetric synthesis of α-amino- and α-alkyl-α-aminophosphonic acids using *N*-*tert*-butylsulfinyl imines as chiral auxiliaries. *Synthesis* **2007**, 3779–3786 (2007).

47. H. M. Peltier, J. A. Ellman, *N*-sulfinyl metalloenamine conjugate additions: Asymmetric synthesis of piperidines. *J. Org. Chem.* **70**, 7342–7345 (2005).

48. N. Yisimayili, H. Liu, Y. Yao, C.-D. Lu, Stereodivergent construction of vicinal acyclic quaternary–tertiary carbon stereocenters by Michael-type alkylation of α,α-disubstituted *N-tert*-butanesulfinyl ketimines. *Org. Lett.* **23**, 7450–7455 (2021).

49. S. Zhao, R. B. Andrade, Development and scope of the arene-fused domino Michael/ Mannich reaction: Application to the total syntheses of Aspidosperma alkaloids (−)-aspidospermidine, (−)-tabersonine, and (−)-vincadifformine. *J. Org. Chem.* **82**, 521–531 (2017).

50. C. M. F. Mansson, N. Z. Burns, Aqueous amine-tolerant [2+2] photocycloadditions of unactivated olefins. *J. Am. Chem. Soc.* **144**, 19689–19694 (2022).

51. O. Delgado, A. Monteagudo, M. Van Gool, A. A. Trabanco, S. Fustero, A practical entry to β-aryl-β-alkyl amino alcohols: Application to the synthesis of a potent BACE1 inhibitor. *Org. Biomol. Chem.* **10**, 6758–6766 (2012).

52. N. A. Aslam, S. A. Babu, Direct lactonization of α-amino γ,δ-unsaturated carboxylic acid esters via olefin activation: Stereo- and regioselective production of homoserine lactone scaffolds having contiguous stereocenters. *Tetrahedron* **70**, 6402–6419 (2014).

53. C.-H. Zhao, L. Liu, D. Wang, Y.-J. Chen, Asymmetric Mannich-type reaction of a chiral *N*-(*tert*-butylsulfinyl) ketimine with imines: Application to the synthesis of chiral 1,3-diamines. *Eur. J. Org. Chem.* **2006**, 2977–2986 (2006).

54. S.-L. Cai, B.-H. Yuan, Y.-X. Jiang, G.-Q. Lin, X.-W. Sun, Asymmetric cinnamylation of *N-tert*-butanesulfinyl imines with cinnamyl acetates: Total syntheses of (+)-lycoricidine and (+)-7-deoxypancratistatin. *Chem. Commun.* **53**, 3520–3523 (2017).

55. D. M. Ballweg, R. C. Miller, D. L. Gray, K. A. Scheidt, Stereoselective synthesis of α-silylamines by the direct addition of silyl anions to activated imines. *Org. Lett.* **7**, 1403–1406 (2005).

56. T. Kochi, J. A. Ellman, Asymmetric α-alkylation of *N'*-*tert*-butanesulfinyl amidines. Application to the total synthesis of (6*R*,7*S*)-7-amino-7,8-dihydro-α-bisabolene. *J. Am. Chem. Soc.* **126**, 15652–15653 (2004).

57. J. Tanuwidjaja, H. M. Peltier, J. A. Ellman, One-pot asymmetric synthesis of either diastereomer of *tert*-butanesulfinyl-protected amines from ketones. *J. Org. Chem.* **72**, 626–629 (2007).

58. D. A. Cogan, G. Liu, J. Ellman, Asymmetric synthesis of chiral amines by highly diastereoselective 1,2-additions of organometallic reagents to *N*-*tert*-butanesulfinyl imines. *Tetrahedron* **55**, 8883–8904 (1999).

59. X. Liu, M. P. McCormack, S. P. Waters, An aza-Prins cyclization approach to functionalized indolizidines from 2-allylpyrrolidines. *Org. Lett.* **14**, 5574–5577 (2012).

60. J. F. Teichert, B. L. Feringa, Phosphoramidites: Privileged ligands in asymmetric catalysis. *Angew. Chem. Int. Ed. Engl.* **49**, 2486–2528 (2010).

61. C.-Y. Chang, T.-K. Yang, Asymmetric synthesis of ACE inhibitor-Benazepril HCl via a bioreductive reaction. *Tetrahedron: Asymmetry* **14**, 2239–2245 (2003).

62. Y. T. Chen, R. Lira, E. Hansell, J. H. McKerrow, W. R. Roush, Synthesis of macrocyclic trypanosomal cysteine protease inhibitors. *Bioorg. Med. Chem. Lett.* **18**, 5860–5863 (2008).

63. G. Liu, D. A. Cogan, T. D. Owens, T. P. Tang, J. A. Ellman, Synthesis of enantiomerically pure *N*-*tert*-butanesulfinyl imines (*tert*-butanesulfinimines) by the direct condensation of *tert*-butanesulfinamide with aldehydes and ketones. *J. Org. Chem.* **64**, 1278–1284 (1999).

64. A. S. Alshreimi, G. Zhang, E. J. Shim, D. J. Wink, L. L. Anderson, Gold-catalyzed *N*-alkenylation of isoxazolines and the use of alkenyl gold intermediates in the synthesis of 2-amino-1-pyrrolines. *ACS Catal.* **14**, 2229–2234 (2024).

65. Y. Zheng, A. Perfetto, D. Luise, I. Ciofini, L. Miesch, Direct synthesis of CF_2H-substituted 2-amidofurans *via* copper-catalyzed addition of difluorinated diazoacetone to ynamides. *Org. Lett.* **23**, 5528–5532 (2021).

66. E. Greve, S. V. Lindeman, C. Scartelli, L. Lin, R. Flaumenhaft, C. Dockendorff, Route exploration and synthesis of the reported pyridone-based PDI inhibitor STK076545. *Org. Biomol. Chem.* **18**, 6665–6681 (2020).

67. J. T. Colyer, N. G. Andersen, J. S. Tedrow, T. S. Soukup, M. M. Faul, Reversal of diastereofacial selectivity in hydride reductions of *N*-*tert*-butanesulfinyl imines. *J. Org. Chem.* **71**, 6859–6862 (2006).

68. X. Xiao, H. Wang, Z. Huang, J. Yang, X. Bian, Y. Qin, Selective diethylzinc reduction of imines in the presence of ketones catalyzed by $Ni(acac)_2$. *Org. Lett.* **8**, 139–142 (2006).

69. Y. Yang, G. B. Hammond, T. Umemoto, Self-sustaining fluorination of active methylene compounds and high-yielding fluorination of highly basic aryl and alkenyl lithium species with a sterically hindered *N*-fluorosulfonamide reagent. *Angew. Chem. Int. Ed. Engl.* **61**, e202211688 (2022).

70. A. Chelouan, R. Recio, A. Alcudia, N. Khiar, I. Fernández, DMAP-catalysed sulfinylation of diacetone-*D*-glucose: Improved method for the synthesis of enantiopure *tert*-butyl sulfoxides and *tert*-butanesulfinamides. *Eur. J. Org. Chem.* **2014**, 6935–6944 (2014).

71. L. R. Reddy, A. P. Gupta, Y. Liu, Asymmetric synthesis of α-amino acids by reduction of *N*-*tert*-butanesulfinyl ketimine esters. *J. Org. Chem.* **76**, 3409–3415 (2011).

72. Q. Wei, F. Zhang, X. Zhao, C. Wang, J. Xiao, W. Tang, Ru-catalyzed highly diastereoselective hydrogenation of *N*-*tert*-butylsulfinyl ketimines for the synthesis of aryl glycine derivatives. *Org. Biomol. Chem.* **15**, 5468–5471 (2017).

73. R. A. Bauer, C. M. DiBlasi, D. S. Tan, The *tert*-butylsulfinamide lynchpin in transition-metal-mediated multiscaffold library synthesis. *Org. Lett.* **12**, 2084–2087 (2010).

74. H. A. Khan, J. A. Ellman, Asymmetric synthesis of α-aminophosphonate esters by the addition of dialkyl phosphites to *tert*-butanesulfinyl imines. *Synthesis* **45**, 3147–3150 (2013).

75. J. A. Fernández-Salas, M. M. Rodríguez-Fernández, M. C. Maestro, J. L. García-Ruano, Stereochemical aspects and the synthetic scope of the S_Hi at the sulfur atom. preparation of enantiopure 3-substituted 2,3-dihydro-1,2-benzoisothiazole 1-oxides and 1,1-dioxides. *Chem. Commun.* **50**, 6046–6048 (2014).

76. C. H. Ko, D. Y. Jung, M. K. Kim, Y. H. Kim, Asymmetric synthesis of 1,2-amino alcohols using *tert*-butanesulfinimines as chiral auxiliary. *Synlett* **2005**, 304–308 (2005).

77. W. Yan, D. Wang, J. Feng, P. Li, R. Wang, Zinc-mediated diastereoselective synthesis of 3-amino oxindoles by addition of methyl and terminal alkynes to *N*-*tert*-butanesulfinyl ketimines. *J. Org. Chem.* **77**, 3311–3317 (2012).

78. S. Yang, G. Bian, Z. Chen, X. Xia, M. Zhou, C. Cui, L. Song, Highly efficient synthesis of chiral quaternary 3-aminooxindoles promoted by zinc(ii) chloride via Et_2Zn-catalysed addition of Grignard reagents to isaltin-derived *N*-*tert*-butanesulfinyl ketimines. *RSC Adv.* **7**, 38216–38219 (2017).

79. D. J. Vyas, R. Fröhlich, M. Oestreich, Activation of the Si−B linkage: Copper-catalyzed addition of nucleophilic silicon to imines. *Org. Lett.* **13**, 2094–2097 (2011).

80. T. M. Kamenecka. P. R. Griffin, *N*-Benzylbenzimidazole modulators of PPARG. WO2013078233 A1 (2013).

81. R. Almansa, D. Guijarro, M. Yus, Synthesis of highly enantiomerically enriched amines by the diastereoselective addition of triorganozincates to *N*-(*tert*-butanesulfinyl)imines. *Tetrahedron: Asymmetry* **19**, 2484–2491 (2008).

82. N. Kurisawa, A. Iwasaki, K. Teranuma, S. Dan, C. Toyoshima, M. Hashimoto, K. Suenaga, Structural determination, total synthesis, and biological activity of Iezoside, a highly potent

Ca^{2+}-ATPase inhibitor from the marine cyanobacterium leptochromothrix valpauliae. *J. Am. Chem. Soc.* **144**, 11019–11032 (2022).

83. L. Li, X.-M. Chen, Z.-S. Wang, B. Zhou, X. Liu, X. Lu, L.-W. Ye, Reversal of regioselectivity in catalytic arene-ynamide cyclization: Direct synthesis of valuable azepino[4,5-*b*]indoles and β-carbolines and DFT calculations. *ACS Catal.* **7**, 4004–4010 (2017).

84. T.-D. Tan, X.-Q. Zhu, M. Jia, Y. Lin, J. Cheng, Y. Xia, L.-W. Ye, Stereospecific Access to bridged [n.2.1] skeletons through gold-catalyzed tandem reaction of indolyl homopropargyl amides. *Chin. Chem. Lett.* **31**, 1309–1312 (2020).

85. Z. Nairoukh, M. Wollenburg, C. Schlepphorst, K. Bergander, F. Glorius, The formation of all-*cis*-(multi)fluorinated piperidines by a dearomatization-hydrogenation process. *Nat. Chem.* **11**, 264–270 (2019).

86. C. S. Li, V. Nguyen, Transdermal administration of an Enalapril ester. WO2003022270 A1 (2003).
